# Reconstruction of the sialylation pathway in the ancestor of eukaryotes

**DOI:** 10.1038/s41598-018-20920-1

**Published:** 2018-02-13

**Authors:** Daniel Petit, Elin Teppa, Ugo Cenci, Steven Ball, Anne Harduin-Lepers

**Affiliations:** 1 Université de Limoges, Laboratoire Pereine 123, av. A. Thomas, 87060 Limoges Cedex, France; 2 Bioinformatics Unit, Fundación Instituto Leloir -IIBBA CONICET, Av. Patricias Argentinas 435, C1405BWE Buenos Aires, Argentina; 3 University of Lille, CNRS, UMR 8576 - UGSF - Unité de Glycobiologie Structurale et Fonctionnelle, F 59000 Lille, France; 4 UGSF, Bât. C9, Université de Lille - Sciences et Technologies, 59655 Villeneuve d’Ascq, France

## Abstract

The biosynthesis of sialylated molecules of crucial relevance for eukaryotic cell life is achieved by sialyltransferases (ST) of the CAZy family GT29. These enzymes are widespread in the Deuterostoma lineages and more rarely described in Protostoma, Viridiplantae and various protist lineages raising the question of their presence in the Last eukaryotes Common Ancestor (LECA). If so, it is expected that the main enzymes associated with sialic acids metabolism are also present in protists. We conducted phylogenomic and protein sequence analyses to gain insights into the origin and ancient evolution of ST and sialic acid pathway in eukaryotes, Bacteria and Archaea. Our study uncovered the unreported occurrence of bacterial GT29 ST and evidenced the existence of 2 ST groups in the LECA, likely originating from the endosymbiotic event that generated mitochondria. Furthermore, distribution of the major actors of the sialic acid pathway in the different eukaryotic phyla indicated that these were already present in the LECA, which could also access to this essential monosaccharide either endogenously or *via* a sialin/sialidase uptake mechanism involving vesicles. This pathway was lost in several basal eukaryotic lineages including Archaeplastida despite the presence of two different ST groups likely assigned to other functions.

## Introduction

Sialic acids are nine-carbon negatively charged monosaccharides deriving from neuraminic acid (5-amino-3,5-dideoxy-D-*glycero*-D-*galacto*-2-nonulosonic acid) frequently described at terminal positions of sialylated molecules of Deuterostoma, more rarely in Protostoma. Due to their terminal position and properties, sialic acids like *N*-acetyl neuraminic acid (Neu5Ac) and its deaminated form KDN (2-keto-3-deoxy-D-glycero-D-galacto-nonulosonic acid) contribute to the acidity and hydration of cell membrane glycoproteins such as mucins providing relevant glycan barrier in mucosal protection^1^. The quantity and diversity of sialic acid molecules reported in Deuterostoma has long been thought to reflect specialized cell interactions like host/pathogen and immune recognition or nerve cell function^2,3^. Sialic acids are also components of prokaryotic cell envelopes glycolipids known as lipopolysaccharides (LPS) and of capsular polysaccharides of Gram-negative bacteria including the pathogens *Escherichia coli* K1, *Haemophilus influenzae*, *Pasteurella multocida*, *Neisseria meningitidis* and *Campylobacter jejuni*^4–6^. These molecules mimic cell surface sialylated molecules commonly found in humans thereby escaping the immune system and bactericidal activities of neutrophils. A number of other nonulosonic acids are described in proteobacteria among which the 5, 7-Diamino-3, 5, 7, 9-tetradeoxy-D-*glycero*-D-*galacto*-nonulosonic acid (Legionaminic acid (Leg)) and the 5, 7-Diamino-3, 5, 7, 9-tetradeoxy-L-*glycero*-L-*manno*-nonulosonic (Pseudaminic acid (Pse))^7,8^, which show structural, biosynthetic and functional similarities to sialic acids. Notably, they are implicated in cell motility, confer bacterial virulence and are thought to have important protection role in harsh marine environments. From an evolutionary point of view, sialic acids show discontinuous distribution across lineages and seemed to appear relatively late in opisthokonts^9,10^ although they were also described in some pathogenic fungi^11,12^; They are notably absent in plants^13,14^, in Archaebacteria or in the Ecdysozoa *Caenorhabditis elegans*, whereas Kdo (3-deoxy-D-manno-oct-2-ulosonic acid), an eight-carbon keto-sugar structurally related to sialic acids is widely described in Gram-negative bacteria and plants as a component of bacterial LPS and of rhamnogalacturonan-II (RG-II) in the cell wall of plants^15^. Therefore, it has been proposed that nonulosonic acids and sialylated molecules could be opisthokonts innovations that have evolved in the last common ancestor of Deuterostoma^16^ and that their presence in microbes would result of either lateral gene transfers (LGT) from opisthokonts to Bacteria or convergent evolution from microbial biosynthetic pathway^17,18^, although it still remains a matter of debate^19^.

**Figure 1:**
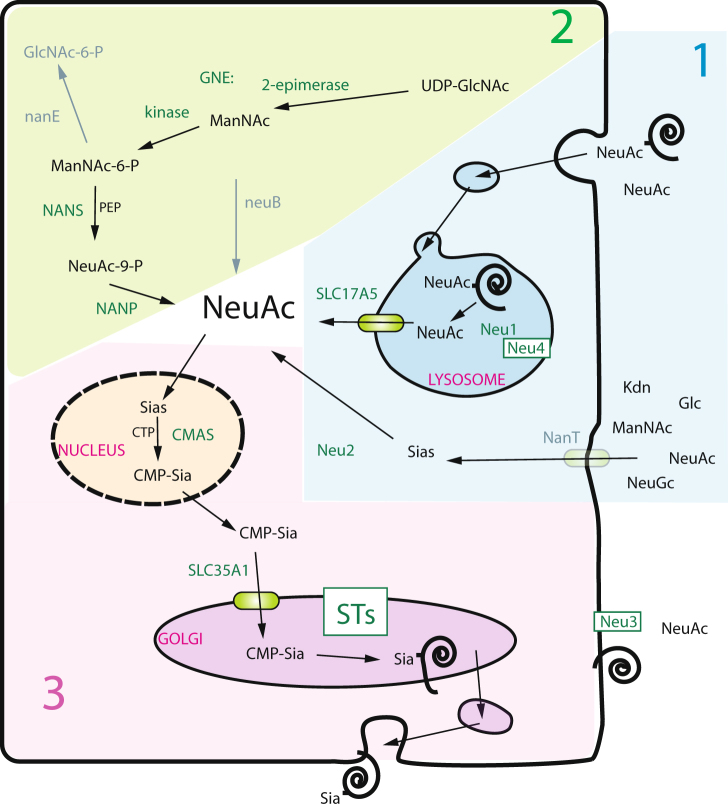
Schematic representation of the sialic acid metabolism pathway in eukaryotic cells. The key steps of the biosynthesis of sialic acid and its transfer and removal from sialoglycoconjugates is depicted (pink background). Cytosolic sialic acid molecules in eukaryotic cells, originate either from (1) exogenous sialyloglycoconjugates *via* the lysosome after Neu1 and SLC17A5 action or *via* an as yet unknown mechanism of uptake at the plasma membrane (blue background) or (2) from a cytosolic UDP-GlcNAc molecule biosynthesized in the hexosamine pathway (green background). Abbreviations used are indicated as follows: GNE: UDP-GlcNAc 2-epimerase/ManNAc kinase (the 2 enzymatic domains are fused in Deuterostoma); NANS: Neu5Ac-9-phosphate synthetase also known as *N*-acetylneuraminate lyase; NANP: Neu5Ac-9-phosphate phosphatase; CMAS: CMP-Sialic acid synthetase; STs: Golgi sialyltransferases; Neu1–4: sialidase 1–4; Sia: sialic acid; CMP: cytidine monophosphate; CTP: cytidine triphosphate; PEP: phosphoenolpyruvate; SLC17A5: sialin; SLC35A1: CMP-sialic acid transporter. Key steps in *N*-acetylneuraminate biosynthesis in Bacteria are indicated in grey characters.

Among the various enzymatic actors involved in sialylation reactions (Fig. 1), sialyltransferases (ST) are grafting sialic acids from an activated sugar donor (CMP-sialic acid) to a variety of oligosaccharides found on glycoproteins and glycolipids. These type II membrane proteins primarily found in the Golgi apparatus of eukaryotic cells^20^ have pivotal roles in the biology of all cells and the cognate ST-related genes were predicted to be instrumental in Deuterostoma evolution^16,21–23^. In Bacteria, several ST have been biochemically characterized^24,25^. The known bacterial ST belong to different families of the Carbohydrate-Active-enZYmes (CAZy) database^26^ where the biosynthetic enzymes are classified according to their enzymatic activity and structure: ST of the CAZy family GT38, GT52^27^ and GT 80^28^ are ST with a Rossman-like GTB fold^29,30^, those of the recently described CAZy family GT97 are bi-functional UDP-Gal transferase/CMP-NeuAc transferase like the SiaD/W of *N*. *meningitidis*^31^. On the other hand, ST of the CAZy family GT42 show a GTA-variant2 fold^32,33^. These bacterial ST protein sequences contain two short peptide motifs (D/E)-(D/E)-G and HP motif towards their C-terminus that are functionally important for enzyme catalysis and substrates binding^34–36^. However, no common consensus peptide motifs could be found between bacterial and eukaryotic ST sequences indicating their divergence. Noteworthy, the CMP-Kdo transferases mainly described in plants and bacteria belong to different CAZy families suggesting their different evolutionary origin: GT30 encompasses CMP-Kdo transferases with a predicted GTB fold, whereas GT73 groups CMP-Kdo transferases with a GTA fold^34^ and the newly reported GT99 CAZy family shows CMP-Kdo transferases like the *Raoultella terrigena* WbbB catalyzing the transfer of β–Kdo on LPS O-antigen^37^. Up to now, all the eukaryotic ST have been classified as inverting enzymes in the CAZy family GT29, which denotes their common modular organization (GT-A-like fold) and their common ancestral origin. We reported the existence of 20 paralogous ST genes in the human genome^38^. Furthermore, the cognate enzymes are organized in four families, namely the ST3Gal, ST6Gal, ST6GalNAc and ST8Sia according to the glycosidic linkage formed and the monosaccharide acceptor used^39^ and are highly specific for the donor and acceptor substrates. All these enzyme sequences share a series of four conserved peptide motifs known as sialylmotifs, which serve as hallmark for their identification in databases^40^. These conserved motifs, namely the Large (L), Short (S), III, Very Short (VS) sialylmotifs are involved in the binding of the ST substrates and in their catalytic function^41^. Furthermore, family motifs characteristic of each individual family have been identified more recently^40,42^. Outside the vertebrate lineages, we have retrieved several GT29 ST sequences in invertebrate Deuterostoma, such as the sea Urchin *Strongylocentrotus purpuratus* for ST8Sia^21^, for ST3Gal^23^, or for ST6GalNAc^39^, and the hemichordate *Saccoglossus kowalesvskii* for ST6Gal^22^. In the invertebrate Protostoma, only Arthropods show members of the GT29 restricted to the ST6Gal family. We recently described in the sponge *Oscarella carmela* a sequence orthologous to the common ancestor of the vertebrate ST3Gal I, ST3Gal II and ST3Gal VIII^23^, whereas no GT29 ST-related sequence could be identified in Fungi. Interestingly, green plants have been shown to harbor ST-like sequences sharing most of the conserved sialylmotifs. In *Arabidopsis thaliana*, a sequence possesses all the sialylmotifs, whereas the two others lack the sialylmotifs III and VS^39^. These plant GT29 ST-like sequences have been proposed to catalyze the transfer of Kdo and/or of 2-keto-3-deoxylyxo-heptulosaric acid (Kdh) on RG-II and to be required for proper pollen tube elongation^43–45^. Interestingly, 69 ST-related sequences exhibiting conserved sialylmotifs were recently identified in the Prasinophyta *Bathycoccus prasinos*^46^ raising the possibility that these enzymes and the sialylation pathway could have appeared much sooner than anticipated in the Last Eukaryotes Common Ancestor (LECA) prior the separation of unikonts (Amorphea/opisthokonts) and bikonts (Diaphoretickes/Archaeplastida)^47^.

**Figure 2:**
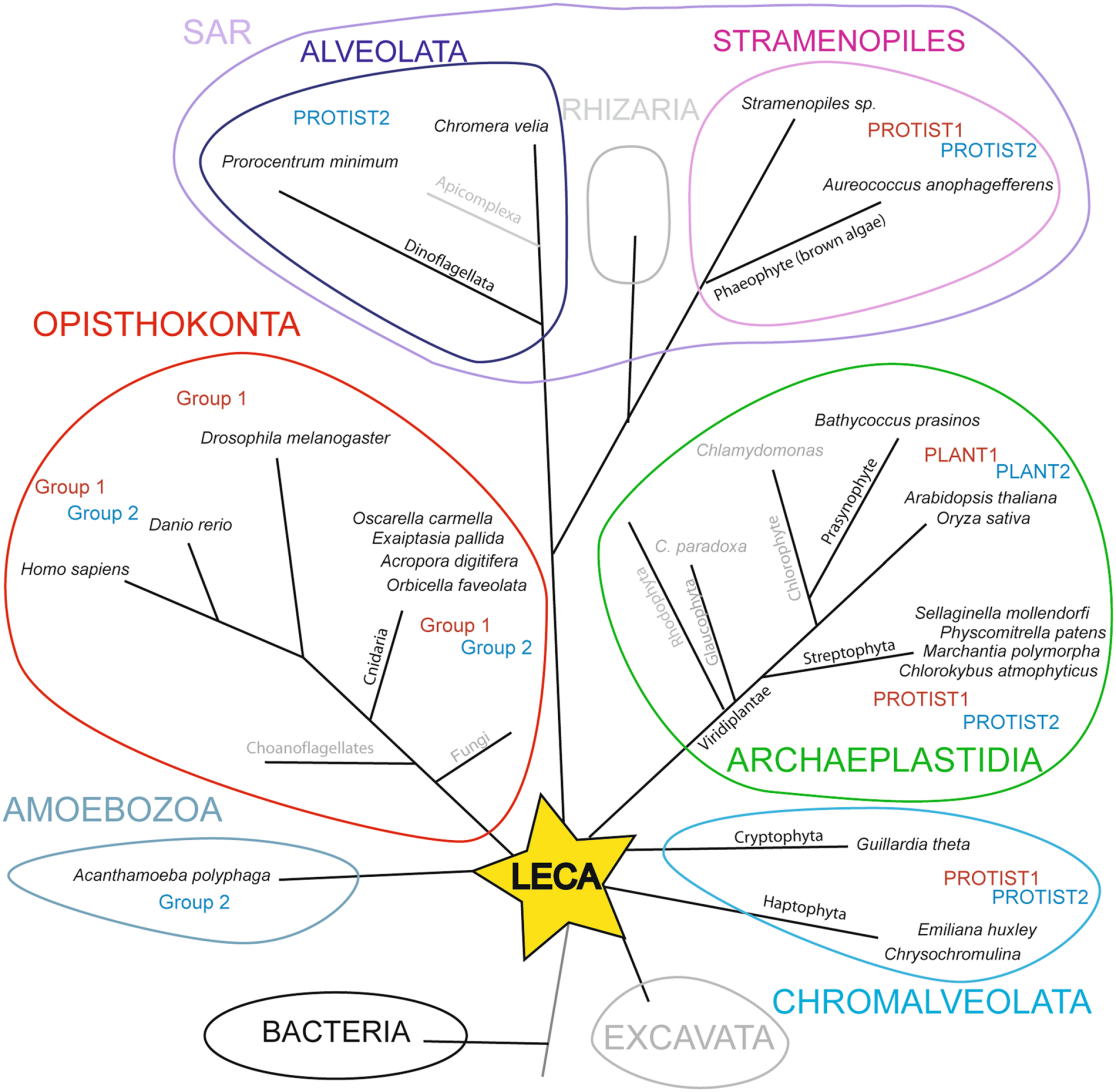
Schematic phylogenetic tree showing the GT29 ST-related sequences distribution in eukaryotes. The figure was constructed using the NCBI taxonomy browser (http://www.ncbi.nlm.nih.gov/Taxonomy) and the data from^47^. The star-like phylogenetic tree indicates the six monophyletic groups of eukaryotes *i*.*e*. opisthokonts (red), SAR (pink), Archaeplastida (green), Hacrobia (blue) and Excavata (grey). Branches length is not drawn to scale. Those organisms showing one or more GT29 ST sequences are indicated in black whereas lineages that have lost ST sequences are indicated in grey. Affiliation of ST sequences to one phylogenetic group or the other (see Fig. 3) is indicated. Abbreviations used: LECA: Last eukaryotes common ancestor; SAR: Stramenopiles, Alveolata, Rhizaria.

This patchy distribution in eukaryotes raises the question of the evolutionary origin of the sialylation machinery and sialic acids. As a first step to tackle this issue, we systematically explored databases of newly sequenced protists, Archaea and Bacteria genomes for GT29 ST-related sequence identification based on sialylmotifs detection. We identified several previously unreported GT29 ST sequences in protists and Bacteria and we inferred the somewhat distant evolutionary relationships between the well-studied opisthokont ST sequences and the other GT29-related sequences (Fig. 2). Secondly, to give an overall framework of this ST-gene family evolution, we also extended our phylogenetic analysis to the evolutionary history of the main enzymes involved in the nonulosonic acid metabolism across the different phyla of eukaryotes^47,48^. We also questioned whether these actors were already present in the LECA or progressively gained in eukaryotes through LGT from Bacteria and/or successive LGT between protists. In this study, we gained strong evidences that the sialic acid metabolic pathway can be traced back to the first eukaryotes, even though many phyla have lost mandatory enzymes, like GT29 ST in Fungi and several lineages of Metazoa like worms and mollusks, or the possibility to activate the sialic acid into cytidine monophosphate sialic acid (CMP-sialic acid) in Archaeplastida.

## Results and Discussion

### Sialyltransferase-like sequence distribution in eukaryotes

To identify ST-related sequences and assess their distribution in eukaryotes, we used sequence similarity approach with Basic Local Alignment Search Tool (BLAST)^49^ in various eukaryotic databases described in the material and method section. In addition, we used Hidden Markov Model (HMM)-based search with the known Pfam domain PF00777^50–52^ and a HMM profile of sialylmotifs conserved in all the animals ST sequences of the GT29 CAZy family according to the strategy reported in Petit *et al*.^53^. Classification of eukaryotes encompasses at least 6 super-groups with (1) opisthokonts (Metazoa, Fungi and Choanomonada), (2) Amoebozoa, (3) Excavata, (4) Hacrobia (Haptophyta and Cryptophyceae), (5) Archaeplastida (Glaucophyta, Rhodophyceae and Chloroplastida, which are red and green algae, and plants) and (6) SAR (Stramenopiles, Alveolata, Rhizaria) although deep relationships remain to be established as for Hacrobia and Excavata^47^. Therefore, we have designed here these unicellular organisms emerging at the base of the eukaryotic phylogenetic tree as protists. As illustrated in Fig. 2, our exploration of the databases led us to take into account 1 GT29 ST sequence of the Archaea *Candidatus Methanomethylophilus alvus* a methanogen present in the human gut^54^, 19 from protists (Hacrobia (Cryptophyceae and Haptophyta) and SAR (Alveolata and Stramenopiles)), 30 sequences from Archaeplastida and 106 from opisthokonts genome^47^ (supplemental data 1). One partial ST-related sequence was identified in an Amoebozoa genome, but was not used for phylogenetic analysis. Unexpectedly, we identified several GT29 ST-like sequences from Alpha-, Gamma-, and Epsilon-Proteobacteria (α-, γ- and ε-Proteobacteria) and 21 of these sequences were considered in this study. The accession numbers and phylogenetic distribution in the genome of diverse eukaryotes and Bacteria are gathered in supplemental data 2 Table 1.

As observed before in Metazoa, ST sequences show a patchy distribution in eukaryotic genomes^19^; Even though homologues of GT29 ST are widespread among the three domains of life, no ST-related sequence was found in the premetazoan genomes of Choanoflagellata nor in Fungi. Intriguingly, the ST gene copy number was found to be highly expanded in some eukaryotic genomes as in the *B*. *prasinos* genome^46^ or of the unicellular opisthokonts *O*. *carmela*, which contains a large number of ST-related sequences. It is interesting to note that most of these organisms are marine organisms among which, a number of Gram-negative marine bacteria like *Alteromonas* or *Idiomarina* of the γ-proteobacteria phylum or *Loktanella* of the α-proteobacteria phylum (supplemental data 2). The LPS macromolecule, the major charged component of the outer membrane of these Gram-negative bacteria is exposed towards the external environment and is prone to structural changes offering protection against harsh marine environment. Interestingly, the core region of LPS contains three ulosonic acids with 2 Kdo residues one of which is carrying a neuraminic acid residue^55,56^.

### Conserved peptide motifs: the sialylmotifs

**Figure 3:**
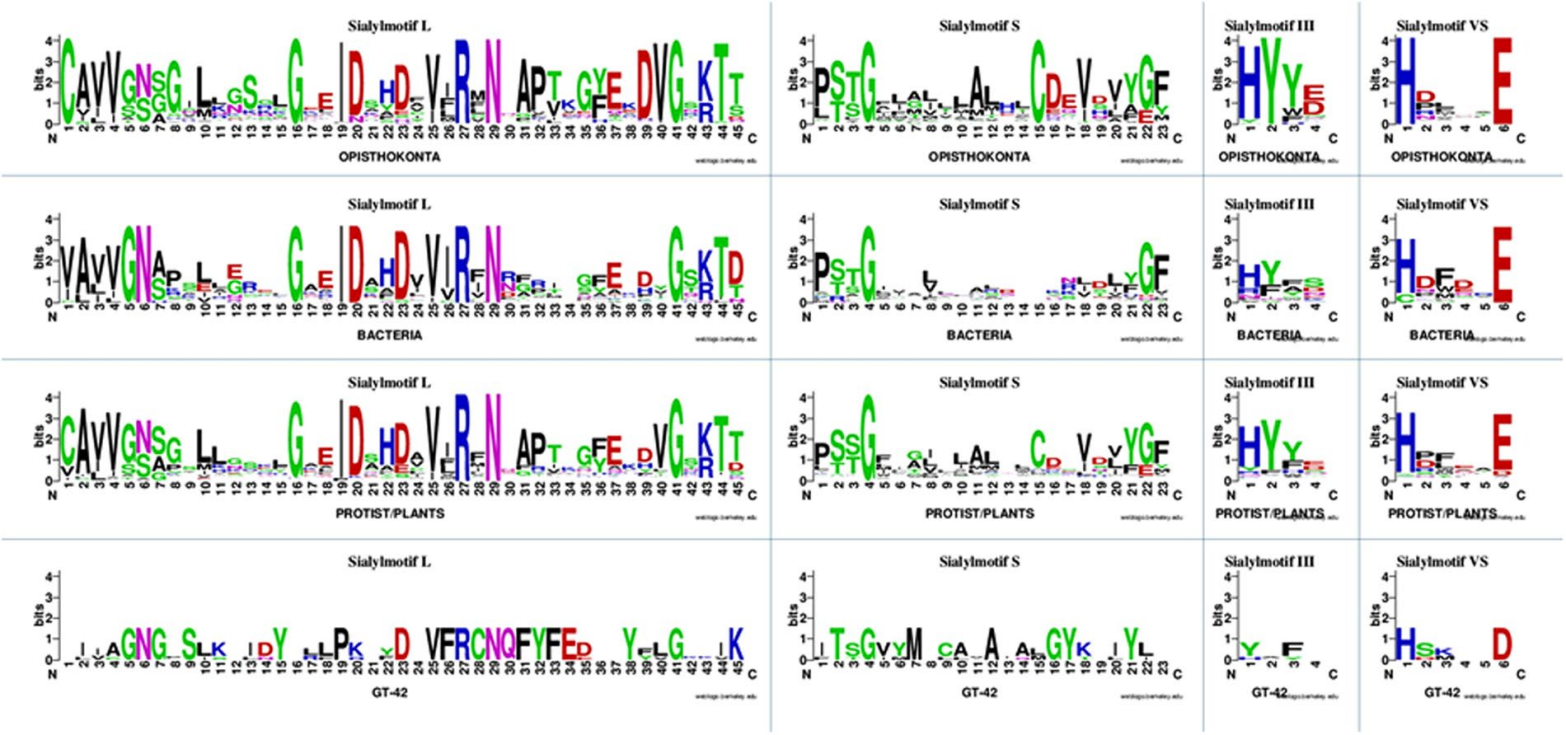
Sequences logos of the ST conserved motifs. Four multiple sequence alignment of all the ST sequences corresponding to 102 opisthokonts, 23 Bacteria, 119 protists/Archaeplastida GT29 ST and 5 ST of GT42 CAZy family sequences was carried out with MUSCLE in MEGA7.0.18^64^ (Supplemental data 1). The informative region used was restricted to the ST catalytic domain encompassing the 4 conserved sialylmotifs *i*.*e*. L (39–45 aa), S (21–23 aa), III (1–4 aa) and VS (1–6 aa) as defined previously^40^. Sequence logos of each sialylmotif in the 4 groups of ST sequences show sequence conservation as the overall height of the stack and the relative frequency as the height of symbols within the stack. Symbols are colored according to their chemical properties polar amino acids (G, C, S, T, Y) are green, basic (K, R,H) are blue, acidic (D, E) are red, hydrophobic (A, V, L, I, P, W, F, M) are black and neutral polar amino acids (N, Q) are pink^95,96^.

Although GT29 ST proteins found in opisthokonts show little overall sequence identity, analysis of multiple sequence alignments (MSA) has led to the identification of conserved sialylmotifs L, S, III and VS in the catalytic domain of ST that are structural and functional signatures^40,57–60^. We carried out comparative protein sequence analysis and constructed MSA with the 180 newly identified GT29 ST sequences. We considered 2 eukaryotic groups, *i*.*e*. opisthokonts (102 sequences) and Archaeplastida and protists (119 sequences) and 2 bacterial groups, *i*.*e*. 23 GT29 ST bacterial sequences and 5 bacterial sequences of the phylogenetically related GT42 ST (Supplemental data 1). Figure 3 depicts the first level of amino acid conservation pattern, with sialylmotifs L, S, III and VS being retrieved in the catalytic domain of Archaeplastida and Bacteria GT29 ST sequences, which further suggests a common evolutionary route for these newly identified ST sequences. Furthermore, fully conserved amino acid (aa) positions are indicative of important aa residues to maintain the structure and protein function and their persistence along evolution suggests strong evolutionary pressure. Conversely, these sialylmotifs are rather weakly conserved in the Bacteria ST of the more distantly related GT42 family^61^ (Fig. 3), although this observation should be taken with care due to the low number of GT42 sequences. Interestingly, the transmembrane domain and the conserved disulfide-bound cysteine residues in sialylmotif L and S of opisthokonts ST^62,63^ are not found in Bacteria GT29 ST sequences suggesting different topology of these bacterial proteins.

### Molecular phylogenetic analysis of eukaryotic ST

**Figure 4:**
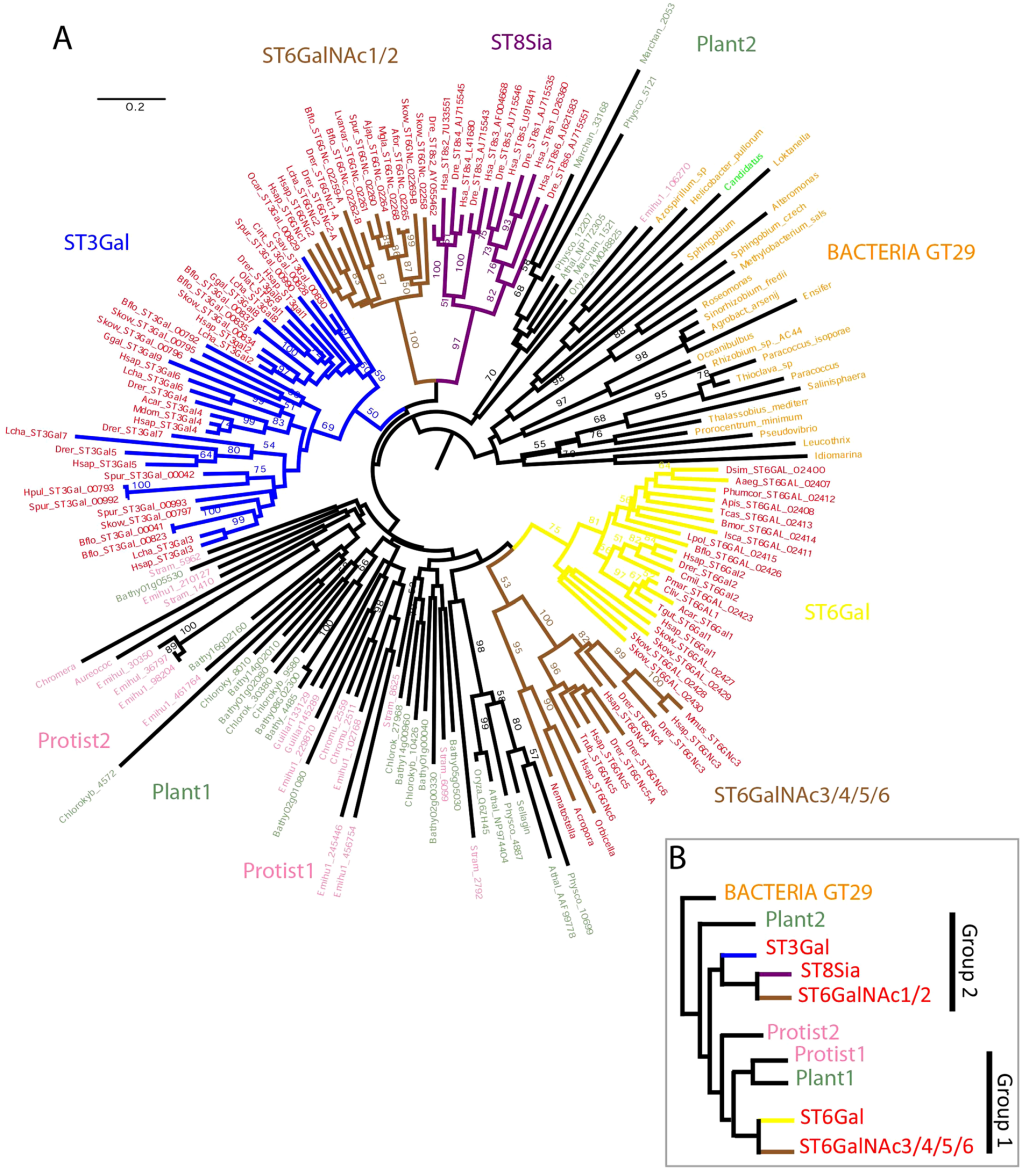
Minimum Evolution phylogenetic tree of 180 ST of the GT29 CAZy family. (**A**) The evolutionary history of the 180 ST of the GT29 CAZy family was inferred using the Minimum Evolution (ME) method. The optimal tree with the sum of branch length = 58.17312355 is shown. The tree is drawn to scale, with branch lengths in the same units as those of the evolutionary distances used to infer the phylogenetic tree. The evolutionary distances were computed using the JTT matrix-based method and are in the units of the number of amino acid substitutions per site. The ME tree was searched using the Close-Neighbor-Interchange (CNI) algorithm at a search level of 1. The Neighbor-joining algorithm^97^ was used to generate the initial tree. The analysis involved 180 ST amino acid sequences and all positions with less than 95% site coverage were eliminated. There were a total of 92 aa positions in the final dataset. Evolutionary analyses were conducted in MEGA7^64^. Bootstrap values were calculated from 350 replicates and values greater than 50 are reported. The tree is midpoint rooted (**B**) Schematic ST tree summarizing the distribution of the eukaryotic ST sequences in two major groups.

Firstly, we conducted MSA with the 180 selected GT29 ST protein sequences using MUSCLE algorithm in the MEGA7.0 software^64^ and refined MSA by hand. An informative region of ~92 aa residues (74–92 aa) was considered in the ST catalytic domain encompassing the four sialylmotifs L, S, III and VS and the family motif a (supplemental data 1). In the phylogenetic tree obtained, sequences are distributed in a non-rooted manner and comprise 10 clusters in a star-like shape (Fig. 4A): five clusters were found uniquely in Metazoa, among which ST6Gal and ST6GalNAc3/4/5/6 form a group (Group 1), and ST3Gal, ST6GalNAc1/2 and ST8Sia another one (Group 2). The monophyly of these families is supported by bootstrap values ranging from 36 (ST3Gal) to 99 (ST6GalNAc1/2). The remaining clusters have very low bootstrap values, as a result of weak variation amounts from the center of the star. It is interesting to note that the ST6GalNAc family appears to be composed of two unrelated groups of sequences, ST6GalNAc1/2 close to ST3Gal and ST8Sia families on the one hand and ST6GalNAc3/4/5/6 close to ST6Gal family on the other (Fig. 4B). Likewise, the Archaeplastida GT29 ST-like sequences are distributed in two independent groups (Group 1 and Group 2, in Fig. 4B) well supported by high bootstrap values (98% in both cases). Interestingly, these bacteria GT29 ST-like sequences constitute a basal group in the GT29 ST phylogenetic tree with sequences from a Dinoflagellata (*Prorocentrum minimum*) and an Archaea (*Candidatus Methanomethylophilus*) included inside, suggesting two different LGT events from Bacteria. To further test this assumption, we rooted the tree, with the phylogenetically related bacterial ST of the CAZy family GT42 used as an outgroup^61^. This GT42 CAZy family includes the bacterial ST named Cst I and Cst II, which are α2,3-ST and multifunctional α2,3/8-ST^33,65^ respectively from *C*. *jejuni*, and the multifunctional α2,3/8-ST Lic3B from *Haemophilus influenza*^66^ with a predicted GTA-variant fold^34^ (Supplemental data 2). We carried out MSA with MUSCLE in MEGA 7.0 software^64^ in the informative region comprised of ~92 aa residues defined previously (Supplemental data 1). The newly obtained phylogenetic tree by the ME using JTT model shows a GT42 ST group at the base of the tree and an almost identical distribution of the GT29 ST sequences. The two eukaryotic ST groups share the same contents although ST8Sia position is no longer associated to ST6GalNAc1/2, but is associated with Plant2, close to the base of group 2. Despite the weak changes with this new rooting, bacterial GT29 ST sequences appear to be the best outgroup for the eukaryotic GT29 ST sequences. Our data further points to a bacterial origin of the GT29 ST (Supplemental Figure S1) that were transferred from Bacteria to eukaryotes, either i) directly in the LECA or ii) in an eukaryote and and then transferred several times by LGT between eukaryotes, as previously reported for other genes^18,67,68^. This later hypothesis could explain that most of the protists acquired the GT29 ST-like sequences through endosymbiotic gene transfer from an Archaeplastida. However, we favor the first hypothesis since the sequence of Viridiplantae and protists on one hand and those of opisthokonts on another hand seems intertwined in the phylogenetic tree (Fig. 4A), indicating a common origin followed by duplication and subfunctionalization. This view is supported by the nature of bacteria showing GT29 ST-related sequences. Of the 23 bacterial sequences, 19 are from α-proteobacteria. Given the massive introduction of α-proteobacterial genes in the first eukaryotes that resulted in mitochondrial incorporation event^69^, we hypothesize that the eukaryotic GT-29 ST are linked to this endosymbiotic event. There are two clusters containing most protist GT29 ST sequences, rooted near the bacterial cluster: protist 2 takes place near the ST6GalNAc1/2-ST8Sia groups, whereas protist 1 is closer to the ST6Gal-ST6GalNAc3/4/5/6 group. Protist 2 group contains *Emiliana huxleyi* and *Chrysochromulina* sequences, and protist 1 group has a richer repertoire of species: *Stramenopiles* sp., *E*. *huxleyi*, *B*. *prasinos*, *Chlorokybus atmophyticus*, *Aureococcus anophagefferens*, *Guillardia theta*, and *Chromera velia*. There is a small series of protist sequences (*B*. *prasinos* and *G*. *theta*) more or less connected to Bacteria and Plant 2 clusters. Assuming that the ST phylogenetic tree is rooted by the bacterial ST cluster, the ST world appears to be divided in two in eukaryotes (Fig. 4B), although Plant 2 cluster and more or less allied protist sequences remain difficult to position. Nevertheless, the five metazoan families of ST have no counterparts in protists, Archaeplastida, or in non-eukaryotic organisms. The basal position of most of these taxa indicate that the differentiation between ST8Sia, ST6GalNAc1/2 and ST3Gal is at least older than the emergence of Sponges (951 Mya), and that the split between ST6Gal and ST6GalNAc3/4/5/6 dates back before the emergence of Cnidaria (824 Mya). Given the weak statistical support, it is difficult to assign accurately the protist clusters from which the metazoan ST sequences would have evolved from. There have been several rounds of ST duplication in protists as the species can occur in the different clusters, *e*.*g*. *E*. *huxleyi* in protist 1, protist 2 and *G*. *theta* in protist 1 and the group close to Plant 2 (Fig. 4).

### Bayesian inference and maximum likelihood with mixture models assess ancestrality of GT29 in eukaryotes

Since Bayesian inference and Maximum Likelihood (ML) using mixture model allows decreasing artefacts due to long branch attraction^70–73^, we also used newly developed models to perform phylogenetic analysis of the GT29 ST either rooted with or without the bacterial GT42 ST (Supplemental Figures S2 and S3). For those two datasets, we built a tree using the CAT-GTR^72^ model with Phylobayes 4.1^74^ and 100 bootstrap replicates were performed using the LG4X^71^ model using IQ-TREE^75^. The phylogenetic trees obtained confirmed the analyses performed with the JTT models (Fig. 4 and Supplementary Figure S1), displaying Bacteria as the putative source for this protein in the different eukaryotes. However, if mixture models reinforce the hypothesis that the ST of the GT29 CAZy family were inherited from bacteria in eukaryotes, we were not able to greatly improve node values for a better understanding of the relationships between the different functions. In addition, the trees did not allow us to predict function of those enzymes in unicellular eukaryotes or in Bacteria. These phylogenetic trees also indicated that the GT29 ST could have been transmitted among unicellular eukaryotes through a series of plastid endosymbiosis, since all the eukaryotes bearing ST GT29 found until now are plastid bearing organisms, except for opisthokonts^76^. Moreover, the topology of the different trees also indicated a relationship between Archaeplastida clades and the Alveolata (represented by *C*. *velia*), Haptophyta (represented by *E*. *huxleyi*), Stramenopiles (represented by *A*. *anophagefferens*) or Cryptophyceae (represented by *G*. *theta*).

### Sequence similarity networks using Cytoscape

**Figure 5:**
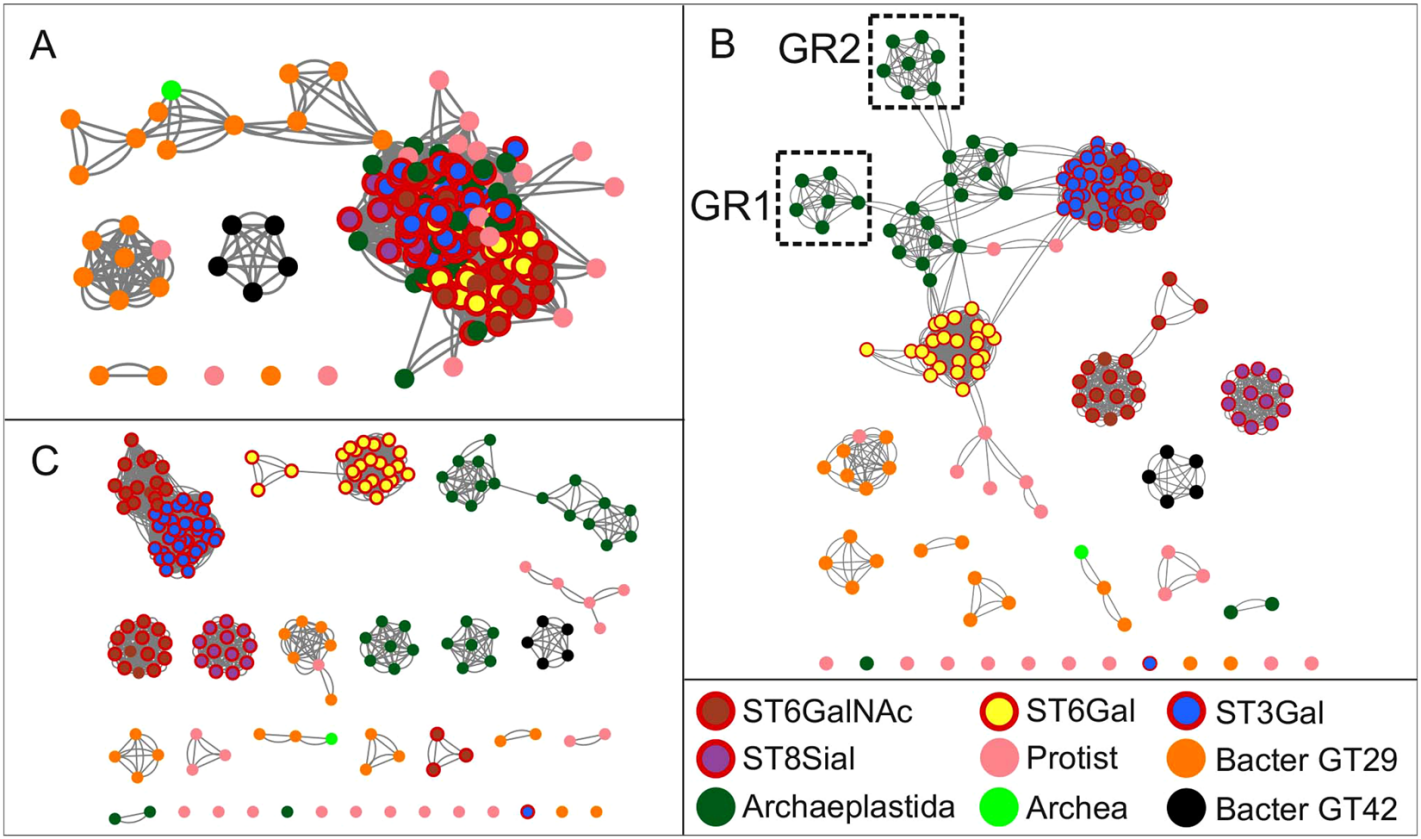
Sequence similarity network of the GT29 CAZy family. Each node represents a sialyltransferase protein sequence and the edges (lines) between nodes represent their pairwise relationships. The color of the node indicates the phylum where the protein is from (brown: ST6GalNAc, yellow: ST6Gal, blue: ST3Gal, purple: ST8Sia, pink: protist ST, orange: Bacterial GT29, green: Archaeplastida, fluorescent green: Archaea and black: Bacterial GT42. The nodes representing ST sequences from opisthokonts are highlighted with a red border). The same network is shown at three different thresholds, varying the cutoff value from permissive to stringent. (**A**) Similarity Network using a permissive threshold (E-value = 5E-15). Most of the sequences form a big cluster, with the exception of GT29 and GT42 bacterial sequences that form distinct clusters. (**B**) Network using a more stringent thresholds (E-value = 5E-23) nodes are associated with more significant relationships. Sequences break out into connected distinct clusters. Archaeplastida sequences form distinct clusters, where two of them correspond to GR1 and GR2 groups. The Archaea sequence (fluorescent green) shows similarity with GT29 bacterial sequences. (**C**) At a more stringent cut-off (E-value = 5E-25) sequence s of the GR1 and GR2 groups are disconnected from the other Archaeplastida sequences. Protist sequences do not form pure cluster, and at stringent thresholds sequences tends to break out into small and disconnected clusters. This point out the relative small similarity between ST protist sequences.

We examined the similarity of full length ST sequences identified in opisthokonts, Archaeplastida and Bacteria using sequence similarity networks to visualize relationships across these various ST groups. Sequence similarity network shown in Fig. 5 recapitulates the information obtained in phylogenetic trees. Using a permissive threshold (E-value = 5E-15), GT42 and bacterial GT29 ST sequences form distinct clusters showing that they are the most diverse sequences in the data set. This analysis shows also that the protist and Archaea ST sequences are related to bacterial GT29 ST sequences (Fig. 5A), whereas the bacterial sequences of GT42 family form a distinct cluster without connection to any other sequences. This latter cluster remains unchanged at the different tested cut-off values indicating a high degree of similarity within the GT42 family and the low similarity between GT42 sequences and the rest of the ST-related sequences. In the network generated using a more stringent cut-off (E-value = 5E-23), the Archaeplastida ST sequences break out into distinct clusters, where two of them noted GR1 and GR2 correspond to the two groups defined in phylogenetic analysis (Fig. 4B). Of particular interest, ST8Sia and ST6Gal sequences found in opisthokonts form two distinct clusters, whereas ST6GalNAc sequences are split in 2 clusters, one of them in close relationship with ST3Gal sequences. ST6GalNAc1 and ST6GalNAc2 sequences have a higher degree of similarity with ST3Gal sequences than the rest of the ST6GalNAc sequences (*i*.*e*. ST6GalNAc3, ST6GalNAc4, ST6GalNAc5 and ST6GalNAc6) suggesting either a common origin or convergent evolution for these ST sequences. At this threshold, 9 protist ST sequences are disconnected from any node and the remaining protist ST sequences belong to small and dispersed clusters showing the comparatively low degree of similarity between them (Fig. 5B). Finally, using a more restrictive threshold (E-value = 5E-25), ST sequences break into pure clusters with the exception of the clusters of ST3Gal and ST6GalNAc1/2 sequences. The GR1 and GR2 groups are disconnected from the other Archaeplastida ST sequences. The Archaea ST sequence shows similarity between two GT29 bacterial ST sequences corresponding to *Azospirillum* and *Helicobacter* organisms (Fig. 5C). This sequence similarity network analysis also support the assumption that the functional divergence of GT29 ST into 4 distinct families occurred essentially in the Metazoa lineage.

### Other actors of the sialylation machinery

**Figure 6:**
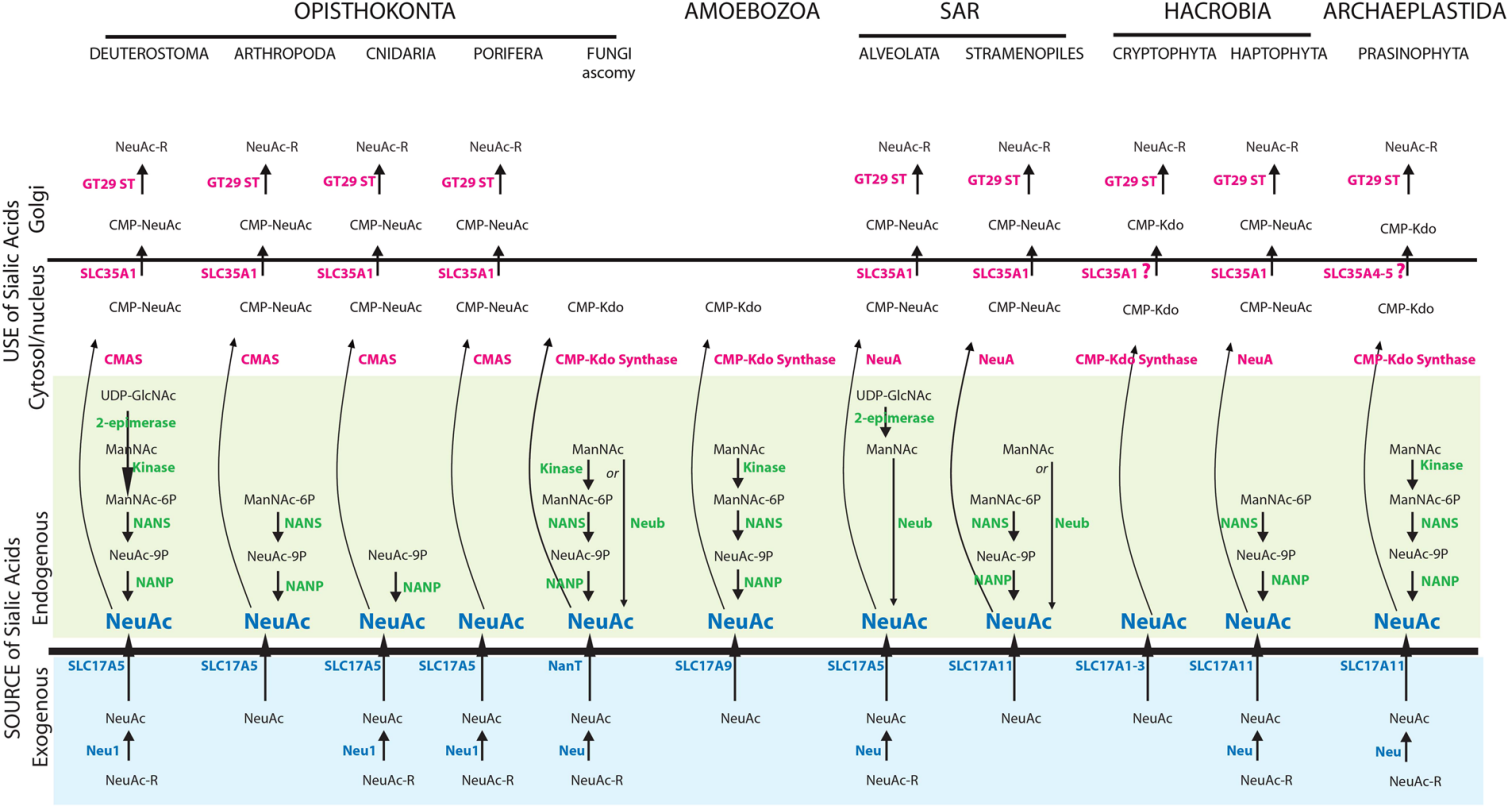
Schematic illustrating the proposed scenario of evolution of the sialylation pathway in eukaryotes. The model presented here summarizes the data presented in this study and is based on prior literature. The five eukaryotic groups of opisthokonts, Amoebozoa, SAR, Hacrobia and Archaeplastida and only a few lineages among these groups are represented. The two pathways of sialic acid synthesis represented in the Stramenopiles and Ascomycota (Fungi) exist in fact in two different species of these lineages. Of note, the NanT enzyme found in Fungi concerns essentially Basidiomycota. Abbreviations used are indicated as follows: 2-epimerase: UDP-GlcNAc-2-epimerase; CMAS: CMP-Sialic acid synthetase; ST: sialyltransferases; Neu1: sialidase 1; NANS: NeuAc-9-P synthase; NANP: *N*-acetylneuraminate-9-phosphatase.

To conduct efficient sialylation reactions, eukaryotic Golgi GT29 ST enzymes should have access to an activated sugar-donor *i*.*e*. CMP-sialic acid. For that purpose, the eukaryotic organism must dispose of either an exogenous or endogenous source of sialic acid molecules and should be able to convert sialic acid into CMP-sialic acid. Despite a good understanding of the sialic acid metabolism pathways in opisthokonts, Coelomata and Bacteria^6,77^, nothing is known in protists. To tackle this issue, we also conducted an integrated evolutionary study of this biological system leading to the synthesis of sialylated molecules at the eukaryotic cell surface. We explored the evolutionary trajectories of the major components of the sialylation pathway that could provide the eukaryotic cell with sialic acid either from the eukaryotes environment (*i*.*e*. Sialidases (Neu) and the transporters Sialin and NanT) or from endogenous sialic acid biosynthetic pathway (*i*.*e*.: UDP-GlcNAc 2-epimerase, NeuAc-9-P synthase (NANS) and *N*-acetylneuraminate-9-phosphatase (NANP)) and those molecules that provide activated sialic acid donor substrate for the Golgi GT29 ST enzymes (*i*.*e*.: the CMP-NeuAc synthase CMAS and the Golgi transporter SLC35A1). As detailed in supplemental data 4 and supplemental Figures S4–S10, our phylogenetic analysis led to the conclusion that LECA had an exogenous source of sialic acid through lysosomal Neu1 and an as yet uncharacterized sialidase in addition to transporters of SLC17A family (mainly the SLC17A5 (Sialin)) and a probably less specific transporter named SLC17A11. LECA also harbored an endogenous source of sialic acid using ManNAc as an initial substrate, and Man-kinase, NANS and NANP enzymes. In the cytosolic compartment of LECA, the CMAS enzyme could activate sialic acid molecule into CMP-sialic acid, which was then translocated into the Golgi compartment via the SLC35A1 transporter and transferred to glycoconjugates by ST GT29. Interestingly, various eukaryotic lineages showed a replacement of the canonical pathway providing sialic acid to the cell by a bacterial pathway (Fig. 6): (i) for the synthesis of Sialic acid in Alveolata, and isolated cases of Fungi and Stramenopiles, (ii) for the intake of Sialic acid in the Fungi Basidiomycota. Both systems were lost in the Streptophyta among Archaeplastida and Lophotrochozoa among Metazoa. Regarding the use of cytosolic sialic acid, the loss of the transporter SLC35A1 was associated to a replacement of CMP-NeuAc by CMP-Kdo synthase and a loss of GT29 ST (Fungi and Amoebozoa) or a shift in the function of GT29 ST to the transfer of Kdo (Archaeplastida Streptophyta, and maybe Prasinophyta and Hacrobia). In Deuterostoma, the major change concerns the possibility to start the biosynthetic pathway two steps earlier than in LECA, through an LGT of the 2-epimerase from parasitic or symbiotic Alveolata. In Vertebrata, production of CMP-Neu5Ac became progressively nuclear instead of cytosolic, although the biological significance remains to be understood.

## Conclusion

It has long been known that the oligosaccharide structure built up in the Endoplasmic Reticulum (ER) and transferred on nascent proteins in the *N*-glycosylation pathway is remarkably conserved in eukaryotes^78,79^. In addition, recent phylogenetic works performed to characterize origin and studies of the early evolution of protein *N*-glycosylation in the ER point to a mixed origin (Archaea/Bacteria) of the N-glycosylation pathway enzymes^79–81^. Terminal glycosylation of lipids and proteins (*i*.*e*. galactosylation, fucosylation and sialylation) achieved in the Golgi apparatus of land plants and animals is highly diverse and confers glycoconjugates with enormous function modularity. However, almost nothing is known of how this Golgi glycosylation machinery emerged and its early stages of evolution in eukaryotes^82–84^. In this study, we enquired about the sialylation function of the Golgi apparatus in eukaryotes. Towards this aim, we focused on ST of the GT29 CAZy family known to catalyze the transfer of sialic acid molecules onto vertebrate glycoproteins and glycolipids. It has long been known that, the resulting sialylated macromolecules are trafficked to the cell surface where they serve as the preferred interface between cells and their environment. We used sequence homology and HMM-based approaches, and search of specific sequences features known as sialylmotifs to identify ST-related proteins in Bacteria, Archaea and eukaryotes genomes. Interestingly, GT29 ST-related proteins could be identified in protist organisms and quite unexpectedly, in several proteobacteria, most of which are α-proteobacteria. This major discovery also unveiled the evolutionary origin of the GT29 ST-related proteins likely linked to endosymbiotic event that resulted in mitochondria acquisition. We deciphered their evolutionary relationships using new strategies of alignments that were not limited to the basic primary structure and conducting careful phylogenetic analysis. Our data revealed that the LECA, a highly complex organism with most eukaryotic hallmarks^85^ already possessed two types of Golgi GT29 ST sequences likely inherited from a single ST of proteobacteria. Furthermore, we suggest that protists ST have conserved similar functions to those of the Bacterial ST GT29 and that the well-known role of Metazoan ST in cell interactions is linked to their functional divergence in 4 distinct families in multicellular organisms. To infer potential ST functions in protists, phylogenetic studies of key actors of the sialylation pathway were carried out (Supplemental data 3, Supplemental data 4) that lead us to the conclusion that LECA possessed the ability to use either exogenous sialic acid molecules or an endogenous sialic acid biosynthetic pathway to produce CMP-sialic acid and supply Golgi GT29 ST with their activated sugar-donor (Fig. 6). During eukaryotes evolution, this sialylation pathway was partially maintained or totally lost like in the Streptophyta.

## Methods

### Sequence identification

Metazoan ST sequences were extracted from our previous papers^21–23^. We used the motif L as seed to BLAST (tBLASTn) against 68 genomes of Ensembl and GenBank databases including opisthokonts, Amoebozoa, Excavata, Archaeplastida, Haptophyta and allied, Alveolata, Stramenopiles and Rhizaria^47,48^. There were hits from Viridiplantae, including Embryophytes (*Sellaginella moellendorffii*, *Physcomitrella patens*, *Oryza sativa*, and *A*. *thaliana*), Charophytes (*C*. *atmophyticus*), Prasinophyta (*B*. *prasinos*), Cryptophyta (*G*. *theta*), Haptophyta (*E*. *huxleyi*, *Chrysochromulina sp*.), Alveolata (*C*. *velia*), Stramenopiles Pelagophytes (*A*. *anophagefferens*). The genomic and transcriptomic divisions at the comparative genomics platform for early branching Metazoa (Compagen) were also screened using BLAST^86^ leading to the identification of Demosponge (*A*. *quennslandica*), Choanoflagellate (*M*. *brevicollis*) and Sponge (*O*. *carmela*) sequences. We also found sequences belonging to CAZy GT29 in α-, γ- and ε-Proteobacteria and in Archaea. The contigs were then submitted to gene prediction using GENSCAN^87^. We verified that the GT29 module was recognized par HMMER3 program implemented in Smart^88^. Incomplete sequences in their presumed catalytic domain were removed. The alignments were performed using MUSCLE method^89^ included in MEGA7.0^64^ and then refined by hand. From the MSA generated, we only retained the sialylmotifs characterizing the GT29 ST (*i*.*e*. sialylmotifs L, S, III, VS) and the one characterizing each family, named motif a, in C-terminal position of sialylmotif L^40^. The resulting MSA had ~92 positions and contained 180 amino-acid sequences (supplementary data 1).

### Phylogenetic analyses

To construct a phylogeny between these sequences, we chose the best protein model to conduct a maximum likelihood method using MEGA7.0 and Minimum Evolution method with JTT model^64^. Bootstrap procedure considered 350 replicates and the divergence time was deduced from Time Tree site^90,91^. In addition, phylogenetic trees of the GT29 ST with or without those of the GT42 CAZy family, were generated using Phylobayes-4.1^74^ under the CAT-GTR model^70,92^ with the two chains stopped when convergence was reached (maxdiff < 0.1) after at least 300 cycles, discarding 100 burn-in trees. Bootstrap support values were estimated from 100 replicates using IQ-TREE^75^ under the LG4X model^71^ and mapped onto the Bayesian tree.

### Similarity Network Analysis

The same data set used for phylogenetic analysis was used to create a custom BLAST database. The data set comprise 185 full length ST-related sequences including the bacterial ST GT42 (supplemental data 1). The pairwise relationships between sequences were calculated by a BLAST all against all in the custom database and the resulting E-value was taken as a measure of similarity between sequences^93^. The network was visualized using Cytoscape^94^, where each sequence was represented as a node and edges were defined between any pair of nodes with an E-value less than a threshold using the Cytoscape force-directed layout.

## Electronic supplementary material

Supplemental Dataset 1, Dataset 3, Dataset 4 and Supplemental Figures

Supplemental dataset 2
