# Supplementary material for: Reconstruction of the sialylation pathway in the ancestor of eukaryotes
Source: Sci Rep. 2018 Feb 13;8:2946. doi: 10.1038/s41598-018-20920-1 (PMC5811610; doi:10.1038/s41598-018-20920-1)
Supplement: Supplementary file 1 — Supplemental Dataset 1, Dataset 3, Dataset 4 and Supplemental Figures [file 41598_2018_20920_MOESM1_ESM.zip › Supplemental data 1.pdf]

Reconstruction of the sialylation pathway in the ancestor of eukaryotes

Daniel Petit[1], Elin Teppa[2], Ugo Cenci[3,4], Steven Ball[3,4] and Anne Harduin-Lepers[3,4,*]

[1]Laboratoire de Génétique Moléculaire Animale, UMR 1061 INRA, Université de Limoges Faculté des Sciences et Techniques, 123 avenue Albert Thomas, 87060 Limoges, France

[2]Bioinformatics Unit, Fundación Instituto Leloir -IIBBA CONICET, Av. Patricias Argentinas 435, C1405BWE, Buenos Aires, Argentina

[3]Univ. Lille, CNRS, UMR 8576 - UGSF - Unité de Glycobiologie Structurale et Fonctionnelle, F 59000 Lille, France

[4]UGSF, Bât. C9, Université de Lille - Sciences et Technologies, 59655, Villeneuve d'Ascq, France

[*]Corresponding author: anne.harduin@univ-lille1.fr

Correspondence: Anne Harduin-Lepers, Laboratoire de Glycobiologie Structurale et Fonctionnelle, CNRS UMR 8576, Université Lille Nord de France, Lille 1, 59655 Villeneuve d’Ascq, France. Phone: +33 320 3362 46 ; FAX : +33 320 43 65 55 ; E-mail : anne.harduin@univ-lille1.fr

Short title: Origin and transfer of sialic acid in LECA

Supplementary data 1:

1- **Full length eukaryotic ST sequences**
2- **ST motifs sequences**
3- **MSA of all the ST motifs sequences**

1 – Full length eukaryotic ST sequences. 180 ST sequence of the GT29 CAZy family identified in public databases and 5 ST sequences of the GT42 CAZy familly were used in this study. See supplemental data 2 for corresponding abbreviated names accession number and taxonmy.

>Spur_ST3Gal_00992

MISDKVVKQETQTNESSKMGRTTVLRLVDRQAPSILRSLFYLILVCILVLAVDVMLLQYSKNSRSNQLFGYDNMGMPQRRSRPSLDSEEDSRSMRR
PMRHKVKPDPRGPLPSLAVPINRAYFESSNVTCRKGYARTRLQNLYGDRFDPDIPLFVDSSFLDRPNAVNLPMPFGFRDAERTVLNILKHMPETEI
PEDIPQDSCLRCVVQGNGGIASRTRLGEVIDNFDVVFRLNSAPTINHENDVGRKTTFRMAYPESAFRRPEQYDPGWTFVLMMFKPLDLVWLETIIR
GRKMNSRDGFWKKIPLSVPKPTKDFRIFNPEILQDTASMMGMMGVRLCDEVAVAGFGYDPNKPGAALHYYDSLPMREIWKSWTHDIHHERGILRLL
KQQGIITDLSGGIR

>Spur_ST3Gal_00993

LQVTPASGQEYIFSNKSIIDRKVSPKDRKVTPKSIIREDEVKTCQPKRAADRMENLFGGQFNRSLSPFASLSSLRSANSTKYPLPFGFHGTERTAS
DVVGKLPDTDIPADIKKLPCRRCIVIGNGGILKGSKLGSHIDQYDAVFRLNVAPIKGYESDVGTKTTVRAVYPESSNIGSKAYTQNWTLLVVPFKT
NDLKWFDTVIEGRDLKKLKGFWSTTVDFVPKPRKDLRLYNPEITSELSFNLIGMKSGKGSKNVPTTGAMVIMVAIRVCDEVSVAGFGYDMKRLDTP
IHYYSSQKTKAILSSFTHDIDHETKFLHKLVEQGVINDITGGLRTKL

>Hpul_ST3Gal_00793

MGRATVLRLVDRQAPSILRSLFYIILVCIVVLAVDVVLLQYSKNSRSNQLFGYDNMGMPQRRSRPNLDSEEDSRSMKQPMRHKVKSDQRGPLPSLA
VPINRAYFESSNVTCRKGYARTRLQNLYGDRFDPDIPLFVDSSFLDRPNAVNLPMPFGFREAERTVLNILKHMPETEIPEDIPQDSCLRCVVQGNG
GIASRTRLGEVIDNFDVVFRLNSAPTINHENDVGRKTTFRMAYPESAFRRPEQYDPGWTFVLMMFKPLDLVWLETIIRGRKMNSRDGFWKKIPLSV
PKPTKDFRIFNPEILQDTANMMGMMGVRLCDEVAVAGFGYDPNKPGAALHYYDSLPMREIWKSWTHDIHHERGILRLLKQQGIITDLSGGIR

>Skow_ST3Gal_00797

DGCRRCIIVGSGGILKNKGHGRVIDEYDVVIRMNVSPVKGYEKDVGTKTTIRITYPEGAPRKNNLYDVDALLGIVVFKLADLNWLDSVLHRKTPST
KGFWSKIAMSVPKNPRQTRIINPLIIREASFDLLGYPTNLGKMNKNVPTSGTISIMLALRLCDEVDVVGFGYNTKEPEALIHYYEKVKMKIITSLF
THDINHETAFLKKLVEF

>Bflo_ST3Gal_00792

MKRRVTMRTMELAMRLFVFLLGAGVIMINVFYSYLSHKIQQREGPSLQEYYRSLNYTDEVFLKRIGIRPPGPKFSHPIKYPEGDTCVPGYARSKVT
EMNPKFNTNLPLFVKKGFREWKGMMDDGHDKLPFYLSDQDCKKIDQLLDLLPNSEPALLTGGGKCRRCIVVGSSGLVSGENLGSTIDKYDIVMRMN
EAPVHGYEKDIGSKTTFRFLYPESATSKEVDPDADYIIVPYKTDDLAWLLAVVQKEKPEGSFWKEIGETLNVDSSHIHILNPQIARSTSYEHVGMI
NIPTIGTISIITAFHFCDMVDIVGFGFRHDKLYHYYENKTIEGEFEGLFSYFTLIHDFAKEKEYIKDLVRNAVVNDLTHGYED

>Skow_ST3Gal_00796

FVSRFDPNIPLFLTSNFKEWTDYARMKTSSEPFGFKNHCKRCILVASSGVSTGKGLGKLIDKYDVVIRMNNAPVKKYEKDVGSKTTFRLVYPESAF
RTSSSYDKNSVLVFVPYKLDDYLWLEAVIKgDPIKKGLKFWKKSATSLNKPADKIQILNPYLGHEVANYHMKTKVKRPSTGAMAVVMALHYCDHLH
ITGYGYHPNVTIHYYEKSKGIPRDGAHSWFNENRYIMKLLYC

>Skow_ST3Gal_00795

CTRCVLVANSGILIGSKLGDVIDTYDIVIRMNNAKTVGYADDVGRKTTFRFIYPESALMDTSQYNRDSDIVFMSYKEDDYWWLASVLKKKISGYFW
KEIPNSLDKPEYQIRLLNPAIHQEARDIHLCSKVSQKRPSTGIVAMVFAFHYCDIVDIAGYGDDPRMPNHYFSDPVLDNIRVTSTTHCWDNEAKYI
ERMLYCGAIRK

>Hsap_ST3Gal3

MGLLVFVRNLLLALCLFLVLGFLYYSAWKLHLLQWEEDSSKYSHSSSPQEKPVADSVVLSFDSAGQTLGSEYDRLGFLLNLDSKLSPRTLCTVVFG
LDCILESPGEPKKLLMPASHPLEILKSLSEDTAFALGFLKLPRPAELATKYANFSEGACKPGYASALMTAIFPRFSKPAPMFLDDSFRKWARIREF
VPPFGIKGQDNLIKAILSVTKEYRLTPALDSLRCRRCIIVGNGGVLANKSLGSRIDDYDIVVRLNSAPVKGFEKDVGSKTTLRITYPEGAMQRPEQ
YERDSLFVLAGFKWQDFKWLKYIVYKERVSASDGFWKSVATRVPKEPPEIRILNPYFIQEAAFTLIGLPFNNGLMGRGNIPTLGSVAVTMALHGCD
EVAVAGFGYDMSTPNAPLHYYETVRMAAIKESWTHNIQREKEFLRKLVKARVITDLSSGI

>Lcha_ST3Gal3

MGVLVHVRNLLLALCLVLVLGFLYYSAMKLHWNSWGQDSHFVTNPFDAGGQSLGLEYDRLGFLLRLDSRLTLELNSKYTNFTEGACKPHYAATQMT
AIFPRFMKPAPMFLDISFKRWARIKDFPPPFGIKGQDNIIQRILETTKEYNLTPELNSRSCKRCIVVGNGGVLANKSLGSKIDEYDVIIRLNGAPV
KGYEKDVGAKTTIRITYPEGAIQKAEGYEKDSLFVFAGFKPQDFKWLKCIVYKEKVNLADGFWKSVATIIPKEPHEIRILNPYFIREAAFTFIGLP
TNNGMMGKGNIPTLGTVAITMALHNCDEVAVAGFGYDLNNPNAPLHYYESIKMLAIKESWTHNIGREKEFLRKLVKGQVISDLTNGI

>Drer_ST3Gal4

MTLKATKTWFLRLLPVVLFFICYYCSQFYFESSGGKSPHPTLALCPNYRLQKKWENLNLKSMSRKPELFLKLEDFFWKDHLSAEALPYGIKGSELL
LLKVLAAISSFTMPANIESLDCRTCAVIGNGFALKNSSLGEIINKYDVVIRLNDAPVRGFEEDVGNKTTLRLFYPESASYNPGIHNDPDTLLVLVP
FKQQDLRWLKEILYDEKRVSKGFWKPPPQIWLGRASQIRVLDPYFLRITARKFLQIPVQVHPTTGLLAVFVALNYCDVVHVAGFGYPASRNQNQPI
HYYGQQTMKSMFQNSYHDLNQEAQILHRLEEQGVILYLHPHS

>Hsap_ST3Gal4

MVSKSRWKLLAMLALVLVVMVWYSISREDRYIELFYFPIPEKKEPCLQGEAESKASKLFGNYSRDQPIFLRLEDYFWVKTPSAYELPYGTKGSEDL
LLRVLAITSSSIPKNIQSLRCRRCVVVGNGHRLRNSSLGDAINKYDVVIRLNNAPVAGYEGDVGSKTTMRLFYPESAHFDPKVENNPDTLLVLVAF
KAMDFHWIETILSDKKRVRKGFWKQPPLIWDVNPKQIRILNPFFMEIAADKLLSLPMQQPRKIKQKPTTGLLAITLALHLCDLVHIAGFGYPDAYN
KKQTIHYYEQITLKSMAGSGHNVSQEALAIKRMLEMGAIKNLTSF

>Mdom_ST3Gal4

MIGKSRWKILAVVALFLVMAWYSISREDSFYFPVQMEGLKCPQGEVEKKAAQLIGNYSRDQPTFLQLKDFFWIQKTSTYQLPYGVRGSEDVLLRVL
AITNYAMPESIQKLKCLRCVVVGNGHRLKNSSLGNTIDKYDVVIRLNNAPVVGYEGDVGSKTTMRLFYPESAHFSPKVQNNPDTLLVLVPFKAIDF
HWIETILRDKNRVLKGFWKQPPLIWDVNPKQVRILNPFFMEVTAAKLLSLPIRPSRKIKQKPTTGLLAITLALHLCDLVHIAGFGYPDSSNKKQTI
HYYEQITLKSMSASEHNVSHEALAIKRMLEIGAIKNLTYF

>Acar_ST3Gal4

MIHKSRWKILGVLTVFGVMVWYTISREESFDFHIMQDERMTCPLGEVEKKAAQLIANYTRDHPLFLQLKDYFWVRTPSPYELPYGTKGAEEILLRL
LAVTSYSLPESVQSLKCRKCAVVGNGHRLRNSSMGDVINKYDVVIRLNNAPVHGYEHDVGSKTTMRLFYPESAHFNPKTENNPNTLLVLVAFKPMD
FLWMETILHDKKRIRKGFWKQPPLIWDANPEQVRILNPYFMEVTAAKLLNIPMKQLRKFKQKPTTGLVAITLALHFCDMVDIAGFGYPDSANKKQS
IHYYEQITVKSMATSGHNVSHEASAIKKMLVMGLIKNLTYF

>Lcha_ST3Gal6

MRKKYLYFLLIIILFASFMKLFYMWWGPWSSIMKVAGFRPNKAELESNYNQEYKHTDLRTIQDPFLCMDSVVKPNSEDKQQLPYGMRKAEYLLIRVL
KQLEKCDVPMTVKKKFSKKCIVVGNGGVLRNKTLGEKIDSYDIIIRLNNGPVIGHEKDVGKRTTFRLCYPESIFSNSSHYDPDTIVVLLIFKQEDL
QWLLDLINGKKVNAKGHWKAPADTLIYKLSQIRILNPIFLKIAAFEFLHLPLVHDKKKMPVHPTTGIIATALALHLCDETHIVGFKYNFNEPNSSL
HYYEDVSMSAMTKIPYHNITAEQAFLKELRSLKIITDLTEN

>Hsap_ST3Gal6

MRGYLVAIFLSAVFLYYVLHCILWGTNVYWVAPVEMKRRNKIQPCLSKPAFASLLRFHQFHPFLCAADFRKIASLYGSDKFDLPYGMRTSAEYFRL
ALSKLQSCDLFDEFDNIPCKKCVVVGNGGVLKNKTLGEKIDSYDVIIRMNNGPVLGHEEEVGRRTTFRLFYPESVFSDPIHNDPNTTVILTAFKPH
DLRWLLELLMGDKINTNGFWKKPALNLIYKPYQIRILDPFIIRTAAYELLHFPKVFPKNQKPKHPTTGIIAITLAFYICHEVHLAGFKYNFSDLKS
PLHYYGNATMSLMNKNAYHNVTAEQLFLKDIIEKNLVINLTQD

>Hsap_ST3Gal5

MRRPSLLLKDILKCTLLVFGVWILYILKLNYTTEECDMKKMHYVDPDRVKRAQKYAQQVLQKECRPKFAKTSMALLFEHRYSVDLLPFVQKAPKDS
EAESKYDPPFGFRKFSSKVQTLLELLPEHDLPEHLKAKTCRRCVVIGSGGILHGLELGHTLNQFDVVIRLNSAPVEGYSEHVGNKTTIRMTYPEGA
PLSDLEYYSNDLFVAVLFKSVDFNWLQAMVKKETLPFWVRLFFWKQVAEKIPLQPKHFRILNPVIIKETAFDILQYSEPQSRFWGRDKNVPTIGVI
AVVLATHLCDEVSLAGFGYDLNQPRTPLHYFDSQCMAAMNFQTMHNVTTETKFLLKLVKEGVVKDLSGGIDREF

>Drer_ST3Gal5

MRRVMKQSSCYFSKRTMILLLSLALMSLAFLKLPSFHTELKPVEVPVDNKFRKRVHSHVREILDKECRPSFARQRMVTEHHGSTPTIDPFLNKNMK
LDEQIFQYPPPFGFLDMKKKLEEILNLLPVSSEQRLGERDCRRCVVVGNGGILKGLGLGHLLNRFDIIIRLNSGPLQDFSADVGNRTTIRMSYPES
CPKVWEDTDPDLKYVAVIFKSVDFHWLRAMISRTPVSLWDRLFFWQNVPMSVPVKTSQFHLLNPQIIREMALDLLNYPE PKKRLWSWDQNIPTLGL
TALNLATYICDEVSLAGFGYNLSQKEAPLHYYDSVPMTTILKEAMHNVQKETVFLKRLVASGSITDLTGGIHCSFC

>Drer_ST3Gal7

MVTLKHLSVNDNEEDGVPLLPEASSLESPTAQHSRAESRDFFLSRRNNFIVSIVLLLSLYSALLVPAYLPTAEPIWTNESSAENLALLNQSASLLS
QSCHSGWSVERMRTMLSTPAGLLKIPVFLENGTGDWALPPPLGLQGSEEMAEQALKALPFTAVPTGPETCRRCVVVGSRGILHSKNLGAHIDHANI
IIRVNNAPVFGFESDAGSRTTIRLIYPEGAPSHIQEYERTEVVALVVFKSLDLAWMTSVVTKEPLVWWSKLWFWKDVIDSIPLQPENVRILNPEIM
YRTGQVLQTYAEQQRKVMVPTLGITAVVVALQVCDEVSIAGFGYDLQHPGAPLHYYGSLRMDAMKTQVVHDVSAETVFLKELVKAGAVRDLTGAL

>Lcha_ST3Gal7

MVTSFHPSGLYNSSVNRFEILWDERRWNLLTSILLLFCGYAALLYPPYCLDPQDIWKRYTLEPRRIQALHNQTAVLLSRPCQAQFSQRKLSELYPG
KVPRNLSVFVEAWEPEGSRYRPPFGFRGCEEELRQALAVLLPVTRLPSSQLPAPACRRCVVVGSAGILHGSRLGAYIDQHDIIIRKNGICLKGYTI
VNIQIKTVVGISEYERMNSVQNYQSVYVFSLFLYMSIYLYTHTSVTTSIFVPPQGLWTKLWFWQSVVEFIPLCPKNFRILNLEIIRETALEFLGYP
EPQDSPFRINQMVPTLGVSAVVMATHLCDEVSLAGFGYDLSRPQAPLHYFETVHMDAINAQAMHNVDTEKLFLAALVKAGAVNDLTGGI

>Ggal_ST3Gal9

MQQLLPRSRRMCMKTSMNAIAVVRRLFLVLFQCKKLLFLLCVGLCVKSLYHVTNTLGRHIENGALYRVFGGDPFMDTGGQELESRTTWRHVGACRR
RFMWRKPFLSRFESHWEPFLTSKSIYSLLDRPFKQELEEVLWLHKLPFGLHSSVLSAFSTLQLLPEQDLPGSFGRLWCQRCIVVGNGYSVHGQHFG
KMIDSHHVIIRLNDAPVKEYKKDVGERTSIRLFFPESALPNPLENSDNNTLMVFVPFKPLDFLWLREVLLKTKNKTTEGFWRQPPQEWNWNISQLR
ILNPYVTYEATYKLLQLNASSKRYATTGIIALNLALHLCQEVNIAGFGYPGNHDTTTPIHYYNTGLSRKNELFQHNLTAERNWLLKMIEWGVIADL
TSHAFQAQNH

>Spur_ST3Gal_00042

MSSEIDCECCQLCTGCTRPKHPCMISAMNILKIRIGILRKVLILGIVLVTISLLYFVRPLQETHVSRSYISSENDGEVGGGKQDGVDYDVQSEFMG
VLPRVLDSAKRSGVVSHLRSLIGLGWKDNSGTSSKTEIKFDSHAERLLDILEVNLFQNKTNAIDEVTVIKPSRKEGIHRSTSDEGEIPVGDGLQCI
PGKSRDRMLNLFGNGYDANLHPFIGSKTGVSGVMSLPPPFGFRKSESALIKAVTVVGQSDFPTSNSCSRCVVVGNGGVMKQSAMGPIIDDFDVVIR
LNDAPTVGYEKDVGSKTTIRMAYPESSFQSSLLYKGNWLYVIVIFKQADLLWVADVAAGKKAPSLKFWKSVARAVPKPSDEFRIFNPLIIRETAEL
VGMKVGNGKVGKNVPTTGSFAISMATRLCDEVSVAGFGYDVTKPLHYYDKLQMKIVKESWTHNIDIEKKWLLKMVQQGVIHDLTRGIK

>Bflo_ST3Gal_00823

MSTLTRTYLVIIIIGFSVVLSLLYYVSSHMMTPQHRREHLHDSPGLLRKHTGSQPSLYEEPGLSNATENPSTSKDDNQSHQIRVLEETTDNKTYSR
TTLPLKQTTKTVKQTAKTVKQTAKTVSNGGAPKPETRPIFVQKVEEKETVKDAVAMEMGDENGVADEEEVCRPHHASSTIVNTHPKFRKDIPLFVT
NDYKSYSSVMNYSPPFGLRGTDKDLMNVLKILPQASTMPENIERIPCKRCIVVGSGGILLGKKLGPQIDDFDIVIRMNNGPVKGYEEDVGHKTTIR
MSYPEGALQDPGGYHQDSLFLMVPFKSLDFLWLQRIIGKQSVGDIASRFWKSIAHAIPKNADKFRIVNPALLQETSFDLIGFPTMGGKMGKNVPTI
GSLAIIWAINYCDEVTVAGFGYDLSQPTVWLHYYKDAKMSSIAKSWTHDINKEKEFLKTLVRNGVITDLTGGIIGHV

>Bflo_ST3Gal_00041

MRRFPHYLPLNQNSEDTPTMSTLTRTYLVIIIIGFSVVLSLLYYVSSHMMTPQHRREHLHDSPGLLRKHTGSQPSLYEEPGLSNATENPSTSKDDN
QSHQIRVLEETTDNKTYSRTTLPLKQTTKTVKQTAKTVKQTAKTVSNGGAPKPETRPIFVQKVEEKETVKDAVAMEMGDENGVADEEEVCRPHHAS
STIVNTHPKFRKDIPLFVTNDYKSYSSVMNYSPPFGLRGTDKDLMNVLKILPQASTMPENIERIPCKRCIVVGSGGILLGKKLGPQIDDFDIVIRM
NNGPVKGYEEDVGHKTTIRMSYPEGALQDPGGYHQDSLFLMVPFKSLDFLWLQRIIGKQSVGDIASRFWKSIAHAIPKNADKFRIVNPALLQETSF
DLIGFPTMGGKMGKNVPTIGSLAIIWAINYCDEVTVAGFGYDLSQPTVWLHYYKDAKMSSIAKSWTHDINKEKEFLKTLVRNGVITDLTGGIIGHV

>Hsap_ST3gal1

MVTLRKRTLKVVTFLVLFIFLTSFFLNYSHTMVATTWFPKQMVLELSENLKRLIKHRPCTCTHCIGQRKLSAWFDERFNQTMQPLLTAQNALLEDD
TYRWWLRLQREKKPNNLNDTIKELFRVVPGNVDPMLEKRSVGCRRCAVVGNSGNLRESSYGPEIDSHDFVLRMNKAPTAGFEADVGTKTTHHLVYP
ESFRELGDNVSMILVPFKTIDLEWVVSAITTGTISHTYIPVPAKIRVKQDKILIYHPAFIKYVFDNWLQGHGRYPSTGILSVIFSMHVCDEVDLYG
FGADSKGNWHHYWENNPSAGAFRKTGVHDADFESNVTATLASINKIRIFKGR

>Olat_ST3Gal1

MSPLPQNKFWTFFLLCCILTFTTLLFSYTFTDPPFHFLKYASRISGSFFSKDICACDSCMADLGEDAWLADHFNLSILPLMTRDSSALSEDTYRWW
QWLQREENPANYTQVVEELFRIIPEEGVFMDAGPQRCRTCSVVGNSGNLKGSNYGALIDTSDLVIRMNKAPTKGFEKDVGAKTTHHVMYPESAVDL
SNTTSLVLVPFKTLDLQWIISALTTGTISRTYMRVKERIQADKDKVVVINPRFIKYVYESWLEGRGHYPSTGFLTLMFALHICDEVSVFGFGADQH
GNWHHYWEHNQLAGAFRHTGVHDGDYEYNLTLTLADKHKIQMFRGR

>Lcha_ST3Gal2

MRCSFRLWLLSVALLLVFVTSLLYTYSLSSVSGLAYIDMLGVEDTPRVKLVPSYPHLQKQKNAPLLKMCTCARCLGDLGVSDWFDENYNMDVLPVW
TKENINLPPDVYYWWLMLQPQFKPYNLKNVLGKLFEIIPGMNPYNSWDPQSCRRCAVVGNSGNLHGSGYGKAIDMHDFIMRINQAPTVGFEADVGS
RTTHHFMYPESAKNLAPNVSFVLVPFKTLDLLWITSALSTGEIRFTYAPVKQFLRVDKDKVQIYNPAFFKYIHDKWTEHHGRYPSTGMLVLFFALH
VCDEVDVYGFGADSRGNWHHYWENNRYAGEFRKTGVHDADFEAHIIDSLVQIGKIRVYRGK

>Hsap_ST3gal2

MKCSLRVWFLSVAFLLVFIMSLLFTYSHHSMATLPYLDSGALDGTHRVKLVPGYAGLQRLSKERLSGKSCACRRCMGDAGASDWFDSHFDGNISPV
WTRENMDLPPDVQRWWMMLQPQFKSHNTNEVLEKLFQIVPGENPYRFRDPHQCRRCAVVGNSGNLRGSGYGQDVDGHNFIMRMNQAPTVGFEQDVG
SRTTHHFMYPESAKNLPANVSFVLVPFKVLDLLWIASALSTGQIRFTYAPVKSFLRVDKEKVQIYNPAFFKYIHDRWTEHHGRYPSTGMLVLFFAL
HVCDEVNVYGFGADSRGNWHHYWENNRYAGEFRKTGVHDADFEAHIIDMLAKASKIEVYRGN

>Ggal_ST3Gal8

MRCRKRALLVLALCLPLGLWLLFRASVSGLGLPPSLPTVRCTASANASAWFSARYDAAAGPLLTGPAHELSPDVVHWWLTLQGTPSIVPLRAILQK
LFAVVPAPNGSVWDPSHCRTCAVVGNSGRLKGSRHGLQIDAHHWVLRMNRAKTAGFEMDVGARTTHHFMYPESAMNLWPGVHLVLVPFKPLDLKWV
TSAFSTGELTHTYTRVKQFIKADRNKVLILSPAFLKYIHENWTERHGRYPSTGFTALLFALHACQQVSVFGFGADSKGNWHHYWEENRWSGAFRRT
RVHDADVEFSLIQRLAAEGRILFYQ

>Lcha_ST3Gal8

MARHNHRIMWLLTIILLLCVYMVIYDMGEDKQKLIKIPSIRRLSGRTIVLDKKLCSCEKCVSEKEESAWFDERFDPNFQPILMTEVQDIPSHALQW
WLVQAGNKNYNLSESIAKLFTVVPRTNHSGIRDPAHCRKCAVVGNSGNLKGSNHGKEIDAHHFVIRMNRARTAGFEPDVGIKTTHHLMYPESSQDL
QPGVHLVLLPFKIMDFEWIRSALTTGEITRTYFRVQQFIKADKDKVLIINPTFFKYVCDHWTEHHGRYPSTGMTALVFALHICDEVSVFGYGADSN
GNWHHYWENNRNGGAFRRTGVHSGDFESQIIKKLADEGKIIFYK

>Drer_ST3gal8

MMSRRKLCLGALLGAILFLMIAAHTIQKAGVVPITDLPTHHDVRIPVKNHTLLHPVTGPRRGHSCSCTSCIAYIGVSEWLDQRYDQKQKPYLTGRD
DDVDPLSLKWWLSLQASDGTIKDVTQKMFKIISPPHEDETPRQNQCRKCAVVGNSGNLLKSKYGALIDSHSTVIRMNKAVTVGYDEDVGYRTTHHF
LYPESAIHLRPGVHLVLLPFKLKDMQWLSSALSTGEIKMTYMRVKNRIDADKDKVMVVNPAFFKYTHDRWTERHGRYPSTGIVAIIFALHLCDEVS
VFGYGADAQGNWHHYWEYNRYAGAFRKTGVHNADFETEIIQRLSAEGKIKLYR

>Bflo_ST3Gal_00835

MIRMTVKLFITTIVFVLIMLGAMYHYVQPGKSPIARKTGGTAPPDEDDKNNSQLSRWWRKFQGDGEPVQLQANKSAAVTTPPVVVPTPPVVVQPTC
KRIWQKGRSSWFDSRFDDNIRPVWSRANIELPADARKWWMSLQSHKDEDPAPLLNALFDMGAPDVDPWASRNLTGCLRCAVVGNSGNLRQSNYGEE
IDGYDLIFRMNDAPTKGWEKDVGHRTTHHFMYPESATDLPDDVSFVLLNFKPLDLKWMKTSLTDGSITRTWTRVKGRIKANKTKILVYNPAFFKYV
NDKWTEHHGRYSSTGSLVILFAVHVCDEVDVYGYGADKLGNWNHYWTTHYSGAAHRSTGVHDSEFERVLLEKLQSEGIIKIHRGNAANK

>Bflo_ST3Gal_00837

MYHYVQPGKSPIARKTGGTAPPDEDDKNNSQLSRWWRKFQGDGEPVQLQANKSAAVTTPPVVVPTPPVVVQPTCKRIWQKGRSSWFDSRFDDNIRP
VWSRANIELPADARKWWMSLQSHKDEDPAPLLNALFDMGAPDVDPWASRNLTGCLRCAVVGNSGNLRQSNYGEEIDGYDLIFRMNDAPTKGWEKDV
GHRTTHHFMYPESATDLPDDVSFVLLNFKPLDLKWMKTSLTDGSITRTWTRVKGRIKANKTKILVYNPAFFKYVNDKWTEHHGRYSSTGSLVILFA
VHVCDEVDVYGYGADKLGNWNHYWTTHYSGAAHRSTGVHDSEFERVLLEKLQSEGIIKIHRGNAANK

>Csav_ST3Gal_00830

MLINFKTRRVVAMLLVVAIIITYSWLLIWSTRNALTQSPQNMSEKKAPVINLVAGERKRPQQARVQGRRIDLGRSNYSHLHNETFPNKKCGRDLDA
SEKRWFKGRFNPEIQPVWTESTLEIDYLVYDWWLSLQSSRGENLDKVFENLYKIGVPRNNPFARPNHDNEAICRRCAVVGNSGNLINSKYGNIDSH
DFVIRLNKGPTEGFENDVGRKTTHRFMYPATASSLAQDVSLVLLPFQPQDVKWLLSALTTGELTNTYQPVISRVTCDKSKIVIISPTFIRYVHDRW
TQHHGRYPSTGLIAIIYALHECDQVDLYGFGADSAGNWHHYWEDLPPHIAGAFRQTGVHDSDKESSMINQLHIHRLLTLHVPGKDL

>Skow_ST3Gal_00834

MSYSRVRRRLPVVTFIALIFLLSLFTLMYKLYSPILVSNANMKSTARLKDSFTAATLLSSNCTRMPVWTKGEYKFFDDRYSNISTIWSSKHKEMTP
EALQWWLRIQQSKDTFSTSDLRNLVGKLLEIIPDVMPYATNVSNRCLQCAVVGNSGNLRDSRYGQLIDSHDYIMRMNTAKTVGFEKDVGSRTTHHF

MYPESFVEVIGETKFVLIPFKPLDVSWLISALTTGTIVRTYMPVRKQINVSKKMIEVYNPAFMYYVHKTWNELHGRYPSTGMLVLLFAMHICDQVN
VFGFGATSKGNWDHYYQPPVEPSLEKDSAFKLTGVHNAEFETDIVKQLEEIGKITVYRGSRV

>Spur_ST3Gal_00990

MARATLKAVTVVSSVLVFGLLVGYRLADPSVYESGGPSKRDSSHSTLESNDHRTSYTTRARIGIELNSTTTAAGGQRCRRLWKKGTSEWFDEKYND
SLLPVWMKENKKMSEEIGKWWLSLQRRENSDYIGALDKAFEVIPNPQRFLTRNVSRCLRCAVVGNSGNLRNSGYGTAIDKHDVVVRINQAKVKGFE
KDVGQKETHRLMYPESFMDIAPETNFVLLSFKVIDLQWARSAITTGEITKTYTNVRRKIRVTPSKILFYNPALMYHIHREWIDRKGRYPSSGTLAV
FFALQFCDEVSVYGMGANSKGFWDHYWEVNDGSRNSAFLKTHVHDSVHEFEVIKKLADEKIITMFQGVR

>Ocar_ST3Gal_00829

MVHKIMPRCRFVSFSIFLFVIMAIFVFIRHQTSWEESVSLEDPMMRSQPAVKPEETKDQPAGKPKETKDQPAGKAEEAEDYPRRDSTICPNIGTLL
ANPNLPRILKSKFHPDVKVMLTQDTKTLDEPINSWWKKLQPFDNKRDFTELAEDLFTVIPGENPFSPGVCRRCAVVGTAGRLKGARQGKLIDSFDI
VIRMNRSPVKGYEVDVGSKTSYHLVYPESAVGYRGAESSGKLVLFPFKVLDIEWLKSIFTTHPISKGWTHLPTNLGLKPTDAMVIHPEFIYYVAKT
WLEGKGRWASAGALSVVWALHICNEVDVFGFGANKYGNWDHYYEKFSSKEKDPFRRTGVHDANIEE

>Ocar_m10828

PINSWWKKLQPFDNKRDFTELAEDLFTVIPGENPFSPGVCRRCAVVGTAGRLKGARQGKLIDSFDIVIRMNRSPVKGYEVDVGSKTSYHLVYPESA
VGYRGAESSGKLVLFPFKVLDIEWLKSIFTTHPISKGWTHLPTNLGLKPTDAMVIHPEFIYYVAKTWLEGKGRWASAGALSVVWALHICNEVDVFG
FGANKYGNWDHYYEKFSSKEKDPFRRTGVHDANIEETVRMELHKEKIIRFHPGN

>Bathy02g00010

MGLTTEARRTTMEFPSSHVSVPFASRSGTTAPEEDNKSEAFCSPSGVLSTPRMNKAASKVFVDGMPERMLPKPSQIFKTVDVVEDGHTSSSSSKGV
PERIARRVKFKRCAVVGNGGILKSAEFGQAIDAHDVVFRQNQAPTATYELFVGEKTTFRVLNKKWTQQYSRGDKEYLPLEKDVYLIASRGIDKIAR
NLVKAYPRRPDVRVVGLESAVRGAVGKLMREFKSKADKCLAKGFKPKGGNTPSSGIISTVLAMSLCDEVNLYGFGVDDRNVGRGWRNQLPSALPPE
AKYQYYVLRGTERSGVDAVHSMELEHAILDGLVGAGYINRCGAGKETCGLNPRKKIGRLGNRKDLLKAESMFNAREIMMRERASKGKEEKEEEDGG
GDDDDEHKDDAVSTTGKAFYGENSNESNSDDKIEEAGELDERLSDDELDEDDALTKMMNFAAKGMKYLFSSSSSSVLDEDDPLAPR

>Hsap_ST6GNc1

MRSCLWRCRHLSQGVQWSLLLAVLVFFLFALPSFIKEPQTKPSRHQRTENIKERSLQSLAKPKSQAPTRARRTTIYAEPVPENNALNTQTQPKAHT
TGDRGKEANQAPPEEQDKVPHTAQRAAWKSPEKEKTMVNTLSPRGQDAGMASGRTEAQSWKSQDTKTTQGNGGQTRKLTASRTVSEKHQGKAATTA
KTLIPKSQHRMLAPTGAVSTRTRQKGVTTAVIPPKEKKPQATPPPAPFQSPTTQRNQRLKAANFKSEPRWDFEEKYSFEIGGLQTTCPDSVKIKAS
KSLWLQKLFLPNLTLFLDSRHFNQSEWDRLEHFAPPFGFMELNYSLVQKVVTRFPPVPQQQLLLASLPAGSLRCITCAVVGNGGILNNSHMGQEID
SHDYVFRLSGALIKGYEQDVGTRTSFYGFTAFSLTQSLLILGNRGFKNVPLGKDVRYLHFLEGTRDYEWLEALLMNQTVMSKNLFWFRHRPQEAFR
EALHMDRYLLLHPDFLRYMKNRFLRSKTLDGAHWRIYRPTTGALLLLTALQLCDQVSAYGFITEGHERFSDHYYDTSWKRLIFYINHDFKLEREVW
KRLHDEGIIRLYQRPGPGTAKAKN

>Drer_ST6GNc1-A

MQKAWGVVSLILTFCVLLYLVLWNNLSENIHSKTIIISSLILSPLGNTNLFKTQKHLNVERKVNITPIPVLYKKNFTKLPVWDFEDVYLRDSNARK
PTCPKSLHNTEDPEFKESVLPDIQLWLYKGQLNMSEWNRLAHFNNPFGFMEYNYNEIKRAVDLIPKPRSSILLPVPKGSKDGCIRCAVVGAGGILN
NSKMGREIDSHDYVFRVNGAVTKGYEEDVGNRTSVYVHTAFSLYATILTLKKYGFHNIPQDEGIKYVMIPEGLRDFEWLQGLLQGKAANGSFKGVR
PLNFFNGHFNESRFNVLHPDFLRYIRNRFMPSKQMQGNYWAMYRPTNGAFALFLAIHTCDMVNAYGFITEDHHKYSNYYYEKFKKTSVIFYINHDY
GLEIKETTRLWNHQTLSKTLT

>Hsap_ST6GNc2

MGLPRGSFFWLLLLLTAACSGLLFALYFSAVQRYPGPAAGARDTTSFEAFFQSKASNSWTGKGQACRHLLHLAIQRHPHFRGLFNLSIPVLLWGDL
FTPALWDRLSQHKAPYGWRGLSHQVIASTLSLLNGSESAKLFAPPRDTPPKCIRCAVVGNGGILNGSRQGPNIDAHDYVFRLNGAVIKGFERDVGT
KTSFYGFTVNTMKNSLVSYWNLGFTSVPQGQDLQYIFIPSDIRDYVMLRSAILGVPVPEGLDKGDRPHAYFGPEASASKFKLLHPDFISYLTERFL
KSKLINTHFGDLYMPSTGALMLLTALHTCDQVSAYGFITSNYWKFSDHYFERKMKPLIFYANHDLSLEAALWRDLHKAGILQLYQR

>Lcha_ST6GNc2

MSFTRAKLRNCFGQSQQKACPNNIRKRIVKSSFKDKFLETIPVLQWSNHATEGEYNRLKNYAGAFGWQNVDFDTIKESLNLLNSSANSQMFDDWDQ
RPNKMNSCICCAVVGNGGILNDSQMGREINQHDYVFRANGAVVKGFEKDIGNRTSFYIFSTNTMRNSMASYRGVGFLGPPQSKETRYIFLPDHDRD
YLLAKAAILNIPIDRGRDKSNNPPTFFGERVGREKFKVFHPDFIRYLRNRFLWARILNSKHRDIYRPSTGAVMLLAAIHTCDEVSAYGFMTPDYTK
YSDHYFDKKFHKVVFYSNHDYRLEMQLWHDLHKAGIIRLYQGRGNN

>Drer_ST6GNc2-A

MTGPLPKRLLLWFIGLLGIALLALIVVCVAMQIQVPWQVLTLQSFTINHYRLFPVKNNNSSPGFQLSTANEVETNEVLEQNSVQQSDVQDSSANLI
NNAKSDQLNPNANFIKPVPKTEAKPFIPDFFGDQYATDDVPLQTNCPTSIMKLLPQSPFAEKFLHSIPVLQWAKHFTTEEYQRLSHYAGAHGWGSV
DVEVLKSSLRILNTTANHLMFDDWESRTNKSECIRCAVVGNGGILNGSRKGTEIDAHDYVFRVNGAALNGFEKDVGSRTSFYTFSTNTMRNSMRSY
AGVGYSGPPISKETRYIFLPDHDRDYILMKAASTHTIIEKGPERSNKPPKYFGEDVTVEKFKIYHPDFIRYLRNRFLRSNLLNTGAKKIYRPSTGA
VMLLAAIHTCDQVDAYGFMTPDYNLYSDHYYDKVRHPVHFYANHDMRMEMKLWQKLHEAGLMKLFMRT

>Bflo_GT29_ST6GNc_02259-A

CPSALRKNPEKVPEGLKLIPDVPVLMWNEHINPDEYARLSQFKMNYGWQGIPYADIKNCLRHLNTSDHRYMFPGWTPDHTGCIRCAVVGNGGILRG
SGKGKEIDGHDFVWRVNSAIIEGYEEDVGKRTSFYFHDINTMKNSQAATRKFGYKHPPQDKDTVYTTISSSIRDYVYFDAAISWKPVESGRDKSKA
PPSQYGERPANTKFRMLHPDFMRYLKNFWLDSPRRKGSNIYRPTTGGSMLLTALHTCDVTDVYGFITEDHRQYNDHYYESEWHKVVFYANHDFQME
IEIWDKLDKAGLMKLYRGNRTETARKRRK

>Bflo_ST6GNc_02262-B

MQPPTLRYVIIRDIPVLLTSSHVIFSEYERLRRYLPPYGWANVSFEDVVSAARHLNGTLHRSLFNESQGGAPVRCAVVGNGGILRGSGKGEEIDAH
DFIFRVNAAIVKGFEADVGSRTSFYFHTATTLQNSLRSAKKYGFVHVPNTTETVYVDIPTFERDYLFLDAVLSWKPVQRGKDASKSWLNSTRGYGR
FEAIYRPSTGAVALLTAVHVCDVTDAYGFITKTWRNYTNHYYENTFKKFTFFANHDFNLEIKLWDDLDRAGIINLYRGNITGG

>Skow_ST6GALNAc_02258

MLTSQFKISMFFSSLRSGNRVVKVICTLLVISVFATILYTTHLSTQFWPSTLYESNEHASLSSVIVSAVAENPAENPTHEKGSTSIDHNNVTNSSK
VSKPKATAPKTKGIKDENEKLLPNADVFRHEDSYKKYMQCPTTLRNKLIKSKVFKDRFIADIPVLMWDEHKTLEEYNRLSQYWGCYGWQGYGQDLI
NRTLSYLNSPSHRYMFDNQLNLKGQKSGDNKCIRCAVVGGGGILNGSRKGEEIDSHDYVFRVNIAATKGYEVDVGKKTSFYSYTITTMRNSLLGGR
KRGFTVAPHDKETKYLVFPCEISDYILVENRSSNKTEKDFKDTGPKHWGIPKFGKTVKNTDFKMLHPDFQRYMEWYWIRSSRKYKKVNRPSTGALM
LIAAIHTCDEVSAYGFGVNFRKYTTHYYDKQYTKFVYYANHDFKQELKLWNDLDAEGIIKLYKRADDK

>Skow_ST6GALNAc_02269-B

MAVAKGYTRLDILKFVFVSCLLICIIVFYGYRSLWLGSDKNIRSIKLYRPPDNSSALRLASSNVRYVLEKLKADSRSRNLNRTLLTDDIESKLNHS
IRLINSVQLSEHLQVPDIPNTDNTSPYFDTPTEFISELEKLINLRQNETAGLSLAEERKYNKDDQYKYYAHCPSTLRKKILEIPELRKKFIPDIPI
LLTSDHFNKEEYDRLSHFKTPQGWSNVDYKEVADVASNLNTDGSRYMFDNLVSKWKNTARCVRCAVVGNGGVLKNSKLGEEINSHDYVFRVNVAVT
KGFESDVGDKTSHYVFTMVTLDNSIRGGNKYGYKSAPNGKDIRYVLVPCEQWSYQVIGAALSGKPLPRSQDGYSRSPPTFPVKLNNNNVKVLHPDF
SRYLFWSWVDSPGRKRDIYRPQTGAFMLLVALHTCDEVDVYGVGASYSNYTDHYYDNGRHVEYANHDYTTENKLWARLNELGIINLHT

>Lvarvar_ST6GNc_02261

NDTSINIMQRTVSAHSRNIGRRYSKDKILPILSNMLRGTKTEAELGDMERTSVNGSFFRSNLDDIQDQDYSQAPELPMAKMLIEEKKYRHDDQFEK
YIKCPTSVRKKMLDNPKIRSKFIPDIPILMWDKHFNASEYRRLTQYKGINGWRNINYTDVRSMVGQLNSPNNQYMFDDRLRKVKGQSSDCTTCAII
GNGGILKGSGKGAEIDAHDYVFRVNAAVTKGFENDVGKKTSFYCFTMVTLHNTLALRHRTGFEIPKGDENLRYLFFADSDWTYTFLSAALSGKPLP
KSTGKYHRRGPPAFPKPLTADNIKVLHPDFERYIKLYYFFSWVNSSAQHKDVHRPTTGAIMLLAALHTCDQVSIYGFAGSYSAFSEHYYDKTYSKH
VNYANHDNNAENKLWQFLDEQGIVKLYRRSAEEG

>Spur_ST6GNc_02260

MAGCSKWFLVCLVLCLVGLVIVLVINDASIDVMQRTVTSHRKNFGRYSKDAIAPLLSSMLGGSKTAAELKDGEKTSINGSFFRSTLKGILDNDFSQ
APELSIPQMVIEERKYRHDDQFEKYLKCPTTVRKKMLDNSNIRSKFVSDIPILMWDKHFNASEFRRLTKYKGVNGWRDINYTDIRGMVGQLNSPSN
QYMFDDRLRKGQVPSNDCITCAVIGNGGILNGSGKGAEIDAHDYVFRVNAAITKGFEKDVGKRTSFYCFCMHSLYNTLAKRRKTGFEIPKDDENLR
YLFFAKSEWTYTYLDAALSGKPLPASTGKYHRGPPAFPKQLTADNVKVLHPDFERYLKWSWVNSSAQHKDIHRPTTGAIMLLAALHTCDQVSIYGF
AGSYADYSEYYYDKTFSKHVNYANHDYNSENKLWQFLDEQGIVNLYHRTPKEGK

>Ajap_ST6GNc_02264

MAGSEPSKGLLSRGRCVFFACIILVLIVTYLAMVVSETGSSTIPVQFQSNIRKSFARFANGTINNFNRIVDSTFKYRDPQRKEYTQPKVDSGIRAF
AQNLTVLPTTASSLPTEESIPDFVPKFLVKKEPDENDEYASEKVYYHDNQYLQYLKCPTSVRRKMMTMPEMQSKYIVDIPVLMWSKHFSNSEFQRL
NKFRGINGFDDVDTDDVRGTLDALSSPNNQYMFDKRMTNGKPNGGCITCAVIGNGGILNGSRKGQEIDAHDYVFRVNTALTTGFEEDVGRRTSFYC
FTMITLSNTLRGSRQFGFRSPPYYEDIRYLFFADSGWTYEYLNAVLHNKPAPFSRGKYHRGPPQFPKGINKENIKVIHPDFERYLKWSWVNSKHQH
KNVHRPTTGAIMLLAALHTCDQVDIYGFGGSYDKFSEHYYDKKFQKHIFFANHDNIAENNLWEQLHKLGIVKMYKRD

>Mgla_ST6GNc_02268

MAVSTTRLARSVFALCVVLSIGLFAVKVYKLDTPTLNITSGSVEPSKKVPHHSNLLQRFLHNVLERSFHKATSSPVLEENKTLAIIQSFSNSQPAN
STYGEHFYREIEKRTRLREPSWSLQEKQYARDSQFIHYLSCPTSVRKKILLEDYSNRKFLPEMPILIWNEHFSKDEYQRLSKFQGINGWSEIDAQE
VSDALQLLSSPSNRYMFDDRMVNGAIPKDTGCTRCAIIGNGGVMNGSNKGEEIDAHDYVFRVNVALTKGYEKDVGSKTSFYCFTMITLSNSLRGAR
SYGFKAPPYQKGMKYVFFADSAWTYNYLSAVLNNKPPPKSRDKYKRSPPNFVKQLQAEDVKVVHPDFERYLKWSWVNSTAQHKPVHRPTTGAIMLL
LALHTCDEVNVYGFGGSYAKFSEHYYDKSFHRHIFFANHDNNAENALWKRLHELGIINLYIRN

>dre_ST8s2_AY55462

MSFEFRILMFGIGTALVIFVIIADISEVEEEIANIEDSRKFHLKSVALQSNRSSDLNAAPTSLVTYRKSKVSSLASPSDIKRKTSNSSSSEWTFNR
TLSNLIRKNILKFLDAERDISILKSTFKPGDVIHYIFDRQSTTNISENRYHLLPTVSPMKNQHYRXXKCAIVGNSGILLNSSCGREIDSHDFVIRC
NLAPVEEYAADVGLRTSLVTMNPSVVQRAFQDLNSEEWVQRFVQRLQSLSGSVLWIPAFMAKGGEERVEWAIRLILLHTVNVRTAFPSLRLLHAVR
GYWLTNHVQIKRPTTGLLMYTMATRFCDEIHLYGFWPFAHDPDGKPVKYHYYDTLTYHYTSSASPHTMPLEFRTLSALHRQGALRLHTGPCKPPT

>Drer_ST8S3

MVRVVSVLGLVMFSVALLILSLISYVSIKKDFIFTAPKYANAGGPRMYMFHAGFRSQLAMKFLDPAFTSLNTALNENLQESSNWRFNRSAYAELNK
EIAQHIDVPHNFTLTKNSVRVGQLMHYDYSSHKYVFSIGENLRSLLPDASPVLNKRYNTCAVVGNSGILTGSRCGPEIDKYDFVFRCNFAPTEVFR
RDVGRRTNLTTFNPSILEKYYNNLLTIQDRNNFFLSLKKLDGAILWIPAFFFHTSATVTRTLVDFFVEHKGQLKVQLAWPGNIMQYVNKYWKTKQL
SPKRLSTGILMFTLASSLCEQVHLYGFWPFGWDPNTGKELPYHYYDKKGTKFTTKWQESHQLPTEFKLLFKMHADGVLKLSLSHCA

>Drer_ST8S4

MRLSRKHWTVCTISVLLVLFYKTTDIGRNEVHQKASLTWYLEPSATRLMANGSEKLFGNVLNGLDLGVGWKINATLVSIIRKDILRYLDAERDVSV
IKSNFKPGDTIRYVLDRRRTFSVSQTLHSLLPEVSPLKNKTFKTCAVVGNSGILLKSGCGKEIDNHSFVIRCNLAPLEGFADDVGLRSDFTTMNPS
VIQRVYGGLREETQQENLIQRLRQLNDSVLWIPAFMVKGGMKHVDTVNELILKHKLKVRTAYPSLRLIHAVRGFWLTNKINIKRPTTGLLMYTMAT
RFCDEIYLYGFWPFPKDASGNPVQYHYFDGLKYRYFSNAGPHRMPLEFQTLQRLHSKGALKLTTSKCTST

>Drer_ST8S5

MGYSDPTASRDLLGNRSLCFIFICAFGLVTLLQQILYGKNYIKRYLERIDGSLQFNSSSCKELRQDITDVKVLTMVKTSELFERWRNLQVCKWDQN
KEETDNFKMSLSRCCNAPSFLFTTKRNTPSGTKLRYEVDTSGILHISPEIFKMFPDDMPFSKSQFKKCAVIGNGGIIKNSKCGREIDASDFVFRCN
IPPVSDLYSQDVGSKTDLVTINPSIITERFQKLEKWRKPFYEVLQNYENSSVVLPAFYNTRNTDVSFRVKYMLDDFESSRGVFFFHPQYLLNVQRF
WAVQGVRAKRLSSGLMLVTAAMELCEEVHLYGFWAFPMNPSGIFITHHYYDNVKPRPGFHAMPYEIFNFMHMHARGIVHVHTGPCR

>Hsap_ST8S2

MQLQFRSWMLAALTLLVVFLIFADISEIEEEIGNSGGRGTIRSAVNSLHSKSNRAEVVINGSSSPAVVDRSNESIKHNIQPASSKWRHNQTLSLRI
RKQILKFLDAEKDISVLKGTLKPGDIIHYIFDRDSTMNVSQNLYELLPRTSPLKNKHFGTCAIVGNSGVLLNSGCGQEIDAHSFVIRCNLAPVQEY
ARDVGLKTDLVTMNPSVIQRAFEDLVNATWREKLLQRLHSLNGSILWIPAFMARGGKERVEWVNELILKHHVNVRTAYPSLRLLHAVRGYWLTNKV
HIKRPTTGLLMYTLATRFCKQIYLYGFWPFPLDQNQNPVKYHYYDSLKYGYTSQASPHTMPLEFKALKSLHEQGALKLTVGQCDGAT

>Hsap_ST8S3

MRNCKMARVASVLGLVMLSVALLILSLISYVSLKKENIFTTPKYASPGAPRMYMFHAGFRSQFALKFLDPSFVPITNSLTQELQEKPSKWKFNRTA
FLHQRQEILQHVDVIKNFSLTKNSVRIGQLMHYDYSSHKYVFSISNNFRSLLPDVSPIMNKHYNICAVVGNSGILTFIQCGREIDKSDFVFRCNFA
PSEAFQRDVGRKTNLTTFNPSILEKYYNNLLTIQDRNNFFLSLKKLDGAILWIPAFFFHTSATVTRTLVDFFVEHRGQLKVQLAWPGNIMQHVNRY
WKNKHLSPKRLSTGILMYTLASAICEEIHLYGFWPFGFDPNTREDLPYHYYDKKGTKFTTKWQESHQLPAEFQLLYRMHGEGLTKLTLSHCA

>Hsap_ST8S4

MRSIRKRWTICTISLLLIFYKTKEIARTEEHQETQLIGDGELSLSRSLVNSSDKIIRKAGSSIFQHNVEGWKINSSLVLEIRKNILRFLDAERDVS
VVKSSFKPGDVIHYVLDRRRTLNISHDLHSLLPEVSPMKNRRFKTCAVVGNSGILLDSECGKEIDSHNFVIRCNLAPVVEFAADVGTKSDFITMNP
SVVQRAFGGFRNESDREKFVHRLSMLNDSVLWIPAFMVKGGEKHVEWVNALILKNKLKVRTAYPSLRLIHAVRGYWLTNKVPIKRPSTGLLMYTLA
TRFCDEIHLYGFWPFPKDLNGKAVKYHYYDDLKYRYFSNASPHRMPLEFKTLNVLHNRGALKLTTGKCVKQ

>Hsap_ST8S5

MRYADPSPNRDLLGSRTLLFIFICAFALVTLLQQILYGRNYIKRYFEFYEGPFEYNSTRCLELRHEILEVKVLSMVKQSELFDRWKSLQMCKWAMN
ISEANQFKSTLSRCCNAPAFLFTTQKNTPLGTKLKYEVDTSGIYHINQEIFRMFPKDMPYYRSQFKKCAVVGNGGILKNSRCGREINSADFVFRCN
LPPISEKYTMDVGVKTDVVTVNPSIITERFHKLEKWRRPFYRVLQVYENASVLLPAFYNTRNTDVSIRVKYVLDDFESPQAVYYFHPQYLVNVSRY
WLSLGVRAKRISTGLILVTAALELCEEVHLFGFWAFPMNPSGLYITHHYYDNVKPRPGGHAMPSEIFNFLHLHSRGILRVHTGTCSCC

>Drer_ST8S6_AJ715551

MRVMRTLMRWLLPVILLCSFCSVAFWIFISNNNVIPHPASRIPQKASNTQSCKACKDSVIIGKALGNYSNSWKKHEANYKRFRLLLNEKCHAVSKA
VVTQNNTPLGSNVVYDGERRKPLQVTQALYNILAKEQPFGNATWESCAVVGNGSVLANSSCGEEINSAQFVIKCNLPPLDDRYEKDVGNKTNLVTA
NPSILHEKYSGLMERRRPFVESLHSYGQALLLLPAFSYGHNTPVSLRAFYTLEDFGRDPLPIFLNPEYLRKLTKFWREQGLNSVRPSTGLIMASLA
LEICTNVHLYGFWPFGKHPNDSRPITNHYYDNRESKKNVHSMPSEFEQLLKLHKQGVVHIHLGECQPAHR

>Hsap_ST8S6_AJ621583

MRPGGALLALLASLLLLLLLRLLWCPADAPGRARILVEESREATHGTPAALRTLRSPATAVPRATNSTYLNEKSLQLTEKCKNLQYGIESFSNKTK
RYSENDYLQIITDIQSCPWKRQAEEYANFRAKLASCCDAVQNFVVSQNNTPVGTNMSYEVESKKEIPIKKNIFHMFPVSQPFVDYPYNQCAVVGNG
GILNKSLCGTEIDKSDFVFRCNLPPTTGDVSKDVGSKTNLVTINPSIITLKYGNLKEKKALFLEDIATYGDAFFLLPAFSFRANTGTSFKVYYTLE
ESKARQKVLFFHPKYLKDLALFWRTKGVTAYRLSTGLMITSVAVELCKNVKLYGFWPFSKTVEDIPVSHHYYDNKLPKHGFHQMPKEYSQILQLHM
KGILKLQFSKCEVA

>Drer_ST8S1_AJ715535

MVSLRCHRSKYIWATLGVLALLWLYIFPVYRIPSDKEMVEEVLRQGQTWSRNQTAVELYRKLLTDCCNPKRMFAVTKENSPLGKVLWYDGEFYHYH
TVTNETYPIFVQDTPLQLPLKRCSVVGNGGVLKHSGCGNEIDRADFIMRCNLPPLSKDYTDDVGTKTHLVSANPSIIEKSFQNLLWSRKSFVESMK
AYGSSYIYIPAFSMKPGTDPSLRAYHALADSSSNQTVLFANPDFLKNVGIFWKNHGVHGKRLSTGLFLVSLALGLCEEVTAYGFWPFSVGLDERPV
SHHYYDNILPSSRFHAMPEEFLQLWHLHKSGTLRMRVGSCAKERMELKREK

>Hsap_ST8S1_D2636

MSPCGRARRQTSRGAMAVLAWKFPRTRLPMGASALCVVVLCWLYIFPVYRLPNEKEIVQGVLQQGTAWRRNQTAARAFRKQMEDCCDPAHLFAMTK
MNSPMGKSMWYDGEFLYSFTIDNSTYSLFPQATPFQLPLKKCAVVGNGGILKKSGCGRQIDEANFVMRCNLPPLSSEYTKDVGSKSQLVTANPSII
RQRFQNLLWSRKTFVDNMKIYNHSYIYMPAFSMKTGTEPSLRVYYTLSDVGANQTVLFANPNFLRSIGKFWKSRGIHAKRLSTGLFLVSAALGLCE
EVAIYGFWPFSVNMHEQPISHHYYDNVLPFSGFHAMPEEFLQLWYLHKIGALRMQLDPCEDTSLQPTS

>Bathy02g01080

MMRASVVPSRRLRPLLFLSLLVILLRTPTKTNAYSSSSSSSSSTTTTRTTPSLFLSDIFQKMKSQIFYDQTGQSAPTKSISKIKIHGSRDSNVFET
DDVAPMTEEEVEESLAEHEDAEEEGEEGPYVVEKKRKEDGEDDDGKIEEGEEEEKDGSITDATFEMKVSHNARWCGGQTSGFGWKARSEAEKHRKN
LAKNSERRRSEWGGRRRMLMPSVDGDIGIVEESDGDDENDDDENESILRTMLKLNNNATTWSFVQDEETDVSIENVQRRNTLALRAPRGGRTRGGT
GTTPISGKYSQGYFGCERIWGKIKNENENKATRVDAIPDNELSPGLRAVKMCIKDISKCGADAMRGAAELPSTSLIHALPNSGKKKYNSCAVVGNA
GTLLKSKEYGEAIDKHDVVMRFNVMTLTAQLAANVGTRTTFRMVNHLRSRHACCPKSQGGKGKMPEKGGKAKGMSLILWHPGRQAQLLRACKKNIP
NAKVTSIPESYIKKEVNAMNAMRKDLMRLGFGPFNDWKQGTSGFHGILLLAGMCDHLSLYGITSFSAKKGKSKGPDQYGGRGSKNMASWVWHDWEG
EAYAWRLLHATGKATVCSNS

>Athal_NP172305

MKRSVRPLFSALLFAFFAATLICRVAIRRSSFSFASAIAELGSSGLMTEDIVFNETLLEFAAIDPGEPNFKQEVDLISDYDHTRRSHRRHFSSMSI
RPSEQQRRVSRDIASSSKFPVTLRSSQAYRYWSEFKRNLRLWARRRAYEPNIMLDLIRLVKNPIDVHNGVVSISSERYLSCAVVGNSGTLLNSQYG
DLIDKHEIVIRLNNAKTERFEKKVGSKTNISFINSTLCKPSHRAPLLITDPRFDVMCARIVKYYSVKKFLEEKKAKGFVDWSKDHEGSLFHYSSGM
QAVMLAVGICEKVSVFGFGKLNSTKHHYHTNQKAELKLHDYEAEYRLYRDLENSPRAIPFLPKEFKIPLVQVYH

>Oryza_AM048825

MKRRHLPPVLVLLLLSILSLSFRRRLLVLQGPPSSSSSSRHPVGDPLLRRLAADDGAGSSQILAEAAALFANASISTFPSLGNHHRLLYLRMPYAF
SPRAPPRPKTVARLRVPVDALPPDGKLLASFRASLGSFLAGRRRRGRGGNVAGVMRDLAGVLGRRYRTCAVVGNSGVLLGSGRGPQIDAHDLVIRL
NNARVAGFAADVGVKTSLSFVNSNILHICAARNAITRAACGCHPYGGEVPMAMYVCQPAHLLDALICNATATPSSPFPLLVTDARLDALCARIAKY
YSLRRFVSATGEPAANWTRRHDERYFHYSSGMQAVVMALGVCDEVSLFGFGKSPGAKHHYHTNQKKELDLHDYEAEYDFYGDLQARPAAVPFLDDA
HGFTVPPVRLHW

>Physco_12207

MAFQLREFSSINSFSFGKYPRIQSHMLLLFLFAFITVVALFTSVTHISGRDVGAASVAIAGLGRGRKPHLYRSNITNDSTFLAFMISEPDVEHEKA
AIEVLEGSNDPHHRLRGRAGSWPSRLPTPRYAPFWSQFRALLRTWVAMKWYDPKIMQSLVTMIKGPIDRHHHRSNRKKRLYGTCAVVGNNGILLNS

TFGKAIDAHKVVVRLNNARIKGFEKHLGGKTTIAFMNSNILHKCARRVRCYCHPYGDDISIVMYVSQVPHLMDMAMCDPVHAAPLLTDPRFDALTS
CIAKWYSVHEFVEQTGKVVNEWPWVQGFHYSSGMQAVMLALGICEEVDLYGFEKGIGTNKTMSRHHYHTAQRKELHIHDYEAEYVFYDDLVIASHS
NSGSNHSIPFLCDAGIRIHIVRVFGG

>Physco_4887

MGMDVRPVTECKVKLLFTDPSAAIWVRNSITILTREYIDGLPNGWRDYAWKRINKGYRSMCSNITLCNEKLAPVIPSTPPFIPRQYGRCAVVGNSN
DLLQDLFGAEIDEFDAVIRMNGAPVENYTHYVGEKTTFRILNRGSAKALDKIVALSASAQETIIVKTTIHDFMSRMIREVPILNPVYLMVGAPLGK
SAKGTGVKAIEFALSVCEMVDIYGFTVDPGYLEWTRYFSESRRGHAPLQGRAYYQTMECLGVICNH

>Physco_10699

YRCASCVRSAVDRCHNKSSCEDELQLVLPNKPPFKPRQFATCAVVGNSGDLLLAKFGSEIDAHEVVLRDNEAPVNKTYDKHVGRKRTFRLIGEGVA
RNLREVVKSTMDDLVTQLLNIFPVNLDGNSVEFRRGAKGTGIKSIELAVSMCDVVDIYGFTVDPGYADWTRYFSAPRRGHNPLQGRAYYQLLECLG
VSLLTSSLRLTVTTVRPVPGVFMDQSNNKTVTIRSLQILKIHSPMREKSKQNCSVIPSREALSAARVAAFQLKRIRQSDEPGPFSACVVYKKYQIN
DGRRYQSMLLVNSRRQQKTTFVLAGASDMSTIRRTSNYKKWELLRITDLRNQAQKYQIDVGGVSFYKIDGNKLDSLLTTTTSERQRSVVNDYMPSP
PKAYLQAKPAAKSGTAKCSTRSTIDLGLIGLKEI

>Physco_5121

PRIQSHMLLLFLFALITVVVFFTSVTHISGRDVGAASAAIARLRRGRKLHLYTSNITNDSTFLAFVISEPDVENEKAMIKEVLEGSNDPHRRLRDR
AGSWLSRLPTPRYAPFWSQFRALLRTWVAMKWYDPKIMQSLVTTIKGPIDQHHHHNNRKKRLYGTCAVVGNSGILLNSTFGKMIDAHEEIVRLNNA
RIKGFEKYVGGKTTIVFMNNNILHKCARRVRCYCHPYGDNISIVMYVSQVPHLMDMAMCNPVHAAPLLVTDPCFDALTSCIAKYSVREFMEQIRKV
VNEWPWVQGFHYSSGMQAVMLALGICEEVDLYGFEKGTGTNKTVSRHHYMAQRKELHIHDYEAEYVFYDDLVMASHSNSGSNHPIPFLCDAGIRIP
PVRVFGG

>Marchan_33168

MVVLAVVRGALGLVSCSSICKDGNDGPGPEDDDAEDADDDEAEEIADSILLQDYSSWTVVGGSSNMGFAARGKPVQQQGIVGAGGGGGGAGVAMPP
LRNRHCLSASLLLTIIMSFVVSHRLLTTYLAETVIIRSTVSKEKLLNYSLINVADAAERLKMKGLLERSEGGRYSIKEVLFLKRPPPQQFAIGMDR
NSAVRRRQTENAWMVPLTSRRFDPYVVTFYKMARNWLAEGDFDPRVMQDLVRTVKEPMDRRSRDAYLHTPAPSVHEVEGSLSAPSRYRSCAVVGNS
GILLNSSYGALIDSHEMVIRLNNAKTQGFQKHVGSKTTLAFMNSNILRMCSRRPDCACHPYGEDVPIILYICQAPHLMDVALCGDVHKAPLVVTDG
RLDSLCARIVKWYSVKNFVETSGLPVENWDSAHHSFYFHYSSGFQAVVVALGICDRVSMFGFGKHPLLKHHYHTDQRRELALHDYEAEYIFYDDLV
HNRSIPFLSESGISIPPVTIYY

>Marchan_2053

MVLKQKAMKQKPRGKSSSSCYCMKLIKLSSFLCLLLMLANIAAGLGYGNPKFGRKIQRTWMLLRSWKLYRLFHYNYLHPECRKMSSSFQDAFNQTL
IAYAGYNKQEAKSSDNLKQLLDGKFRVMFDRRLHKPIYFQHRATFRQRLKMLAINFGMSFEPKVLQELLEKVKEPMDNHFQRLNYSGVTPGARYKT
CAVVGNSGEVLNTTRGSFIDSHEMVIRINNAKARVSAPSLADFVGSKTTLMFMNSHILHQCSRNWKCSCHPYGSEVPIMLYLSDMEHVLDVAYCGK
EHIAPLFLTDRRFDMLVEKIGRWYSIKQFIQKGRAQKLAERKLWELFKQWENVHRRDYHYSSGLQAVVLALGLCEQVHLFGFGKNPKFQHHFHTKQ
RAEHYSHDYEAEYLFYHDLEKKSIDANPFLCETATQIPPVHVFR

>Marchan_1521

MAVPKVKEAIVSSSTRGNRLIFPPGSVRSVVKVLLVLCLVLLAAFVVVTYPIWGAGQIPNLLLGVSFGSWREDVARQQNSKEIQVEEVGNSGPKRS
LFNESFLAFAAIDPAEENEKRKIRMILDGKVEDMQNAHNKWNYQRRMDHYVDLDQNRRRVRPRYESWLLQLQNPKFAGSWFKFRQLLQSWSRYKHY
DPLVMHELMQLVKKPIDQFYSDRNGGPVEEGKRYKTCAVVGNSGILLNRTFGDFIDSHAMVMRLNNAKLLGFEKHVGTKTTLSFVNSNIYHACSRR
LKCFCHPYGEVPLLMYLCQVQHMMDVAYCASSHKTPVLVTDPRLDNLCSRLVKWYSVKRYVEATGHSVKSWSHHNLGDFHYSSGFQAIVLALGICD
KINILGFGKSPDAKHHYHTNQKGELDLHDYAAEYVFYDDLVHNRTSTIPFFSEVPDFKFPSVDVFL

>Chromu_2511

MRTASIDELRVLAPNATVDYCRKLLERCDWDVTAAACEMLVPPGAPPSPRQHAAQPQLGRLEALAEKILRNRACSVEKTRGVRNHPTARTCKEHHE
DRGGRSIDLERVGAQRHGNSQKLLVDNINAIDSWLSNASFAADPALRWPGPVRELNRAHVEATSDPIMSWMVPHVRRDGPLVRAVPHGGKHGGALG
TRYQPQRVRERELIDELTAPIFIAAERPRKLTSEVAPFGMRMGGKSEDDMVSSIRRYVANSSQPRQCRYRTCAVVGSSGALRGTHHGASIDAHTAV
IRINAAPTHKHEAAVGRRTTWRVHNSEKPFMLAANDVPELQLVICHMAWLGSCQHQAFSGAYGTTIAYINPRFYSQLFELLGRPRDKQSPSTGLLA
IAIALGTCERVTLYGFGAGGGGANKRCRHYWECTAWEDESKYYDPLHTFHDWQAEERLRQLWLEAGLVSLGTEAVATEAAGGGIDDVGARNASEAV
RRWGTVRKQWARDLRALQRARQARRQQGHKAGDGRDLVTTKELLERFGHAAGGGGSGDDGNSGGGGGMPATAGGGTRSRLKGGRKGDRGDRSVGEV
LEAGAEAGSVAGREIRRPLRSQAEMAEPFTRAVAPPPPP

>Chromu_2559

MSAVSQQQALKAAPNLQPFYSIHPLDMHALELIQTVAKISSKDSSCTACRDAAANRAALQVKPRVRAGLTADGTLPQPRCDGSISPDRSQLSAPPF
AAAERPRRLIEEHAPFGLRMSGKPERQNIATVKRYLRNATQPLVCRFRTCAVVGSAGRLRGSRLGRAIDAADAIFRVNAAPTRKHEADVGARTTWR
VHNSEKPFFMAALDVPELNLVICHNRWIGACQHQAYGGVYAERTAAINPRFYSELWSLVKQGRRKSTKQQVPSTGLLAIALGLSVCDNVTVYGFSR
PSDPQARCSRHYWECPKWAERERYLDPKHEFHDWLGEVALRERWIRDGLITDGLNRNRPER

>Emihul_229870

MMAYACCVKRRVASAGPLSEHNSAFDIRAVREALEALCAQRPPFGLLADLSTLEGILHGLGLPSEAATRGTGGVPNASHSRHSRTAAAAAARSEAC
RRLAARYAYDSPVVVGGGGRARGGAASRCAVVGSGGTLVGSGAGAAIDSHEVVYRFNLAPAGERWAGDVGTRTTFRLFNGQSRAGARRVNDSRGAT
HLLYCPFDRWLGKCLLSGVRWPPKGAVAAKGSDRPRHPRGTGSWLLVNPVFSLRTAEQQWAHGGRRGRMASTGLLGVALAAAACDHVTLFGFGNDS
DAGTAATCAHYWECSRNQSRYFGGKAGYHDWHAQWRVLSDWVASGNLSYWTRRD

>Emihu1_461764

MPSTAASASGATASAASAAADATSQPSLSLPLCESTCSPPDGAAPRCSGRGSCVAWHGMEWCECDASATERYVGLRCDRRLAAGAECQGSCSGRGR
REYGEHIDANECVFRINRAPTAGYERHVGSRTTYDFVNSFPHVRGLSILPRTHTKLIHGMVAEPWAVRDAPQQRYSGFDEYLVWADGHAEVVARNP
GLDAYFLDLEWLRSSWEAYYAHLVRLESPRASRARPSSGWHVTRLALGVCRKVRLYGFSLEDGDFHYFDSSVQATVTPPMRDLRYGYTHKFAFEHA
VFANLSAAMPDRLELLQ

>Emihuxl_98204

MPLPRCNASAPDVAPKASMRQRLCAKPEMRAALSAASVPVLWSRDLVRSQKMFMNEKPFAASKFQPFIEQLPRNSLMSSAWPDGCEMCDTCAVVGA
SGSLRKFEHGAQIDGHSLVLRPNWIINKGFEKNVGTRTSINVFFGVEGMMKHFERHQRSVPEERRAIGLITSNSHLSVSSFFRYLIRVHKSTALNK
TATAAETLVTPSRVFLITDSIYHKALAEICRATGEGCMWQRTSGTMRPSTGFIAVIIALQICRNVSLFGLTDDPCQPFHYYGKPKANCTKAIPAKN
DEPLHWFEKEHEIYARWHREGRLTVYS

>Emihuxl_30350

MIKPTIERPSSVASVSSPCRSCSRGPLLAVLLISLVVTNAEEVSNLTNVWPTANGRPLITTAVVSSTPSNLRIAYCLALVGAPTVAMAAQHLAAIS
LLRQTGTKKDVLSMQSPNSILRLGDALRTLDTKVVFVPHVEGKCRTSSSAEQSRFRFSFSFHILRVYEQTQYDKILYLDGDLAVRTPPDRLVEAWA
RQGTTELRTPTGCNRVPDQKHYNTGVWGITPSRDYAARLYPWLAAGKFPCGIGSQTWVEFFGANNSWTRASLAWNLKADQGTSRCMRKWGLREAHV
VHWSGNRKPLGLRTADPVEARALAAYQAAHLRWLTFLDAPAVDNIRIWHNRTHTQRSGATNQSALSMPLPRCNASAPDVAPKASMRQRLCAKPEVR
AALSAASVPVLWSRDLVRSQKRFMNEKPFAASKFEPFIEQLPRNSLMSSAWPDGCEMCDTCAVVGAGGSLRKFEHGAQIDGHSLVLRPNWIITKGY
ENKVGTRTSINLFFGVEGMMEQFEKHQRSLPEERRAIGLITSNSDRSVASFFRYMHRVRKSTVLNKTATAAETLVTPSRVFLTTDAVYHKALAELC
RATGEGCMWQRTSGTMRPSTGFISVIIALQICRNVSLFGLTDDPCQPFHYYGEPKATCTKAIPTKNDEPLHWFEKEHEIYARWHREGRLTVPT

>Emihul_36797 (Emiliania huxleyi CCMP1516 _GT29_02681 XM_005789169)
MPLPRCNASAPDVAPKASMRQRLCAKPEMRAALSAASVPVLWSRDLVRSQKMFMNEKPFAASKFQPFIEQLPRNSLMSSAWPDGCEMCDTCAVVGA
SGSLRKFEHGAQIDGHSLVLRPNWIINKGFEKNVGTRTSINVFFGVEGMMKHFERHQRSVPEERRAIGLITSNSHLSVSSFFRYLIRVHKSTALNK
TATAAETLVTPSRVFLITDSIYHKALAEICRATGEGCMWQRTSGTMRPSTGFIAVIIALQICRNVSLFGLTDDPCQPFHYYGKPKANCTKAIPAKN
DEPLHWFEKEHEIYARWHREGRLTVYS

>Aureococ

MMLWAVLPWLLRAGAVAFDARIPAGSRCAVVFNSGVLRKFRHGAAIDAHDVVIRINMLNRTGHESFLGSRWTHEFASFQKPLDGYACPADKFAAEA
PRGVVGLSFERHSPADCGRHAVCCRDRILARHGSWVLLPEAFSLACRELVVGTHLSQCSSGFAAALLARSRCANVTVFGANDDPCFPYHYTDPPFP
PDTCARALRRRKAPPGYVKYHRSPHSFNREHEVLREWRDAGDLGGKGEDTLKVVKGKENILRVSIFRGDEENDKLTVVEGDYNFLYVSVTRGGKGE
DTLTVESGDSNRLKGDEENDKLTVVEGSNNLLHVSVSRVHSQNHRRVLDMFFGGAGEDTFTVLGKTTTDSGAHGKGNRTLQGSTPLIVGLCVGAAV
LLLVLAVVVRKLRGRAAEETSTPQLDAENFRRLTVRCVPRGSGRLGAITGATSRRRRPRFPRGDAAAPDRGRYFARDQAASALRESSRPQPTSSDI
AHGAITGATSRRRRPRFPRGDAAAPDRGRYFARDQAASALRESSRPRPTSSDVAHGAITGATSRRRRPRFPRGDAAAPDRGRYFARDQAASALRES
SRPQPTSSDIAHGAITGATTQEAHKPRTTSSLVITTRTTPTSATRCPSRRRSRRCNAVKDVSSRRGSGRGDRPFDHEPVETRRRGALPLRLAPRPE
VGQAGRPLRPDASARAPGSTPARSSRATRLAAAFANGGVIQGGSQGTSASRARWRPEAFANGVPPWGAPDPVASDSNSDGDS

>Emihuxl_106270

MPHHAAEAVVAAQAWMQGARPSPSNEARQLMPNRAAMLANLVGGPGIFVDPLKKPPPRPVGCEGRLLRSPVLSSAPVDNLAYFHRTKLFKMANLTQ
VTHLARRAHVPLAYQRARTCAVVGSSSKLLDAAEGSLIDASELVFRMNHAPVLPELRAYIGSRTDVHVDPIQLHGAFGSINASDATSSQIYTCTSN
HDYPGCLTFSPKLSSVSGPHDRRTCKHCGARVLAGVSERWDRTFPGLDQYARALMDSTKMPKGRFIRPTTGFVALLLALHVCDEARLFGFGMSART
HCSQYKFAQLDEAGRPFFDMSRSDRVHNYNAEQQWIATATRNYTRTTLTCRDLPLVYEPGDAAAVVAAPALSAGPADGPSAVTAVAAAPPPLGPS

>Emihuxl_456754

MASTPCQVELLQQNSFARCTLGKTFGCTGASSIWVANCRGRFRCGPNGVPFRCGFPPGAAKYSCRCSGARRAHTPLQLTADDFRSLSPYFHTADAD
RLVASETTPRFSNTSEVREWQRLTRARLRGVWTTDRPHASCAVVGSSAALLARRLGSEIDSHALVIRANQACYCWRSIATDADPRFHPSAWRRAQR
LIHVNHTRCARVGCYPSTGAMAVLYAIDNCRRVTVYGFGSNEAQRMHLLATFLQVASGKLARLESRDGRQQYGKADYFAETAAYHDVVQEWAWLGR
LHASGALTWRGQPAVAPDADSYGRWHQMEPTRRR

>Emihuxl_245446

MRPLRLSTCPAVSLLGSVVPAEIARHIRRVVRAIRASIKVSASTRAHSALYHSPYKPPLRQRGLPLLINIEARGRRLFAMKPHAAAHHPLHPVLLC
LLIRIRQAFLGHLRSRRLAAPLENARSCRPDLLTGAGIWLATSAPSTGIRMQHRHRVWQHRLNAAPRGRCKQGMFPLLLCCSLESRCGLRTLGVRL
SPCCSIRSPSAERLLKACENLVALKVSLEHVVGSAGATVTELCEKNPPYGFGPLCRAADSSSSLRMLRGLRDPARCFYRSCAVVGASGNLLGSRYG
AEIDSHDAVVRINLAPDGPMTARSKAAPHRHEPTWISDGYGYLTHYSRFWLAPPHGHGSHPNMSGEPLLAVVCHEPGRNMGRCRAERLAHTFAHSE
SASYLINPGLLGEIASDEFRGVRGQKTPSTGMVAIALARKMCGAESGASGGYHNFSAQAVVLRRMAQRGKRP

>Nematostella

MMMVMTMIVMTNNDDGGDDNDDYDAGVASDDDALEEMLTKDKKLEHGFSSTMDLHVQRLLGVSYHQVSVSKVKKVTIAGITLVAIILFFSLTFHSA
RKSAEQEAAFVDKRTFTDVTMTTTQPMTTRESQALNGEQSKTSASHILTTKSPQLTATKKVNKIQTTHQQNPENRYKPTVSKQGHHTHREERIDNV
PLTVQPKLNTDFVLAMNPRIPIDLNIKCRTCALVTNSADLLGSNAGSVIDSSDCVIRLNTAPTAGFELDVGGKTTVRIVSQEQLGELLHYVWREPE
EMRDLSYLVLYGPRNALCRNCTFFKMFQKVSGEYLGDKFVRYTKAYDANLRTGDIKKTAKESDVTSSVVSRLSPRFHAMNVMHRVCDNSPRHAPSR
HAYPRDNSPRHAPSRHASPRDNSPRHAPSRQNPQHNITLEYDTKRHDTLGYGTKQHDTHGYDTKRHDTLGYDTKRHDTHGYDTKRHVTHGYDTKRH
DTHDYDTKRHDMHYKTTRHARLRYKTTRHARLRFKTTRHAIQNDTTRSATIQNETLGYDKKRHVTLGYDTKRNARLRYKTTRHAWLRQKTTRHARS
RHKTAVITRFTHSFLCSGPSFRKSWQSHVKIDCDGHREKVLRDIRRYRLEERVYDDWVDKYDLEFLYP

>Orbicella

MPLDFPRYYAGVADEDMLDEFLVGRRELDHGQSSTMDVRRFLNVKYHQASVGKFKKVAAVSTIAFVLIIVMVFTFGSPPTKKIAMKTLPASTEAKE
PGAEKHRHSFVLATNPDMPIDLALKCNTCALVSSSGMLLGSNAGSQIDSADCVFRLNSAPTLGYERDVGSKTTVRVVSVTGLKSLIRDAWQKTLAS
FEDLDYMILLGPEELLCENCTLTNLYKKFAEYLHNTELLRVRQETYQKAGPSNLYKILHNESRSTILPSNQSIRHGTGKPLSKVKFPPPQGFLHVN
CSRLFLLRVSKLLYVGFIRFRSRTIPLFYWVPDSWRHECLSREGRVQQQVLQGLKRLRQESEIFNSWARQYHIEFFYPSQKVT

>Acropora

XTCSVIGDPHIITFDDTNKAGYTMHMRECAYVLSEHCHMPNGTDRGFSIAISSRFINVKYQQASVGKIKKAAAATTIAVLFIIIVMFTIGSPRTEN
LPLKSPLPDTTESKREEAAKTQLSFALATNIGTPFQLSLKCNACALVSSSGILLGSNAGSQIDSTDCVFRLNSAPTLGFEKDVGSKTTIRVLSAAS
FKSLIRETWKRTLNSFEDADYFILLGSEKLLCKNCTLSKLYERLSHYLGNTKLLQVTKENYEM

>chromera

MQAQAAAESTPPEPPIPNKAAGTGKKTQRQPAAQKKKKVVLNEELLPTGLSIWEVPTISRRIADLPWSNLTAFRAAFPNDTSSSTIKRVRGTHDYG
AEEQLRSFFIQHGLANLRFPGVNATQRVEWDRPPLLGLLEGELKGPVLDHVAAPKNGRLPFSIPSKLYDQWKGLVKGLLEKRPRFHLDSTVPKGSE
YPTCALVASGPDMLNAKFGAEIDAHDVIFRMNCAPVNGFESHVGSRTTFRTTYPESTRQLKRHCESDSTDLKNETKTDIWCEGIVDLPSEGGTVIG
MVFHPQNLEYYHCGALGCPKSSKYKFATEYVPYSADTFTVLNPEVIWIAQRLVPRNETQGWPSAGGILVALTGL

>Emihuxl_102768

MYAGKVHMGSDGRAGAGQASGGVCVPPADGSASALVQHADMDQLCRHNPPYGFRALCKERSAVAMLRGLSNPRVCRYKTCAVVGSGGSLLGARLGA
SIDAADAVIRVNLAPDAREAARARSAPHRDLATWVSDVGSRTTWRVLAMEGYGYLNHYGRFWLKPPLGHGKHDNMSGIPQEPLLAIVCHEPTAGTG
RCRAERLRQTFAHRWAASYLVNPLLLREWSRRLFAGVRHQRVPSTGMNAVAFASQLCGSVHLYGFGNGSCPAACYHYYDCGEARPGGGVAQSYMFG
GDPGATGGYHNFSAQAAVLRRLAESGAVHAHWGTCGHSSGDPPDPALLNRRGGGSRGAAALRTHRRRGSSRTKGQIDNV

>Athal_AAF99778

MRSHQAGRKLPLLQLLGCVAVFSVFVFTIQSSFFADNNRKLDLQPEDIQILSDFQSSVQQCVANRGLGLSAHIIDHCNLILKFPEGTNSTWYNAQF
KVFEALEFKYNVCEAVLLWEQYRNMTTVLTREYLDVRPDGWLDYAAMRIAQLGADKCYNRTLCEEHLNVILPAKPPFHPRQFHKCAVVGNSGDLLK
TEFGEEIDSHDAVFRDNEAPVNEKYAKYVGVKRDFRLVVRGAARNMIKILNGSDNEVLIIKSVTHRDFNEMIKRIPNPVYLFQGIVLRRGAKGTGM
KSIELALSMCDIVDIYGFTVDPGYTEWTRYFSTPRKGHNPLQGRAYYQLLECLGVIRIHSPMRSERKEDWSSVPSREMISRAHTAALRLQRSQQPT
SSKRDGSGQFGNCKVWGDADPTKGPVSGSPDMSETRKKSNYKKWEVMPFRSLRKEARDHYIQMKGVSQYKMDGNKLDDLVCVRHPLKLDT

>Athal_NP_974404

MINDKEDDSVDLDESNRFTNEDLEALQSLQNGFQKTDLMMQSANGLGLQAAMGRDYCKVSINFPKDTVPKWLVIILVLVSSKYGLPYYYVLDYVQQ
QKDPKSGELEGLSYEFDLCEAVATWEQVRNSSTILTKEYIDALPNGWEDYAWRRINKGIQLQQLNTCLVDCSQSQYTNNRCQNKSLCIEKLSLVLP
ETPPYFPRQFGRCAVIGNSGDLLKTKFGKEIDTYDTVLRENGAPIQNYKEYVGEKSTFRLLNRGSAKALDKVVELDEKKQEVLLVKTTIHDIMNKM
IREVPIKNPVYLMLGASFGSAAKGTGLKALEFALSTCDSVDMYGFTVDPGYKEWTRYFSESRQGHTPLHGRAYYQMMECLGLIKIHSPMRADPNRV
VKWVPSRSTIRSARIAAEKLLRRVGAGSADPLASCSIVKKRNKNKRPMVSHLRKPVSDHQKFVRSTSMYPVEHSPGHGQLCITPAD

>Oryza_Q6ZH45

MRVLPLALAAAIFSGVTAILVYLSGLSSYGGARVSDADLAALGALQSGFSKCVDANGLGLKAIPGEDYCRVVIQYPSDTDSKWKDPKTGEPEGLSF
EFNLCEAVASWEQVRNSTTILTKEYIDALPNGWEEYAWRRINKGIHLNKCQNRTLCMEKLSLVLPETPPYVPRQFGRCAVVGNSGDLLKTKFGDEI
DSYDVVIRENGAPIQNYTEYVGTKSTFRLLNRGSAKALDKVVELDETKKEALIVKTTIHDIMNQMIREIPITNPVYLMLGTSFGSSAKGTGLKALE
FALSMCDSVDMYGFTVDPGYKEWTRYFSESRKGHTPLHGRAYYQMMECLGLVKIHSPMRGDPGRVVKWAPTKDTIEAARVASEKLLKRPGAGSEGP
LSSCTMIKKREKGKTPKRSVVRHAALKHLEYMRGATRYPLERNAGGGYLCMINER

>Sellagin

DASTLDSFQRQVQKCVERNGLGLTATVKDACKIVLKYPEGTNSTWRNEQFKIYEPLEYEFDVCETLLLWEQYRNVTTILTREYLDARPDGWMDYAA
KRIAQLGAANCSNRSLCEEHLELILPPKPPFWPRQFRTCAVVGNSGDLLKTEFGKEIDAHDIVIRDNEAPVTTKYAKHVGMKRSFRLMARGVVRNL
LEVAKGSDDEVLIIKSVIHRDFNAKIKLLPNPVYLFQGVVFRRGAKGTGIKSLELALSMCDSVDMYGFTVDPGYTEWTRYFSAPRKGHNPLQGRAY
YQLLECLGTMRLHSPMREARKQDWSVIPTRATVNAAYYAAMGLKRKPGKDHGAFKNCKVWGSSSRNGRLSGSKDMSETRKNSNYAKWEKTSVNSLR
SQAQHHYRAMEGVTMYKIDGNKLEDLVCIRPQQEEQRSHGK

>Stram_2792

MGLAKNVGQVKDRSSIFYSMDDFDPNTTEGKKNLEKIAFGHQEKSSENKYPGFFEHHGRSGSSMENLRTAHRLLQKMFARNPPPSKGRWKTCAIVG
ASDGLDGKNLGSEIDSHDAVIRVNDHPTIGFEDDVGRKTTFRIIVNDVLYSILSQVGKRTLAKVKLPSKCIDVFSGSATFNYWASTERTVNYAKNR
NPNDWMGCYSGYKENETLLWHGFGFFPNLNLLKALVEHDDDVTKLNVWIAKEGIFRDRFRAVFPYTNIGSIADSQGEKVWRAAFGFCDKVSLYGFS
FRGDAMGYKYYWGDAKSSKKIGSPYFLAFMRSKWPNSTRPYDPDKHCRRSNIARLAVAGCMGLPLRFGDGPEEYIDYLNGFSI

>Bathy05g05030

MKPPPSSSVSSLSSRREAKRTRRYVMLLAFVSFLAPLVYHQNHESHPQTKRGESRISTFAHHHFSSLHDQHRTSIAKSYGHVGQIEDDDEDDVIAH
NNRNDEGSSSLSSSSSPSNCNKRGIVVNNKCQCLKLWRGRTCEEGPNIFSFKSKSSEKLPSSRKVDIPLQFEGDFTTNKEQLRKTCEDGNIKVFLP
GKVPPMRVIGTCKSVEQAGVPLKDVVSKKPYKSCAVVGNSGMLAYGQNGKEIDSHDVVIRFNGAPTKGLENRVGTKTTFRLVNSKWLDFRESKDEV
ILWNMRGAGALEDYIKRRAEKGKDEKFYLLSSSFVNYVGEMAYQLSNSFDNLPGGFNAGDYTPTSGWTGLILAVNVCTEVKLYGMQISEAQGVPYH
YHNRCPQPYGERDQAEWLMFQRFARHKLASFKEPCIEECHTDNPDDCKRCMSERSDFYGEEATKEGRIAWEKSPMPHYCLARSMLLEKEKKHMEKG
GQSYTKEERDKFLSEINKKIAPPAPLRAPRKRRPAK

>Bathy16g00850

AKSLGRRCAVVGNSGSLLDHTYGSEIDSHDAVIRFNAAPTKGYEKHVGKKTTIRVQNIDNLGFREPKDEMLVFTARDEKTFRHFSEYQKKKYLKHK
EKTHVQRAFNPEFWCHVWDWVDRRKLKPSSGFAGVVMALKACSGKVSLYGFSHNATQFHYFNHLDEKVTTQE

>Bathy12g00960

MRSRVAEHRPVAEDLESANSTFGPKRVDYEKNPPAKGKAMTRTKRRDKILFVVLLVLCFACYWNQPPLSAGILTPNESYLKQFKRDSDGRISNLSD
VREFREKEVEKEKKMSPNAVRVKYSGGEDADDAKKTPASTSTKSHATTTRNSGKSHHHPQEQHHAHSFIPVKPHVGAWTKDAHLKAFEDFMVPSRV
EVLVQSNKGGGEEEDEEDDEEKEKEEEIEREEEEGEEEEGGVAIAGDAGGGEKEEEEEHGGEEEERADGGEEKRVAAHAFCAPTGKSKAAAEDIKS
QKLAFKDITDLKDLMDGIQVPKNGLWKAARAPQLMVHTLPYAEKAFGKDLAENLEADGCIPEMDFTRRGKTCAVVGNGGVNMRDQNQGYGIDGADI
VIRFNDGPTSGFEKYVGRKTTFRLINNQWSRHVADKGPKGPWAESEALLLFGSGAKRSFRNVCKKSTSPILYMDPTLSLRARELYRRVYARLSGAN
LVNAEGRNAPPSGIEGLLLAFAVCDSVRVYGFHTDIPHHAVHTVPHVLTDFGENGRWNDDMFEV

>Chlorokyb-8010

LLLLDRMAAEAKALVAGLDVRYDGPLSLHRACIVNTSFVELPNGVTRGKRKEGDVYRVDNMLYTEFPGWDAQERYGTCAIVGNSGALLRAEYGREI
DAHDMVWRFNQAPVKGYETHVGARTTHESLNGYWTKQLLDDHRGYRWNWRSRETQLVLFEMFDPSAFGLGKSRLQVTEKERWWRLSYVKLTRSHPD
HHVLALSPRLVAVGFQLYRELRRRFHRHDLGRYTGEKPMSGFYVFFFALQVCDEVDLYGFQPWRDSDTKSGPMVTKYHYFDSAVPRPGSHSFDLAR
YVYQLFAMQNENIRIFD

>Chlorokyb27068

FTVNKLRLDYLNNSGLNYTYTSNPKVSGKRRAVNVXVSQELYDASPDVDAFASQLFDRCAIVGSSGVLLKYDMGEEIDSHDCVIRFNAAPTQGYEK
QVGKKTTMRLVNTQHAGFQEGKEVTIQQMQSQAGIRLYAAYKQQNPDARHYAFDFDFSRYVSSNLGTLPTGGFFAVFLALQRCTSVRLYGFHFQPG
FGIGHHYFNDEEPVTGEKAIHDYAAEHEVLTKLVNSGVITLAEPCVAGCFNETGIRCDNCPRGTFCECGNSNPLPIALPGFCRASGQQHCFRQCAD
AQECPGGATEFNEPTCPASVESSSASELLCRERTLGGVLM

>Chlorokyb-9580

GMFXGAPFTLRGIPEAFLPLPSVTSTQPTTPILRKKDGTSALEWFDFRSCAVVGNSGSLLLSKFGSNIDSHSAVIRQNQAPTRSYEKHVGGKTSIR
MLNKIWLKRYALASFLKWLPLEPNMSLAASRGEPPLMARLRAAHTLSSVIGSGVRKLMAAFKQRRKCAGLNSRFEGGSTPSSGIYSVALALALCER
VTVYGFGTVSQGRSGRSVSYQYYRLRGTERSSGNPVHSFEAERALIKALAKDGYLTFCTEESAKTCGAHAVQ

>Chlorokyb-10426

FLDQKQKAAVHFTARQNSTIELSRVTDKLLQALPQHDEAFPLTTYASCAIIGSSGILLNYAHGEEIDSHDIIFRFNSAPTQSFERHSGRRTTHRLT
NSRNFAYREYPDEVILVHLRNPTSLRQLQDRRLRRKDKRIYGLNPSFHRYMDRSFNFLSTSGLNGIIMALHKCARVDLYGFHIHPQHGAYYHYYNK
NDVAANAGRDDEEWAVVKALVENNFVTFREPCVLECHNVNPDVCKHCLAQA

>Chlorokyb-4572

MSQVGYVRVHRFEPDLLSRLNIPDGVEPPVVPASPSMPWTCDWLACQPTCSKRGKVNDEGECLCEAPYTGVDCEKDRVHSEAREIMDGLDLDYNGA
ITMSQTEVKTSPSGVLYIPIPPAEGGERPTRYIITPSQLRHLPSTEWRPAFNTCAIVGNSGSLLYEQYGKDIDEHDAVFRFNQAPTLGYEVHVGSR
TTFEFLNSAWVQQLLDSTRSRSHGHIASAMPQWNWRQNTTGLILFEMYDMAAWPFRTRAQIEAKEAWWRRAYVRLRTRYSSRHVVSLSPRLVAWSV
GKYEELRQRFRDQGMGEFRGEKPMSGYYATLFCMQACQQVSLYGFSPYRDRDKRDPLAGRYHYFDRARPRPGSHAFDLTRLAFEAFARAGDIVEVV
D

>Chlorokyb-30380

MRISNQSRRNKMFDTVKSVWHGLRHMRTHGDPMKLSARAVRRKLVTEGGPPLPQRLTCALVGNARSLMRNNNTHTSGRGTEIDGHEAVLRLNQAPT
RGYEKRVGPRTTHRLINHRWARAYAENQDRLALESGVTLVMSRTDPREFLTYAQMFSSAHRGAQALLLHHDALNATGATLRHLKAKLEHMRGRPYA
GKGSPSSGFVGVWLLLQMCQQVDVYGVGDEAPSKTGKRSAWHYFENAKFRASREFGSDPHHSWELEHHVLQIFDAAGMIRHVLAPPAQRKARCCRS
RSGTSRANRTALARLMRAMCWGLWGLMCVCL

>Bathy16g02160

MHPHAMVPKSKEKGIRVRRSSMEKTIFCVLIVIVIWRGIAVSGLFSDSDSSSSMMMGRQNENDDFEIGLGGGFKRSNNEDEETEDYGGDGAEEKKV
SDDISGGSSSSKSKSNSNSGSKRSSESNSESNPTRGGGGGGRGGGKSGGTSSRSNEKRASGGTAARSNSRTQKMEDDIFETLRDDSQPLVTHKRLF
IVNKPVNPEDPKDSRTKPVLQHWLESTTGDTNETLIYHFPRSLFEQLPLDEPKFEFKTCAVVGNSGVTLLKEYGEEIDNHDAVIRINMAPIRGFEK
YVGKRTTFDVVNSHNVREILQGVRRWRPANEGETKLVLFETASHFARYHLCQPLLKKYGAENAILLNPLFSNRAHSLWIKLKELLEKTKNSQYNRK
PMSGFFAVLYALQMCEKVDLYGFDAYTSRKKSYRYHYFDNVQGFTDVHSFDLAIEVFKFIAKKGTKGGLTINV

>Emihuxl_210127

MPAVDCAPTPNATCSRGCLVWAGTARTLADAPQVFRRARDTGAILGRDGSQRTLTLAASQSSRGLLSVTAQADGSARIKQASWRPLLRAPSGKPSF
SFAVPRGASLFPARDPLAVGRRFATCAVVGSSGAVLSPRPPCGRAIDQHEAVFRINTAPVRGFEEYVGSRETFRVLNGPYEQYVAGLPAAYPRGWG
TPPPDGSPVRVLADVTSAENARQWAALAEAWRRAAGPRRARAQLLLKDHAVLHYYLALLGHPRHRPDYPSTGLLAILVASLRCDSVSLFGFTFHSG
RNRAQYAAGSCEHVYHYYERLGWRNQLKSHNLTAEADAVRALEAAGVGQADVDT

>Bathy01g02080

MDKETFRMTPKRERRARRLVMMMMMSLLILGLVLMVFRTTAATTGGGENGGKGRYDHPSSLAFSSRVSTTFGGGGGGGGRDAMMSENVKDGCHCAT
TTRERQQCPDCSEECESMKSSNTAIDESIAGCDEKALKKASSSKIDVEIAALRAQLREANEKVKELQDIMKAKCHVTDYTQCSSACYSRVMRIKEQ
NAQCQKRLQSRGCTDCTVVAARSISKDRIATRRAAYHLIATALKAESKCGGSAGTTIDADRERMKLDVTDRERASEIQEKMTKNQREEITGEADMD
DFLISTIDRSTLLDGALAHALEDLSIGNEEDKVTAEAYEKALKEIRAAASAGASAESKWLEQLRNRAEDINREANLKDLMMKKADVFRRDMASELS
FPSIYGIPQSLGRALYKVANTGAKHAREMLKLAPRYNRDFKTCAVVGNSQRLLLDLNGKEIDGHSAVIRMNNAPTVGFDRFVGNKTTLRTLNSIWT
QRYSNTQIDSNKVANVMPLELGVTLVGTRVVPGDHFALQQRLRKSGRGDVSFVKMTDPSIAQAGKLLQNYKTALESVRKLEYAGKGSPSSGFLAIF
AALQICDQVTVYGVGTESDLRSGSSQWHYWEKADYYQTSREFGEAPHHSWELERDVMLVLEATKKLRIVTPKSRDDQAATKAERIATAAILASDAA
VRRHRTKANQQCARVGLSVCGCDAESDALHGGSGILEVAGKVSHGNRPAAKASVTLQKFDKTVAKKDDVSVVQRAAIALAHSYHLELPGVAAGSTS
KKKKGTSASSSEEADGDDASNEEESSDGESNDSVLDAANSDDVVSEDHHAESNNGASHGGHGFMSNFFHGNHGGN

>Bathy08g02300

MRGSFFTTNGSAHNARFASGTTGVHKRKKMMLLVLFVFVVCLVCGTWTFRGSSAAGKNDAPFTSSSYSKRSGGKGKQTLLDSFEKEVHEHVFEPIA
HGLFGGGSDDDTVSDEDDDDDDSERGGEKEEEEDETIAADANADLKYREENEKAEMHDEEENQEADGYEVATMEKNAAIEREVRLEETARKASNMP
KAFAIHRGNAKCANAPKEMLPRFSKQIGLRKKEGKVLPAKAGYVGVSRIDGATKGTVRVRTENGPRTKKVESSANPTVKNRDEEALIACLEDLSKC
QLDSENDSIRKAAWKLGGTMDAKLGKSLAGIERKKTCAIVGNGGALMAKEYGQYIDKHDVVVRINVLDNNNAKFNASLGQKATHRVLSYKMSKDVC
CLQPASKHPPDNDELIYLAWFPAMRKQLISRLAKRQKRKVIEMHDAHLKSIIQSFKKMREELVRLGFGPFEDWEYMTSGMHAVLTFARSCETVDVY
GFTTDSGGKEPYWFTGRKVAPRSGRTQHAWDHERMILRLLAASGVVNVCT

>Bathy14g02010

MVFLFHVTAKNNTLLICLVLGVVRLVSFHIQLSHSNSKGDSSHGIPGAPTELSCKTEKTEKPGIDELPILMPFDRSRMGKRGYLRKENLFIHKANF
LRYHSSTVFKSRNFAANATEIWSKYAWDRCALVGNSGSLLSADFGKEINEHDIVIRFNQAPTKGFEKFVGNRTTFRVLNALWTNIYAKNVKLNKDG
SLDSRVNIRLGEIRSIPLEMDATLIISRTSVENFNFVYSALQKKRMDVRLLLLAPRVVTAAKWLLNEYRRLLCTHGYGPYTGGHTPSSGFVAIFVL
LQLCKDIDLYGFGDSGLSARYHYYTGGGHRNSGNIVHSWVLEMEMIEALHRNDLLKIH

>Bathy02g00330

MSFSTFIKIIQRRQNRLIKMGAPPPKREYRRRRHRAKFLLCCFSFLLFSLLCAFLSSSSSLSSSFRGNKVATPAIENDDDEVDYNNNAQQHEQHEQ
QNDDDNDDDNEKEEEEHNTSILLNNNNIHHQKRNNNKHRIGGSGTGASKQLGFMNENLVSKLKELGEKRQLEEEEEEEEDSKKPSFKGSFVMNKRR
RKMNEEIPFPRKKLRDTLQHYSLGKAKRDLIRVIPEEDEVRLTLLTRSNRKSSSSNNNNNSFRKKNVCAVIGNGGTLLAYDLGEEIDSADVIIRLN
AGPIKGFEKKVGSRTDYRLVNRLHMGFRETKEEMVLQHCTTPDALDEWTRFKVKHEDDINVKKKLFAIDPDFHELAISYTDKGVVSNGFYGLLLAT
ELCDQVKVYGFFRKWKGNVYYHYYNGEAPNEGQSFRDDKEGDRLEMWLKERHATHKMGEPCLQQESDEECKNCAPGLVCAKTAWHPVPKPGYCYDE
KHPETPANAKGLPRANCARKCGNSDICPGGKRGFCSKSLVETSECVFS

>Bathy14g96

MLVGTPSKLSRIGGSPSNKKRVWMMILKRWFPLLAVVMTLFVLTWTEETTRGGKDRGGGKEFMTRSLDFDEKDSSFSGKDVLSSFTSSSSDGFNGG
FGGKRSGEEEEEEEEEEEDEQSGGAKSGSKNTNNNNNNNNPYSLVATIEDEDDDEEIDAEDASSSERSRSSTSSNNNNNNSGKNRVVGNKRRKCGK
HGAYSEKLNECLCSALYEGDGCERVKPMPTEFKGFDCLKTFTGEFEGDLAINRDRVVRDKQVAVTLPGKEKDPDGGYRILVPNEEPLFSQFAKILP
KKDEIGRSFFGTCAVVGSSGIVLNYEHGGDIDDHDMVFRFNSAPTRGFEKHVGSKTTYRITNTQNWGFHEPKTEESILIHFRAKSAIKGLFWNSKQ

KKPLKLYAFAPDFVEYVAQKVNFLATSGLYGILLALQRCHSVSIYGFQVSTQHGTLYHYYDPCDVPANVERDDTEWIVIRELAKHGFISFREPCVA
ECHETKTQCDECKEANEDFTKKKVKLPSRAKCDPNAVSKGHLEVPWRLERRQARRRGGGHNK

>Bathy01g00040

MRGPDSSSTKSKAQRRVWILAFIFVGVVLFSFGNNFNNEDEMNGTVGIKEGERSTWENVKRMSYISSSWSSTSNYGDDSISSTQPRYGSESNEDDS
AEEIAEAGESNERYDGDGEMNTHAVIKRDNKKFLNNFNGAVAMHRGSCKEGSQIQTPWIGKDGKKISLGSCTKKVLAVLPDTDPLAFRRYNTCAVV
GSGGIMVHKSVAADALGEAIDKHDAVFRFNLAPTKGFEKSVGSKTTIRLINRKHFGFREFENETSLQHTTTPDVMKQFWDYKSLHPDLPTYPIDGS
FYKYVMSDKDVANSRPSNGYLGLNLALMICDKINVYGFARNWKQRNMRYHYFNREEPNESQMLRDGKGEMPLLEHLVKKNKNRIKMVHT

>Bathy01g05530

MRRHAIEQVVLSSSNRLSKRRPSAKRFKTDLIPMFVGALIACFFLSIVWTVRIQKVEEKKHGGKDVLDGLVPNTNTKFLKSTPPEEFSAIPKDDYA
NDDDIDDDNEDDDNERGRDDEEQQRQEEEEEKKRRQATDEYQKNIENRAKATSRMNEHTREEVIEELFEKAQREDEKKHDAQVFESLSLDGEEKEE
VKKAVLKKEEEVVQLSMKPSLVPVVKKEKKAEKRKETEVEEIEEEITDETKASVAQKARKHKSLHATTLKHLAALARTPTVLSLERKVTAHGKITC
GGTEAVVFKTWHNFFTANENITRTLPPVEPRITEVVNHAQRYCRHRGECDFTKIYANVKLKNDENMNKKQPYKNDEVLPTCAVVGNAGSLRNAKFG
EEIDAHDIVLRFNNGRAKHFEKQVGTKGHLRMYNGPYVEGKMGGEVTIAQLRDSSVNHWIRQYEKHRETFPESFIMDPEIICRAWDLVNREGEKPS
SGMVGITFAMRLCSSVDIYGFSAESYFNETERPHYYDWERPKLGRENVHPFEAERKIYKALQKEGLITLHEVEVGDENSNNPSNDSSTSSSSSSNS
NNHDEN

>Stram_1410

MYSVWKSKLHDTLLHFNKYKIYGEYQRRRYPRQPKFYIPNQQFLKYMEKRKGRSPFILHAVIFAINACKNVDVYGADGYGGNSAYYGQVENSYRNS
EINFLGNALRSKTINFHKREGSRSNIFNGVSKTFNKSLKFGKENPISSKWGKVVSNGSKSLPRFETCAVVGSGGILKTYQHGPEIDAHDAIFRING
APTRGFETIVGANTTFRVSYPPHCGAINGLERSGIICNDYGTGGFKNLLRLWEYGKSKMKATCVNLQMGNCRQTYDPKFVIFTSIVRNELLKKVAR
PTGGMVAIYVALQACRYVNVYGFTIGQAHKRKTKMRGGYHYFDKSDLQLNKPN-VHTFSQEGEYIEELEKLKYIVNRT

>Stram_5962

RRFKTCAVVGNGGILLDNPYNGYTIDQHDAVFRINEGPTTGFEKHVGVKTTVRLSYGPSCGGMSQAEYAHNICALPLEWHSEATIFAKKYDSWELI
YKDQYAHWKNYASFTKETSKIHAEGNVSVPLEFALANLDILKDLKKTVGQWPTTGIVGIRLATEICDCVNIYGFSAGRHVNSGKETFKYHYYDTLV
LPGYQANKDEKDKENPHKYNKEGDWILNLIKSGTINDMSFPQDNKDWKL

>Guillar133129

MKFVSPQSRGSSPALLLLLLIWGPRASSEKGVGQTWIGDSFLEIEIERVPREFRHGEVMNVAVTMVGDGRSLAMGASPSFIVFIDHSPIKDEDGAT
TIHKFPPLNQYNESGYTLQLLLTQLAEGWHWISVGVLDLDQTSHRVVYEIGLDFTVVGAFSSMEQDALKTSVSKCIAPSSTSQGCRDYVQLRLSPL
SPFLTRTELAMLKNTAESYFGHVIDEGLLEMFPWDYEKSAFESCAVVGSSGHLLGSGFGRDIDVHDLVIRFNDAPAGGEYESDVGSRTTHRVLHSS
GKILEFLHANLEELTRNNETLLFRGDWKPDIDKYFQLIHPIVAEDVCNESKCGRIRIFSTYFNLLVWRWIGARGADCVPSSGMIGIMWALQSCLHV
DTYGFGRHPSVRQGPRTRYKYYGEVYDVGDAGHNWNLEEELHDRLQAAGLLVRNFG

>Guillar145289

MRARAQLLLALLACSASVRAVSHCEEETSAGLQVFRPSRNEYVRTGEVDIEFRVSRQTSSRSRFYIFLNDTEVASGMRGGEGEIISRSILLLEPGR
TSLTVIHSCDDGAGEGLDKEKYIGAALSSFTAVDPSTADSHLLQPDQLKSHDRIQVQLPERGWSVVVESSLERDPAKYSSSDDSIDIDCMVIQSSL
TDVPSSPPVLPPGTEVEGRATFSGQPAGAVTWPPLGYDHSLKRSTSISNMHFQLAVKDIQRPGIYNFTVLIDLRDYGMVETQLQFEFEGTLSDPLE
KNYPVARSSGRHVDFTFLLVVRQVKTVQHPVRLDAAGADNSFSRVKSLLRKSCAVVGNAGHIFGSKLGAEIDSHEAVMRFNEVVLAPWEAPTESFE
EDVGGKTTWWVTANFPGLGGEETPEILFYYPNFKNDVEDALYWVVNRTREDIFFISPSFVQFAWKQISAKGLDKIPTSGFLGIL

>Ocar_m43735

KRHNTCALVGSSYNLMKHEFGAEIDSHSLVARVNDPPITGYEKHVGSRPADISISNLLIPLPKELHGRGECAHLPHNHSLMVHCSLKWDKPDKTGT
AKMEEDRNNNCTSNLWEKHGLKTFTMSTPVFALAEKARQYQRAGNLTTSRPHQVATKGAASCGLRSIVFMMHLCKEVHLYGFGGNLPNEPFKYYSK
LQTMDALKSTWHDFNREKEFI

>Azospirillum sp

MKKNFRVFEKSDNDGFHVIYRVLGLKLKVFNAKASLRDLREQVEDLKSRINRDNADLVAAMWETYGCTDFLLKHFDFISHSRFAWQSYFCHFWLIW
FSCLLESGQVEKAERMIDRFLAYRNGRLDEFERFLPVSKYMFDRGIYNERVMMAAKVHEALEKSRDENLFKNLLEGKRVAVVGNAPSEIGRKKGAE
IDAHDLVIRFNNFRLKGFEDDYGSKTDIWVRGSGGSDVEDRTEPYRLVAWEADYDHWRVAHNHLDILYRQLCEGQKIYNFDWVCHMSLRKASGVDF
PTTGMVLIWEIFRKFGNFNNVDFYGFSFCQDKFDTYATHYFNDRTEAEAKRRSRVHRLDKEAEFLQRLVNGKGNFDEN

>Leucothrix

MQAIARVYYWAAQLRRSRGAYDKTTLSFFRKAYEILDCPTYCLAHARLYRDINGSIDETLLTALQTALIKTQVESQRYAIETLISESLHQRTGLVI
NKNKTSPNRTQPTCSKELLEINDMQAQWREELTDFIYQKIVAVVGNSACLRNSNLGKKIDQQDIVCRFNRYPRHASHAPDTGQRIDIWITSPEVLN
EQLVLPESVRWIIISGGDVRFTLRNWSGCHDYLKQQKRIITIPIKCWSNLVQQLKAPPSAGLLWTNYLISLGLPGKNIQLYGFGKNNTRSKQYHII
SPQYKASKRHAWQDESTVIKDLKRQGKITTTQTMTLGVFTRGLARNTYIKKHLQTNDVILVPNKEKASSLDIIVGWGRKKNTQKAKLYAEQNYIQY
ASVEDGFIHSMSQGRLGASSWSLVIDKTGLFYDATQTSDLEQLINASSLSEQQYSRARTCLKLITDHHITKYNNAKLQLPESLQRYTLPILVIDQV
DGDTSIPYALASKDNFEQMLAAAISENPDSDILIKTHPDVMAGKRKGCLTLNTPLPSNVHLLSDNINSLVLMKQVKKVYVVSSQTGFEALLLNKPV
VCFGAPFYAGWGLTDDRLPTDLPVFQRRSARPDLLTLFHATHIQYSRYLDPISQRPCELEAVLNYVKLQYKHYQKNTGKLFCFGFPLWKKRFVPYY
LKSPDNEIHFIRNEAQAQKLGCDESSRILIWSSRHEAAAKKIVDKTSAKLIKIEDGFLRSINLGSNYAPPSSLVFDSRGIYFDPNQPSDLEHILLY
HQFDEALITRAKNLRTQITALEISKYNVGTRQSENLFHCPEGKTVILIPGQVADDASIKLGCKDIKENLGLIKLVRQRNPDAYIAYKPHPDVVSKN
RKGELSWNAIESYCDQIVYNTSITDCLKQADEVHTLTSLVGFEALMRELPVYCYGLPFYAGWGLTQDEHTIPRRNRQLTLDALVAGTLIVYPRYFD
WTSRSFTTPERLTDELHLRKTNHTPSKTLQDNALVQKIDCFLNLIKAVISPSFK

>Idiomarina

MTHPTFNSREALYQCQRSLRAQRKTALLLRKHGHYNLGTLALFNVVWRKTKRPRDFLNYLNFKRDLGFVIRSREAKLLEDFLKMDFFDRIYHRITQ
FHLRQVRNFIIEQKQRKETPWVEPQYRSSMLRWLSQQHAWNSQLKEKLSHAKRIAVVGNHPKLKGSKLGDEIDDADFVVRFNLFQSSETNVEDIGT
KLDLWVTAPAYKGPQPQLTPFILVTGPAMLSKQQNWHALRYTQSPLVDTSLSVWRNLVMKLGAPPSAGVATLAFIKSQTSATIELYGFDLRPLETG
INNEVYHHAKQNHRATGRHNWSAERVWLQYFSKQHNLVSEQTHD

>Roseomonas

MDDKRLPPTFKISDIVAEFSSVAIVGNSPRLMENSHGAAIDSHDLIIRVNDGRIQGFEQHTGQRTTLRYIGVPLKERYQAFFRGFAEDSLIFTRAE
NRLILQELGCHTPARYIRQHSQVPLAAFGKLAGLIEIGSFPERPPRSGIVLLSLLVDAMQAGKKVSIFGMETEARSSGAEHFYADGRKFAGSTATW
ENYHCPMEREFETLKALAAQSLITIN

>Alteromonas

MSLEFKAITPVINLSGSVAVVGNSPKILGKGLGQEIDTHDDVIRFNGAVISNYVNDVGSKTTIVFVGIDLAYLFTDPYKRPKQSDEENQEIRVFNA
RKVVELFPSVKFVSFDPSNEERNNANKQYLSAMHLKNGGAKYIHYFAEEGPASAMYYHTANKDLEGLGLESRLSFGGPRTGFKTILRLVLSGVKPK
LYGFDIDTSLEFANHYYDDVTNEKISEYKPHDIQGEMRALVEMHSKSLINIVC

>Rhizobium sp. AC44

MDLLTPFEFVDSFEAGQRVAVVGNAPTLAGRKAGKWIDSHDIVIRFNDCRVRGFEADVGSKTDILISNPYAETRPGSYLNDLLPPVTVLVINPQTR
RGNKEEFLRWVGNNRVLFTYTPDLKIDTLDRQHIALTTGTYGVSLVANLLKPKNLSVTGFTMFAAGSDYHYWSDITPSGIKAHSPQTEAHVFVDML
NSLRVPVEATPEIFEMAKRSNKNFADHVRLHHFEPKAGRWRLSI

>Sphingobium Czech

MKMSDQEAGNFFFDAVARKKDKPASIAVIGNSPRLGQDRNGERIDAHDVIIRVNDGRTTGFEEFGGSRTDIRFVGVPLKERYWDFFRELDEPSTII
TRVENEPVLKNLGYAGEPVYYPNRDVHTHAALPILSRYVETGAYPKKPPRSGIVILSFLSPFFEKGVPISLFGFEIEPRNGGEEHYYKDGRDFGRA
LQHWDDAHCPMEVEFAVLGRLRDKGYIRFF

>Methylobacterium sal

MSVLSDWIIEKGSSDSIAIVGNAPKILEREDGALIDAHSTVIRINDGRSVGFEKHCGAKTDIRFVGIPIKERYRKFFTDLREPSLLVTRRANKIVL
DELGHDGDSVYIDDYSKIINSALPTLSKFVSIAEIPAKPPRTGIVITSLLAPLFGKSKITLFGFETDLRVTGLEHYYNDGRVFQKVVDNWQSHCPM
EFEFGLLHDLQARNYIDIR

>Salinisphaera

MLHWLDDAAIDRALDAAFRVASFGVFRIATRYSAGPLVGGRNEFASVHDAEWWCQRLAAVFGHAETIENTPREYCVIVTAPVDAALAARVADLQRR
AKRRATWARRRQRLAGRLWRLVRGTVSERRLLRELAGKHVALVGNAVSLAERDYGTAIDAADVVVRCNRGILVAEYSHGSRTDWVVTGLPISRATA
ESRGIQRMVWVSRRAKMMRNIPAWMFASGRLHMFSKARDVHLARELGKIASTGMKAIDLLAASDCARLDIYGFDFGASHSASQPTRPMSPDHDFDA
ERRRALSLIEADARLHWHP

>Paracoccus sp

MNRLGFLIARTLRNETALQSLSVPQSDLLAALKDKHVALVGNARALAQTGHGKRIDNADLVIRINRAPMPAAASHGTRTDWLALATRLPDADRDRI
APARILWMSPKRKRLDWQTAISPGFYLHPLGDYQALRDQLGAPPTTGAMMIALITWSQMARLELFGFDFFASLSLSGRRTAEQVPHDFSQESAWVQ
ELISSDRRIIRH

>Oceanibulbus

ARIEVKRVFRERVIESLDRVPQKVAVIGNSPSVLEKESGQFIDSCDVVIRINNFQTAGFEKYIGSKTDFAIFTAASKPNPEVVRLPPNRRLLHAAN
YHASKEKLQARLMAENGIGLKVSEVTSIPPTLYFYGLATLMGLPSGSWPSTGSVALQLAMDVFSTHKCDIYFTGIDFFKNTGREIDHYFSHTSLSD
GKHNSHLEANYFDRYIYSDEVIRI

>Paracoccus isoporae

MTPLRFYIARLMGNDDMLSSLSVPRDELLGTLRGKSVALVGNARALSGGTQGAEIDDHDRVVRINLAPMPSPESHGRRTDWLGLATRLPRSERARI
DPARILWMSHKRKRLDHRSAHTDGFYLHPRADYDALRDTLGAQPTTGAMLIELLLRSELARLDLYGFDFFASKSLSGSRSAEQVPHDFTGEADWVN
RLRQRDPRLKLHPPG

>Loktanella

MNRLRFEIAKFRQDEAQLSTLGLPRAALLERLESRRVALVGNARALAATDFGREIDMADLVIRLNSAPMPAARSHGSRTDWLAMSTPVPEATLRDR
RPDLLLWMTAKRKRLPWRIARREGFHLNPADENARLRRRLDAPATTGLMVIDLLARSGVSRAALYGFDFFASKSLSGRRDATQVPHDFDAERLFVE
MLLSTDPRFVHRKG

>Thioclava sp

MTRLGFLINRTLRREAPLAALSVSQKVLLGDLAGKSVALVGNARALAEGQAGAEIDAHDVVIRINRAPMPSAESHGTRTDWLALATSLPEAERARV
APSRILWMSHKRKRLDWQTATSPGFYLHPLDDYEGLKLKLGAQPTTGAMLIEMLLRSDLARLDLYGFDFFASLSLSGRRSADKVPHDFAAEAEMVG
GFLASDPRIIRN

>Thalassobius mediterr

MATEYLKLHAQNPEYGKTSLRLLGYVLPLLEDLNAKRVLDFGCGKGALGKKLQSLGYDVSFYDPYVPEFAKDPEGQFDAVLCTDVMEHIPESELNG
VLEAIASKSANVLFVISLTFADALLSDGSNAHYTIKPPAWWQQRLAPYFSNVTEVPTRQDTAVGYTSWAPKNDTVAQISQHRKLERRAAKRSELYN
RPLREIGSYLLERKKLSALADLTKGKSVALVGNAKSLREKSLGGEIDAHDVVIRLNRGPIMSTEISGQKTTVLATSIPISMGLFKQRGCELALWLT
PKRKKFPLWFLKKKYNCAVFPKSHHKALSRTIGSRPTSGVMALHSVLDGEPSQVTLFGFDGFASGSLSSDMTAEQAPHDFVSEQTYIDQLVERLPF
LTRA

>Helicobacter pullorum

MENVSLSIIVPFYNKENYAERAFNSLILQKYVNLEILLIDDCSSDNTYEILSTLYNKYSGNHKIRLLQQKTNQGYGAAVNLGIKEAKGEFIAIFEP
DDELPEYYYLILLQQILKDSAEVVFYDTYLEIRESLKTKTINMYNPCHLNTKKSFTLEEEEIQKRLCIGNVGICMGIYKKEFLIHNECFCNENSRG
FEDIAFIAQVMQKVQKIKIVPGGGYKYTKDGLTQSTNVSSNIYKIIIVAKFVLDRLDKTNTRYPSILGYLLNHLKTYYDKAAIIQNDFFKKEIWAL
SANIIKKQTIKCNHRVANFIKSIDKNCIVNVVDNYTPVTYAKTKPLAKMLDEDFSIIRSYGYLKFYLLMQEDIDTLPQSTIYNILGDIMVFANIPE
SSKDTEFKAFISYFLKHISINKIHKTISFNNFIVLVTSMGIAPLAPNHIQTHLDVCDEMTEIYQQTNDLKIKNINFFLIKTRNQTPLQEYLKNKSI
AVVGNSPCEIGRNKGAEIDSHDVVIRFNNFSLDKDFIIDYGKKTNIWVLTPALNSILKRETLQEFDFILTPISNRIIPMDRFGVLNNWLQSGIKVV
RIDCIDYLIHYDIRVMSLGLIVICYLLDKVQFSNLTLYGFSLKEQNQGVTHYFDGDPSKGKILSIHKWDKESQILKEICKILRGDNNA

>Sinorhizobium fredii

MYQAFIGPNRIADLGGYVHLMQDGEKFKAALKTQMDDHQPHFLFNGFQSLLQDTSIMALSTALANAIPVTIYWHETAWNLRQLAEKQAKNFAKARQ
LLQCLVVENWVPTSQCLHAVATMMGFSLDTFRIVYEVVDLANFSVNANPATKSEDEPLVIAGAGVPDERKGIDIFSYLAKTIPTLTNRNVEFRWYA
ATKSRETNLDVPYPKSIKWMGHHTDFYNSLKDVDVFVLTSRDDPSPLVVFEALASGHPAFAFATTGFNEMLPPEYVALDPDDMCQRIASMVENFVP
DPQRYRAIAENYSVEHFRNRAFRNRHAILRNLPDHRGEIVDLYVEDDASTLDAKLENLRKMQGNILRMMKTAQRQRVNADAKGLRLDHVAKERDEA
KEISRRLANDLKIEYFRDSRRTARESRKKSFALPFLSKPPEKLNVLVLGNAPSVLHRELGREIDKFDVVIRINNFRIRGYEKHVGSKTDYALISPA
CMPSSELSALLPSKVFVFGANLRDDYEKIKSRLTAKDRGCQVIPPPENILKSSIYVDAMRLEMDFDLAGNQWPSTGIVAVQWARDMHGKAARVHVH
GFDFYSDNRVTLTRYFDVTTKADGKHDFDREKRHLQQMVDKGRIVRL

>Prorocentrum minimum

MSEYVKLHSLNSGYGKTGARLLPFVTPLIYDLGAEKILDYGCGKGALGKRLQEHNHHVVLYDPFVPEFSKWPGEDFDLALCTDVLEHVPEDDLDGV
LGQISGLSNKVNFVISLTFADALLPNGSNAHCTVKSSEWWLEKIKRHFPEAYEVPTRQKTAISFTTWKVSDSTLARLRRVKWLSSLKNKVKLLFSW
PIKAAAAKLIGTRRLSQLCELVEGKSVAVVGNAKSIDEHLFGSEIDSHDVVIRINRGPILSAEKSGSKTTVIASSIWVSKSLFLQRQAELFLWLTP
RRRNLPLWMISISWRGVIFPRDMYRFLQKKLESRPSSGVMVVAAVAECNPKSISIFGFDSFQSGSLSNGAMTQSSPHDFDAEQRWVQENIDPALLH
IR

>Pseudovibrio

MQLKRYFAQKSVAVIGNSKNLLNYQYGSEIDAHDVVVRMNRGVTIPQKNCFGENTNVWCYSTLKLVKDIYRKNNCDYRVCMSPKSRVLTNRCSTDL
FFPITLWKRLNNELEARPSVGAMVTYLLAQCDPASVDIYGFDFKTSKSFYEKSNNTGSHNFQREQKLLQQIIIDKGWSFKDCSLEYNIEATEWKLS
AFMKKYFST

>Agrobact arsenij

LKGIASRLATDIRMEHYRESRRKAKAARPKKGIIPFFKETSPLRVLVLGNAPSVLERELGAEIDKFDVVIRVNNFRIRGFEKHIGSKTTYALISPA
CMESDDLKSLDPRNVFVLGANLRNDYEKIKTRLTDEKRGCHVVPPAENVLNPALYVDALRVDMNFDLSETQWPSTGIVAVQWARDMHGKAASIYVH
GFDFYSDNRSTLTRYFNVTTKSDGKHDFDREKQFMASLLNKGAIKKL

>Sphingobium (WP_004207941.1 MULTISPECIES: [Sphingobium] SCOP + GT29 (jaune avec proba 10-17)

MRDISNLSPREWKAIAAQAEDEEDLALAATALDHLLKQNARDDILLARLFRVYSNGGQRQKALQLAESAFADGVRGRRLTSMYILSDFTSYNLPNC
LDDIEAKLALFRHNPAVEGRAAMAYAWHGNVDRALDHARVAIARADEAATADMIRLLLARRVYQLSHRDAADRICRMGRQSPDLRPAILFDRLLVR
TGNVANDATTHQLFRQLLTMDDFPRRTELVAQHIDFRWRLNGPSPALLDEIDQHLASGPRQDVIPLTLMKLSMAMQLGDEALALQTLRSHPELARK
ASACLPVARLLRDHGTGTDAPVDPEVAAYASLYDELADSEAVLRKRLGNPDVTCAVVGNSSCEIGRGHGAQIDAHDEVVRFNRFDTSPPFDRDYGS
RTTVLVRVGNDRPEIGIDMAPNTLVLISSASILYRGRAWRAALRMRDEGHQLCVFPQRFQTQLSKMLVGSPSSGLSFVHLLKALRGTLHREDFFGF
AFVDQIGNQAKSAHYFEAAKPSAMHQWDRELEIFNAMFDDGASCD

>Ensifer

MKGQATPHFLFNGFQSLLLPNSLSALATAVTNGIPVTIYWHETAWNLRQLAQRRAGNFQKARELLQCLIVENWVPTSQCLHSVATMLGFSLDTFRI
VYEVVDLTKFKVNVEAAPKAAGAPLVIAGAGIPDDRKGIDIFSYIAKTVPTLVDRPLEFRWYAASETREDNDDIPYPKEIKWMGHSKNFHDALKEV
DIFILSSRDDPSPLVVFEALASGHPAFAFATTGFNEMLPREYVALDPDDMCRRVASLASDFQPNPSKYRAIAEGYSVQNFKDRAFRRTHSIVKNLP
DFTHEMVDLYQEMSAETLDEKISKLQGVQTNILKLMRVAQRQRVNTDARGLRLDHVLKELDQAKQIAKRLAGDLKIEHYREARRTARRSVPWVKRL
AGQEKAIKVLVVGNAPTLLEREIGDKIDKFDVVIRVNNFRTGGYEKFVGSKTDYALISPACMESAELRALDPSKVFVSGSNLRDDYDKISKRLMDE
NRGCKVLPPQENVLKSSIYVDGMRIEMDFDLAKDQWPSTGIIAVQWARDRHGKAADVYVHGFDFYSDNRTTLSRYFGVTTKSDGKHDFDREKAYLN
SLIQKKLVSRL

>candidatus

MKNDVWLIYLCCLIEKNEIDEFNKILIKYNKMYNMKDVERFLPLSHYLSQSTDLLKNIKVPYFFNDTLKKSNDVYEKLKNNENLFTDLVKDKSIAI
VGNSGCEIGLGRGNEIDSHDIVIRFNNYCIDGYEQDYGTKTDIWVRGSAGEDIILRDPSNYKLIVWEADYDHFMVHFDNLDTLSKDLSNFPNKISN
FNEETHVKLRQLSGLKFPSTGALTVWATYLAKSNLNNVDVYGFSFIGNNYSDTNHYYGEESRLAQDHDFENEIKFMHNFYFDHKRNGKELKKSDV

>Drer_ST6GNc4

MKSQRFYWLCLVLLSVCLLLWYRYMMRSGFAMKTGLQGYVRIHPKTRLKSNLLDFHCGRCAVVSSSGQMLGGGRGPEIDQQDCVIRMNVAPTAGYE
ADVGNRTSLRVVSHTSVPLLVRQQGYFFRREADTKYVIWGPEKNMRQDGKGKTFNALVTLAKKYPHSHVHTITREKVQYCDGVFQNETGKNRMKSG
AFLSTGFFTMILALEVCDSILVYGMIDGSYCSNANHSFVPYHYYEPLHLDECRMYRVHEHAKRGGHRFITEKLIYSRWASQGKLRFVYPPWTSQEH
QPP

>Mmus_ST6GNc3

MACILKRKPVLVVSFIALCILLLAMRLVNDATFPLLLNCFGQPKTKWIPLPYTFRQPLRTHYGYINVRTQEPLQLNCNHCAIVSNSGQMVGQKVGE
EIDHASCIWRMNNAPTKGFEEDVGYMTMVRVVSHTSVPLLLKNPDYFFKEASRTIYVIWGPFRNMRKDGNGIVYNMLKKTVDAYPDAQIYVTTEQQ
MTHCDRVFKDETGKDRVQSGSYLSTGWFTFILAMDACYSIHVYGMINETYCKTEGYRKVPYHYYEQGKDECNEYLLHEHAPYGGHRFITEKKVFAK
WAKKHRIVFTHPNWTLS

>Hsap_ST6GNc6

MSSNKEQRSAVFVILFALITILILYSSNSANEVFHYGSLRGRSRRPVNLKKWSITDGYVPILGNKTLPSRCHQCVIVSSSSHLLGTKLGPEIERAE
CTIRMNDAPTTGYSADVGNKTTYRVVAHSSVFRVLRRPQEFVNRTPETVFIFWGPPSKMQKPQGSLVRVIQRAGLVFPNMEAYAVSPGRMRQFDDL
FRGETGKDREKSHSWLSTGWFTMVIAVELCDHVHVYGMVPPNYCSQRPRLQRMPYHYYEPKGPDECVTYIQNEHSRKGNHHRFITEKRVFSSWAQL
YGITFSHPSWT

>Hsap_ST6GNc5

MKTLMRHGLAVCLALTTMCTSLLLVYSSLGGQKERPPQQQQQQQQQQQQASATGSSQPAAESSTQQRPGVPAGPRPLDGYLGVADHKPLKMHCRDC
ALVTSSGHLLHSRQGSQIDQTECVIRMNDAPTRGYGRDVGNRTSLRVIAHSSIQRILRNRHDLLNVSQGTVFIFWGPSSYMRRDGKGQVYNNLHLL

SQVLPRLKAFMITRHKMLQFDELFKQETGKDRKISNTWLSTGWFTMTIALELCDRINVYGMVPPDFCRDPNHPSVPYHYYEPFGPDECTMYLSHER
GRKGSHHRFITEKRVFKNWARTFNIHFFQPDWKPESLAINHPENKPVF

>Hsap_ST6GNc4

MKAPGRLVLIILCSVVFSAVYILLCCWAGLPLCLATCLDHHFPTGSRPTVPGPLHFSGYSSVPDGKPLVREPCRSCAVVSSSGQMLGSGLGAEIDS
AECVFRMNQAPTVGFEADVGQRTTLRVVSHTSVPLLLRNYSHYFQKARDTLYMVWGQGRHMDRVLGGRTYRTLLQLTRMYPGLQVYTFTERMMAYC
DQIFQDETGKNRRQSGSFLSTGWFTMILALELCEEIVVYGMVSDSYCREKSHPSVPYHYFEKGRLDECQMYLAHEQAPRSAHRFITEKAVFSRWAK
KRPIVFAHPSWRTE

>Hsap_ST6GNc3

MACILKRKSVIAVSFIAAFLFLLVVRLVNEVNFPLLLNCFGQPGTKWIPFSYTYRRPLRTHYGYINVKTQEPLQLDCDLCAIVSNSGQMVGQKVGN
EIDRSSCIWRMNNAPTKGYEEDVGRMTMIRVVSHTSVPLLLKNPDYFFKEANTTIYVIWGPFRNMRKDGNGIVYNMLKKTVGIYPNAQIYVTTEKR
MSYCDGVFKKETGKDRVQSGSYLSTGWFTFLLAMDACYGIHVYGMINDTYCKTEGYRKVPYHYYEQGRDECDEYFLHEHAPYGGHRFITEKKVFAK
WAKKHRIIFTHPNWTLS

>Drer_ST6GNc6

MSFRSAEGVSIMQALAMTVAEIPVFLYSTFGQQWQQGQRMVIYGAVFLITTLLILYSSSSSADLSRSFKTSNFRQIHHTLKTTNLKKWAGKDGYLP
VYGNKSLNLHCHHCALVTSSSHVLGSQAGEEIDRTQCVFRMNNAPTSGHQSDVGNRTTVRVVAHSSVFSVVRKPAEFLNRSENPIIIFWGPSSKIS
REAKGTLYHLIQRVSMTYRNLSFFFISPSKMQKFDALFQKETGRDRKKSQSWLSTGWFTMVIAIEMCDNIKVYGMVPPNYCGRRPQPKRMPYHYYK
PRGPDECVTYLQNERGLRGSHHRFITEKQVFARWAKVYNISFTSPTW

>Trub_ST6GNc5

MKMRVCQGVGGIIIVTIVTSFMVAYNSSGDTSSSLTSHRVAEPVPSKKQPKGGFTLEGYTRVVDHQPLKMHCGTCALVTSSGRLIRSKRGQEIDRS
DCVIRMNDAPTVGHQRDVGQRTSLRVIAHSSLQRVLQSRQELLNSSQDVAYVFWGPSSCMRRDGKGHVYNSLRLMKQLMPALKLYIISRPKMLKFD
ELFKKETGIDRKSSNSWLSTGWFTMAIALELCDRVDVFGMVPPEFCRSSSHPSVPYHYYEPNGPDECSMYLSHERSRQGSHHRFITEKAVFANWAR
TLNVHFHQPDWKPMAVVSGPNSSRT

>Drer_ST6GNc5-A

MKTRTRHGIILIGVVTVLTSLLFVYNSTSADTPGDQLHSDGHQIDVASRQTFSLEGYISIIDHKPLKTHCRSCALVTSSGHMTGSGRGAEIDETEC
VIRMNDAPTRRYQKDVGQHTNLRVIAHSSMQRVLRNRYELLNSSQDTFFIFWGPGNYMRRDGKGLVYNNLRLMKQLMPKLQVYIISRPKMLQFDEL
FKNETGKDRKRSNSWLSTGWFTMAIAIEMCDRINVYGMVPPEFCKPHTKPSVPYHYYEPSGTDECTMYVTHEQGRHGSHHRFITEKRVFAKWARMF
NIHFYQPDWGPPPVINNNNNTNS

>Drer_ST6GNc3

MAWIWKKKSVIVTSLIVVVSLFLVVINCSEKPYFLLQPVFGQSFSRNWMFSRPPHKASKPHHGYLSVPNQEPLKLHCEVCSVVSSSGQILGREAGA
DIDQSSCIWRMNNAPTRGFERDVGHRTDLRVVSHTSVPLLIQKPQHFFGQGNETVYVVWGPLRNMRQDGKGIVYNMLRQAVENYPQARIYVTTEER
MNYCDTVFKKETGKDRIQSGSYLSTGWFTLILAMDMCKEIRVYGMINDTYCKSEGYKKVPYHYYEAGSRDECAEYLLHESAPYGGHRFITEKAVFA
KWAKTHPIKFFSPEWQLS

>Dsim_ST6GAL_02400

MRQVTRASSGQSQALLSCLIIAVCAALIIQAGQIQGAKAQPGREPGEVNSPGNGNGTTGHRVPRSVFHVRWNPNEKFIVESRENPAINSSKLAPHP
RLKVSKNTKLTLSPKLYLCHDKHSELCHNKTQQFRQRIVRAFEKAMVESVNESQANHYNVDYKPVFGDSFEEQYYPSTCLVMEAGVRVLRRKDAPF
NKLPFGRLFPRQKLFRNVKDIKTCAIVSSAGSLAGSKLGRFIDTHDIVMRFNHAPTQGHEVDVGSKTTIRVVNSQVVTKPEFDFTRAPIFRNVTIA
AWDPGKYNGTLEDWLTSADYDLFTNYELYRRRYPKSRAFLIDPHSVWRLWQSLQMFAGNRPISRNPPSSGFIGLALLLPHCPQVDFVEYVPSTRLN
GRCHYYSKEMNSACTFGSWHPLAAEKLMALDMNMAEDDDMSVFQFGILRIRRPDKLLCGFNFFGY

>Hsap_ST6Gal2

MKPHLKQWRQRMLFGIFAWGLLFLLIFIYFTDSNPAEPVPSSLSFLETRRLLPVQGKQRAIMGAAHEPSPPGGLDARQALPRAHPAGSFHAGPGDL
QKWAQSQDGFEHKEFFSSQVGRKSQSAFYPEDDDYFFAAGQPGWHSHTQGTLGFPSPGEPGPREGAFPAAQVQRRRVKKRHRRQRRSHVLEEGDDG
DRLYSSMSRAFLYRLWKGNVSSKMLNPRLQKAMKDYLTANKHGVRFRGKREAGLSRAQLLCQLRSRARVRTLDGTEAPFSALGWRRLVPAVPLSQL
HPRGLRSCAVVMSAGAILNSSLGEEIDSHDAVLRFNSAPTRGYEKDVGNKTTIRIINSQILTNPSHHFIDSSLYKDVILVAWDPAPYSANLNLWYK
KPDYNLFTPYIQHRQRNPNQPFYILHPKFIWQLWDIIQENTKEKIQPNPPSSGFIGILIMMSMCREVHVYEYIPSVRQTELCHYHELYYDAACTLG
AYHPLLYEKLLVQRLNMGTQGDLHRKGKVVLPGFQAVHCPAPSPVIPHS

>Drer_ST6Gal2

MKSSLKQWRRLALGLILVWALLFLALLSYFMESRVDDPHAAAALSYTDTRRLTSLQGNPRTIMATHLGLATSSAPSTSSNTQQEQSQEENPSADPQ
PSPLSQEAYPYPDPQSLAAWSAFGTQDVGSRSTGVSRNRERQEYNQDSPQEDEDEEEEVIGGEEEDEEGGDEGRGRTTKRVARHGSSDPHEYYVPR
YKSIVHGLWKGSLSMGMLSPRLQRAMKDYLNNNKHGVAYRGHRKAKQSRQQVLCELKKREKIRTLDGAEMPFSKLGWQKIVPALPLSQIHRPGLKT
CAVVTSAGAMLHSGLGKEIDSHDAVLRFNTAPTVGYERDVGNKTTIRIINSQILANPMHRFNRSSLYKNVTLVAWDPAPYTLNLHQWYSNPDYNLF
TPYMEYRMRFPSQPFYILHPKYIWQLWDVIQANNLENIQPNPPSSGFIGILLMMSLCEEVHVYEYIPSLRQTDLCHYHERYYDAACTLGAYHPLLY
EKMLIQRMNIGSEDELKRKGKVTLPGFNKVHCEP

>Cmil_ST6Gal2

MKANTKQWKHLVLIGILAWALVVLLLFIYFTDLKTDERPARSLRYTETRSLFPIQGKQRVIVGNLQNSKLSPVHGENNLYFEDHDDDVSLLGLDSL
NGQNLKWTREEDNGEFEVTKRVKDRKIAAHQQPPNWVMEQVVNITKDANTYDEIVVQDAKPHKRAMKRQEQSSYSVDPFLDNLDSLDLEDLQFSKS
KAVLIKLWKGNFTYNELNPRLQRAMREYIYENKHGVQFKGRRGTKKLSGDELLCELKKRVRLRTLDGNETPFSLLGWKKYVPKIPLSKIKLKLRVF
RKCAVVASAGAILNSSLGDEIDSHDAVMRFNAAPTKLYEQDVGSKTTIRILNSQILANSKHNFINNALYKNIILVVWDPAPYSINLIKWYKKPDYN
LFTPYLRYRRRNPAQPFYILHPRFLWQLWDIIQENTQEKIQPNPPSSGFIGIVIMMALCDSINIYEYIPSIRQTDLCHYHERYYDSACTLGAYHPL
LYEKLLVQRMNKGTEADLYSKGRVSLPGFHSIKCAGEK

>Cliv_ST6GAL1

MVHINALKKLMCVLVVILVALTVCLWRETRRSYYVPFKTENDDLQVHRTSEKWTSLKSQGLFHEAVSELGHIPQTSFGNHNKVKGSTSGTAEKFKK
AADSVKVWDKDSSSRNLIPRLQKVRINYLSMNKYNVTYNGKRNTAKLSPEKLLCQLRDRVNVTMIEGSDGPFDTSEWQQYIPGKSLNETVGRLGRC

AVVSSAGSLKSSHLGQEIDSHDAVLRFNGAPIKGFQEDVGQKTTIRLVNSQLVTVEEQQFLREELYNTGILIVWDPAPYHAEIHEWYRKPDYDFFE
SYKLYRSIHPEQPFYILNPKMQWQLWDILQENSLEPIQPNPPSSGMLGIVIMMTLCDEVHVYEFLPSKRQTDICHYYQKFHDRACTMGAYHPLLFE
KNLVKHINQGTDEDIYMHGKVTLPGFRNVHC

>Hsap_ST6Gal1

MIHTNLKKKFSCCVLVFLLFAVICVWKEKKKGSYYDSFKLQTKEFQVLKSLGKLAMGSDSQSVSSSSTQDPHRGRQTLGSLRGLAKAKPEASFQVW
NKDSSSKNLIPRLQKIWKNYLSMNKYKVSYKGPGPGIKFSAEALRCHLRDHVNVSMVEVTDFPFNTSEWEGYLPKESIRTKAGPWGRCAVVSSAGS
LKSSQLGREIDDHDAVLRFNGAPTANFQQDVGTKTTIRLMNSQLVTTEKRFLKDSLYNEGILIVWDPSVYHSDIPKWYQNPDYNFFNNYKTYRKLH
PNQPFYILKPQMPWELWDILQEISPEEIQPNPPSSGMLGIIIMMTLCDQVDIYEFLPSKRKTDVCYYYQKFFDSACTMGAYHPLLYEKNLVKHLNQ
GTDEDIYLLGKATLPGFRTIHC

>Bmor_ST6GAL_02414

MHNTYDVKYKSGPKNALSKHDVMCALKRIKVNTVTKRDEPFARYDFQIPEGPLQDGTFYNTCAVVSSAGALLGSRLGDFIDSHDMVLRFNNAPTDN
YTDDVGSKTTFRILNSQVVSKPEYKFLEDPLYKGVSIIIWDPANYSSTLEEWYNHPDFPLFPVYKKFLEETPNADAHLLNPQVLWSLWAVLQNVSP
YRLRRNPPSSGFIGLWFSLNRCRRVRVFEYVPSTRASRRCHYYAHRDDVACTFGAWHPLAQEKALAETLRDNSDIDTFQRGFIDITGLATIQC

>Bflo_ST6GAL_02426

MTTKWVFVLITMVTVTAVFCLYVTRHIFYVETVGTKARAKWIPAYAKHREIHSDDFRDNRTTATTSTKTTTTDERTRNVPSFSSEARRLLCSLQSA
ARFRTLPADESPWKQLGYGDIEARPGLLQLYAGGFRTCAAVMSSGAMLGSRLGKDILDSHDAVLRYNSAPTKRYQSDVGSKTTLRLMNSQVVVKQS
KYHWFTDPRYRNVTMVLWDPMSYGGTMAEQWYKKPDFNLFPAYEKRRRESPWEHVYIMDPRYLWELWEVLHRNTREKIVRNVPSSGFMGIHLLLSL
CDTVDVYEYVPSKRETQRCHYYGEEMDIACTYGAYHPLRAEKELIKKLNIGPEEDVRNKGKITLPGFGSIQC

>Aaeg_ST6GAL_02407

MLRDVSLGKCCMICFAVFCLVFAAQLEADRRSVFQFKHNASERQLRMGAEKPSIVNDERDAPRTRPQLRSSKNSNVAFYPTKYACGDPSMTECVNK
TQVFKARVLSEFRRILKESFEENNYYRVHYNKPIFDGESELCRLKRADVRTLSWRDPPFNWNEIGSYFPLNPLFKERNASCAVIASAGSLKGSRLG
DFIDDHDVVMRFNHAPTKGFEVDVGSKTTVRVVNSQVVTKKEFKLLTAKHFRHVSIAAWDPGKFDQSLEEWIKSPDFNLFENFKKYREKYPGSNFH
LVDPRSIWRAWTALQDKTHTKIMRNPPTSGFIGLGLLIPACRYIDMIEYIPSSRMNGLCHYYDSEINSACTFGSWHPLAAEKLYVLQMNTADEFTT
FQRGVVRISLDSNSGC

>Afor_ST6GNc_02265

KNENKTPTIIKIFSNSHPANSTYGEKFYREIENRTRLRKPLWSLEEKEYVRSSQFIHYMTCPTTVRKKMMLDDFSSRKFIPEMPILLWNEHFSKAE
YQRLSTFKGINGWSDIDAQDVSDSLQLLSAPNNRYLFDDRMVNGVIPKKPGCTRCAIIGNGGVMNGSNKGEEIDAHDYVFRVNVALTKGFEKDVGS
KTSFYCFTMVTLSNSLRGGGRYGFKAPPYQKGIKYVFFADNEWTYNYLNAVLRNKPPPRSKDKYKRGPPNFVEQLKAEDVKVVHPDFERYLKWSWV
NSTAQHKSVHRPTTGAIMLLLALHTCDEVNVYGFGGSYTTFSEHYYDKSFVRHVFYANHDNNAENALWKRLNDLGIINMYIRN

>Lpol_ST6GAL_02415

MGRIIAISIWMFIVMMIFGVGGYVYVLWIQYWRFAKNKHFSHAAAIRRTFLENNLTSLSLKEVAQSLNSPSQYHLEVTANKTEKNSFAEEHKSLVD
NLIKKINTYKHQLIVQFRKWQLQGASVISDQSQSHNDYKVHFKGKRSFGYKSETDLVCSAKHDVPVSMLSADDTFFKEIGLAPLFPNKNFNQVLGL
YNSCAIVSSSGSMYKSGLGKEIDTHDVVLRFNDAPTKGYEKDVGNKTTVRILNSQVVSKPEFDFSHSPLYENMLLIVWDPPGYEDDIKKWYQHPDF
DFFPFYWERRQRLPEENFFVVHPTVIWKAWNFIQENTAVPIKKNPPSSGFLGLLLLLQHCNTVDIYEYIPSMRLTKRCHYYDVHENLGCTLGDWHP
LASEKLLSLAMNAASDLQVFVNGKISIKGDVKC

>Phumcor_ST6GAL_02412

MIKTSDMKTVAFLVWIFFNLAFMSMGCYIYLIVSTYWCGQTKETIQPILDKKLLKFDKMNHVVFYETKNESSNGVNVIASNNKYVAVLKKYSRFRR
RTTRPCNNNVNCSSESHNDRLEKFKNKLFIQLRRVLHEESNAFKMDNPYFVHYNGPRERYQMLSSNDIKCRLKKSELRLLTKKDLSFKTLNNLRND
KNEKEEEFLEGMQYKTCAIVSSAGSLLNSKLGHLIDQHEIVVRFNHAPTVTYEEDVGSKTTVRLVNSQVVSKKEFNFTTSSLYKNVTLVVWDPSNY
SATLQEWYEKPDFNFFERSEIHMESLGFFTKLTSMRIRKNPPSSGFLGLALLMPYCDTIDFFEFVPSTRLTKKCHYFEDFEDDSCTFGVWHPLSAE
KILSLAINEMNDSVVFNTGFIRIKGYKDLKC

>Pmar_ST6GAL_02423

GERVWARPRPQQPRTPATPSETSDNPAAPTAVRAAARAAARRVAARGPRGGRVGRRARARGQLSPRLQRAREKYIEQNKHGVRYAGAAGRPPSTGP
LSAAGRRDLLCLVAERVTALRSTLTLGGSEPPFSAMGAWREALDAGGLERAVSGGRSLERCAVVSSAGAMLGSGLGREIDAHDAVLRFNAAPTEHF
EKDVGTKTTIRLVNSQILARPQNNFSRSTLYRDVTLLVWDPAPYSLNLTQWFHDPDYNFFPAFLRRRQRHPTQPAFVLHPAYLWRIWELLQDSASE
HIQPNPPSSGFLGVVLMMWLCDEVNVYEFLPSRRRTDLCHYHEDYTDRACTEGAYHPLLYEKNFVKRLSTSSDHDLFWAGRATLPGLRRAMESCPP
RARQ

>Skow_ST6GAL_02427

MARKRKDDVAPAMYRHFLSIVLIMFALVAIYIMLLYSEQRHCIKLDKGMRQLVVYARRILDQYSQEEWVKELQHGDIDLDDTQVKSAIDRLVLSFR
KYTARKEGSETVAESRSSLTEQNEFDLSVFLKEYDKTFNNYYLMTSHNPYGVNFSGTIYNESRDSQDLLCDLKRRAKYDVLKRDTTPFKEIGISKY
FPEISLYEKFKGFESCAIVASAAYFKNATLGKEIDSHDAVLRFNDAPTEGFEENVGRKTTIRLMNTLIFEEERFGFETKDIFRNITLLVWKSGPYN
GNLYRFYSLYKDSKPFFDKYIQWREAHPWESFYIMDPTPIWRTWDIIQENTPNITIRKTLPSSGIMGISLMQRMCDTVNVYGYLIPNGDPPLCHYY
DGMCSKATWHPVEYEKKLVRHINFGSTDDILKHGRMTVPGFRTFHCD

>Skow_ST6GAL_02428

MGMTKKVTIRCLGMCIAAIALVVVTRQLYDKPFAASSGNIQQILRIQSRTQSDGGFLSNLFNPLRVDFTGERHFQNLNSVNTVCKFKLSANASVLD
KSTRPYQDLGLSKYFPKKNIFNAHEKRYNTCAIVSSSSFMTDSGLGEEIDSHEAVMRFNNAPTVGFERDVGNKTTMRVINLDIMLLPDFYSNYIFN
NVSLVAWYQAPYTFTPDKWYEHNGAAFYEKYIERRISRPEEKMYVINPAWHWNIWDILQEYSEENINKHVPSSGFTGIILMQQICDQVNVYGFVTP
KTRLLHYYSNVLINAPTGYLWHPMVQEKRIAKIMNRGPEEEITNHGKITLPGFNQMHCL

>Skow_ST6GAL_02429

MMGYDTTPFKEMGISKYFPGRNIYEKYNYSRCAVVLSSFYMNETGFGDEIDSHEAVLRLNDAPTVGYEKSVGRKTTIRLLNSKCFNDSTFINDIEA
MFVNTTLVTWKSGPYNGNLYKWYQRESVKELLAKYIRWRMVQPSQDFYVINPTSLWDIWDDIMYFNKGKPIKKIVPSSGFTGLLMMLGLCDKVDVY
GFGINDVSSCHYYDKPGCNSASKRWHPLQEKAIMAAMHDGPISDLDKHVVTVNGFKHINCNGT

>Skow_ST6GAL_02430

MVIVGISSFAYNSSRKSKSWIRLALLVTVVHLVLLVFIAQTSSNKTLLVLGNYYEWFNIGDNSSQSFGSFGTENMQTPSRTSYNSSTRSVSSRTEC
YNRVLYNSSEMVCTLRTAALKILDRQTSPFNKLGLSRYLPNGSAFLEMKTYKWCAVIGSSYFLLGSKLGKEIDSHDAVLRFNDALVTGFERDVGSK
TTIRLINSQMLVKKKKNASLLKALNVNSTLFVWKAGGRDENLMKWYTVSKDMFNNYIKWCIHRPEQCPYVINPSVLWKCWDIIQRYTKERMKNIVP
TTGFTGIHLMHHICESIDVYGYVTPTNTNCHYYDVHKCPRLAWHPVEAEKNFLKTLHVGNENDLSVKGKLSLYGFHHDCVTC

>Isca_ST6GAL_02411

GRICATVCSLSRLFPTPRDTEPFKRMGYSRYFPTTNLYESCYNSCALVSSAGSLLGSRLGNEIGKYAHQAVLRFNDAPTQGFEQDVGSRTTVRLLN
SQLLSRPEFDFFNSPMFRNLTLIAWDPSRQGQSLEQWVKKPDFDLFPGYWLRREVLPEEPFYLLHPDSVWEAWHFLDRENPGPVVRNPPSSGFLGL
LLLLGLCRRLDAYELVPSMRLTKRCHYYEVHEDLGCTLGDWHPLAAEKLLALHMAAPEVLDQVFAQGRLPLGGGSSAAP

>Apis_ST6GAL_02408

MKESSNYGSVKNENPYNVPYRPQAPNNVKSDWTCDMASSVEKFRTLEKNDIDHMLTKNIPDVPLFDDNEVFGTCAIISNAATLRNSNLGYFIDQHD
LVLRFNNAPTKGYEKDVGSKTTIRILNSQVVTKPQFQFVSSPLYKRLKLLMWDPSNYTSSINEWITNPEHNFIDNYISFRKSNPRSNFHIVHPQYL
WRLWDYIQDHTTAHIRRNPPSSGFLGLAMLLPRCTVVNMFEFIPSERMTHRCHYYHEKVDVTCTFGIWHPLAAEKLLMLTANTMPDQTVFHTGFLS
IPGYKSPICTSL

>Tcas_ST6GAL_02413

MRALVVSIWVFINLVFFGMCGYMYLLWSQYWMFMERQTGTTTSTYDQQIYYYNRGFLPNDNKTHNETKLRIKHSLKDNVTVTLIKNSKPRFPNLQG
REFELDTKKYICLKNDSFKNCSSKSLEFKEKILKELRRVLEDEGNVLKIGAENPYNVQYEGTKGNFYDKTPKEIMCELEKIQVKTLKRGDVAGGPH
NLGDFLPKRGLFENRNFNSCAIVASSGALKDSNLGKKIDAHDLVLRFNHAPTKGFEPDVGRKTTIRVLNSQVVTRKEFNFLESDIYKNVTIVAWDP
SNYKASLDDWLEKPEFNLFPTYVEYKKRNDKARFFLINPQSLWELWDFLQDNSPSRLRRNPLSSGFLGLGILLPLCNFIDVFEYVPSTRVTKRCHY
YDPEDNPACTFGVWHPLAAEKLLTYYINTIDDRTVFQDGYVRILGFKNFKC

>Acar_ST6Gal1

MVHVSSFRKCIYVLLAFILVLTICLWKETRKGVIFSLRLEGRSDHIPWKMWRWGTSKEEPVQEVAVIATGKVPLLSNTEVNLSTKVTRSNLIIKVW
NKDSTSQSLHPRLQKARQTYLKINKYKVNYSGPKHAHKLRPKELLCQLRNRLDFRMITATDSPFDISEWEPYVPKGNISIKLGRCAVVSSAGSMKS
SHLGEDIDSHDAVLRFNGAPIKGFQADVGEKTTIRLVNSQLITVEEKKFLADPQYNFGTLILWDPAPYHSSIKEWYKKPDYNFYGSFKHYRKKYPK
QPFYILNPHMQWQLWDILQENSPEDIQPNPPSSGMLGKLIMMNFCDEVDVYEYLPSKRQTNICHYYQKIFDQACTMGAYHPLLFEKNIIKHLNIGK
DEDIYNYGKATLPGLRNVQC

>Tgut_ST6Gal1

MVHINVLKKFMCVLVVVLVALTVCLWRETRRNYYDPLKTRNDDLQGHRSLEKWNTVKSQGLFHEAASELGQVSKTWLGTQNKVKGSTSETDEKSKK
AVFGKVWDKDSSSRNLIPRLQKVRKNYLAMNKYNVTYNGKRNAKLSPEQLLCQLRDRVNVTMIQRSDGPFGTSEWQQYLPGKSLSEAVGHLGCCAV
VSSAGSLKSSHLGQEIDSHDAVLRFNGAPVRGFQDDVGQKTTIRLVNSQLVTVEEQQFLREPLYNTGILIVWDPAPYHAEIREWYRKPDYNFFESY
KAYRRAHPEQPFYILNPKMQWQLWDILQENSLEHIQPNPPSSGMPGIVLMMTLCDQVDVYEFLPSKRQTDICHYYQKFHDHACTMGAYHPLLFEKN
LVKHMNQGTDEDIYTHGKVTLPGFRKVHC

>Ocar_m41732

SFHANLPRRDLPRFGSCAVVSSASSLLRYEFGPLIDSHDAVIRLNGAPTIGYEKHVGSKTSLRVINARVARNLSLLEPEIRLEFREGKSIFFVRDT
PPSLTRGEKETGYDRLNSGKFVPLDTYRDLREKFGESDLYLMHPLFAFVVGLELLRYGEGKKDIFISSSSGFHAVLMALFLCDSVSSFEVATADDL
SRKKPYYFKIPNENDMNHSYYRGASWHPLNLERRILERLSDSTIGNATSSFH

>Ocar_m18701

SLSKSTLLCCFKRMRQKMPLSLTKLDSVLLLHSNCSRYLTQSLPERKPQMFNSCAVVSSGPSSMLKFKLGKEIDSHDAIFRINMAPTKSFEKYVGS
KTTIRVLNSKILKHPSSYLKLVKTDRSQLNNKSRLLYFVREMTPPNLPKAQNACLVMDRLLTGYMKFQKEAKTVEAHVNHPLFSRFCSLAELMFRL
KAPGRKRGLSFSSGTFAVLSAIWLCEYVTSYEIASNDPLSKNSSYYFDGKSGKAYSAFHPLPAERSVLEKL

>Ocar_m126566

NKSASCLKQGSSKKDLLCCVKMIKKTLPLGLAADDGSFLKQIGFSDYFLRLLPEHDVPRFGSCAVVSSAPSLLKYQMGNEIDSHDAVIRINLAPTK
EFEKHVGKKTTIRFINSGVAAKMNKNLVGLDSSNVTYFVRDLPLSAKPGSNFSQIWDTGVFHPVRRYVKNRENFPSNVIFLNHPLFAVFAGRDFVT
NVLGKKPHYTLSSGSQAFLMALFMCESVTAYELATTDSLSRQFQYYFDKKNVPYSTVHPLDIERKVLATFGS

>Ocar_m311493

SGYPMMLNGYRSIIDPNEPLKMNCTTCSLVSSSGRILGQSKGAEIDGADCVLRMNIAPVKGYEGDVGKRTTARILSQFSVKFARNQLLAAENLRYF
IAWGGNVHLGQKTANYKKMLEKAIQLPNIGFFRSSEEYYWYQDKVFENITGRPRQKSGTWLSTGWFTFDVIKNACKRTKVYGMIPEVFCRDPTAPK
ALYHYWDPNAGDECAYYSRMENMRAGAHRFMTEKAIFARWALQHSITFHSPDWDPKLHTEAH

>Ocar_m63885

MQTSIFIKNRETTDGQQKQLEHLSIETTHRPSQKASPTKRESQRDMKKIPGYIDVTVTSKELSLACQSCSLVGSSGHLLSEKAGEEIDKADCIFRM
NQAPVRGFEKDVGRRTTVRVLNHVCPVLSHKPTQHQLFQLETSDVAVVWAPPKRLPLILTRLKQIAHDFPHMRVYLITAQKMKNLDELFAKETGRD
RVKSGSWLTTGWFTFDLVLRACDQVSVYGLPPANYCSLPNAKEVPYHYFNRVASECKYYKDGHHRFVTEKSIYAKWSLNRNVSFYLPSWDAKNNS

>Ocar_m10101

PFRKRHRRSRPSTRVIFGYTDMNNSSLPLQLTCHSCALVGNSGHLLGSRCGQLINKADCIFRMNEAPVPGFKKDVGNRTDVRIVNFHISPLEGKIQ
AEKLIFQRKKSEIIVVWGPAAKISSSTLISTLEGIVKRYPRIKVYLVSKTKMRDLIQLFTIETGVAVKKTNSWLSTGWIAFDVVLRACDSVAVYGF
PPSDYCKESDGRNISYHYYGKSESECKYYNNHHHHRFADEKAVYARWALTHNITFNAPPWDPRS

>Ocar_m178569

KQIVSSGEVPSEDVFESWRQSKGNLTCAVVGNSDILSGSNKGQLIDSHEVVFRFNNYETKGYEKDVGNKTTFQNLYRRIAKYYKPPSRVIVITQAL
VELTWVENVHKVGIDKKKVLLLHPDFIYLVRTWLKSVDSSYKNIPSSGIYTLLLALYVCDEVSIFGFGVNGSYYGHYYGVRGKVSRVPLRQTSYHM
WDTEKMLRDRLQTVGVIKVYQ

>Cint_ST3Gal_00828

MLINFKLSRVIAMLLVVAIFLTYSWLLLWSTKTALQTNRKNKAGQDEVPVINVIKEDSYVQQKTQNLNKGKRFDLGRVNHSHPREEIQQNNKCGHQ
LDASQTRWFRARFNPEIEPVWTQSALEIDYLVYDWWLSLQSSEAENLDKTFEALYKEGVPRKDPFARLTHDREAGCRSCAVVGNSGNILNSNYGNV
IDGHDFVIRMNKGPTYNYENDVGSKTTHRFMYPTTAASSLPQGVSLVLVPFQPLDIKWLLSALTTGEITRTYQPLVRRVTCDKSKITIISPTFIRY
VHDRWTQHHGRYPSTGLLALIYALHECDEVDVYGFGANRAGNWHHYWEDLPPHVAGAFRKTGVHDSAQENEIIDQLHIHGLLRVHRSEQSS

>D2KQO2CAMJU

MTRTRMENELIVSKNMQNIIIAGNGPSLKNINYKRLPREYDVFRCNQFYFEDKYYLGKKIKAVFFNPGVFLQQYHTAKQLILKNEYEIKNIFCSTF
NLSFIESNDFLHQFYNFFPDAKLGYEVIENLKEFYAYIKYNEIYFNKRITSGVYMCAIAIALGYKTIYLCGIDFYEGDVIYPFEAMSTNIKTIFPG
IKDFKPSNCHSKEYDIEALKLLKSIYKVNIYALCDDSILANHFPLSININNNFTLENKHNNSINDILLTDNTPGVSFYKNQLKADNKIMLDFYNIL
HSKDNLIKFLNKEIAVLKKQTTQRAKTRIQNHLSYKLGQALIINSKSVLGYLSLPFIILSIVISHKQEQKAYKFKVKKNPNLALPPLETYPDYKEA
LKEKECFTYKLGEEFIKASKNWYGGGYIKFYFKDVSRLKREIKEK

>Q7BP25CAMPJU

MSMNINALVCGNGPSLKNIDYKRLPKQFDVFRCNQFYFEDRYFVGKDVKYVFFNPFVFFEQYYTSKKLIQNEEYNIENIVCSTINLEYIDGFQFVD
NFELYFSDAFLGHEIIKKLKDFFAYIKYNEIYNRQRITSGVYMCATAVALGYKSIYISGIDFYQDTNNLYAFDNNKKNLLNKCTGFKNQKFKFINH
SMACDLQALDYLMKRYDVNIYSLNSDEYFKLAPDIGSDFVLSKKPKKYINDILIPDKYAQERYYGKKSRLKENLHYKLIKDLIRLPSDIKHYLKEK
YANKNR

>Q9CLP3Pasteurm

MDKFAEHEIPKAVIVAGNGESLSQIDYRLLPKNYDVFRCNQFYFEERYFLGNKIKAVFFTPGVFLEQYYTLYHLKRNNEYFVDNVILSSFNHPTVD
LEKSQKIQALFIDVINGYEKYLSKLTAFDVYLRYKELYENQRITSGVYMCAVAIAMGYTDIYLTGIDFYQASEENYAFDNKKPNIIRLLPDFRKEK
TLFSYHSKDIDLEALSFLQQHYHVNFYSISPMSPLSKHFPIPTVEDDCETTFVAPLKENYINDILLPPHFVYEKLGTIVSKKSRFHSNLIVRLIRD
LLKLPSALKHYLKEK

>Q17WF9ST38He

MNKKPLIIAGNGPSIKDLDYALFPKDFDVFRCNQFYFEDKYYLGREIKGVFFNAHVFDLQMKITKAIVKNGEYHPDHIYCTHVEPYGYVNGNQQLM
QEYLEKHFVGVRSTYAYLKDLEPFFILHSKYRNFYDQHFTTGIMMLLVAIQLGYKEIYLCGIDFYENGFGHFYENQGGFFEEDSDPMHDKNIDIQA
LELAKKYAKIYALVPNSALVKMIPLSSQKGVLEKVKDRIGLGEFKREKFGQKELERQKELERQKELERQKELERQKELERQKELERQKELERQKEL
ERQKELERQKELERQKELERQKELERQKELERQKELERQKELERQKELELERSLKARLKAVLASKGIRGDNLIIVSLKDTYRLFKGGFALLLDLKA
LKSIIKAFLKR

>A6YD31Lic3B

MNGTICPINQSINQSINQSINQSINQSINQSINQSKSVIIAGNGTSLKSIDYSLLPKDYDVFRCNQFYFEDHYFLGKKIKKVFFNYSVIFEQYYTF
MQLIKNNEYEYADVILSSFLNLGDSELKKIQRLEKLLPQIDLGHSYLKKLRAFDAHLQYHELYENKRITSGVYMCAVATAMGYKDLYLTGIDFYQE
KGNPYAFHHQKENIIKLLPSFSQKKSQNDIHSMEYDLNALYFLQKHYGVNIYCISPESPLCNYFPLSPLNNPFTFIPEEKKNYTQDILIPPKSMYK
KIGIYSKPRIYQNLVFRLIWDILRLPNDIKKALKAKKMRLRK

2 – ST motifs sequences. Selected conserved motifs (sialylmotifs L, S, III and VS) and family motifs were selected for MSA and phylogenetic analysis .

>Spur_ST3Gal_00992

CVVQGNGGIASRTRLGEVIDNFDVVFRLNSAPTINHENDVGRKT--TFRMAYPES--LQDTASMMGM-MGVRLCDEVAVAGFGY--LHYYD--THDIHHE

>Spur_ST3Gal_00993

CIVIGNGGILKGSKLGSHIDQYDAVFRLNVAPIKGYESDVGTKT--TVRAVYPES--NVPTTGAMVIMVAIRVCDEVSVAGFGY--IHYYS--THDIDHE

>Hpul_ST3Gal_00793

CVVQGNGGIASRTRLGEVIDNFDVVFRLNSAPTINHENDVGRKT--TFRMAYPES--LQDTANMMGM-MGVRLCDEVAVAGFGY--LHYYD--THDIHHE

>Skow_ST3Gal_00797

CIIVGSGGILKNKGHGRVIDEYDVVIRMNVSPVKGYEKDVGTKT--TIRITYPEG--NVPTSGTISIMLALRLCDEVDVVGFGY--IHYYE--THDINHE

>Bflo_ST3Gal_00792

CIVVGSSGLVSGENLGSTIDKYDIVMRMNEAPVHGYEKDIGSKT--TFRFLYPES--NIPTIGTISIITAFHFCDMVDIVGFGF--YHYYE--IHDFAKE

>Skow_ST3Gal_00796

CILVASSGVSTGKGLGKLIDKYDVVIRMNNAPVKKYEKDVGSKT--TFRLVYPES--KRPSTGAMAVVMALHYCDHLHITGYGY--IHYYE--AHSWFNE

>Skow_ST3Gal_00795

CVLVANSGILIGSKLGDVIDTYDIVIRMNNAKTVGYADDVGRKT--TFRFIYPES--KRPSTGIVAMVFAFHYCDIVDIAGYG---NHYFS--THCWDNE

>Hsap_ST3Gal3

CIIVGNGGVLANKSLGSRIDDYDIVVRLNSAPVKGFEKDVGSKT--TLRITYPEG--NIPTLGSVAVTMALHGCDEVAVAGFGY--LHYYE--THNIQRE

>Lcha_ST3Gal3

CIVVGNGGVLANKSLGSKIDEYDVIIRLNGAPVKGYEKDVGAKT--TIRITYPEG--NIPTLGTVAITMALHNCDEVAVAGFGY--LHYYE--THNIGRE

>Drer_ST3Gal4

CAVIGNGFALKNSSLGEIINKYDVVIRLNDAPVRGFEEDVGNKT--TLRLFYPES--VHPTTGLLAVFVALNYCDVVHVAGFGY--IHYYG--YHDLNQE

>Hsap_ST3Gal4

CVVVGNGHRLRNSSLGDAINKYDVVIRLNNAPVAGYEGDVGSKT--TMRLFYPES--QKPTTGLLAITLALHLCDLVHIAGFGY--IHYYE--GHNVSQE

>Mdom_ST3Gal4

CVVVGNGHRLKNSSLGNTIDKYDVVIRLNNAPVVGYEGDVGSKT--TMRLFYPES--QKPTTGLLAITLALHLCDLVHIAGFGY--IHYYE--EHNVSHE

>Acar_ST3Gal4

```
CAVVGNGHRLRNSSMGDVINKYDVVIRLNNAPVHGYEHDVGSKT--TMRLFYPES--QKPTTGLVAITLALHFCDMVDIAGFGY--IHYYE--
GHNVSHE

>Lcha_ST3Gal6

CIVVGNGGVLRNKTLGEKIDSYDIIIRLNNGPVIGHEKDVGKRT--TFRLCYPES--VHPTTGIIATALALHLCDETHIVGFKY--LHYYE--
YHNITAE

>Hsap_ST3Gal6

CVVVGNGGVLKNKTLGEKIDSYDVIIRMNNGPVLGHEEEVGRRT--TFRLFYPES--KHPTTGIIAITLAFYICHEVHLAGFKY--LHYYG--
YHNVTAE

>Hsap_ST3Gal5

CVVIGSGGILHGLELGHTLNQFDVVIRLNSAPVEGYSEHVGNKT--TIRMTYPEG--NVPTIGVIAVVLATHLCDEVSLAGFGY--LHYFD--
MHNVTTE

>Drer_ST3Gal5

CVVVGNGGILKGLGLGHLLNRFDIIIRLNSGPLQDFSADVGNRT--TIRMSYPES--NIPTLGLTALNLATYICDEVSLAGFGY--LHYYD--
MHNVQKE

>Drer_ST3Gal7

CVVVGSRGILHSKNLGAHIDHANIIIRVNNAPVFGFESDAGSRT--TIRLIYPEG--MVPTLGITAVVVALQVCDEVSIAGFGY--LHYYG--
VHDVSAE

>Lcha_ST3Gal7

CVVVGSAGILHGSRLGAYIDQHDIIIRKNGICLKGYTVNIQIKT--VVGISEYER--MVPTLGVSAVVMATHLCDEVSLAGFGY--LHYFE--
MHNVDTE

>Ggal_ST3Gal9

CIVVGNGYSVHGQHFGKMIDSHHVIIRLNDAPVKEYKKDVGERT--SIRLFFPES--RYATTGIIALNLALHLCQEVNIAGFGY--IHYYN--
QHNLTAE

>Spur_ST3Gal_00042

CVVVGNGGVMKQSAMGPIIDDFDVVIRLNDAPTVGYEKDVGSKT--TIRMAYPES--NVPTTGSFAISMATRLCDEVSVAGFGY--LHYYD--
THNIDIE

>Bflo_ST3Gal_0823

CIVVGSGGILLGKKLGPQIDDFDIVIRMNNGPVKGYEEDVGHKT--TIRMSYPEG--NVPTIGSLAIIWAINYCDEVTVAGFGY--LHYYK--
THDINKE

>Bflo_ST3Gal_00041

CIVVGSGGILLGKKLGPQIDDFDIVIRMNNGPVKGYEEDVGHKT--TIRMSYPEG--NVPTIGSLAIIWAINYCDEVTVAGFGY--LHYYK--
THDINKE

>Hsap_ST3gal1

CAVVGNSGNLRESSYGPEIDSHDFVLRMNKAPTAGFEADVGTKT--THHLVYPES--RYPSTGILSVIFSMHVCDEVDLYGFGA--HHYWE--
VHDADFE

>Olat_ST3Gal1

CSVVGNSGNLKGSNYGALIDTSDLVIRMNKAPTKGFEKDVGAKT--THHVMYPES--HYPSTGFLTLMFALHICDEVSVFGFGA--HHYWE--
VHDGDYE

>Lcha_ST3Gal2

CAVVGNSGNLHGSGYGKAIDMHDFIMRINQAPTVGFEADVGSRT--THHFMYPES--RYPSTGMLVLFFALHVCDEVDVYGFGA--HHYWE--
VHDADFE

>Hsap_ST3gal2
```

CAVVGNSGNLRGSGYGQDVDGHNFIMRMNQAPTVGFEQDVGSRT--THHFMYPES--RYPSTGMLVLFFALHVCDEVNVYGFGA--HHYWE--
VHDADFE

>Ggal_ST3Gal8

CAVVGNSGRLKGSRHGLQIDAHHWVLRMNRAKTAGFEMDVGART--THHFMYPES--RYPSTGFTALLFALHACQQVSVFGFGA--HHYWE--
VHDADVE

>Lcha_ST3Gal8

CAVVGNSGNLKGSNHGKEIDAHHFVIRMNRARTAGFEPDVGIKT--THHLMYPES--RYPSTGMTALVFALHICDEVSVFGYGA--HHYWE--
VHSGDFE

>Drer_ST3gal8

CAVVGNSGNLLKSKYGALIDSHSTVIRMNKAVTVGYDEDVGYRT--THHFLYPES--RYPSTGIVAIIFALHLCDEVSVFGYGA--HHYWE--
VHNADFE

>Bflo_ST3Gal_00835

CAVVGNSGNLRQSNYGEEIDGYDLIFRMNDAPTKGWEKDVGHRT--THHFMYPES--RYSSTGSLVILFAVHVCDEVDVYGYGA--NHYWT--
VHDSEFE

>Bflo_ST3Gal_00837

CAVVGNSGNLRQSNYGEEIDGYDLIFRMNDAPTKGWEKDVGHRT--THHFMYPES--RYSSTGSLVILFAVHVCDEVDVYGYGA--NHYWT--
VHDSEFE

>Csav_ST3Gal_00830

CAVVGNSGNLINSKYGN-IDSHDFVIRLNKGPTEGFENDVGRKT--THRFMYPAT--RYPSTGLIAIIYALHECDQVDLYGFGA--HHYWE--
VHDSDKE

>Skow_ST3Gal_00834

CAVVGNSGNLRDSRYGQLIDSHDYIMRMNTAKTVGFEKDVGSRT--THHFMYPES--RYPSTGMLVLLFAMHICDQVNVFGFGA--DHYYQ--
VHNAEFE

>Spur_ST3Gal_00990

CAVVGNSGNLRNSGYGTAIDKHDVVVRINQAKVKGFEKDVGQKE--THRLMYPES--RYPSSGTLAVFFALQFCDEVSVYGMGA--DHYWE--
VHDSVHE

>Ocar_ST3Gal_829

CAVVGTAGRLKGARQGKLIDSFDIVIRMNRSPVKGYEVDVGSKT--SYHLVYPES--RWASAGALSVVWALHICNEVDVFGFGA--DHYYE--
VHDANIE

>ocar_m10828

CAVVGTAGRLKGARQGKLIDSFDIVIRMNRSPVKGYEVDVGSKT--SYHLVYPES--RWASAGALSVVWALHICNEVDVFGFGA--DHYYE--
VHDANIE

>Bathy_4485_02g00010

CAVVGNGGILKSAEFGQAIDAHDVVFRQNQAPTATYELFVGEKT--TFRVLNKKW--NTPSSGIISTVLAMSLCDEVNLYGFGV--DRNVG--
VHSMELE

>Hsap_ST6GNc1

CAVVGNGGILNNSHMGQEIDSHDYVFRLSGALIKGYEQDVGTRT--SFYGFTAFS--YRPTTGALLLLTALQLCDQVSAYGFIT--DHYYD--
NHDFKLE

>Drer_ST6GNc1-A

CAVVGAGGILNNSKMGREIDSHDYVFRVNGAVTKGYEEDVGNRT--SVYVHTAFS--YRPTNGAFALFLAIHTCDMVNAYGFIT--NYYYE--
NHDYGLE

>Hsap_ST6GNc2

CAVVGNGGILNGSRQGPNIDAHDYVFRLNGAVIKGFERDVGTKT--SFYGFTVNT--YMPSTGALMLLTALHTCDQVSAYGFIT--DHYFE--NHDLSLE

>Lcha_ST6GNc2

CAVVGNGGILNDSQMGREINQHDYVFRANGAVVKGFEKDIGNRT--SFYIFSTNT--YRPSTGAVMLLAAIHTCDEVSAYGFMT--DHYFD--NHDYRLE

>Drer_ST6GNc2-A

CAVVGNGGILNGSRKGTEIDAHDYVFRVNGAALNGFEKDVGSRT--SFYTFSTNT--YRPSTGAVMLLAAIHTCDQVDAYGFMT--DHYYD--NHDMRME

>Bflo_ST6GNc_02259-A

CAVVGNGGILRGSGKGKEIDGHDFVWRVNSAIIEGYEEDVGKRT--SFYFHDINT--YRPTTGGSMLLTALHTCDVTDVYGFIT--DHYYE--NHDFQME

>Bflo_ST6GNc_02262-B

CAVVGNGGILRGSGKGEEIDAHDFIFRVNAAIVKGFEADVGSRT--SFYFHTATT--YRPSTGAVALLTAVHVCDVTDAYGFIT--NHYYE--NHDFNLE

>Skow_ST6GNc_02258

CAVVGGGGILNGSRKGEEIDSHDYVFRVNIAATKGYEVDVGKKT--SFYSYTITT--NRPSTGALMLIAAIHTCDEVSAYGFGV--THYYD--NHDFKQE

>Skow_ST6GNc_02269-B

CAVVGNGGVLKNSKLGEEINSHDYVFRVNVAVTKGFESDVGDKT--SHYVFTMVT--YRPQTGAFMLLVALHTCDEVDVYGVGA--DHYYD--NHDYTTE

>Lvarvar_ST6GNc_02261

CAIIGNGGILKGSGKGAEIDAHDYVFRVNAAVTKGFENDVGKKT--SFYCFTMVT--HRPTTGAIMLLAALHTCDQVSIYGFAG--EHYYD--NHDNNAE

>Spur_ST6GNc_02260

CAVIGNGGILNGSGKGAEIDAHDYVFRVNAAITKGFEKDVGKRT--SFYCFCMHS--HRPTTGAIMLLAALHTCDQVSIYGFAG--EYYYD--NHDYNSE

>Ajap_ST6GNc_02264

CAVIGNGGILNGSRKGQEIDAHDYVFRVNTALTTGFEEDVGRRT--SFYCFTMIT--HRPTTGAIMLLAALHTCDQVDIYGFGG--EHYYD--NHDNIAE

>Mgla_ST6GNc_02268

CAIIGNGGVMNGSNKGEEIDAHDYVFRVNVALTKGYEKDVGSKT--SFYCFTMIT--HRPTTGAIMLLLALHTCDEVNVYGFGG--EHYYD--NHDNNAE

>dre_ST8s2_AY55462

CAIVGNSGILLNSSCGREIDSHDFVIRCNLAPVEEYAADVGLRT--SLVTMNPSV--KRPTTGLLMYTMATRFCDEIHLYGFWP--YHYYD--PHTMPLE

>dre_ST8s3_AJ715543

CAVVGNSGILTGSRCGPEIDKYDFVFRCNFAPTEVFRRDVGRRT--NLTTFNPSI--KRLSTGILMFTLASSLCEQVHLYGFGW--YHYYD--SHQLPTE

>Dre_ST8s4_AJ715545

CAVVGNSGILLKSGCGKEIDNHSFVIRCNLAPLEGFADDVGLRS--DFTTMNPSV--KRPTTGLLMYTMATRFCDEIYLYGFWP--YHYFD--PHRMPLE

>dre_ST8s5_AJ715546

CAVIGNGGIIKNSKCGREIDASDFVFRCNIPPVDLYSQDVGSKT--DLVTINPSI--KRLSSGLMLVTAAMELCEEVHLYGFWA--HHYYD--FHAMPYE

>Hsa_ST8s2_7U33551

CAIVGNSGVLLNSGCGQEIDAHSFVIRCNLAPVQEYARDVGLKT--DLVTMNPSV--KRPTTGLLMYTLATRFCKQIYLYGFWP--YHYYD--PHTMPLE

>Hsa_ST8s3_AF4668

CAVVGNSGILTFIQCGREIDKSDFVFRCNFAPSEAFQRDVGRKT--NLTTFNPSI--KRLSTGILMYTLASAICEEIHLYGFGF--YHYYD--SHQLPAE

>Hsa_ST8s4_L4168

CAVVGNSGILLDSECGKEIDSHNFVIRCNLAPVVEFAADVGTKS--DFITMNPSV--KRPSTGLLMYTLATRFCDEIHLYGFWP--YHYYD--PHRMPLE

>Hsa_ST8s5_U91641

CAVVGNGGILKNSRCGREINSADFVFRCNLPPIEKYTMDVGVKT--DVVTVNPSI--KRISTGLILVTAALELCEEVHLFGFWA--HHYYD--GHAMPSE

>Dre_ST8s6_AJ715551

CAVVGNGSVLANSSCGEEINSAQFVIKCNLPPLDRYEKDVGNKT--NLVTANPSI--VRPSTGLIMASLALEICTNVHLYGFGK--NHYYD--VHSMPSE

>Hsa_ST8s6_AJ621583

CAVVGNGGILNKSLCGTEIDKSDFVFRCNLPPTTGDVKDVGSKT--NLVTINPSI--YRLSTGLMITSVAVELCKNVKLYGFWP--HHYYD--FHQMPKE

>dre_ST8s1_AJ715535

CSVVGNGGVLKHSGCGNEIDRADFIMRCNLPPLKDYTDDVGTKT--HLVSANPSI--KRLSTGLFLVSLALGLCEEVTAYGFWP--HHYYD--FHAMPEE

>Hsa_ST8s1_D2636

CAVVGNGGILKKSGCGRQIDEANFVMRCNLPPLSEYTKDVGSKS--QLVTANPSI--KRLSTGLFLVSAALGLCEEVAIYGFWP--HHYYD--FHAMPEE

>Bathy2g01080

CAVVGNAGTLLKSKYGEAIDKHDVVMRFNVMTLAQLAANVGTRT--TFRMVNHLR--KQGTSGFHGILLLAGMCDHLSLYGFSA--DQYGG--WHDWEGE

>Athal_NP17235

CAVVGNSGTLLNSQYGDLIDKHEIVIRLNNAKTERFEKKVGSKT--NISFINSNI--FHYSSGMQAVMLAVGICEKVSVFGFGK--HHYHT--LHDYEAE

>Oryza_AM48825

CAVVGNSGVLLGSGRGPQIDAHDLVIRLNNARVAGFAADVGVKT--SLSFVNSNI--FHYSSGMQAVVMALGVCDEVSLFGFGK--HHYHT--LHDYEAE

>Physco_12207

CAVVGNNGILLNSTFGKAIDAHKVVVVRLNNARIKGFEKHLGGKT--TIAFMNSNI--FHYSSGMQAVMLALGICEEVDLYGFEK--HHYHT--IHDYEAE

>Physco_4887

CAVVGNSNDLLQDLFGAEIDEFDAVIRMNGAPVENYTHYVGEKT--TFRILNRGS--SAKGTGVKAIEFALSVCEMVDIYGFTV--TRYFS--AYYQTME

>Physco_10699

CAVVGNSGDLLLAKFGSEIDAHEVVLRDNEAPVNKTYDKHVGRK--TFRLIGEGV--GAKGTGIKSIELAVSMCDVVDIYGFTV--TRYFS--AYYQLLE

>Physco_5121

CAVVGNSGILLNSTFGKMIDAHEEIVRLNNARIKGFEKYVGGKT--TIVFMNNNI--WPWVQGFHYSSGMQAVMLALGICEEVD--HHYMA--IHDYEAE

>Marchan_33168

CAVVGNSGILLNSSYGALIDSHEMVIRLNNAKTQGFQKHVGSKT--TLAFMNSNI--FVETSGLPVENWDSAHHSFYFHYSSGF--HHYHT--LHDYEAE

>Marchan_2053

CAVVGNSGEVLNTTRGSFIDSHEMVIRINNAKARVSADFVGSKT--TLMFMNSHI--QFIQKGRAQKLAERKLWELFKQWENVH--DYHYS--SHDYEAE

>Marchan_1521

CAVVGNSGILLNRTFGDFIDSHAMVMRLNNAKLLGFEKHVGTKT--TLSFVNSNI--FHYSSGFQAIVLALGICDKINILGFGK--HHYHT--LHDYAAE

>Chromu_2511

CAVVGSSGALRGTHHGASIDAHTAVIRINAAPTHKHEAAVGRRT--TWRVHNSEK--QSPSTGLLAIAIALGTCERVTLYGFGA--RHYWE--FHDWQAE

>Chromu_2559

CAVVGSAGRLRGSRLGRAIDAADAIFRVNAAPTRKHEADVGART--TWRVHNSEK--QVPSTGLLAIALGLSVCDNVTVYGFSR--RHYWE--FHDWLGE

>Emihu1_229870

CAVVGSGGTLVGSGAGAAIDSHEVVYRFNLAPAERWAGDVGTRT--TFRLFNGQS--RMASTGLLGVALAAAACDHVTLFGFGN--AHYWE--YHDWHAQ

>Emihu1_461764

C--SGRGRR----EYGEHIDANECVFRINRAPTAGYERHVGSRT--TYDFVNSFP--ARPSSGWHVTRLALGVCRKVRLYGFSL--FHYFD--THKFAFE

>Emihu1_98204

CAVVGASGSLRKFEHGAQIDGHSLVLRPNWIINKGFEKNVGTRT--SINVFFGVE--MRPSTGFIAVIIALQICRNVSLFGLT---FHYYG--LHWFEKE

>Emihul_30350

CAVVGAGGSLRKFEHGAQIDGHSLVLRPNWIITKGYENKVGTRT--SINLFFGVE--MRPSTGFISVIIALQICRNVSLFGLT---FHYYG--LHWFEKE

>Emihul_36797

CAVVGASGSLRKFEHGAQIDGHSLVLRPNWIINKGFEKNVGTRT--SINVFFGVE--MRPSTGFIAVIIALQICRNVSLFGLT---FHYYG--LHWFEKE

>Aureococ

CAVVFNSGVLRKFRHGAAIDAHDVVIRINMLNRTGHESFLGSRW--THEFASFQK--SQCSSGFAAALLARSRCANVTVFGAN---YHYTD--PHSFNRE

>Emihu1_106270

CAVVGSSSKLLDAAEGSLIDASELVFRMNHAPVPELRAYIGSRT--DVHVDPIQL--IRPTTGFVALLLALHVCDEARLFGFGM--CSQYK--VHNYNAE

>Emihu1_456754

CAVVGSSAALLARRLGSEIDSHALVIRANQAPRTLVSQESSTRT--NPAVFSGGV--CYPSTGAMAVLYAIDNCRRVTVYGFGS--MHTFL--YHDVVQE

>Emihu1_245446

CAVVGASGNLLGSRYGAEIDSHDAVVRINLAPDGPMTAAAPHRH--EPTWISDGY--KTPSTGMVAIALARKMCGAVHLYGFGN---------------

>Nematostella

CALVTNSADLLGSNAGSVIDSSDCVIRLNTAPTAGFELDVGGKT--TVRIVSQEQ--SRLSPRFHAMNVMHRVCD-----------RHAYP--RHAS-RD

>Orbicella

CALVSSSGMLLGSNAGSQIDSADCVFRLNSAPTLGYERDVGSKT--TVRVVSVTG--FPPPQGFLHVNFLLRVS-KLLYVGFIR--LFYWV---------

>Acropora

CALVSSSGILLGSNAGSQIDSTDCVFRLNSAPTLGFEKDVGSKT--TIRVLSAAS-------------LLCKNCTLSKLYERLS--LLQVT---------

>Chromeria

CALVASGPDMLNAKFGAEIDAHDVIFRMNCAPVNGFESHVGSRT--TFRTTYPES--DLPSEGGTVIGQNLEYYHC-GALG-----YKFAT--FTVLNPE

>Emihu1_102768

CAVVGSGGSLLGARLGASIDAADAVIRVNLAPDARLATDVGSRT--TWRVLAMEG--RVPSTGMNAVAFASQLCGSVHLYGFGN--YHYYD--YHNFSAQ

>Athal_AAF99778

CAVVGNSGDLLKTEFGEEIDSHDAVFRDNEAPVEKYAKYVGVKR--DFRLVVRGA--GAKGTGMKSIELALSMCDIVDIYGFTV--TSKVW--RKKSNYK

>Athal_NP97444

CAVIGNSGDLLKTKFGKEIDTYDTVLRENGAPIQNYKEYVGEKS--TFRLLNRGS--GSAAKGLKALEFALSTCDSVDMYGFTV--LHYYQ--HSPMRAD

>Oriza_Q6ZH45

CAVVGNSGDLLKTKFGDEIDSYDVVIRENGAPIQNYTEYVGTKS--TFRLLNRGS--GSSAKGLKALEFALSMCDSVDMYGFTV--LHYYQ--HSPMRGD

>Sellagin

CAVVGNSGDLLKTEFGKEIDAHDIVIRDNEAPVTKYAKHVGMKR--SFRLMARGV--GAKGTGIKSLELALSMCDSVDMYGFTV--LQYYQ--HSPMREA

>Stram_2792

CAIVGASDGLDGKNLGSEIDSHDAVIRVNDHPTIGFEDDVGRKT--TFRIIVNDV--IADSQGEKVWRAAFGFCDKVSLYGFSF--M-YYW--TRPYDPD

>Bathy5g53

CAVVGNSGMLAYGQNGKEIDSHDVVIRFNGAPTKGLENRVGTKT--TFRLVNSKW--YTPTSGWTGLILAVNVCTEVKLYGMQI--YHYHN--HGRIAWE

>Stram_8625

CAVVGNSGSLLDHTYGSEIDSHDAVIRFNAAPTKGYEKHVGKKT--TIRVQNIDN--LKPSSGFAGVVMALKACSKVSLYGFSH--FHYFN---------

>Stram_699

CAVVGNGGVNMRDQQGYGIDGADIVIRFNDGPTSGFEKYVGRKT--TFRLINNQW--NAPPSGIEGLLLAFAVCDSVRVYGFHT--HH--------
----

>Chloroky_8010

CAIVGNSGALLRAEYGREIDAHDMVWRFNQAPVKGYETHVGART--THESLNGYW--EKPMSGFYVFFFALQVCDEVDLYGFQP--YHYFD--
SHSFDLA

>Chlorok_27068

CAIVGSSGVLLKYDMGEEIDSHDCVIRFNAAPTQGYEKQVGKKT--TMRLVNTQH--TLPTGGFFAVFLALQRCTSVRLYGFHF--HHYFN--
IHDYAAE

>Chlorokyb_958

CAVVGNSGSLLLSKFGSNIDSHSAVIRQNQAPTRSYEKHVGGKT--SIRMLNKIW--STPSSGIYSVALALALCERVTVYGFGT--YQYYR--
VHSFEAE

>Chlorokyb_10426

CAIIGSSGILLNYAHGEEIDSHDIIFRFNSAPTQSFERHSGRRT--THRLTNSRN--FLSTSGLNGIIMALHKCARVDLYGFHI--YHYYN--
CHNVNPD

>Chlorokyb_4572

CAIVGNSGSLLYEQYGKDIDEHDAVFRFNQAPTLGYEVHVGSRT--TFEFLNSAW--RQNTTGLILFEMYDMAAWPFRTRAQIE--RYHYF--
SHAFDLT

>Chlorok_30380

CALVGNARSLTHSGRGTEIDGHEAVLRLNQAPTRGYEKRVGPRT--THRLINHRW--GSPSSGFVGVWLLLQMCQQVDVYGVGD--WHYFE--
HHSWELE

>Bathy16g216

CAVVGNSGVTLLKEYGEEIDNHDAVIRINMAPIRGFEKYVGKRT--TFDVVNSHN--RKPMSGFFAVLYALQMCEKVDLYGFDA--YHYFD--
VHSFDLA

>Emihu1_21127

CAVVGSSGAVLSPRCGRAIDQHEAVFRINTAPVRGFEEYVGSRE--TFRVLNGPY--DYPSTGLLAILVASLRCDSVSLFGFTF--YHYYE--
SHNLTAE

>Bathy1g02080

CAVVGNSQRLLLDLNGKEIDGHSAVIRMNNAPTVGFDRFVGNKT--TLRTLNSIW--GSPSSGFLAIFAALQICDQVTVYGVGT--WHYWE--
HHSWELE

>Bathy08g02300

CAVVGNGGILKSAEFGQAIDAHDVVFRQNQAPTATYELFVGEKT--TFRVLNKKW--NTPSSGIISTVLAMSLCDEVNLYGFGV--YQYYV--
VHSMELE

>Bathy14g02010

CALVGNSGSLLSADFGKEINEHDIVIRFNQAPTKGFEKFVGNRT--TFRVLNALW--HTPSSGFVAIFVLLQLCKDIDLYGFGD--YHYYT--
VHSWVLE

>Bathy2g00330

CAVIGNGGTLLAYDLGEEIDSADVIIRLNAGPIKGFEKKVGSRT--DYRLVNRLH--GVVSNGFYGLLLATELCDQVKVYGFFR--YHYYN--
WHPVPKP

>Bathy14g00960

CAVVGSSGIVLNYEHGGDIDDHDMVFRFNSAPTRGFEKHVGSKT--TYRITNTQN--FLATSGLYGILLALQRCHSVSIYGFQV--YHYYD--
NEDFTKK

>Bathy01g00040

CAVVGSGGIMVHKSLGEAIDKHDAVFRFNLAPTKGFEKSVGSKT--TIRLINRKH--SRPSNGYLGLNLALMICDKINVYGFAR--YHYFN--LRDGKGE

>Bathy01g05530

CAVVGNAGSLRNAKFGEEIDAHDIVLRFNNGRAKHFEKQVGTKG--HLRMYNGPY--EKPSSGMVGITFAMRLCSSVDIYGFSA--PHYYD--VHPFEAE

>Stram_1410

CAVVGSGGILKTYQHGPEIDAHDAIFRINGAPTRGFETIVGANT--TFRVSYPPH--ARPTGGMVAIYVALQACRYVNVYGFTI--YHYFD--VHTFSQE

>Stram_5962

CAVVGNGGILLDNPNGYTIDQHDAVFRINEGPTTGFEKHVGVKT--TVRLSYGPS--QWPTTGIVGIRLATEICDCVNIYGFSA--YHYYD--PHKYNKE

>Guillar133129

CAVVGSSGHLLGSGFGRDIDVHDLVIRFNDAPAGEYESDVGSRT--THRVLHSSG--CVPSSGMIGIMWALQSCLHVDTYGFGR--YKYYG--GHNWNLE

>Guillar145289

CAVVGNAGHIFGSKLGAEIDSHEAVMRFNLAPTESFEEDVGGKT--TWWVTANFP--KIPTSGFLGILWALQECDRVSTYGF-----------------

>Ocar_m43735

CALVGSSYNLMKHEFGAEIDSHSLVARVNDPPITGYEKHVGSRP--DISISNLLI--GAASCGLRSIVFMMHLCKEVHLYGFGG--FKYYS--WHDFNRE

>Azospirillum sp

VAVVGNAPSEIGRKKGAEIDAHDLVIRFNNFRLKGFEDDYGSKT--DIWVRGSGG--DFPTTGMVLIWIFRKFGNNVDFYGFSF--THYFN--VHRLDKE

>Leucothrix

VAVVGNSACLRNSNLGKKIDQQDIVCRFNRYPRASHAPDTGQRI--DIWITSPEV--APPSAGWTNYLISLGLPKNIQLYGFGK--YHIIS--RHAWQDE

>Idiomarina

IAVVGNHPKLKGSKLGDEIDDADFVVRFNQSSETNVE-DIGTKL--DLWVTAPAY--APPSAGVATLAIKSQTSATIELYGFDL--EVYHH--RHNWSAE

>Roseomonas

VAIVGNSPRLMENSHGAAIDSHDLIIRVNDGRIQGFEQHTGQRT--TLRYIGVPL--RPPRSGLSLLVDAMQAGKKVSIFGMET--EHFYA--HCPMERE

>Alteromonas

VAVVGNSPKILGKGLGQEIDTHDDVIRFNGAVISNYVNDVGSKT--TIVFVGIDL--HLKNGGIHYFAGPASAMHNKDLEGLGL--PR-----

>Rhizobium sp. AC44

VAVVGNAPTLAGRKAGKWIDSHDIVIRFNDCRVRGFEADVGSKT--DILISNPYA--IALTTGTYGVSLVANLLKNLSVTGFTM--YHYWS--AHSPQTE

>Sphingobium czech

IAVIGNSPRLGQDRNGERIDAHDVIIRVNDGRTTGFEEFGGSRT--DIRFVGVPL--KPPRSGIVILSFFEKGV-PISLFGFEI--EHYYK--HCPMEVE

>Methylobacterium sals

IAIVGNAPKILEREDGALIDAHSTVIRINDGRSVGFEKHCGAKT--DIRFVGIPI--KPPRTGITSLLAPLFGKSKITLFGFET--EHYYN--HCPMEFE

>Salinisphaera

VALVGNAVSLAERDYGTAIDAADVVVRCNRGILAEYSH--GSRT--DWVVTGLPI--KIASTGMKAIDLAASDCARLDIYGFDF--SHSAS--DHDFDAE

>Paracoccus

VALVGNARALAQTGHGKRIDNADLVIRINRAPMPAAAH--GTRT--DWLALATRL--APPTTGMIALITWSQMA-RLELFGFDF----FAS--PHDFSQE

>Oceanibulbus

VAVIGNSPSVLEKESGQFIDSCDVVIRINNFQTAGFEKYIGSKT--DFAIFTAAS--SWPSTGSVALQLAMDVFSKCDIYGIDF--DHYFS--KHNSHLE

>Paracoccus isoporae

VALVGNARALSGGTQGAEIDDHDRVVRINLAPMPSPEH--GRRT--DWLGLATRL--AQPTTGLIELLLRSELA-RLDLYGFDF----FAS--PHDFTGE

>Loktanella

VAIVGNSSVERGRRKGAEIDGHDIVIRFNLPKQQAFAIDYGSKT--DLIVINHSI--RRGTEGFFGFSMTDQIGDSPTSANYFR--RHNWK-----

>Thioclava sp

VALVGNARALAEGQAGAEIDAHDVVIRINRAPMPSAEH--GTRT--DWLALATSL--AQPTTGLIEMLLRSDLA-RLDLYGFDF----FAS--PHDFAAE

>Thalassobius mediterr

VALVGNAKSLREKSLGGEIDAHDVVIRLNRGPIMSTES--GQKT--TVLATSIPI--SRPTSGVMALHSVLDG-EQVTLFGFDG----FAS--PHDFVSE

>Helicobacter pullorum

IAVVGNSPCEIGRNKGAEIDSHDVVIRFNNFSLKDFIIDYGKKT--NIWVLTPAL--RVMSLGLIVICYLLDKVQNLTLYGFSL--THYFD--IHKWDKE

>Sinorhizobium fredii

VLVLGNAPSVLHRELGREIDKFDVVIRINNFRIRGYEKHVGSKT--DYALISPAC--QWPSTGIVAVQWARDMHKRVHVHGFDF--TRYFD--KHDFDRE

>Prorocentrum minimum

VAVVGNAKSIDEHLFGSEIDSHDVVIRINRGPILSAE-KSGSKT--TVIASSIWV--SRPSSGVMVVA-AVAECNSISIFGFDS--SN-----PHDFDAE

>Pseudovibrio

VAVIGNSKNLLNYQYGSEIDAHDVVVRMNRGVTIPQKNCFGENT--NVWCYSTLK--ARPSVG-AMVTYLLAQCDSVDIYGFDF----FYE--SHNFQRE

>Agrobact arsenij

VLVLGNAPSVLERELGAEIDKFDVVIRVNNFRIRGFEKHIGSKT--TYALISPAC--QWPSTGIVAVQWARDMHKSIYVHGFDF--TRYFN--KHDFDRE

>Sphingobium

CAVVGNSSCEIGRGHGAQIDAHDEVVRFNRFDTPPFDRDYGSRT--TVLVRVGND----PSSGFVHLLKALRGTHREDFFGFAF--AHYFE--MHQWDRE

>Ensifer

VLVVGNAPTLLEREIGDKIDKFDVVIRVNNFRTGGYEKFVGSKT--DYALISPAC--QWPSTGIIAVQWARDRHGKAADVYVHG--DFYSD--KHDFDRE

>Candidatus

IAIVGNSGCEIGLGRGNEIDSHDIVIRFNNYCIDGYEQDYGTKT--DIWVRGSAG--KFPSTGALTVWATYLAKSNLNNVDVYG--NNYSD--DHDFENE

>Drer_ST6GNc4

CAVVSSSGQMLGGGRGPEIDQQDCVIRMNVAPTAGYEADVGNRT--SLRVVSHTS--AFLSTGFFTMILALEVCDSILVYGMID--YHYYE--GHRFITE

>Mmus_ST6GNc3

CAIVSNSGQMVGQKVGEEIDHASCIWRMNNAPTKGFEEDVGYMT--MVRVVSHTS--SYLSTGWFTFILAMDACYSIHVYGMIN--YHYYE--GHRFITE

>Hsap_ST6GNc6

CVIVSSSSHLLGTKLGPEIERAECTIRMNDAPTTGYSADVGNKT--TYRVVAHSS--SWLSTGWFTMVIAVELCDHVHVYGMVP--YHYYE--HHRFITE

>Hsap_ST6GNc5

CALVTSSGHLLHSRQGSQIDQTECVIRMNDAPTRGYGRDVGNRT--SLRVIAHSS--TWLSTGWFTMTIALELCDRINVYGMVP--YHYYE--HHRFITE

>Hsap_ST6GNc4

CAVVSSSGQMLGSGLGAEIDSAECVFRMNQAPTVGFEADVGQRT--TLRVVSHTS--SFLSTGWFTMILALELCEEIVVYGMVS--YHYFE--AHRFITE

>Hsap_ST6GNc3

CAIVSNSGQMVGQKVGNEIDRSSCIWRMNNAPTKGYEEDVGRMT--MIRVVSHTS--SYLSTGWFTFLLAMDACYGIHVYGMIN--YHYYE--GHRFITE

>Drer_ST6GNc6

CALVTSSSHVLGSQAGEEIDRTQCVFRMNNAPTSGHQSDVGNRT--TVRVVAHSS--SWLSTGWFTMVIAIEMCDNIKVYGMVP--YHYYK--HHRFITE

>Trub_ST6GNc5

CALVTSSGRLIRSKRGQEIDRSDCVIRMNDAPTVGHQRDVGQRT--SLRVIAHSS--SWLSTGWFTMAIALELCDRVDVFGMVP--YHYYE--HHRFITE

>Drer_ST6GNc5-A

CALVTSSGHMTGSGRGAEIDETECVIRMNDAPTRRYQKDVGQHT--NLRVIAHSS--SWLSTGWFTMAIAIEMCDRINVYGMVP--YHYYE--HHRFITE

>Drer_ST6GNc3

CSVVSSSGQILGREAGADIDQSSCIWRMNNAPTRGFERDVGHRT--DLRVVSHTS--SYLSTGWFTLILAMDMCKEIRVYGMIN--YHYYE--GHRFITE

>Dsim_ST6GAL_24

CAIVSSAGSLAGSKLGRFIDTHDIVMRFNHAPTQGHEVDVGSKT--TIRVVNSQV--NPPSSGFIGLALLLPHCPQVDFVEYVP--CHYYS--WHPLAAE

>Hsap_ST6Gal2

CAVVMSAGAILNSSLGEEIDSHDAVLRFNSAPTRGYEKDVGNKT--TIRIINSQI--NPPSSGFIGILIMMSMCREVHVYEYIP--CHYHE--YHPLLYE

>Drer_ST6Gal2

CAVVTSAGAMLHSGLGKEIDSHDAVLRFNTAPTVGYERDVGNKT--TIRIINSQI--NPPSSGFIGILLMMSLCEEVHVYEYIP--CHYHE--YHPLLYE

>Cmil_ST6Gal2

CAVVASAGAILNSSLGDEIDSHDAVMRFNAAPTKLYEQDVGSKT--TIRILNSQI--NPPSSGFIGIVIMMALCDSINIYEYIP--CHYHE--YHPLLYE

>Cliv_ST6GAL1

CAVVSSAGSLKSSHLGQEIDSHDAVLRFNGAPIKGFQEDVGQKT--TIRLVNSQL--NPPSSGMLGIVIMMTLCDEVHVYEFLP--CHYYQ--YHPLLFE

>Hsap_ST6Gal1

CAVVSSAGSLKSSQLGREIDDHDAVLRFNGAPTANFQQDVGTKT--TIRLMNSQL--NPPSSGMLGIIIMMTLCDQVDIYEFLP--CYYYQ--YHPLLYE

>Bmor_ST6GAL_2414

CAVVSSAGALLGSRLGDFIDSHDMVLRFNNAPTDNYTDDVGSKT--TFRILNSQV--NPPSSGFIGLWFSLNRCRRVRVFEYVP--CHYYA--WHPLAQE

>Bflo_ST6GAL_2426

CAAVMSSGAMLGSRLGKDIDSHDAVLRYNSAPTKRYQSDVGSKT--TLRLMNSQV--NVPSSGFMGIHLLLSLCDTVDVYEYVP--CHYYG--YHPLRAE

>Aaeg_ST6GAL_247

CAVIASAGSLKGSRLGDFIDDHDVVMRFNHAPTKGFEVDVGSKT--TVRVVNSQV--NPPTSGFIGLGLLIPACRYIDMIEYIP--CHYYD--WHPLAAE

>Afor_ST6GNc_2265

CAIIGNGGVMNGSNKGEEIDAHDYVFRVNVALTKGFEKDVGSKT--SFYCFTMVT--HRPTTGAIMLLLALHTCDEVNVYGFGG--EHYYD--NHDNNAE

>Lpol_ST6GAL_2415

CAIVSSSGSMYKSGLGKEIDTHDVVLRFNDAPTKGYEKDVGNKT--TVRILNSQV--NPPSSGFLGLLLLLQHCNTVDIYEYIP--CHYYD--WHPLASE

>Phumcor_ST6GAL_2412

CAIVSSAGSLLNSKLGHLIDQHEIVVRFNHAPTVTYEEDVGSKT--TVRLVNSQV--NPPSSGFLGLALLMPYCDTIDFFEFVP--CHYFE--WHPLSAE

>Pmar_ST6GAL_2423

CAVVSSAGAMLGSGLGREIDAHDAVLRFNAAPTEHFEKDVGTKT--TIRLVNSQI--NPPSSGFLGVVLMMWLCDEVNVYEFLP--CHYHE--YHPLLYE

>Skow_ST6GAL_2427

CAIVASAAYFKNATLGKEIDSHDAVLRFNDAPTEGFEENVGRKT--TIRLMNTLI--TLPSSGIMGISLMQRMCDTVNVYGYLI--CHYYD--WHPVEYE

>Skow_ST6GAL_02428

CAIVSSSSFMTDSGLGEEIDSHEAVMRFNNAPTVGFERDVGNKT--TMRVINLDI--HVPSSGFTGIILMQQICDQVNVYGFVT--LHYYS--WHPMVQE

>Skow_ST6GAL_02429

CAVVLSSFYMNETGFGDEIDSHEAVLRLNDAPTVGYEKSVGRKT--TIRLLNSKC--IVPSSGFTGLLMMLGLCDKVDVYGFGI--CHYYD--WHPLQ-E

>Skow_ST6GAL_02430

CAVIGSSYFLLGSKLGKEIDSHDAVLRFNDALVTGFERDVGSKT--TIRLINSQM--IVPTTGFTGIHLMHHICESIDVYGYVT--CHYYD--WHPVEAE

>Isca_ST6GAL_2411

CALVSSAGSLLGSRLGNEIGAHQAVLRFNDAPTQGFEQDVGSRT--TVRLLNSQL--NPPSSGFLGLLLLLGLCRRLDAYELVP--CHYYE--WHPLAAE

>Apis_ST6GAL_02408

CAIISNAATLRNSNLGYFIDQHDLVLRFNNAPTKGYEKDVGSKT--TIRILNSQV--NPPSSGFLGLAMLLPRCTVVNMFEFIP--CHYYH--WHPLAAE

>Tcas_ST6GAL_02413

CAIVASSGALKDSNLGKKIDAHDLVLRFNHAPTKGFEPDVGRKT--TIRVLNSQV--NPLSSGFLGLGILLPLCNFIDVFEYVP--CHYYD--WHPLAAE

>Acar_ST6Gal1

CAVVSSAGSMKSSHLGEDIDSHDAVLRFNGAPIKGFQADVGEKT--TIRLVNSQL--NPPSSGMLGKLIMMNFCDEVDVYEYLP--CHYYQ--YHPLLFE

>Tgut_ST6Gal1

CAVVSSAGSLKSSHLGQEIDSHDAVLRFNGAPVRGFQDDVGQKT--TIRLVNSQL--NPPSSGMPGIVLMMTLCDQVDVYEFLP--CHYYQ--YHPLLFE

>ocar_41732

CAVVSSASSLLRYEFGPLIDSHDAVIRLNGAPTIGYEKHVGSKT--SLRVINARV--ISSSSGFHAVLMALFLCDSVSSFEVAT--HSYYR--WHPLNLE

>ocar_m1871

CAVVSSGPSMLKFKLGKEIDSHDAIFRINMAPTKSFEKYVGSKT--TIRVLNSKI--LSFSSGTFAVLSAIWLCEYVTSYEIAS--SYYFD--FHPLPAE

>ocar_m126566

CAVVSSAPSLLKYQMGNEIDSHDAVIRINLAPTKEFEKHVGKKT--TIRFINSGV--YTLSSGSQAFLMALFMCESVTAYELAT--QYYFD--VHPLDIE

>ocar_m311493

CSLVSSSGRILGQSKGAEIDGADCVLRMNIAPVKGYEGDVGKRT--TARILSQFS--GNVHLGQKTANYKKMLEKAIQLPNIGF--YHYWD--AHRFMTE

>ocar_m63885

CSLVGSSGHLLSEKAGEEIDKADCIFRMNQAPVRGFEKDVGRRT--TVRVLNHVC--SWLTTGWFTFDLVLRACDQVSVYGLPP--YHYFN--HHRFVTE

>ocar_m10101

CALVGNSGHLLGSRCGQLINKADCIFRMNEAPVPGFKKDVGNRT--DVRIVNFHI--SWLSTGWIAFDVVLRACDSVAVYGFPP--YHYYG--HHRFADE

>ocar_m178569

CAVVGNSDILSGSNKGQLIDSHEVVFRFNNYETKGYEKDVGNKT--TFQNLYRRI--NIPSSGIYTLLLALYVCDEVSIFGFGV--GSYYG--YHMWDTE

>Cint_ST3Gal_00828

CAVVGNSGNILNSNYGNVIDGHDFVIRMNKGPTYNYENDVGSKT--THRFMYPTT--RYPSTGLLALIYALHECDEVDVYGFGA--HHYWE--VHDSAQE

>D2KQ2_CAMJU

IIIAGNGPSLKNINYKRLPREYD-VFRCNQFYFED-KYYLGKKI--KAVFFNPGV--KRITSGVYM-CAIAIALGYKTIYLCGI--IYPFE--CHSKEYD

>Q7BP25_CAMPJU

ALVCGNGPSLKNIDYKRLPKQFD-VFRCNQFYFED-RYFVGKDV--KYVFFNPFV--QRITSGVYM-CATAVALGYKSIYISGI--LYAFD--NHSMACD

>Q9CLP3_Pasteurm

VIVAGNGESLSQIDYRLLPKNYD-VFRCNQFYFEE-RYFLGNKI--KAVFFTPGV--QRITSGVYM-CAVAIAMGYTDIYLTGI--NYAFD--YHSKDID

>Q17WF9_ST3_8_He

LIIAGNGPSIKDLDYALFPKDFD-VFRCNQFYFED-KYYLGREI--KGVFFNAHV--QHFTTGIMM-LLVAIQLGYKEIYLCGI--GHFYE--MHDKNID

>A6YD31_Lic3B

VIIAGNGTSLKSIDYSLLPKDYD-VFRCNQFYFED-HYFLGKKI--KKVFFNYSV--KRITSGVYM-CAVATAMGYKDLYLTGI--PYAFH--IHSMEYD

3 - MSA of all the ST motifs sequences carried out with MUSCLE in MEGA7.0.18 [1]

185 100

Spur_ST3Ga

CVVQGNGGIA SRTRLGEVID NFDVVFRLNS APTINHENDV GRKT--TFRM AYPES--LQD TASMMGM-MG VRLCDEVAVA

GFGY--LHYY D--THDIHHE

Spur_ST3Ga

CIVIGNGGIL KGSKLGSHID QYDAVFRLNV APIKGYESDV GTKT--TVRA VYPES--NVP TTGAMVIMVA IRVCDEVSVA

GFGY--IHYY S--THDIDHE

Hpul_ST3Ga

CVVQGNGGIA SRTRLGEVID NFDVVFRLNS APTINHENDV GRKT--TFRM AYPES--LQD TANMMGM-MG VRLCDEVAVA

GFGY--LHYY D--THDIHHE

Skow_ST3Ga

CIIVGSGGIL KNKGHGRVID EYDVVIRMNV SPVKGYEKDV GTKT--TIRI TYPEG--NVP TSGTISIMLA LRLCDEVDVV

GFGY--IHYY E--THDINHE

Bflo_ST3Ga

CIVVGSSGLV SGENLGSTID KYDIVMRMNE APVHGYEKDI GSKT--TFRF LYPES--NIP TIGTISIITA FHFCDMVDIV

GFGF--YHYY E--IHDFAKE

Skow_ST3Ga

CILVASSGVS TGKGLGKLID KYDVVIRMNN APVKKYEKDV GSKT--TFRL VYPES--KRP STGAMAVVMA LHYCDHLHIT

GYGY--IHYY E--AHSWFNE

Skow_ST3Ga

CVLVANSGIL IGSKLGDVID TYDIVIRMNN AKTVGYADDV GRKT--TFRF IYPES--KRP STGIVAMVFA FHYCDIVDIA

GYG---NHYF S--THCWDNE

Hsap_ST3Ga

CIIVGNGGVL ANKSLGSRID DYDIVVRLNS APVKGFEKDV GSKT--TLRI TYPEG--NIP TLGSVAVTMA LHGCDEVAVA

GFGY--LHYY E--THNIQRE

Lcha_ST3Ga

CIVVGNGGVL ANKSLGSKID EYDVIIRLNG APVKGYEKDV GAKT--TIRI TYPEG--NIP TLGTVAITMA LHNCDEVAVA

GFGY--LHYY E--THNIGRE

Drer_ST3Ga

CAVIGNGFAL KNSSLGEIIN KYDVVIRLND APVRGFEEDV GNKT--TLRL FYPES--VHP TTGLLAVFVA LNYCDVVHVA

GFGY--IHYY G--YHDLNQE

Hsap_ST3Ga

CVVVGNGHRL RNSSLGDAIN KYDVVIRLNN APVAGYEGDV GSKT--TMRL FYPES--QKP TTGLLAITLA LHLCDLVHIA

GFGY--IHYY E--GHNVSQE

Mdom_ST3Ga

CVVVGNGHRL KNSSLGNTID KYDVVIRLNN APVVGYEGDV GSKT--TMRL FYPES--QKP TTGLLAITLA LHLCDLVHIA

GFGY--IHYY E--EHNVSHE

Acar_ST3Ga

CAVVGNGHRL RNSSMGDVIN KYDVVIRLNN APVHGYEHDV GSKT--TMRL FYPES--QKP TTGLVAITLA LHFCDMVDIA

GFGY--IHYY E--GHNVSHE

Lcha_ST3Ga

CIVVGNGGVL RNKTLGEKID SYDIIIRLNN GPVIGHEKDV GKRT--TFRL CYPES--VHP TTGIIATALA LHLCDETHIV

GFKY--LHYY E--YHNITAE

Hsap_ST3Ga

CVVVGNGGVL KNKTLGEKID SYDVIIRMNN GPVLGHEEEV GRRT--TFRL FYPES--KHP TTGIIAITLA FYICHEVHLA

GFKY--LHYY G--YHNVTAE

Hsap_ST3Ga

CVVIGSGGIL HGLELGHTLN QFDVVIRLNS APVEGYSEHV GNKT--TIRM TYPEG--NVP TIGVIAVVLA THLCDEVSLA

GFGY--LHYF D--MHNVTTE

Drer_ST3Ga

CVVVGNGGIL KGLGLGHLLN RFDIIIRLNS GPLQDFSADV GNRT--TIRM SYPES--NIP TLGLTALNLA TYICDEVSLA

GFGY--LHYY D--MHNVQKE

Drer_ST3Ga

CVVVGSRGIL HSKNLGAHID HANIIIRVNN APVFGFESDA GSRT--TIRL IYPEG--MVP TLGITAVVVA LQVCDEVSIA

GFGY--LHYY G--VHDVSAE

Lcha_ST3Ga

CVVVGSAGIL HGSRLGAYID QHDIIIRKNG ICLKGYTVNI QIKT--VVGI SEYER--MVP TLGVSAVVMA THLCDEVSLA

GFGY--LHYF E--MHNVDTE

Ggal_ST3Ga

CIVVGNGYSV HGQHFGKMID SHHVIIRLND APVKEYKKDV GERT--SIRL FFPES--RYA TTGIIALNLA LHLCQEVNIA

GFGY--IHYY N--QHNLTAE

Spur_ST3Ga

CVVVGNGGVM KQSAMGPIID DFDVVIRLND APTVGYEKDV GSKT--TIRM AYPES--NVP TTGSFAISMA TRLCDEVSVA

GFGY--LHYY D--THNIDIE

Bflo_ST3Ga

CIVVGSGGIL LGKKLGPQID DFDIVIRMNN GPVKGYEEDV GHKT--TIRM SYPEG--NVP TIGSLAIIWA INYCDEVTVA

GFGY--LHYY K--THDINKE

Bflo_ST3Ga

CIVVGSGGIL LGKKLGPQID DFDIVIRMNN GPVKGYEEDV GHKT--TIRM SYPEG--NVP TIGSLAIIWA INYCDEVTVA

GFGY--LHYY K--THDINKE

Hsap_ST3ga

CAVVGNSGNL RESSYGPEID SHDFVLRMNK APTAGFEADV GTKT--THHL VYPES--RYP STGILSVIFS MHVCDEVDLY

GFGA--HHYW E--VHDADFE

Olat_ST3Ga

CSVVGNSGNL KGSNYGALID TSDLVIRMNK APTKGFEKDV GAKT--THHV MYPES--HYP STGFLTLMFA LHICDEVSVF

GFGA--HHYW E--VHDGDYE

Lcha_ST3Ga

CAVVGNSGNL HGSGYGKAID MHDFIMRINQ APTVGFEADV GSRT--THHF MYPES--RYP STGMLVLFFA LHVCDEVDVY

GFGA--HHYW E--VHDADFE

Hsap_ST3ga

CAVVGNSGNL RGSGYGQDVD GHNFIMRMNQ APTVGFEQDV GSRT--THHF MYPES--RYP STGMLVLFFA LHVCDEVNVY

GFGA--HHYW E--VHDADFE

Ggal_ST3Ga

CAVVGNSGRL KGSRHGLQID AHHWVLRMNR AKTAGFEMDV GART--THHF MYPES--RYP STGFTALLFA LHACQQVSVF

GFGA--HHYW E--VHDADVE

Lcha_ST3Ga

CAVVGNSGNL KGSNHGKEID AHHFVIRMNR ARTAGFEPDV GIKT--THHL MYPES--RYP STGMTALVFA LHICDEVSVF

GYGA--HHYW E--VHSGDFE

Drer_ST3ga

CAVVGNSGNL LKSKYGALID SHSTVIRMNK AVTVGYDEDV GYRT--THHF LYPES--RYP STGIVAIIFA LHLCDEVSVF

GYGA--HHYW E--VHNADFE

Bflo_ST3Ga

CAVVGNSGNL RQSNYGEEID GYDLIFRMND APTKGWEKDV GHRT--THHF MYPES--RYS STGSLVILFA VHVCDEVDVY

GYGA--NHYW T--VHDSEFE

Bflo_ST3Ga

CAVVGNSGNL RQSNYGEEID GYDLIFRMND APTKGWEKDV GHRT--THHF MYPES--RYS STGSLVILFA VHVCDEVDVY

GYGA--NHYW T--VHDSEFE

Csav_ST3Ga

CAVVGNSGNL INSKYGN-ID SHDFVIRLNK GPTEGFENDV GRKT--THRF MYPAT--RYP STGLIAIIYA LHECDQVDLY

GFGA--HHYW E--VHDSDKE

Skow_ST3Ga

CAVVGNSGNL RDSRYGQLID SHDYIMRMNT AKTVGFEKDV GSRT--THHF MYPES--RYP STGMLVLLFA MHICDQVNVF

GFGA--DHYY Q--VHNAEFE

Spur_ST3Ga

CAVVGNSGNL RNSGYGTAID KHDVVVRINQ AKVKGFEKDV GQKE--THRL MYPES--RYP SSGTLAVFFA LQFCDEVSVY

GMGA--DHYW E--VHDSVHE

Ocar_ST3Ga

CAVVGTAGRL KGARQGKLID SFDIVIRMNR SPVKGYEVDV GSKT--SYHL VYPES--RWA SAGALSVVWA LHICNEVDVF

GFGA--DHYY E--VHDANIE

ocar_m1082

CAVVGTAGRL KGARQGKLID SFDIVIRMNR SPVKGYEVDV GSKT--SYHL VYPES--RWA SAGALSVVWA LHICNEVDVF

GFGA--DHYY E--VHDANIE

Bathy_4485

CAVVGNGGIL KSAEFGQAID AHDVVFRQNQ APTATYELFV GEKT--TFRV LNKKW--NTP SSGIISTVLA MSLCDEVNLY

GFGV--DRNV G--VHSMELE

Hsap_ST6GN

CAVVGNGGIL NNSHMGQEID SHDYVFRLSG ALIKGYEQDV GTRT--SFYG FTAFS--YRP TTGALLLLTA LQLCDQVSAY

GFIT--DHYY D--NHDFKLE

Drer_ST6GN

CAVVGAGGIL NNSKMGREID SHDYVFRVNG AVTKGYEEDV GNRT--SVYV HTAFS--YRP TNGAFALFLA IHTCDMVNAY

GFIT--NYYY E--NHDYGLE

Hsap_ST6GN

CAVVGNGGIL NGSRQGPNID AHDYVFRLNG AVIKGFERDV GTKT--SFYG FTVNT--YMP STGALMLLTA LHTCDQVSAY

GFIT--DHYF E--NHDLSLE

Lcha_ST6GN

CAVVGNGGIL NDSQMGREIN QHDYVFRANG AVVKGFEKDI GNRT--SFYI FSTNT--YRP STGAVMLLAA IHTCDEVSAY

GFMT--DHYF D--NHDYRLE

Drer_ST6GN

CAVVGNGGIL NGSRKGTEID AHDYVFRVNG AALNGFEKDV GSRT--SFYT FSTNT--YRP STGAVMLLAA IHTCDQVDAY

GFMT--DHYY D--NHDMRME

Bflo_ST6GN

CAVVGNGGIL RGSGKGKEID GHDFVWRVNS AIIEGYEEDV GKRT--SFYF HDINT--YRP TTGGSMLLTA LHTCDVTDVY

GFIT--DHYY E--NHDFQME

Bflo_ST6GN

CAVVGNGGIL RGSGKGEEID AHDFIFRVNA AIVKGFEADV GSRT--SFYF HTATT--YRP STGAVALLTA VHVCDVTDAY

GFIT--NHYY E--NHDFNLE

Skow_ST6GN

CAVVGGGGIL NGSRKGEEID SHDYVFRVNI AATKGYEVDV GKKT--SFYS YTITT--NRP STGALMLIAA IHTCDEVSAY

GFGV--THYY D--NHDFKQE

Skow_ST6GN

CAVVGNGGVL KNSKLGEEIN SHDYVFRVNV AVTKGFESDV GDKT--SHYV FTMVT--YRP QTGAFMLLVA LHTCDEVDVY

GVGA--DHYY D--NHDYTTE

Lvarvar_ST

CAIIGNGGIL KGSGKGAEID AHDYVFRVNA AVTKGFENDV GKKT--SFYC FTMVT--HRP TTGAIMLLAA LHTCDQVSIY

GFAG--EHYY D--NHDNNAE

Spur_ST6GN

CAVIGNGGIL NGSGKGAEID AHDYVFRVNA AITKGFEKDV GKRT--SFYC FCMHS--HRP TTGAIMLLAA LHTCDQVSIY

GFAG--EYYY D--NHDYNSE

Ajap_ST6GN

CAVIGNGGIL NGSRKGQEID AHDYVFRVNT ALTTGFEEDV GRRT--SFYC FTMIT--HRP TTGAIMLLAA LHTCDQVDIY

GFGG--EHYY D--NHDNIAE

Mgla_ST6GN

CAIIGNGGVM NGSNKGEEID AHDYVFRVNV ALTKGYEKDV GSKT--SFYC FTMIT--HRP TTGAIMLLLA LHTCDEVNVY

GFGG--EHYY D--NHDNNAE

dre_ST8s2_

CAIVGNSGIL LNSSCGREID SHDFVIRCNL APVEEYAADV GLRT--SLVT MNPSV--KRP TTGLLMYTMA TRFCDEIHLY

GFWP--YHYY D--PHTMPLE

dre_ST8s3_

CAVVGNSGIL TGSRCGPEID KYDFVFRCNF APTEVFRRDV GRRT--NLTT FNPSI--KRL STGILMFTLA SSLCEQVHLY

GFGW--YHYY D--SHQLPTE

Dre_ST8s4_

CAVVGNSGIL LKSGCGKEID NHSFVIRCNL APLEGFADDV GLRS--DFTT MNPSV--KRP TTGLLMYTMA TRFCDEIYLY

GFWP--YHYF D--PHRMPLE

dre_ST8s5_

CAVIGNGGII KNSKCGREID ASDFVFRCNI PPVDLYSQDV GSKT--DLVT INPSI--KRL SSGLMLVTAA MELCEEVHLY

GFWA--HHYY D--FHAMPYE

Hsa_ST8s2_

CAIVGNSGVL LNSGCGQEID AHSFVIRCNL APVQEYARDV GLKT--DLVT MNPSV--KRP TTGLLMYTLA TRFCKQIYLY

GFWP--YHYY D--PHTMPLE

Hsa_ST8s3_

CAVVGNSGIL TFIQCGREID KSDFVFRCNF APSEAFQRDV GRKT--NLTT FNPSI--KRL STGILMYTLA SAICEEIHLY

GFGF--YHYY D--SHQLPAE

Hsa_ST8s4_

CAVVGNSGIL LDSECGKEID SHNFVIRCNL APVVEFAADV GTKS--DFIT MNPSV--KRP STGLLMYTLA TRFCDEIHLY

GFWP--YHYY D--PHRMPLE

Hsa_ST8s5_

CAVVGNGGIL KNSRCGREIN SADFVFRCNL PPIEKYTMDV GVKT--DVVT VNPSI--KRI STGLILVTAA LELCEEVHLF

GFWA--HHYY D--GHAMPSE

Dre_ST8s6_

CAVVGNGSVL ANSSCGEEIN SAQFVIKCNL PPLDRYEKDV GNKT--NLVT ANPSI--VRP STGLIMASLA LEICTNVHLY

GFGK--NHYY D--VHSMPSE

Hsa_ST8s6_

CAVVGNGGIL NKSLCGTEID KSDFVFRCNL PPTTGDVKDV GSKT--NLVT INPSI--YRL STGLMITSVA VELCKNVKLY

GFWP--HHYY D--FHQMPKE

dre_ST8s1_

CSVVGNGGVL KHSGCGNEID RADFIMRCNL PPLKDYTDDV GTKT--HLVS ANPSI--KRL STGLFLVSLA LGLCEEVTAY

GFWP--HHYY D--FHAMPEE

Hsa_ST8s1_

CAVVGNGGIL KKSGCGRQID EANFVMRCNL PPLSEYTKDV GSKS--QLVT ANPSI--KRL STGLFLVSAA LGLCEEVAIY

GFWP--HHYY D--FHAMPEE

Bathy02g01

CAVVGNAGTL LKSKYGEAID KHDVVMRFNV MTLAQLAANV GTRT--TFRM VNHLR--KQG TSGFHGILLL AGMCDHLSLY

GFSA--DQYG G--WHDWEGE

Athal_NP17

CAVVGNSGTL LNSQYGDLID KHEIVIRLNN AKTERFEKKV GSKT--NISF INSNI--FHY SSGMQAVMLA VGICEKVSVF

GFGK--HHYH T--LHDYEAE

Oryza_AM04

CAVVGNSGVL LGSGRGPQID AHDLVIRLNN ARVAGFAADV GVKT--SLSF VNSNI--FHY SSGMQAVVMA LGVCDEVSLF

GFGK--HHYH T--LHDYEAE

Physco_122

CAVVGNNGIL LNSTFGKAID AHKVVVRLNN ARIKGFEKHL GGKT--TIAF MNSNI--FHY SSGMQAVMLA LGICEEVDLY

GFEK--HHYH T--IHDYEAE

Physco_488

CAVVGNSNDL LQDLFGAEID EFDAVIRMNG APVENYTHYV GEKT--TFRI LNRGS--SAK GTGVKAIEFA LSVCEMVDIY

GFTV--TRYF S--AYYQTME

Physco_106

CAVVGNSGDL LLAKFGSEID AHEVVLRDNE APVNKTYDKH VGRK--TFRL IGEGV--GAK GTGIKSIELA VSMCDVVDIY

GFTV--TRYF S--AYYQLLE

Physco_512

CAVVGNSGIL LNSTFGKMID AHEEIVRLNN ARIKGFEKYV GGKT--TIVF MNNNI--WPW VQGFHYSSGM QAVMLALGIC

EEVD--HHYM A--IHDYEAE

Marchan_33

CAVVGNSGIL LNSSYGALID SHEMVIRLNN AKTQGFQKHV GSKT--TLAF MNSNI--FVE TSGLPVENWD SAHHSFYFHY

SSGF--HHYH T--LHDYEAE

Marchan_20

CAVVGNSGEV LNTTRGSFID SHEMVIRINN AKARVSADFV GSKT--TLMF MNSHI--QFI QKGRAQKLAE RKLWELFKQW

ENVH--DYHY S--SHDYEAE

Marchan_15

CAVVGNSGIL LNRTFGDFID SHAMVMRLNN AKLLGFEKHV GTKT--TLSF VNSNI--FHY SSGFQAIVLA LGICDKINIL

GFGK--HHYH T--LHDYAAE

Chromu_251

CAVVGSSGAL RGTHHGASID AHTAVIRINA APTHKHEAAV GRRT--TWRV HNSEK--QSP STGLLAIAIA LGTCERVTLY

GFGA--RHYW E--FHDWQAE

Chromu_255

CAVVGSAGRL RGSRLGRAID AADAIFRVNA APTRKHEADV GART--TWRV HNSEK--QVP STGLLAIALG LSVCDNVTVY

GFSR--RHYW E--FHDWLGE

Emihu1_229

CAVVGSGGTL VGSGAGAAID SHEVVYRFNL APAERWAGDV GTRT--TFRL FNGQS--RMA STGLLGVALA AAACDHVTLF

GFGN--AHYW E--YHDWHAQ

Emihu1_461

C--SGRGRR- ---EYGEHID ANECVFRINR APTAGYERHV GSRT--TYDF VNSFP--ARP SSGWHVTRLA LGVCRKVRLY

GFSL--FHYF D--THKFAFE

Emihu1_982

CAVVGASGSL RKFEHGAQID GHSLVLRPNW IINKGFEKNV GTRT--SINV FFGVE--MRP STGFIAVIIA LQICRNVSLF

GLT---FHYY G--LHWFEKE

Emihul_303

CAVVGAGGSL RKFEHGAQID GHSLVLRPNW IITKGYENKV GTRT--SINL FFGVE--MRP STGFISVIIA LQICRNVSLF

GLT---FHYY G--LHWFEKE

Emihul_367

CAVVGASGSL RKFEHGAQID GHSLVLRPNW IINKGFEKNV GTRT--SINV FFGVE--MRP STGFIAVIIA LQICRNVSLF

GLT---FHYY G--LHWFEKE

Aureococ

CAVVFNSGVL RKFRHGAAID AHDVVIRINM LNRTGHESFL GSRW--THEF ASFQK--SQC SSGFAAALLA RSRCANVTVF

GAN---YHYT D--PHSFNRE

Emihu1_106

CAVVGSSSKL LDAAEGSLID ASELVFRMNH APVPELRAYI GSRT--DVHV DPIQL--IRP TTGFVALLLA LHVCDEARLF

GFGM--CSQY K--VHNYNAE

Emihu1_456

CAVVGSSAAL LARRLGSEID SHALVIRANQ APRTLVSQES STRT--NPAV FSGGV--CYP STGAMAVLYA IDNCRRVTVY

GFGS--MHTF L--YHDVVQE

Emihu1_245

CAVVGASGNL LGSRYGAEID SHDAVVRINL APDGPMTAAA PHRH--EPTW ISDGY--KTP STGMVAIALA RKMCGAVHLY

GFGN------ ----------

Nematostel

CALVTNSADL LGSNAGSVID SSDCVIRLNT APTAGFELDV GGKT--TVRI VSQEQ--SRL SPRFHAMNVM HRVCD-----

------RHAY P--RHAS-RD

Orbicella

CALVSSSGML LGSNAGSQID SADCVFRLNS APTLGYERDV GSKT--TVRV VSVTG--FPP PQGFLHVNFL LRVS-KLLYV

GFIR--LFYW V---------

Acropora

CALVSSSGIL LGSNAGSQID STDCVFRLNS APTLGFEKDV GSKT--TIRV LSAAS----- --------LL CKNCTLSKLY

ERLS--LLQV T---------

Chromeria

CALVASGPDM LNAKFGAEID AHDVIFRMNC APVNGFESHV GSRT--TFRT TYPES--DLP SEGGTVIGQN LEYYHC-GAL

G-----YKFA T--FTVLNPE

Emihu1_102

CAVVGSGGSL LGARLGASID AADAVIRVNL APDARLATDV GSRT--TWRV LAMEG--RVP STGMNAVAFA SQLCGSVHLY

GFGN--YHYY D--YHNFSAQ

Athal_AAF9

CAVVGNSGDL LKTEFGEEID SHDAVFRDNE APVEKYAKYV GVKR--DFRL VVRGA--GAK GTGMKSIELA LSMCDIVDIY

GFTV--TSKV W--RKKSNYK

Athal_NP97

CAVIGNSGDL LKTKFGKEID TYDTVLRENG APIQNYKEYV GEKS--TFRL LNRGS--GSA AKGLKALEFA LSTCDSVDMY

GFTV--LHYY Q--HSPMRAD

Oriza_Q6ZH

CAVVGNSGDL LKTKFGDEID SYDVVIRENG APIQNYTEYV GTKS--TFRL LNRGS--GSS AKGLKALEFA LSMCDSVDMY

GFTV--LHYY Q--HSPMRGD

Sellagin

CAVVGNSGDL LKTEFGKEID AHDIVIRDNE APVTKYAKHV GMKR--SFRL MARGV--GAK GTGIKSLELA LSMCDSVDMY

GFTV--LQYY Q--HSPMREA

Stram_2792

CAIVGASDGL DGKNLGSEID SHDAVIRVND HPTIGFEDDV GRKT--TFRI IVNDV--IAD SQGEKVWRAA FGFCDKVSLY

GFSF--M-YY W--TRPYDPD

Bathy05g05

CAVVGNSGML AYGQNGKEID SHDVVIRFNG APTKGLENRV GTKT--TFRL VNSKW--YTP TSGWTGLILA VNVCTEVKLY

GMQI--YHYH N--HGRIAWE

Stram_8625

CAVVGNSGSL LDHTYGSEID SHDAVIRFNA APTKGYEKHV GKKT--TIRV QNIDN--LKP SSGFAGVVMA LKACSKVSLY

GFSH--FHYF N---------

Stram_6099

CAVVGNGGVN MRDQQGYGID GADIVIRFND GPTSGFEKYV GRKT--TFRL INNQW--NAP PSGIEGLLLA FAVCDSVRVY

GFHT--HH-- ----------

Chloroky_8

CAIVGNSGAL LRAEYGREID AHDMVWRFNQ APVKGYETHV GART--THES LNGYW--EKP MSGFYVFFFA LQVCDEVDLY

GFQP--YHYF D--SHSFDLA

Chlorok_27

CAIVGSSGVL LKYDMGEEID SHDCVIRFNA APTQGYEKQV GKKT--TMRL VNTQH--TLP TGGFFAVFLA LQRCTSVRLY

GFHF--HHYF N--IHDYAAE

Chlorokyb_

CAVVGNSGSL LLSKFGSNID SHSAVIRQNQ APTRSYEKHV GGKT--SIRM LNKIW--STP SSGIYSVALA LALCERVTVY

GFGT--YQYY R--VHSFEAE

Chlorokyb_

CAIIGSSGIL LNYAHGEEID SHDIIFRFNS APTQSFERHS GRRT--THRL TNSRN--FLS TSGLNGIIMA LHKCARVDLY

GFHI--YHYY N--CHNVNPD

Chlorokyb_

CAIVGNSGSL LYEQYGKDID EHDAVFRFNQ APTLGYEVHV GSRT--TFEF LNSAW--RQN TTGLILFEMY DMAAWPFRTR

AQIE--RYHY F--SHAFDLT

Chlorok_30

CALVGNARSL THSGRGTEID GHEAVLRLNQ APTRGYEKRV GPRT--THRL INHRW--GSP SSGFVGVWLL LQMCQQVDVY

GVGD--WHYF E--HHSWELE

Bathy16g02

CAVVGNSGVT LLKEYGEEID NHDAVIRINM APIRGFEKYV GKRT--TFDV VNSHN--RKP MSGFFAVLYA LQMCEKVDLY

GFDA--YHYF D--VHSFDLA

Emihu1_210

CAVVGSSGAV LSPRCGRAID QHEAVFRINT APVRGFEEYV GSRE--TFRV LNGPY--DYP STGLLAILVA SLRCDSVSLF

GFTF--YHYY E--SHNLTAE

Bathy01g02

CAVVGNSQRL LLDLNGKEID GHSAVIRMNN APTVGFDRFV GNKT--TLRT LNSIW--GSP SSGFLAIFAA LQICDQVTVY

GVGT--WHYW E--HHSWELE

Bathy08G02

CAVVGNGGIL KSAEFGQAID AHDVVFRQNQ APTATYELFV GEKT--TFRV LNKKW--NTP SSGIISTVLA MSLCDEVNLY

GFGV--YQYY V--VHSMELE

Bathy14g02

CALVGNSGSL LSADFGKEIN EHDIVIRFNQ APTKGFEKFV GNRT--TFRV LNALW--HTP SSGFVAIFVL LQLCKDIDLY

GFGD--YHYY T--VHSWVLE

Bathy02g00

CAVIGNGGTL LAYDLGEEID SADVIIRLNA GPIKGFEKKV GSRT--DYRL VNRLH--GVV SNGFYGLLLA TELCDQVKVY

GFFR--YHYY N--WHPVPKP

Bathy14g00

CAVVGSSGIV LNYEHGGDID DHDMVFRFNS APTRGFEKHV GSKT--TYRI TNTQN--FLA TSGLYGILLA LQRCHSVSIY

GFQV--YHYY D--NEDFTKK

Bathy01g00

CAVVGSGGIM VHKSLGEAID KHDAVFRFNL APTKGFEKSV GSKT--TIRL INRKH--SRP SNGYLGLNLA LMICDKINVY

GFAR--YHYF N--LRDGKGE

Bathy01g05

CAVVGNAGSL RNAKFGEEID AHDIVLRFNN GRAKHFEKQV GTKG--HLRM YNGPY--EKP SSGMVGITFA MRLCSSVDIY

GFSA--PHYY D--VHPFEAE

Stram_1410

CAVVGSGGIL KTYQHGPEID AHDAIFRING APTRGFETIV GANT--TFRV SYPPH--ARP TGGMVAIYVA LQACRYVNVY

GFTI--YHYF D--VHTFSQE

Stram_5962

CAVVGNGGIL LDNPNGYTID QHDAVFRINE GPTTGFEKHV GVKT--TVRL SYGPS--QWP TTGIVGIRLA TEICDCVNIY

GFSA--YHYY D--PHKYNKE

Guillar133

CAVVGSSGHL LGSGFGRDID VHDLVIRFND APAGEYESDV GSRT--THRV LHSSG--CVP SSGMIGIMWA LQSCLHVDTY

GFGR--YKYY G--GHNWNLE

Guillar145

CAVVGNAGHI FGSKLGAEID SHEAVMRFNL APTESFEEDV GGKT--TWWV TANFP--KIP TSGFLGILWA LQECDRVSTY

GF-------- ----------

Ocar_m4373

CALVGSSYNL MKHEFGAEID SHSLVARVND PPITGYEKHV GSRP--DISI SNLLI--GAA SCGLRSIVFM MHLCKEVHLY

GFGG--FKYY S--WHDFNRE

Azospirill

VAVVGNAPSE IGRKKGAEID AHDLVIRFNN FRLKGFEDDY GSKT--DIWV RGSGG--DFP TTGMVLIWIF RKFGNNVDFY

GFSF--THYF N--VHRLDKE

Leucothrix

VAVVGNSACL RNSNLGKKID QQDIVCRFNR YPRASHAPDT GQRI--DIWI TSPEV--APP SAGWTNYLIS LGLPKNIQLY

GFGK--YHII S--RHAWQDE

Idiomarina

IAVVGNHPKL KGSKLGDEID DADFVVRFNQ SSETNVE-DI GTKL--DLWV TAPAY--APP SAGVATLAIK SQTSATIELY

GFDL--EVYH H--RHNWSAE

Roseomonas

VAIVGNSPRL MENSHGAAID SHDLIIRVND GRIQGFEQHT GQRT--TLRY IGVPL--RPP RSGLSLLVDA MQAGKKVSIF

GMET--EHFY A--HCPMERE

Alteromona

VAVVGNSPKI LGKGLGQEID THDDVIRFNG AVISNYVNDV GSKT--TIVF VGIDL--HLK NGGIHYFAGP ASAMHNKDLE

GLGL--PR-- ----------

Rhizobium_

VAVVGNAPTL AGRKAGKWID SHDIVIRFND CRVRGFEADV GSKT--DILI SNPYA--IAL TTGTYGVSLV ANLLKNLSVT

GFTM--YHYW S--AHSPQTE

Sphingobiu

IAVIGNSPRL GQDRNGERID AHDVIIRVND GRTTGFEEFG GSRT--DIRF VGVPL--KPP RSGIVILSFF EKGV-PISLF

GFEI--EHYY K--HCPMEVE

Methylobac

IAIVGNAPKI LEREDGALID AHSTVIRIND GRSVGFEKHC GAKT--DIRF VGIPI--KPP RTGITSLLAP LFGKSKITLF

GFET--EHYY N--HCPMEFE

Salinispha

VALVGNAVSL AERDYGTAID AADVVVRCNR GILAEYSH-- GSRT--DWVV TGLPI--KIA STGMKAIDLA ASDCARLDIY

GFDF--SHSA S--DHDFDAE

Paracoccus

VALVGNARAL AQTGHGKRID NADLVIRINR APMPAAAH-- GTRT--DWLA LATRL--APP TTGMIALITW SQMA-RLELF

GFDF----FA S--PHDFSQE

Oceanibulb

VAVIGNSPSV LEKESGQFID SCDVVIRINN FQTAGFEKYI GSKT--DFAI FTAAS--SWP STGSVALQLA MDVFSKCDIY

GIDF--DHYF S--KHNSHLE

Paracoccus

VALVGNARAL SGGTQGAEID DHDRVVRINL APMPSPEH-- GRRT--DWLG LATRL--AQP TTGLIELLLR SELA-RLDLY

GFDF----FA S--PHDFTGE

Loktanella

VAIVGNSSVE RGRRKGAEID GHDIVIRFNL PKQQAFAIDY GSKT--DLIV INHSI--RRG TEGFFGFSMT DQIGDSPTSA

NYFR--RHNW K---------

Thioclava_

VALVGNARAL AEGQAGAEID AHDVVIRINR APMPSAEH-- GTRT--DWLA LATSL--AQP TTGLIEMLLR SDLA-RLDLY

GFDF----FA S--PHDFAAE

Thalassobi

VALVGNAKSL REKSLGGEID AHDVVIRLNR GPIMSTES-- GQKT--TVLA TSIPI--SRP TSGVMALHSV LDG-EQVTLF

GFDG----FA S--PHDFVSE

Helicobact

IAVVGNSPCE IGRNKGAEID SHDVVIRFNN FSLKDFIIDY GKKT--NIWV LTPAL--RVM SLGLIVICYL LDKVQNLTLY

GFSL--THYF D--IHKWDKE

Sinorhizob

VLVLGNAPSV LHRELGREID KFDVVIRINN FRIRGYEKHV GSKT--DYAL ISPAC--QWP STGIVAVQWA RDMHKRVHVH

GFDF--TRYF D--KHDFDRE

Prorocentr

VAVVGNAKSI DEHLFGSEID SHDVVIRINR GPILSAE-KS GSKT--TVIA SSIWV--SRP SSGVMVVA-A VAECNSISIF

GFDS--SN-- ---PHDFDAE

Pseudovibr

VAVIGNSKNL LNYQYGSEID AHDVVVRMNR GVTIPQKNCF GENT--NVWC YSTLK--ARP SVG-AMVTYL LAQCDSVDIY

GFDF----FY E--SHNFQRE

Agrobact_a

VLVLGNAPSV LERELGAEID KFDVVIRVNN FRIRGFEKHI GSKT--TYAL ISPAC--QWP STGIVAVQWA RDMHKSIYVH

GFDF--TRYF N--KHDFDRE

Sphingobiu

CAVVGNSSCE IGRGHGAQID AHDEVVRFNR FDTPPFDRDY GSRT--TVLV RVGND----P SSGFVHLLKA LRGTHREDFF

GFAF--AHYF E--MHQWDRE

Ensifer

VLVVGNAPTL LEREIGDKID KFDVVIRVNN FRTGGYEKFV GSKT--DYAL ISPAC--QWP STGIIAVQWA RDRHGKAADV

YVHG--DFYS D--KHDFDRE

Candidatus

IAIVGNSGCE IGLGRGNEID SHDIVIRFNN YCIDGYEQDY GTKT--DIWV RGSAG--KFP STGALTVWAT YLAKSNLNNV

DVYG--NNYS D--DHDFENE

Drer_ST6GN

CAVVSSSGQM LGGGRGPEID QQDCVIRMNV APTAGYEADV GNRT--SLRV VSHTS--AFL STGFFTMILA LEVCDSILVY

GMID--YHYY E--GHRFITE

Mmus_ST6GN

CAIVSNSGQM VGQKVGEEID HASCIWRMNN APTKGFEEDV GYMT--MVRV VSHTS--SYL STGWFTFILA MDACYSIHVY

GMIN--YHYY E--GHRFITE

Hsap_ST6GN

CVIVSSSSHL LGTKLGPEIE RAECTIRMND APTTGYSADV GNKT--TYRV VAHSS--SWL STGWFTMVIA VELCDHVHVY

GMVP--YHYY E--HHRFITE

Hsap_ST6GN

CALVTSSGHL LHSRQGSQID QTECVIRMND APTRGYGRDV GNRT--SLRV IAHSS--TWL STGWFTMTIA LELCDRINVY

GMVP--YHYY E--HHRFITE

Hsap_ST6GN

CAVVSSSGQM LGSGLGAEID SAECVFRMNQ APTVGFEADV GQRT--TLRV VSHTS--SFL STGWFTMILA LELCEEIVVY

GMVS--YHYF E--AHRFITE

Hsap_ST6GN

CAIVSNSGQM VGQKVGNEID RSSCIWRMNN APTKGYEEDV GRMT--MIRV VSHTS--SYL STGWFTFLLA MDACYGIHVY

GMIN--YHYY E--GHRFITE

Drer_ST6GN

CALVTSSSHV LGSQAGEEID RTQCVFRMNN APTSGHQSDV GNRT--TVRV VAHSS--SWL STGWFTMVIA IEMCDNIKVY

GMVP--YHYY K--HHRFITE

Trub_ST6GN

CALVTSSGRL IRSKRGQEID RSDCVIRMND APTVGHQRDV GQRT--SLRV IAHSS--SWL STGWFTMAIA LELCDRVDVF

GMVP--YHYY E--HHRFITE

Drer_ST6GN

CALVTSSGHM TGSGRGAEID ETECVIRMND APTRRYQKDV GQHT--NLRV IAHSS--SWL STGWFTMAIA IEMCDRINVY

GMVP--YHYY E--HHRFITE

Drer_ST6GN

CSVVSSSGQI LGREAGADID QSSCIWRMNN APTRGFERDV GHRT--DLRV VSHTS--SYL STGWFTLILA MDMCKEIRVY

GMIN--YHYY E--GHRFITE

Dsim_ST6GA

CAIVSSAGSL AGSKLGRFID THDIVMRFNH APTQGHEVDV GSKT--TIRV VNSQV--NPP SSGFIGLALL LPHCPQVDFV

EYVP--CHYY S--WHPLAAE

Hsap_ST6Ga

CAVVMSAGAI LNSSLGEEID SHDAVLRFNS APTRGYEKDV GNKT--TIRI INSQI--NPP SSGFIGILIM MSMCREVHVY

EYIP--CHYH E--YHPLLYE

Drer_ST6Ga

CAVVTSAGAM LHSGLGKEID SHDAVLRFNT APTVGYERDV GNKT--TIRI INSQI--NPP SSGFIGILLM MSLCEEVHVY

EYIP--CHYH E--YHPLLYE

Cmil_ST6Ga

CAVVASAGAI LNSSLGDEID SHDAVMRFNA APTKLYEQDV GSKT--TIRI LNSQI--NPP SSGFIGIVIM MALCDSINIY

EYIP--CHYH E--YHPLLYE

Cliv_ST6GA

CAVVSSAGSL KSSHLGQEID SHDAVLRFNG APIKGFQEDV GQKT--TIRL VNSQL--NPP SSGMLGIVIM MTLCDEVHVY

EFLP--CHYY Q--YHPLLFE

Hsap_ST6Ga

CAVVSSAGSL KSSQLGREID DHDAVLRFNG APTANFQQDV GTKT--TIRL MNSQL--NPP SSGMLGIIIM MTLCDQVDIY

EFLP--CYYY Q--YHPLLYE

Bmor_ST6GA

CAVVSSAGAL LGSRLGDFID SHDMVLRFNN APTDNYTDDV GSKT--TFRI LNSQV--NPP SSGFIGLWFS LNRCRRVRVF

EYVP--CHYY A--WHPLAQE

Bflo_ST6GA

CAAVMSSGAM LGSRLGKDID SHDAVLRYNS APTKRYQSDV GSKT--TLRL MNSQV--NVP SSGFMGIHLL LSLCDTVDVY

EYVP--CHYY G--YHPLRAE

Aaeg_ST6GA

CAVIASAGSL KGSRLGDFID DHDVVMRFNH APTKGFEVDV GSKT--TVRV VNSQV--NPP TSGFIGLGLL IPACRYIDMI

EYIP--CHYY D--WHPLAAE

Afor_ST6GN

CAIIGNGGVM NGSNKGEEID AHDYVFRVNV ALTKGFEKDV GSKT--SFYC FTMVT--HRP TTGAIMLLLA LHTCDEVNVY

GFGG--EHYY D--NHDNNAE

Lpol_ST6GA

CAIVSSSGSM YKSGLGKEID THDVVLRFND APTKGYEKDV GNKT--TVRI LNSQV--NPP SSGFLGLLLL LQHCNTVDIY

EYIP--CHYY D--WHPLASE

Phumcor_ST

CAIVSSAGSL LNSKLGHLID QHEIVVRFNH APTVTYEEDV GSKT--TVRL VNSQV--NPP SSGFLGLALL MPYCDTIDFF

EFVP--CHYF E--WHPLSAE

Pmar_ST6GA

CAVVSSAGAM LGSGLGREID AHDAVLRFNA APTEHFEKDV GTKT--TIRL VNSQI--NPP SSGFLGVVLM MWLCDEVNVY

EFLP--CHYH E--YHPLLYE

Skow_ST6GA

CAIVASAAYF KNATLGKEID SHDAVLRFND APTEGFEENV GRKT--TIRL MNTLI--TLP SSGIMGISLM QRMCDTVNVY

GYLI--CHYY D--WHPVEYE

Skow_ST6GA

CAIVSSSSFM TDSGLGEEID SHEAVMRFNN APTVGFERDV GNKT--TMRV INLDI--HVP SSGFTGIILM QQICDQVNVY

GFVT--LHYY S--WHPMVQE

Skow_ST6GA

CAVVLSSFYM NETGFGDEID SHEAVLRLND APTVGYEKSV GRKT--TIRL LNSKC--IVP SSGFTGLLMM LGLCDKVDVY

GFGI--CHYY D--WHPLQ-E

Skow_ST6GA

CAVIGSSYFL LGSKLGKEID SHDAVLRFND ALVTGFERDV GSKT--TIRL INSQM--IVP TTGFTGIHLM HHICESIDVY

GYVT--CHYY D--WHPVEAE

Isca_ST6GA

CALVSSAGSL LGSRLGNEIG AHQAVLRFND APTQGFEQDV GSRT--TVRL LNSQL--NPP SSGFLGLLLL LGLCRRLDAY

ELVP--CHYY E--WHPLAAE

Apis_ST6GA

CAIISNAATL RNSNLGYFID QHDLVLRFNN APTKGYEKDV GSKT--TIRI LNSQV--NPP SSGFLGLAML LPRCTVVNMF

EFIP--CHYY H--WHPLAAE

Tcas_ST6GA

CAIVASSGAL KDSNLGKKID AHDLVLRFNH APTKGFEPDV GRKT--TIRV LNSQV--NPL SSGFLGLGIL LPLCNFIDVF

EYVP--CHYY D--WHPLAAE

Acar_ST6Ga

CAVVSSAGSM KSSHLGEDID SHDAVLRFNG APIKGFQADV GEKT--TIRL VNSQL--NPP SSGMLGKLIM MNFCDEVDVY

EYLP--CHYY Q--YHPLLFE

Tgut_ST6Ga

CAVVSSAGSL KSSHLGQEID SHDAVLRFNG APVRGFQDDV GQKT--TIRL VNSQL--NPP SSGMPGIVLM MTLCDQVDVY

EFLP--CHYY Q--YHPLLFE

ocar_41732

CAVVSSASSL LRYEFGPLID SHDAVIRLNG APTIGYEKHV GSKT--SLRV INARV--ISS SSGFHAVLMA LFLCDSVSSF

EVAT--HSYY R--WHPLNLE

ocar_m1870

CAVVSSGPSM LKFKLGKEID SHDAIFRINM APTKSFEKYV GSKT--TIRV LNSKI--LSF SSGTFAVLSA IWLCEYVTSY

EIAS--SYYF D--FHPLPAE

ocar_m1265

CAVVSSAPSL LKYQMGNEID SHDAVIRINL APTKEFEKHV GKKT--TIRF INSGV--YTL SSGSQAFLMA LFMCESVTAY

ELAT--QYYF D--VHPLDIE

ocar_m3114

CSLVSSSGRI LGQSKGAEID GADCVLRMNI APVKGYEGDV GKRT--TARI LSQFS--GNV HLGQKTANYK KMLEKAIQLP

NIGF--YHYW D--AHRFMTE

ocar_m6388

CSLVGSSGHL LSEKAGEEID KADCIFRMNQ APVRGFEKDV GRRT--TVRV LNHVC--SWL TTGWFTFDLV LRACDQVSVY

GLPP--YHYF N--HHRFVTE

ocar_m1010

CALVGNSGHL LGSRCGQLIN KADCIFRMNE APVPGFKKDV GNRT--DVRI VNFHI--SWL STGWIAFDVV LRACDSVAVY

GFPP--YHYY G--HHRFADE

ocar_m1785

CAVVGNSDIL SGSNKGQLID SHEVVFRFNN YETKGYEKDV GNKT--TFQN LYRRI--NIP SSGIYTLLLA LYVCDEVSIF

GFGV--GSYY G--YHMWDTE

Cint_ST3Ga

CAVVGNSGNI LNSNYGNVID GHDFVIRMNK GPTYNYENDV GSKT--THRF MYPTT--RYP STGLLALIYA LHECDEVDVY

GFGA--HHYW E--VHDSAQE

D2KQ02_CAM

IIIAGNGPSL KNINYKRLPR EYD-VFRCNQ FYFED-KYYL GKKI--KAVF FNPGV--KRI TSGVYM-CAI AIALGYKTIY

LCGI--IYPF E--CHSKEYD

Q7BP25_CAM

ALVCGNGPSL KNIDYKRLPK QFD-VFRCNQ FYFED-RYFV GKDV--KYVF FNPFV--QRI TSGVYM-CAT AVALGYKSIY

ISGI--LYAF D--NHSMACD

Q9CLP3_Pas

VIVAGNGESL SQIDYRLLPK NYD-VFRCNQ FYFEE-RYFL GNKI--KAVF FTPGV--QRI TSGVYM-CAV AIAMGYTDIY

LTGI--NYAF D--YHSKDID

Q17WF9_ST3

LIIAGNGPSI KDLDYALFPK DFD-VFRCNQ FYFED-KYYL GREI--KGVF FNAHV--QHF TTGIMM-LLV AIQLGYKEIY

LCGI--GHFY E--MHDKNID

A6YD31_Lic

VIIAGNGTSL KSIDYSLLPK DYD-VFRCNQ FYFED-HYFL GKKI--KKVF FNYSV--KRI TSGVYM-CAV ATAMGYKDLY

```
LTGI--PYAF H--IHSMEYD
```

Associated references:

1. Kumar S, Stecher G, Tamura K: **MEGA7: Molecular Evolutionary Genetics Analysis Version 7.0 for Bigger Datasets**. *Mol Biol Evol* 2016, **33**(7):1870-1874.
