# Supplementary material for: Reconstruction of the sialylation pathway in the ancestor of eukaryotes
Source: Sci Rep. 2018 Feb 13;8:2946. doi: 10.1038/s41598-018-20920-1 (PMC5811610; doi:10.1038/s41598-018-20920-1)
Supplement: Supplementary file 1 — Supplemental Dataset 1, Dataset 3, Dataset 4 and Supplemental Figures [file 41598_2018_20920_MOESM1_ESM.zip › Supplemental data 3.pdf]

Scientific reports

Reconstruction of the sialylation pathway in the ancestor of eukaryotes

Daniel Petit[1], Elin Teppa[2], Ugo Cenci[3,4], Steven Ball[3,4] and Anne Harduin-Lepers[3,4,*]

[1]Laboratoire de Génétique Moléculaire Animale, UMR 1061 INRA, Université de Limoges Faculté des Sciences et Techniques, 123 avenue Albert Thomas, 87060 Limoges, France

[2]Bioinformatics Unit, Fundación Instituto Leloir -IIBBA CONICET, Av. Patricias Argentinas 435, C1405BWE, Buenos Aires, Argentina

[3]Univ. Lille, CNRS, UMR 8576 - UGSF - Unité de Glycobiologie Structurale et Fonctionnelle, F 59000 Lille, France

[4]UGSF, Bât. C9, Université de Lille - Sciences et Technologies, 59655, Villeneuve d'Ascq, France

[*]Corresponding author: anne.harduin@univ-lille1.fr

Correspondence: Anne Harduin-Lepers, Laboratoire de Glycobiologie Structurale et Fonctionnelle, CNRS UMR 8576, Université Lille Nord de France, Lille 1, 59655 Villeneuve d'Ascq, France. Phone: +33 320 3362 46 ; FAX : +33 320 43 65 55 ; E-mail : anne.harduin@univ-lille1.fr

Short title: Origin and transfer of sialic acid in LECA

Supplementary data 3:

1- **Full length sequences of sugar-nucleotide transporters SLC35A (1), Sialidases (2), CMAS (3), UDP-GlcNAc 2-epimerase (4), sialin SLC17A and NanT (5), NANS (6), NANP (7)**

2- **MSA of sugar nucleotide transporters SLC35A (1), Sialidases (2), CMAS (3), UDP-GlcNAc 2-epimerase (4), sialin (5), NANS (6), NANP (7)**

Supplementary data 3: full length sequences of sugar-nucleotide transporters SLC35A (1), Sialidases (2), CMAS (3), UDP-GlcNAc 2-epimerase (4), sialin SLC17A and NanT (5), NANS (6), NANP (7) and MSA of sugar nucleotide transporters SLC35A (1), Sialidases (2), CMAS (3), UDP-GlcNAc 2-epimerase (4), sialin (5), NANS (6), NANP (7)

1 - Nucleotide sugar transporter (NST); CMP-sialic acid transporter sequences (SLC35A1)

>Human_35A5 AAQ89353.1 SLC35A5 [Homo sapiens]

MEKQCCSHPVICSLSTMYTFLLGAIFIALSSSRILLVKYSANEENKYDYLPTTVNVCSELVKLVFCVLVSFCVIK
KDHQSRNLKYASWKEFSDFMKWSIPAFLYFLDNLIVFYVLSYLQPAMAVIFSNFSIITTALLFRIVLKRRLNWIQ
WASLLTLFLSIVALTAGTKTLQHNLAGRGFHHDAFFSPSNSCLLFRSECPRKDNCTAKEWTFPEAKWNTTARVFS
HIRLGMGHVLIIVQCFISSMANIYNEKILKEGNQLTESIFIQNSKLYFFGILFNGLTLGLQRSNRDQIKNCGFFY
GHSAFSVALIFVTAFQGLSVAFILKFLDNMFHVLMAQVTTVIITTVSVLVFDFRPSLEFFLEAPSVLLSIFIYNA
SKPQVPEYAPRQERIRDLSGNLWERSSGDGEELERLTKPKSDESDEDTF

>Human_35A4 Q96G79.1

MSVEDGGMPGLGRPRQARWTLMLLLSTAMYGAHAPLLALCHVDGRVPFRPSSAVLLTELTKLLLCAFSLLVGWQA
WPQGPPPWRQAAPFALSALLYGANNNLVIYLQRYMDPSTYQVLSNLKIGSTAVLYCLCLRHRLSVRQGLALLLLM
AAGACYAAGGLQVPGNTLPSPPPAAAASPMPLHITPLGLLLLILYCLISGLSSVYTELLMKRQRLPLALQNLFLY
TFGVLLNLGLHAGGGSGPGLLEGFSGWAALVVLSQALNGLLMSAVMKHGSSITRLFVVSCSLVVNAVLSAVLLRL
QLTAAFFLATLLIGLAMRLYYGSR

>Human_CMPSAt SLC35A1_HUMAN CMP-sialic acid transporter OS=Homo sapiens GN=SLC35A1

MAAPRDNVTLLFKLYCLAVMTLMAAVYTIALRYTRTSDKELYFSTTAVCITEVIKLLLSVGILAKETGSLGRFKA
SLRENVLGSPKELLKLSVPSLVYAVQNNMAFLALSNLDAAVYQVTYQLKIPCTALCTVLMLNRTLSKLQWVSVFM
LCAGVTLVQWKPAQATKVVVEQNPLLGFGAIAIAVLCSGFAGVYFEKVLKSSDTSLWVRNIQMYLSGIIVTLAGV
YLSDGAEIKEKGFFYGYTYYVWFVIFLASVGGLYTSVVVKYTDNIMKGFSAAAAIVLSTIASVMLFGLQITLTFA
LGTLLVCVSIYLYGLPRQDTTSIQQGETASKERVIGV

>SLC35A1_Danio NP_001122283.1 [Danio rerio]

MASESVSVLFKLYCLTVMTLIAATYTVALRYTRTVSTELYFSTTAVCLAEIIKLLLSLIMLVRETGDVGRCRAAL
VTHIFRSPKELLKLSVPSVVYAIQNNMAFVALSNLDAAVYQVTYQLKIPCTALCTVLMLNRSLSRLQWFSVFMLC
AGVTLVQWTPPHSTKVQVEQNPFLGFMAIAVAVLCSGFAGVYFEKVLKSSDTSLWVRNIQMYLSGIAVTLMGVYM
TDGARVLEKGFFYGYTPWVCLVVFLASVGGMYTSVVVKYTDNIMKGFSAAAAIVLSTVASVLLFGLQITLTFISG
ALLVCVSIYLYGLPKQDTTKVMKAGAEQDDTHKLISV

>SLC35A1_Callorhinchus XP_007903258.1 [Callorhinchus milii]

MAQENVTLLFKIYCLTVMTCVAATYTVLLRYTRTTLTEMYFATTAVCLTEVIKLILSLGIFAKETGDLGMTLQHL
RDHILRSPKEMLKLSVPSVVYAIQNNMAFVALSNLDAAVYQVTYQLKIPCTALCTVWMLNRSLSKIQWLSVFVLC
TGVTLVQWKPVETTKVQVEQNPVWGFTAIAIAVLCSGFAGVYFEKVLKSSDTSLWMRNIQMYISGIVITAAGILL
NDGAGVMEKGFFFGYTHWVYLVIFLASVGGLYTSVVVKYTDNIMKGFSAAAAIVISTIASIFLFGLQITSTFIAG
TILVIVSIYCYGLPKKNTTTLLPVEVEKSKETLNV

>Bflori1 XP_002607200.1 EamA-like transporter family BRAFLDRAFT_68003 [Branchiostoma floridae]

MAEGASVVFKLYCLAVLTVMAASYTVLMRYTRTVEGVRYYSTTTVFVTECAKMFFTLCILLKEHKGSIRKVTQEL
KGNIVXKPMEMLKMSVPSIVTYQMKIPCTALLSVMMLGRSLSSMQWIAVFVLTGGVILVQGIGGEAVSHTSGTEG
SYVVGLTALTIAVFCSGFAGVYFEKLLKGSDTSLWVRNVQMYTWGMLSAFLGVVMHDWQNVRENGFLYGYTPLVW

LVVLLGSGGGIYTSIVVKYTDNIMKGFAAAAAIVLSTVASIMFMGLVVGWMFVLGASLVIAAIFLYGLPKTNTEK
LPARKSGTAQNV

>Bflorid2 XP_002594507.1 UDPgal transp BRAFLDRAFT_59803 [Branchiostoma floridae]

MKYISLVILVVQNASLILTMRYARTMPGDMFFSTTAVVMAEVLKLVGCVLIIMCQYVGFRACTQHLYSELFGNPM
DSLKMAVPALVYTLQNNLAYVAISNLSAATFQVTYQLKIMTTALFSILMLGKSISRMQWVSLFLLFAGVSAVQLE
STGATSSGKATGEKVETEQNPLLGLIAVVVSCISSGFAGVFFEKVLKGSVASVWVRNIQLAFFSILLGLISMWTK
DGAAVSEKGFFYAYNWVTWMTICMQAFGGLLVAVVVKYADNILKGFATSFSIILSCIASVYLFSFHITLQFAFGA
TLVIFSIYLYGKPARPTTTNSDGMKGLPTKISETG

>Caenorh_elegans NP_493723.3 Nucleotide Sugar TransPorter family [Caenorhabditis elegans]

MNRANDTSSNLKLISLVVLIVQTTALVLTLRYSQTQKSEGPRYLSSTAVVCAEIIKLITCFFVIYRNNGYRFSGM
LNELNREIFASPQTRADSLKVAVPAIMYVIQNNLLFFALKKLDAATYQVTYQLKILTTAIFSVTMLGKSLHRYNW
MALILLTAGVALVQYPSGDSTTSKSTAAEHDASDNILGLGAVLAACFSSGFAGVYFEKILKTSKVSLWIRNIQLA
FFSVFGALLVCWLYDWQAISDDGFLRGYNGVIWIVVLLQAYGGLVIALVVKYADNILKGFAVSLSIILSSFTSWL
VLGDLTITTTFAIGATVVIFATFLYGHEPKSTPAEAHNA

>Caenorh_remanei XP_003094322.1 hypothetical protein CRE_08530 [Caenorhabditis remanei]

MNRANDSTSSNLKLISLVVLIVQTTALVLTLRYSQTQVSDGPRYLSSTAVVCAEIIKLITCIFVIYRNAGYRFSG
MLAEMNREIFATPQTRSDSLKVAVPAIMYVIQNNLLFFALKKLDAATYQVTYQLKILTTAIFSVTMLGKSLHRYN
WLALILLTAGVALVQYPSGDSPAKTSSVHDASDNILGLGAVLAACFSSGFAGVYFEKILKTSKVSLWIRNIQLAF
FSVFGSLFVCWLYDWQAISDDGFLRGYNKIIWIVVLLQAYGGLVIALVVKYADNILKGFAVSLSIILSSFTSWLV
LGDLTITTTFAIGATIVIFATFLYGHEPKKSPIAHNA

>Clunio_UDPGlut CRL04548.1 CLUMA_CG017620 putative UDP glu transp [Clunio marinus]

MGIQRINPNNLKYVSLLTLTVQNSAVSLCMRYAKTRDGDIFFSSTAVFLAEVVKLITCIGLVFMEEGTFLRLKAS
VHNAIIKNKVDTMKMTVPSLIYVLQNNLLYVSAANLDAATYQVTYQLKILTTAVFAVIMLRKKLIPTQWGSLLIL
VLGVALVQLSQGGETRIINVAQNRFLGFSAALGACCLSGFAGIYFEKMLKTSDLSVWMRNIQLSLLSLPLSLLMT
MINDYTAISNQGFFFGYDLFVWFLVLLQACGGLIVAVVVKYADNILKGFATSLAIVLSCIVSIYLFSFLLTLQFA
FGTILVIISVFLYGYDPNKNNKSTKKSSNHADEESLLDSEPQNV

>Ceratitis_UDPNAcT XP_012160926.1 UDP-N-acetylglucosamine transporter isoform X3 [Ceratitis capitata]

MSATSIPTTAMNIHVNANTLKYISLITLTLQNAILGLSMRYARTRPGDIFISSTAVLMSEIVKLFTCLVLVFNEE
GKDAQKFVHSLHKTIVANPVDTLKVCVPSLVYIVQNNLLYVSASHLDAATYQVTYQLKILTTAMFAVFILRRRLI
NTQWGALVLLIAGVVMVQLAQTVDDSVSSAPPTGPEQNRMLGLWAALGACFLSGFAGIYFEKILKGADISVWMRN
VQLSLLSIPFGILTCFINDGRQISNVGFFHGYDLFIWYLIFLQAGGGLIVAVVVKYADNILKGFATSLAIVVSCV
ASIYIFEFHLTIRFAVGAGLVIASIFLYGYDPKKSTVGIKQGSTGTGQTQLEDEEKLLPRV

>Apis_UDPGalt XP_012350252.1 UDP-galactose translocator [Apis florea]

MKSQQRTGQILKYVSLITLTLQNALVGLSMRYARTRSGDMFLSSTAVVMAEVVKFLTCLILVFIEEGNFLKFFDS
LKLIIIKQPIDTLKVSVPSLLYIIQNNLLYVSASNLDAATYQVTYQLKILTTAFFAVVILRRSLRNTQWGALILL
VIGVVLVQLAQSSDTSLPSGIEQNHLLGFSAALSACFLSGFAGIYFEKILKDSDISVWIRNIQLSLLSLPFGLIT
CFVNDGEMLQKQGFFFGYDLFICYLIILQAGGGLIVAMVVKYADNILKGFATSLAIIISCVASIYLFNFNLSFQF
SIGAILVILSIFMYSHQPKSTIVDKHSLTDKV

>Human_UDPNAct SLC35A3_Q9Y2D2_HUMAN UDP-N-acetylglucosamine transporter

MFANLKYVSLGILVFQTTSLVLTMRYSRTLKEEGPRYLSSTAVVVAELLKIMACILLVYKDSKCSLRALNRVLHD
EILNKPMETLKLAIPSGIYTLQNNLLYVALSNLDAATYQVTYQLKILTTALFSVSMLSKKLGVYQWLSLVILMTG
VAFVQWPSDSQLDSKELSAGSQFVGLMAVLTACFSSGFAGVYFEKILKETKQSVWIRNIQLGFFGSIFGLMGVYI
YDGELVSKNGFFQGYNRLTWIVVVLQALGGLVIAAVIKYADNILKGFATSLSIILSTLISYFWLQDFVPTSVFFL
GAILVITATFLYGYDPKPAGNPTKA

>Human_UDPgalt SLC35A2_P78381_HUMAN UDP-galactose translocator

MAAVGAGGSTAAPGPGAVSAGALEPGTASAAHRRLKYISLAVLVVQNASLILSIRYARTLPGDRFFATTAVVMAE
VLKGLTCLLLLFAQKRGNVKHLVLFLHEAVLVQYVDTLKLAVPSLIYTLQNNLQYVAISNLPAATFQVTYQLKIL
TTALFSVLMLNRSLSRLQWASLLLLFTGVAIVQAQQAGGGGPRPLDQNPGAGLAAVVASCLSSGFAGVYFEKILK

GSSGSVWLRNLQLGLFGTALGLVGLWWAEGTAVATRGFFFGYTPAVWGVVLNQAFGGLLVAVVVKYADNILKGFA
TSLSIVLSTVASIRLFGFHVDPLFALGAGLVIGAVYLYSLPRGAAKAIASASASASGPCVHQQPPGQPPPPQLSS
HRGDLITEPFLPKLLTKVKGS

>Exophiala XP_013311993.1 PV05_10130 [Exophiala xenobiotica]

MAPQDGVATIYGVPTKYASLLTLVVQNSSLVLVMRYSRILPGPRYLSSTAVVLSELLKCLICLTIHLREQRSQSQ
YSRLPTLSDDASTSSNQHAYSLRQLWTDIFGVKSGFLKLLVPAVLYTLQNNLQFVAASNLDAATFQVTYQCKILT
TALFAVLMLGQTLSAKKWLALVILTAGVACVQIPSSTTSTTKQPGNYMLGIIAVAVACLCSGFAGVYFEKVLKGG
HTASIWVRNIQLSVGCLAIALVGALMWDGQAIRQDGFFQGYNPIVVVTICIQAAGGLIVAMVIKYADNILKGFAT
SLSIILSTVASVFLFNFMPTIYFLLGSILVFSATYLYSMPDSPKATEVSTIEAEKGPVVNVEDYDSDNTSASHPA
PQPFRDESDLDSGDENLDATSIYEPDDGNKSRNYAPSLITTPTAEKKVFTELGHVG

>Capronia XP_007724679.1 A1O1_05604 [Capronia coronata CBS 617.96]

MAHQDAFSTICGVPTKYVSLFTLVLQNSTLVLVMRYSRILPGPRYLSSTAVVLSELIKCIICLSIHIRDQQTQSR
YSRLPTLSDDNVPTSTSSGYSLHQLWNDLFSLKSGFLKLLVPAVLYTLQNNLQFVAATNLDAATFQVTYQCKILT
TALFAVVMLGQSLSSRKWLALVILTAGVACVQIPSSTTPLHVHQGNYLIGISAVTIACICSGFAGVYFEKVLKGG
QHGSIWVRNIQLSVGCLGIALFGALVWDGKAIREGGFFQGYNAVVVATVCIQAAGGLIVAMVIKYADNILKGFAT
SVSIILSTVASVFLFNFVPTVYFLLGSVLVFVATYMYSMPGSPSVDASIPFDPSEKGPILVVENYDGEDGLDLKA
GPSSFSDESDLESGDEGSHIGSSIYAPDDGTHSRNFSPCLLSTSTMEKKGLSELDHAP

>Sugiyamaella XP_018735171.1 UDP-galactose transporter Gms1 [Sugiyamaella lignohabitans]

MSSAPTLFGVPLKYLSLVTLTVQNSSLILVMHYSRVMPGYTADSRYYASTAVLLNEIIKFVFCTIVCVYQQGFKS
AFEDVFSGDCWKLSIPAFLYTLQNTLQYVAVSNLDAATFQVTYQLKILTTAFFAVTILHKDLSKLQWLCLVLLTF
GIGLVQMPPEAFNGLVAFVIESATTAVGKGSPVAEGPGSTKPAGGSASKLGAKAAKVVREVAAAVVEKEEMNGLI
GFVAVSIACILSGLAGIYFEKVLKGSKKVSLWTRNVQLSMFSLIPALFLGVLAKDGSKIAENGFFHGYNNVVWAA
IWLQAIGGIVVALCVKFADNIAKNFATSISILISFVASVYFFDFAVHVNFLIGATVVVGVTYLYSLSPASARRPS
SSDEEYKPLPDQRSRSD

>Tuber XP_002837333.1 [Tuber melanosporum Mel28]

MPKTSANRYFTSTAVFLNEVLKLSISIWVAYLERRKVDGPKVPVAKSIKHLCGDVFRPDNWKLAIPACLYTLQNS
LQYIAVSNLDAATFQVTYQLKILTTALFSVLMLHRNLNAKKWASLIMLTVGIAIVQLPAAAKSIPDNPEMNRAIG
LIAVAVACTISGLAGVYFEKVLKGSNTTLWVRNVQLSFYSLFPAFFIGVVAKDGREILERGFFDGYNNVVWSAIG
FQAFGGIVVALCVNYADNIAKNFATSISILLSFIASIYCFDFEVTIGFMIGASIVLFATWLYSAPDGLIPKWKQE
YIPLEQADPKSDHDGKGHEGEEIVGKSSIDGLKRSD

>Monosiga XP_001749336.1 [Monosiga brevicollis MX1]

MAVDSLPGIRAGAIFVPYKYVALVLLMVQTTSSILVLRYSRTREGGAYLSTTAVVMAELFKLLGSAVLLNYERRE
SPLETIGYMYRELFINWVSSLKLSVPALLYTVQNNLLFVALSNLPAASYQVTYQLKILTTAIFSVIMLGRSLNMY
QWLSLVLLMGGVALVQMPSSSDEEDPTAIKPIGNQIVGLIAVLSACCSSGFAGVYFEKILKGTKQSLWLRNVQLG
LFGMVLGLIGVYANDGQAVAENGFFQNYDGITWTAISLQAFGGLIIAAVIKYADNILKGFANSISIILTGLISFI
MLADFQLTFMFAIGAFLVMGATFLYGHTPAPAASATPAKPNGLPK

>Salpingoeca XP_004990357.1 UDP-N-acetylglucosamine trpt [Salpingoeca rosetta]

MAQEPLPGIHMGAMFFPYRYIALLLLMVQTTSSILVLRYSRTVNGDGGHYISTTAVVMSECFKLVGSFFLLQRET
GLGLVQTYRHMYGEIMGNWKGTLKLSVPALLYTVQNNLLFIALSNLSAATYQVTYQLKILTTAVFSVTMLSKVIS
SRQWISLVLLMAGVALVQMPADDGSGDATMPEDANKNQFVGLVAVLSACCSSGFAGVYFEKILKGTKQSLWLRNI
QLSLFSIVLGLIGVVVNDGDRVAEGGFFQYYSTVTWIAISLQAFGGLIIAAVIKFADNILKGFANSISIILTGLL
SYLLLGDVRFTMYFAVGTMLVVASTFMYSHPSSQPAPKPTLPQTGHK>Nematostella XP_001626974.1 predicted protein [Nematostella vectensis]

MYVSSTAVIMAEVFKVATCLVILLVMQGGVLSWLRHLYDSIIGQPIDTLKLSVPALIYTIQNNLQYVAISNLDAA
TFQVTYQLKILTTALFSVLMLNKSLGRLQWLSLVMLFAGVSIVQLQSSSTKSSSTSQPNATMAPSANLATKQNAL
LGFGAVVMSSLCSGFAGVYFEKILKGTSGSVWLRNVQLGAYSTVIGLIGMQLNDGAKIAEKGFFQGYSSLVWSVI
CMQAFGGLLVAVVVKYADNILKGFATSFSIVLSCIVSIYLFAFHASLQFVVGAALVCTAIYLYSTPPQQQLPQKG
KLTPASNV

>Exaiptasia KXJ18975.1 UDP-N-acetylglucosamine transporter [Exaiptasia pallida]

MAVKLNPFVRAGSVNIPLKYVCLGVLVLQTSVLVLTMRYSRMVHEGPMYISSTAVVMAELFKVIACVFVIFHHCG
YRWRAFVNELNNDIMQKPLETFKLAVPSGLYTLQNNVLYIALSNLDAATYQVTYQLKILTTALFSVAMLNRKLNA
YKWFALVLLMAGVALVEWPTDGDKSAKKDLTASSKFVGLIAVLSACCSSGFAGVYFEKILKGTKASIWLRNIQLG
SFGIIFGLLGVLFNDGKAVQANGFFQGYNYITWMVIFLQAFGGLVVAAVVKYADNILKGFATSLSIILSSVVSYY
LLQDFNPSISFFVGVFFVLLATYLYGKPSPEPSQLFLPIKTSDNTSNI

>Entoamoeba XP_008858508.1 UDP-N-acetylglucosamine trp [Entamoeba nuttalli P19]

MNSSVVSLIFLVLLCCQTVIQSILGRYSRGVLHETYSIPSTIVFNEILKFLICLVMLKFVHKKENLLQHVIYLIK
TSLVASVPGCIYFIQNMLLYIILQNTQAAVYTVIIQLKVFTTAIFSVLFLGRKLSVAQWRALALLVTGVILVEIS
TNRYSSEKKNETENNLLGIVLSLVMACCSGFSGVYMEKILKNKTSGTEPLNIWERNIQLCVYGCGFALLSTFIFD
GKSILDNGFFGGWSYITILLIIIQGVGGIFVALVMTYADNIVKGFSIGCAIVLTTICSIFIFGTQVDTTFIIGAA
FVIISIANYNDKYVKAEN

>Reticulomyxa ETO13082.1 UDP-N-acetylglucosamine transporter [Reticulomyxa filosa]

MLQYTALQNLPAPVYAVLQQLKILSAAVCSYLILRESLASRQWRALFLLMAGGILIENHTFEIHASGNLSLTAND
PVKGTIAILTIVSLSGVAGVITQLLLKNKSTSGTSMAQLELSVWDRNAQLAFWSILIGVFSLILDYQWITDHKHI
FFGWTLNTWILVFLWSSGGILVALTIKYTDVIIKGFASAISLIVISVLGWLVLGDVLDLVFGIGAIVTVIATFNY
NDKDPVSTGSMIKKGSNNSANVSKNDADNEINDEDDVVYIDPKEAEKPTK

>Phaeodactylum XP_002179931.1 [Phaeodactylum tricornutum CCAP 1055/1]

MVGLVLLMRYSRTQHDDTQPLYLASTAVFLMEVMKLVICVGVIAVQTKSGVLHELYTHTIGSPLELLKLTVPSLL
YTVQNNLLYLALTNLDAATYQVCYQLKILTTALFSALLLQRKFSTMKWLSLVVLTIGVAIVQLSGSGDQHSEQDS
KAATDAVDDTNGTAAAHTRWVGLVAVLCAACTSGFSGVYFEKILKGSRTSLWIRNVQMGLSSIVIAYLTVYVKDA
EAIRTQGFWGGYNTLVWTVVTVQAVGGLIVATVVKYADNVLKVFATSFSIVVSCIVSAFLFDFHPSVSFLVGASL
VVTATVMYSSPETRTRKTRRRPVLPIHHRNNTATTKSRV

>Nannochloropsis XP_005853624.1 udp-n-acetylglucosamine trt [Nannochloropsis gaditana

MHSIFGGMKRADTCKYVALLVLVLQNSAVVLTVRYSRMRKDVMYISSTAVVMSEIVKLVVASFLVGMEEGGLCGL
GSKLYHDIVLKPADFAKLLVPAFLFTIQNNLLFVALSNLDAASFQVLYQLKILTTAVFSVVLLNRQLTCRQWLSL
LVLIVGVSLVQTSGLKDGSTSSTAGRNGSTSLGFVCVLLASCSSGFAGTYFEKVLKDSEISVWVRNVELALIGIP
VGVFGVWYTDGAAVREAGFFSGYSPLVWSVVGLQAVGGIAIALVVKYADSVLKNFSTSVSIVVSCLVSYVVFGET
DLSPQFLAGVSLVMYSTFLYGTSSSGMCCLSEGGSFTPNKGVARKESNKVWSVACCAKQAASTRDDPLASVPFLE
SADKENRQRKDCESHGNKSNRVSASVA

>Aureococcus XP_009040768.1 AURANDRAFT_32438 [Aureococcus anophagefferens]

MESSLLRHGSLALLVAQDTALVLLMRYSRQRSGSMYISSTAVCSMEVMKLSVCFLMLLCGEAHGSFGMLVFMIRK
EVLGRPKEVAKLALPALLYLIQNNLLYFALSHLQATPYKVTYNLKILTSAFFSVTLLGQRLGRRRWISLVVLFLG
VTIVQTDNPKNELSRHHSGLGSQTLGFVAVGGAAITSGFSGVYQQRILQSCKTDMWIRNVQMGVTSVTLGFLCTF
LKDRQAIADGGFFQGYSRLVWVVVSLQALGGLNVAFILKYADNILKGFAAAFSTIASCIIEMVLFQFRPSPLFLF
GSALINIAAYFYNTPATKRPTKCDASALHSV

>Chrysochromulina1 KOO26117.1 udp-galactose translocator [Chrysochromulina sp. CCMP291]

MLSMRYSKMPSQPKYLSTTAVVSAEVVKIVLSFAVLFSQMGTPAVGFVWRAVFVAWKDTLLVGVPAFIYLVQNNL
LYVATTHLDAATCQVAYQLKLLTTAFFTVTMLKRHISNRRWAALGLLFVGVVMVQAPAGSSGAPAKLGQSPVLGM
AAVFGACFLSGLAGVWLERIIKQTADVPIWVRNIQLGVVSLALGLGSVALLDGAAVAERGFFQGYTWLTASVVLQ
VSAGGLLVGLIMKYADNLLKGFATSLSIILSSVVSTFIPAFGFEPGLAFFCGSALVIAATIIYSSPAAVIAVGDV
GIV

>Emliania1 XP_005764129.1 EMIHUDRAFT_459747 [Emiliania huxleyi CCMP1516]

MQKDAAGAERASLVSASETSSEQAVSKSTKTALLLKWGSLAVLMVQNSSAFVVTRYTRQGKPNELYLTSVVVLMV
ELIKMAICLLLLLRDARGSVRELCAELQHHLWVERRTTLLLGVPALCYAMQNNLVFVAISNLSAAAAQVLYQLKT
ISTAFFTVLLLRRSFRAAQWVSFLLLMAGVALVQSEDGKSSSAPTGAAPWLGVSAALAAASISGFAGVFLEKMFT
SGGTSLWMRNVQLGLFAIPLQCIAIAQVDGQRVMRRGLVQGFHTSTWVVVAVQVVGALCTAVVIKFAGNVLKTFA
TVLALLLTCGSSMVLFDFHPTQLFFEGIGLVCVSVWLYARPGDVSDVLGLCRRKESSPRSAPLRHIPDDVSEEEL
PPKDGQIVV

>Emiliania2 XP_005789116.1 EMIHUDRAFT_62969 [Emiliania huxleyi CCMP1516]

MVSFTGWVALATFVAQNTAAALLMRYAKTRMEPYNSAVAVLLQEVALKLPISVVLFAVECGGFGQMVQKIRRDFS
ERPLEWAQLSVPALLYLVTNISVYVGYDNLEAALGMVMYQSKIVFTAIFSVTLLNKKISVNQWCGGGAPARALSP
GRRHGGGRSLAAAAEKNRVVGMGAFLLAAVCTSFASVYFEKMIKSDSKPSLWLRNLQLAMYSGVIAVAGLLINDY
DEIARQGPLHGFGFWTWCAVLNNALGGLLVAVIIKYADNILRSFAQGLAIVSGAVGSYLLFDFQITAQFMLGVVL
VIGAVFLYGAQASTPTELCEQLALSCLASVAPSRGDSALSDASADAPLKPAAYASVQPTEPHTADEVEEP

>Chrysochromulina2 KOO28939.1 isoform a [Chrysochromulina sp. CCMP291]

MKLYNSESSDKERLLESSSSSNAQRSEERLGTLLKYGSLGFLILQNSSHVLLLRYSRVVPGECSQYVVSVAVLFA
EICKLVFCVFVLCFTEGGPLSAFVVLDRDIWQRKMDTIKVSVPALCYTLQNNLQFVAATHLGAELLQLLYQTKTL
STAIFGVVILAKQLRCNQWCALTVLVAGVVLAQSSQRKPGESLYKGDAAIGVMAALGVSICSGFASVYLERILKG
DKTSIWVRNVQLCIFSIPLQLLAVYRTPGDWDKVQTQGWMVGFCPSTWAVVFMFAFGGLLVAIVIRFADNNLKNL
AMAVAILVSCVASIPLFGFEPNGTFAAGAFFVILSIFLYAWTPRSATAPSNYLPLPSSK

>Guillardia XP_005839607.1 GUITHDRAFT_101787 [Guillardia theta CCMP2712]

MHSTHEYKQVPLQEMNVKKDDLISNDVSDIEVANGSAHDHETQKDNAKAMASNKTNQLIKAAALVGLVFQNTSLI
LFMKQASITPSEDGKKALTTTVVVMVEFFKITACILEIAYRRRKSGGLISEIREEIVGKPRETMMLLVPAFMYLA
QNNLLFIAVANLEAVVYQVIAQLKILTTAGFSILILERKLTIQQWSSLVLLTIGAAVVQVDNSSPGQVAKKTEAN
LSSTIGLACALLAQCTSGFAGVFCEKMLKGGSSNMSVRNIQLGVPGFVFGIAGVLLTDYTKVTTGGFFQGYTYLT
WIVICLHSIGGLLVTVIMKYADNIAKTIAIGISLVVSTAVSMYIFDFVLTTNFCIGGSAVIFASFMYSSNLKMCP
APKPVN

>Bathycoccus1 XP_007515605.1[Bathycoccus prasinos]

MEDWGRKSVVTLALTVLTSSQGLLIAASKANGVKYDYAVTSANCTVETTKMLMSLLALVKIWRTVGVNEDNRIST
SWSELWVYPIPAALYLVKNLLQYYVFLYVDAPSYQILKNLNIISTGILYRIFLKKILSGVQWSALILLALGCTIA
QLTSGSDQVLSTPFMGLMMAIVMAILSGAAGVYTELIMKKQPKRNVNAQNVYLYLFGVIFNMVAIFLYDYDAVFG
RGYFYGYNAIVCTMILNHSLSGIAVSLVMKYADNIVKVYSTSVAMILTTLVSIPLFGFQLTLPFVLGTSVVSVAV
YLHYQSKDTPQPPGALTK

>Bathycoccus2 XP_007511207.1 CMP-sialic acid transporter [Bathycoccus prasinos]

MSFPGRAQELFGKNDASSKKNQTKRLDSFYYEEGDDGVDNSNKNNNNINNNVNNNTKKNRHKLAALFLVALTLEN
TASMLARRYAVGILQLDFSKNVVLCVNEFLKLLFSLGMKYRKTDIKSFARLKTHIFERVVKTATPMLVPAFVYLV
VNLISYPSLQRVDASVFTAISNLKVLATAIFAQILLNSRISNRVWRTLTQLVLGVTLISWESSPNNPVIHKRVHQ
NWYEHISDMFDLSYAFGVFLALVQTMLSGFGSVYFEKVLKKRTKEDEEENLGKKLDVESPSSASLAKSSSPFSSS
TELDVWDRNIQLALCSILIYVPISIYETKGNLFQGWTFLVIFIAALHALGGILVALSVLYSSSVTKTVAVCAALV
LTTVFGHILFFEPLNGPILLGCAMVIISVWAYKDDAYVEAKLRSAGFVV

>Bathycoccus3 XP_007511683.1[Bathycoccus prasinos]

MAKSSSISGMRKLVVAATLTVFTCSQGLLMEASKVRGKYPYNSAVVPLLSELVKLILSILLLRRARARDPAGTIM
TTDVKSVMLFPIPSIIYVMHNNVQFYTMAYVDAATYQILGNLKIVTTGILFRFALGRLMTRTQWIALLLLTVGAT
VSQISGCKGETLSAPMAGYALGVLSACLSATAGVYTEFLLKKNNDNLYWQNVQLYAFGVVFNGLRLTWDDFFGEN
SGGNWLFDCTNGFTAITWLIVINFSFSGLFVSWLQKFADTIVKVYATSSAMLLTALLSVSFFGLEPSLQLFLGIT
IACCSLVLYFMPPDLPKEKGMGVLPVTSGGGVNGDGNTSGSAYGGGATPRGGGSGGDKLN

>Bathycoccus4 XP_007509185.1 [Bathycoccus prasinos]

MTAGYYKLMMAATLTLATSSQGLLTTASKSNGEYRYNFATVPFLAEVLKLVVSSLLLHRQFLIDPKGTHITRDWK
SALLYPIPSIIYLIHNNVQFLTLQYVDPSTYQILGNLKIVTTGLLFRIILKRHLNRLQWIALALLMIGATISQIS
CDKGTTLAAPLMGYVLGIISACLSALAGVYTEKLMKMNNDNLYWQNIQLYGFGVIFNGLRLFFDDVNVGYSNGIS
LWPRVVTRGYNIITWFVVFNLAFTGLLVSWIMKYADTIVKVYSTSMAMLVTMLFSIILFDISPNLQLLLGILTSS
ISLRLYYFDTAELHPDITNKNSKLRDRI

>Anthurium JAT49509.1 UDP-galactose transporter [Anthurium amnicola]

MANGATILGIPLKYVSLFTLCIQNSTLVIVMRYSRLDKENLYYTSTAVFLSELTKLIICLYVAGRNQIRETGRLN
IPELSSQIFAPDAWKLMIPAALYTIQNNLQYIAVSMLDAATFQVTYQLKILTTALCSVIMLKTSLSGIKWFSLVL
LTFGVALVQMPSGDSVNSKDDDDVSMEKIVGLGAVAVACVISGIAGVYFEKVLKNSQTSVWIRNVQLSFFSLFPA
LIMGVWWKDGSGVWENGFFYNYNYVVVAAIACQAVGGIIVAMVVKYADNILKGFATSISIILSFLASVYLFEFIV
TSTFLMGATLVLIATYMYSKPDPPKQETLSNNKEKGEDDGYGYDYAPANNVGNGYTSVNGSESQV

>Oryza XP_015645198.1 CMP-sialic acid transporter 2 [Oryza sativa Japonica Group]

MEYRRVKDQESYDVVSQKDIESPGERSLSSTSATSSLSTAGASKGKNSWKLKSIVTLALTLLTSSQAILIVWSKR
AGKYEYSVTTANFSVEALKCLLSLIALYRTWNSQGVTEDNRLSTSFDEVSVYPIPAILYMVKNLLQYYIFAYVDA
PAYQILKNLNIISTGVLYRIILKKKLSEIQWAAFILLCAGCTTAQLNPSSDHVLQTPIQGWVMAIVMALLSGFAG
VYTEAIIKKRPSRNINVQNFWLYIFGMLFNLVAICVQDFDAVMNKGFFHGYSFITVLMILNHALSGIAVSMVMKY
ADNIVKVYSTSVAMLLTAVVSVFLFGFHLSLAFFLGSTVVSVSVYLHSVGKPQPQK

>Eleaeis XP_019704318.1 CMP-sialic acid transporter 2 [Elaeis guineensis]

MENYEDVSQNDIESLHGKPLSGVISNSTPSSLGGSTKSSWKLKSIVTLALTLLTSSQAILIVWSKRAGKYEYSVT
TANFSVEALKCALSLVALAKIWRSQGVTEDNKLSTSFDEVSVYPIPAILYLIKNLLQYYIFAYVDAPAYQILKNL
NIISTGVLYRIILKKKLSEIQWAAFILLCAGCTTAQLNPSSDHVLQTPFQGWVMAIIMALLSGFAGVYTEAIIKK
RPSRNINVQNFWLYIFGMIFNLIAIVVQDYDEVMNKGFFHGYSFITVCMILNHALSGIAVSMVMKYADNIVKVYS
TSVAMLLTAVVSVFLFGFHLSLAFFLGSTVVSVSIYLHSIGKPQPQK

>Zea ONM58952.1 CMP-sialic acid transporter 4 [Zea mays]

MEHRRVKDQESDGVSQKDIENFDKISLSSNTSTSSLSTAGGPKDSSKLKSIVTLALTLLTSSQAILIVWSKRAGK
YDYSVTTANFSVETLKCLLSLVALSRIWNSQGVTEDNRLITSFDEVRVYPIPAMLYLVKNLLQYYIFEYVDAPAY
QILKNLNIISTGVLYRIILKKKLSQIQWAAFILLCAGCTTAQLSPSSDHVLQTPIQGWVMAIVMALLSGFAGVYT
EVIIKKNPSRNINAQNFWLYIFGMLFNLVAICVQDFDAVMNKGFFHGYSFITVLMILNHALSGIAVSMVMKYANN
IIKVYSTSVAMLLTATVSVFLFGFHLSLAFLLGSTVVSVSVYLHSVGKLQQRN

>Brachypodium XP_003562852.1 CMP-sialic acid transporter 2 [Brachypodium distachyon]

MEYRRVKDQESYDVISQKDIESPDGRTLSSTTATSTLGAAGGLKGKQSWKQKSVVTIALTLLTSSQAILIVWSKR
AGKYEYSVTTANFSVEALKCLLSLLALYRTWNSQGVTEDNRLSTSFDEVSVYPIPAILYMVKNLLQYYIFAYVDA
PAYQILKNLNIISTGVLYRIILNKKLSEIQWAAFILLCAGCTTAQLNPSSDHVLQTPIQGWMMAIVMALLSGFAG
VYTEAIIKKRPSRNINVQNFWLYIFGMLFNLVAICVQDFDAVMNKGFFHGYSFITFLMILNHALSGIAVSVVMKY
ADNIVKVYSTSVAMLLTAIISVFLFGFHLSLAFFLGSTVVSVSVYLHSVGKPQQQK

>Vitis AFN67104.1 CMP-sialic acid transporter-like protein [Vitis vinifera]

MEYRKLKDLDKDDGAVGDDIESLRGKALSGAHTNNMATLGGHSIDRTKWKRKSIVTLALTVLTSSQAILIVWSKR
AGKYEYSVTTANFLVETLKCALSLAALVRIWGNEGVTDDNRLSSSLDEVIVFPIPAALYLVKNLLQYYIFAYVDA
PGYQILKNLNIISTGVLYRIILKKKLSEIQWAAFILLCAGCTTAQLNPSSDHVLQAPFQGWMMAIVMALLSGFAG
VYTEAIIKKRPSRNINVQNFWLYVFGMVFNAVAIVIQDFDAVMNKGFFHGYSLITVLMIVNHALSGIAVSMVMKY
ADNIVKVYSTSVAMLLTALVSVFLFGFHLSLAFFLGSTVVSVSIYLHSIGKLQR

>Hydra1 vulgaris_scaf38568_Ctg24371 ACZU01024371.1

EVKSFASWMRYLYNNIIADPLSTFKVAIPSFIYVLQNNLQFIAISNLDAATFQVTYQLKILTTALFSVLMLNKSL
TKGQWFSLFLLFVGVALVQFQPNQVNNSLTSQNPIVGLTAVVVSSLCSGFAGVYFEKILKGSGNVSIWLRNIQLG
IFGALIGAVGMIANDGTKIKQNGLLFGYSAIVWFVIFMQAFGGLLVAVVVKYADNILKGFATSFAILVSCIVSIY
AFNFVLSLEFVAGSI LVIVAIYIYSLPQNK

>Nematos_sc43 Ctg6974 ABAV01006974.1Length: 1356

FQITNQLKIMTTALFSILLLNKSISRKRWFYLLMLMIGVAVVEIELHRKIAAKMNKKEADAASKSFLIGFLSVLA
ASVISGFAGVFLEKIVKHKSTSLWIMNVHLYSWGVCLGVLGVVLKDGYQISQLGFFYGYDSVVWTVVALASAGGI
LVSLVLKYASTITKGFATSCAIVLSSLASVIIFGFDPSIYFILGAFLVVFAVILYGI

>Emilh_sc1_Ctg98 AHAL01000098.1Length: 28401

VITLLARESRIPVAGRPMYLGAAAVLVSECIKLPVCLAVISRQLGGPRQMLADVRQRVVVGWRDTLAMGLPALLF
GLQNMLFFVSVSNLSATSYQLWSQSKTLTTALFYVLYLGGALRRLQWLSLSLLCAGVGLVQASDSVSAAAGAASA
GRPLLGISAVLASSLLSGFANIYLEKKMKASEASLWVRNVQLAVFGIPQAALPSPSAERALRDREPPRRHRRTLA
ALAASGPFVGFTPTVWSVVWLRALGGLLVAAVIKYADNIVKTYATAVAIVLTCVVSSVLDGSRPTRAFLQGMSLV
LASVLLYNRKPKPSPPPLDDGEAAARRQAGRL

>Bathy_ct342 FQSR01000342.1Length: 8558

MMAVVARISGMRKLAVAATLTIFTCSQGLLMEASKVDGKYPYNSAVVPLLSELVKLILSVMLLRRAKTRDPKGTI
MTMDLKSVMLFPIPSIIYVMHNNVQFYTMAYVDAATYQILGNLKIVTTGILFRFALGRMMTRTQWIALLLLTVGA
TVSQISGCGDETLSAPVVGYMLGILSACLSATAGVYTEFLLKKNNDNLYWQNVQLYAFGVVFNALRLTWDDFFGE
NSGGNWFLDCTNGFTAITWLIVINFSFSGLFVSWLQKFADTIVKVYATSSAMLLTALLSVSFFGLEPSLQLFLGI
TIACCSLVLYFMPPDLPKEKNSVLPVTSAGHGGGSTVGGSGYGGGATPRGSGGGDGDKLN

>Guil_sc140_Ctg3497 AEIE01003497.1 Length: 2412

VVVSECLKVLACVFILAYHHREKVGQVIWQEAIVNWKDTLKLVRPSSCSILILEVFLDSKVGQVSVPAFVYMIQN
NLLYVATSNLDAATCQVTYQFKILTTALFTVTMLNRTILPLKWLSLVILVIGIALVQLPNIGAGSAFNIAASGNP
ALGLSAVVAACFMSGFAGVYFEKMLKGTPTSVWMRNIQMGTIGGILALAAVFIKDGQAVLSAGFFQGWNLFVWGV
VTQLGGLILPLVVRYPNNILKGFATSLVRFKTPSS

>Emil_sc187_Ctg5084 AHAL01005084.1Length: 38370

MFKLRNVVLVLLVAQTTSIVLLMRYSRTVVRPLDAPMYLASAAVFLAEAAKLPCCLYMVARVGGGCRGFVELLQS
EVLNQPGETLKCAVPALAYTVQGNLLFAALSNLDPPTYQITYQTKTLFTAVFSRMMLGRRLAHSQWLALFLLFLG
TVLVSDLCEPHPGRPRPLATRRDLGAAVLSSLSSVYFERMLKKPARSAMADRAGLWLRNVQLQLRSYGLLHGFDG
VVWLVVLLNGCGGLLVAATMKYADNIVKCFAAALAIITGMLLSVPLFNVPLSPIFLVGAASTVSATMLY

>Diplonema_LMZG01004873.1 Diplonema papillatum LMZG01004873.1

KYGGSTGLKFGPLGWLSLGCLVIQNSALTLTMRASRLSGGSTGPMYLATSAVVVCELIKLVLSFIMLFLEKGSWS
GMAGAINTDILTTPSQNFYLLIPSALYTIQNNLQYFAASNLDPASFQVLYQMKLVTTALLSVVMLKRSLSPTQWF
SVFILTAGIAMVQTSEAPVTTAAKQIKNSDENHLLGFIAVLAACCTSAGAGVYLEKLVKQTKPSVWIRNIQLAVF
GLIVGCLGCFMKDREAVAKGGFLQ

GFSPIVWAVVLLQSLGGILIAVVVKYADNIIKGFATGLSIICSCLASVFLFNFAVTPQYLAGASTVIVCQLPRCP
SRV

>Bodo saltans scaf448 CYKH01001645.1

MAALSLIVLVLQNSTLVLLTRYSRFSQPPGEMYHTSTLVLNQEIMKMVFCLVIFYIERKQELPHCNIAHELSSIV
FHRETLKLVVPAGLFTLQNFLIFVALSHLDAMTFQVLSQTKLLSAAVFSVWLLDRRLNRWQWISLVLLTLGVYFS
QQTAQRESFTKDSGNHAGGGAGGHQAQQLQHQQRYQSFFLGAVACIISGLSSSFAGVYFEKVVKTTSPSLAVRNI
HLSLFGIPFALLSLLVLDIIPTWWNPHLPRFRFWQGYNGMTWCLVFVHAFGGLLVAVVVKYADNILKGFATGVAV
IVSGAFAALFWGYEPSMLFVVGCVLVTTSSMVYHKKDNAPSHPIRSSRHNSEKD

>Diplonema ctg13729 LMZG01001553.1

MAENDEKQPGKGVSEVKQAHTRSPATSGAPEVPEKVRTDPTRVTVSICGFSINTGYLSLLVLVVQNAALVLLTRY
SRSQQKGELYRSSSLILNQELLKLAACLGYMLWENRKTTTRFADQLYHAVFCLETVQLGVPAVLFTFQNYLLFVS
LSHLDAMTFQLLSQVKLLLAALFSVWILDRYLTCVQWTSVVVLTFGIVLASQVPGTEKVAPQIHQNVLLGAFSCV
ASGTSSAFASVYFEKILKGTPPSIAVRNVQLGIFSIILAVGSMLVIDGVPGRSFVYFGGYSALTWVLVLVHAGGG
ILVAVVVKYADNILKGFATGVAIIVSGLYAAVAWGFAPSLHFIVGACIVTAGTCVYNIPDPNVPAIQVFRRPFPE
GGEQRRVAKSRVATDISPAF

>Trypano_grayi JMRU01000019.1

MRYHTSTLVLNQEVVKMVLCIIIFAVENRLALRAPLLEIRDTSAVSPSVSTLLWRVVFHKETLKISVPAALFTLQ
NYLIFVGLANLDATTFQVWSQTKLLSAAVLSVLMLGRRLSMMQWVSLFVLTLGVLLTQQQDLKKTETIPTSEEHN
SQRPLLGIAACVLSGISSSYAGVYFEKVVKTTAPSLAVRNIHLSLFGIPFAAISMFFLDILPTWRNPAEQEQTFE
YWRGYDQWLTLCLVFIHALGGLLVAIAVKYADNIVKGFATGVAVVVSGVMSFSIWGVVPSMAFLWGCALITLATV
VYHKYDIRVQSRDFHRRLRGVMRTIDETLAVDSRRAHTNSLPRARSNSPERRNSFSLVPHLPSWTERRSEQRRHE
LQAEREDAQRLLDSLRYAREKREPLRDETTANRLSGPDVSVGPLCPSCSERQCQCEASTPSPMRSPPLLLIPTID
QHQQRNQ

>Sycon1 sctid19399| scgid3055| UDP-galactose translocator Length = 1278 Sycon ciliatum

LELKYVSLIILTFQNAILILSVRYTRTLEGKMYISTTAVVSAEVMKVAFCICVLISQRRGVFRFLALLNEQVFQN
WQDTLKLSIPAILYTLQNNLQYVAVSNLNAATFQVTYQLKILTTAIVSVILLGKVLTRIQWTSLVILFVGVCMVQ
VQLQNPKEPSVTVAPTPMQDPLRAATAGSSAPPSKQNPILGLIAVIISAFSSAFAGVYFEKILKGSAQVSVWMRN
IQLGVFGTVIGLVGMLMNDGTAVAKNGFFYGYTGWVWFVIFMQAFGGLLVAVVVKYADNILKGFATSLAIILSTV
ASLYLFPDFRVSFTFLIGTSLVISAVYLYG

>Sycon2 sctid78829| scgid22258| UDP-NAc transporter Length = 1008 Sycon ciliatum

KYLSLIVLVLQTTALVLLMRYSRAVGTGPMYLASTAVVMAEVFKLVSSLLLFWWELARESYRFHSPIVFLTHLRT
VLFSQPFELLKLAVPALLYTIQNNLLYVALSNLDAATYQVTYQLKIMTTAIFSVLMLGKQLDVKKWTALVMLMFG
VACVQMPSSSSKSYSATHTVVGSS-
VVGLVAVLSACCSSGFAGVYFEKILKGTKPSLWLRNFQLAFFSILLGFGGVVL-
DRKVVLSEGFFHGYSPLVWTVVILQGIGGLVVAAVIKYADNILKGFATSVSLIFSAVISFIFLDDLELTSLFVIG
ASLVIAATMLYSQPEKKSDNVHPPQPAS

>Sycon3 sctid88776| scgid31310| CMP-sialic acid transporter 4 Length = 1053 Sycon ciliatum

KASAVIFISYMALFINQGLLVTASKRKDGSFAYNATTVVLMTEVVKLGMAIFLHLREATVKETISTIRQNLQVFA
LYFVPAALYTIYNNLVFVNLKHYDPTTYFILLQLRVVATGIIFQVLFGKQLTRIQWSSLVLLMIGCAVKNVRFWS
PAVGPTTAALSNNTSASHNPVVPRLDLHLLYLLFQVFASCFAGVYNEYLIKSNGSISVPLMLQNVFMYVDSIVAN
IVALALTQSLGDAVSSHNVHALLDWNVIAIILNNASVGIVTSLFLRSLNSILKTFASGLELVFTAILAWIFFGIR
VGVSTWLAIFIVSSAIYLY

>Amphimedon1 XP_019849718.1 UDP-galactose translocator-like [Amphimedon queenslandica]

MNKEKAGGLKYASLFILTIQNALLILSMRYSRIQVGEMYISTTAVVLSETLKLFTCVVILFISEKKGLIEFTAYL
FQSVIVNWRDTLKLSVPALVYMVQNNLQYIAVSNLDPAVFQVTYQLKILTTAVFSVIMLQKTLTRTQWGSLFLLF
IGVTLVQLKLASSNESENENNSQIVGLLAVIVSCLSSGFAGVYVEKMIKGGGASLWMRNIQLSLFGSLTAVLGML
MNDGGAVMSLGFFYGYNFLVFFVVFQQALGGLIVSVVMKYADNILKGFSTSLSIIISCVVSVFVFSFVISTYFVI
GCSLVLIAIYLYGRYQPTSPSLPQIVSSPSAPNVAAGN

>Amphimedon2 XP_003383398.1 UDP-N-acetylglucosamine trp-like [Amphimedon queenslandica] ARNm

MSGVNQKTETMKSAGGGVGVNLKYLSLIVLVVQNTSMVLVMRYSRTMEGPRYLSSTAVVLSEITKFVTCFFLVLN
GNGWQIGITLRELKTEIIDKYIETLKVCIPSFLYTVQNNLLYVALSNLDAATFQVTYQLKILTTAIFAVTLLRKQ
ILKSQWLALGMLTLAVALVQWPSGGSESSTNTNSTNSMKLVGLVAVLLACVSSGFSGVYFEKMLKGSETSIWIRN
IQLGILGLVFGLMAVFVTDYNKVMKDGFFQGYNIVVWTVIALQALGGLIVATVIKYADNILKGFATAVSIVVSSV
LSYFFLGDFDPTIKFGIGTVLVIGATFLYSYQPPKKPEPILPTTQQEHSDKIENKV

>Acinetobacter WP_081408536.1 EamA family transporter O-acetyl-serine/cysteine export gene [Acinetobacter sp. NCu2D-2]

MSPRIQGYAFVLITMCIWGGFTLTARLNALWNISAWDIVALRFSLAFLILMPILLYRKEASFLLRKEAFILAMIG
GVIYCLFAYSAFHYAPTAHAAIFLNGCIPLCTAIVAYFLLNQAFDKHTWASLVIMIIAICLMSILMYRETGIPFG
MGDVLFFISAILWGMFTVLLRKYQLSAWQAMSGVAIWSAVVYVPIYLLFLPKHLTDPTPIHLISQTLFHGIFVVI
IATLTYAEAIKRLGAFKAGSITNLAPFIAALLAVPLLGESLSIAMICGLVGMAIGALQPWRWVLHRDSLEARLAE
QKKQSK

>Hydra XP_002159120.1 CG14040 [Hydra magnipapillata]

MEAETLVGVQSLFRDIVKHSNVLLLYMIPAFLYCLYNNLAFTNLRSYDPTTYFLLLQFRVVITGVIFQFLFNKKL
SRTQWFSLILLTVGCIIKHLHLSKETGLPKISFTLNMSLLMILLQIFCSCFAGVYNEYLLKDKGDSAPFMLQNVF
MYTDSVICNVLLLSYSGEIYNVFLKKNIDSVLHPIVLTVVLNNGAIGIVTAMFLKSLNSILKTFASALELMFTAI
LSWIIFGIPVNFMTIVAIGIVSFATLLYAKNPVDNTPKTTGLSKV

>Acropora1 XP_015764247.1 UDP-galactose translocator-like [Acropora digitifera] EST assembl_3216

MAAGGTSVSVSVKVWSLLILTLQNALVVLTLRYTRTLPGDMYLASTAVMITELLKAVVSVAILFGQSGNVVDFFKSL
YSITFGQPLDAAKMLVPACIYTVQNNLLYIAVSNLDAATYQVTYQLKILTTALFSVVMLKKSISKIQWFSLFMLF
VGVSVVQLQPVTDKAQSAKSPSLHSQNPVLGLAAVVASSLCSGFAGVYFEKIVKGTRMSLWARNFQLALFSIIIG
TGGMYINDGEKIRQKGILFGYHKLVWLIVGLQAFGGILVGFVVKYTDNILKGFSAAISIVVSCIASVYLFQFKVS
IQFVTGAGLVMLSTYLYGIGQSSAAKESKHDKSKLDKKVD

>Acropora2 digitata adi_EST_assem_15762   Length = 1338 Expect = 6e-61

FRMFIFAILTLQATCYFSLLRYSRTRPHKMYFTSTAVFLAEVIKLLVTLVVIVSQHRNLKQLGSFLVDSFLLNPM
DTMKLSLASVLYVIQNNLVYIAMTHLESTTFQVLNQLKILSTAIFSIALLKRQLTKRKWISLFLLSLGVTMVQVD
SQTSVQAVILTDSNNPATKNKQSSILGFSAVLASSLASGFAGVYMEKIFKGQKRKSFWVANAQLYAIGVILGLVG
VFYQDGGGIAKMGFFYGYDLVVCLVILFASAGGIIVSLTLKYASCITKGFATSFAIVLSSFVSAYVFNFIPSMQF
IVGAASVVAAVLTY

>Acropora3 digitata adi_EST_assem_7374   Length = 1401 Expect = 5e-81

SLKYISLITLTVQNASLILTIRYSRTLPGDMYITTTAVVFAEILKVLASLLILLLQKGSVRDWLHFLYSSIIGQP
FDTLKLSIPALIYTVQNNLQYIAISNLDAATFQVTYQLKILTTALFSVFMLSKNLVKLQWVSLVMLFAGVSIVQL
QPTNLSPNTTHPLDSEKAQVTQNPFLGLVAVIMSCLCSGFAGVYFEKIVKGSQTSVWLRNIQLGIYGTIIGIAGM
YVKDGEKISDKGMLFGYSTLVWIVIFMQAFGGLLVAVVVKYADNILKGFATSFAIILSCVVSVYLFSFQLSSQFL
FGAGLVILAIFLYGRPQNQDKT

>Acropora4 digitata adi_EST_assem_3553   Length = 1158   Expect = 2e-65

IPISLKNICLGILVVQTSSLVLTMRYSRTHKQPGEQMYIASTAVVFAEIFKVLACLAIIFRGSSYRWSVFANQLR
EEILAKPWETLKLAIPSGLYTIQNNLLYVALSNLDAATYQVTYQLKILTTALFSVCMLHKRLGMLKWLSLVLLMI
GVTLVQWHVDEKAPTSKELSLSGQVVGVLAVLTACCSSGFAGVYFEKILKGTKASIWMRNVQLGSFGVVFGLMAV
MTNDGSQVSRGGFLQGYNRITWIVISLQAFGGLVVAAVVKYADNILKGFATSLSIVVSSFVSFYFLGDFQPTSFF
FLGTCGVLVATFLYGVPERQAAEVK

>Tribolium P_973292.1 CMP-sialic acid transporter 1 isoform X1 [Tribolium castaneum]

MSTFSWIELFPTKLSFVIFLAYILLFVGQGILVTASQKADNQYDYNIITVVLLTEVLKLIVSTLLYCKDNSPKSL
VNNIVENRKVLGLYFVPALLYCFYNNLAFVNLSVFDPTTYYLLLQLRVVVTGILFQVIFSKTLSKKQWLSLLILT
FGCMLKQINFTNQEKKSFISFDIVGLNGIFILLQIFCSCLAGVYNEYLLKKQGADVNIFIQNVFMYLDSIVCNVV
LLSVRVSLSSAFTYENISKVFHYKVLLVMFNNAAIGIVTSFFLKTLNSILKTFASALELVLTAILSYLFFRIAIH
LNTVLAIGAVMYAVYLYSQNPVSSKASSRQSDQIALIKSEQV

>Drosophila XP_017130017.1 [Drosophila elegans]

MSTNWRELFPTKLTFVIFLLYMSLFIGQGIFVTASQESNNSYGYNTVTVVLLTEVFKLIVSTCLYCRDHNLRSLV
RDVHKDRNVLALYMVPAFLYCLYNNLAFVNLATFDPTTYYLLLQLRVVVTGILFQIIFKKYLSQRQWISLIMLTL
GCMMKQIDFGSFYSDTNDDSELAAIQQSSINRTAIDAPQAHGKNMSGFDFSLSAVFILAQTICSCLAGVYNEYLL
KDKGADVNIFVQNVFMYLDSIVCNAVILLLRGELLDAFSPHNLSSIMRFSVLIIIVNNAAIGIVTSFFLKYMNSI
LKTFASALELLFTAVLCYFLFSIPIYMNTALAIAVVSYAIYLYTQSPVVNLGKVRPLANLSDATAKSTDKRKLLD
EEAAESDLDMV

>Desulfovibrio WP_051286295 Eam famlily transporter D. gigas

MQTRMSGRDTAVALLTTFIWGLNFVVIRWGLGEWPPLFFAALRFAVVAVPAVFIIGRNGLPWRLVLATGSLVGVG
YFSLLFVGMQLGMPPGLSALVSQAQVLFTALFSVWLLRQRPTAWQWCGMGVAGAGLYLSAGNSLDLAPLAGFALV
LLAALFWGMGNIMMKKLGGGMKVDGFRLTIWMSLVPIVPNLILSFWTEQGQWEALSHISWMGAFAVLYTAWGSTI
IGWGAWAWLLRKYAAPQVAPFSLLVPVVTLVIAVSLGMEPLKPETGMAAALLLGGVALTLFGDRLGGFCLRRQWL
RPRAAEDGYSTKS

>Phaeomarinobacter WP_081826331.1 EamA/RhaT family transporter [Candidatus Phaeomarinobacter ectocarpi]

MTTPHLAFMVLINLIWGFALVAGKGSLEHFPPFLFMAIRFGLVALVLLPFLSIHWGRMRDVMLIALCAGPIGFGF
FFAGLAIAEASVVAIASQMAVPISTIFSVMFLGERIRWRRWLGITLAFLGVMVISFDPRVFSYIEGLIFVLCSAI
VGSFGTILQRRIKGVGTFELQAWVATVALPFSLAATFLFETADPVALMASADWIEWGGIIYVAFASSLVGHAGIY
WLLQRYEVSQTAPYTLLAPLFTVTFGVWLLGDMLTERMIFGGLITLAGVLIISLREKKFTERVTRGA

>Pontibacillus WP_027446152.1 EamA family transporter [Pontibacillus marinus]

MKGKFSTFLVLLAAILWGTKGSAQTFTPENAHPIAVGTMRLAVGGGSLLFVALLLGQVNLKNWPIKATILASLSM
AFYQPMFFSAVHITGVAIGTVIAIGSAPILAGVLERVFLKRRLSKVWWYSTFLSILGCTLLFINTGSVQVNPYGI
LMALGAGLSFASYAMVSEKLVKEHSSLSVIAIVFTLSAFLLLPFLFVYDMSWVTEARGLMVSLHLGIIATGLAYF
LFARGLIHTPSSTAVTLSLAEPLTAALLGVFLVGERLELLSWMGISLLLVGITMLIVSPKRSA

2 - **Sialidases sequences**

>Saccoglossus1 XP_006821458.1 sial isoform X1 [Saccoglossus kowalevskii]

MYVKGAPCVVFVVLTFWCGFPVFSAGFTTFRNVTPKVVQEKLIWIKGQEEVSLYRIPIISYTSQGSLIAASEARK
YSGSDHGPKFLAVKRSTDKGYTWEPQQFVSDDGSIVKDGLNLGAILSDDEKGIMFIFYTMCAHYDHCDTSTTHVI
RSYDDGVTWTNSFNVSEQIGTRMFAPGPGYGIQKKLNPNKGRLVVCGHSTLSGDGMFTVISDDHGNTWRYGGMVK
SIPYNAPKKSGDFQPDECQPVELPDGSIMINMRNQGHYHCSCRIITRSFDGAETFSFDNLYFDEALIDPACAAST
LYHNGVLFFVNAMSTNKRENLTLQWSYNNGTTWSGKLNIFPGYSAYSAMTAFSNSLKYGDELYILYEKGQNSANS
MDSLSFVRVALYG

>Saccoglossus2 NP_001277018.1 [Saccoglossus kowalevskii]

MYVKGAPCVVFVVLTFWCGFPVFSAGFTTFRNVTPKVVQEKLIWIKGQEEVSLYRIPIISYTSQGSLIAASEARK
YSGSDHGPKFLAVKRSTDKGYTWEPQQFVSDDGSIVKDGLNLGAILSDDEKGIMFIFYTMCAHYDHCDTSTTHVI
RSYDDGVTWTNSFNVSEQIGTRMFAPGPGYGIQKKLPPHVGRLVVCGHTSKRGMVCVISDDHGETWKNGGYIKSI
PFKQPTKAGDFNPNECQPVELPDGSIMINMRNQGHYHCSCRIITRSFDGAETFSFDNLYFDEALIDPACAASTLY
HNGVLFFVNAMSTNKRENLTLQWSYNNGTTWSGKLNIFPGYSAYSAMTAFSNSLKYGDELYILYEKGQNSANSMD
SLSFMCTTRKDEKSQTIGHLAKDMEMLEMGITWNEFHWLAQDREGWRSYTGALCSSGSEED

>Saccoglossus3 NP_001277015.1 [Saccoglossus kowalevskii]

MSVHVVSSSSVFFWLCLIFVAYASLSPLIVDDQVLWTSGDEGDISFYRTPMLINTPNGDLLAFAGARKYSEGDTA
QKIATMRRSRDKGASWDPTVFVIQDLGPETDYFNFGTITVDDAVNSLVFLYCHCPHNVCKDSLPTVFMMRSYDWG
YTWSKPVNLSISNPIFYNFAYCPGPGYGIQKKIGPYKGRLISCGHTIKTFKAMHCIISDDHGDTWKLAGTVPTIP
YGYGLKTGDFQMDETTIIELPDGSLMLNSRNEHHYHCRCRIIATSEDGADTFPFQNIRLDETLVDPACDGSLISH
NNVLFFSNPANPSARVNMTLRWSLDFGKSWDGALVINAGSSEYSCLSSIDDNHVGLLYEKGGYKEMSFAKIRIN

>Saccoglossus4 NP_001277016.1 [Saccoglossus kowalevskii]

MGSESTIFIMISAVLCQASAVLSPVVIEEQYLFQPGNPGEVDAYRIPISIMCPDRSILAFAEGRKYGRGDYGSKF
LAMRRSKDSGDTWTTTEFVLDDNPSVDGLNLGNVIIDYYNNSVIIVYVHAHTHSELYMVKSLDWGVSWGKAIALS
DRNPKLKGYEWTAGPGYGIQLKYGPHKGRLLSCGHGMSLELREASCLYSDDYGDTWHLGGRVLGIPFNHTKNTGD
FAPGESQIVEMYDGTLIMMSRNTEAFHCKCRIFSKSYDAGESFPVSDIWMVDDLPDPNVCGSILHHNGILYFSGL
DSRTSRTNMTLYWSLDNGITWPGYLPIYLHNSAYSCLTAIDNNNIGLVYEKGNNGHIAFSKIRLHA

>Saccoglossus5 XP_002742125.1 [Saccoglossus kowalevskii]

MNWKTENFNCASYRIPAIVYNKGVFLAFCEARRTSFRDYGEMDLVLRRGILLEKQVEWEGIQVIASMRGERTMNP
VPIVDNVNKAVVVVFNTFPSNVTETDLLKEGVFNQTVFVMKSTDNGITWSDPQDITDTTLGKVEPAWTIFSPGPG
HGIQLSSGRLIVPGNYYVKDPVEPGIWPQIKTAVLEFFDVLYGAVGTSNVFYSDDGGSSWHVGGSVPTANAGELE
TGKTIHANESQVTELDDGVICINCRTLGVEMPRVQAFSKDDGMTFTVAQVVDKLVEPGYTGSFMPSTKNFGGCQG
SILGFPAPEGVPDGFSKTWALFSNPADKENRINLSVRLSIDGCKTWSAPWSLCPGPSAYSDLTYFEMDDGNGGIN
KLFACLYECGTTDFPYEKIVFQMFTLESVLEGTKH

>Saccoglossus6.1 [Saccoglossus kowalevskii]

MRSIRLKTCTLLVFVSFITIYVVMYMLPDRNNQPQLTQYQHQHHHRDENIIPFFNKPHEPRLVKQNTDFKLPIRW
KQDNVFMSEVMDVFTGNEKGFNVTRVPGMLYHDGQFLVFCEARARLADNGKMAIVSKRGLLQPDNSVQWEESKIV
VQVAGLRTSNPCPVYDRMRNTVILVFLAVDPRIGEKYLMKMGQHQVTVAVVKSRDFGKTWSDPIDITGTTIDTIQ
NPPVAMYAPGPGHGIQLESGRILIAGNHYQKDWLGIAGPRSLLNNSDYSNVIYSDDGGDNWHMGGIVPSSRDLAG
QKIITNEAQVAELDNGVVVMAMRTLMSRQPRAQAFSYDGGITFQKAEVHTSLIEPGYKESYGFLIPKNTPGCQGS
ILSFPAPASNYGNQKWVLFSNPASKTTRKYMSVRLSYDSCKTWSEPWTIYPWYSGYSDLTYLGIDQGVPSLPSFM
RVERCQKPRK

>NEU1_HSA NP_000425.1 NEU1 precursor [Homo sapiens]

MTGERPSTALPDRRWGPRILGFWGGCRVWVFAAIFLLLSLAASWSKAENDFGLVQPLVTMEQLLWVSGRQIGSVD
TFRIPLITATPRGTLLAFAEARKMSSSDEGAKFIALRRSMDQGSTWSPTAFIVNDGDVPDGLNLGAVVSDVETGV
VFLFYSLCAHKAGCQVASTMLVWSKDDGVSWSTPRNLSLDIGTEVFAPGPGSGIQKQREPRKGRLIVCGHGTLER
DGVFCLLSDDHGASWRYGSGVSGIPYGQPKQENDFNPDECQPYELPDGSVVINARNQNNYHCHCRIVLRSYDACD
TLRPRDVTFDPELVDPVVAAGAVVTSSGIVFFSNPAHPEFRVNLTLRWSFSNGTSWRKETVQLWPGPSGYSSLAT
LEGSMDGEEQAPQLYVLYEKGRNHYTESISVAKISVYGTL

>NEU2_HSA.2 NEU2 [Homo sapiens]

MASLPVLQKESVFQSGAHAYRIPALLYLPGQQSLLAFAEQRASKKDEHAELIVLRRGDYDAPTHQVQWQAQEVVA
QARLDGHRSMNPCPLYDAQTGTLFLFFIAIPGQVTEQQQLQTRANVTRLCQVTSTDHGRTWSSPRDLTDAAIGPA
YREWSTFAVGPGHCLQLHDRARSLVVPAYAYRKLHPIQRPIPSAFCFLSHDHGRTWARGHFVAQDTLECQVAEVE
TGEQRVVTLNARSHLRARVQAQSTNDGLDFQESQLVKKLVEPPPQGCQGSVISFPSPRSGPGSPAQWLLYTHPTH
SWQRADLGAYLNPRPPAPEAWSEPVLLAKGSCAYSDLQSMGTGPDGSPLFGCLYEANDYEEIVFLMFTLKQAFPA
EYLPQ

>NEU3_HSA AAI36398.1 NEU3 []

MRPADLPPRPMEESPASSSAPTETEEPGSSAEVMEEVTTCSFNSPLFRQEDDRGITYRIPALLYIPPTHTFLAFA
EKRSTRRDEDALHLVLRRGLRIGQLVQWGPLKPLMEATLPGHRTMNPCPVWEQKSGCVFLFFICVRGHVTERQQI
VSGRNAARLCFIYSQDAGCSWSEVRDLTEEVIGSELKHWATFAVGPGHGIQLQSGRLVIPAYTYYIPSWFFCFQL
PCKTRPHSLMIYSDDLGVTWHHGRLIRPMVTVECEVAEVTGRAGHPVLYCSARTPNRCRAEALSTDHGEGFQRLA
LSRQLCEPPHGCQGSVVSFRPLEIPHRCQDSSSKDAPTIQQSSPGSSLRLEEEAGTPSESWLLYSHPTSRKQRVD
LGIYLNQTPLEAACWSRPWILHCGPCGYSDLAALEEEGLFGCLFECGTKQECEQIAFRLFTHREILSHLQGDCTS
PGRNPSQFKSN

>NEU4_HSA NP_001161074.1 NEU4 Homo sapiens]

MGVPRTPSRTVLFERERTGLTYRVPSLLPVPPGPTLLAFVEQRLSPDDSHAHRLVLRRGTLAGGSVRWGALHVLG
TAALAEHRSMNPCPVHDAGTGTVFLFFIAVLGHTPEAVQIATGRNAARLCCVASRDAGLSWGSARDLTEEAIGGA
VQDWATFAVGPGHGVQLPSGRLLVPAYTYRVDRRECFGKICRTSPHSFAFYSDDHGRTWRCGGLVPNLRSGECQL
AAVDGGQAGSFLYCNARSPLGSRVQALSTDEGTSFLPAERVASLPETAWGCQGSIVGFPAPAPNRPRDDSWSVGP
GSPLQPPLLGPGVHEPPEEAAVDPRGGQVPGGPFSRLQPRGDGPRQPGPRPGVSGDVGSWTLALPMPFAAPPQSP
TWLLYSHPVGRRARLHMGIRLSQSPLDPRSWTEPWVIYEGPSGYSDLASIGPAPEGGLVFACLYESGARTSYDEI
SFCTFSLREVLENVPASPKPPNLGDKPRGCCWPS

>Emiliania XP_005763320.1 Emiliania huxleyi

MTMLLLATLLSALTPPPDLTSTDLFTPGMPDTAGVTYACYRIPSMVWVANEASPHAVLLAFAEGRRGSCADKGDV
RIVARRSSDGGQTWSSIEQVAVEAGHTIGNPAPVADLAVAGSVHLVHARDDTQALAVFLVSSADGGRSWSERRNL
TAALKANPAPEAFVMPGPPGGVQLQSGRLVVGMYGEDEAKQVRSYAAFSDDHGRTWAHGSPAGTSASGPVYGGGE
NQIVPYGGGGTLAMFLRGRTTAADDVSHNHGLAWLNISGSYCEGSAVATPDGGLLSRRAEQPWMTSLTVVYFWPG
FKATAPEMGYPVLQPVLQYGERGRAWALQSWFVDANDRRFPVATAPAVDVQPGDRITSYMSLSADGSTWTVSGTN
RESGEDSTLHIAHSRAGRADYDYAMLVNENINVDERCDRMPAAPSLTFTNVTVNGHAKPAWATRADCHGSPRCDC
GNAASIGANGDVTLSWSTDPRSRPSRRASR

>Sporothrix KIH89835.1 Sporothrix brasiliensis

MRSSLLASFGLAVLTAAAPAHRSSDLITNTTLATASSGPFNYYRIVSLQSLGNGVVLAAFDGRPTGADAPAPNSI
LQRRSTDYGATWGSLTYIAEGQAANAGTGALAYGFSDPSYVYDSATSTLFNFHVFSKDASFASSVLGHDDTSRNI
TSAEVSVSTDGGLSWSTDPTNQPNLPPVASSQVGVPPLLTSAVKPNGSPVGSVSDVGAVLGEFAASGQGIQLQYG
AHAGRLIQQYLGRVVQPSGSVAYQAYSVYSDDHGATWQMGTPTGVGMDENKVVELSNGTVMLNSRPSDGSGYRKV
ALSNDGGLTYTTPRTETQLPDPACNGQISRLYPSAAENSAQAKILLFTNANSKTSRANGTARFSCDDGNTWSTGT
VFSPGATSYSTITPLGDGTVGLFYEGLNNQLIYLRVDTAWLGINC

>Pseudogymnoascus KFY06985 Pseudogymnoascus sp.

MLYTSQSTLRYAVSVLGLCALPSVLTPVQAAPTPFTINSRELFKEQVLATNGVGPSPYYRIVALANLGNGVVLAA
YDGRPDGGDSPSPNSILQRRSTDGGETWGAPTYISQGQLGSTGVQKYGFSDPSYIVDQDTGKIFNFHVFSKNVGF
GSSVIGNDDANLNIISSQVSVSTDGGVSWSTDPKNQPSLPPAASSLITKSVKPVGQTVNGVASVGGVVGQFAASG
EGIQLRYGAHAGRLIQQYLGKVIQPSGSTASQAYSVYSDDGGATWHMGSPVGTGMDENKVVELSNGNVMLNSRPS
DGSGYRKVAISTDGGVTYSTPKSETQLPDPANNGAITRMYPDAAEGSANAKILLFTNANSKTNRVNGTARYSCDD
GTTWSSGRAFQTGVTSYSTITALGNDQFGIFYEGADNTLKFAKVDKAWLGVSC

>Bodo CUM57966.1_Bodo saltans

MSSVISAFGMLLLAIVLIANTAVIAEPSPFHEVDVFTIGEAGYFCIKIPYLFTTFNGTMLAFGEGRKESCSDFAP
TDLVFKVSEDAGATWGPLRVLHTNGSNTVGNAAPLQLRSNGRILIPFCINNLYVYQMHSDDNGHTWSIPEFIPGA
TQPDWQWVGTGPPGGLQLASGRLVVPSYHSYVANTDGDIARIHMMINDDPSGNASSWFVGGAATGIEWTNECQAV
ELEPNHLLITTHGFVLPQRMQVESFDGGMTLGEPYFIDIGAPFGGCEGSIAKLNNSLLS

>Chrysochromulina4 KOO27867.1 sp
MEPSKPSSRAAYIQQSVRRSGRPQPYLQLEVQVMSVGKPPTTTLDSPTFGAATAMAGASLESPRVPSGPMGAPSG
QHVMLRRSSSLSSSSYVAPTVSLPPPEEFAGGDTDRKAGSASEAPRRESLSPSKRSRRESGGAASASSTSTGTAT
VRAAPDPSPSLDEAEEESVEGLGSPKKRPSLGKRESGAAVAERAAAAASAPPSITIVDMFTPGMLRVNVFRIPSL
LPLPSGVVLSFAEARPQLVDHGVINIVMRRSTDAGRTWGLARVCVEGAMVGGAAKFTVGNPTAVYDKTTKLIWLL

FCTNHAADAEWMIHAREGRDTRRVWITSSADEGLHWTVPAEITASVKLPGWTWYATGPGAGIQLTSGRLLIPSNH
AGDVYEPNCPYLASTQRSRMVAHVIYSDDHGATWHIGGVAAKHTNECTISQLGDRRVLLNARDWSGRFLRAVQLS
SDDGASWQKHRYDRELIEPRPQGCQGSMLCVPPPEGRAGPGILFFCNPSSDRREMLTIRRSDDGGMTWSRSYCLE
EGPSAYSCLGRTADGALACLYERADRISFAKIPSHVDGPLGVF
>Chrysochromulina3 KOO22942.1 sp.
MLFGLLAGLIAVLVPCAVFTEAERNFCYSIVSKVNRHTNCGVDWSLPLTEEQAQRAISCDHEQCDEPRRGDPVAV
FWAGYNDTACFRIPTVITSHTGTLLAFAEARLTSCSDDTDHKLVLRRSLDGGTTWGDLIVAVDGIVPCEGCPASV
SNANPVAVWLQGGRAAILLHFNTLNNPTQLVHGVAMQIWSFDDGLTWEGASVLTYPPVHNIGGLIGPSVGLQADD
GTIFFSARQPERGTFLYWSSDFGASWRASELVPTDEFGLDECSIAWLRNSSDGEILMNCRTRLNERALMTWTADG
LPGRVTYPGLTDENCQGSLLNAGGAHFLSHINSRWERAHLQIHRSTDGGKNWTEVRKVRRGAAAYSQLIELGAAP
RGGGIRLGIIFEGGTWCPYEVIGFWQFTFRESAARRTALSVDSPA
>Chrysochromulina2 sp. JWZX01003082.1
MLTGVLFTLGLAKTSQALRVDQDCQAVIDDYCRESCDLRSRGCDYPTVARYSGKSGTVEWRCYSPSTLDNNQTYR
GGDCYCSRGHEIQEALSLCFNDVTTVFSTGMAGEHCYRIPTIIRLEQGPLLAFAEQRDPGCGDGGSNNLVMRRST
DDGQTWGDLITIAAGGGAPFSNPNPVEVDLGDHKLAVLLHYDTMNNPTSAHHGSNMQIWSYDGGATWVNSTNISA
FMPEGYAGCMPGPSVGVQSMDGTIYFSCHGFGVNGFLYWSRDLGRTWRASEVLASLNECSIALLANQSIAMNCRT
AGSFREQLTWAPDGSLLGEIRRPAGLVDPNCQGSLLAHGGALYLSNDNTTSGRTHIVVKRSADAGLTWDEGRLIW
AGPAAYSQLVGMGDSRLGLLFELGVETTYESIGFRSHQISSRPHSTAEILSRMAPSASLITYLTMWYVGNYWYNI
YNKNAGKLSGGANFAFTNATMQLVIGCAYAIFLWLAPDARKQPTLTLGQFMSLAPLGFFAAVAHGGAVYAMSAGA
VSFGQIVKAGEPVFAAAVGYFVYKKSESVPKLICLLPVIGGIAIASMQELDFTMASLIAASTANVASAFRGSENK
RVMTGELSSAIGSGGNSYALTTIWATILLTPTVFISGEFDRIDEYLALLRSNSTFRYNLLMSGVTFYLYNE

>Chrysochromulina1 sp. JWZX01002253.1

MRESARELNDARLRDGVEKVKTTPVSCCWMLVRICLLRLYCTDLNYGVIKRGFDVFLVASTPINDPDPRVTARPQ
TLPMCIRYATLECATNAMKSYPLFVLVFASAAPAISAAPTMFDVFHRQQVDSANVSYFCFRIPALVRRADDALIA
FAEGRHVNCDDAGDVRIVRRISYDEGETWSDIAQVKVEPGHTIGNPCPIVDLVTGHIHLLYSRDNKEVFVTRSTD
GGESWGASTNLTASLKLELDPESIFVATGPPGGVQLASGRLVGGFYYNGLNGTRSAAIFSDDHGATWRRGAEVFV
SQTPLPNASSVVYMGGECQVTSYAPAGPQGLVMLMRVRGDFPTNAVDHNHATAISRDGGETWTVASLIHATSSYC
EGSIATVGKSVLISAPSTTNGGRANLTVWAARPVGESLDAQYSLTVYPGASAYSSMLAGKRGGTVLNLFERDNDP
YVPKNLTLATFDVPALR

>Aureococcus anophagefferens ACJI01001184.1

MVSPRCPLLALATTLLVNGRELTPTTLPPAGHGRAWSSYDWCDCRTGLANVTIWAGDEQLTLAVGPATAFVALDA
ATTELRWRCDGMARAQRASAPAATRVWGARFSSETKTPFCGAASGRLAWTGWAANDNVLSVPFVSGGDGYACTKI
PSLLRLASGAFLAFAEARWPDCGDFSRTDLVAKRSEDGGKTWSAAQVVAPAAPGTRGLCGHEVVVGNAAPVQATK
GPRAGRVLVPHTRDNFAVWSVHSDDDGRTWSAPRALPGLADTDAAGPDCNRTMAYFGVNTTASVAAWVEAIARGD
GDPYREWGSKLAGPWQFLGLGPPGALELDDGRLIAPGYHAYIRGLDGGGGAGAGVDLPVSQLYNNLALGHVVYSD
DGGDSWALASRRGFGGGAAGGSHGANEMQLVQLRNGSLLANSRSLSTGSRQARLAARSDDRGLTWTPSSFVDALP
EPFNGCQGSLARSPRALYFSHPDPLPNDAALPRALRGLGGGVNLTGRDHLTLWASRDEGDTWDVRGVVDAGTAGY
SSLAVVGGEHCDAVYVLYEQADPAAASLAAASAAAVVGGLAVLNPDRLVLYEEAACG

>Orbicella Orbicella faveolata MPSW01000116.1

KVYHDLNLITANYAVNAFSIIFLGDIGVNTYRIPIITSLPDGSLIAMSEGRKHSSADSGPKFLAMRRSKDQGITW
SNTTFIEDDGEDPDGLNLGTVFVDEDANRVFVVYSYCAHKCVYHTTYLISSDDFGDTWSKPSNLSDQIGTSLFAP
GPGFGIQKKQDPHRGRLITCGHASPGHDGVFCIVSDDHGVNWRVAGSILQNGEFEPDESQLIELYDGTLLMTSRN
QQSFHCHCRIMSKSYDGAESFAHSDIYFDETLIDPVVAASLLQYRNTVYFSNPASKLLRINMTVRWSGDKGTSWV
GSLGVWKGPSGYSCLTNVPGKLANDTFIGLVFEKGNIRYYESIVFVRLRV

>Nematostella XP_001630023.1_Nematostella vectensis

MAGLALPVTKELFYSILIMLSFGPKKCYLQRSEEFGSLNEVVLWKRGDHGVSDYRIPLITSLPDGSLIALAEARK
HSSSDSGPKFLAMRQSRDQGVTWTNTTFIEDDQEATDGLNLGSIVVDEITKSVFVMYSFCIHKCKYATTYVIRSD
DNGQSWTMPKNISQQLGGLQFAPGPGYGIQVECSSPKGYQVEYSSPKCYQVECSSPKGYQVEYSSYKGYQVEYSS
PKGYQKALDPWKGRLIICGHSIGKTEGLYCVASDDHGNSWHRTAELPRTNQFDPNECQMIEQSDGVVLLSARNQQ
HEECRCRMMAKSYDGCETFAHSDIYYDHTLIDPTVAASLLNWKYTVYFSNPASRTLRVNMTVRVSGDMGETWKGL
RPIWQGPSGYSCLSGIPTSQGNDSYIGLAFEKGLISPYESITFVKLRV

>Blastocystis OAO16540.1_Blastocystis sp. Chromalveolate

MMKTLVFAILLCVIACDPCDGCNPRVKSVELFKPWEDGFPCIRVPSIIRTPHALIAYAECRMITGDGCNPTPIHK
ILNDNTMYVCQKKSVDGGKTWGNLTYPFGREIATREASPVYDFINDKLVVVANLMDSQLIMTVSEDEGDSYSPME
NIEEKVGAAHTTYVGVGHGLQIQGGPHRGRLIFIGHHGAYEYDPVWYTDDVGKTWAASNTLMKKMDEAQLVELGD

GIVMANMRNQHLQGNTRAVSISMDGGENFGPIYYDPALIDPICAASIIRDYRTNVIYFSNAAHPTKRINMSVKRS
SNWGKTWDRTLLVYEGPSAYSDMIDLDSDEFIGMTWETNSTGCAGESCRTLFSFIPKNF

>Karenia2 DAA35231.1_Karenia brevis NEU1.1 dinoflagellate

MAHVLLYTRMARVLCILMCSWCHVPAFAWMTPECQNIVRSTCVGHCYPELAGVPCDDIRCKISSDGRDQPKMEHC
NCSWNAKMANLLATCDSRADGSVTVFGAGEANISCFRIPAIVRTVKGTLLAFAEARHGGCGDGQVHQIAVRRSID
QGKTWSELTFAAGSDQLFVGNPYPIAMSGGDVALVFVNHTHGHGADLGSGNGVIFSRDDGITWSAPLDISQGFGA
ATGSLPGPGAGIELGASHRMLVVSHHGAYVNDYISYSDDRGTTWKTIGQTFPKMDEATLADLGGGSVLLNMRHRA
ENTKGRGVARSQDGGLSWSNITYDSALIGPVCQGSMAAFNGSVFFSNPASSSGRDHLTVRRSDDGGHTWTAKLLI
QEGRSAGYSSLVQGPVLDDNHGGILYESTAAGSVDFTVFPLNFQDRDLTVIV

>Karenia1 DAA35232.1_Karenia brevis NEU1.2

MSLSTMCSALSAGLMALATAASTKVGLVNVFSKGESGYYCIKIPYLTSLPDNSLLALGEARWGSCSDYTKTDLVM
KTSWNGGKTWSALKIFHTGGEADTVGNAAPVVVGQDLIIPFCLNNSKVFIKRSGDLGETWSNAEDISSNVTRAHW
KWVGLGPPAGLLLSSGRILIPGYHTDIFGPYVDNGDVSKGHAMLSDDGGKSWRLSADDDFGGRHFPNEDQAVELS
GGHVAIFARGLGFFRTRTVSEDGGDHWGTTEVLETLPQPFIGCEGSTIKCRESKRLVYSGPVHFMTRLREDMSIY
VSDDEGRTWQKYMRVDPGFSAYSALAWHGKNLALLYDRSNVTTDVRFEPDHMSFAALPNPCVSNDVLI

>Monosiga5 XP_001749953.1 [Monosiga brevicollis MX1]

MAVVVAVLLVLVASVLGLPSSHSNWQSLVNKTDLFVNNQPYGITYPCYRQPALILMGEGLLAFAEGRNISSCAPP
LQAAAPSPVEEVGGLVLRRSTDLGRTWSAPITLYSGNIDFYVTVYDAEKDVGWLFLQEESAVRVFNTTDQGYTWA
GPFAFTAPVPPGLRPTVQPAVGHGLQLQRHLCAAPGGCAEAGTLVVPFVCMSDTATPVERVHSDTACASCQSCLL
LSSDHGASWRFAALGQLGSREAQAVQIASNTSSAWLYVTERNLGPMPGHRMYAISHDAGHSYTETGINKGLVTPI
TADWTGTVASVESSFNQQPSSPLFMALPFNATVRCNLTLMTSLDAGATWAAQTVFWPGLGAYTDLSALPTGLGVI
FENGEATFADRISFANLLQ

>Monosiga4 XP_001749157.1 [Monosiga brevicollis MX1]

MPTLASHWAVYARVSCLRTVFEGGKEGYFCYRIPSLVQLGPDSYIAFAEGRKFSCEDHGWNDIVAKTSSDGGQTW
SALAVVYGESSTKANITIGNPAPVVLHDGTLLLVFCRNNQAMLLLNSTDGGQTWGGFRDVSAGIVDPTWPFFATG
PPGSIQLHTGRILVPADHTSGSTQSMAIYSDDYGQTWTRGAMIPDGNECQAVELPDGTVMMNMRTSQAHRQVAFS
KDGGATWSDPVVTVNGTVCEGSTIMLPDSTGTLVQSFPNSNDRANVSIQTSTDGLSWGNSIQVYAGSSAYSSLQQ
ANNNTAVALLLEMDSYQKIAFAMVSLV

>Monosiga3 XP_001743349.1 [Monosiga brevicollis MX1]

MKLLALGLVLGCLSVADAAIQVDQVDVFIGGTRGHACYRVPTAVTLPNNTVLMAVESRRTTCGDQAPKDIELVRS
QDGGFTWSEPVMLVGSLTGNTTYRNPYLTVVPGSSTTATPDLVLLQFVNSTLDEPWTTFQMESRDAGLTWSSAQP
VNLSAWNGTLAGPGLGITLAHTHPGRIIMCGASGYHAGHSANGVVWFSDDQGASYVVSAVFPEMQECQVAELADG
TLLINFRNSHLNSSCDCRAQSVSHDGGLTWGPLTYIPDLIEPVCSAGLVAANDTLYFTNPNSRDQRINMSVQSSM
DGGNTWSHEVTLWPGPSGYSVATNHPNSKCTLGVAYERGEQSYAERITFAHINLCYE

>Monosiga2 XP_001749247.1 Monosiga brevicollis

MGEQVEGQRPFRNATRQIAQRVEPGCHNRQSQSIANSRRQQTADSRQPRAAARQCIKFISLSLSLSLTHTHTHTR
NTKKKVTVHTHTHTHTHTHTHTHTHTAAVPVPPVGPSVVFAKGEAGYYCIKIPDVVHFPNSSTLLAFGEARRDSCSD
YTATELVMKRSEDLGRTWGNLTVVHANGAHVVGNAAPVLLPSGRLLLPHCLDNKVVYTMHSDDQGMSWSTPTLVS
NAVRDDWRWIGLGPPGGLYNRGRVVIPAYHDTSPHGDDGQFTHPHTLLSDDEGATWRIGATINSTELDPFFSNEN
QAAVLSNGSLFFSARTLLERRSQWLSHDNGESFGPVQLVETLIQPVDGCEGSIVTLASGALVLTHPAAWDLRYNL
TAHVSQDNGTSWTPSVVIDPLPSGYSALVDLSSPEGGQAVVGLLWEHAPLPKQLVFVPTDIRFQVIPLSL

>Monosiga1 XP_001748984.1 Monosiga brevicollis

MRCSGLRSGLIVAWLLALLGGSVRAQATVDAACQKVLDNWCNGLEDQTCVNALSSAGAELPLYARYDVAANDDEK
QWRCYSPSSLTPDLSGYSNGTSYCTREQEILDVIADCDVNRTQIAVFTPGELGYPCIRIPSILLAGDNRTLLAFA
ECRNWTGDGCEPQGFEDARYNSTSNANRDLCMKTWSALHVIQRNAAQPMPVWDYQQNQLVLNFVQLTPGDNLQSM
SKDFGQTWTPRGTCPSLDGSPGHAVAPNRLLFIGHHGAYQYDSVWYSDDGGLTYNVSKTNFTFMDEAQLVELPDG
RVMANMRNNHINACKCRAYALSEDGGASFGAIQFADALVSPVCMASILRGLDGNVYFANPGQTSGRSGGMLRRSA
DGFAWNQSALVWPGSYAYSCLTHVPKSNCIGLLWETSAPDCTGDSCVCVFSRLSTADASFGDDC

>Oscarella2 DAA35230.1 NEU1.2 Oscarella lobularis

PTRPRTRGLAFLAFVAVSEGRYRINPKPHHDPQYVWTPNGADQMGVYRIPLITAAPNGNLIALTEGRKIGGGDTG
YKFLSQRSISSQVVVSTQGDQKWSPTIEEIDNGYTMSDGVILGAVVTDNVTKTMFVIYGECEHACPTTRTYVINS
TDNGLSWSDPVDITHHIGTNKTVFSPGPGTGLQKMHAPHKGRLVFCGHYHDIIDGQSYMNTEGLKCIYSDDHGRS
WKNGTVIPGMLYNQPKVQGDFLPNECQPVEMSDGSILISIRNQYKYHGAARMFASSVDGG

>Oscarella1 BK008542.1 NEU1.1 Oscarella lobularis

PRVRTKGSLVLYFSIIFSSSAELFLNPFVEEEFILWNKRGDGDVDHFRIPIATWNERGAIVAVSEARKNTSADRS
AKFLAVRRMAHSVWSKDAWSPTMFIENDGFASDGVNLGAVLSTTNGTTIVVYGTCMHTCPKTRTFTMTSHDDGLT
WGEPVDITEQIGGVYEIFAGGPGYGIQKRHDPHQGRLIFCGWNCTAPFSDDHGKTTGVKCIYSDDEGMRWKSGAW
IPGIPYGQEKAAHDFTPSENQLVELSDGSILITTRNDVGYHGPYRVLARSFDA

>Penicillium KXG53360.1 Neuraminidase [Penicillium griseofulvum]

MLSTLMQSARELSLTRVLSLCAILAVATPAASAAITSQATLAKDGAGPFAHYRIVALANLGNGILLASYDGRPDG
GDSPSPNSIMQRRSTDGGKTWGEPTYVAKGQPKSSSAEQYGFSDPSYVVDSKTGKVFNFHVFSKNQGFLGSKVGN
DDTDRDIVSAEVSVSTDNGVSWSTDPENQPKLPPVASSQAGAPPLITKNIKPVGSTANGVKNVGGIAGLFASSGQ
GIQLKYGAHAGRLVQQFLGRVVQPSGNVISQAYSVYSDDGGATWKKGNVVGNGMDENKVVELSNGNLMLNSRPSD
GSGYRKVAISTDGGVNWSTPKTENQLPDPGNNGAIARAYPDAKQGSAEAKILLFTNANSQSSRSNGTIRYSCDDG
KTWSAGSVFQKGTTSYSTVTALGGDRYGIFYEGAGNELIYLEVSKAFIGINC

>Psychromonas WP_019614358.1 hypothetical protein [Psychromonas
ossibalaenae]

MKIVKYALALLLTLSSYCYAAGADNKNLQIGPQDYIEKWDLAEMGFPDQGYYDIADQPQYNPGLKSQTITFWFKI
PQAGSVQQLVQKGNTSSGEAGWSLFTNNNSLYYRININGQKADLQADFADLNLEQWHHFTAVVDQQAGKLSAWIN
GQKLDQNSGEYASTFTPGEFSNEAPIRVHQKTSGYMVDDLRLYDRSLSEQEASSFIDNTNLPPQAVVDYKHLQDT
QYLFSAQDSSDPEGDELQYMWDFGYGEIKFGKNLNHDFEWGGDYPVKLTVIDPWRNTDQQTVTISVSGEERPLRE
VVVYQPGTEGYACFRIPTIVKAGNGDLLAFAEGRVDNCGDHGNINVVLKRSSDDGITWGPLTVIAEFGQLAAQNM
TAVYDEYYPHTQADGTPVVNSQGEAQFGRLMVMWNNADGGEADVSDDDHPAQRWVLYRTSLDHGVTWSEPTDITD
QVRIPGQKMHIPPTGHAIQLNTQEAAEAGRFGRLFFSGQYNPVGATSATANENYAYWSDDHGETWEIGGLIAGES
LNEVQAVELANGDIMFNSRNYRPTADKRRAVTLSHDGGSSFGETVDDDNLIEPTVASAVIRYTREDRDDKNRLLF
SNPHSQSSRVNMTVQLSYDEGQSWPVKKTINPGTSAYSDLVIHDDMRIGLFYEATTGEIRYASFTLEYLTDGEDF
IPE

>Rickettsiales OUX58241.1 hyp protein CBE49_01020 [Rickettsiales bacterium
TMED289]

MKRADILRSRCPFIVYRQFKMKFFFSRIFFLFNFIIHHPTFPISENTKYIFKSGSDGYNTFRIPTTITTNSGVVL
AFAEGRKNSSSDTGDIDIVLKRSLDGGKTWGKLIVVRDDEDNVCGNPSPVVDSKTGKIFLLSTWNRGDDSEKEII
DLISNDTRRVYIMNSEDEGLNWTVPKEITNSVKKSNWTWYATGPVHGIQIQKGKKSGRMIVPCDHIEAETKKYFS
HIIFSDDGGNSWEIGGITPQDNVNECTIAEIGRGKLILNMRNYDRTKMNRQISISNDYGESWEKLQFDETLVEPI
CQASILRYTFKGKKKNFLFFLNPGDEGERINMTLRLSRDLGLSWDEMIILHKGPSAYSDITKLPNGNLGCFFEAG
QNSPYEGIKFKEINLNDFR

>Rhodanobacter SFL01759.1 sialidase-1 [Rhodanobacter glycinis]

MPSAPLYRRLATVAIAGIIAVAAPSQAAAAPAPGQAHVPLFQRITLARPGDLDVHTYRIPALAVAKDGTLLASYD
ARIDAAHDLPGNIDVMLRRSRDMGRSWSAPQRVVHYGDGIGAGDSSLLVDRDTGRIFLFYAYAPRGIGFATSQPG
NTTDSSTTLHPRYVWSDDNGASWQGPRDLTATIKSPTWKGMFATSGHGLQLFNDQGARGRLIQPYVFRGPNNLNH
VVNIYSDDHGTSWKTGVPIGDNLDESKAVELADGTVMENMRSSDPRMHTRMLARSHDGGIHFGPVAPEPQLPDPH
NNGDIIRVAPLAAPGQAKAHWLLFSNTADPHERRRLTLRLSCNDGRTWDAGRVLDAGDAMYSVMTRLPDGSFGIF
YEDGAGNLSFARFNLAWLHHGCHQG

>Endozoicomonas WP_067586971.1 exo-alpha-sialidase [Endozoicomonas
ascidiicola]

MTVFSRTLAVMTTISTIGLLMFLVMFLLFNASGIEALAALTALAVIYGAVYGFTSLRVIKSLWTDRSEIEKKPSR
SLPYFVAFTTVAALLVMVFLDSVLYDYFQPVSIFDEVIAALGVAWGFLAIVLVLSAFMVRKLSRLVAHRMAALAV
VALAGWPLMATTEWINDPEYHPDLTVVNDVFVGGENGYAIYRIPGMLIIPAGSRLSSGEVLNSDRLLAIAEARRD
GALDNGVIDLVLKISDDGGRSWNEQQVVCRYEVGDARGKCGNPTPVFEQTNGIVFLAHNISGIDGMTPVMISSPD
GGSSWSERIVLPYDDLIFGPGHGIQKSKAPAIGRIVVPGYQVQKGGNRALVLLSDDLGQNWRKSELLNDGDESAV
AELEDGSIYLSTRQHAALGRAPAPNGRWMSTSDNGGISWLDVKKDVALYTPVCQASVLEYGQKGLLFSNPAHTKA
RVNMTVRLSNDQGSSWNTSVSVSVYPGASGYSDLGALSNGDVAVLFENGRMSYSEKITLALLPEQMLNDG

>Kriegella_WP_089893641.1 exo-alpha-sialidase [K. aquimaris]Bacteroidetes

MIHFDRNALLLLLLSIFITSLNAQTGVNNPIESKELNYIFEGNQEGYECFRIPAVVKTNKGTLLAFAEGRKNGCSD
TGNIDLVLKRSVDHGKTWSNLQVVWDDKDNTCGNPSPVVDVSTGTIFLLSTWNLGTDHESEIIKQTSQDTRRVFV
LKSKNDGKRWSKPEDITAEVKLPNWTWYATGPGSGIQITGGKYKNRLMIASDHIEAKTNKYFSHVIYSDDHGKTW
QLGGTTPNDQVNECEVVELSDHSLMLNMRNYNRNKNMRQLAYSYDGGNSWENMGHHDELIEPICQASILRHKSLG

KVNIFFLNPANAKKRVNMTLRMSDDDGKTWGQSRQIFAGPSAYSDLTPIDDENIGLFYEAGHKSPYEGIAWEILN
VRNGLEN

>Pedobacter WP_083505471.1 exo-alpha-sialidase [P. ginsenosidimutans]
Bacteroidetes

MFYTIKNLKKIGGLCCFSALILCCINVTYAATHIATSRVDSLNFIFKAGENGYACFRIPALIRTQNGSLLAFAEA
RKNNCGDSGDIDLVVKRSLDKGKTWTGLQLVWSDSTNTCGNPVPIQDQTTGNIILISTWNLGTDHEKQIMDGSSK
DGRHVYMLSSGDNGKTWSAATEITAQVKKTGWTWYATGPCHGLQVLKGKYAGRLVVPINHVETGTNQNFAHTIYS
DDHGKSWNLGNNTPQDKMNETTLAEISKGRLMLNMRNSDRTIKTRHTAISKDGGQNWANVQIDTTLIEPICQGSL
LSHFYRNNKPTLLFSNPANTKLRANLTLRLSKNDGKTWKYNMVLHPGPSAYSDIAVIDKQIIGCFFEAGYAKPYE
GIVFKAVNYSDLTK

>Sphingobacteriales_OJY82384.1 exo-alpha-sialidase [S. bacterium 40-81]
Bacteroidetes

MQNAKALYLKFNFCILFLFFCMETLQSQELNYLFKQDESGYKCFRIPAIVSTNNGTLLAFAEARKNSCSDTGDID
LVLKRSEDGGKSWSALEVVWSDGENTCGNPAPVVDKTNGNIILLSTWNLGTDHEKDIIALKSQDKRRVFMLKSTD
DGKTWLPAREITRDTKKEDWTWYATGPVNGIQVQKGKYKGRLVIPCDHIEAVSKKYYSHAIYSDDGGDNWKLGNS
TPQDQVNECTIAELSDGKLMLNMRNYNDTRVRQVSVSKDGGHSWADLKGDPALIEPVCQGSLLSYKQAGKKVLAF
SNPANKEQRINMTVRLSSDNGKTWRYSKVLHEGPSAYSNLVVLPNGNLACFYEGGIKNPYEGIVFREIPLSDIKG
K

>Aspergillus_XP_751452.2 extracellular sialidase A. fumigatus Af293 Asco

MQSMRFMILALLVQFLPAWAINDPAKSAAPYHDEFPLFRSANMASPDKLSTGIGFHSFRIPAVVRTTTGRILAFA
EGRRHTNQDFGDINLVYKRTKTTANNGASPSDWEPLREVVGSGAGTWGNPTPVVDDDNTIYLFLSWNGATYSQNG
KDVLPDGTVTKKIDSTWEGRRHLYLTESRDDGNTWSKPVDLTKELTPDGWAWDAVGPGNGIRLTTGELVIPAMGR
NIIGRGAPGNRTWSVQRLSGAGAEGTIVQTPDGKLYRNDRPSQKGYRMVARGTLEGFGAFAPDAGLPDPACQGSV
LRYNSDAPARTIFLNSASGTSRRAMRVRISYDADAKKFNYGRKLEDAKVSGAGHEGGYSSMTKTGDYKIGALVES
DFFNDGTGKNSYRAIIWRRFNLSWILNGPNN

>Metarhizium_XP_007806611.1 extracellular sialidase M. acridum CQMa 102]
Asco

MKASVLFVWLTTTLGGSLAAAVVKDIAKDVPPAHKEFVLFKSSNMAGADKLANGVGFHSFRVPAVVTTSTGRVLA
FAEGRRHDNRDLGDVKVVMKRTKTVNSHGSNPSDWESLQVVTKDDGVWGNPTPVVDGPTIYLFLSWNNAKYSRDG
EDRLPNGKKTKKVDGSWKSRRHTYLTQSTDDGQTWSEPREMTRQLTPGGRGWDVVGPGNGIVLTSGEVVVPAMGR
NIVGKGTPGQRTWSYKRLKGAGAEGTIVQTPDGKLYRNDRAGKDEEYRKIARGTLAKFGSFTLDKGLPDPDCAGA
TLLFNRADDKGPARVVFLNSADKNTRRAMRVRISYDKNAKEYSHGRKLSDAPVSGAGNEGGYASLTKTADSEIGA
LVETDFDQTGGSKDDYHAIIWRKFNLSWVLHGPKK

>Arthroderma_XP_002847861.1 sialidase [Arthroderma otae CBS 113480] Asco

MGVKQWLVSLAVVALSATVIQAKVDDPAGKAAQYHKEYALFKSENMPSPDKLSSGVGFHSFRIPAVIRTNTGRIL
AFAEGRRHNNRDYGDINLVYKRTKTPTNNGENPSDWESLREVVGTGPHTWGNPTPVVDGNTIYLFLSMNDGAYSQ
NGNNVLLNGEKTKKIDSTWVGRRHLYLTESIDDGNSWSKPVDMTKTLTPDGQAWDAVGPGNGIKLTSGELVIPAQ
GRNIIGTGPAGNRTWSMQILKGAGSEGTICQTPDGKLLRNDRPGPKGHRSVARGTLSGFGPFATDNGLPDPACQG
SILSYNRDEPARTIFMNSASVDRRTAMRDAAKFNYGRDLQDAPLKNVGNEGGYSSMTKTSDYKIGALVESDWYED
KGGEKSHRAIIWRRFNLSWIINGPHN

>Arthrobacter_OOP63695.1 [Arthrobacter sp. SRS-W-1-2016] Actinobacteria

MAAAAALMTSTVVPAYAALDPFPSPNVPSNPPGSYVEQVLASNGDNAIDPVLGKYYRIVALADLGDGVLLASYDG
RPDGGDSPSPNSIVQRRSTDGGKTWGSPTFIARGQLSSAGVQKYGFSDPSYVVDKVTGTVFNFHVYSKNQGFAGS
VLGNDDSDPNVISSQVSVSTDRGLTWSTDPGNQPTLPVPSSYPAGSAYAGFAGPLITATVKPNGSTINGVPNVGG
VVSMFASSGEGIQLKYGAHAGRLIQQFAGTIVQPDGSRQIQAYSVYSDDHGKSWNMGTPTGTGMDENKTVELSDG
RVMLNSRDSTGANPHFRKVAYSSDGGVTYSTPVAETQLPDPRNNGSITRMYPDAAAGSADAKKLLFSNSPTPTAG
GARVDGTVRYSADDGKTWGSSRVFKAGSMSYSTLSALSDGTFGVLYEGDNNTITFGKFDTAWLSPVAAVVVPGAV
TVTNGGSAQLSVTVTNNDSSPLPASTVSLDALQDWTSTTADLASLDPGASATVQLTVAAPSYAKAGAVPLTARLN
VGGASITAPVSVTVAGGSAGNVVGAYIFGARNDAGRDLASHPYAAGSPVPYKFRVYSTGNVTESVVPQSGNFQPF
LPPGSGNCRFSALGVWGNYLCGTPQHTVTADEAARGFFVPSTVWQVTGAGAATQNYTVTGDEVDLLVRNPQLSGS
VVSTFNDVDGDGFAGPGDTVTFAYTVTNTGNVALTDLSAAALGLSKPTLSVGESVNLASAYTLTEADIAAKQLPV
TTIPVSANNGAKLASLQLDRQAVALKVKPAMPGSEPALASQELIGAHPPLDLGLGADKYRGGQTVTVHNVEYGQW
YYVYLNKRSYRLGWFFPTQQNTLSFVLPKDVKNGMDSLVVLDSEGHQVSFGDFQVTPFGSK

The following sequences were used for MSA but were not included in the tree because they are too divergent

>Janthinobacterium SDA58235.1 sialidase-1 [Janthinobacterium sp. 551a]

MSTVLPIQSLQRLAAACVLGLGLGLGAAPVAQAAISASTPFPAADASYGCYRIPAVVTLQNGTILAFAEGRPSGC
ADFGNIQVVMKKSLDGGSTWSALSVVASNGTLQAGNPVPVLDTLDPAHPGGRLFLFYNTGNASESTIRNGGAGVR
EQWVVTSTDGGATWSTPSNITAQTAKIGAAPYNNGAGWRTLAMGPGHGVQMRSGRIVVPGNFTAGPPQTGYADGR
AYVFYSDDHGASYQIGNDTGYPSANESTAAELSTSQLMLNSRDQSGLTKKRIVSVSSDGGANFSAGFANATLIDP
VCEGSLLNLGWNGKQYLLHANPASTTSRNNLTVRASKDDGQSWPFSLLITAGASAYSDLTQVDAGNIGILYENGS
NGIRFMKIPLSTVITAP

>Bathycoccus_XP_007512415.1 prasinos]

MKKKREFVVVLLFLVFVYFFLASFFNSSSASSSSFKSRSRGNGDGFNIQEKKTGTNGDGLFFSSKNNNNKDNNKD
NEENLRALEEIFLEEESQSSVSADPLDLQFLGLQSSLGGASSSCKFQFEIASWRETRLFFALHPECRHLDKCESE
LPSQTTLSKQVFDSGGGGGSSVDSRDEKNEMIIKHSTYVKRFDEDLRYAHMATIAKMKDERMILLYQAAPAAKKD
VDGSDGGGGDDGNGGGDGGDATYYEYKLATEGLQDQHIEYTQSKDLEGKRWTPPVKLPIYNDAAVWSPVVHIDNN
QVYVFYSESTGCKKAIPCKPPQCPKGEVCEVDSHAEMCHHTPKSLWVPGGDIKYIKSIGDPAENKWTKAVTILSQ
DEGGGIPKVIANSLIVMKKTGHWVLPYWREQQNAIEDGTCTRKPEFKGSSGPDCRWNKRSRCMTGAQPFSGVLVS
ENRGKTWQPRGQITQSNTSLIEGSIAELNDGRIIQVFRTRVGCLYKSYSKDEGKSWTEPEPMPVPNPNSKVFLMR
LEPNGELLLAFNNMKNQYRSRRGATKCRACRTHLHVAISRDGDGNEWETIARLEDEIGYEAPRIHYPYMAQKDAS
SVLVAYTRFYLGKRKGLTSLDQGVKVAEIDLKSIV

>Bathycoccus sp. FQSR01000356.1

MPPKGAVLGGGRRKKRDFFDGGVDDRRRFRRFFFSLLFVVLERIASVAIGLASVAIVVDDFWTVSSTIGNDITKA
SSSSFKASSHKYAFTPTNDKMEYAWAVKGRALPSETRDAKLGTTEPEFKYTHMAMPTVLPDGSIAVAFQASPTEY
EGSIRQALYWAISKDDGLTFSTPKMIAKDDQLPVWTPVLKVSGKRVFLLYTVSSRKCRYYDQSRNALRHSPGGDV
MYKVSDDNGKTWSSGQVVLSYGSEDGMPKVIANTLVELEDGSWVLPFWREPGKTCPQIKSEVKDASLLRKGSAGV
IRSTDQGLTWQVFGNLTATKGESWLIENTVVERKSTTKNKKEKNNVLVQHFRTKAGVAFVSLSKDNGKTWTEAAE
TTLPNPNSKMHTVRIDDNIEDDSSTSFFVAX

>Trypanosoma_grayi JMRU01000526.1

MPRHTRFVFLLLSLLLAFDGIRPTHALGVNSRRVELFARGKSTVPFEQDDGEVVPRVAHSFRIPSLVEVDGVLVA
IGDARYLSDDDNAFIETAVKFSVDGGSTWETQIAIKNSRVNSERSRVLDPTVIVKGNKIYVLVGSYNITSTYWTW
QGDGSDWEPLLAVGEVNKTTVGGKPNATITWGKPVSLKSIFPKTIAGAPSAQFLGGVSRAIVLNDKTLVFPVQAT
NEKKQITSMIMYSDDDGASWSFGSGNTHIGCSEPSLVEWEGKLIMNARVDNAPRKVFESTDMGETWSEAVGTLSR
VWGPSPSRTEPGSQGSFITVTIEGVRVMLFTVPLNFKGRWLRDRLHLWVTDNRRIFDVGQVSDGDEDSPYSSLLY
TKAGKLYCLHEMSHDRVYSLIFAELIDELELVRSVVRTWKAQDEYFSKTCSSTASEALPSDGGCAAAVPTGGLVG
FLSGNSDDHHWQDVYRCVDANVTGAEKVTNGLLFKGHGAGALWPVNLQGQNRRYEFMNNAFTLVATVTIREAPAC
EKNPLLGVSMTVGGGKRVLGLSYGKNYQWRPVYGGVLMNAEETWELNKPYQVAITMGADTSSVYVDGKVLGGAAQ
PLPPALKVRDFSHIYIGGLADGSKGPSSSVTVANVLLYNRVLSPHEVETLSLYKDSIAVAPDARLEAPSPFAIPG
AETAVMPKTDGGASPLPQVPQSPPDAVPRATSETVTPAVNHGGGRRENEDDGPLAEVIQCPEADREESSLDSTVD
VDASRSDEYDAAQAHARMLLLLLLGVWGLAALY

>Emiliania_XP_005775390.1 E. huxleyi

MLRLMILSCLCIAAASAQAPPNPCTAAECTPIFYPGLNGSQCYRIPSIIATHRGTLLAFAENRLGGCGDQGMHNL
VVRRSSDNGATWGPLITVFAGVVPCPGCPAAVSNPNPVEVTFPNGTRSILLAFDTMNNPSVAHHGLDMTMWSHDD
GVSWVGARATEYPPQPNVGSLIGPAVGLQLQAPEARGQIVFWITSGFGFTSGFLAVSKDYGETFTASQHVLKEPG
SECSIAFAAGPGNSTLIMNCRSGQDSRRAQLYWSLLDNGTYASTEPTYPTQLTDPGCQGSILSAAAGAGSVPGSG
SGAGAATHVLYTSNAASTSARERMTIHRSDDGGVSWSAGQVIHAGPSAYSQLVQLPNGSMAVLFEAGVRGAYETI
SFAPFAWTPPHTGPDFY

>Reticulomyxa_ETO10819.1 R filosa

MCGIDPQSMVPKGLQIRQEDTNTRGKNKNVSTAATTNTNTNTNTNTNNSNNNNDDNNNSKKKEDVFTHLMSQLPL
LNSILSSLPEPLAEMHDLMTPTNSMPWNWNVNTNTDTNINMNMNMSMNVKSSSIKAKVNPPMATTMMKPTTTNSK
VKPNGIGNGNGNGNVSAERKRASNNGKDPVKQMDSSLERLTWDTMDSSVLECSVAPIYHPATLYRAARRAHENII
RKRYNSQLCCEFVTQLSNHWPSFSFGPDETTESDSLCSQLVGDVKRKYRRNKTTSVNLDLSLNELSLETIGNESH
HLDNGTIIQRKNVSLVTLCAQLSLAFVLYSSQHNANCNTKGSIAVWNKDMVENIAFAMELSLCKCLDYHRIHLIM
MALLDKNEEWSWNACCEIVRRYGRRLEVTQLSCLHLYLDRMRLRSQVNLASDQELRIQAIIAKTVRCLAHPSSST
DNTTSDGCVSPQSPFHSSSKQHYPFESFHTRATTSPGLSFPQKKFNTAVRGNTKIAQFNFFFIS

3 - CMP-Sialic acid synthetase (CMAS or CSS)

>CMAS1_Dre

MDAVNENGKRAMKDDSHGNSTSPKRRKSRHISALILARGGSKGIPLKNIKMLAGVPLIGWVIRAAVDSNVFNSVW
VSTDHEEIAKVALAWGAKVHKRSPEVSQDSSSSLDTIREFSRQHREVDVICNIQATSPCLHPKHLTEAVELITKQ
GYDSVFSVVRRHNFRWKEVEKGGDCSTEPMNLNPACRPRRQDWSGELCENGSFYFAKKELIEQGLLQGGKKTYYE
MKPEYSVDIDVDIDWPVAEQRVLRFGYFGKDKPEVVRLLLCNVSGCLTDGQIYTSASGEEMVSINIRDQIGISML
KKEGVKVILLETYPIAKALAVRLSERMGCPLLHHMDDKLKEVERIMVEEKLEWKEVAYLGNDEADVKCLELAGLS
GVPVDAPTVALNHTKYTCHNAAGHGAVREFAEHILLLKKKAKSQMEQDRICRDAF

>CMAS2_Dre

MEGGRCAQVSSVPASPSSGHRHRAALILARGGSKGIPLKNIKNLAGVPLIGWVLRAALDSDVDSVWVSTDHDEIE
RVAKLWGAKVHRRSPEVSKDSSSSLETIQEFIRLRPEVDVICHIQATSPCLHPHHINEALQKITHQGFSYVLSVV
RRHQFRWEELQENEDRNPKSFNINVAQRPRRQDWPGELYENGSFYFSTRKAWESGLTELGRIAYYEMPPEFSVDI
DVDIDWPVAEQRVLRFGYFGREENAAVRLFLCKVSGCLTNGQIYMSVSGEDLVTINARDVAGIQMLQKENIEVIL
ISSVNEPLSRGVLEKVSQRAGCGLSFTEQRNALEMQKLMDKRKLHWDQVAFMGSESEDVEIMSQAGLNGVPSDAP
VAELIAAKYTCQRAGGHGAVREFAEYILSMKRKSSREENHERIDKHNF

>CMAS1_HSA_Q0E671.1 CMAS + HAD

MDAVNENGKRAMKDDSHGNSTSPKRRKSRHISALILARGGSKGIPLKNIKMLAGVPLIGWVIRAAVDSNVFNSVW
VSTDHEEIAKVALAWGAKVHKRSPEVSQDSSSSLDTIREFSRQHREVDVICNIQATSPCLHPKHLTEAVELITKQ
GYDSVFSVVRRHNFRWKEVEKGGDCSTEPMNLNPACRPRRQDWSGELCENGSFYFAKKELIEQGLLQGGKKTYYE
MKPEYSVDIDVDIDWPVAEQRVLRFGYFGKDKPEVVRLLLCNVSGCLTDGQIYTSASGEEMVSINIRDQIGISML
KKEGVKVILLETYPIAKALAVRLSERMGCPLLHHMDDKLKEVERIMVEEKLEWKEVAYLGNDEADVKCLELAGLS
GVPVDAPTVALNHTKYTCHNAAGHGAVREFAEHILLLKKKAKSQMEQDRICRDAF

>Leucothrix mucor WP_022954484.1 mannose-1-phosphate guanylyltransferase

MKAMILAAGLGTRMRPLTNHAPKPLLPVGGKPLIVWHLEKLQACGFTEVVINIAWLGGMIPEALGDGSQLGLSIQ
YSDERDEPVLETGGGIIKALPLLGDEPFLVVNGDIWCDYNFQADFKLQEGDLAHLVLVDNPPHHLEGDFLLQNGR
LLETGEQKLTFSGIGYYHPDLFADLEYGKRALAPVLRDGMREGKVSGERHSGVWLDIGTPERLSELDKSLL

>HSA_GTPmanP Q9Y5P6.2 Mannose-1-phosphate guanyltransferase beta

MKALILVGGYGTRLRPLTLSTPKPLVDFCNKPILLHQVEALAAAGVDHVILAVSYMSQVLEKEMKAQEQRLGIRI
SMSHEEEPLGTAGPLALARDLLSETADPFFVLNSDVICDFPFQAMVQFHRHHGQEGSILVTKVEEPSKYGVVVCE
ADTGRIHRFVEKPQVFVSNKINAGMYILSPAVLQRIQLQPTSIEKEVFPIMAKEGQLYAMELQGFWMDIGQPKDF
LTGMCLFLQSLRQKQPERLCSGPGIVGNVLVDPSARIGQNCSIGPNVSLGPGVVVEDGVCIRRCTVLRDARIRSH
SWLESCIVGWRCRVGQWVRMENVTVLGEDVIVNDELYLNGASVLPHKSIGESVPEPRIIM

>Thelohanellus_GTPMan KII62015.1 Mannose-1-phosphate guanyltransferase beta-A [T.kitauei]

MTKALILAGGYGTRLRPLTLTRPKPLVEFCNKPILFHQIDALVQAGVTEIVLAVSYMCDMLKKEVDVYSRKRGVD
VVFSKEDEPLGTGGPLALAKEYLKGDKPFFVLNSDVICDYPFKEMMTHHLIVGAEGTIAVVKVDDPSKYGVVVSD
ETSKILQFVEKPKDFISNKINAGIYLLNPSVLDRIPLRPVSIEKETFPQMALDQTLYCFRLQSFWMDIGQPHDYL
MGSKLYLQHLKLNNPKNLGHCSFEHKNGLIHNTVTFGDGCFIGENVVIGHDCKIGEGVHLDNCVILSNTTIKSHG
YIDNSLIGWRCTIGEWARIQNCSVLGENVVVQDEVLINGCKVLPQKCISESLYTSKIIM

>Nematostella_GTPman XP_001637465.1 predicted protein [N. vectensis]

MKALILVGGYGTRLRPLTLSKPKPLVEFCNKPMLLHQVEALVQAGVKHIILAVSYRAELLEKEMKEQEQKLGIKI
TISQEKEPLGTAGPLALAHDYLTVNNEPFFVLNSDVICDFPFREMVEFHKKHGKEGTIVVTKVEEPSKYGVVVYN
SETGCIERFVEKPEVFVSNKINAGMYIFNPSILNRIEMRPTSIEKEVFPFMAKDSQLFAFDLQGFWMDVGQPKDF
LTGMCMYLNSLRKKSPELLHEGPGIIGNVLVCPTSKIGDHCRIGPNVVIGPGVVVQDGACLSRCVVLKDATIRSH
SWIQSSIIGWKSVVGQWVRMEGVSVLGEDVIVKDELYINGGRILPHKSIGASSPEPQIIM

>Hydra_GTPman XP_012558341.1 mannose-1-phosphate guanyltransferase beta-like [Hydra vulgaris]

MNALILVGGYGTRLRPLTLSRPKPLIEFCNKPMLLHQVEALAKAGVKHVILAVSYLSDMLEEELKKEEEKLGIKI
SMSHEREPLGTAGPLALARHWLEESTEPFFVLNSDITCEYPFEEMINFHKKHGKEGTLVVTKVDEPSKYGVVVFD

ASTGKIENFVEKPAEFVSNKINAGMYIFNPSILKRILPKPMSIEKEVFPFMAKDGELYCFELKGYWMDIGQPKDF
ITGTCLYLNALKKNSSDELYKGPGNVGNVLVHSTAKIDDDVQIGPNVVIGPDVIVEKGACLSKCVIMKGTLIKSH
SWINNSIVGWKSSVGKWVRMEGVCVLGEDVHIQDEIYLNGAKVLPHKSLAASIPEPNIVM

>Amphimedon_GTPman XP_011407597.1 mannose-1-phosphate guanyltransferase-like A.queenslandica

MILAAGRGERMGELTLERPKPLLEVAGISLIEHQIRHLQRAGFRDIVVNLAWLGGQIKERLGDGSRFGVRIDYSH
EPEGALDTGGGIRRALAMLGDGDFVVINADVWTDYPFERLRSSAGQGDAFIVLVDNPPHHPHGDFGFLPDVVSGS
GKEDGERTEGQAATEIVRGQVIEASDEKRLTFSGIGRYSPRLFAERKEGRFPLSMILREAMRAGTAHAEHYRGEW
ADIGTPERLAAIDRKVRCEIASGVLRPRVI

>Drosophila_GTPman XP_017873577.1 mannose-1-phosphate guanyltransferase beta [D.arizonae]

MCGSPNTAAGSGARALILVGGYGTRLRPLTLSTPKPLVEFANKPILLHQLEALVDAGCRQVILAVSYRAEQMEQE
LKVEADKLGVELIFSHETEPLGTAGPLALAKPLLSASSEPFFVLNSDVICDFPFKQLMQFHRNHGKEGTIVVTKV
EEPSKYGVVLYDELGCITNFIEKPQEFVSNKINAGIYIFNPTVLDRIEVKPTSIEKEVFPNMAQQQELYAMELSG
FWMDIGQPKDFLTGMCLYLSSLRQKQSSKLYTGPGVVGNVLVDPTAKIGEGCRIGPNVTIGPDVVIEDGVCIKRS
TILKGANVRSHSWLDSCIVGWRSTVGRWVRIEGITVLGEDVIVKDELYINGG

>Physcomitrella_GTPMan XP_001782193.1 [P. patens]

MKALILVGGFGTRLRPLTLSVPKPLVDFANKPMILHQIEALKAVGVDEVVLAINYQPEVMMNFLRDFEKSLGIKI
TCSQETEPMGTAGPLALARDKLDDGSGESFFVLNSDVISEYPLRQMIDFHKKHGGEATIMVTKVDEPSKYGVVVM
DESNGCVERFVEKPQQFVGNKINAGIYLLSPRTLDRIELRPTSIEKEVFPKISAEKSLFAMVLPGFWMDIGQPRD
YNTGLRLYLDSLRKKAPEKLASGSTFLGNVIVDESAKIGEGCLIGPDVSIGQGCTIEAGVRLSRCTIMRGVRVKK
HACISGSIIGWHCTIGQWARIENMTVLGEDVRVSDEIFTNGGVVLPHKEIKASILKPEIVM
QVLPHKSIAASVPEPQIIM

>Arabidopsis_GDPman CAB79775.1 GDP-mannose pyrophosphorylase like protein [A.thaliana]

LKALILVGGFGTRLRPLTLSMPKPLVDFGNKPMILHQIEALKGAGVTEVVLAINHQQPEVMLNFVKEYEKKLEIK
ITFSQETEPLGTAGPLALARDKLVDESGQPFFVLNSDVICEYPLLEMIEFHKTNRAEASIMVTEVDDPSKYGVVV
TEEGTARVESFVEKPKHFVGNKINAGIYLLSPSVLDRIELRRTSIEKEIFPKIASEKKLYAMVLPGFWMDIGQPK
DYITGQRMYLNSLREKTPQELATGDNIIGNVLVHESAVIGEGCLIGPDVVIGPGCVIDSGVRLFGCTVMRGVWIK
EHACISNSIVGWDSTVGRWARVFNITVLGKDVNVADAEVYNSGVVIEEQGL

>Bartonella henselae YP_033089.1 3-deoxy-manno-octulosonate cytidylyltrf alpha prot bact

MALEPIILIPARIGSTRLPQKALAEIAGKPMIVHVAEQAKKAAFGRIIVATDHNNIAKVVTAYGHECIITCRDHK
SGSDRIYEALTHIDPERRYNVILNVQGDLPTITPHEIISALRPLENSLTDIATLGAKIVEENEKTDPNIVKIIGT
PLSHNRFRALYFTRATAPYGDGPLYHHIGIYAYRREALEKFVALKPSPLEQREKLEQLRALEHNMRIDVEIVDTI
PLGVDTQRDLERVRKILA

>Campylobacter jejuni NP_282477.1 acylneuraminate cytidylyltransferase (flagellin modification)

MAEILCTICARGGSKGVKNKNIRKINKLEMIAYSIIQAQNSKLFKHIVISTDSDEIASVAQKYGAEVFFKREAHL
ANDRTAKLPVMRDALLRSEEHFKTCFETLIDLDASAPLRSSLDIKKAYESFVENDNSNLITAVPARRNPYFNLVE
IQNNKVVKSKEGNFTTRQSAPKCYDMNASIYIFKRDYLLENDSVFGKNTGLFVMDESTAFDIDSELDFKIVEFLI
SLKNLSPKDF

>Bradyrhizobium lablabi WP_057858225.1 flagellar modification protein B

MKSICTMCARGGSKGVIGKNARELLGKPLLAWSIEQARQTGLFEAIAFSSDSDLLLETALQAGADLAVKRPDEMA
TDTAPKIPAIRHCLEQAIARTGITPDVFVDLDVTSPLRLPSDIAGAVELLNRSGARSVITGAPARRSPYFNLVEE
RADGSVGLSKSADPPIVRRQDAPRCFDMNASIYVWRVAPFLESPAVFYPDTQLFEMPEERSVDIDSDLDFTLVEL
LLRKRLALPETQS

>Tepidicaulis marinus WP_045444633.1 flagellar modification protein B

MRRICTILARGGSKGVPGKNIRPLGGKPLIAHSIVQALASGLFETVAVSSDDEAILRAAEEAGADELVLRPADLA
SDTAPKLPAILHCVEEVEKRHGHSDVVVDLQPTAPLREPSDIQGAVALLEESGAPNVITGSPAKCSPYFSLVEER
ADGTVGLSKPVDPPLARRQDAPRTFDMNGSVYVWRREVLTADMKLFQPGTRLYEMPEERSVDIDTALDFEFAAFL
MGRRRA

>Pelobacter carbinolicus WP_011341002.1 acylneuraminate cytidylyltransferase

MEKIIAFIPARGGSKGIPRKNIKNLAGRPLIYWVLDAATRCDLIDKVYVATDDAEIAEVVDRFGSERVEVVSRGS
ETATDTASTESAMLEFSRQRNFEHMVLIQATSPLLEAGDLDRGIRKYLNNKADGLVSVVRQKRFYWQVADEGRAV
PVNYDPCSRPRRQDFDGILVENGAFYVSRKDSLLKSGSRLSGHVVTYEMPEATYFEIDDEVDWQIIEGMLANRKI
SVTSEQSGKLKKVKLFLTDVDGVLTDAGMYYGESGEELKKFNTRDGKGIELLRNHGIKTGIITSEATRIVLNRAK
KLKMDYVFTGIKDKLSVFNKLLLETGIDASETAYIGDDINDLEVLAAAGLSASPGDGVASVKRVVDYVCDKRGGE
GCVREFVEKILE

>Nitrosomonas sp. SDY82160.1 N-acylneuraminate cytidylyltransferase

MRWVAFMPLRANSKSIPDKNIRSIAGRPLFAWSLEQAIVSQCFNEIYVATDSSRIRRMVLDEFSSGVTVLDRSAE
TCTDTASTESALLEFQQRISFDVVGLIQATSPLTQADDFHAARHKFVAENLDSLLTAVSSKRFFWTQSGTPLNYH
PAKRPRRQDFEGWLMENGAFYLTRAKVLEETGSRLGGRIGIHEMPAETAIEIDDEADWIVVEQLLLRQKLASIQM
GASQIKAFILDVDGTLTDGGMYYGADGEALKKFNTRDAHGLQLLRENGIRVCVFSSEDSPAVAARMKKLNIAEYY
PGIRNKLAALERKIKLWGISLQDIAYMGDDLCDLECLSQAGVALCPADAVPEILQRAHYVCTRPGGHGAVREACD
LILRIKPRTIISK

>Shewanella denitrificans WP_011495733.1 pseudaminic acid cytidylyltransferase

MNVAFIPARGGSKRIPRKNIKLFKGKPIIGYTIEAALQSGCFEQVIVSTDDDEIAAVAESFGAIVPFRRPAEFAN
DHATTMQVVIHGIDWMLRNGLAPKKMCLMYATAPFITPVLLQESLALLDANPNKHYCFAVTEFAAPIQRGFSVSP
AGDIEMFQPEHLTTRSQDLIKAYHDAGQFYWGKTDAFLKNLPVFSAHSVPYLLPQHLVQDIDTLDDWHRAEWLFA
MQQKEKI

>Strongylocentrotus purpuratus NP_001119780.1

MSDSSDSNRKSGHFACLILARGGSKGIKLKNIKALAGQPLIAWVLRAAIDSGEFDSVWVSTDHADIARISKEWGA
QVHMRSPHTARDQATSIEAMQEFLKEHPEVKFVANVQCTSPCLHPSHLQRTCHMIRNLGYDSVFAVNRRHLFRWT
ETPIDQAVSTKAENLDPAKRPRRQDWAGELYENGSFYFATRELLMAGLFQGGKVGYCEMQPEYSVDIDTDIDWPI
AEQRVIKFGYFGKTKPQGVKLVVFAADGVLIDNQINFTGTGEEVRSFSLSDSIGIKHLREKGVEVRVIADEENAV
TTKLAARLGIAMATESKNKVDLLNEWRKGLDLDWSQVAYMGQDTPDLDCMKTVGIGGAPVDAQNEITTTAKFVAK
SKGGKGAGREFCDHLLLVMEKAKSAAEDRKI

>Aureococcus anophagefferens ACJI01000583.1 CMAS

MAAHVDAKPHALILARGGSKGIPRKNIKPLNGVPLLVYNVKAALASGVFGRVVVSSDDDEILAVAARAGASTHAR
SAASAADAASSEAGVFDYVDATPSCAVCCLVQCTSPLTGAEDFAAGHARFVAQGADSLVTVVRAHRFLWKQAADG
SAAPCNYDPVKRPRRQDWDGELIENGAFYFFTVAALRASGSRLSGRVVAHEMPEETLAEIDTLTDWQIVEGLAAD
KFKDAPKAARRAPGPAPREPRRRAAPRRTTSARRRPGPELSFEL

>Condylostoma magnum CVLX01001070.1

KAMTTIATICARGDSKGLPGKNTKNLLGKPLIAYSIEQALQAKEIDAVYCSTDSQQIADIAQQYGAIIPALRPKE
LADDYCSKLDVINHLVALVKPNHNDLSRIIDLDVTSPLRDIIDITACVELLDDETDLVITGYEADKNPYFNMVEY
DEDGSIKLVKPPLYGVTGRQRAPKVYAMNASIYAWHASSLHKGLWSGKVKLHEMPRSRSIDIDDEIDFELVEYFM
RKKIKSE

>Prorocentrum minimum JXLM01000002.1 CMAS

ILARGGSKGVPGKNIRPLLGKPLIVHSIEQAQASKLFEHIAVSSDDQAILETARAAGVQHLVQRPPELATDSAAK
LPAIRHCVETVEKDIGELFDVVADLQPTSPLRLPSDIVGAVALLDDGDVDNVITGSSAKCSPYFNLVEERSDGSV
GLSKPTDPPIVRRQDAPRTFDMNGSIYVWRRATLMRDVGLFLEKTHLFEMPEERSADIDTELDFQFVEFLAERSA
AH

>Chromerat velia ARZB01112074.1

ENASDTASTEAAMLEFCEKAEADFDVFCLLQATSPLTQRTDIDRGLEKLQKEGLDAVLSVVNTHRFTWSKDGKPQ
NYDFNNRPRRQDFEGLLIENGAVYCTTKNALLASKNRLSGKIGIIEMSEDTLVEIDSETDWQVVEQLLISHFKQN
KSAQRINYLILDVDGVFTDGRVTFTNEGEFSKVFDMRD

>Chromera velia ARZB01004620.1 CMAS

AVIPARGGSKGLPRKNVRPLAGLPLIAYSIRAGQAASSVDRLVVSTEDAEIAAVARDSGAEVVERPAALARDDTP
TRPVIEHAVELLAGGGYVSDAVLTLQPTSPLRTARHIDEAAALFAAAPSADSLVSCVRVPHIFHPNSVMRLTEDN
TLEPYLGEPTVTRRQDKGIAYARNGAAIYITRTDRIKDFVFGGRLLPYEMPLEDSIDIDGEDDWRLAEERLKAQ

>Paramecium biaurelia JPFL01001250.1 CMAS

DCMNILFIIPARGGSKGLPGKNIKPLNGIPLITHSVQHALASKYPKKVIVSTDDAKIAAVAKEAGAEIVVRPADI
SGDTASSESALIHALTTVEQQGYIPDLIVFLQCTSPIREMDDIDNAIELLLKEKSDSLLSASPNHRFIWKKGPNG
FYSVNYDYNKRQRRQDLEPEYVENGSIYIFKPWVLKENNNRLGGKVSLYLMSDESSYEIDSAMDFLIIENLMKGL
E

>Micromonas commoda XP_002506929.1 epimerase2 + CMAS

MPACPNTEADKASLYAPDKCTDMKTTLRRKICVVTSNRSDWSKLKLVAINLRKLCTSQDNQEASDIQVDIICLGS
HLLHELGATKNIVKEDFPNAYELHTLVAGDSVESMTDSVGFGIVKLTSLLCALKPNIVLVHGDRFDAFCAAIAAN
MLNLTIAHVEGGELSGTVDGTLRHAITKLSHLHFTCTPEAARRIRGMGENPASIFVTGCPSYESLFAVSATCWED
EKMDQFFNGTPFKLKPNKFILVIMHPVTNDLEESNTLYGSLLSCLFSRKTPTVMFYPNVDPGNKSMIQTLHKHQK
ADPASTSWLRLVTHMPHAKFTALMRHASAMVGNSSAGIRESCVFGIPTLNLGSRQEGRRVPANVTTLVKPSIRSI
DCWFDNELGKRYAQSTMYGFPDSAKRIAHHLSRIDTSAGQLKQFWEPRYALLPPLQPRQYVSRTQAILADSTSSP
TSSPIGRCKILGLITARGGSKGIPGKNIIDLNGKPLIQYTIEAALSSKQLDRVILSTDSDEIAEVAQNCGCEVPF
RRPSELAADDSSHLACIVHALNILRETECFVPDFVVILQPTSPFRKSIDIDSCINIMLTSSCDMVLSVCESSLNL
SKNFYFAADGTLSPFAESTAEIDYTPRQKLLKTYAENGAVYVLRTQSLLYPPDNAPNVGSFRSADTKGYEMPVER
SLDIDNPFDLHVARLLMAKPF

>Chrysochromulina sp JWZX01000205.1 CMAS

MTKSPPPVLTPRAGKVVNTSEPMPKIVAVIPARGGSVSIPKKNIKLLLGRPLIDWVIKAARDSKIFAEIWVSTDD
DLIAASAIKCGALVHRRAAHTATSTASTESALKDFADAHPDYEYMCLIQATSPLIRPEDFVEAMRIMRDQKADSL
VTAVRAHRFLWSVDPTTKVAKAKNYEPLKRPRRQDWEGELVENGAFYYYTKAHWDATGCRLGGKLVLYEMAEHTF
VELDSLVDWQMVSHMAYDFGYFPEDAPRQAPDMGGTAQSSTFAVGLATGFALAAGLAFLMGRK

>Paracoccus denitrificans ABL71620.1 acylneuraminate cytidylyltransferase

MKIAIIPARGGSKRIPRKNIRPFGGRPMIAWPIAAARDSGLFDHVIVSTDDAEIAETALAHGAEVPFPRPVELAD
DFTPTRAVINHAIRAMEEAQGREVTLACCIYATSGFLDASDLVGAHGLLAAEPAAGFVFAALHFPHPPQRALCRG
PEGGVAMLHPEHAGTRSQDLCETFHDAAQFYWGRRDAFLSGAPMFSTLARPWILPRDRALDIDTPEDWDWAERLL
ALRQAAGEIPA

>Thioclava sp. WP_078540131.1 pseudaminic acid cytidylyltransferase

MKLAVIPARGGSKRIPRKNIRPFGGRPMIAWSIAAARTSGLFDRIIVSTDDREIAETARALGAEVPFFRPANLSG
DYTGTVPVIAHAVDWQNTQGEPAAEVCCIYATAPFIEVDDLRRGLVILQESGADFAFSVTRYAAPIQRALRVRQD
NRVEMFDARQFNTRSQDLEEAWHDAGQFYWGRAEAWLSGKPLFGTHAAPVVLPRHRVQDIDTPEDWDRAELLLRA
RGFQ

>Thalassobius WP_058314783.1 pseudaminic acid cytidylyltransferase

MTEAIAIIPARGGSKRIPRKNIRDFAGKPAIAWPIEAARSSGLFSSVVVSTDDQEIADCATGFGANVPYMRDASL
ADDYTGTTDVIRDAVQRLKLAPDTPVCCIYATALFVVPKDLAKGDDLLQNGATWAMSVCEYPTPIDRAYRRDGDQ
LVPRDPSKMPMRSQDLEPAYYDVGQFYWAKAKTWLDPEARVWDGARGVEIPALRAVDIDTESDWQRAEFLMSHLQ
DQS

>Leucothrix_KDO mucor WP_022950774.1 3-deoxy-manno-octulosonate cytidylyltrf

MKTILVIPARYNSTRLPGKPLLSIAGKPMIQRVHECAQQAGFDNIIIATDDERIAEVCASFSADVCMTNEAHETG
SDRLSEVVALRGFDDDDILVNLQGDEPLTPSVNLHQVAQNLVDHPEAMIATLCTPIVDVEDFTNPNVVKVVTDNA
GMAMYFSRASIPYQRDPSLDVTDFAFRHIGIYAYRAKYLRDFVKMESCQLEQLEKLEQLRAMWYGTRIHVDVAKE
IPGAGVDTAEDLAAVENVFLKRLAS

>Guillardia theta XP_005840663.1 CMP-KDO-Synthetase
MRPITLLPTRALSTLGAHAAVIIPARMASKRFPGKPLALIGGKPLVQRTLERVKRARHVDEVFVATDDQRIANLI
EGIGAKCIMTSHECESGTERVREASQQLSAEFDIIVNVQGDEPLIDPAHVDKLITHMKKNQADLVSTLACPIRSM
KDFKSPNVVKVVFGLQNQALYFSRSPIPYLPSDFKGPDEEKSQENASDQHRPFKHLGIYAFRRNFLEL

>E_coli AAA83877.1 CMP-KDO synthetase

MSFVVIIPARYASTRLPGKPLVDINGKPMIVHVLERARESGAERIIVATDHEDVARAVEAAGGEVCMTRADHQSG
TERLAEVVEKCAFSDDTVIVNVQGDEPMIPATIIRQVADNLAQRQVGMATLAVPIHNAEEAFNPNAVKVVLDAEG
YALYFSRATIPWDRDRFAEGLETVGDNFLRHLGIYGYRAGFIRRYVNWQPSPLEHIEMLEQLRVLWYGEKIHVAV
AQEVPGTGVDTPEDLERVRAEMR

>Zea mays CAB89846.1 CMP-KDO synthetase

MPICAPSSDSSASASSGLGARVWVLHGLALGAAAAAAAVAYLYRRPTGFRSRAVGIIPARFASTRFEGKPLVPIL
GKPMIQRTWERVMLASSLDHVVVATDDERIAECCRGFGADVIMTSASCKNGSERCCEALKKLDKHYDIVVNIQGD
EPLIEPEIIDGVVMSLQRAPDAVFSTAVTSLKPEDAFDTNRVKCVVDNLGYAIYFSRGLIPFNKSGNANPKYPYL
LHLGIAGFDSKFLKIYPELPPTPLQMEEDLEQLKVLENGYRMKVIKVDHDAHGVDAPEDVEKIEALMRTRNIQ

>Emiliania huxleyi AHAL01007974.1 + AHAL01005401.1

MAAPASPVFDGDVVPRIVAVIPARGGSVSVPRKNIKILQGRPLIDWVIQAAEYSGIFDEARRAAGTDIITAAATA
AAAVSATPVWVSTDDDEIHMIAKKCGAKVHRRSPHTATNTASTESALVDFVKARRLAWPAPPIAPLRSRRRATSP
LITPLDFQKGWDAMRRAEISPRSRRDIVGRCRAPRDEISAEIELTSLRDTAEIQPRYSRDTADRGRRAKADSLVT
CVRAHRFLWSVDKRSGVARAVNYDPAKRPRRQDWDGELIENGAFYFTTKEVMEREECRLGGRVALHEMEEHTFAE
LDSPTDWQSRGNKNTNTVWQIVANMAMIHGFWPPGSVKPEGTDESPGSAEVTILGDSKLGLAAAAGLGMGVGLGV
AGVAALLAAAAAGLLRSKAAA

>Hydrocarboniphaga effusa WP_083844567.1 acylneuraminate
cytidylyltransferase

MTPSPSWPSSKRGSSRLRQEPEMRWLAYMPLRGGSKSIPGKNIRPLAGRPLFAWSLGAAIDSGCFDEIWVGTDSD
DIRRSVEQEFGPKVKLFRRGAQTCTDAASTESALLEFATAVDFDVLCTIQATSPLTRVEDFRRAQQRFDEQRADS
LVTAVRTHRFFWTDDGRALNYDPLKRPRRQDWAGTLMENGAFYFSSRAVLESGRCRLGGKIVVHEMAADSAAEID
EPEDWTTIERLIEKSRPA

>Bathycoccus_KDO1 prasinos XP_007511142.1 3-deoxy-manno-octulosonate
cytidylyltransferase

MGCGQSKNHPEFTPGKQDGKVDYRAKKAALARSHKHDGPSGRLPASIDEQNKKKQPRVLGIIPARYGSTRFPGKP
LADIAGKPMIWHTYTNACKSRALDLCIVATDDARIKKVVESFGGICVLTNPECVNGTERCLEVYKKCIKKGEEYD
VIVNIQGDEPFIEPEHIDLVAQIVCEAEEDDVCMGTLCRPAIDRKDVEGVNNVKVVVDRNMNALYFSRAIIPHNK
SGTYDPDTKYLRKLGIYSYRADFLPEYVRMPESMLQISEDLEQNKVIEAGYKIKLGWVEDAVHGVDTVGQLEALN
LAIERGELMHRGKRIKKGGKSFKGQF

>Bathycoccus_KDO2 XP_007508518.1 3-deoxy-manno-octulosonate
cytidylyltransferase [prasinos]

MKSVFAFALGALAGIFGFQKHQEGVFGKKFDSKSTRNAAIEITKEDVNDAETNAAEAEKAPKEEEKEEKKRTKVL
GVIPARYASTRFPGKPLVMIAGKPMIVRTYLQAKKASQLDALVVATDDERIRDAIVKAGGDVVMTDEAIPNGTER
CAQAADRTAGEYDIVVNIQGDEPLIEPEIIDEVVLALKNAPRECVYSTPVTPLKHEEVKMQGRVKCITDVNGYAI
YFSRGMLPNNKKGEVDTSFDYWLHLGLQCYDREFLKLFPIMPATPMQMQEDLEQLKVIENGYKIKVIRVNHCAHG
VDEPEDVASIEKIIAEDPSLD

>Dictyostelium polycephalum EST FLTC01000833.1

MKILGVIPARLAATRFPNKPMAPIGGIPMIGHCYLRSKLCSLLDEVYVATCDQEIKDYIESIGGKAVMTSDIHER
ATERSAEALLNIEKELNGEVFDIVVMIQGDEPLIYPEMIEEVLEPMLKKNVPVSNLMAALPTQEERDNPNNVKVA
KDFNEKALY

>Acytostelium ellipticum FLTD01002867.1 CMAS + HAD

MWTMDERKPLTVAIIPARGGSKQVPRKNLQRVGGVPLVERAVRAAAAAPGIDLVVVSTDDDEIAAVATSAGARVV
RRPAALSGDTATSESAILHALDDLEANGDAVGIVAFLQATSPFIPSDALADAVEEIRSDRADSVFSAHETYAFLW
REGEGSEAVAINHQAAHRPRRQDREPHHLETGAFYVFRAAGFRENRHRFFGRTRIAAVPEWTAIEIDDADQLRVA
RALARLHEAPTPIPVRAVVTDFDGVHTDDTAIIDADGDERVRVSREDGMGVALLRRAGVPLLILSTEVNPVVRAR
ADKLRVPVLHGIDDKETALRNWARDNEVPLGDIAYLGNDVNDLPAMRIVGWPVAVANAHPLVIEEARVVLRRRGG
EGAVRELIERVLSS

>Galaxea1 fascicularis GFAZ01021753.1

NFPTKLAGSLGRMRFVALVPARGGSVSIPLKNIKDLAGRPLLAWCVKAALDSGCFDEVYVSTDHDGIAEVAGKAG
ARVHRRAPETATSSASTELAMADFARAHPDYDVLCLIQATSPLTTPYHFREAIALFKRHQADSLVTAVRSNRFRW
RIDPDGAAHAENYLPAKRPRRQDWDGELIENGAFYLTKKDVLEKDNCRLGGKIALYEMPEHTFVELDSPVDWSIM
ECLCKEHGYTPAANSEDR

>Amphimedon queenslandica ACUQ01025494.1

MRRLRRCQALVPSGRCPIAESLRGGLGAFVGRIVGSLGIVQDGIVQEDPRCPQRRGSPAAGCCERYPERYPERYP
RKDSSREKRMSESLADPPARVVVIPARYASSRLPGKVLADIGGRPMIALVCELALAGRVDAVIVAGDDERILEAA
RAEGAQAILTSSEHRSGSERIAEAISLLGLDPDVVVVNIQADEPLLPPTLIDQVAGLLVDRPEFAMATLCEPIEK
ARDIFDPAVVKVVFDAQGRALYFSRAPIPWQREAWSSTASLRPAADEDEDALDRLPATAGVRHYRHIGLYAARAG

>Physcomitrella_KDO XM_001784898.1 patens mRNA, complete cds

MGEHGSSSSLAPLLVHAAVAAGAITIAAALNHYWFRARLKARPRVVGVIPARFKSSRFEGKPLVHIMGKPMIQRT
WEQAMKCTSLQAIVVATDDDRIADCCRGFGADVVMTSENCQNGTERCNEALEKLAAKYDIVVNIQGDEPLIEPAI
IDGIVLALQMCPDAVYSTAVTGLKPEDASDPNRVKCIVDKNGYALYFSRGLLPSNKKGIPNPDFPYMLHLGVQCY
DAKFLKVYASMPPSPLQLEEDLEQLKVLENGFKLKVIKVDHEAHGVDTPGDIAKVEAVMRAHHMD

>Caenorh_KDO XM_003087186.1 Caenorhabditis remanei CRE_22763) mRNA, complete cds

MILRVVDQAKKVQGFDDLCVATDDPRIAALCRAEGVDVVITDPNHPSGTDRLSEVARIKGWDAEDIIVNVQGDEP
LLPAQLVIQVAELLAQKADCSMSTLCEPIHQFDEFQRDSIVKVVMSKFNEALYFSRAQIPYDREGVKQVEQKLHQ
HAYRHLGLYAYRVKLLQEYVTWDMGVLEQLESLEQLRVLENGHRIAIDIAQVNLPPGVDTQADLDRLNALDVAVF
E

>Drosophila_KDOl XM_017212856.1 Drosophila eugracilis KDOlike(LOC108106007), mRNA

MISPLVIIPARLASTRLPGKPLADIAGRAMILRVLDAAIAADVGPVAVAAADQEICDVVTQTGGRAVLTDPDLPS
GSDRVWQAAQTLDPDGVHDVIINMQGDLPTFAPDDLRAVMRIMPDPTYDIGTLVAPVLSDEEKDASSVVKVACHF
ETDTVCAPALYFSRSAIPWGEGALWHHVGVYGWRRDALKQFVSLPESGLERREKLEQLRALEAGMRIGCARIKTA
PFGVDTPADLDRARQYFKA

>Hyalella_KDOl XM_018150667.1 H. azteca KDO-like (LOC108664086), mRNA

MKNRDNPNLFKIGALLVALVRSLNYAKTHFVTSAPDITHLPIDSGVEIAFAGRSNAGKSSALNTLTQQKNLARTS
KTPGRTQLINLFEVEPDCRLVDLPGYGFAQVPEAIKRKWQKSLGEYLQKRESLKGLVVLMDIRHPLKDLDQQMID
WAVSVDIPVLLLLTKADKLASGAQKRQLDMHYSQSRCQRFNLSGSPIDKHFFYHLPSLCVVLSGHLSIVSDRYCS
SRFPGKPLVKIAGIEMIKRVADIARYVCDSNHDCRYLVATDDQRIVDFCQQHQIPVKTTSTSCQSGTERCWDIVA
NLEQKPKFIINLQGDNPLCPPHVIQALIDTWRHEQADIFTPYIQLSWDEFNQFIENKQMTPFSGTSVLVNKMGFA
LAFSKNVLPAIRDLAQAKKQMAKSPVKRHIGLYGYTFDALQDYFILPESLGEKSYIEGLEQMRFLENGLKVKMVE
VDYRGRKTTSGVDSPEDISRVEQIIAEFGELILNQ

>Bombus_KDOl XM_003492952.2 B. impatiens LOC100745105 mRNA

MKNRDNPNLFKIGALLVALVRSLNYAKTHFVTSAPDITHLPIDSGVEIAFAGRSNAGKSSALNTLTQQKNLARTS
KTPGRTQLINLFEVEPDCRLVDLPGYGFAQVPEAIKRKWQKSLGEYLQKRESLKGLVVLMDIRHPLKDLDQQMID
WAVSVDIPVLLLLTKADKLASGAQKRQLDMHYSQSRCQRFNLSGSPIDKHFFYHLPSLCVVLSGHLSIVSDRYCS
SRFPGKPLVKIAGIEMIKRVADIARYVCDSNHDCRYLVATDDQRIVDFCQQHQIPVKTTSTSCQSGTERCWDIVA
NLEQKPKFIINLQGDNPLCPPHVIQALIDTWRHEQADIFTPYIQLSWDEFNQFIENKQMTPFSGTSVLVNKMGFA
LAFSKNVLPAIRDLAQAKKQMAKSPVKRHIGLYGYTFDALQDYFILPESLGEKSYIEGLEQMRFLENGLKVKMVE
VDYRGRKTTSGVDSPEDISRVEQIIAEFGELILNQ

>Podocoryna carnea GCHV01014581.1

MHKTTVSESGDHSGKGKMNTTASGSSENASASERKRKMTGSDKEPGKKPHLSALILARGGSKGIPLKNIKVLAGV
PLIGWVIRAAFDSGVFDSVWVSTDHAKIAEVAEEFGAQVHRRSPKVSTDSSSSLDAIQEFLRLHNEVDIIGNIQA
TSPCLHPNHLQDATRKIWEDGCDSVFSVVRRHHFRWQEVKTGGVDTKPLNLDPANRPRRQDWDGELCENGSFYFA
TRELIEKGYLQGGKMAYYEMLPEFSVDIDIDIDWPVAEQRVLRFGYFGKDQLEQVKLLVCNIDGCLTDGQMYLSA
GGEELVSWNIRDTAGISMVQEQGVEVCLISSRDCHIK
LLAKKTDCQLEQNAENKVEVLELWKNKRKLSWEQVAYMGNDESDVECLKKAGVSGVPHDASSDAVKAAGYTCRNS
AGRGAVREFTDHILLLSEQARAKSQQKKNTE

>Acyrthosiphon pisum NP_001156112.1

MTVRNSHWVALFSQLFVLSASVVLFMIPIYLLWSHITYEQNHLSNYKPHVVALILARGGSKGIPRKNLAVIQNTT
LLRRSLDTINDCGLFHDVWVSTDDKEIAAEAELAGAKVFHRSAETASDEATSLSAVKEFSMYHQKVDIFAIIQCT
SPFMTAEYLTRAYKMAVNQKFESVFSVTRTHKLRWKLDSDGQLQPSNFDVTKRPRRQDWDGEYVENGMFYFTYKN
LIVNNKLQGGKLGVVVIPLNRSLEIDTQFDLQAARLLAPLLDKPKNVTNRTNEIIKKS

>Drosophila bipectinata XP_017089480.1

MFLLKSAPALILLMFIIGISCRELCSKNNIHALILARGGSKGIKLKNLAEVGGISLLARTIKTIQNSACFEHIWV
STDDKRIALEAQKYGAIIHDRPEKFAKDNTPSIDAIKEFLEVHATVKDFALFQCTSVFLKPTYIQEAVQKFKTHD
CVFAAKRSHYLRWKEVNDSLVPVDFNLESRPRRQDWKGDIVETGMFYFSKRKLVDKGLLQNNRCSIVEINVEDGL
EIDSSHDLSLAKYILSSRTKTDL

>Bemisia tabaci XP_018904120.1

MSPISKRFGWYDWWPLDGWNDRWYLLRCRQLMKISLVLTCTLLPQAGTIAHDTFAPSSDRETFRSAALILARGGS
KGIKKKNLSVVNGKTLIKRAVENILESDVFDSVWVSTDDMEIAMEAQKATALLHWRHPSTATDSAASILGVQDFF
AKHPDLEVIGLIQCTSPFLTSQFLIKALRLIEEAHCDSVFSVTRSHQLRWNLDQRGDLHAANFDATKRPRRQDWN
GEFVENGMFYVFKTSLLAENVLQGGRARVVEIPKNRSLEIDTEFDLVVAKVVAQILDSESQQMIP

>Daphnia magna JAN83715.1

MHVAGLILARGGSKGIRLKNLALLKGTPLLLWSLKTMSQSRGLTSIWVSSDHDDILELAHQNGAQIHRRSAESSS
DGASSLDAINEFLDSHSEVDVVALIQCTSPFVRVAHLNEALAKMTSGCYDSVFSVTRSHSLRWTQLEPNETEVDV
ANTEEIRAVNFDPHCRPKRQDWNGDLVENGAFYLSSVQLLRRGLIQGGKISFVEMSAAHSIDIDTAYDLWLAGQQ
ASYFGFEPDGDSGDVNHLQRC

>Thelohanellus kitauei JWZT01001893.1 CMAS KII70985.1

AIIPARGGSKRLPNKNIMLLADKPLIVWTIEAALDSHCFDHVYVSTDSPDIGIIAKIAGAEVPYLRPDNLATDEA
ATNDVVDHLVGWVEKNVAPIAKVAILQPTSPLRTAYHIKEAFSLYKKKKANAIVSVCELEHPYQFCNKLDPSLSL
DNFLNQKNNKRTQELERYYRLNGAIYLFDREFVGKQSELYGPNSFAYIMNSRDSVDIDNEFDFEFASFLMSLK

>Hydra1 vulgaris ABRM01020176.1 CMAS

ARLCERLLPQRGAVLRNGHQPAAVPRTHGVHAGPCGRRAGQGIAPMRLAVIPARGGSKRIPRKNIKSFCGKPMIA
WSIQAARKSGLFDRVVVSTDDLEIAEVARQWGAEVPFMRPPEISDDHAGTTPVIAHAVRWFIDQGISPELVCCLY
ATAPFVHISDLRAGLA

>Hydra2 vulgaris ABRM01004088.1

MTKIYGIVPARMAASRFPGKPMYPILGRPMVEHVFLRAAMYQGWSKLTLATCDDEIANFAASKGISCTMTGSHHT
RALDRVAEAVQRLGEPVAEDDIVVCVQGDEPMMRPDMINVVIEPLLHDASKAGTILAMHIVDKDIWTNPDTVKLV
HNATGEVLYTSRAPLPYCKGEFSADLMARRIYGIFAFRWKYLKMFTEHAETRLEQLEACDSNRILDMSFRQHIAP
YPNIQSYSVDSPGDIALVEKYMQGDALWESYK

>Hydra3 vulgaris ABRM01023149.1

MTYTVLIPARLASTRLPNKPLVDIAGLPMVVRVAQRVKAGLDASIRVVVAADDASIVAACESHQIECLLTRVDHP
SGSDRLAEACALLGLSDDSVVVNVQGDEPLIDTGLVSAVADLLQAHPHATMGTAAHAIHDVADFKNPNVVKVVTD
ASGLALYFSRAPIAWWRDGFANGIDTLPDPAPLRHVGIYSYRVGFLKTFPTLPQAPIEVVEALEQLX

>Clavaria fumosa CVRD01107425.1

MGFTVIIPARLASTRLPNKPLADIAGLPMIVRVARRAAESGAGRIVVATDAPEVAAACAAHGVQALMTRADHPSG
SDRLAEAVEQLGLADDAVVVNVQGDEPLIAPAMIDACAATLTAQTDCVMATVAHALTDASEFTNPNVVKLVTDAA
GRALYFSRAPIAWWRDGGGQPNQALRHVGLYAYRAGFLRRFPQLAVSPLEQIESLEQLRVLWHGERIAVHVSPER
PGPGVDTPEDLERVRRLIQV

>Puccinia arachidis LBLN01031890.1

MSFIAIIPARYASTRLPGKPLVDIHGKPMVVHVMERARESGASRVIVATDHPEVARAVEAAGGEVCMTRADHHSG
TERLAEVIEKYQFADDQIIVNVQGDEPMIPADIVHQVATNLAQADAGMATLAVPITDAEEAFNPNAVKVVMDARG
YALYFSRATIPWDRERYAQSREQIGDTLLRHIGIYAYRAGFIRRYISWEPCPLEQIELLEQLRVLWYGEKIHVAV
AKTIPGVGVDTPEDLTRVRAAMLR

>Beauveria bassiana ANFO01000218.1

MSFVAIIPARYASTRLPGKPLKEINGKAMVLHVLDRARESGAERIIVATDHPDVARIVEAAGGEVCLTSPDHQSG
TERLAEVIEKCGFSDDTVIVNVQGDEPMIPPVIIQQVANNVATSQAGMATLAVPIESAEEAFNPNAVKVVMDAQG
YALYFSRATIPWDRERFAASREEIGDTFLRHIGIYGYRAGFIRRYVSWAPSQLEQIEMLEQLRVLWNGEKIHVAV
AKAIPSIGVDTPEDLERVRLAMR

>Pleurotus salmostramineus BDGN01023126.1

MSFIAIIPARFASSRLPGKPLADIGGKPMVVHVMERAKESGADRVIVATDHPDVFAAVEAAGGEVCMTRADHQSG
TERLAEVIELYGFSDEDIIVNVQGDEPLVPPVIIKQVAENLASCEAGMATLAVPIETAEEAFNPNAVKVVMDAKG
YALYFSRATIPWERERF

>Oscarella1 comp7984_c0_seq1 len=1506 path=[1:0-1121 1123:1122-1505]
Length = 1506

RAIGLILARGGSKGIPLKNIKLLNGRPLLYWTLHAMRDSGVADEIWVSTDHDDIARISEAGGAKVHYRSAEVSKD
TTSSLEAIQEFFHIQKLQCDVVIVAQATSPCITPNHYKKLMDKYIIGGYDSIFTVTRRRDFRWKQVEGNAATEAV
NFDPRHRPRRQDWSGELYEDGAVYATTPEQVQLGILQGGKVGYYEMPPHLSIDIDNPDDWPIAEKKVRQYGYNPE
DVTQKPLVIGLLVCDADGTLTNGQTYVTNDGKEMRSYHCKDASGIRRMKQQGVEVKIICTEKCEAHHHKAKEMDV

PIAAGCMDKLSQVDKWRKELNLEWTQVAYIGDDIFDLECIKTAGVGGAPNDAELDVRLAARFISPLNGGQGAVGN
FCNYLI

>Oscarella2 comp40723_c0_seq33 len=3116 path=[3346:0-1154 4501:1155-1583

KAIGLILARGGSKGIPLKNIKLLNGRPLLNWTLHAMRDSGVTDEIWVSTDHDEIERIAVEGGARVHRRITEVSQD
NTPSIVSVLEFVDVQNPQCDVIVRAQATAPCIRPEHYRELMKKYIGGGFDSMFTVTRRHPFRWKEVENGSSSEAL
NFDPSNRQRRQDAAGELCETGAVYVTSLQALKATRTFQGGDKVGYYEMAPHLCVDIDDPDDWHVAEDKVRRYGYN
PAKTVGRKSIRLLVCDADGTLTDGKIYVGADSGWTCSYHSRDATGIRRIQQRGVDVKIVTTSRFAHRETAKEINA
TIANKCEDKLAQVDEWRQEMRLEWSQVAYIGDDESDLECINMAGVGGAPSDAEEVVKSAAQYIAPVGGGLGAVNN
FCKYLME

>Lethenteron_camtschaticum_APJL01003329.1

GNYLAAAMPSGGHLSALVLARGGSKGIPLKNIKPLAGVPLVGWVLRAAKHSAVFDSIWVSTDNDDIERVAKKFGA
KVHRRSPEVSKDSTSSLETIQEFLKHHELDSVMNIQATTPLLNGLTSDSTSNNTRHKLENAFESNTAQLRKPPQC
LIIEVCAGSAMSTVPLGHNPAKRLRRQDWSGELCENGSIYLATRKIIEDGYLQHGKVGYYEMKAEHSVDIDIDID
WPIAEQRVLWYGYCGEQQAAGIQLVVSAIDGCLTDDHLRVSSSGEEHSTFCASDMAAIRTLQGKGVEVTKFAEKL
GCKLKQGTADKLSQLNSWRVKLGLGWEQVAYFGKESSDAGCLQKAGLSGVPLSAVSDLAKSYARFQSTLSGGQGA
FRQFA

>Petromyzon_marinus_AEFG01022991.1 AEFG01022993.1 (il en faut 2 pour avoir
la seq quasi complète)

RCEVNFRRRSSSHPRGSFKRAAMASGGHLSALVLARGGSKGIPLKNIKPLAGVPLIGWVLRAADIERVAKKFGAK
VHRRSLEVSKDSTSSLETIQEFLKEVDSVMNIQATSPCLHPEHLQEVISMFRSGQYDSIFSVVRRHHFRWQEVNP
AKRLRRQDWSGELCENGSIYLATRKIIEDGYLQVGKVGYYEMKAEHSVDIDIDIDWPIAEQRVLYGYCGEQQAAG
IQLVVSAIDGCLTDDHLRVSSSGEEHSTFCASDMAAIRTLQGKGVEV

>Branch_belch1_XP_019615988.1

MSSSEGPEPKKPRTSSSELQTLGKMNSLSKTDPELRYKRMGFEPTPELRAEWKRRFGKEEPHIATLILARGGSKG
IPMKNNKKLGGLELIGWVIRAALDSDIMDSIWVSTDHDDIAATARRCGAQVHRRSAEVSQDTCNSWVTIDEFVRY
HPEVDIIVDIQATSPCLHPFHLHDGMRLTLKDGYDSVFTVVRRHGFRWTDPKWGIQPFPLNFDPAKRPRRQEWAG
EIVENGAYYMMTRDVEQQGLMQGGKMAYLVMDPEYGVDIDTDLDWEIAEQRLFRFGYHGRTPKTVKLVVVNIDGT
LLDGQVQVSPTGEELRSFKTTDIVGVQQLQENGIEFRVVAEGDNEVQRALADKLSAKLEENCTDKLTVIDAWRQE
LGLEWKDIGYMGYDISDMTCIKKAGMSACPSDAYAAIQKHSHFISTYRGGQGALREFCEQILKDEEKMGTYADVT
ERIFEDFPKLWSASPTRKARTRSLSQSQSTAGSDVPSI

>Branch_belch2_XP_019626693.1

MDGDCDTIADLCMQRFGKKKPHRACLVLARGGSKGIPLKNIKPLAGTPLVGWVLRAAIDSEVFDSVWVSTDHDEI
AAVSREFGAQVHRRSPEVSRDASTSLETIQEFIREHPEVDIFGNVQATAPCVHPFHLRKAMKAMTEDGFDSLFAV
VRRHAFRWKEVKEGEVTEPLNLNPAKRPRRQDWDGELIENGSFYFSTKDLVQKGLLQGGRVGYLEMAAEYSVDID
IDIDWPIAEQRVLRFGYFGKEKRQAPCLMVVSAEGALTEAQVHLSPTGEEFRSFNVHDINGMRQLASRGVEVRII
SEDESEVHRQLAAKIGCKLEEGCMDKLAVVDSWRKDLGLDWLQVAYIGSDASDSECIKKAGVNGCPMDAQHPAKN
YSRFVSKRRGGQGAIREFCQHVEITIEKANNENKPRPE

>Ciona_intest_NP_001093597.1

MEHSNSAINHLAALILARGGSKGIPMKNIVNVGGLPLICWVLRAAVDSNAFDSIWVSTDSDEVAEVASSYEVVKI
HRRSDEVSKDNTSSMESTQEFLNYHPEIDAIGLLQATTPCIQPSQLLSAAEMIKFGGFDSVFSVVRRHFFRWKEV
KQGKDGDVTHPLNFDPSHRPRRQDWAGELCENGGFYFAKTSVVRQGLFQGGRTGYQEMPHEHSVDIDTPFDLVVA
DYVINKYGYKGK

>Neisseria_KDO CBA03570.1 3-deoxy-manno-octulosonate cytidylyltransferase
[N. meningitidis]

MNTENLKTVIVVPARYASTRLPGKPLADICGKPMIQHVYERACKVPYIDDVIVAVDDRRVAEVVESFGGKVIMTS
TQHDSGTDRLVEVMGKYAADIYINIQGDEPLIRSEDIALLAQGMKDEQISVGTLCHALPASEATNPNTVKVVLSA
NGNALYFSRSPIPYPRDKEHAYYFKHIGVYAYRKETLAKYSNLKQPDIELSEKLEQLRLLDAGIDIRVFEVPETG
PGVDTPECLERVRAIMSGQLPIDKHSVNLADIRLVITDVDGVLTDGGIFYNENGECLKRFHVRDGLGIRLLEESG
IKVAVLSGRDSPTLRKRIDDLGISYYQLGIKDKHAACIELMQEANCLK

4 **- Uridine diphospho-N-acetylglucosamine (UDP-GlcNAc) 2-epimerase**

>HSA_GNE_AKI72325.1

MEKNGNNRKLRVCVATCNRADYSKLAPIMFGIKTEPEFFELDVVVLGSHLIDDYGNTYRMIEQDDFDINTRLHTI
VRGEDEAAMVESVGLALVKLPDVLNRLKPDIMIVHGDRFDALALATSAALMNIRILHIEGGEVSGTIDDSIRHAI
TKLAHYHVCCTRSAEQHLISMCEDHDRILLAGCPSYDKLLSAKNKDYMSIIRMWLGDDVKSKDYIVALQHPVTTD
IKHSIKMFELTLDALISFNKRTLVLFPNIDAGSKEMVRVMRKKGIEHHPNFRAVKHVPFDQFIQLVAHAGCMIGN
SSCGVREVGAFGTPVINLGTRQIGRETGENVLHVRDADTQDKILQALHLQFGKQYPCSKIYGDGNAVPRILKFLK
SIDLQEPLQKKFCFPPVKENISQDIDHILETLSALAVDLGGTNLRVAIVSMKGEIVKKYTQFNPKTYEERINLIL
QMCVEAAAEAVKLNCRILGVGISTGGRVNPREGIVLHSTKLIQEWNSVDLRTPLSDTLHLPVWVDNDGNCAALAE
RKFGQGKGLENFVTLITGTGIGGGIIHQHELIHGSSFCAAELGHLVVSLDGPDCSCGSHGCIEAYTSGMALQREA
KKLHDEDLLLVEGMSVPKDEAVGALHLIQAAKLGNAKAQSILRTAGTALGLGVVNILHTMNPSLVILSGVLASHY
IHIVKDVIRQQALSSVQDVDVVVSDLVDPALLGAASMVLDYTTRRIY

>Drer_NP_957177.1 [Danio rerio]

MERLEKPKKERLRVCVATCNRADYSKLAPIMFGIKSHPDIFDLEVVVLGSHLIDDYGNTFRMIEQDDFDIGSKLH
TIVRGEDEAAMVESVGLALVKLPDVLQRLAPDILLVHGDRFDALALATAAALMNIRILHLEGGEVSGTIDDSIRH
AISKLAHYHAVCTLSAERHLISMCEDHSRILLAGCPSYDKLLSAYKRDDYADIIKSWIGDDVKEQDYIVALQHPV
TTDIKNSIKIYELMLDALISFNKKTLILFPNIDAGSKEMVRVMRRKGIEQHQNFRAVKHVPFDQFIQLVAHAVCM
IGNSSCGVREAGAFGTPVINLGTRQTGRETGENVLHVRDADTHNKIYHALELQFGKRYPCSKIYGDGNAVQRILK
FLQTIDLSEPLQKKFCFPPVKECISQDIDHILETQSALSVDLGGTNLRVGIVSMKGKVVKKYVQLNPKTFEERIE
LILTMCKQAMADAVHLNCRILGVGVSTGGRVNPQDGVVLHSTKLIKEWSSVDIRTPLSSALHLPVWVDNDGNCAA
LAERKFGHGKGVENFVTIITGTGIGGGIIQHNELIHGNTFCAAELGHIVVSLEGPECMCGGHGCIEAYSSGLALQ
REAKRLHDEDLLLVEGMTLNNKEQVNAIHLINAARLGNSKAETVLHTAGTALGLGIVNILHMINPSLVILSGVLA
VHYETPVRQVIGQRALLTAQGTKVMVSDLEDPALLGAASMVLDYTTRRTY

>Bflo_XP_002588099.1

MDTEGLNVKNRKMRVCVATCNRADYSKLAPVMFALRDDPDMELQVVVMGSHLIDDYGSTYRMIQQDEFEVDGYLH
TIVRGEDEASMAESVGLALVKLPDVLVRLKPDLLIVHGDRFDALSLATCAALMNVRILHIEGGEVSGTIDDSIRH
SITKLSHYHACCTDRARRRLLSMCEDNDHILLAGCPTYDKLLSCNAKNYDNVLSKWVGEGVKPKDFIVALQHPVT
TDIKGSLKMFDLMLDALLEFNVKTLVLFPNIDAGSKEMTRMIRLRGLEHHPNFSLAKHIPYDQFIILLANTGCLI
GNSSAGIREAGAFGTPVINLGNRQIGREAGENVLHVRDADTKGKILHAVNLQYQKQFPCSYIYGDGHAVQRIMKF
IHKIRNDLEKPIQKSFIFPPMKDTVSQDIDHILETQSALAVDLGGTNLRVAIISQQGEVLHKMSELTPPTYEQRM
DLLVRMLVEATSKAVELSCRILGIGISTGGRVNPHEGMVLHSTKILEEWSSIDLRTPISSKLHLPVWVDNDGNCA
ALGEKKFGKGKGSEDFITLIVGTGIGGGIVLNNELVHGANFCAAELGHISVSMDGPDCMCGSSGCVESYASGIAL
QREAKKLHDADELLVPGVHLREGEEVTGKHVIQAAQLGNKKAEKVVETACEALGLAVCTLLHTVNPSHIILCGHL
APHYVDGVREVIQRRALPSAANSIQIMVSDLEEPALLGAASIVLEYATRRIY

>Polaromonas_sp WP_007873900.1

MKRKICVVVGSRANYSSIKAVMRAVAAHPALELQVVAGASALLDRYGAVVDIMEKDGFTIDARLFMLVEGETPAT
MAKSTGLGLIELPTIFERLQPDVVLTVGDRFETMATTLAAAYMNIPLAHTMGGEVSGNIDENIRHAITKFATIHF
PASEDARNRIIKLGEQPDTVHNVGCPRMDLVAGFLRNETEVSNQLLSEGVGSSFDVKGDFLLVSQHPVTGEYGSG
ERQVAATLGACLELGMQTLVLWPNADAGSEDVSRGIRKFREQHPDAPFRYVKNLPPEIYMPLMARTRCLIGNSSS
AIREGAFIGTPAVNIGSRQTARERGQNVVDSGYTQAEILAAIRDRLKTGRLPSNPIYGDGHAGPRIAEILATATF
NINKVITY

>Campylobacter_concisus WP_085657992.1 [Campylobacter concisus]

MRKICVVTSTRAEYGLLYWLLKEISADSELELQIIATGMHLSPEFGLTYKEIEKEFNIDKKVEILLSSDTSIGIS
KSMGLAQISFSEAYEELNPDIVVVLGDRYEIFSATSAAMIARLPIAHIHGGEATEGLIDEAIRHSITKMSHLHFT
ATNEYKNRVIQLGEDPDRVYNVGGMGIENIKRLKLLNKKEFEESINFKLNKKNLLVTFHPVTLENDTAEEQFQAL
LNAIDELENTNIIFTKANSDTNGRIINLMIDRYVSNNQDKSVCFASLGQLRYLSALQYVDAVVGNSSSGLAEAPS

FKIATINIGDRQRGRIKASSVIDCQADKNSILKAFDKVYSYDFQQVLKNVVNPYGDGCASKKIIEVIKRVDLTNI
LKKSFYDLKVDL

>Nonlabens_marinus WP_041497253.1 [Nonlabens marinus]

MNKKICIVTGTRAEYGIMYWLIKFLQEDLDIDLQLIVTGMHLSPEFGSTYKEIEGVGFKIDKKIEMLLSSDSEVG
ISKSMGLAQISFSEAFQDLAPDVILVLGDRFEIFSAVAAAMIARIPVAHLHGGEATQGLIDEPIRHSITKMSQLH
FTATEVYRKRVIQMGENPSRVFNVGSPGLDNIHRLTLLDKRQFEKSIDFKLNQKNILITFHPVTLEKNTSKKQFK
ALLHSISNLEDTSFIFTKPNSDTDGRIIIQLIDEYVSKNPKTSCAFKSLGQLRYLSALKYVDVVLGNSSSGLAEA
PSFKVATIDIGDRQKGRIKADSVISCDPTEESISQALQLAFSKHFQESLKNVKNPYGEGGASEKIVKILKDFDYS
DILKKKFYDINYSI

>Bacteroides_fragilis WP_032530837.1 [Bacteroides fragilis]

MRKICVITGSRAEYGLLSGLMAKIQFDRDLQLQIIATNMHLSPEFGLTYKEIETDGFRIDKKVEMLLSSDTANGT
AKSVGLATIGFADAFEDLSPDLILVLGDRFEILAAVSAALFYKIPVAHIHGGEITEGAYDDNIRHAITKMSHLHF
TSAEPYRRRVIQLGESPDRVFNVGALGVENIKHVPLLSKEELEKSLNFQLGDKSLLVTFHPVTLENSTAEQQCMN
LLESLESFPDYRIIFTFPNSDTDGRIIIDLIKRFVERHRSESVAFVSLGKVRYLSTLQYVSAVVGNSSSGIIEVP
SFGIPTLNIGDRQKGRISSRSVVHCGTSKEDICLGIQTVLSDPVVRIAKQKRNPYDKKGTADAICTILKTVSLDG
LTSKSFYDL

>Sinorhizobium_meliloti WP_017271146.1 [Sinorhizobium meliloti]

MSKKLLFLTGTRADFGKIEPLAVAASKAGYAINFFVTGMHMLEKYGATKHEVHRLEGVEVHEFINQRLGDPQDLI
LSKTILGVSDWVYEHKPDLVIVHGDRIEALAVSLVCATNYIRCAHIEGGEVSGTIDEIYRHCNSKLCTYHFVSSE
IARARVLALGEDPQFVYNIGSPELDLHRGDSGVTLSKVRDRYAIPFSEYGIVTFHPVTSETDTIGAQARSLFTTL
AESGKNFVIISPNNDPGSDDIFAEISKLDPHRFRHLPSMRFSYFSELMKNAKAIIGNSSAGVREAPFLGVPSLNV
GTRQNNRSSAGTITDASAFDTDTIREFLNTTWGRKFESDQSFGNGNAASRFVQVLNDDTFWSAPLQKQFND

>Gymnopus_Luxurians KIK55994.1 luxurians

MPQSKQHLLFITGTRADWGKLEPLVKKAVAGGSPTDIFVTGMHTLKDYGRTETEIRKLPGIRICTYVNHSAGDDP
TTILSNTLLSLRNWVQQFRPDMLVVHGDRVEAFAACIVAAQMPLPCAHIEGGELSGSIDEIYRHCNSKLSTYHFV
SSETAKRRVTSLGEDPSTIFNIGSPELDSHFGLSGVTIDEVKTSYGISFTEYAMVTFHFVTSETETIGDQAASLF
DCLTNSGKNFVVISPNNDPGTEKIGDVISRLDPSRFHRVSSLSFNDFSILMKDCKAVIGNSSAGVRETPFLGVPS
LNIGTRQQNRAEADSITHSSAFDTATIQKFLETKWGKRFDPDYAFGRGNAAQQFVEVLGREGFWEKDMQKIFYEE
R

>Termitomyces_sp KNZ76402.1 Termitomyces sp.

MTGTRADWSKMQPIARGIVKSGAFACLDILSTGMHEDGQHGNTGNDIEGSVQGVRQYHRRPNHLQGSDPVNAGIE
TMKIIADLTREHGYTDIFIHGDRIEANYSSLVALSLNMRIHHIEGGEITGSVDNALRYSVSAHAHFHYVCNEEAK
NRLIRCGNNPATIFVIGSPEMDRHAQTGERSLEEIKRHHKISYDKYGIASLHPVTTVDPTVTLEEALYFFFALRA
SGKSWIVIKPNNDPNSEQILLALEKVLEEDQKSAQPQFFVTDNIPFDDFSTLQANAAFAAGNSSAMNMDAVINIK
AFIPDLIVHTIKVKWDKKFERDTTFGTGKAEETFTQILAAPGYLDRDVQKRFFFSI

>Photobacterium_sp WP_064605358.1 [Photobacterium sp. J15]

MSKKILFLTGTRADFGKLKSLINKVEQSDELEAHLFVTGMHMLSQYGMTSIEVEKSGFKNIYKYINQNSHDSMDS
ILAKTIHGLSDYVKELKPDLIIVHGDRVEAMAGAIVGSLNNVLVGHIEGGEVSGTIDELIRHSVSKMSHIHFVCN
DKAKQRLIQLGELESSIFVIGSPDLDIMTSTSLPNIQKVKLRYNIPYDDYALFMYHPVTTDVKNLKEKIRKVIDG
MISTDDNYVIIYPNNDHGSEIIIQELERLRNNPRFRIFPSIRFEYFLTLLKNADYMLGNSSAGVREAPFYGLPSI
NLGSRQDNRSKAPSIINVLENQEQIYNAVVDAKKSKFEPTKEFGQGDSDLRFIEIINNKDCWRTNVQKVFVDLVI
N

>Ensifer_sp SDO01308.1 [E. sp. YR511]

MKVPARAFLNALPHLPPGTFLDIFITGMHMLPEYGLTKNEVLGLEGVREFHEYDNHAQGPDQVDVAEETIKHLKK
VIKREKITDMFIHGDRVEALACATAARLLNVRVHHIEGGEITGSVDNDIRYAVTALSRFHYVCTEDAQQRLIATG
QPAETVFNVGSPEMDAHAVVGSLTLDQVKELHGIPFTGQFGIVSFHSVTTEQQFMANDAANLFATLEEFAMGGGK
SIKPFLVIMPNNDPGTDDIRRIIEIIFDDRDSELFFRAANIPFEHFSVLQRNAALFIGNSSAGVREMPFHGVASI
NVGSRQDGRAPDIQSIVTVAPNDNATMLKALEEGWGKKFPRDTTFGDGECEERLTQIFKTAGHFNLPVQKTLNFG
Y

>Micromonas_commoda XP002506929.1 Micromonas commoda +MEP cytidyl transf

MPACPNTEADKASLYAPDKCTDMKTTLRRKICVVTSNRSDWSKLKLVAINLRKLCTSQDNQEASDIQVDIICLGS
HLLHELGATKNIVKEDFPNAYELHTLVAGDSVESMTDSVGFGIVKLTSLLCALKPNIVLVHGDRFDAFCAAIAAN
MLNLTIAHVEGGELSGTVDGTLRHAITKLSHLHFTCTPEAARRIRGMGENPASIFVTGCPSYESLFAVSATCWED
EKMDQFFNGTPFKLKPNKFILVIMHPVTNDLEESNTLYGSLLSCLFSRKTPTVMFYPNVDPGNKSMIQTLHKHQK
ADPASTSWLRLVTHMPHAKFTALMRHASAMVGNSSAGIRESCVFGIPTLNLGSRQEGRRVPANVTTLVKPSIRSI
DCWFDNELGKRYAQSTMYGFPDSAKRIAHHLSRIDTSAGQLKQFWEPRYALLPPLQPRQYVSRTQAILADSTSSP
TSSPIGRCKILGLITARGGSKGIPGKNIIDLNGKPLIQYTIEAALSSKQLDRVILSTDSDEIAEVAQNCGCEVPF
RRPSELAADDSSHLACIVHALNILRETECFVPDFVVILQPTSPFRKSIDIDSCINIMLTSSCDMVLSVCESSLNL
SKNFYFAADGTLSPFAESTAEIDYTPRQKLLKTYAENGAVYVLRTQSLLYPPDNAPNVGSFRSADTKGYEMPVER
SLDIDNPFDLHVARLLMAKPF

>Chromera3 velia ARZB01056196.1

MEQPVKVCVVTSTRADYSKLRGLMLALEKSPLFELVPLVIGSHLLIESGYSFRLVEKDFAKSATKVSTVAAGNDT
NNMADSVALTILKVSQFLALAKPKIVIVHGDRFDAFGVASASTLLQIFTVHLEGGELSGTIDGKLRHSISKLSDL
HFTCTEAARMRVIEMGEENSAVFCTGCPSYDVFLEMEHIGSGHSTEDLLVTHRVVPHEYFIIMYHPDTCSIQDTV
KQYRSILEAVDRTGERAILFYPNIDPGSKDMIQTLHEIQKTSPTFSKNVNCMTSLPHEEFIILLKNCKAIVGNSS
AIIREAAFFGTPAVNVGTRQQGRTCPPNVFSVKGENSDEVLQALLEHPKERFDRDTTYGDGHAVSRMMKHLEGVL
GQL

>Chromera1 velia ARZB01071396.1

MTSAKRRISIVVASRANYGRIKSVLRAIEDHPDLELSIIVAASALLYRYGEVVERMREDGFEIAAQVYSIVEGEN
PTTMAKSTGLGVIELSTLFENLKPDIVLTVADRFETMATAIAASYMNIVLAHTQGGEVTGSIDESVRHAITKLSH
LHFVTTDLSRQRVLQMGEDPDAVFITGCPSIDPIASIDLSLPDDLFEMYGGSGPTLDPRKPYVVVLQHPVTTEFG
QGFEQINETLAAVHALGLQTVWLWPNIDAGSDDVSKGLRMFRTRHHKAPIHFFRNFGVEDYARLINNAVCLIGNS
SSGIREGAFMGVPVVNIGTRQTGRERGPNVIDVGYDRGEIQAAAQRQIDHGRYPENPLYGDGQAGRIADLLAQAN
ISIQKSFHRC

>Chromera2 velia ARZB01004620.1

RICVVIGSRANYSSIKSALRAIDQHPELELQLVIGASALLDRYGSVVDLIEADGFRADARVHMLIEGETPVTMAM
STGVGMIELPQIFSRLDPHVVLTIGDRFETMATTLAAAYINIPVAHTMGGEVTGTIDESIRHAVTKFAHIHFPAS
RDAAERIEKLGERPEMIFQVGCPRIDLVAEVLERDEADVNSHLFDTGVGDRLDLSGPFVVMSQHPVTTEYGEGER
QITETLHAVRQVGPVIALWPNPDAGSDDIARGLRKWREQGKAENMHFFKNLPIDVYIRLMRRAVCLVGNSSSGIR
EAGFIGTPVVNIGSRQAGREHGENVLHVDYDRGAIADAMMQQQRNGRWESQSTYGDGRAGMRIADTLASLGDVEV
QKRITY

>Sterkiella_histriomuscorum LAST02025428.1

KILFLTGTRADFGKIKSLISILEKQKEFEVFVFVTGMHLQEEYGYTLIEIERCNFKNIHTFVNHTHETTMDLTLA
KTIEGFSAYCNTVKPDMIVVHGDRVETLAGAIVGSLNNILVTHIEGGEVSGTVDELIRHSVSKLSHIHFVSNQEA
AKRLIQMGEVKESVFTIGSPDIDIMFSDKLPDLNTVKEYYKIPFENFSIVMFHPVTTEIENMKQYAENFVHALLK
DNHNYIVIYPNNDLGSRFVLDSYDKLKSNERFRVFPSLRFEYFLTLLKNSQFIIGNSSAGIREAPYYGIPIINIG
TRQQNRAIHADIINVDYSEKQIAEALSIIDSHKVQKSDDDFGQGNSAELFLSCLQKSDIWQLNHQKQFDS

>Arcobacter_marinus WP_079580078.1

MNKKRIVFLTGTRADFGKIKSLIKITQKSKLFDVHIFATGMHMIAKYGKTIIEIEKSGFKNIYPFINHDDIDHMD
RNLAKTIDGFSHYILEIKPDLIVVHGDRIEAMAGAIVGSLNNILVTHIEGGEISGTIDELIRHSISKLSHIHLTS
NEQAKKRLIQMGEDRNCIFNIGSPDLDVMNSKNLPTLKVVKKYYEIDFKEYAIVMFHPVTTEMVKLKKQIKTFLN
SLIKSDLNYIVIYPNNDMGSNIILEEYKKITNNNKFKIYPSLRFEYFLVLLKNAQFIIGNSSAGIREAPYYNIPT
INIGNRQNNRVKSKTIKSIDFIEKDITKAIKDALLTKKVTKDVDFGNGNSDKKFYNLLQKNTFWNIQNQKQFKDL
N

>Prorocentrum1 minimum JXLM01000037.1 PIGL NEUB

MGGRVFGSGGDLLDSRACRCVMFVTGSRAEYDILYPVIEAVSRSESLRPEIVVTGSHLASVYGLTVKDVEADGYP
VVAKIDNLLASDSDAARAKSAAIQLQGLVDVVAYRRPDFLVVVGDREEAITVALTGGYLDIPVVHIAGGDTADDF
NIDNSVRHAVTKLAHLHMTASAESAERVLRLGEEPWRVHNVGAPGLDRLVGEPEIALACVWKMMKVAPVEGPFLI
MIQHPLLPEMHDAERQMKATLDAVVACGLPTFVSSPNSDPGNQAMSRLLSDYANRYPKLVAYKNLSRAVFVNLMR
HASALVGNSSCGIIEAPLLKLPVVNVGSRQVGREHAGNVEFVDYNATEIEAALKKAVFDDQYRAQVEVAKNPYGD
GTAGKQICDVLVQETDQRRLLHWLILTEMTASAGYSAERIAARENEIGAVSEAFGFQSIHRLGYETAALDVAPIG
EIVKKIAVIFAQIHPSDVYLPFPGDAHTDHKISFDAALSCTKWFRAPYVRRVLAYETLSETDIVRRPGGLDFIPS
LYENITDWSAKKRSIIELYGEEVGDAPFPRSLEIVDAKEKVRGEAMNQVTVIAEAGVNHNGSLERAIEMIDVAAD
IGVDVVKFQTFNAEALVTRSAPKADYQKETTEESETQLDMLRALELDAQAHRTLIQRCAEKGVQFLSTPFDLGSL
DLLANELNVATLKVGSGELTNAPLLHAX

>Prorocentrum2 minimum JXLM01012202.1

TRAEYGLLYWLLKDIQADPELQLQLIVSGSHLSPEFGLTYQQIEDDGIVITEKIEMLLSSDSAVGTVKSMGLALI
GLADTFARLQPDVLVLLGDRFETLAAAQSAMLMKIPVLHLHGGEITEGATDDAIRHAVTKLSYLHATSTEAYRQR
VIQLGEAPERVQNVGAIGLDHLYRSTLLTKEQLAQALDFKITRPYFVVTYHPVTLADEPPLESFNALLSALDQFK
QYQIILT

>Condylostoma_magnum CVLX01013231.1

MLWDKTLATGTHLSKKHGLTKHEITKAGLIINEEIFMDLDDDRPLALSKSVGIGLMGFAEAYDRLKPDIIVVLGD
RFELISAVTPAFMANIPIAHLHGGEITAGAFDDSIRHAISKMSHLHFVAHDDYKARLCQLGEQPERIFTVGGMGV
DALGHLQFLCKDELEKSLDFTFLKRNLLITHHPVTVELGKARQELKALLHALDELKETGLIFTMPNADPDSDALA
LMIKNFAKDKAHVKCFASMGQLNYLSTMRVSDAVVGNSSSGICEAPSFHIGTINIGDRQQGRLSASSVIHCTGDY
QEIKQAIHKLYSHEFQDKIKQTINPYGQGNASTQIHEILKSHPLEELIKKRFHDL

>Paramecium_biaurelia JPFL01000669.1

GGYKMIRVGVLTSSRADFGIYLPLLKALQKEEAFHLELIVFGTHLSKFHGYTVEQIEREGFKIAKRIESLLLGDT
PSAIASAYGLTALKFAEYWQENEQSFDVVFALGDRFEMAAAVTASIPFGIKIAHLHGGETTLGAIDNIYRHSISL
ASQLHFVAAEPFAKKLKQLLDDEKASIYNVGSLSLENLKNIDFLSVEAFKNKWQIDLNIKTILVTVHPETVAYQK
NLAYCEETIKALEQLANEFQIVITMPNADTAGMVFRELAKKQQSIKIIENFGTQSYFTCMKYAKLMVGNTSSGIV
EAASFQKYVLNLGDRQKDRLCGENVIHVPFNHDLIVKNTLEYATKEYNGENIYFKSNPSEIIIQILK

>Paulinella_chromato ACB43177.1 P. chromatophora

MTTPPLVSIVLGTRPEAIKLAPVIMSFQKCGALRTRTILTGQHREMVNQVMKLFQLSFDKDLALMEPNQTLTHLT
ATILEGLRKEFLVHRPSLVLVQGDTTTALASAMAAFYEQIPVGHIEAGLRTNDIDDPFPEEGNRCLISQITKLHF
SPTIQSAANLQASGVIGEIHITGNTVIDALLSMSQKKSTVCQINGLNWKDKRVILATVHRRENWGTRLNGIAYGF
FKLLERHSDVALLLPLHKNPAVRKPLNDILKDHPRAFLTEPLAYDQLVAAMRSCSLVLTDSGGIQEEAATFGKPV
LILRRTTERSEAVEGGTARLIGTDTTDILNEASLLLKDDSAYRAMSQFNNPFGDGKASDRILTAARTFLNC

>Spizellomyces_punct KNC96568.1 [S. punctatus DAOM BR117]

MMETPPVKTVKILIDHEKLHFTKREIQSPRSIRISRRTRLLALLACTVLLFIFFVSSLDTSTHLVSQSRVTSKDI
EFLLPDDGGLNKSPRVMIVMGTRPETIKMFPIIRALNTHGINPIICVTGQHQEMVEPLLRLFDIKPHVNLNVMKS
GQSLNSLTERIVGRMTKAVTHYRPDWLLVQGDTTSAFVASVVAFHEKIAVGHVEAGLRTYKRYSPFPEETSRRLI
AGLASYHFVPTQHAANNLKAEGIPSENVYITGNTVIDALQWVASLEPSPEVASSLLEIEEAAKSHTSQYRLLLVT
MHRRENLGSPLISVCKAIKRIVDGFPDVHIVLPVHMNPTVQETVTQFLGNDKRITLLGPLSYEFFAQLLKRATLL

LTDSGGVQEEGTAFSKPILVLRNDTERPEGVTAGVAKLIGTDEDNVFRHVQELLTDKQAYAAMAIKTFPYGDGTA
GSKIVDIVLKGSAQIAVNRPTIASDKLVEAESAKTTLTNVSESTSAASIAPRPRSFVRAPTKPRQLNLTALLELP
SSYPENGDGKGLNGITAVISGYNRVEVIPRLLGSLFNQTVPPLEIWITVFASKSVAAIKQAVDDFANSHKAQKIP
IKFLQGDVQLKYFGRFEVGLQIPTKYAVFFDDDCLPGANVFRNFLHVQNIVGGEFRGLYGAKGHIVPVQNSNNYK
ETYGLGQVIHPEVITQVDLVGGIWFMEKDWIKLMFRENAVTWETGEDFQITYTLSKYANLPTFVFPVAASDPSSH
LVTPDYRAISASGDTTHGAISVNGKEMDIHRLRDYITFKHFTRGHTRVLMSEMWRPNEMRILYLVDTIQDARLFK
PLHEYIWATTLQKPVPNIRLRPFPVLIGRYPQSQLLSELGISWTLESFHTGVFDLGVASEFSRKSRAVDIATEVM
MSFQQVIETVRPDLVIVPNSPDDPAIVAAAITARSFNGFNVVAWNKKNAKDVLLPDESNSNERLTPLKDLVDGII
ETNEMVARILEEIAVASWLARRRDVRTDFRN

>Ostreococcus_lucimar XP_001422307.1 [Ostreococcus lucimarinus CCE9901]

MKDIESERFDRTAYSAEIKIAIVFGTRPEAVKMAPVIQAVARSSTLSAILISTGQHKQMLEQVLRQFSLQDKIQH
ELALMKPNQQLAELTSSAVRAVDGVLRSSKPDAVLVQGDTTTAFITSLAAFYLKIPVGHIEAGLRTRDIYSPFPE
EVNRQCISVMATYHFAPTEHAAKNLYDEGRRTNVFTTGNTVTEPSDRVIELSKVVKTVSTLRDVRLLLLTAHRRE
NLGEPILNIFTSIEKLLQEYPDVVVIYPIHLNPMSDHAPPTTTHLRRLLIVPPLDHADLLFMMKESFFVMTDSGG
IQEEAVTLGKPVLVLRDTTERPEGVLAGAAKLVGHGAESIYTEAASLLKDPDSYRSMSGSKKTYGDGNAAGNIVA
KEKQKERRGTVPKK

>Cupriavidus_sp WP_071014909.1 [Cupriavidus sp. USMAHM13]

MRKKIICVVGTRPEAIKMAPLINSLKNEDWVECKVLATAQHREMLDQVLGLFDICPDIDLNIMRPNQSLVELTSR
LLVEVDRVLSQEAPDAVIAQGDTTTVMTVALACFYRGIPFGHVEAGLRTWDMRNPFPEEMNRVIAGRIATWHFAP
TESSRGNLLKEGVPEREIVLTGNTVIDALLDVVTRCDHLVPNIPQGRRLILVTSHRRENFGEPFEEICRAIRTLV
ENNSDVEVLYPVHPNPNVKETAHRMLSGLDRVTLTDPLDYLPFVAAMRGAYLILSDSGGVQEEAPALGKPVLVLR
RETERPEAVDAGVVKLVGPDHARIVEEAQRLLDDASAYQAMARGVSPYGDGKASQRIVKVLREYFA

>Naegleria_gruberi EFC41069.1 predicted protein [N. gruberi]

MPRVVIVLGTRPEAIKCAPLISHLKSNHHSELEVIVLSTGQHSEILKQTLGVFHQHVDIDLKLMTPNQSISSFFT
LAFQKISLEFEKQGPISMVLVQGDTTTSLVAALAASYLKIPVGHVEAGLRSYDFNSPFPEELNRKVIDSFASLLF
APTEFSKAALVREGICPQHVHVTGNTGIDSFYSLKSLPPKIIPELLQNIRSFKTKHVDATTSSHRTVILVTMHRR
ENIPHFKEMCNAIKTISNTFGKNVMVILPVHPNPNAKSVVHEVLSSLDNVQLVDPIAYDIFPHVISEADIVVTDS
GGIQEESASIGKPVILMRDTTERPEGIYIGTIKKIGVNYHHIVKAMTDAIKDSQNSKLILSKHIFGDGKASMRIS
KIVRDFVTSKLPESSECSTQFIQDSIAESVYSQNFLPFESRKKELLERSKPKDPLTMQELFALPSAYTPATKTSD
EFSVTVVVGLYKRTGLVKRWMQALLSQTHQPKVVWMVFFASPKAEEIENEIQEAKLLATSNNISLFINKGEMQLK
YFGRFQLALQSPTKYIAVFDDDCIPEKRFLEAAIHTINTNNYRGILGTKGTPTAEDYFYGPVSGSEQIIEVDVVG
GSWFMEREWVKLLFREKMFSWATGEDWHLCSNARKYANIRCFVMPVSRTLETNSYSGDYMQISNNGDTTGRVQGT
SEARSLIIQKETQRGNRLMNSYRNNDLRTAFVFLSNQNEGRTILKMLSSLELPFTIQYSLGVADQSIAQIDSDEL
SKITKFKSFNDFMLSREFDTAQTNLSSAAETMYHFDMSIQQQQATAVILVGSSPTPSTLAVATACQINKIPVINF
VLTEKSNKLVESVSVLTVRSTPIDGNLLDETKRILTETLTKIFT

>Capsaspora_owczar KJE94661.1 [C. owczarzaki ATCC 30864]

MQANVLTPADSSSTTPLSASPVPSGGASLVGVAARIAVRPTATRVLSIFGTRPEVIKFYPVLKEMDRRSESLLSI
TCVTGQHRQMIDPLLSLFDIATDIDLNLMTQGQTLSNLSARLFTALAPVFEAVRPHLVLVQGDTTTAMIAAMCAF
YYRVPVGHIEAGLRTNDRYNPFPEEVNRRIVSVVGNLHFAPTNYAAEALRAERIDMQTVFVTGNPVIDAILLIRE
KAPSAAARQLLDRVEAVDAATNATLPAGQKARHILLTAHRRENHGEGIAAICRAVKRIIAQHKDVNVWYPVHLNP
AVCVPVRQELGKVERVHLLDPLEYDVFVHFLDRMYMVMTDSGGIQEEVTALAKPTIVLRETTERPEGVDAGVTKL
VGIHEDTIVKEANVLLTDEQTFAKMSCKCFPFGDGTAAKQIVDIMLMSVSEVKLSIGA

>Dictyoglomus_turgidum YP_002352477.1 UDP-N-acetylglucosamine 2-epimerase [D. turgidum DSM 6724]

MGSFKVISIVGARPQFIKLAPFSIELRKSGIKEVILHTGQHYDENMSDLFFKELEIPEPDYNLGIGSSSHGEQTG
RMLIGIEEVLIKEKPDVVIVYGDTNSTLAGALASSKLHIPLAHVEAGLRSFNKKMPEEINRIVADHLSDILFAPT
ETAVENLKREGIEKGVYLVGDIMFDALMHFSKLAKEKSKILEKLSLYPKGYYLVTIHRAENTDNPERLKSIFSAL
QELDKEVIFPIHPRTKNKVKEFGLEDYLKSRIRIIDPVGYLDMIQLEKNAYAILTDSGGVQKEAFWLKVPCITLR
EETEWVEIVKLKWNRLVGANKEKILEAVRNIKEGEDVNFEENYSAPKMREILVRELEKRGGEFK

>Vibrio_cholerae WP_046127216.1 [Vibrio cholerae]

```
MSEHQKKVAVFTGTRAEYGLLFWLLKDIQADPELQLQLLVSGMHLSPEFGETYQQIERDGFHIDEKIEILLSSDS
AVGTAKSMGLGVLGFADALARLKPDVLVILGDRFEALAAAQTAMILRIPVIHLHGGEITEGAYDDAIRHAITKLS
YLHGTSTEAYRQRVIQLGEAPERVKNIGAIGLDHLKRAQFMDVPALAQSLNFALTQPYFLVTYHPVTLGEEAPEV
SFQALLDALDTYPEHQIILTYPNADDGGRRIIPMLEAYAAANPQRVLAIPSLGQVRYLSAVKHASVVIGNSSSGI
IEVPAFDVPTVNIGVRQQGRLAAKSVLHCRVSKEEIEQAIQTAITRGYKADGDVIDNPYGQGDSSAQVIAMIKSL
HFEPCKSFYDLPFEPIE
```

>Rhodopseudomonas_palustris WP_011440433.1 [Rhodopseudomonas palustris]

```
MTKRKICVVVGSRANYSSIKSAMRAIQDHPALELQLIVAASAVLDRYGSVVNLIEKDGFRPHARVTMLIEGETPA
TMAKSTGLGLIELPTLFEQLGPDVVLTVGDRFETMATTLAAAYMNIPVAHTMGGEVSGTIDESIRHAVTKFAHIH
FPASQGAAERIIKLGELPRHVHMVGCPRIDLVAEILGRSSGGLDAGLFDLGVGQQFSVDEPFALVSQHPVTTEYG
TGEAQITLTLEAVREQGLAAIVLWPNADAGSDDISRGIRKWRERKLDDRMHFFKNLPIETYVNLMRSAACLVGNS
SSGIREGAYIGTPVVNIGTRQHMRDRGDNVIDVGYDKKQISDAIARQVEHGRYAMDPIYGDGTAGTKIADILVTE
RVDVQKCITY
```

>Rhodobacter_capsulatus WP_023918691.1 [Rhodobacter capsulatus]

```
MTREILFVTGTRADFGKIEPLALAARDRGFKVSFLVTGMHMLDRYGLTKIEVHRVQGATVHEFLNQREGDPQDTI
LAKSIIGFSDIFAELRPDLVVFHGDRIEALACALVCATNYIRSAHIEGGEVSGTIDEVFRHCNTKLAACHFVSSE
AAAKRVMTLGEPADRIHVIGSPELDFHARPSGVTLPEVMTRYDIPFDDYGVTLFHPVTSEAATMGRQAADLFGAL
EASGKNFVVIAPNNDPGSREIFAVLEQLPRERFRLIPSMRFAHFSELMKHAACLVGNSSAGVREAPFLGIPSLDI
GTRQTNRAEAPSLFSADAAEREKIAAFLATEWGKRYPPHTAFGEGRAAERFLEVLADEGFWQGGLQKTFADHG
```

>Ganoderma lucidum WGS BACH01000612.1 [Basidiomycota]

```
MKQSTRRLLFVTGTRADWGKLKPLAEAAAAEGFLTDFFVTGMHLLEDYGMTEKEIRKLPNVQICAYPNHRAGDAP
TTILSNTLTGLRDWVPQNKPDLLIVHGDRVEAFAACTVASQLSLRCAHIEGGEVSGSIDEIYRHCNTKLSTYHFV
SSETAKRRIISLGEDPRRIFTIGSPELDDHVRENGVTIGEVKHKYGIPFTEYGIVTFHSITSETDTIGDQARHLF
DCLSNSGKRFVVIWPNNDPGTEKIHAVISALDPSRFHGVSSLSFDDFSTLMKSCKAVIGNSSAGVRETPFLGIPS
LNVGTRQRNRAEDVPSITHCSAFDTPRIEEFLDSKWGQRFRPHHGFGRGTAAQEFVGTLNVDEFWEQDLQKVFYD
AR
```

>Talaromyces wortmannii MJVA01000852 Ascomycota

```
ISQRRRRIAFSSNTRADWVKLQPLADILVENGFDVDIFVTGMHMIKEYGLTFKNIEKHTKFGVFTRNTWAPGDSE
IQNATVTMEATFQLLQQADYDLLIVHGDRLEAKAAADAAHLFRCRLGHVEGGELTGGDDNKNRYAITAVADYHFP
SSQDAADRLICCGQRIETIFPIGSPDLDVFLRPSAIPIDHVLHKYGIPFKDFGIAPFHPNSAESETSGEQAMNFY
STLLASGRNFVVPRPNNDKGTENVQEVLDSLPSDRVCVVPNFEFNDYVVLLKQAGCVAGNSSVVVTSAPAIGKPC
LNIGSRQQGRTPPTNGLFNFSPNDRHGILRCLRENWGKTYPSNTHYGDGCAAERFFEALSSRSFWEVSQQKLLFN
QK
```

5 - Sialin, SLC17A and NanT

>Sialin_HSA NP_036566.1 sialin [Homo sapiens]
MRSPVRDLARNDGEESTDRTPLLPGAPRAEAAPVCCSARYNLAILAFFGFFIVYALRVNLSVALVDMVDSNTTLE
DNRTSKACPEHSAPIKVHHNQTGKKYQWDAETQGWILGSFFYGYIITQIPGGYVASKIGGKMLLGFGILGTAVLT
LFTPIAADLGVGPLIVLRALEGLGEGVTFPAMHAMWSSWAPPLERSKLLSISYAGAQLGTVISLPLSGIICYYMN
WTYVFYFFGTIGIFWFLLWIWLVSDTPQKHKRISHYEKEYILSSLRNQLSSQKSVPWVPILKSLPLWAIVVAHFS
YNWTFYTLLTLLPTYMKEILRFNVQENGFLSSLPYLGSWLCMILSGQAADNLRAKWNFSTLCVRRIFSLIGMIGP
AVFLVAAGFIGCDYSLAVAFLTISTTLGGFCSSGFSINHLDIAPSYAGILLGITNTFATIPGMVGPVIAKSLTPD
NTVGEWQTVFYIAAAINVFGAIFFTLFAKGEVQNWALNDHHGHRH

>SLC17A1_HSA NP_005065.2 sodium-dependent phosphate transport protein 1
[Homo sapiens]

MQMDNRLPPKKVPGFCSFRYGLSFLVHCCNVIITAQRACLNLTMVVMVNSTDPHGLPNTSTKKLLDNIKNPMYNW
SPDIQGIILSSTSYGVIIIQVPVGYFSGIYSTKKMIGFALCLSSVLSLLIPPAAGIGVAWVVVCRAVQGAAQGIV
ATAQFEIYVKWAPPLERGRLTSMSTSGFLLGPFIVLLVTGVICESLGWPMVFYIFGACGCAVCLLWFVLFYDDPK
DHPCISISEKEYITSSLVQQVSSSRQSLPIKAILKSLPVWAISTGSFTFFWSHNIMTLYTPMFINSMLHVNIKEN
GFLSSLPYLFAWICGNLAGQLSDFFLTRNILSVIAVRKLFTAAGFLLPAIFGVCLPYLSSTFYSIVIFLILAGAT
GSFCLGGVFINGLDIAPRYFGFIKACSTLTGMIGGLIASTLTGLILKQDPESAWFKTFILMAAINVTGLIFYLIV
ATAEIQDWAKEKQHTRL

>SLC17A3_HSA AAH17952.1 protein [Homo sapiens]

MATKTELSPTARESKNAQDMQVDETLIPRKVPSLCSARYGIALVLHFCNFTTIAQNVIMNITMVAMVNSTSPQSQ
LNDSSEVLPVDSFGGLSKAPKSLPTKSSILGGQFAIWEKWGPPQERSRLCSIALSGMLLGCFTAILIGGFISETL
GWPFVFYIFGGVGCVCCLLWFVVIYDDPVSYPWISTSEKEYIISSLKQQVGSSKQPLPIKAMLRSLPIWSICLGC
FSHQWLVSTMVVYIPTYISSVYHVNIRDNGLLSALPFIVAWVIGMVGGYLADFLLTKKFRLITVRKIATILGSLP
SSALIVSLPYLNSGYITATALLTLSCGLSTLCQSGIYINVLDIAPRYSSFLMGASRGFSSIAPVIVPTVSGFLLS
QDPEFGWRNVFFLLFAVNLLGLLFYLIFGEADVQEWAKERKLTRL

>SLC17A6_HSA AAH69629.1 (sodium-dependent inorganic phosphate cotransporter
[Homo sapiens]

MESVKQRILAPGKEGLKNFAGKSLGQIYRVLEKKQDTGETIELTEDGKPLEVPERKAPLCDCTCFGLPRRYIIAI
MSGLGFCISFGIRCNLGVAIVDMVNNSTIHRGGKVIKEKAKFNWDPETVGMIHGSFFWGYIITQIPGGYIASRLA
ANRVFGAAILLTSTLNMLIPSAARVHYGCVIFVRILQGLVEGVTYPACHGIWSKWAPPLERSRLATTSFCGSYAG
AVIAMPLAGILVQYTGWSSVFYVYGSFGMVWYMFWLLVSYESPAKHPTITDEERRYIEESIGESANLLGAMEKFK
TPWRKFFTSMPVYAIIVANFCRSWTFYLLLISQPAYFEEVFGFEISKVGMLSAVPHLVMTIIVPIGGQIADFLRS
KQILSTTTVRKIMNCGGFGMEATLLLVVGYSHTRGVAISFLVLAVGFSGFAISGFNVNHLDIAPRYASILMGISN
GVGTLSGMVCPIIVGAMTKNKSREEWQYVFLIAALVHYGGVIFYAIFASGEKQPWADPEETSEEKCGFIHEDELD
EETGDITQNYINYGTTKSYGATTQANGGWPSGWEKKEEFVQGEVQDSHSYKDRVDYS

>SLC17A7_HSA NP_064705.1 vesicular glutamate transporter 1 [Homo sapiens]

MEFRQEEFRKLAGRALGKLHRLLEKRQEGAETLELSADGRPVTTQTRDPPVVDCTCFGLPRRYIIAIMSGLGFCI
SFGIRCNLGVAIVSMVNNSTTHRGGHVVVQKAQFSWDPETVGLIHGSFFWGYIVTQIPGGFICQKFAANRVFGFA
IVATSTLNMLIPSAARVHYGCVIFVRILQGLVEGVTYPACHGIWSKWAPPLERSRLATTAFCGSYAGAVVAMPLA
GVLVQYSGWSSVFYVYGSFGIFWYLFWLLVSYESPALHPSISEEERKYIEDAIGESAKLMNPLTKFSTPWRRFFT
SMPVYAIIVANFCRSWTFYLLLISQPAYFEEVFGFEISKVGLVSALPHLVMTIIVPIGGQIADFLRSRRIMSTTN
VRKLMNCGGFGMEATLLLVVGYSHSKGVAISFLVLAVGFSGFAISGFNVNHLDIAPRYASILMGISNGVGTLSGM
VCPIIVGAMTKHKTREEWQYVFLIASLVHYGGVIFYGVFASGEKQPWAEPEEMSEEKCGFVGHDQLAGSDDSEME
DEAEPPGAPPAPPPSYGATHSTFQPPRPPPPVRDY

>SLC17A8_HSA AAI43397.1 [Homo sapiens]

MPFKAFDTFKEKILKPGKEGVKNAVGDSLGILQRKIDGTTEEEDNIELNEEGRPVQTSRPSPPLCDCHCCGLPKR
YIIAIMSGLGFCISFGIRCNLGVAIVEMVNNSTVYVDGKPEIQTAQFNWDPETVGLIHGSFFWGYIMTQIPGGFI
SNKFAANRVFGAAIFLTSTLNMFIPSAARVHYGCVMCVRILQGLVEGVTYPACHGMWSKWAPPLERSRLATTSFC
GSYAGAVVAMPLAGVLVQYIGWSSVFYIYGMFGIIWYMFWLLQAYECPAAHPTISNEEKTYIETSIGEGANVVSL
SVGLLSAVPHMVMTIVVPIGGQLADYLRSRQILTTTAVRKIMNCGGFGMEATLLLVVGFSHTKGVAISFLVLAVG

FSGFAISGFNVNHLDIAPRYASILMGISNGVGTLSGMVCPLIVGAMTRHKTREEWQNVFLIAALVHYSGVIFYGV
FASGEKQEWADPENLSEEKCGIIDQDELAEEIELNHESFASPKKKMSYGATSQNCEVQKKEWKGQRGATLDEEEL
TSYQNEERNFSTIS

>SLC17A9_HSA NP_071365.3 isoform 1 [Homo sapiens]

MQPPPDEARRDMAGDTQWSRPECQAWTGTLLLGTCLLYCARSSMPICTVSMSQDFGWNKKEAGIVLSSFFWGYCL
TQVVGGHLGDRIGGEKVILLSASAWGSITAVTPLLAHLSSAHLAFMTFSRILMGLLQGVYFPALTSLLSQKVRES
ERAFTYSIVGAGSQFGTLLTGAVGSLLLEWYGWQSIFYFSGGLTLLWVWYVYRYLLSEKDLILALGVLAQSRPVS
RHSRVPWRRLFRKPAVWAAVVSQLSAACSFFILLSWLPTFFEETFPDAKGWIFNVVPWLVAIPASLFSGFLSDHL
INQGYRAITVRKLMQGMGLGLSSVFALCLGHTSSFCESVVFASASIGLQTFNHSGISVNIQDLAPSCAGFLFGVA
NTAGALAGVVGVCLGGYLMETTGSWTCLFNLVAIISNLGLCTFLVFGQAQRVDLSSTHEDL

>Basidiobolus ORX71022.1 [B. meristosporus CBS 931.73]

MSEEYSWSSTTQGLILSAFFYGYICTQVLGGFLADKFGGKSVLALGAFLWSLFTLATPWVAHHVFLLFACRMLLG
AGEGLGFPAIHSLIAQWIPHHESSRAVATVTAASYAGAILALLISAPIAASSLGWKWIFYIFAIMGFGWNIPWYI
FGASTPDQCSELSSLRSMYDGKKDNQIPSYTPLGGDLESVTEGGEEYHRDLDSSKAVGEEEVLVQNNQQRLDNRI
PWCKILRAKEVWAILINQFCNSWGFYVLLSWLPTYYKDVFNVDLNELGYFSVLPYIVQGLVGLASGFIGDYAIYR
LGIPVVTVRRVAQCVGMLGPGVFLLLAGYTATSVTIGMVYISLALGFNSLTLIGVSISQLDIAPRFAGIIFGIGN
TAATLPGILGVTVTGLLLESKEAWPLVFGLAASFYFVGAWTWLFWGGGSQIVIP

>Aureococcus XP_009034535.1 [A. anophagefferens] Stramenopile
MSKRLLLVALGVCAGSAYSLPALRPKGLRTRHTLHAPTTHALVAAATPAAGDDAPPAAELRGGASGEPGGTLVLL
GAVVMLFVSVHRSLFSVGIVPIQKELGLGASTVGVLQSAYLAGYALTNAPGGAIADRLGGAPVMLACLAAWSVAV
ALMPVAAASPAPVAALVALRLLFGLASGPALPGSLAVVSRWLPLAKRSSGIADVFVFFNAGNAAGLLLGGLIPVL
GWRALMVGGGAAGLAWGAGGLALAKSIAAKHDPPEDDAPAAASSPSGGLSLRQVGQLAALTHVHNCLNWSFFFMQ
AWLPTYFSQELGLELSKSAGLSALPMVTMALGSKVGGQAATRLIAEGWAPFDVRRLMIAISSLIPAAALLALGGV
ADAGVAVACLVLALGAHSFSSAGYHAHIADVAPSSSGKILGFTNTVGVFVGIVANVVTGRVLEATGSFRACFALA
AAIYASEFAVFFGFVRGGRLV

>Ciona1 XP_018669669.1 sialin [Ciona intestinalis]

MMNIDEPLKSSENVSFPRGVPRKKFYVRQRYVLASLGCMGYFLISCLRNCINVTVLSMVRWNNTETNVTDAINLT
IEKSKGFEWSSSQEGLFLGAYFYGYVCTNILGGWLGNKFGFPIVFGLPIFISALLSIATPFAAYTSFPLVIACRV
LMGLLQGASVCAFQGCWSSWAPPLERSLLNSIALSGFPFGLGFANFFAGYICGTLGWEAVFYILGGVAIGWSVVW
IIIVSDSPRTNRCITAAEVAYIEKSIGFTSKVNLQDMLQSQVVPWGAIMRSRSVWAIFVAHFCDNWSGYTFNAIL
PTFMSKIFNFNVFQSGAVMTAPYIAQVVVSILAGFMTDAARQRKWISTSAARKVNTIIAQSFLNLFMIIACYCTD
STTVIVLFVVGMSLRGFTYAGHNSSPIDIAPMYAGVVFGISNTIASITGFLGPLTAGLVITDKTSIGQWQTMFWI
NGALGFFGSIFFACFASEVERKWAQIKDSDKETDLKTNLKSGLTDDEC

>Ciona2 XP_002122757.1 sialin [Ciona intestinalis]

MTTISSSEEESINTSTSTSPILNSNENSNKEYFKKPPTVKTWLSTRFCLAYLACFGFMNVYALRVNLSVAILSMV
NSSYMEIHTHNNISDACPASPSHKNSTTGEFNWDAHKRSLVLGAFFYGYILTQLPGGYLAGRFGGKWLFGLGILC
TSVLTLLTPVATRTSFVLLIILRIFEGIGEGVTFPAMHSMWGIWAPPSERSRLVSITYAGCHLGTVIAQPISGIL
CASTFLGGWPSVFYVFGTLGILWCIVWFIFAHSKPADHPRITTSELNYIQSNLEPKDDSVSVPWWEIATSKRVWA
ISIAHFCNNWGFYTLLTCLPTYLKDVLKFDIQQDGFISALPYLVMWISINFNGLLADYLREKEILNTTQTRKIFN
AVAFIGPGIFLVASGFVGCNKVAAVSLICLATAFNGAGFPGFNTNHVDIGPRYAGILMGITNTWATIPGFAAPAV
VGLLTENNPSRSQWRIVFYIAAGVYLTGTILYSLMATGEEQEWNRGKKKVELDQKQPLLQ

>Chrysochro1 sp. JWZX01001700.1 [Chrysochomulina sp.]

DAKIEGRLLSAFYWGYAVSQTPLGMLAQRINAQHLLMLAVLVWSASSVGVALVGNSPEAVPAIFALRVLVGMAEA
ANYPCQMQLLSVWAPYKERSRFWSFAATGEALGTIVALGVGPALVHAYGWRSIFFACAASGGVWALLFGLLAASH
PEKHRGISTTELAHIQASRPPRPPPVFTPFWRILTNRPFLATIVTHCAYNWGYYVGLSWVSKFFNSKYDADLAQL
GLLSIAPYIALLIATCLSGIVADGLENGCGASATCARKVVNSIGMVGGALGFALLAIVCGPPGRLPRSQAYVGAG
CLSVAIGLGGFAVGAGYWANFVDLSPRHSQVLLAISNSFASVPGILGVSLTGSLLSSTGDDWRIIFLIAGAVEFG
GALIFIAFAEAAEQQFDDDAGPRGCMWRSSRRSRHAFLAGSTSRSVKLLDEHEGMYGLHG

>Emiliania huxley AHAL01000302.1

MRESLVLLIASAAALRTPLPPLGYVPAALSASAPPRTQSLLLRSAARATALTAAAAAEIKPQLPPPEDVPSARVT
VLLLCCAIGAVCALDRVLISIAILPMAEQFAYSDSTKGAIAAGFSLGYCLGLLPAGVLASSGSPRQVLLGGLVVW
SAATCATPLAAASSVPALLATRAVMGVGEAAAVPTLQAVAARFVQPERRSLFWGCLTASLSCGTIAAYVLAPPLI
AEQGWPFVFEAFGAAGLSIAVLWAVLGADAPRELESAGASSSGGGSSSSRIGDAGAGGGDSAGGGDGGMGEVPWR
ALASSRPVWAMTAAHCSSNFFMYFGLSWLPTYFSYQFGLSTADASTAALYPFAAGAVGSLAAGAACDALVSSLHF

RRTDARKVMQSVALGGPALAMLALCLLSAGAGGLQLERDEAEALFVVAVGCQAGSAAGYGCGAQDISTRLSSLIY
GGTSVFAVIAGASGQYFTGWLLEQNGRDFTPMFALVVAVELAGLVAWNRCLVFTLFCEQTRNRKGDRQRQDRPRE
PQQPRHRRSSNIIECVRRAEASAQSAFCPGVTSTGARLYRCDFRRRHDTLVLKVE

>Guillardia tetha AEIE01001896.1
MAASSSAGGAGPSAAPAPSSFSLSTAHVRPTPLRNDAGFTGRGEGGEANEDGGGEEEEEDGGRVDDPWEEKKLLH
DTPRGYIPKRFILSLLLHLGLFVVYALRVCISVAAAAPSAHALPSNSSVPTEVTGVSMYTEYSWTNTEQGIILGS
FFNGYITTQIIGGILARRYGGKSVLFGGVLLASLFTFLTPPASKNFTMLVICRTLMGAVEGVSFPAILTIIGHWA
PAQERSTMVGFVYAGAYTGNVVTFPLSAWIMDTYGWRTIFYFFACLGFLWCLLFHLFTTSTPSQHRSMHAAELNK
ILATTQVAEDVPATVPWKKILVCMPAWALFVVHTCFNWAFYTLLTQLPSYMALVLGFNMQQSGFLSSMPYLFMFI
VSILGGMLADWTISRGLLSRTRVRKTWMVTSLMVPACCLVLCGYASSWPVAIFFMTCALGFSGLSNAGYSANYLE
ISAGLSGILISLGNTLATVPGMVSPVLTGVIMDAHGCSIEHVSSCQQAYQTVFWIAFMVYTFGAVFYATFATSER
VLKF

>Cryptosporidium ubiquitum LRBR01000265.1
KDLEREDLQDECDYRRNFSDVPNFEYGSNSPLDLKSKQEATAISCISLVSMLMFSIFLCYADRIIMPSCIKSISE
EFGFNKSDQGFILGLFYGGYIWTQIIGGYISDTSKLGGKGVLFFGVTFWSLCMIFTSFLSYMGITGFIICRIFLG
VGEGVSFPALNSIVGHHIPSKYSSTVISIIIASSFIGGGFAAFVTPPMILSLGWRGPFYVFGLIGVFWSIVWLFL
DVKSLSWTNKPSIYEFYDTKEKNDLEIQLPDFQFKKKLSFEISPKYQAKTDLLGSFSIGLDCLNKHEDEKRDSSI
EYNIENLQEKKSRYWPIFNFVKVLLLNKSIFAIIVAQYCHGWTQFGFVTWMPIYFTDVCKVNSAYLGYYTTPPWV
LQAFFVIFFGFLADKLVSSSIKPIIVRKLFQSVSMFVGAGCQLALVLLNNMGLTSASYAIMVISLMFIFNTMSGG
GVTVYQFDIAPEFPAVVYAIGNTFGTIAGLFSVSLTGLILNRSEVNLQVIGSELGGSVLIERWKWVLAIYAIHNI
IGGLLFVLLAD

>Sphingorhabdus WP_074203861.1 MFS transporter [Sphingorhabdus marina

MQKRHQLVLMAFLAIFICYMDRVAISVAIIPMVETYGWDLSTQGLVLSSFFIGYLLTQVVGGKLADRYGGKVVLG
FGVFIWSLFTLVAPPAAALGITVLIVARILMGMGEAVTFPAIYALYARWIPVKERSRSAGFSNSGIPLGTVFALL
ATPIIVAQWGWEWVFYLFGGVGFIWCVIWYYAIAPTPSRQSGISQSELDRISADTIAEAPATATPWGALLGNMPV
WAIIVAHFCNNWWFYVLLAWLPTFVTQGLGVDYASVGLFAMMPHIALFLCMNISGLVADRLIGRGMTITHVRKLM
MIIGFGGSIIALLLVGQVESAVGAIAIMTVGSAIGGFAVSGFFVNHMDIAPEHAGTLMGITNTAGTIPGIVGVMV
SGWILDSTGSWALVFQVAAAVSAFGLLFYLLFGSGERQFTPETSQPATSH

>Chromera vela ARZB01002148.1
LSVAKDPVAESLHLSDRQMGWAMSIFALGYALFQTPAGLLADKYGPRKILTAVVALWSIFTALTGAAWNYITLLI
VRFLFGAGEAGAFPGMARAIYSWIPLSERGIVNGINFSGGRIGAAFALPLVAWLMDLVGWRLTFVLLGAIGILWG
ILWFLWFRDDPKDHKGMSDPEVNYIREHTNRDVKEEKKVKLDFGKLFRSKTMWQLMGQYFASNFTFFFCLTWLFP
HIKEKYGLDALEAGFYASAPLIFGALGNWFSGFLVDFIYKKNHWALSRKVPAIIGFLLASIGIVASVYMDEVTPA
IILISIAVFGADMTLSPSWSVCVDVGKQFSGAVSGTMNMAGNIGSFLTALAFPYLLELTGSEVPFFYLAAGLNLL
AIV

>Cellvibrionaceae WP_086931727.1 [Cellvibrionaceae bacterium 017]
MNNTKTHSVDHSNEVAPKVPLRYWIVVATFLLSVLLYIDRICVSVAKESIVSDLSLTDKEMGWVLSAFALGYALF
QAPSGWLADRFGPRKILTTVVILWSLFTALTAAAYNYMSMLIVRFLFGAGEAGAFPSISRVNFSWIPIEERGLVT
GINFSGSRLGAAFALPTLAWLIAVIGWKVTFVVLGLIGVFWALLWFWFFRDEPQSHPFISTHEKHHILEKRQKNT
TTTSSINVSTLFGSGNMWLVMMQYFCSNFIFYFSLTWLYPHLKAKYALDSVSAGLYASLPLIMGACGNWLSGWWV
DRIYAKNQWQLSRRLPAMVGFACAAIGLVCSLYMENVIGAVLFLSLAIFGADMTLSPSWSFCVDIGKNNAGTVSG
TMNMAGNVGAFVTSLAFPYLKDWFGSVTPFFLIGAGLCVMGILCWSKMDAEKPLT

>Congregibacter WP_008293696.1 [Congregibacter litoralis]
MTQSELARGFGRVSRWPKRHVLVGFCFLATFICYIDRVNISVAIIPMAEEFGWSATTKGYVLSSFFVGYLLAMIP
TGWLANKYGGKLLLGVALIGWSLFTFLTPIAAGVSLGALLLTRVLMGMGEAASFPGVYNLLGRWIPKQEKSRAAA
VNLTGIPLGTIFALSTTGLLVSAYGWQSVFFVFGASGLLFAVVWLRVVHAKPSVHPTISAEERALLAELENSEAD
DKEPIPWGLFLRHPSIWALFINHFCANWTLYLFLAWLPSYFRDVQGLSIAGSGLFAIGPWVCQFLAGNLSAVVAD
RWIAKGISVTFVRKFMQCGGLIGGAAMLLLAAQASTPGMALFTLCAAFGVSSMAWAGFACNHLDVAPKHADVLFS
ITNIGGTLPGIVGVALTGVLVDLTGGYTATFVVAAGINVFGAIVWLLFATGEPIVGAPQETQA

>Enterobacter WP_034828518.1 [Enterobacter cancerogenus]
MNKARYLLVGVAFTLTALLYIDRVAISVAKESVTGEFGLTDTEFGWVLSIFALGYAIFQAPAGAMADKWGPRKML
AIFVLIWSAFTGLTGLAWGFASLLVFRFLFGLAEAGAFPTFARAIYSWLPAGERALAQGINLSGSRLGAAFALPF
VAWLLTTAGWRESFLILCAVGLIWAVCWYAWFRNDPAEHKSISKAELAIIEAGRESNVATSENSTPPDMSFRSGN
MWLLMGQYFASNFTLFFALTWLFPYLKKTYELDAVTTGFLAAAPLVAGAIGNWTGGAVVDFLYRRGLGMYSRRLP
AMFGFALSAAGMFGCMSADSAVSAVAWLCLAVFGSDITVPASWAYCIDTAGKKSGVVSGKMNMAGNLGSFVTSLA
FPYLMLAFNSTTPFFIAAMGFNLLAVIFWLRLSSGKREQRVPLNSN

>Micromonas pusilla ACCP01000320.1
MHGSACTTARAPCAPPRRASAPSRRGATRRAVASSRASSSSSSAETARRPPSSRVVELRASVSVSAADAPIRRRG
RRGSRPDRSRSSATAFASSSSSSSSSSSSSSSSSSSSPSPSSPSSPSSSSSSSSYRFVVVALIALALLLCNADRVV
MSVVGLPLANLNGWSPSVVGLVQSSFLWGYALTPLVGGVLADKIGGDAVLMYGITLWSLATIATPFAATSASLPL
LLLTRAVMGLGEGVALPCMNNLVARWAPTRERSRAVSVCMGGFQSGSMVGLLAAPHMLRVGGVHGPFVAFGALGL
TWAVAWRFAASAYPRGSRREGRVSDAELSLIEDGGAVTEEKQWQQSEEGGTNGKGGGKVPFRLLLSKMPTWACVV
ANFVNNWGYFILLAWMPLYFREVVGLDLASASYFSALPWATMAVVGVCAGALADWMIARGVSTTTTRKLIQGVGF
LGPAAALVALTFTSTPAQALTALTVAIGCTAFTQAGFLVNFQEIGPRYVGALHGMANTAGSAAGIVGTYGAGVIL
GKTGSWSAVLLITAGVYAFGAVFWLAFSTGERVFD

>Bathycoccus1 sp. FQSR01000298.1
MTTVASIGTSDLARASGNGLYGSDGPLSGERGRGPRRRFHFATTSRRARNDCGLRVSNDARSDTFSFSSFASSGS
SRANYRGSNASSSSFRVFSTAAIDGGETGEEIQEENNNYRFTILFLVALALLLCNADRVIMSIAGLPMSKMNGWD
VKVLGLIQSSFLFGYALTPIFGGVLADEIGGARVLLGVAPLLMSKFGIAGPFYVFGTIGVAWAAVWNVCATSYPR
VNERVGKVELKFIEDGGAIVDSSNSRGGEVTVVAEKKKTPWKMLLSHPATWACVVANFVNNFGFFILLAWMPKYF
NDVVKLNLATSSWFSALPWATMAVSGVFAGILADRMLSEWKVSTATTRKFIQSVGFLGPALCLLILGLKGFAITP
GFALTTLTVAVGCTAFSQSGFLVNFAEIGPKYASTIHGVANTAGSIAGMVGTYVVGWILSHSVESGWTNVMYMTS
AVYVVGALVWLTFMSGEQVFF

>Bathycoccus2 sp FQSR01000361.1

HEKEASSRAFDVSNNKRQVIAKAGGGASAEDTSGVPESELFDLSEGTNKKRWAMVFSLFVAFVLCNLDKVNMSVA
IVPMAKSFGWTATQKGLVASAFFWGYAFTQIPGGWLSSKYGGKAVLFYGVVLWSLGTLIAPWCAGLGMGPLLASR
FLVGLGEGVAPSAATGILAKTIPASQRSKAVTATFGGLDVGSLLGLLIAPPIILFLGGWQAVFYLFGVLGFVWGL
WWWFGYANDKSVDMKETEADSSKVSGGLNIPWVKFAKSKEFWALMVAHFTWNYFSYGLLAWLPSFLSSALNVSLA
KSSFLSILPYLSTVAVTTLVAPIADSLENKGILSRTDVRKMSQTLCFGGGAVALSTVGFIVSRTPPASVTNTTIV
MIMSALAFCFGMGAWVRTGLFCGHQDLSPKYASIMLGVTNTAAAIGSLLSTFFIGYFMEVTNGSWAWSLFYPIAA
LQVASALLFSALWKSTPIDFDP

>Dictyostelium deminutivum FLTF01000036.1
MEGGSPPSSKHPSYTYQKDMMTSIDTSTLSNSIASLSNSIEAPISHGSGQLIFPTKGGRYSRFSGEIPKYYIPRR
LFLIVLGFYVMQIPAHFLCNRFGGKRVFLIGVTGSILCTIVLPPIAHANQSALVLIRVLTGLSQGIAYPTMNWLM
NQWIPPSQRTSSAAIIWSGAYIGTIVADFSVPKIIESHSWDVCFYLFGAVGLFWALLWLIFLKDDPANAWGIHPN
EVHAIKSQMETPGEPATATETQYLLNNPTSYNINSTDGADISSSSQEPDLKVVLKKLFSNSGIYAVLIYFLTTSF
GFYMLLMWYPTWLSKVSGLTSGSTLGFFSVLPYIGSFALSNISGLITDYLLSRGVRKIVLRKVFGILGGVLPGAL
LLIISFSDISSSNQIAAMVLTIATAGFSAAGVNVNTLDLAPNYAGIVMGIANTVATIPGIVGPLIA

>Amphiamblys sp MKPU01000040.1 Microsporidia
MSESEPFLTTETVEEERTRVRHVFALLCSVGIIIAYADRGNMALSIVPMAEEYRWSMVEKGSVLSGFFYGYVVTQ
VLSGWAADLFGGVNLAFSGMVVCCAGILCIPFAASRGVWALFLCRAVLGVFEGLVFPVFHSMVCRWIPPCERSFT
VSLFTASMQLGMIFSNILSPLSIESSLGWRGVFFVSGAVGLVWAGVWLFFMTNTPREHRSVSEKEAVYIEREIRR
QEKGDETEVQDYLCGCTMLDAFDTDPRGIPWRGVFSSGVFWAVLATQFFNSWCMFVFQQWLPTYYRAVFQTDAFA
TGLSVVLPYSVQCCVALCVGYFGERVVQQARRRTLLRRVAQVCAMCIPASLLLVVVLCPASYTVSLCVFTVCIAA
NALSIIGVQVAHMEIGPRYGGVLFGIINTVCILAGLVGVKTSGWILEATGRWDVVWGVCCLSFAAGALVWGCFYR
AELLFS

>Strongylo6 XP_780445.3 sialin [Strongylocentrotus purpuratus]
MSGNICENDDGLHDSMEHRPLIRSTDGSEEVKNKEEFLVAPKVPGLISARHALAFLAFLGFVNVYAMRVNLSVAL
ADMVNSTTTVNSTQISCPVNVSQSNTTQKEGEFNWDSNTKEQILASFFYGYILTQIPGGFLGDIMGAKWLFGSGV
LCTAIFTLLTPVAARTGLPWLIVVRVIAGIGEGVTFPAMNAMWANWAPPVERSRLLTFTYAGSHFGTVLALPISG
VLCNSDFLGGWPSVFYVFGVCGVVWFILWIALVHDKPEKHPRISPEELQFLQKAIKPRDKSLKVPWFKMLTSVRL
LAISLSHFSNNFGFYTLLTNLPSYLKFGLGFDISQSGFLSAVPYLVMWITINAGGQVADFLRGRYILSTTNTRKL
FNTLGLVLPAAFLVITGYIGCNHVLAVAMLTLAVGTGGFAMSGFNVNHLDIAPAYGGLLMGITNTIGTIPGILGP
SMVGLYVTNQYDISQWQIAFWVCCGVYMFGAVTYLLMGTGELQPWADPELQAKPCQRPGDKKDTEGLPYCQYDTS
NSVNGGLSNTGQSGVVMS

>Strongylo5 XP_783585.2 sialin [Strongylocentrotus purpuratus]

MAPRLDLEEDKAIVCDILHGKDAEEGEGKPLLQSNRRPANTLCSCRYIVSYISFLGIACAFMNRVNISVTMTAMA
NSSYSSTYDLFNSTAELCPARSNNDNEENVREGEFPWDAHTQEQILAAIYYGFPVLQVPAGILADKYPQSCIWLI
GVGYLLSAIFTLFVPLAAYAGGAPLIITLRILSGLSESGTYPGLYSLMSRWAPPADRSKLLAIVFAGSSVGQIIA
QPISGILSESDFLGGWPSTFYLFGSLEVLWCILWFLVIYPSPMAHPRISQEEKDYIMAELKLEDEPPKDYPWKHF
FTSLPLLAVVVADFALMWVLYSLTSNLPIFLKEALRFDISQAGILSAVPHIVFFIFILGGGVLADFLLSHTNFSI
TTVRKFMTTIGILPSGIFLVLAGYVGCNAPLAITFISLGLACTGLAYSGSCLSMMELATPYAGMVVAVSYSIATF

TGFISPAVVAMYTENQADIAGWRSFFWVTFGITVVAWLLFMIFGTSELQPWAKGDRRKDKTEHRETKYGGIIDNG
LEYNSEREEILPPDYT

>Strongylo3 XP_795625.3 sialin isoform X1 [Strongylocentrotus purpuratus]

MGCGLSFFADDDVKSSHHETNQDDDSERHRPPSPRDAHDINLNRIKTISKSVAIDEKLGVVPHENVDEPVMPPNS
SCSTRYLVAYLACLGFLTAFMGRAALSVSMTAMVNSTIDHELYEMNHRNQSFDFQVCKANGPTNASAKGQDGPFQ
WSSWTQELILASFYNGFPWLQLPAGYLADANGRAAVWLLGFGYGVSAVCTLLTPLAAHLGVPFLMATQVVSGITQ
GWTFPVFIALMSRWAPPSERSSLVAIGTSGIPLGQVIGQPVAGLWASSEFAGGWPSAFYFSGALGVLWFIVWMCL
VYPSPSSHPRISSKEREYIEKSLLHETKKAAVYPWKSMLTSVPVLAVCIADFSLLWILYLFTTNLPIYLKTVLQF
DIRQTGFLAAVPFVFYWLVWTVGGRFTDFLIARTKFKITTIRKLLTFLAFMPSSVLLVAMGYVGCNTSVAMVTLT
LGLSITGLAMSGASMCQLDFAPRYSGIISAVVNMFAATSGFLAPLLIGRFTENQADPRGWKIVFWTSAGIMMFGL
VFFLIFGTAEIQPWARKTEDVNKNGTDDKSRRAESDVSLFLPQCKSTSV

>Strongylo1 XP_789996.4 sialin-like [Strongylocentrotus purpuratus]

MEEEYTAYSKGLDRSLERISSRTNNEDSEKQPLLFSKKDPGSKLKWSTRYFVAVMACLGFASSYGSRVNMSVAII
AMANRSYSTDYSQNQSSTELCPQHGDLYSNETGGRSKEGEFPWDAHTQELILSAYYYGFVLLQVPAGILSDRYPK
SCSWIYGLGVFLSSVGSLLIPLAAHHSVTAIFVTRFICGLAESGTYPSMYSFMSRWAPSADRSILLAIVYAGLPI
GHIVMQPISGLLAESSYGWPSSFYMIGVIGIIWFVFWTIFIYDSPNSHPSINSEEKEYLIKELQLWKKPPKSYPW
FRIITSLPVMAVGMADFAILWILHSLTTNLPIFLSEALRFDVITAGALSAFPYAILLMTLLGGGFLADYLISRTK
LSRTVIRKLMTSLGLIFSALFMVSSGYTGCNAAATITMLSLGLASTGLVYSGASLTQMEFATPYAGIVTAIANTF
ANTPGFLSPLIAGVVTENQADMLGWRKMYWIAFGIAIFAEVIVLLFCTSELQSWAVIEKDDQKTEKYKEVK

>Strongylo2 XP_785484.3 sialin [Strongylocentrotus purpuratus]

MGSPDPIEEDCNHRPDHGPNNETISARNAHDNGYDDDWHSIELGEKKPLLGDKEGQGRKEGGCYVPKRYELLFLA
WLGTAMMFVMRINVSVTMAVMTNATFTTQAEDPNRTAKTCRSPNSTSEDENGQDGGEFLWSSHEQELILAGFFYG
YAAAQIPGGWLTDKIGGTRVFGISMFLSAAVSLLSPIAAKLGYSYFFAVRILCGIGEAGTFPALGSMVARWFPPQ
DFSAAFSIALSSAKFGTFIGTVLSGLISGSDLLGGWPMTYYAFGGFSLVLVVVWMARIYETPGRHPRISSKEKQY
LSTTVITEKTEKISIPVVDMLTSIPVWTIIVTSFCGSWTNQAMFTNLPIYLKHVQGMDIELIGIAAAIPFAVESF
FLVVGGMISNKLIKRQVLGVITTRKLVTFIGFALSALCFLLLAYVGCDTVLAVVFMIGAVGFNGICTCGYYVNLM
DIAPRLAGSLIGLVNSSSAFSGAISPYVVGVLTPNQSDITGWQTVFIICCGINSFACLVFLVFGSGKEQKWAKQP
GKDDS

>Amphimedon1 XP_011404686.1 sialin-like [Amphimedon queenslandica]
MAKSEKEALLKERCISASPEKEELSHWEAAKMLWHQDCCPCLPARYILAVMSFFGFVIVYALRVNLSMAIVAMVG
NSSNETDHDHFNWTSTQQGWILSAFFYGYIITQIPGGFLAGVIGGKLVLGLGIVITAALSLLTPLAARIHFGALI
ALRVLEGFFEGVTFPAMHAMWGKWAPPAERSILTTITYAGPFFGNVISFPLSAVLCVYGFAGGWPSVFYTFGILG
IIWYIFWLLLIFDTPAKHPRISEREKEYIEGGIADVNVGSEKVPVPWLQILTSPAVWAIIVAHVSVNWGNYIMLT
CIPSYFHDALGLSFGQNLIENGIFSGIPYVGFVVSTIIAGQVADFLRKKWISTKNVRKGITLIAFVFGAAGLLVT
GYFGKKKLDSVIFLTISVTGVGLANAGFNINHIDIAPRFAGVLMGITNCAATIPGIVAPTVAKYIAQKPPEGSDE
RHIYQAEWREVFIIAAEVYLFGAAVYLILAEGTKQWWADGVKRAQKETKDGLIVSQEQKTTIQEKNGSLLGSGSR

>Amphimedon2 XP_011404487.2 sialin-like [Amphimedon queenslandica]
MASTFSMDEREALLHNRCNSPDSGEPQAARWSVGFKKAWRRDYCPWIPARYTLAIMSFLGFVIIYALRVNLSMAI
VIMVNNSANGSANNQFNWTSTEQGWILSAFFYGYVITQIPGGLLAARYGGKTVLGLGIVITAALTLLTPLAAKLN
LGVLIGLRVLEGLFEGVTFPTMHAMWGKWAPPAERSTLATITYAGPIVGNVVSFPLSAVLCVYGFDGGWPSVFYT
FGILGIIWYIFWLILIFDSPANHPRISKAERDYIESGIPDADKMVPIPWFQIFTSRAVWAIIVCFISYGWGGYIM
LTCIPSYLHDTLGLSLGNNLIENGIFSAIPYXGYAIVTIVGGRVADLLRKCISTKNVRKAMTISALVLGAAGLLV
CGYFSKSKFDSVLYLSLTVGVSALIQAGFMINHIDLAPRFAGVLMGITNTFGTLPGIVSPVVAKYIAQKPPSGSD
VKHIYQEEWREVFLISAEIYIFGAAVYLILADGKKQWWADGVKKERKNYSIQ

>Amphimedon3 XP_003387261.1 sialin-like [Amphimedon queenslandica]
MVQRLTYIDLPARYTLAIMSFLGFVMIYALRVNLSMAIVIMVNNSANGSTNNQFNWTSTEQGWILSAFFYGYTIT
QIPGGLLAARYGGKTVLGLGVVITATLTLLTPLAARINFGALIGLRILEGLFEGVTFPTMHAMWGKWAPPAERST
LATITYAGPIVGMVVSFPLSALLCVYGFAGGWPSVFYTSGILGIIWYIFWLILIFDSPANHPRISKAERDYIESG
IPDADKMVPIPWFQIFTSRAVWAIIVCFVCYGWGGYIMLTCIPSYLHDTLGLSFGNNLIENGIFSSIPYIGYAVV
TIVSGQVADLLRKRGISTKNVRKGMTVSALVLEAAGLLVCGYFSKSKFDSVLYLSLTVGAGALIQAGFMINHIDL
APRFAGVLMGITNTFGTIPGIVAPVVAKYMAQEPPSGSDVKHIYQEEWRKVFLISVQVYIFGAAVYLILADGKKQ
WWADGVKKERKDSSIQ

>Oscar m.103051 Length = 491 compagen [Oscarella carmela]

SPRPAGSRFYVPARYVLAFMALLGFANVYALRVNLSVAMVKMKDEFGWDSSTRGIILGSFFYGYIVTQIPGGMLA
TRYGAKWVFGLGCLCTTVLTLVTPLAAYKSKWLLVAVRVLEGLGEGVTFPAMHAMWSKWAPPMERSKLATIGYTG

SYVGTVIAMPISGLLCDHGFTSHPHGSRWPSVFYVFGVVGLLWCVAWLCLVHSSPQSHPRISPEEREYIEMAIKE
GNKKSPVDPNAAIPFGPMMKSSAMWAIIISYFCNNWGFYTLLTDIPSFFKDVLGFDITQDGFLSAAPYAVMAAVV
PLTGWIADTLREKNILSTTKTRKLMNTPGQLLPALFLVITGYTTNKTIAVACLCMAVGFGGMAMSGFNVNHLDIA
PHYAGILMGITNAAGTLPGFIGPYVAGAMTPAHPGSHTLQLEWRNVFYLAAEIYAFGGLAFLILSSGELQWWA

>Thelohanellus kitauei JWZT01000915 Cnidaria Myxozoa

MAKERESNKTQTSSCPVRHNKTTTLIQIRTEKFEWSESMQNYVLLAYNVGYMIGHVPGAFLSITFGYRRVMIFCL
AASTILTIASLFAAPHQWVFFAIRSMIGLVNGPLYPIIHETIAGHSPPSERTFLTMFTHTGNLVSLAIILPIGGL
FIDNFVNGWKYVFILSGALGVLATIMWTLFVYSEPEENPWMSESEQKYITGQIYPLGKPPKKTMKNVPFCAIFTS
PHLYIFTIAHFAKMFVLYMNLFGIVKYLYKFFTLSPVMAGNLAVIPFLADFIFQTFYPKFVNSMKKSGMKITVIR
KLNTFIGSFGCSSFLIASSFISCTNLVGGIILQSLSLMFLAPFQAGYFTAIIEHAPQYSGVTFSFINIGGTFSGV
VQRFLVAKLSERIPDMRVAYKYSFIITGIVSFVLSMVYVVFGTADLQPWAQTGNKKAEMNKKEEVKNGKDLEVEA

>Hydra2 ACZU01055588.1 [Hydra vulgaris]
CVRTSMSICISALSDELHWDKKTEGFVLTSYFAGYLLTNIIGGYLADFKGGERVIFYSTFVWSFFTILIPALVNS
VVKFGFKTVIVCRFIIGLSQGMFFPSLSAILAKSVSIEDRSFIFSCTFSGSSIGTVITGLFGTILLEQVHWSSVF
YFFGIASFLWAFLLKCLHWSKKKNPLDTDNIRGIKKIVPIKVLLFNKSVLAMLFAYFSMSVSFYVFLSWSPTYFH
ETYPKGKAWLFNVLPYLASFLLSLGSGKVANMLIVCGFSVTFVRKLFGSLLFLGMCFFSLLLAATHSFETTLFVM
TLNIGMNAFAASSVAINPQDLSPEQAGVLYGVANTCGALGGFLGVLISGIILSNSGEWFRVFIMTSVISFLGFLV
YIMYGSGK

>Hydra1 XP_012556561.1 PREDICTED: sialin-like [Hydra vulgaris]

MKFKAKMMHRGSVKQKSSANEDKHVEACNTEGDRDVFTNSSEKTYEKEKHFESCQNVVNENLPLDRRKFSKSSVP
NPENNYNFETVENINNDKNISSHTKYASDHNHINHSLTSLKSCNTTESDILHLKGKTNTARVKLQTSFNTTTSLP
FEQSIEEQDLRTQVGQCGCMSKRYVLAILSFFGFFNVYCLRVDLSVALVAMTNNHTRVMFNGTEYLVAPEFNWST
QLQGHILSAFYYGYLLTQIPGGYIAARFGGKNLFGIAILFSAVLTLMTPMASRSHWMWLFLLRLIEGLCEGCIYP
AMYALWSRWAPPMERAKLVTIPHSGSYAGSVAGTLIAGYLCELCGWAWVFYLFGLLALMWVAVWMIMISDSPEED
NHISTEEKNMILKSLEEDQTSHLLKGSLPWQKIITSMPFLAILVAHTCEGWGFGTMQTGLPKFLSEAMNFRISKT
GEYTATLYLVMGINVFLSGQLADFIRQRCYLSVTTTRKSFTVIGFLANAVAFIISMYMFSPEGAVIVIIVGAGIE
TLAWAGFGINHLDIAPRYASVLFGITNTCATVPNILSPILVGVLTDLGEAGSGGRQHWDRVFYISAAMFLFGALF
YGFFASGERQSWAADKSDESLEEEVIKEVIDQTEQNEITTQTKSK

>Acropora XP_015762709.1 PREDICTED: sialin-like [Acropora digitifera]
MLWEYLLFYIGISMAESETILKTSSSSYRSRRAAECCIPCKCRYVLAALSCSGFCVIYLLRVNLSVALVAMVNST
YTNEKVSTSNPECQRNLSTSSLQKDEQFNWDEKTQGLILGSFFYGYILTQLPGGWLGARFGAKNLFGFGVLTTSV
LTMLTPLAACHSVGTLIAVRILEGLGEGVTFPAMHAMWSSWAPPLERSKLITFSYAGLQLGTIIGMPLTGYLCSS
SFWGGWPSVFYIFGAIGVIWFFIWMTFTYDRPSDHPRISIKEREYIESTIGEGQDKRQRVCLAHLQPAVHNYIII
SFIVCYCYDCKKSLFASFISLYLFQDGFLSAIPFLCMYVFGISAGQVADWLRLNKILPTGEVRKVFATGGFLVPA
CLMVSTSYVGCEDTTLAVLLFSLALGTSNFNAASFNVNHLDIAPRYAGVLMGITNAAGTIPGIVGPFVVGVLTNN
EPTREQWQKVFYISAGVYVLGWLTFTLLASGKQQSWNTPYENLFVPIEIPREPRTPIMASVLENGGYTSCNSEEG
LATFDARANYRH

>Nematostella XP_001637577.1 predicted protein [Nematostella vectensis]

MNTEEDCLLRPDVRRSRPGTCWRVRYTLALLACLGFCVVYGLRVNLSVALVAMVNSTYDGIDGNTNSECPGGNTP
DTTYKNGDFNWDQKTQGLILGSFFYGYICTQLPGGWLASQFGGKHLFGFGVLCTSIFTLLTPWAAHQGIPMLIAL
RILEGLGEGVTFPAMHAIWSSWAPPLERSKLLTLSYAGCQLGNIIFMPLAGVLCASEIAGGWPSVFYIFGSLGIL
WFVVWTLMVTEKPADHPTISEAERDYIISSIGTSQDRKTTKQNTPWSAIWSSPAVWAIIIAHFCNNWGFYTFLTL
LPSYFKEVLNFSILTNGFLSAVPYSTQWLFIVIAGQIADWLLRRRISSVSDVRKVFCVTAFIIPACLLVATSYTG
CTTTTLSVCLFSTALGFTGFNLSGFNVNHLDIAPRYAGILMGITNSFGTVPGIAAPYVAGWITNKKPDRAHWQIV
FFISAGLYVIGAISFVTLSKGSERRWNIPEKDMIQESVRKL

>Exaiptasia1 KXJ15574.1 Sialin [Exaiptasia pallida]

MDEEEEVLVRRVSRSKCTLSCRHLLALLSSLGFCVVYALRVNLSVALVAMVNSTYENSNSNXQNRECPGGMDDES
SHTGGTFNWDQKTQGIILGSFFYGYIVTQIPGGWLASRIGGKHLFGLGVLCTSVFTLMTPWAAHQGVGTLVALRV
LEGLGEGVTFPAMHAVWSSWAPPLERSKLATFSYAGAQIGTIISNPVSGALCDSNFLGGWPSVFYIFGSLGILWF
IVWTVLVYDKPADHPRISKEEKDYIQTIGSQQDARKKGHSTPWFAIWTSPVVWAIIIGHFCNNWGFYTFLTSLPL
YFKEVLNFKILQNGILSAIPYAAQFTVIVAGGQIADWLRDHRIVTTTEVRKILTIGGLIAPACLLVATSYTGCND
RGLSVGLFSTALGLTGLGSSGYNINHLDIAPRYAGVLMGITNMFATIPGIVSPYVTGWITNDNTTRAQWQKVFYL
SAILYVVGGISYLILGTGKEQLWNTPEEDLYVQVEFSREERRPTVFNSDXDERTGTHPVVV

>Exaiptasia2 LJWW01000218.1 MFS1 [Exaiptasia pallida]

GRASMDEKQPLIIQPHRSKTSTSKCFVPCRYVLAILSFWGMSVTYSLRVNLSIALVAMVNSTFANANADLNKPEC
EGDGNTKTHNKVGTDKYMWMILAAFFYGYIVTQIPGGWLASRFGGKHLFGLGILCTSVLTLLTPWVAHRGVGAFV
ALRLLEGLGEGMAFPSINALWSSWAPPLERSKLVSITFSGSRIGTILALGISGFLCDSDVTGGWPSVFYLFGAQG
ILWFIVWTIFIHEKPAEHPRISIEEKQYILKSLGSTHDVQRKAHSAPWLSILTSRAVWAIFIVNFSTSWGFYIFL
TTLPMYFQQVLNIXIAKNGVVSAIPNALAYVFIMVSGVMADWLQKSXISTTAEVRKIFTLAGLLPPAFLLVANSY
AGCNDKTLSIIIFSIAVGLNGLTASGFLVNHLDIAPSQLVVNGKRFSILLLPSTSLVP

>Crassostrea XP_011446826.1 PREDICTED: sialin [Crassostrea gigas]
MSFESSSEDPMDYLKTKDSEKEPLLGGKVVAAPAGFGSRHVLAFWAFLGFFNVYCLRVNLSVALVAMVNSTNNDP
SSVNSTECKEDIPANTTSTNTGEFNWDSNTQGLVLGAFFYGYIITQIPGGWLAEVFGGKKLFGFGVLCTAILTLL
TPLAARWNLYVFIALRVIEGIGEGVTFPAMNAMWGKWAPLWERSKLLSFTYAGAQLGTVFSMPISGILCKSDFLG
GWPSVFYLFGVIGCVWFAVWMLVVHDTPAQHPRISQEEKDYIETSVGTRQKLKTPWLSIWTSPAVFAICAAHFAN
NWGFYTMLTCLPTYMKKILHFDVQQDGFLSALPYLVCWMCQTISGQLADYIRKNQYLTTANTRKVVNSCGLLLPA
ILLCCVQFAGCNHAAVVAIVTFAVGLGGFCMGGFNCNHIDIASNFSGTLMGITNMFATIPGFLGPAVVGWLTSHE
DTRAKWQIVFYISAAIYVTGCILFNVFARGEEQEWNIPEVSVLVYKGNKINERDRSESSQNSISSRIQSAQ

>Biomphalaria XP_013086318.1 sialin-like [Biomphalaria glabrata]
MARSTRTGSALSNSSVHSGSQDMDESDALLPSYAELERKKKDEEGSQCIAARHVLAIMAFLGFVNVYCLRVDLSV
ALVAMVNSSSSSSNSSKSTDCPAPGKDNSTSPAKVGEFDWDETTQGYILGAFFYGYIVTQVPGGWLASKFGGKNL
FGYGVLCTSVLTLITPVAARYSVYLLIAVRVLEGLGEGVTFPAMHAMWGSWAPVWERSKLAAFTYAGAQLGTVIS
MPVSGLLCDSDFGGGWPSAFYVFGSLGCVWFVAWMFLVHNTPAEHPRISTSEREYIESSVGKREHIKTPWLQILT
CPALWGISLAHFTNNWCYYTLLTCLPAYMSNILRFNLKENGLLSAMPYAVCFLTQNTSGVLADFLRGRGYLSTGN
ARKLFTSIGLLSPAIFLIVVNYVGCDHVLAVLFLTLSVGLQGCIMGGYNINHLDIAPKFAGVLMGITNSLGTIPG
FMGPAVVGYLTNNNQTRGQWQIVFYITAGMCFVGAIGYDLLAKGEEMHWSRLPHKEQEIIVPERTRTPVTRSGRP
VSIGSVNVNSSDTDLLVPPPENNL

>Aplysia4 XP_005101179.1 sialin-like isoform X1 [Aplysia californica]
MDMSSQPVVEISPLFCSQRLTLAMMVFLGNCVSYFARVNMSVAILCMVRSDVTVLNGSLSNVTNVASHTVDSCGG
PDQALTADLLRDDTGEFSWDKGTQSKLLAMYFYGYIFTQVPGGWLASRFGGRRVWGVCMFVSAVCTLLTPVAVRT
HIYIAYGLRFLLGFVSGVNFPCIQAIFGRWVPPLERSIMNAIASSGMLLGNIFTFSISGVLCKHGFDNGWGSIFY
LSGVMFLLWVVAWFPVTSDTPAQHKRVSAGERKYIEGSIGRSSVAKVSDVPWVSIIRSGPLWAIIVSHFCGNWLT
YTLLTSLPTFMKESLRFDVSQNGALSSAPYLTQLVTSLMSGVAGDKLRQRRVMSTTAVRKTFQLAAFWGSAVCIV
LAGQMTCDQRYWAVVLLCLCVGFMGLTISGFTVNHLDLAPAYAGILFGITNTMGTVPGMIAPLVAGAMTPNRSAE
EWRSVFYVCGAVAGVGGIIYVILADGELQEWAVPPDVSGNMEMTFPADEKEDDSLMEKSESYIQDGGECDVVIKS
GNEK

>Aplysia3 XP_012943082.1 PREDICTED: sialin-like [Aplysia californica]
MTKDSAVQTNHTNHNSNNKNPTYHHTYLHTNNTDPTYHHTYPHTNHTNHPEPTPPVMPTERTALLNNSDHVTLTP
RSKVKDERPADTGGFFSCRHILALMAFFGFVNVYALRVNLSVALVAMVNNTGTDKAGHNDTRDGECPAPGGANAT
DGGKSGEFDWDPTTQGYILGAFFYGYIVTQLPGGWLASRFGGKRLFGLGVLCTSVLTLLTPVAARTSEYLFIALR
ILEGIGEGVTFPAMYAIWGQWAPVWERSRLVAFTTAGAQFGTVISLPLSGLLCDSDILGGWPSVFYIFGAVACLW
CVFWMLIVHDTPAKHPRISVAEREYILTSLGDTRHISPPWKQIFLSPALWGIMGAHFASNWGFYTLLTSLPTYMA
DILKFDMKQNGLLSALPYAGSWIIGPVAGLVADIIRRRHILSTVCTRKVFNTLGCLIPATTLILVGYVGCNHVLV
VVCLTLSVSLASFNGGGFSVNHLDLSPRFAGILLGLTNTVATIPGFVSPALVGYLTDGNETRAQWQIVFYVAASI
YALGALIFALLARGVEQPWSKSHDTEFLVPEPEHPPAAGVSADNNDVLGGGDVKVKAVVAAGLDHSNAAGAPTVG
GYGSVHAVDAQTKSVNNVQSA

>Aplysia1 XP_012943348.1 [Aplysia californica]
MTKTTSQDNEKGTIGGEKVPILLSKRWILAYIGMVGFFFIYSLRVNLSVAIVCMVKDDVNKTTSTNLSTSNEATC
TADTPSNSTNNGEFDWDKDTQSLMLSAFFYGYIVLQVPGGWMAGRFGGKRVLGIGVLVTAIATVLLPVAARQDHR
AVYALRVIMGLAEGVSFPCMHSMWGRWSPTMERTRLASFTYAGLNVGNVVTYIVSGLLCAYGFDNGWAAIFYLTG
GAAFIWVFAWFVFVYDSPEDHPSISQAERDYIVSHRGDKQTKKFKVPWKSVVKSRAMWMCLGAHLCNNWMHYTLL
TGLPTFMKQVLKYDIKQNGVLSAVPYMAMIFTQIFAGHAADFLRRRFLSTTHTRRLFQSVSFLGSGACIVAVGFI
DCERRTIAVVLLTLAVALEGLCYAGYMVNQVDFAPRYAGVLFGITNFISTIPGTLAPIVTGKLTPNNYQSEWRTV
FYLCGAFTLIGTIIFGGFASGEIQPWAVGDEEDDLNMEMTVKPDSADAKMDNGGHNNLAFSKLDEKKEKSTD

>Aplysia2 XP_012942626.1 PREDICTED: sialin-like [Aplysia californica]

MALAGDEKDENNEGAEGPEIEATPLLASSRLGLAIIGFLGFINVYAVRVNLSVAIVCMVNQTAIRSLTAGDNGTD
SGSDECAAVSSDNGTSSDLAEMGSLEWDKTTQGTILGSFFWGYLFGQVPAGWFATKFGGKWVYGITMLIAAVATV
FTPLLAEAHYIALIIVRVILGAVTGMTFPAMHAIWGNWAPPLERTKLMTFTYAGAQVGIVIGFPLSGFLCKYGFA
GGWPSIFYVTGAFSLIWAVAWILLVSDSPSTHKRISDAEREYILSSLQSGSVTENKRDIRVPWLKIMTSLPVYAI
IIANVASDWGAYTLLTNIPTYISEVLKFDIAANGLVSALPYIAFWIVINLGGWVADFVRDRKLLTTGVTRKAFNA
FGKILPAIMLISLGYVDCSQPTVAIALLVLAVSLTGTQYSGFLVNHVDIAPAFAGILFGISNSIAAVTGFISPVI

VGVITEERQTRAEWQIVFYVAAAIYIFGAVFYIIFASGELQPWARVETSGTTRAANHELAVNSEGENLKMLQEGE
ATKV

>Lottia XP_009066082.1 Lottia gigantea

MLYFSENVFCSCINTRTVLAIMSFLGFVNVYCLRVNLSVALVAMINNTGLDNSNKTVTEECPGDVVNSTSPSTHP
GEFHWDQTTQGVILGSFFYGYITTQLPGGWLAEKVGGRRLFGYGILCTSLLTLLTPVVARWSLPGLIVLRVVEGI
GEGVTFPAMHAMWGSWAPLLERSKLVSISYSGAQLGTVISMPISGLLCDYGFAGGWPSVFYVFGALGCIWVFFWM
FIVYDHPSKHPRISKSERLYIESTTGKREMQNLFQNTTTPWKSIFTSGPLWGIAAGHFANNWGMYSMLTCLPTYM
KEILKFDIKANGILSALPYLALWLLQLISGFIADWLRGRGILNTNKTRKLMNTLGLLIPAVFMIGVSYSGCDHTL
AVVLLIFAVGTGGFSMAGYQVNHLDIAPRFAGSLMGITNMIATIPGFLAPLVVGILTDHQETSGQWRIFFFITAA
IYIVGAILFLALSKGEELAWAKTRELENLIEKSVQSNVLPYDSDRTNLLHNKDI

>Capitella teleta AMQN01003594.1

MGEETLWEYMTSCRVTLALLTSVGYAFVYILRVDLSVAIVCMVKDPFINSTLDNNSVSIDNYSDYSDGCGGQAGL
GSSPSPYKGEFEWNKEMRGIILGSFFYGYILTQVPGGWLTVRFGPKKVFGVAMVINVMATILTPIAARVSVYLVL
VLRVLLGLCQGVCFPASHHMWGRWAPPLERSKLVSFSYAGTIFGTVVALSLSGFLCSVEFAGGWPLIFYIYGGAC
AVWCVAWFYLMHDTPMKHPRITQKERNLITSEVGAAVYHKSTHVPWKHIFKSPATWAIVTAHVCNNWGSYTVLTS
IPMYMKEVLYFDMKSNGLLSAVPYMVMYVVSIFAGWFADFLRQRRILRTVTVRKMWQCISFWPPGVCIAAMGFMS
CEHRYVSVMLLSATIALTAFGRSAYSVNHVDIAPRYGGALFSLSNTVATFPGFISPAVVAALTPNGSQAEWKIVF
FSCGAVYAIGGVIYIIFARADVQSWATPSEIDDINIGEEELKEELKELRD

>Tribolium XP_015837357.1 sialin isoform X1 [Tribolium castaneum]
MTTLTELINTIRIIAIIAQPANQHTYLPKMTFEKKSPTCITSSYEVTLSTTTSDPKWMFWKKRRYVVAVLAFFGF
FNAYALRANLSIAIVAMTENKTTVLENGTTIQEPPEFDWDSKVQGYVLSSFFYGYITTQLLGGWLSAKIGGKRVF
GGGIAVTAFLTLITPILARINLSLLLTLRVIEGIFEGVTYPCIHAVWSRWAPPLERTRLATLAYAGSHIGTVVSM
PVSAYLATALGWPSIFYFFGSLGLIWFVIWWVVVAESPAEDSRISKEELEYIEQSLGNVDAKRNIVYPWKSIFTS
APVWAIVVAHFTDNWGFYTLLTQLPKFMKEVLNFPLNTSGILSAIPYLAMAITIQLSGHLADRLLEKKIFTTTQV
RKIFNCGAFIIHAGFMIGAAFCETAVSTITCLVLAVGLGVFSWSGFGVNYLDIAPQHASVIMGVSNTFGTLAGIF
SPIVTGYIVTTPSADEWQIVFFIASGLFVLGSIVYGIFASGEVQPWAFQCCEDDSTEKKTAAYENNYCGVED

>Aedes XP_019539846.1 PREDICTED: sialin-like [Aedes albopictus]

MDVTESSKLSDGISAPLWMFWKRRRYIVVFMAFFGFFNVYALRVNLSVAIVAMTEEREVVLANGTTVVEQYFDWT
TQMQGFVLSSFFYGYILTPFLGGFISNKLGGNYVFGVGIGTTAILTLLTPLAAKAGLGWMIGVRVIEGIFEGVTF
PCIHAVWSKWAPPSERSRMASIAFAGNYAGTVVAMPLSGIFANAYGWESVFYIFGVIGCVWFVLWTFMIKTSPEV
DRGISDKEKEFILSSLGRTEGVPEKIRHPWKAMLTSMAVWALVASHFSENWGFYTLLTQLPTFLKDTMHFQLEKT
GFISAIPYLVMGILLFVSGYLADLCQVKGWLTTTQVRRFFNCGAFLGQTVFMITGAFILKPGATITCFTIAVGMG
AFAWSGFAVNHLDLSPKSAGVLMGISNTFATIPGIVSPILTGYITSNKSDDEWRVVFYIAAGIYLIGCVIYWIWA
SGELQPWSIEAQEKLMKERNGEKNGFDGGYVNKMKIEDEK

>Drosophila1 XP_017125620.1 sialin [Drosophila elegans]

MAFMPCFYVPKRVNLAIMLFMACLLSYMMRVNLSINIIAMVEDTSSHENGTEVEALPNYGPRYNWTQSDQALLLG
AYFYGYMITSLPAGTLAEMLGARNVAGYSCLVAGILTALTPAAAAWDKYAVFAVRFLIGFLNGVVYPCCHSLVSK
WAPPDEKGKFVASLMGGTFGTVITWPLSGVIIENMGWDWAFYMVGIFVLIVVSIWFFLVTDTPAQHSTISLKERE
YIENSLGSTLSNKKKWPPYKELILSLPFWSLMLLHYGSMWGLFFLITATPKFLSEVLGFNLSSAGFLSSLPHVAR
LLCAFGFGAVADWIRRRGWLSVTRMRKAFCLPSHILPGVMLIILAYFGRDPYVCVAIMTISLGFNGAATASNLAN
SQDLAPNYAGTLYGIINCVGTTPGIFSPLIVAAFTKNENTIDQWHWIFIIGAAAYILPALFFWVFGSGKIQKWNE
VETTESREDIVNTKL

>Drosophila2 XP_017133157.1 sialin [Drosophila elegans]

MAQVEARTVLWYMTFMGFIVNYMIRINLNITIVDMIVGKGAISSNLTENSTLENSTKLDERFSLERWFLDWASIP
YDRDGFNWNEKQQGALLGSFFWAHWTLQIPGGILATKYGTKLIFGWSNGIGVFCCFLIPIVSYWSYTGLIVLRVF
QGWITGLAWPSMHVLTAKWIPPNERSKFVSAYLGSSVGVALFYPIFGYIIDWTRWEWVYYICGIVGTLWFIAWQF
LVFDTPAQHPRIADSERKYIEKSLGASVQSAKGPTPWKAIATSRPVWLNVVAQWGGIWGLFTLMTHAPTYFSKIH
HWNIRATGFLSGLPHLMRMIFAYVFSTFADYLLRTDRLSRTNVRKLATFICCGVKGLLILALAYFGYNATAAIML
VTVATMFHGAVSSGPLASMVDLSPNYAGIVLGVSGMIGGMPGFISPLIVGQLTYQNQTIEAWKNVFLLSSFMLTG
SGILYVLFSESTLQPWNSGCHQLPESGLKELQNLGNNKEDEEEEKKPLKADQDDKYTETNSVAERETKSKSDGDE
K

>Drosophila3 XP_017132353.1 sialin [Drosophila elegans]

MDDAKEQPQWGIKLSKFFVVPQRWILAIMGFFAIFNAYTMRVCLSQAITVLVVKRNHTGSAHSDAICEPDEDDGA
PVSREGGDYEWSEEKQGLILASFYIGYIVTHLPGGVLADKFGGKWTLSIGIFLTAAFTLITPVCIVYGGADALIV

LRVLMGLGEGTTFPALSVLLAAWVPATERGMLGALVLGGGQVGSIAGNLLSGLILDSMDWPWVFYIFGIIALVWF
TVFSLICYSYPYKHPFIKPSEREFLKQEIPPKDTNQPKTPWLAIFANIPMWALISAQIGHDWGFYIMVSYLPKYM
SDVLRFSIKANGLYSSLPYVTMWIMSLLSGCVADQMIKRNFMSTTNTRKIMTGLAAFGPAIFMVGASYAGCNRLL
VVALFTICMGLMGTYYAGMKLTPLDLSPNYAGTLMAITNGIGAITGVISPYLVGVMTPNATMMEWRIVFWVAFAV
LFITAIVYCIWASGEVQPFNDGTNSIKKKDQTS

>Drosophila4 XP_017116975.1 sialin [Drosophila elegans]

MSNTEKGGLHRVRNFLSCRQVLNLLTMLGFMLNYALRVNLTIAIVDMVRPNVTSAGNATVSGNDTLSGNSTAGNG
SMSSASPDGVDVYEERFPWDSYQTNFVLGCFFWGYILTELPGGRLAELIGGRRVFGHSMLWASLLTLITPLAAHI
NYVVLIVVRVVLGFMLGASWPAIHPVAAVWIPPMERSKFMSNMMASSLGAAITMPICGYLISVAGWASVFYLTGA
VGLLWSLAWFTFVYETPATHPRISAEERREIEEAIGTTTSKKRPSHVPWGQLLCSPAVWAIIICHGLAVFGFFTV
VNQLPTFMSKILHFDIKQNGLFSSLPYLGKYVMAVASSYLADYLRKKGTLSTTATRKLFTTFALVIPGLLMIVQV
FLGYDATWSVTIFSLALFAHGAVTAGYLGNGLDIAPNFGGTIFGLANTLSSFGGFLSTWMVGALTFKDDSFHSWQ
IVFWILGATYISGAVVFAILGSGELQPWNNPPERVKISDVTQEEGVPLKNGK

>Daphnia KZS04440.1 Major facil transporter 17 [Daphnia magna]
MVQQSSLLVNPVEPNELDEKLRLIPQDDEDQGCWGTRHTLVLLGFLGFANVYAMRVNLSVTIVAMVNQSAIPHIN
QTAVDICPVTPGSDQSGLVKDGEFAWDEYKQGIILGSFFWGYVLTQIPGGRLAELFGGRKLLGYGILSTSVFTLL
TPFAARASDTLLIFCRVLMGLGEGVTFPAMYAMLAEWAPPWERSKMAAFAFTGAQFGTVITLPLSGILCEHGFDG
GWPTVFYVFGTLGIVWFAVWMPLTADSPSRHKNISKEEKEYICSSLRDSIKRKSAPVPWRCMFRSKACWAVGAAN
VGHAWGFYTLLTELPSYMDNILHFNMKQNSFVSALPYLAMWLFSLLCSTIADLLISKQIFRVITVRKIFNSIGQY
GPALALVGAGFIGCNTVAAVILLTLAVGLSGASYSAFQVNFVEIAPPYAGTLFGVTNAVANLCGFMAPYAVGVLV
KGNQTLGQWRLVFLIAAGVYCVSNTIFLIFGSSQVQPWAECEDLGAEESIHVEENEESNDFLSRPFPYIQPDTLF

>Caenorhabditis NP_499065.1 Unchar slc-17.2 [Caenorhabditis elegans]
MEGATTKPRLVPSTRFALSLVMFFGCLVTYMMRTNMSFAVVCMVNENKTDTGVEKVSRCGKEMTPVESNSSVIGE
FDWDKQTTGMVLSSFFYGYIGSQIIGGHLASRYGGKRVVFVTILGSALLTLLNPVAARTSEYALAILRAAIGFLQ
GATFPAMHTMWSVWGPPLELSVLTGVTYAGAQIGNVIVLPLSGFLCEYGFDGGWPSIFYIIGVFGVLWTAVWWYV
SSDKPATHPRITPEEKQYIVTAVEASMGKDTGKVPSTPWIKILTSPAVWACWAGHFAGDWGAYTMLVSLPSFLKD
VLGLNLSSLGAVASIPYIAYFLAINAGGVLADTLRSKGILSTLNTRRAAMLVALIGQGIFLVASGYCGCGQDVLV
IIFITCGMAISGLQYAGFVVNYLEIAPPFSGTVMGTGNTISALAGIISPAVSSYLTPNGTQEEWQMVLWLTAGIL
TIGALLFSIFASGEVQPWAKLTAEEGHEMAPLREGEKIELATA

>Mycobacterium ANZ82570.1 NanT [Mycobacterium tuberculosis] 10 DTM

MAAPRLTGDQRNAFMASFLGWTMDAFDYFLVVLVYADIATTFHHTKTDVAFLTTATLAMRPVGALLFGLWADRVG
RRVPLMVDVSFYSVIGFLCAFAPNFTVLVILRLLYGIGMGGEWGLGAALSMEKVPAERRGVFSGLLQEGYAFGYL
LASVAALVVMNWLGLSWRWLFGLSIIPALISLIIRYRVKESEVWEAAQDRMRLTKTRIRDVLGNPAIVRRFVYLV
LLMTAFNWMSHGTQDVYPTFLTATTDHGAGLSSLTARWIVVIYNIGAIIGGLAFGTLSQRFSRRYTIVFCAALGL
PIVPLFAYSRTAAMLCLGSFLMQVFVQGAWGVIPAHLTEMSPDAIRGVYPGVTYQLGNLLAAFNLPIQERLAESH
GYPFALAATIVPVLLVVAVLTAIGKDATGIRFGTTETAFLVRHRNRH

>Klebsiella OUE88183.1 nanT [Klebsiella aerogenes] 12 DTM

MSTTTQNIPWYRHLNRAQWRAFSAAWLGYLLDGFDFVLIALVLTEVQGEFGLTTVQAASLISAAFISRWFGGLML
GAMGDRYGRRLAMVTSIVLFSLGTLACGFAPGYTSLFIARLVIGMGMAGEYGSSATYVIESWPKHLRNKASGFLI
SGFSVGAVVAAQVYSLVVPVWGWRALFFIGIVPIVFALWLRKNIPEAEDWKEKHEGKAPVRTMVDILYRGQHRVL
NIIMTFVAGASLWYCFAGEARIAGLVALLGLVCAIIFISFMVQSSGKRWPTGVMLMVVVLFAFLYSWPIQALLPT
YLKTELAYDPHTVANVLFFSGFGAAVGCCVGGFLGDWLGTRKAYVCSLLASQLLIVPVFAIGGTNVWVLGLLLFF
QQMLGQGISGILPKLIGGYFETDQRAAGLGFTYNVGALGGALAPILGALIAQRLDLGTALGSLSFSLTFVVILLI
GLDMPTRVQRWIRPEAMRTHDAIDGKPFSGAVPFSRGKSTLAKSKN

>Escherichia NP_417691.4 sialic acid transporter [Escherichia coli str. K-12 substr. MG1655] 12 DTM

MSTTTQNIPWYRHLNRAQWRAFSAAWLGYLLDGFDFVLIALVLTEVQGEFGLTTVQAASLISAAFISRWFGGLML
GAMGDRYGRRLAMVTSIVLFSAGTLACGFAPGYITMFIARLVIGMGMAGEYGSSATYVIESWPKHLRNKASGFLI
SGFSVGAVVAAQVYSLVVPVWGWRALFFIGILPIIFALWLRKNIPEAEDWKEKHAGKAPVRTMVDILYRGEHRIA
NIVMTLAAATALWFCFAGNLQNAAIVAVLGLLCAAIFISFMVQSAGKRWPTGVMLMVVVLFAFLYSWPIQALLPT
YLKTDLAYNPHTVANVLFFSGFGAAVGCCVGGFLGDWLGTRKAYVCSLLASQLLIIPVFAIGGANVWVLGLLLFF
QQMLGQGIAGILPKLIGGYFDTDQRAAGLGFTYNVGALGGALAPIIGALIAQRLDLGTALASLSFSLTFVVILLI
GLDMPSRVQRWLRPEALRTHDAIDGKPFSGAVPFGSAKNDLVKTKS

>Fonsecaea XP_013281077.1 [Fonsecaea pedrosoi CBS 271.37] Asco 11 DTM

MAHIESPTKETVPAARHYSDEYEIDNAHMSPGKYAATRLSTLKPPMAKAPNPFKLLAMLNTQQWLFFLVAFFGWS
WDAFDFFTVSLTVSDLAETFNKSNTDITWGITLVLMFRSVGSTIFGIAADRYGRKWPFIINNILFIALELGTGFT
QTYGQFLAVRALFGIAMGGLYGNAAATALEDCPEEARGIISGMLQQGYAFGYLLATAFARALVNTTSHGWRPLFW
FGACPPVLIILFRLCLPETNTFRARQALRAQQGSLAGTFISEGRVALKKHWLLLIYMVLLMAGFNFMSHGSQDLY
PTMLTNQFKFSADAVTVTQVVANLGAMTGGTTIGYCSQIFGRRFSIIFISIVGGALLYPYTFVTSKAVIAAAFFE
QFCVQGAWGVIPIHLMELSPGSLRTFVVGTSYQLGNLASSASSTIESTIGERYPLEPLINKEGKAVKRYNYGKVI
CIFMGAVYAYTILLTFVGPERRGRSLNVEHDEDMAEVTHTDLSHQIPVRGDSSEDENVIPGEKV

>Amphimedon_sv2 synaptic vesicle glycoprotein 2B-like Amphimedon queenslandica] XP_019851519.1

MGVANVNKLKMAFSKENNGQWSPLDSEKEAVEMKEIKMESFTPLNPFPEEEDEDSDGPEVGTEPDKTFLIKMATT
VDTTGEEADEAQKEREGIYTDYEEVLRLVGFGKAQILIMLGVGLVLASDSVEVLGIGYILQYLRLETEFGIASWQ
VALLSSNTFVGMLIGGYIWGGLSDIGGRRTVLIMSLFFNSFFAFVSAMSPNYYFLLATRFLSGVGVGGSLPVFGP
YLSEFAGSKYRGSYLNFMSTSWITGAVAVTGVAWALLPQEDIGLTVNGTSVHSWRVFLFLCAVPAFVGGVLCILM
PEGPRYLIEVRKEKRAIRILRNMYYLNNPLKYFNKIDTFPVKGIKLHLSVQSDSSPSIKYLLCNCHKNFLEILKR
TLKLFHKNLVRSTILLMFINFGLSFGSYGVTLWFPSYVDQLTNNGLTTPTSEWCNSTLSSFTLDDNSNKGSSFLC
SCPLTSYSNVTIGISSGDTWRAWQSSATNMTISGGHYKWVWLDTVRWDTVHITNVFINNLLMKNSTWISVRLNNV
FINNTYVCETKFINVSSDNDSTAEELFNESLCQDLLLLPGPFCPAGEGEVMAPKDYDAEYSHDLIITASGYVGNL
VSAIAVFFFIRSYWLAFSLFISTISVFMLYFIRTETGAVVLLCVFKAITIPAWSSTTVLVADLFPTALRSTAVGV
FLLLARIGAIIGTYTFGLFTSTSQVSVPVLLTAAILLGSAILSLMLPWTTRKTSLK

>Orbicella XP_020601787.1 solute carrier family 22 member 15-like [Orbicella faveolata]

MASTSISQRIPKRMDFDSALEHIGGFGRHQLRIYFVVSLISLPLAAQMLIVVFVGAVPEWKCPSPEGDILHCNSS
HARCCVKDGSICPGAEFSTEFTSIATEWNLACTHRYKCELSQSIFVAGYMFGVLIFGILSDKYGRRKPWLFAYIV
GGVLALMSAFVGTYEEYIALRFAMGVMNGGGGLITYVLSTESIGPSYRGSAGTWQQAFYAVGYVVLALEAYFIRD
WRTLTVITILPSFIILLVFKSIPESPRWLASQGRIKEAENILRNIEKENGSNKNEVIFLKTDTTKEDTKTSQYGV
IDLFTHKSVCIVTVIMMFTWCVNSMVYYGLALNVKNLGGSVYINFALASVIELPSYLVTQVLLARLGRRQSLFFF
LLSASVSCFLCMFLQGGQNAAAVSITALVGRFCISASFALLYVYSAELFPTVVRNAGMGISSLSARLGGIVAPFI
VLLGDQRNSLPMFVFACTALLAALVGLKLRETQGRPMPETFAELEENHPRKPKTNGDPY

>Drosophila XP_017121721.1 Major Facilitator Superfamily (MFS) [Drosophila elegans] 9 DTM

MPMTLTASQPDLSSPTAQLLTPQERATARHVVAASFLAWMLDACDFFLVLFTLDNVAHSFNTSLESVLLAPTLTL
MTRPIGAYLCGRAADTYGRKPVMIITILVYSVIEVLSAFSPTLWVFLVLRTLFGIALGGEWGVGTSLIMESVPAS
WRGTASGILQAGYPAGYLLASILFLTLPVVGWRGLFILGGSAVFSALYIWLRVPESPDWLARQKASASPTQKHQG
LWPIVRQNAALCVFAITLMTGFNFMSHGSQDLYPKVFLALERHIPPPSITLIVVLYNVAAIAGGLFFGVLSQRIG
RQYAIALAALLTIPLLPLWTLPQSAFWLAVGAMCIQFCIQGAWGVVPAYLSELSPASIRATFPGLAYQCGNLLAA
SNALLQTEIGRVLGRGLAPALMLTVGLAALAVLVLTLLNAKLHPQRAS

>Homo_sv2 EAW97840.1 SV2 related protein homolog [Homo sapiens]

MVEDAVEAIGFGKFQWKLSVLTGLAWMADAMEMMILSILAPQLHCEWRLPSWQVALLTSVVFVGMMSSSTLWGNI
SDQYGRKTGLKISVLWTLYYGILSAFAPVYSWILVLRGLVGFGIGGVPQSVTLYAEFLPMKARAKCILLIEVFWA
IGTVFEVVLAVFVMPSLGWRWLLILSAVPLLLFAVLCFWLPESARYDVLSGNQEKAIATLKRIATENGAPMPLGK
LIISRQEDRGKMRDLFTPHFRWTTLLLWFIWFSNAFSYYGLVLLTTELFQAGDVCGISSRKKAVEAKCSLACEYL
SEEDYMDLLWTTLSEFPGVLVTLWIIDRLGRKKTMALCFVIFSFCSLLLFICVGRNVLTLLLFIARAFISGGFQA
AYVYTPEVYPTATRALGLGTCSGMARVGALITPFIAQVMLESSVYLTLAVYSGCCLLAALASCFLPIETKGRGLQ
ESSHREWGQEMVGRGMHGAGVTRSNSGSQE

>Strongylocentrotus_sv2 XP_011673012.1 synaptic vesicle 2-related protein isoform X1 [S. purpuratus]

MASLFDESTFTDTDVIIKDGDDVIFKEETLAEVEPQPYLNTSEDSNGTGTKDHEYTVQEAVDAMGFGWFQVKISF
IVGFNWMADAFEIMLLSVLSDKLRCEWDLYPYQQALLTTFVFTGYFIGAPLWGMMGDKFGRKKTLALCSFHIFYF
GFLSSFSPNLIWLLILRGLLGASLGGTSQSVIICAEFLPSKSRGLCLVCLEAFWVIGVCLEITLAMVVMPTLGWR
YLLIFSSFPLVIFVVLVTFLPESASYQQASGNWSGAMATLEDISRTNKKPLPPGKLKRNAELKPKGSIRELFSTK
LLAMTTVILINLWFCNAFLYYGNVLLSAELFSSGVTSCVSTGNNSTTELECFSACKSLDTQGYVGLLVSSLGEIP
GILLTLFMIDTAGRKLTMGLEMLVCAVFSFLLLMCVDGIPQMIFIFVIRGMISGAFQALFVYTPEVFPTNVRSVG
LGWCVAFSKLGSIVTPFVAQVLIKQSVFMTFCVYGGCAVFASLLAFILPTETKGRTLK

>Daphnia_sv2 KZS14128.1 Synaptic vesicle 2-related protein [Daphnia magna]

MLGKLNQRLKRKSESGQYNDLDGESSITNDGRSTSPFSNSFGFLGGDSGNGGLLGSAIEMSAAGAGVVPDDTFTV
DQAVNATGFGRFQVKLSLITGLSWMADSMEMMILSIISPALKCDWRLPDWKQALITTGVFLGMMMSAVFWGQLSD
KYGRKKALALSAVLLSIFGLLSSFSPTFFWILLLRLLVGFAIGCTPQSVTLYAEFLPVQQRGKCVVLLDSFWALG
ACFEVLLALIIMPTLGWRWLLALSTIPVFVFTLVCAWLPESARFLAANGRTEEALAVLRRIAEENGKPMLAGRLI
VDDLTSNDSGTSGSAVSPSVPGNGDMNSLVSLSSDLRWKSPLQRLLSPELKLTSLLLWFIWLACAFCYYGMVLMS
TELLAGAGLAEEEGDCLNRNRGGNSTSHEDCSAGCRVLTSADYTDLLWTTLAEFPGIVVTLVVIEFLGRKKTMAL
EFFIFSLTVFLIMIVCISNRSMLTFMLFLARGIISGVFQAAYVYTPEVYPTYLRSVGIGVCSGMARLGAMVTPFV
AQVLVQDSLNVAIGLYGSVSLLAMVASLCLPIETKGRAMS

>Acyrthosiphon XP_008189196.1 [Acyrthosiphon pisum]

MPILLSLAILTKSEAGILGSASLIGSALGGWSAGMLADRLGRIRVMQLTVLWVACFTALTALCGDFWQFLIVRFL
QGLGYGGEVVVGGVLISEVIRASYRGRVGASIQSGYAIGYAISLAVLPMLLNFFPQQIAWKLFFLAGIVPAILVW
FIRRLVPESPVFLRKKVEENKSSVSDIFKGKNLRITITATLLASGIFGGAYVMITWLPTYLRMELGLPVLSMSGY
LAINILGSLIGPFLYGRLSDRFGRWKTIILFLMGQIAVVSVYMFADISLNITLVLGFFLGSLQGGLASGLTPAFS
ELYSTYIRGSGAGFCASFGRGFGSLMPAIVGIAMQSE

>Hebeloma KIM37555.1 [Hebeloma cylindrosporum h7] basidio 10 DTM

MAPYFFRNLLPKRETRQHARPLLTVLGELTFTQWALYFSGWLAWTCDAIDFFSVSLSVTNLQTQFNRSTHDITTA
ITLTLLFRSVGAVVFGILSDRFGRKWPLVWNLVLDAILELGAGFVQTFPQFLALRSLFGIGMGGIWGLAASTALE
NLPVEARGLASGFLQEGYAVGYLLAAVINLFLVPKAKAGWRALFWTASGISLFAAGIRVLLPESEVFLRAKAAEV
EKGHTTTQKTKIFMKETKDMLKKHWLLCIYAVLLMTGFNFLSHGSQDLYPTYLTVSKGISTHNATVATIIGNCGA
IAGGIFAGWLSQYIGRRLTIIIHVALIGAFIPLWILPSTFSGLAAGAFCIQFGVQGAWGVIPIQLAEMSPPAFRA
TFPGVAYQLGNMVSSASAQIEATAGEHLRTTIIENGVPKNVPDYATVQGILIGVVAAFVVIVTIIGPENHGSHFE
KAKAAFEEGAAVEGPVVDDSGERVLRNRSSDDDDDGSGNEKGGVLSDEKA

>Calocera KZT62269.1 MFS general substrate transporter [Calocera cornea HHB12733] basidio 10 DTM

MDNEKAAMARAENGAGTQQPSGFAQWCADLKPKPTNASNDITASLAALRSVTLLQWAMFFSGFIAWTIDAIDFFS
VSLSVTALTKQFNRSTNDITTSITLTLLFRSVGALVFGMLSDRYGRKWPLIANLILCTIFSLCTAFALNYGAFLG
VRCLFGIAMGGIWGLSAATALENLPVQVRGLASGVLQEGYAVGYLIAAVINLTLVSYDPFGWRALFWVAAGISAF
AAVVRLCLPESQVFLRAKEDKRIKAAEGHEVHSKSKVFLRETRAMLRQHWLRCIYAVLLMTGFNFLSHGSQDLYP
TYLQQTKGFTAHNANIATIIGNCGAVAGGIFAGILSQFIGRRLTIVIFTLSIGAFIPLWILPSGFSPLAAGAFCV
QFGVQGAWGVIPIHLAEISPPAFRATFPGVAYQLGNMVSSASAQIEATGGEHQRTILNGQDAPDYAKVQGIFLGV
VAAFVIFMTLIGAENHSSEFEKHKLGFELGGGQDEAVDESATAGSVTPTRGEGSAEKAAQAQPSAVHRESV

>Gymnopus KIK65072.1 putative sialic acid transport [Gymnopus luxurians FD-317 M1] basidio 9DTM

MASTALSQLWHNATHNLVPRREKKVGQKPLITALLELNLRQWAEFWTGWLAWSCDAIDFFSVSLSVTNLSKQFGR
DTTAITQAITLTLLLRSVGAVLFGIISDRFGRKYPLVLNLLVVSVLELGAGFVQTYSQFLAVRSLFGIGMGGIWG
LAASTALENLPVETRGLASGFLQQGYAVGYLIAAVINLFLVPEQSHSWRALFWTAAGISAFAAFIRLCLPESKIF
LKAREDAKLREQERLGYRAGGAEKTKIFVRETGKMLRHHWKLCIYACLLMTGFNFFSHGSQDLFPTYIQTTKGFS
AHDATVMTIIGNCGAIAGGTIAGFVSQYIGRRLTIITFTLIAAAFIPLWILPNNFAGLSAGAFCIQFGVQGAWGV
VPIQLAELSPPAFRATFPGVAYQVGNMVSSASAQIEATGGDHQKTTISVPVTSDNPTGMKVVPDYATVQGIFIGC
IAAFLLVVTLFGPEKHGYEFEKHRAAFEEGGGDDEALMSEEVLQNRREAGAAGEKSSEDRSSPDAASEDEKGREM
AESA

6 - NANS: N-acetylneuraminate synthase

>AAD45660.1_NeuB_Aeromonas caviae Gammaproteobacteria

MDKIIKPFITINGRKIGPDYPPYIIAELSANHNGDINRAFAIMEEAKKAGADAIKLQTYTADTITFECDSEEFQI
HGGLWDGKNLYQLYKEAQMPWEWHQPLFEKAKELGITIFSSPFDFTAVDLLEDLDAPAYKIASFELIDLPLIKRV
AQTGKPMIMSTGMANAEEIAEAIATAQSNGCQELVVLHCVSGYPAPADQYNLRTIADMAERFGVLSGLSDHTIDN
ATAVAAVALGACLIEKHVTMDRNGGGADDSFSLEPHELAALCKDAKTAWSALGSVNYTRTEAEKGNVKFRRSLYV
IRDIKAGDVLSNDNVRSIRPGFGLAPKYLEQVLGKTAIVDIKRGTPLSFEFI

>AAC43302.1_neuB_Escherichia coli RS218] Gammaproteobacteria

MSNIYIVAEIGCNHNGSVDIAREMILKAKEAGVNAVKFQTFKADKLISAIAPKAEYQIKNTGELESQLEMTKKLE
MKYDDYLHLMEYAVSLNLDVFSTPFDEDSIDFLASLKQKIWKIPSGELLNLPYLEKIAKLPIPDKKIIISTGMAT
IDEIKQSVSIFINNKVPVGNITILHCNTEYPTPFEDVNLNAINDLKKHFPKNNIGFSDHSSGFYAAIAAVPYGIT
FIEKHFTLDKSMSGPDHLASIEPDELKHLCIGVRCVEKSLGSNSKVVTASERKNKIVARKSIIAKTEIKKGEVFS
EKNITTKRPGNGISPMEWYNLLGKIAEQDFIPDELIIHSEFKNQGE

>ABD95628.1_NeuB_Streptococcus agalactiae] Firmicute

MVYIIAEIGCNHNGDINLAKKMVDVAVSCGVDAVKFQTFKAEKLISKFAPKAEYQKATTGTADSQLEMTKRLELS
FEEYLEMRDYAISKGVETFSTPFDEESLEFLISTDMPIYKIPSGEITNLPYLEKIGKQQKKVILSTGMAVMEEIH
QAVNILRQNGTTDISILHCTTEYPTPYPSLNLNVIHTLKDEFKDLTIGYSDHSIGSEVPIAAAAMGAEVIEKHFT
LDTNMEGPDHKASATPDILAALVKGVRIVEQALGRFEKIPDPVEEKNKIVARKSVVAXKPIKKGDIYSIENITVK
RPGNGISPMNWYDILGQEAQDDFEEDEVIRDSRFENQLPEL

>KMQ51676.1_Chitinispirillum alkaliphilum Fibrobacteres

MGVISLGSKLVGAGHPVFVIAEVGINHQGDIDIAKKLIKEAKNCGADAVKFQKRSISRILTREGLEMPYENSNSF
GKTYGEHKRALELDETDYVELKRFCAQNEILFCASGWDEESIDFLDSIDVPFFKMASADLTNLPLLEHTAKKDKP
VILSTGMACLEDVRNAYVTVGKHNDKIAILQCTSTYPSKFSEINLNVLHTFKKEFPDAVIGFSGHELGIAIPPVA
VGLGACIIEKHFTLDRTMKGGDHAASLEPQGFAKMVRDIHHVTEAMGSFEKGIQDSEYPIFKKLTKSIVSACELC
KDDVITADMLTTKGPGTGISPTRMEAVIGKRVAKLIPADSVIKEEDILW

>ABS75841.1_SpsE_Bacillus_velezensis FZB42

MASCQIGTKSIGEGSPVFIIAEAGINHDGKLSQAFELIDAASEAGADAVKFQMFQADKMYQKDPGLYKTAAGKNV
SIFSLVQSMEMPAEWIEPLLEYCKKKGVLFLSTVCDEGSADLLYATSPSAFKIASYELNHLPLLKYVAGLKTPII
FSTAGADISDIQEAYQTITGEGNTEIAIMHCVAKYPAPPEYSNLSVIPMLAAAFPDAVIGFSDHSAHPYDVPCAA
VRLGAKLIEKHFTIDKNLPGADHSFALNPDELKEMVAAIRSVEAELRQGHHAPVPEVLSGSSHKTTTPIEGDIRR
FAYRGIFSTAPIQKGECFTAENLAVLRPGQKPQGLHPRYFELLTDGARAVRDIPADTGITWDDLLTKGSPYDD

>WP_027268045.1_Legionella pneumophila gammaproteobacteria

MRRVLNLGHREISDSSSCYVIAEIGHNHQGSLEKCKDLIQAAVEAGADAVKLQKRNNRNLFTREMYNSPYNSENA
FGSTYGEHREFLEFGKLEYLELKTFCHNLRVDFFSTAFDFESADFLEELDIPYFKIASGDLRNLPLIEYVARFGK
PIFISTGGADFEDVQRVYDRIMPINSNICIMQCTSGYPCEYNELNLRVIETYRKEFPDVVVGFSSHDSGIASPVA
GYVLGARVIEKHFTLNRTWKGTDHAFSLGPQGFRKMVRDIRRVEQALGDGIKRCYQSEENPHYKMAKKIVAGRNL
PIGHIISFEDLKFKSPADGVPPYRVNEFLGKKTLVALNEDDSVNFDVVEI

>WP_022662582.1_Desulfovibrio longus] Deltaproteobacteria

MRSLKLNGTIVNDENEAYVIAEIGNNHQGDVEICKQMFDAAKECGANAVKLQKRDNRSMFTKAAFNQVYNSENAF
GRTYGEHREFLEFGEREYAVLKEYASEIGIDFFATAFDLNSADFLASFDMPFYKIASGDLTNTPLLKHVAKIGKP
VILSTGGGTMADVRRAYEAIMPINDQLAILQCTAAYPCQPEDMNLRVIETYMREFPDVVIGLSDHQNGIAMAMVA
YTMGARIFEKHFTLDRTWKGTDHAFSLTSKGLSNLVRDLHRARTSLGNGVKQPLDLEVSPIKKMGKKIVAARDLP
AGTVLTFEDLAFKCPADGMPPYELDNVLGKTLTAELPEDGDLSFDILK

>OGP61643.1_Deltaproteobacteria bacterium RBG_13_47_9]

MKRQIAINGRVINDETDCYIIAEIGHNHQGDLQKAKELFNVAKACGAEAVKLQKRNNRKLFTKELFNKPYENPNS
FGETYGEHREYLEFGKKEYEELKQYAKEIEIDFFATAFDFASADFLAELDMPAFKIASGDLNNIPLLKYVAKIGK
PMIVSTGGATMENVKRAYDAIMPLNENLCILQCTAAYPAEHDILNLNVISTYRKAFPKNVIGLSDHENGIAMAIA

AFVLGARIFEKHFTLNHTWKGTDHAFSLEPVGFRKLVRDLKRLPKALGNGIKTVYDIEKEPIKKMGKSIVASRNL
KKGMTVFLKDLAFKSPGGGLPPYEFEKIIGKKLKVSMKEDQLFSLDIVKE

>OGP71522.1_Deltaproteobacteria bacterium RBG_13_58_19]

MPELVIDGRLINPEGDCYVIAEVGHNHQGDLETARQLFRAAQEAGAEAVKLQKRDNRSLYTRAMYTQPYDHENSF
GATYGEHREALEFGESQYRELQHYARELGITLLATAFDFPSVDFLAKLEMPAYKIASGDLTNIPLLKYAAQVGKP
LIVSTGGGAMEDVQRAYDAVMPINPQLCLLQCTASYPVDPGDMNLRVISTYRRLFPDLIIGLSDHQNGIAMAVAA
YVLGARVVEKHFTLNRAMKGTDHAFSLEPAGLRKLVRDLRRARAALGDSLKRPLPCEAKPLFKMGKKLVAARDLP
AGHVLTLQDVAIKSPNDGLPPYEFDRVMGQVLVRPLQEDENIAFADLRAASG

>OUV47462.1_Flavobacteriaceae_bacterium Bacteroidetes

MSVIELDKKKVGENHPTYIIAEIGINHQGDLDTAKELIQKASEAGANAVKFQKRSIQRILTQEGLNAPYENPNSF
GKTYGEHKKALELSKSDYAELFACANTHNVSFIASGWDEESVDFLDHLGVSFFKMASADLTNFPLLEHTAKKNKP
IILSTGMSAIDTVKKAYDLINNYNNQIAILQCTSTYPTSFEEIHLNVIRSYKRLFPGVVIGYSGHELGIAVPSAA
VAIGAKIIERHFTLDRTMKGGDHAASLEPLGFKKMVRDIRNIEKALGSSEKTVQKSEIKVFKKLGKSLVTLKDID
INTIITREMITTKGPGTGISPMELGSILGKKTKVHLQKDVIIKETDIEW

>OUX54928.1_Flammeovirgaceae_bacterium Bacteroidetes

MKRNFILPNNTLDNNSSAFIIAEIGINHQGSIDIAKKLILQAKDCGANAVKFQKRSISRILTKEGLEMPYKNRNS
FGKTYGEHKYALELSKDDYRELKKFTDNLGLLFSASGWDEESIDFLMEIDVPFFKMASADLSNFPLLEHTAKTGK
PIILSTGMADMELVKKAVDLVSKFNSNIAILQCTSTYPAQFNEINLNVIRTYLDIFPKYVIGYSGHELGIAISGV
AVALGAKIIERHFTLDRTMKGGDHAASLEPHGFSKMVRDIRHIEGAMGIREKELQESEKPIFKKLAKSIVTSKNI
KKGEKIKSDMLTTKGPGSGISPTKFNDIIGKSLNKDVSIDVIIKDEDIIWS

>OIO66895.1_Candidatus_Marinimicrobia bacterium CG1_02_48_14]

MSTLQLGSKTVGDGNPTYIIAEIGINHQGDVKIARELVRQAALAGADAVKFQKRNVERILTRDGLEMPYDNPNSF
GKTYGEHKRALELTEGDYHELLSFANEMNVDFTASGWDEESIDFLDTLGIDFFKMASADLTNFPLLEHTAKKGRP
MILSTGMADLEMVKQAYQLVRSFNLQIALLQCTSTYPSRFNEINLRVLETFRMAFPEAVIGYSGHELGIAISEVA
VALGAKIVERHFTLDRTMKGGDHAASLEPGGLQKLIRDIRHIEDALGTGEKRIQESEAPYFKKLAKSVVSTMPIP
QGTIITRDMLTTKGPGTGISPARIGDVVGKSPKVDLKGDVVIREDDIEW

>Neisseria_meningitidis_NeuB_NnaB_1XUU In Complex With Mn2+ And Malate

MQNNNEFKIGNRSVGYNHEPLIICEIGINHEGSLKTAFEMVDAAYNAGAEVVKHQTHIVEDEMSDEAKQVIPGNA
DVSIYEIMERCALNEEDEIKLKEYVESKGMIFISTPFSRAAALRLQRMDIPAYKIGSGECNNYPLIKLVASFGKP
IILSTGMNSIESIKKSVEIIREAGVPYALLHCTNIYPTPYEDVRLGGMNDLSEAFPDAIIGLSDHTLDNYACLGA
VALGGSILERHFTDRMDRPGPDIVCSMNPDTFKELKQGAHALKLARGGKKDTIIAGEKPTKDFAFASVVADKDIK
KGELLSGDNLWVKRPGNGDFSVNEYETLFGKVAACNIRKGAQIKKTDIE

>Methanococcoides_burtonii_NeuB_NnaB_WP_011499634.1 Archée

MQKMDRISENAPVFIIAEAGVNHNGSIALAKQLIDVAAKSNADAVKFQTFVTEDVVSINTPKAEYQKHTTESSES
QFEMIKKLELSKTDHKELMKHAEQKNIMFLSTPFDERSVDLLVELGVPLIKISSGEITNHPLLKYISRKGIPIIL
STGMSTLEEVAEAVSVIKDAGCEDLTLLHCTSSYPARVEDCNLLTMETMADVFDVQVGYSDHTSGICVPLAAAAM
GACVIEKHFTLDKNLTGPDHMASLEPTELEEMVRGIRLVEKARGSSVKAPVESELEVRDIVRRSIVAKVNIPAGT
VIAEDLLAFKRPGVGMPPKYLDRLIGKVPISEIKKDSLINFEMFETI

>Treponema_pallidum_SpsE_NP_219001.1 ssp pallidum str. Nichols]
Spirochaetes

MFTCGGRCFRPDADILTIAEIGSAHAGSFDRARALIDAAADAAAAAVKFQLIYAHEILHPLTGAVRLPSGAVSLY
QRFEELEVPLSFYAQCFNHARSRGMLVGISPFGPRSATEALALKPDFLKVASPELNYPTLISTLAAAELPLILSS
GVCLLKEIEGALAQCRQYTKQGSSHALLHCITAYPAPETEYNLALLPALATIFNINVGVSDHSVDPLLVPLLARA
HGACIVEKHICLSRTDAGLDDSIALDPADFRTMTAALNSCARRSPSQIISFLHERGYAPHVVRAVIGSGEKVLAP
SERAHYQKSNRSLHYLHAYPRGTVLQKENLVIVRSEANLSAGEAPEHANLFVGAVLQRSVHAGEGARFGDIIQKG
TCL

>Bacillus_subtilis_P39625.1_SpsE Firmicutes

MAAFQIANKTVGKDAPVFIIAEAGINHDGKLDQAFALIDAAAEAGADAVKFQMFQADRMYQKDPGLYKTAAGKDV
SIFSLVQSMEMPAEWILPLLDYCREKQVIFLSTVCDEGSADLLQSTSPSAFKIASYEINHLPLLKYVARLNRPMI
FSTAGAEISDVHEAWRTIRAEGNNQIAIMHCVAKYPAPPEYSNLSVIPMLAAAFPEAVIGFSDHSEHPTEAPCAA
VRLGAKLIEKHFTIDKNLPGADHSFALNPDELKEMVDGIRKTEAELKQGITKPVSEKLLGSSYKTTTAIEGEIRN
FAYRGIFTTAPIQKGEAFSEDNIAVLRPGQKPQGLHPRFFELLTSGVRAVRDIPADTGIVWDDILLKDSPFHE

>Thermobifida_fusca_NeuB_YXQ47U14 Actinobacteria

MTQNATTDLSVSVPAPVAIGDILVGPGQPTYVIGEIGINHNGDVDIARQLIEVAAEAGCHAVKFQKRTPEICVPK
EQRDKIRQTPWGEMTYLEYKHRVEFGRDEYTEIAKICEENGLQWFASPWDVPSVEFLESFDVVAHKVASASITDH
ELLRALAATGKPIILSTGMSTIEEIDAAVEIFDRSKLILMHSTSTYPMPPEEANLRTIITLRERYGVPVGYSGHE
RGLQISLAAVTLGAVAVERHITLDRTMWGSDHAASLEPAGLQHLVRDIRVIEQALGDGVKRVFPGEEEPRKRLRR
TTA

>EIJ34212.1_Thiothrix nivea DSM 5205 gammaproteobacteria

MSVFIIAEVGVNHNGSLDLAKQLVDVANLCGADAVKFQTFKASTLVTKTARQADYQTTNTQKEESQFDMLTRLEL
SEAGHHALFEYCDRKNIEFMSTPFDLHSIQFLAELGVKRFKIPSGEITNYPYLSLIGSFNKEIILSTGMANLGEV
EAAIQVLLEAGTEQHKITVLHATTDYPTQMIDVNLRAMQTMAQAFKFPVGYSDHTPGIEVPTAAVALGASVIEKH
FTLDRNLPGPDHKASLEPDELKAMVVAIRNIEIALGDGIKRASPNEAKNKPIARKSIVAKQDIQAGKVFTLENLT
TKRPGTGVSPMRWHEVLGKTANRAFVADELIEL

>ADL05453.1_Clostridium saccharolyticum WM1 Firmicutes

MSKIMIIAEAGVNHNGDITIAKQLIDAASVTGADAIKFQTFKTDLLVVPASGKAEYQIKNTGGAMSQYEMLKNLE
LSWQAFEELYHYCALRHIKFLSSPFDEESILFLDRLGVDPIKIPSGEITNYGYLKKTASLKKQVLLSTGMSTEAE
IGEALEILEESGKEIILLHCSSAYPTMMEDVNLNAMATLKDRFHKQVGYSDHTPGIEVPIAAAALGACVIEKHMT
LDKTMPGPDHKASLEPDEFKHMADSIRNIEQALGDGVKRPTLAELKNRDYVRKYLVAAREIRKGEAFTLHNLCAK
RCGYGISPMELHSLLGKTADRNYQKDERIVL

>AEG18372.1_Methanobacterium paludis] Archeae

MEEIDKFNKDNCSCFIMAEAGVNHNGSLNLAKKLIDAAKESGADAVKFQTFKTENLVTKNAEKAEYQKETTTENS
QYEMIKKLELSEDNFRELAKYADEKDIIFLSTPFDFESVDLLDEIGVPAFKLGSGELTNFPLLEHVASKGRPVIL
STGMATMDEIKEAVKLFEDKTNDLILMHCVTSYPAKIDDINLKVIETLRSTFKLPVGFSDHTLGIEMPIAAVALG
SCVIEKHFTLNKNLDGPDHKASLEPADFKKMVLAIRNVEKGMGTGIKELTIEEKEIKKIARKSIVARANIPSETV
ITEEMLAIKRPGTGIEPKFLKSLIGKTTTSKIKKDDFLRWNLIK

>EFM37961.1_Campylobacter coli JV20]

MKKTLIIAEAGVNHNGDLNLAKKLIEIAADSGADFVKFQSFKAKNCISTKAKKAPYQLKTTASDESQLQMVQKLE
LDLKAHKELILHAKKCNIAFLSTPFDLESVDLLNELGLKIFKIPSGEITNLPYLKKIAKLNKKIILSTGMANLGE
IEEALNALCKNGTKRQNITLLHCTTEYPAPFDEVNLKAMQSLKDAFKLDVGYSDHTQGIHISLAAVALGACVIEK
HFTLDKNMSGPDHKASLEPHELKTLCTQIRQIQKAMGDGIKKASKSERKNINIVRKSLVAKKDIQKGEIFNEENL
TTKRPANGISAMRYEEFLGKIATKNYKEDELICE

>Q9NR45.2_NANS_HSA

MPLELELCPGRWVGGQHPCFIIAEIGQNHQGDLDVAKRMIRMAKECGADCAKFQKSELEFKFNRKALERPYTSKH
SWGKTYGEHKRHLEFSHDQYRELQRYAEEVGIFFTASGMDEMAVEFLHELNVPFFKVGSGDTNNFPYLEKTAKKG
RPMVISSGMQSMDTMKQVYQIVKPLNPNFCFLQCTSAYPLQPEDVNLRVISEYQKLFPDIPIGYSGHETGIAISV
AAVALGAKVLERHITLDKTWKGSDHSASLEPGELAELVRSVRLVERALGSPTKQLLPCEMACNEKLGKSVVAKVK
IPEGTILTMDMLTVKVGEPKGYPPEDIFNLVGKKVLVTVEEDDTIMEELVDNHGKKIKS

>XP_783181.3_NANS_Strongylo purpuratus]

MPLEFEIAPGRMIGNDHPCFIIAEIGQNHQGDINIAKQLIKVAKDAGADCAKFQKSELDFKFNQAALEKPYTSKH
SWGKTYGEHKRFLEFSHDQYRELQAYSKEIGIYFTASGMDEKAVEFLDSINVPFFKVGSGDTNNLPYIEKVAKMN
RAMVVSSGMQSMETMTRVYETCKKYNDKFCILQCTSAYPLHPEDVHLRIIQVFQEAFPDIPIGYSGHEGGLSITL
ASVALGAKVVERHITMDKSWKGNDHEASLDPEELAELVREIRIVERSLGSRVKEMRECELPTFNKLGKSVVAAKD
IPAGTVITEDMLTVKVAVPKGILPETIYNLFGKRTAKAL

>EEN59689.1_NANS_Branchiostoma floridae]

MVGGDHPCFVVAEIGQNHQGDINIAKEMIKKAKEAGADAAKFQKSELTRRFNRAALARPYTSPNAWGPTYGETNS
TWSSATISTRSSRLTPNSKASTSLPQQWMRYGLELLPADFLADELDVPFIKVASADANNLPYLKHTASKGKPMIL
SSGMQSMGIMERAVGTVTENMPGEKRLVVMQCTSTYPLPPADVHLRCINLFKEKFPHAIIGYSGHESGIAISLAA
VSLGAKVVERHVTLDKSWKGSDHAASLTFDELKDLCDNIRIVETALGKPIKEPRPTDIFTADLTVIKCSMIFTNE
EKTTKWTCLYMNVQCYDVFSVFSAGVKAAEPFGIPPDQIYELVGQTVTKAIEEDATIPKEALAA

>Q9VG74.2_NANS_Drosophila melanogaster

MLLNDIISGKLVDSVYIIAEIGQNHQGCVETAKKMIWEAKKAGCHCVKFQKSDLPAKFTRSALDREYISDHAWGK
TYGEHKEYLEFSKDQYLQLQAHCKELNVDFTASAMDERSLEFLSALNVPFIKIGSGDANNFPLLKKAANLNLPLV
ISTGMQTMQTVERIVQTMRESGKEDYALMHCVSSYPTDPKDCSLQLISVLRTRFPNVAIGYSGHELGVIISQAAV

LLGARIVERHFTLDKSQKGSDHRCSLEPQELKALTTAITNFKLSSVPMPPQEIVKKLNGDEELEAALQHVESKTI
LPCELPCRNKLGKSIVAARNLNKGYRLQLADMAIKVSEPSGLTAEDFLDLVGKELADNIGEDEPILGNSIIN

>XP_008190466.1_NANS_Tribolium castaneum]

MAELQITPKRTIGHNHPTFIIAEIGQNHQGDVKIAKKLIHLAKESGADCVKFQKTCLTEKFNKSALDRPYRGPHS
WGATYGEHKQFLEFTKEQFRELQSFAAEIGILFTASAMDSQSLRFLASLNVPFIKIGSGDSNNLLLIQEAAAMNV
PLVISTGMQDLGGVRATYECVAQYHKKFALLHCVSAYPTPLQEINLNVIKLYETEFPDVVIGYSGHELGIEASVA
AVAVGAKIIERHVTLDKTQKGSDHQCSLEPQELKLLIEKIRELDITLGKPVKAFQASERPCYAKLGKSLVAATTL
RKGDILQHENIKIKVAEPKGIDASLLKDVIGKHVKGVINEDESILQENLE

>KIX22924.1_NeuB_PseudAS_Flavobacterium sp. 316] Bacteroidetes

MHKNSPVFIIAELSANHNGSLETALETVKAAKRAGANCIKLQTYTADTITLDSEKPDFVIKGTIWDGRKLHDLYK
EAYTPWEWHEKIYKAAEAEGLICFSSPFDKTAVDYLENLNTPAYKIASFEITDIPLIEYVASKMKPIIISTGIAE
LIDIELAVEACKRVGNHNIALLKCTSSYPAPIEEANMCMVKDLAERFNVISGLSDHTMGATVPIVATALGAKIIE
KHFILDRSIGGPDASFSMNEEEFTAMVKAVREAEKAIGNVDYTLTEKQAKGKDFSRSLYIAENITEGEVFTESNL
RSVRPGFGLHPKYFNKIIGKKANQKLEKGTPMSLDFIQ

>XP_001618767.1_NANS_Nematostella vectensis]

NHNIALLKCTSSYPAPIEEANMCMVKDLAERFNVISGLSDHTMGATVPIVATALGAKIIEKHFILDRSIGGPDAS
FSMNEEEFTAMVKAVREAEKAIGNVDYTLTEKQAKGKDFSRSLYIAENITEGEVFTESNLRSVRPGFGLHPKYFN
EIIGKK

>EFX62694.1_NANS_Daphnia pulex]

MTARSFELVNNTWIGIKMAKDCGADCVKFQKSDLHSKFTKSVLARHYESPHAWASTYGEHKTFLEFSEDHYKELM
AFASLINIHFTASAMDEISLDFLVSLGVPFIKMGSGDANNLILLEKAAKTKLPIIYSTGMQDMQTVKTAMERMNA
CNPRVCILQCTSSYPTPEDQVHLRVMQSYRDHFPGSPVGYSGHELGLSITLAAAALGAHVIERHVTLDKSWKGND
HACSLDPLELKSLVEGIRAIEKALGSAVKSKQPSEEPCHQKLGKSVVCTQKLEAGVQLCSEHLTVKVGQPVGWPP
QHLDQLVGKTLSRNVDQDETITSDCII

>WP_018506507.1_pseudaminicAS_Thiobacillus thioparus] acid synthase
betaproteobacteria

MKPIQIAGRQIGPSYPPFIIAEMSGNHNQSLERALEIVEAAAKSGAHALKIQTYTPDTMTIDLDEREFHISDSNS
LWKGASLYKLYGEAYTPWEWHKPIFDRARELGIIPFSTPFDDTAVDFLEALDVPCYKIASFENTDLPLIRRVAAT
GKPLIISTGMATVAELDETVRAAREAGCMDLILLKCTSTYPATAENTNILTIPHLRELFGCEVGLSDHTMGVGVS
VASVALGASVIEKHFTLSRADGGVDSAFSMEPAEMTQLVLEAERAWQALGRVSYGPTQAEKKSLQYRRSLYVVQD
IKAGDVLTKENVRAIRPGLGLPTKFLGEILGKTVSQDVKRGTALNWGMLG

>WP_009207339.1_pseudaminicAS_Sulfuricella denitrificans] acid synthase
betaproteobacteria

MIKIGSREIGRQQAPFVIAEMSGNHNQSLERALEIVEAAAKTGAHALKIQTYTPDTMTLDLNEREFYISDPKSLW
VGTSLYKLYGEAYTPWEWHKSIFDRARELGIIAFSTPFDITAVDFLEGLNVPCYKIASFENTDLPLIRRVAATGK
PLIISTGMATVAELDETVRAAREAGCNDLILLKCTSTYPATAENTNILTIPHLRELFGCEVGLSDHTMGVGVSVA
SVALGATVIEKHFTLNRADGGVDSTFSMEPSEMTQLVVETERAWQALGQVSYGATEAEVKSIQFRRSLYIVQDLK
AGDVLTRENVRAIRPGLGLPTKYLEQILGKIVNQDVKRGTALGWGLF

>CBA31353.1_Curvibacter putative symbiont of Hydra magnipapillata]
betaproteobacteria

MKNISIAGRLIGKAWPPFVIAEMSGNHNQSLERALEIVDAAAQAGAHGLKIQTYTPDTMTLDLDEREFHIDDAKS
LWKGTSLYKLYGQAYTPWEWHKPIFDRAVALGMIPFSTPFDDTAVDFLESLNVPCYKIASFENTDLPLIRRVAAT
GKPLIISTGMATVAELDETVRAARDAGCKDLILLKCTSTYPATASNTNILTIPHLRELFGCEVGLSDHTMGVGVS
VASVALGATVIEKHFTLSRAEGGVDSAFSMEPAEMAQLVLETERAWQSLGDVSYGPTDVEKKSLQFRRSLYLVHD
LKAGTVLMPDHVRAIRPGFGLPPKYLTQVLGRRLNRDVVRGTALAWDLLG

>Hydra_vulgaris_ABRM01085349.1 WGS

GRLIGKAWPPFVIAEMSGNHNQSLERALEIVDAAAQAGAHGLKIQTYTPDTMTLDLDEREFHIDDAKSLWKGTSL
YKLYGQAYTPWEWHKPIFDRAVALGMIPFSTPFDDTAVDFLESLNVPCYKIASFENTDLPLIRRVAATGKPLIIS
TGMATVAELDETVRAARDAGCKDLILLKCTSTYPATASNTNILTIPHLRELF-
GCEVGLSDHTMGVGVSVASVALGATVIEKHFTLSRAEGGVDSAFSMEPAEMAQLVLETERAWQSLGDVSYGPTDV
EKK-SLQFRRSLYLVHDLKAGTVLMPDHVRAIRPGFGLPPKYLTQVLGRRL

>WP_067217189.1_pseudaminicAS_Marinomonas gallaica] acid synthase gammaproteobacteria

MVNVMLNSPFIIAELSGNHDQDFDTAKAMIKAAAEAGVDAIKLQTYTADTMTLNVRNEEFMIGEADSLWQGQNLY
DLYGKACTPWEWHQPLFEYAKSLGLVAFSSPFDASAVEFLESLNVPLYKVASFELTDIPLIQKIAVTGKPTIMST
GMASLEEIEEAVAAFRHISDAELVLLKCTSTYPANMADSNLATIPDLAKRFDCRVGLSDHTRGIGASVASIAFGA
TVIEKHFVLDRSAGGVDAEFSMEPGEMAALVEACHQAYTAIGSIMYGGSDAEQKAKKYRRSIYIAQDLPAGTLLR
PEHIKIIRPSLGLAPKHWNEVIGRTLSADVQLGQPLSWDILD

>WP_087462101.1_pseudaminicAS_Oleiphilus messinensis] acid synthase gammaproteobacteria

MTQALSEVRIASRRVSDQEDPFIIAEFSGNHAQDLAIAEEMVRAAARAGVDAIKLQTYTADTITLDGKSREFQIR
DDSSLWKGETLHSLYKKACTPWAWHSQLFSLARENGLIAFSSPFDETSVELLESLDVPCYKIASFELNHFPLLKA
LARTGKPIIMSTGMATLEDIDASVEYLYQQGCSELVLLKCTSSYPAPIKDANLRTIEDMKRRFQIPVGLSDHCQG
IDVALASVALGANVIERHIVLDRNTEAVDGAFSSTPEEFSRLVGQTKNISAALGKVQYGPSESELDSLKYRRSIY
VTQNLEPGAILTAENIKVIRPGYGLAPKFFEEVLGKKVNKAKLANTPLHADDID

>Bankia_setacea_MRUH01000164.1 WGS Bivalve

GREISAAAAPFVIAELSGNHAQNLDTAKAMIMAAAAAGADAIKLQTYTADTITLNSTNPEFQINEASSLWQGENL
HSLYQKAHTPWQWHAELFQLAKDQGLIAFSSPFDETSVDFLESLQVPCYKIASFEINHFPLLKKVAATGKPVIMS
TGMSSSEEIEEAVAHLRENGCTDIALLKCTSAYPASVADANLATIADMKIRFGLPIGLSDHSLGIGVAIVAAAMG
AEI IERHFVLDRDSNAVDAAFSSTPAELAQLVKEASQIKSAIGS

>Elaphomyces_granulatus_NPHW01007775.1 WGS Fungi Asco

VGDGYPVYIIAEIGINHNGSLEIAKKMIDGAVFAGADAVKFQKRTPEFCVPQHQWNIERDTPWGRMTYLEYKRKI
EFNFDDYERIDKHCKEKGIQWFASCWDVDSVNFMEKFDPPVYKAASASLTDIELLKKMKSTGKPLIVSTGMSTMQ
EIEESIETAGRDNLLIAHSTSTYPCKAEELNLKMVQTLKEKYHGNPIGYSGHEVGLSPTWAAVAMGASFVERHIT
LDRAMWGTD QAASVEIMGLYRLVSNIRDIQRAMGDGIKKVYESELSSLKKLRK

>Tuber-aestivum_CZPR01000680.1 WGS Asco

VYIIAEAGVNHNGERDLAFALVDAAAAAGADAVKFQTFDAARLASKSAPKATYQKQTTDAAESQLAMLKKLELPK
EWHADLQAHAHRSGIEFISTAFDTDSLKFLCEMNLPFFKVPSGELTNGPLLWQFAHTGKPLVLSTGMATLSEVEQ
GLAIVAHALATDREPANLDEVWKNWSQPQARTRLKGHVTLLHCTSQYPTPWGEVNLRAMDTLRAFDLAVGYSDHT
EGGLISVAAVARGAKVIEKHFTLDRSMPGPDHKASLEPDELKAMISQIRVLELALGDACKAPQPSEWDTRRAARQ
QVVAARDIAKGSIIGRDDLTTARADSGFPAAALWELVGTRSEFAVQAGEII

>Melampsora_pinitorqua_AUYS0100826.1 WGS basidio

IIAEIGQNHNGDMDAAEALIRAAKAGGADVAKFQVYDARALFPPKESNPWFDYNCKTELQRGDVVRLARCCEKVG
IEFMASVFDTSRIGWLEDVGVKRYKVASRSVRDTALLAALAKTKKPLIVSLGMWNEPSFPSIPSAAHVDFLYCIS
KYPTPLSDLRLGKVDFKKHSGFSDHSEGITAACGAFVLGARILEKHLTLDKTSYGPDHSCSMTPDDLAAIDRFRR
DWEECAG

>Cronartium_ribicola_AWVX0102625.1 WGS basidio

QFIGVNHRPFIIAEMSGNHNQSLDRALAIVEAAAACGAHALKLQTYTADTMTLDLSHGEFFISDPASLWKDTSLH
KLYQEAYTPWEWHKPIFDRCRELGMIGFSTPFDESAVNFLEELDVPLYKIASFENTDIPLIQKVAKTGKPMIIST
GMASVAELDETVRAIRELGCENFVLLKCTSTYPSTPAFTNLNTIPHLRQLFNCQVGLSDHTGGIGAAVAAVALGA
TVIEKHFTLRRADGGVDSTFSMEPEEMEELVLETERAWQALG

>Lentinus_polychrous_JSYW01008743.1 WGS basidio agaricale

ADVVKFQIYTGTALVSPVESPDRHAHFKRFELTPDQHIALAEMCRAAGVGYVSSVWDLESLEWIDPYMDFYKIGS
GDLTAWPLLRNFAQRGKPILLSTGLATLDEILQTVAFIQSVDDRYRQPEWLCLMQCTSMYPIPDSDANLCVMDTL
RAMTGLSIGYSDHTTGMMALRAATAMGAEALEFHFTLSREGQTFRDHLVSLTADEVLALKDDIAQIIALRGRGVK
VPQPSEVSTGHTTSFRRAAYACRPVAAGATITADDLCV-LRPAHGTDARDFDRIVGATALHAFE

>Acytostellium_ellipticum_FLTD01002867.1 WGS Amoebozoa

MTVSIGSRVIGGGRPAYVIAEIGLNHNGDVDIAKKLIDVAARAGADAVKFQKRTPEISTPEHMRDVPRETPWGVM
SYLDYRRRVEFGRDEYVEIGDHATMLGLDWFASPWDVPSVAFLEDLNVVAHKVASASLTDTELLIALRETGKPVI
LSTGMSTIEQIDRALDTLGTDRVVLMHATSTYPLEPEEANLRVIATLRDRYPGVPVGYSGHERGLQISLAAVAIG
AVAVERHITLDRTMWGSDHAASLEPTGLEHLVRDIRVIERATGDGVKRVFDSERAPMAKL

>Dictyostelium_deminutivum_FLTF01003146.1 WGS Amoebozoa

MTVSIGSRVIGGGRPAYVIAEIGLNHNGDVDIAKKLIDVAARAGADAVKFQKRTPEISTPEHMRDVPRETPWGVM
SYLDYRRRVEFGRDEYVEIGDHATMLGLDWFASPWDVPSVAFLEDLNVVAHKVASASLTDTELLIALRETGKPVI
LSTGMSTIEQIGRALDTLGTDRVVLMHATSTYPLEPEEANLRVIATLRDRYPGVPVGYSGHERGLQISLAAVAIG
AVAVERHITLDRTMWGSD

>WP_094706949.1_pseudaminicAS_Hahella sp. CCB-MM4] acid synthase gammaproteobacteria

MAFKNTVTINGRTLGNGHPPYIIAELSANHNGSLERALSIIDMAHRCGADAIKLQTYKADTITFDSDKEDFQIHG
GLWDGKTLYELYEWAHMPWDWHKPLFEHARKLGITIFSSPFDDTAVDLLEDLGAPAYKIASFEAIDLPLIRYTAS
TGKPLIISTGMATEDEIQEAAAAAADAGCQELVLLHCVSGYPAPAEDYNLRTLTDMMDRYNCLVGLSDHTIDNAA
AIASVALGACLIEKHVTLDRKGGGPDDSFSLEPDDLKMLCESSRTAWQALGSVDYSRKASEVGNAKFRRSLYAVK
NIRKGEPFTLENVRSIRPGFGLAPKHREEILGKSAAHDIEAGTALNWDLIEH

>WP_092376349.1_pseudaminicAS_Desulfomicrobium apsheronum] acid synthase deltaproteobacteria

MTVMINGRAIGPDHPPYIIAELSANHNGSLERALQTIEAAHRCGADAVKLQTYTADTLTIDCDAPDFLIKGGLWD
GFKLYDLYKWAETPYEWHQAMFDHARNLGITIFSTPFDETAVDLLEDLGTPAYKIASFEIVDLPLIRYAASTGKP
MIMSTGMASEEEIDEAVAAAHEAGCKDLILLHCISSYPAPIEQANLRQIPELARRFGVISGLSDHTMGTTAAVTA
VALGACVIEKHFTLSRQDKGPDCEFSLEPHEMERLCVEAKDAWRALGKAGYTREEAEEKSKIFRRSVYFVRDLKK
GAKITRDDIRRIRPGMGLAPKYYESIIGKKTKLSVLKGTATSKDNILFNNENQ

>Acanthamoeba_palestinensis_CDFD01053174.1 WGS Amoebozoa

PMNSIIIQNRPVGAAHSPYIVAELSGNHNGSIERAKDIILEAHKCGANAIKIQTFKPDSITLPCYRDEFMIKKGI
WQGSNLYELYKKTCLPYEWHQELFSYAKNVGITIFSTPFSEADVEFLESLDTPAYKIASNELTHLPLIKEVINTK
KPLILSTGTATIEEIEQTVEFLHQHSCNNFILLYCVSSYPAPLEDYNLSTMKEIQNRFGCLVGLSDHSMGIIAPI
VATTMGACFIEKHFTLDRNDGGSDSSFSLEPAELKLMCDSVRSAYACIGSPTFGYKDCEKR-
SPIFKRHYYAIKDINKGEILTLDNIAA-VRSPQGISSSQYNFVLGKKAINNIQKHDPILAKDVENYEKV

>Paramecium_biaurelia_JPFL01000669.1 wgs

VLIIAEAGVNHNGELQKAIELIDAAAHAKADYVKFQTFKAEKIVNPTAKKAQYQVTNMQGEGDTQFEMLKKLEMG
DDWYPILIQRCQEKGIHFLSTGFDQDSIDFLNDFEIPFYKIPSGEITNKPYLQHIARKGKDIILSTGMADLKEVK
EAIEVIEAEGITRNRITVLHCNTEYPTPMQDVNLLAMNQMAEKLGVQIGYSDHTLGIEVPIAAVALGAKVIEKHF
TLDRNLPGPDHAASLEPAELKEMVKTIRNIELAISGSGIKEPSASENKNRAIARKSLHLREDLKKDTVLQAHHLM
ELRPGDGISPMDIDQVIGKKII

>WP_029638846.1_NeuB_alphaproteobacterium Mf 1.05b.01]

MNHVTVIAEAGVNHNGSLERAIEMVDVAADIGVDVVKFQTFNAEALVTRSAPKADYQKETTEESETQLDMLRALE
LDVHAHRTLIQRCAEKGVQFLSTPFDLGSLDLLANELNVATLKVGSGELTNAPLLHACATAGRDLILSTGMATLD
EVQTMLGVVAHGFLGESSPSEKAFKEAFESGAGKAVLKSRVKLLHCTSSYPTPDGDVNLRAIETLRAKFDLPVGF
SDHSDGIVHAIASVAMGACIIEKHYTLDKDLPGPDHKASATAEELSDLVDGIRRVSAGLGDGCKEPQPAEISNMA
IGRKSLVASTPINAGDVFTTENLTVKRPGTGMRPELYWSLLGTHAARAYAPDDMITQ

>WP_045444639.1_Neub_Tepidicaulis marinus] alphaproteobacteria

MSKPPYVEIIAEAGVNHNGSLERALKLVDVAADAGADVIKFQTFSADAIATKAAPKAAYQKLETGKAQSQYEMLK
ALELDEAAHRTLITRCAERGIEFLSTPFDFPSLDLLANRLELARLKIGSGELTNAPLLLAIARTRRPVILSTGMA
RLEEIEAALGVLAFGYGEGGVPSKSAFQAAYKSGAGQRALRANVTLLHCTSAYPAPDGEINLKAMDTLRQKFGLP
VGFSDHSVGIDIPIAAAALGAAMIEKHFTLDKTLPGPDHKASIEPDGLRALVSGVRRVSAALGDGIKEPRGVETE
TADVARKSLVAARPIAKGELFTEENLTVKRPGTGLSPLAFWSLLGKPAPRAFAPDELIGL

>Prorocentrum_minimum1_JXLM01000037.1 wgs alveolate

SVIAEAGVNHNGSLERAIEMIDVAADIGVDVVKFQTFNAEALVTRSAPKADYQKETTEESESTQLDMLRALELDA
QAHRTLIQRCAEKGVQFLSTPFDLGSLDLLANELNVATLKVGSGELTSNAPLLHACATAGRNLILSTGMATLDEV
QTMLGVVAHGYLGPDRPSEKAFKEAFESDAGKASVLKSRVKLLHCTSSYPTPDGDVNLRAIETLRAKF-
DLPVGFSDHSEGIVHAIASVAMGACSIIEKHYTLDKDLPGPDHKASATPEELSDLVDGIRRVSAGLGDGCKEPQP
AEISNMAIGRSKSLVASTPINAGDVFTTENLTVKRPGTGMRPELYWSLLGTHAARAYAPDDMITQ

>Condylostoma_magnum_CVLX01013231.1 wgs alveolate

IIIAEAGVNHNGDINLAKALIEKAAMAGADYVKFQSFKASELTTKLAQKADYQKENTNPTDSQLDMIKALELSYE
NHKSLIDECKKHHIRFLSTAFDEQSLNMLLELNCLDFIKIPSGEVTNLPFLRVIASKNKPIILSTGMCDEDEVAN
ALAVLTQAGVKKEMITILHCTTDYPTEMQDVNLKAMQTMGKKFGTKIGYSDHTIGIEVPIVAAAMGASVIEKHFT
LDRGLAGPDHKVSLIPDELSQMVKAIRNIELALGDGVKKPTKGELKNRPIARKIIVAKCDINKGDKLNDKNLTTK

>Prorocentrum_minimum2_JXLM01007734.1

YVIAEIGVNHEGDFDTAKRLIDLAKEGGADAAKFQTYKAEKIASKHSPAYWDQNAEPTNSQRELFLKYDAFEEKD
YVALAEHCQNVGIDFASTPFDAEAVSFLDPL-
MTYFKIASADLTNIPMLRLIANTGKPVLLSTGASSLAEIDIAVNELISHGCENLTLLQCTLNYPCPYENANLNMI
DGLKRAFPQFEIGYSDHTHPDPDMMILTAAWLKGASILEKHFTHDKSLPGNDHYHAMDVDDLKTFRANVALLSTA
LGDSHKHPLP

>WP_035074769.1_Desulfovibrio zosterae] deltaproteobacteria

MKISNFDTDKKIFIIAEIGNNHEGDFELAKNMVIKAVAAGADAVKFQTIVPCELVSSDQLERLAMLEKFQFSYEQ
FAELSALAKKEGALFMSSPFDEITVRELNKFVPAFKVASCDVTFYPLLKEIAATGKPIIMSTGAASEEEVLNSCK
YIESCWEKAGIKSPGLALLHCVSSYPTPVNEANLKAVSTLAKTGYVAGYSDHTLGIDAAVLSIGLGARIIEKHFT
VDKNYSEFRDHQLSADPNEFKLMVEKIRSAEALLGSGDITVANCESGNRVLIRRSAAARGNIAEGSIVESDNIRW
VRPGTGITPDKSAMIIGRKATRNLIDGELFKTEDVI

>SBT09427.1_Candidatus_Propionivibrio aalborgensis] betaproteobacteria

MKIGDIDLSREVMVIAEIGNNHEGDFGLARDMILAAAAAGAHAVKFQTIQPDQLVSASQPARLEQLGRFAFSRDE
FAELATIAGAAGTVFLSTPFSPEVVPWLDELVPAFKIASGDNNYASLLAAVAATGKPVLLSTGMAGMTGIREACR
IIETAAQKSGHSAHIALLHCVSAYPTPPAQANLRAISTLADEIGGVVGYSDHTLGIEAAVLSVALGARIIEKHFT
LSKTQSEFRDHALSADPAEMTELVRRVRLAQELLGTGEKLPQEAEAPVIAAARRSIVARLDLPAGHVLTEHDLEW
LRPGGGLMPGQESIVLGHRLLRPVVRGEMLLPDMVA

>WP_008313751.1_NeuB_Leptolyngbya sp. PCC 6406] Cyanobacteria

MKIGRHSLEQDVFVIAEIGNNHEGDIQLAQEMIHAAAASGVQAVKFQTIIPERLVAKSQSARISQLSRFAFSPDQ
FANLAATAAKAQVEFLSTPFAPEVVPWLDDLVPAFKVASGDNNYWALLEAIALTGKPVLLSTGMSYLADVKQAIA
VIETVWQNHGTQSTLIPLHCVSAYPTPLEQANLGAIQTLAQETGQIVGYSDHTLGIEAAIFSVLLGARVIEKHFT
LSKTQSDFRDHALSADPNEMAELVQQVQRVQAILGNGVKTIQDAELPVAAAARRSIVARTSLTAGHVIGLEDLEW
LRPGGGLSPGQEAALLGRCLQQAVAQGEMLTAEMVA

>WP_096701985.1_Magnetospirillum sp. 15-1] alphaproteobacteria

MKIGPIDLDRDVLIVAEIGNNHEGDMALAEEMIAQAAQAGAHAVKFQSIIPAHLVAPDQPARLAQLGRYQLSAEN
HVRLAQVARDNAIMFMSSPFSLDAVDLLAPLVPAMKVASGDNDHVVLLDRIAATGLPVILSTGMTDLDGAAFSAG
TLCRAWAKAGIADPGLVLLHCVSSYPTPADQANLRAIRTLAGLGHVVGYSDHTLGIEAAVLSVALGARVIEKHFT
LSKTQSEFRDHQLSAEPHELKALVERVAEANALLGDGVKRVMPGEEATAQAARRSLCAAADLPAGTVLGLGDLAW
LRPAGGMAPGAEWRILGRRLTRPVAAGDRLALDMFEQ

>Chromera_velia_ARZB01016018.1

MLVAEQDGDVVGFLQALQSSNGTLVIDLLAVLVVAEIGNNHEGDLGRAEEMIRRAAEAGAQAVKFQTIDPARLVA
ADQTARLQQLQRFALTPDDHRHLAAIAADAGVLFLSTPFSLEAVAMLEPLVPAFKIASGDNDFAPLLAQTARTGK
PLLISTGMSRPEDIAQAVTRVRMAWATAEFRDPGLVLLHCVSAYPTPPSAANLRAILALPTYGAVAGYSDHTLGI
EAAVLSVALGARVIEKHFTLSKTQSDFRDHQLSAEPSELAELVERVREAETLLGDGIKRIHEEEAPVAAAARRSI
CAARDLTPGQTVAPQDLTWLRPAGGLSPGSEADLLGRSVRRPVPAGTALTLDMMD

>Condylostoma_magnum2_CVLX01030206.1

GRAIGPDHPPYIIAELSANHNGSLVRALETLEVAKQCGADAIKIQTYTADTMTINCSMPDFMIKGGLWDGFKLYD
LYKWAETPYEWHKPLFEHARELGITVFSTPFDETAVDLLESLDTPAYKIASFEIVDLPLIRYVASTGKPIILSTG
MATEDEIDEALTMAHEAGCKDLILLHCISSYPAPMDQANLRQIPELSKRFDILTGLSDHTMGTSAAVTATALGAC
IIEKHFTLNREDKGPDSEFSLEPDELKRLCIDTKEAWMALGQAGFERQKAEKDNKIFRRSIYFVKDLPAGHVIGP
EDIRRIRPGMGLAPKYFNDVLNKKLKVSVQQ

>Reticulomyxa_filosa_ASPP01005516.1 wgs Rhizaria

REIGPAHPPLVIAEIGINHGGSLEVAKEMVRLAHLSGCECIKHQTHFLDDEMTDEAKAIFPPNADVSIWDVMARC
ALSAGDEVALKDYTESLGLIYISTPFSRKAADFLAEIGVPAFKIGSGEADNLPLIRHIARFGKPVILSTGMQTIE
TIRESVAILDAAGVDYALLEC

>Cladosiphon_okamuranus_BDDF01000009.1 wgs stramenopile

YIIAEIGINHNGDLSKALEMVRKSKEAGCDAVKFQKRTIDIVYSREELARPRENVFGPTNGDLKSGLEFGKDDYD
QIDELCSELSIDWFASPWDEPSVDFLMKYKTPYLKIASAMVMDRDFLKHCASTGRPLLVSTGMCDLPMIRRAVET
IEAANGEIACLYHCTSTYPTLDEEINLMGIQQLQQAFPALRIGFSGHEQGILPSVCAAALGAKSVERHVTLDRSD
WGSDQKASLEMSEVAELVSQIRRVEVVRGDGQVRFYEDEKPIAEKLRR

>Phytophthora_lateralis_AMZP02003661.1 wgs stramenopile

FIIAEAGVNHNGDEKMAVELVEAAAKSGADAVKFQTFSADKLTRKGAEKAEYQKLATGDGDQHGMLKALEMSESL
HRRLFARCTELGIEFMSTAFDEEALDFLVALGIKRIKVPSGEITNAPLLRHMASKGLPLIVSTGMAELDEVVAAI
GIIRAARESHGFAEPLGDIVTILHCTSNYPAESADVNLRAMNTMARTTGLPVGYSDHTLGLAVSTGAVALGACVI
EKHFTLDCELPGPDHKASLEPDQLVALVRQIRDVEVALGSDIKAPTASELPVRDLVRRSVTTVRPLAAGAVVGRE
DVTLMRPGTGIPPVDLDKVIGRKSARHIPAGETV

>Cladosiphon_okamuranus2_BDDF01000073.1 wgs stramenopile

LINGRKIGTEYPPFIIAEMSGNHNQSLDRAMQLVESAAESGVHALKLQTASPEGLTLDIESPEFMIDDPASPWHG
RNLYQLYKEAVTPWEWHKPIFDRCGELGLTVFSSPFELSAIDLLEELNAPCYKIASFELVDLPLIRKAAATGKPL
IMSTGMASVSDIHDAVQAAKAEGNDSIILLKCTSSYPATAADANIATIPHMRETFGVQVGLSDHTLGIGVPCAAA
ALGATVIEKHFTLRREDGGVDSTFSLEPWEFAALVEESERAWASVGRVVYGGAAVEQKSLKFRRSLYIAKAVKKG
EVLTEQNLRIVRPGYGLAPKHIDVLLGRRV

>Aureococcus_anophagefferens_ACJI01001814.1 wgs stramenopile

PCKVIAEIGCNHMGSFETAKELMLLAKATGAGYAKFQKRCPRELLTKEQYDAPHPVPHNAYGQTYGAHREFLEFS
KEQHEQLHAYGKEIGIEWATSVWDVTSAREMADIPCDYLKVPSACNNHFDMLKVLRDEYAGDVHVSTGMTTLAEI
EDVVAFFEATGQAKRLVVYNCTSGYPVPFPSVCMLELVRLYERYGDRVKELAFSGHHLGIAIDVAAYTLGAKWIE
RHFTKDRTWKGTDHAASLEAGGFGKLVRDLEATHQALTLKASEILPIEDEQRDKL

>Emiliania_huxley_AHAL01004984.1

MTRIDFKQPHVMAEIGCNHMGEFETAKELLTLAKEAGASVGKFQKRCPKELLTPEQYAAPHPNPRNAYGDTYGAH
REYLELTVDQHRELKHCEEIGLGYSCSVWDVTSAKEIVSLNPILIKVGSPSNQHWEMQKVLRDEYGGDIHISTGM
TTKEEVEKINCTSGYPVPFEDVSLHATACEQVPFEDVAIGFSGHHLGIAVDVAAYAMGAQWNERHFTKDRTWKGT
DHAASLEPAGLTKLCRDLQATWKCMTPKASEILPIEKEQRAKL

7 - NANP: N-acetylneuraminate-9-phosphate phosphatase (Neu5Ac-9-Pase; E.C. 3.1.3.29)

>NP_689880.1_NANP_Homo [Homo sapiens]

MGLSRVRAVFFDLDNTLIDTAGASRRGMLEVIKLLQSKYHYKEEAEIICDKVQVKLSKECFHPYNTCITDLRTSH
WEEAIQETKGGAANRKLAEECYFLWKSTRLQHMTLAEDVKAMLTELRKEVRLLLLTNGDRQTQREKIEACACQSY
FDAVVVGGEQREEKPAPSIFYYCCNLLGVQPGDCVMVGDTLETDIQGGLNAGLKATVWINKNGIVPLKSSPVPHY
MVSSVLELPALLQSIDCKVSMST

>XP_019642312.1_NANPlq_Branchiostoma belcheri]

MALNTKKVAAIIFDLDNTLIPTKEADDEAYELVREVIEEACPGYDTAAMTAKFRVDLGWNDGCDPEGKLTVDEWR
TKLWENILNKQGITQYNGLPAKMYATWKRERLKRMVFTEEIKQMLVGLRKEYKLQMMTNGPLEPQREKSQVCESD
VYFDSIVLCGLYPEQKPYPSVYQRALQDLQLNADQCVMVGDRLNTDIXGGLNAKLLSTVWINSTGAAIPEGGPVP
HHIVTSVLELPKILDIINGTPPASA

>XP_787883.3_NANP_Strongylocentrotus purpuratus]

MGIAAVIFDLDNTLIWTKQSDANAFVQVARFVQKETPSCNAEEIVSTFRKLLQSAEKDPENKIPIDEWRTQLWKT
ALNSNQNEEFAARVYQLWKKLRLEGLYFDEEVRAQLKRLRLRYKLLLLTNGDSQVQREKVAQIGAEDFFDEIVIS
GDHPEPKPHPSIFKTSCKLLGVEASQCVMVGDSQETDIQGGANARVLATVWINPHGKQPSSDYVKADYTIKSVLE
IDSILQQLHPPS

>KXJ84308.1_NANP_Aedes albopictus]

MGFVIQNYIDAIIIADLLHKEYDLPRDLATEVSTTFLTSYRRCPDNPDVPLAQWRIQLWEDALPSDRKHLAALVY
PRWVEYRTRYLAPSPEIISMLQTLRLQYLLGIVTNGPSASQWEKIDRLALGRFFDCILVSSDLPWAKPDRNIFYA
ACHYLGVRPEECAMIGDKLETDIQGGLESRLAATIWLPLPKDLRLMHDKSLADMGDLAHPDYIVESVLDLPSVLP
LMTSFCGQRCRPVQERRDSYSYIPKTTRNRQASYNRFLPDVPDLYCSSSNSCDSNGTMDSQ

>XP_008195993.1_NANP_Tribolium castaneum]

MENGRPGISAILFDLDNTLIATRKADKQTCSQLAQILWEKWDVPTDFATNASKAFLKAFRKCPENLSMSLDAWRR
LLWAQALGDQYNKYAGEVYRMWLQLRYDNLALSPEIQNLLIKLRQHYFVGLITNGTSRAQWEKIQLLHLQSFFDV
VLVSGDLPWEKPHREIFNIACEYLGVEPQQCIMVGDKLETDILGGLKAKLGGTVWVPLNSIEVGDEDPRPDYVIK
NVTELPNLLPKNPKVPRFRGREELRTRQISAPDFEDSNSNSSDGS

>XP_017870155.1_NANP_Drosophila arizonae]

MAASKFSTATSLTTGLKHTTPFDGICAKISAFYFDLDNTLIPTRAGDSKAIRKLADVLETQYSFTKDDANLATQN
FLKSFRRCPDNSQTSLDSWRTHLWRESLQQKHKHLAEQIYPQWLKLRYRYLAIPPDYVQLLQRMRRAGYLLALIT
NGPSNAQREKINKLHVRGHFDCVLVSSDLPWEKPHPEIFYAACNFLGVKPHECAMIGDKLETDIKGGHLAQLGLT
FWLPLSTSSAASQCLDDVEYKPHVKLNSLLDLYKYFPRLNAVAAAATPSTTNRRSSHSQSSSGSGSISGSGIGSG
AGSNSDHDRGAGVRQQPYGEDSYQRQSAYRRGGSLPAMDCSNSEAENSCDSFI

>XP_015928753.1_NANP_Parasteatoda tepidariorum] Common house spider

MGLNKFQNVTTILFDLDNTLINTKGADRLACQQVSQYLQNCGIRRSTAREVVDKFYNLIREQPHDPNNEDGDVDA
WRVLLWKQALGPGLSTKASIAYSMWKESRTIHLKFDLALQNMLVELRKHYKLGLITNGPSSSQWEKIRKVNGSIF
FDAIVVSGDVKHSKPGVAIFEDAFRILDSSSLECVMVGDNLNTDIKGGIRANCAATVWISDEGLPDREGCPKPDF
QVSNVTDIMDLLPERKGFSFLCQDRSVGLYHSMRDETHTGRVN

>CDG68557.1_NANP_Hydra vulgaris]

DIIEVLKSFNLLKLLFTEVYKKIKCRMNCFTNRNRGIILDFDGTLVQTNAASKFSLNSAKDYLVEKYNILPATAN
LIVNEFVTLVNKSEKAIVYESDEDLWRKQIWQKVLIDNKAANVEIDTFYSYYKESFLELIEIKKEVKKMLKNLQN
SFKIIILTNGNSQWQRKKLEKSEANKYVDDIIISGEHKISKPDPRLFQLACLKLGLESKQCIMVGNNKKADIFGG
FNAGLKATIWIRDKEYDNDSIQPDYIIDDICELESVLVKIF

>XP_001640674.1_Nematostella vectensis]

MAVKGLLFDFDNTLVQTNKSDLEALEKVKQWLMETLSEEQALAATSEFSRLLHEHWVDPDGTKSVHEWRTSLWLK
AINILPENITNITAGELYSFWRESRVKGLGIPTGVQFLLEGLGHQYKMAIITNSDPVIQKEKLEFCKVEKYFDAI

IISGEQPEAKPCVSIFQTACDAIGLAPEDCVMIGDNLVDDIQGGRDAGVRATVWVRGEDAKGPSEKGMKPNFVVQ
SLLELPEVLNKIV

>XP_020912840.1_NANPlq_Exaiptasia pallida]

MADGSDRFVKGILFDFDNTLVTTTKSDIKALENVKERLSNDFXQENVEAXANKYLELLAIGSIDPEGKVDPHLWR
TLLWKKAIDFVSKNDENPKAESIYNLWRFARLEGIKLEKEVADLLKNLREKXKLSLVTNSDPIIQREKLQSCGII
EYFDXXIISGDEPXPKPHPSIFIKACANIGVPPEQCIMIGDNXRHDIQGGVNAGLFATVWVKKESQQEMTISDPR
PDYTIDSVIELPKILATIS

>KLO97606.1_Fusarium fujikuroi]

MHSIFVSTNSATPVMSLPPPDNLFTIESQPTRTMVFFDLDNTLFNHQQSIYYAMSAVRILLPLPREIPLDVLVDK
YNEALDLVYNQYLRNEIAHEDQDSVKVKLFFKSLHLEEPDSKCIARFRSVYKRTYRGKRKAMPGSIETLRSLRKN
GYRTAIITNGPTEIQIEKAKAIGVFDLVDCVITSQEAGHPKPDVRIFQYALEKLDVKPDDAHMVGDSVEADIKGA
LDAQISPILYSPGSNSSLKLLFGKEIPVIRQFDQLPMVLEPPLDSGLD

>OPB44498.1_Trichoderma guizhouense] asco

MELTRMKVVFFDLDGTLFDHYHSLRLAITAIQRNYNELEEKNVDELIDKYNLALQRAYDAYLDKVITYEQADVQK
IHLFFSSLGLPEPSLDEIQKFRDTYKAVYRKNRRATPGSIEALARLREHGYRIAIITNGQVEDQTTKAKAIGIEH
LIDRIITSEEAGYRKPDRRIFQYAIEQLGASLDTTCMIGDSAESDIKGALDAQMFAIMYSPTAQDSQKLLFGQQI
PIIRHMAQLPGYLGIGDH

>CCI44548.1_Albugo candida] stramenop

MYRFGSVCARAPRFFPTIRSQSFVSKALDRCKPSLVIFDKDGTLIDFNLMWGGWVEQQAWKVEMTTGLPVREKLF
DAMGYNWIHRSIRSKGALCCTPMGELYKIAVKVLIGEGTSADRAEQVIQQCWSMPDPVTTSRPLVNDLSALFKTI
KHMQIKIAVCTTDDRAPTIETLKHLNLFPMIDAIACGDDGLPAKPAPEQIWTICQSIGVEPQNTIMVGDTSTDMN
LGKNAGCGLSVGVIGGASSLEDLAEEADVLISSVDKVVKVLFQYGLQASRCVP

>XP_007512834.1_NANP_Bathycoccus prasinos]

MSTLKAVFFDLDDTLVPTSVYDVRAYADVKTSVLAWVGEEKFQSLDVDKLVADFKEKFVKAPWDPEYKTDVFTWR
SRVWEHALKLQNVENAEEVGVKCQKCFDDNRMGTFPLHDCVKELSEYINSKGLEMCIITNGHHRVQRDKLEACSA
YTLFENIIVGGEEVLAGRKEKPDRGIFMKACKYVGCLPSEAIHVGDSLGADIQGGINAELLATVFINVKSRDAST
FQPMPTYTIKHIAELKEIIDKLLVE

>XP_001415685.1_Ostreococcus lucimarinus CCE9901] Prasinophyceae

MSREGDPNGASRAPLRAVFFDFDDTLAETTLADRVAYRECAIRMETVYGLSKKRQDEVIAAYKRRLAERPWNDEF
AHVWTHRERLWAEAFGDDDRGLAMRHDVNSTFRDCRLEQLRLNSSVCGGIEKLRAKNVHVVIITNGHHVVQREKL
AACGIYEVVKLENILVGGEEVLAGRDEKPEASIFHEACKRVDVVPDEVMHVGDSWTADMVGAENAGLRWRVWVSQ
RPDDEKCESEQELSSSKRAKKVDAVPRVENIKEFFELLDEWLDEDGTLPASNILLKTRSRSER

>XP_005772526.1_Emiliania huxleyi CCMP1516]

MRSLRRCRAVIFDLDDTLVPTSKIDRAAILHAAALASGDELPAVVAARFSELLKAEPFPPEPSGLDVPAWRTGLW
ERALSGSSSDGRAGASPAARQAYEAWSSERLSSFRFADDVDAMVRRLQSAGYKTGVLTNGHADVQRAKTAACGAG
SLFGEERVIIAGAGDASTATVARARPLMGGCGPLGEHAEQKPHASIFRVASATLMVGDSYAADVAGGINASLLAT
VWVRPPLEPPETGAQGSLMHGHLSAVPAGQPSPTFTVESVLEVEACLEKIG

>ETR71346.1_NANP_Candidatus Magnetoglobus multicellularis str. Araruama]
deltaprotbact

MKYKKNHTIKSIFFDLDHTLVDCATADKRTYEILATIARKQLDTINTPSLIHDFRQLLIKQPFDPDGRIDVHTWR
IDLWTQALSKQNIDHEDLARQLNHAFHFERLAFYVLDHSVQTLLNDLLQTYTGIIITNGDTTIQRPKLAACNAHQ
YFGQNIIVGGEEPHEKPHASIFEKACDMADCSPQEAIMVGDRLHTDIQGGINAGLAATVWVNPNNDPLDHNGPEP
HFQVVSVLELPAILSGLHK

>WP_009842834.1_haloacid_dehalogenase_Vibrio sp. AND4]

MLKAIFFDMDETLCGTSQADKVAGQEFATWVAQTYPQVNDSVALVQRYLQGVYKKLNAEFPQLIALLPDENAFRC
GLIQVILAEQGIEIDAEQAQQAQNVFDSARMQAFTFFPGVKEMLTELRQHYKLVVITNGPVFSQHPKLKATQMSE
WVDHIIVGGEEPEEKPAASIFHKALNLVGASPEEAIHIGDSLPADIAGANNMGMLSVWVNATGTENPTDIEPHYE
VRETIELNEILKTLTQ

>WP_007107590.1_HADfamhydrolase_Natrinema altunense] Archaea

MGTETTTPLEAITFDLDGTLLRYERSPGTVLEAAFERAGVEPLFAVDEYYARYDDFAERCDSMAALRSECFAALA
AANGYDRRLGRDVAAAFNDERDQSNVELVPPAARVLDELSREYRLAVITNGARDAQRQKIDAVNLERWLDEVVVA

GHDTPPKPDPEPFEQAMRLLDATPATAVHVGDSLETDIVGSAAAGLESVWVSDRSDGSGAEPTYRVASIGDLLTP
PWLSQSETEGQ

>KPV63575.1_HAD-hydrolase_Candidatus Bathyarchaeota archaeon BA1] Archaea

MFLQVDAVLFDLDETLIDSFKGQQAAHIEVSKMLISSLKLRGIQVNFQDLLRQVSDFDDEMEVKLIRERDNWWPT
LFRMLGGRRLSKKTAQRLTKAYWTAYANNSPPYEDAIPTLEYLRSKGYKIGLVTDTDGTPGMKRWRVALTGLEKY
VDAVVVGGEDIMERKPSPELFLLAARRLGAPAERCLMVGDKAFVDIKGAIAAGMKAILIHRREWDIPLKPHHTIH
RLAELRSLL

>SNQ44313.1_NANP_Cellulophaga lytica] Bacteroidetes

MEVDFKNIKVIGFDADDTLWVNETYFREAEETFAKLLEGYETKNKIDQELFKMEMQNLGRYGYGIKAFVLSMVES
ALELSNNKVSNTVIAKILNIGKQMIDQPVELLPGVESVLQKLQSKYRLIVLTKGDLLDQEQKLEKSGLKKYFHHV
EVLSDKKEENYKNLLDHLQIKTSEFVMLGNSLKSDVLPIVKLGAQAVHIPFHTTWVHETVSKEEEESNSFLTLNK
IEDILEYLK

>APD07702.1_NANP_Flavobacteriaceae bacterium UJ101] Bacteroidetes

MNLNSIQHIFFDLDNTLWDFIKNSKITLENLYKEHHVEEEHHIAFSTWYDHYYDINEQLWAEYRDHKITKQELKD
SRFRKAFQAVGVKDTSLPEAFEAVYLNHLPKNNFLRDGAHDLLEYLKKQNKYTLHIITNGFKDVSLKKIKGSGID
SYFDVVVSAEDVNTRKPDPKVFQHALDLAQASKEESIIIGDDYIADIVGGLDFGIQAIFYNILDMEVKNNNFIEI
KHLNEVKKYL

>AMQ60564.1_dUMPphosphatase_Klebsiella aerogenes] gammaprot

MKWDWIFFDADETLFTFDSFSGLQRMFLDYSVTFTAKDFQDYQAVNKPLWVDYQNGAITSLQLQHQRFDSWAERL
NVPPGELNDAFMNAMAEICAPLPGAVSLLNALQGKVKMGIITNGFTSLQQTRLERTGLRDHFDLLIISEEVGVAK
PDARIFDYALAQAGNPSRDRVLMVGDTAESDIRGGVNAGLATCWLNAHQQTLPADLQPDWTVTSLSELEQLLCKH

>KLD50379.1_dUMP_phosphatase_Escherichia coli] gammaprot

MKWDWIFFDADETLFTFDSFTGLQRMFLDYSVTFTAEDFQDYQAVNKPLWVDYQNGAITSLQLQHGRFESWAERL
NVEPGKLNEAFINAMAEICTPLPGAVSLLNAIRGNAKIGIITNGFSALQQVRLERTGLRDYFDLLVISEEVGVAK
PNKKIFDYALEQAGNPDRSRVLMVGDTAESDILGGINAGLATCWLNAHNREQPEGIAPTWTVSSLHELEQLLCKH

>EHX56208.1_FMN_phosphatase_Escherichia coli DEC13B]

MGRISALTFDLDDTLYDNRPVILRTEREALTFVQNYHPALRSFQNEDLQRLRQAVREAEPEIYHDVTRWRFRSIE
QAMLDAGLSAEEASAGAHAAMINFAKWRSRIDVPQQTHDTLKQLAKKWPLVAITNGNAQPELFGLGDYFEFVLRA
GPHGRSKPFSDMYFLAAEKLNVPIGEILHVGDDLTTDVGGAIRSGMQACWIRPENGDLMQTWDSRLLPHLEISRL
ASLTSLI

>WP_021711615.1_FMN_phosphatase_Vibrio azureus] gammaprot

MKFYRSIKPIKAITFDLDDTLYDNVAVIVKMEQELLAWFKEHHPVVAQMSAEDWRVVKRRLLREKPALKHDVTLL
RFVQIQAVFLSQGYSSLQANQIAQQAVELALEWRNRVTIAPDNIALLQTLAEKVPLVAITNGNVDCKKIGLTPYF
QHILRAGPDGAAKPDRDMFQKAQQLLALPADNILHVGDHLISDVKGAISANFASCWFNNTGCQIRTLCHGGLLPD
MEVSQLASLSRLVEG

>WP_050914405.1_HADfamhydrolase_Vibrio harveyi group] gammaprotb
MLKAIFFDMDETLCGTSQADRVAGQEFANWIAQTYPQVADSTAFVQRYLQGVYKKLNHEFPQLIALLPDENAFRC
GLIQTILAEQGIEIDAEQAQQAQSFFDSARMGAFTFFPGVKEMLTELRQHYKLVVITNGPIFSQHPKIKATQMSE
WVDHIIVGGEEPEEKPAASIFHKALNLVDVKPEEAIHIGDSLPADIAGANNMGILSVWVNETGTANPTDIKPNYE
VKETVELKEILKTLAQ

Supplemental Data 3: Multiple sequence alignments (MSA) conducted with Clustal Omega (Sievers et al. 2011 NAR Clustal omega) at the EMBL/EBI web site. MSA were manually curated.

1 – MSA Transporter SLC35A1

```
SLC35A ver4
Sycon3                       VIFISYM-ALFINQGLLVTASKRKDG---SFAYNATTVVLMT EV-VKLGMAIFLHLREATV-TISTIRQNLQ-
Hydra                        ------------------------------------------ ----------MEAETLVGV-LFRDIVKHSN-
Tribolium                    VIFLAYI-LLFVGQGILVTASQKADN---QYDYNIITVVLLT EV-LKLIVSTLLYCKDNSP-LVNNIVENRK-
Drosophila                   VIFLLYM-SLFIGQGIFVTASQESNN---SYGYNTVTVVLLT EV-FKLIVSTCLYCRDHNL-LVRDVHKDRN-
Bathycoccus1                 VVTLALT-VLTSSQGLLIAASKANGV---KYDYAVTSANCTV ET-TKMLMSLLALVKIWRTNEDNRISTSWS-
Vitis                        IVTLALT-VLTSSQAILIVWSKRAG----KYEYSVTTANFLV ET-LKCALSLAALVRIWGNTDDNRLSSSLD-
Eleaeis                      IVTLALT-LLTSSQAILIVWSKRAG----KYEYSVTTANFSV EA-LKCALSLVALAKIWRSTEDNKLSTSFD-
Zea                          IVTLALT-LLTSSQAILIVWSKRAG----KYDYSVTTANFSV ET-LKCLLSLVALSRIWNSTEDNRLITSFD-
Oryza                        IVTLALT-LLTSSQAILIVWSKRAG----KYEYSVTTANFSV EA-LKCLLSLIALYRTWNSTEDNRLSTSFD-
Brachypodium                 VVTIALT-LLTSSQAILIVWSKRAG----KYEYSVTTANFSV EA-LKCLLSLLALYRTWNSTEDNRLSTSFD-
Bathycoccus4                 MMAATLT-LATSSQGLLTTASKSNG----EYRYNFATVPFLA EV-LKLVVSSLLLHRQFLIPKGTHITRDWK-
Bathycoccus3                 VVAATLT-VFTCSQGLLMEASKVRG----KYPYNSAVVPLLS EL-VKLILSILLLRRARARPAGTIMTTDVK-
Bathy_ct342                  AVAATLT-IFTCSQGLLMEASKVDG----KYPYNSAVVPLLS EL-VKLILSVMLLRRAKTRPKGTIMTMDLK-
Human_35A4                   TLMLLLSTAMYGAHAPLLALCHV----DGRVPFRPSSAVLLT EL-TKLLLCAFSLLVGWQA--WPQGPPPWR-
Human_35A5                   LLGAIFI-ALSSSRILLVKYSANEEN---KYDYLPTTVNVCS EL-VKLVFCVLVSFCVIKKQSRNLKYASWKE
Bathycoccus2                 LFLVALT-LENTASMLARRYAVGILQ----LDFSKNVVLCVN EF-LKLLFSLGMKYRKTDIRLKTHIFERVV-
Reticulomyxa                 ------------------------------------------ -------------------------------
Diplonema                    LSLLVLV-VQNAALVLLTRYSRS-QQ--KGELYRSSSLILNQ EL-LKLAACLGYMLWENRKQLYHAVF-----
Bodo                         LSLIVLV-LQNSTLVLLTRYSRFSQP--PGEMYHTSTLVLNQ EI-MKMVFCLVIFYIERKQELSSIVF-----
Trypano_grayi                ------------------------------MRYHTSTLVLNQ EV-VKMVLCIIIFAVENRLLLWRVVF-----
Emiliania2                   VALATFV-AQNTAAALLMRYAKTRME-----PYNSAVAVLLQ EVALKLPISVVLFAVECGGKIRRDFSERPL-
Guillardia                   AALVGLV-FQNTSLILFMKQASITPS-EDGKKALTTTVVVMV EF-FKITACILEIAYRRRKEIREEIVGKPR-
Sycon2                       LSLIVLV-LQTTALVLLMRYSRAVG---TGPMYLASTAVVMA EV-FKLVSSLLLFWWELARHLRTVLFSQPF-
Caenorh_elegans              ISLVVLI-VQTTALVLTLRYSQTQKS--EGPRYLSSTAVVCA EI-IKLITCFFVIYRNNG-ELNREIFASPQT
Caenorh_remanei              ISLVVLI-VQTTALVLTLRYSQTQVS--DGPRYLSSTAVVCA EI-IKLITCIFVIYRNAG-EMNREIFATPQT
Monosiga                     VALVLLM-VQTTSSILVLRYSRTR----EGGAYLSTTAVVMA EL-FKLLGSAVLLNYERR-YMYRELFINWV-
Salpingoeca                  IALLLLM-VQTTSSILVLRYSRTVNG--DGGHYISTTAVVMS EC-FKLVGSFFLLQRETG-HMYGEIMGNWK-
Amphimedon2                  LSLIVLV-VQNTSMVLVMRYSRTM----EGPRYLSSTAVVLS EI-TKFVTCFFLVLNGNG-ELKTEIIDKYI-
Human_UDPNAct                VSLGILV-FQTTSLVLTMRYSRTLKE--EGPRYLSSTAVVVA EL-LKIMACILLVYKDSK-VLHDEILNKPM-
Exaiptasia                   VCLGVLV-LQTSVLVLTMRYSRMVH---EGPMYISSTAVVMA EL-FKVIACVFVIFHHCG-ELNNDIMQKPL-
Acropora4                    ICLGILV-VQTSSLVLTMRYSRTHKQ-PGEQMYIASTAVVFA EI-FKVLACLAIIFRGSS-QLREEILAKPW-
Sugiyamaella                 LSLVTLT-VQNSSLILVMHYSRVMPGYTADSRYYASTAVLLN EI-IKFVFCTIVCVYQQ--SAFEDVF-----
Tuber                        --------------------MPK----TSANRYFTSTAVFLN EV-LKLSISIWVAYLERRKHLCGDVF-----
Anthurium                    VSLFTLC-IQNSTLVIVMRYSRL----DKENLYYTSTAVFLS EL-TKLIICLYVAGRNQIRELSSQIF-----
Entoamoeba                   IFLVLLC-CQTVIQSILGRYSRGVLH----ETYSIPSTIVFN EI-LKFLICLVMLKFVHKK-LLQHVIYLIK-
Emil_sc187_Ctg5084           VVLVLLV-AQTTSIVLLMRYSRTVVRPLDAPMYLASAAVFLA EA-AKLPCCLYMVARVGGGLLQSEVLNQPG-
Emilh_sc1_Ctg98              ------------VITLLARESRIPVA--GRPMYLGAAAVLVS EC-IKLPVCLAVISRQLGGDVRQRVVVGWR-
Emliania1                    GSLAVLM-VQNSSAFVVTRYTRQGK---PNELYLTSVVVLMV EL-IKMAICLLLLLRDARGELQHHLWVERR-
Bflori1                      YCLAVLT-VMAASYTVLMRYTRTVEG----VRYYSTTTVFVT EC-AKMFFTLCILLKEHKGELKGNIVXKPM-
SLC35A1_Callorhinchus        YCLTVMT-CVAATYTVLLRYTRTTLT----EMYFATTAVCLT EV-IKLILSLGIFAKE-TGHLRDHILRSPK-
Human_CMPSAt                 YCLAVMT-LMAAVYTIALRYTRTSDK----ELYFSTTAVCIT EV-IKLLLSVGILAKE-TGSLRENVLGSPK-
SLC35A1_Danio                YCLTVMT-LIAATYTVALRYTRTVST----ELYFSTTAVCLA EI-IKLLLSLIMLVRE-TGALVTHIFRSPK-
Nematos_sc43                 ------------------------------------------ -------------------------------
Acropora2                    FIFAILT-LQATCYFSLLRYSRTRPH----KMYFTSTAVFLA EV-IKLLVTLVVIVSQHRNFLVDSFLLNPM-
Chrysochromulina2            GSLGFLI-LQNSSHVLLLRYSRVVPGE-C-SQYVVSVAVLFA EI-CKLVFCVFVLCFTEGGVLDRDIWQRKM-
Aureococcus                  GSLALLV-AQDTALVLLMRYSRQRSG----SMYISSTAVCSM EV-MKLSVCFLMLLCGEAHMIRKEVLGRPK-
Diplonema_LMZG01004873.1     LSLGCLV-IQNSALTLTMRASRLSGGS-TGPMYLATSAVVVC EL-IKLVLSFIMLFLEKGSAINTDILTTPS-
Guil_sc140_Ctg3497           --------------------------------------VVVS EC-LKVLACVFILAYHHREVIWQEAIVNWK-
Albugo                       TSLCVLC-LQNSLLAVIMRLSRASGH----PQYNTHTAVLMG EV-LKVSISAILIVCVRFRSPFT-TIFDCK-
Nannochloropsis              VALLVLV-LQNSAVVLTVRYSRMRKD----VMYISSTAVVMS EI-VKLVVASFLVGMEEGGKLYHDIVLKPA-
Chrysochromulina1            --------------MLSMRYSKMPSQ----PKYLSTTAVVSA EV-VKIVLSFAVLFSQMGTFVWRAVFVAWK-
Phaeodactylum                ----------MVGLVLLMRYSRTQHDD-TQPLYLASTAVFLM EV-MKLVICVGVIAVQTKSELYTHTIGSPL-
Clunio_UDPGlut               VSLLTLT-VQNSAVSLCMRYAKTRDG----DIFFSSTAVFLA EV-VKLITCIGLVFMEEGTSVHNAIIKNKV-
Ceratitis_UDPNAcT            ISLITLT-LQNAILGLSMRYARTRPG----DIFISSTAVLMS EI-VKLFTCLVLVFNEEGKSLHKTIVANPV-
Apis_UDPGalt                 VSLITLT-LQNALVGLSMRYARTRSG----DMFLSSTAVVMA EV-VKFLTCLILVFIEEGNSLKLIIIKQPI-
Exophiala                    ASLLTLV-VQNSSLVLVMRYSRILPG----PRYLSSTAVVLS EL-LKCLICLTIHLREQRSQLWTDIFGVKS-
Capronia                     VSLFTLV-LQNSTLVLVMRYSRILPG----PRYLSSTAVVLS EL-IKCIICLSIHIRDQQTQLWNDLFSLKS-
Amphimedon1                  ASLFILT-IQNALLILSMRYSRIQVG----EMYISTTAVVLS ET-LKLFTCVVILFISEKKYLFQSVIVNWR-
Sycon1                       VSLIILT-FQNAILILSVRYTRTLEG----KMYISTTAVVSA EV-MKVAFCICVLISQRRGLLNEQVFQNWQ-
Acropora1                    WSLLILT-LQNALVVLTLRYTRTLPG----DMYLASTAVMIT EL-LKAVVSVAILFGQSGNSLYSITFGQPL-
Human_UDPgalt                ISLAVLV-VQNASLILSIRYARTLPG----DRFFATTAVVMA EV-LKGLTCLLLLFAQKRGFLHEAVLVQYV-
Bflorid2                     ISLVILV-VQNASLILTMRYARTMPG----DMFFSTTAVVMA EV-LKLVGCVLIIMCQYVGHLYSELFGNPM-
```

```
   Nematostella              -------------------------------MYVSSTAVIMA EV-FKVATCLVILLVMQGGHLYDSIIGQPI-
   Hydra1                    ------------------------------------------ ---------------EVKSYLYNNIIADPL-
   Acropora3                 ISLITLT-VQNASLILTIRYSRTLPG----DMYITTTAVVFA EI-LKVLASLLILLLQKGSFLYSSIIGQPF-
   Desulfovibrio             tavallttfiwglnf--------------------------- ------------vvirwgliigrng-lpwrl
   Phaeomarinobacter         LAFMVLINLIWGFAL--------------------------- ------------VAGKGSLFLS----IHWGR
   Acinetobacter             YAFVLITMCIWGGFTLTA------------------------ -----------RLNALWNIILLYRKEASFLL
   Pontibacillus             -FLVLLAAILWGTKGSAQTFTPENAH----PIAVGTMRLAVG GG--SLLF-VALLLGQVNL------------

Sycon3                        VFALYFVPAALYTIYNNLVFVNLKHYD-PTTYFILL
Hydra                         VLLLYMIPAFLYCLYNNLAFTNLRSYD-PTTYFLLL
Tribolium                     VLGLYFVPALLYCFYNNLAFVNLSVFD-PTTYYLLL
Drosophila                    VLALYMVPAFLYCLYNNLAFVNLATFD-PTTYYLLL
Bathycoccus1                  ELWVYPIPAALYLVKNLLQYYVFLYVD-APSYQILK
Vitis                         EVIVFPIPAALYLVKNLLQYYIFAYVD-APGYQILK
Eleaeis                       EVSVYPIPAILYLIKNLLQYYIFAYVD-APAYQILK
Zea                           EVRVYPIPAMLYLVKNLLQYYIFEYVD-APAYQILK
Oryza                         EVSVYPIPAILYMVKNLLQYYIFAYVD-APAYQILK
Brachypodium                  EVSVYPIPAILYMVKNLLQYYIFAYVD-APAYQILK
Bathycoccus4                  SALLYPIPSIIYLIHNNVQFLTLQYVD-PSTYQILG
Bathycoccus3                  SVMLFPIPSIIYVMHNNVQFYTMAYVD-AATYQILG
Bathy_ct342                   SVMLFPIPSIIYVMHNNVQFYTMAYVD-AATYQILG
Human_35A4                    QAAPFALSALLYGANNNLVIYLQRYMD-PSTYQVLS
Human_35A5                    DFMKWSIPAFLYFLDNLIVFYVLSYLQ-PAMAVIFS
Bathycoccus2                  TATPMLVPAFVYLVVNLISYPSLQRVD-ASVFTAIS
Reticulomyxa                  ----------------MLQYTALQNLP-APVYAVLQ
Diplonema                     ETVQLGVPAVLFTFQNYLLFVSLSHLD-AMTFQLLS
Bodo                          ETLKLVVPAGLFTLQNFLIFVALSHLD-AMTFQVLS
Trypano_grayi                 ETLKISVPAALFTLQNYLIFVGLANLD-ATTFQVWS
Emiliania2                    EWAQLSVPALLYLVTNISVYVGYDNLE-AALGMVMY
Guillardia                    ETMMLLVPAFMYLAQNNLLFIAVANLE-AVVYQVIA
Sycon2                        ELLKLAVPALLYTIQNNLLYVALSNLD-AATYQVTY
Caenorh_elegans               DSLKVAVPAIMYVIQNNLLFFALKKLD-AATYQVTY
Caenorh_remanei               DSLKVAVPAIMYVIQNNLLFFALKKLD-AATYQVTY
Monosiga                      SSLKLSVPALLYTVQNNLLFVALSNLP-AASYQVTY
Salpingoeca                   GTLKLSVPALLYTVQNNLLFIALSNLS-AATYQVTY
Amphimedon2                   ETLKVCIPSFLYTVQNNLLYVALSNLD-AATFQVTY
Human_UDPNAct                 ETLKLAIPSGIYTLQNNLLYVALSNLD-AATYQVTY
Exaiptasia                    ETFKLAVPSGLYTLQNNVLYIALSNLD-AATYQVTY
Acropora4                     ETLKLAIPSGLYTIQNNLLYVALSNLD-AATYQVTY
Sugiyamaella                  DCWKLSIPAFLYTLQNTLQYVAVSNLD-AATFQVTY
Tuber                         DNWKLAIPACLYTLQNSLQYIAVSNLD-AATFQVTY
Anthurium                     DAWKLMIPAALYTIQNNLQYIAVSMLD-AATFQVTY
Entoamoeba                    TSLVASVPGCIYFIQNMLLYIILQNTQ-AAVYTVII
Emil_sc187_Ctg5084            ETLKCAVPALAYTVQGNLLFAALSNLD-PPTYQITY
Emilh_sc1_Ctg98               DTLAMGLPALLFGLQNMLFFVSVSNLS-ATSYQLWS
Emliania1                     TTLLLGVPALCYAMQNNLVFVAISNLS-AAAAQVLY
Bflori1                       EMLKMSVPSI-----------------------VTY
SLC35A1_Callorhinchus         EMLKLSVPSVVYAIQNNMAFVALSNLD-AAVYQVTY
Human_CMPSAt                  ELLKLSVPSLVYAVQNNMAFLALSNLD-AAVYQVTY
SLC35A1_Danio                 ELLKLSVPSVVYAIQNNMAFVALSNLD-AAVYQVTY
Nematos_sc43                  -------------------------------FQITN
Acropora2                     DTMKLSLASVLYVIQNNLVYIAMTHLE-STTFQVLN
Chrysochromulina2             DTIKVSVPALCYTLQNNLQFVAATHLG-AELLQLLY
Aureococcus                   EVAKLALPALLYLIQNNLLYFALSHLQ-ATPYKVTY
Diplonema_LMZG01004873.1      QNFYLLIPSALYTIQNNLQYFAASNLD-PASFQVLY
Guil_sc140_Ctg3497            DTLKLSVPAFVYMIQNNLLYVATSNLD-AATCQVTY
Albugo                        EMIRISIPALMYVVQNNLQYVAISNLD-AAVFQVLY
Nannochloropsis               DFAKLLVPAFLFTIQNNLLFVALSNLD-AASFQVLY
Chrysochromulina1             DTLLVGVPAFIYLVQNNLLYVATTHLD-AATCQVAY
Phaeodactylum                 ELLKLTVPSLLYTVQNNLLYLALTNLD-AATYQVCY
Clunio_UDPGlut                DTMKMTVPSLIYVLQNNLLYVSAANLD-AATYQVTY
Ceratitis_UDPNAcT             DTLKVCVPSLVYIVQNNLLYVSASHLD-AATYQVTY
Apis_UDPGalt                  DTLKVSVPSLLYIIQNNLLYVSASNLD-AATYQVTY
Exophiala                     GFLKLLVPAVLYTLQNNLQFVAASNLD-AATFQVTY
Capronia                      GFLKLLVPAVLYTLQNNLQFVAATNLD-AATFQVTY
Amphimedon1                   DTLKLSVPALVYMVQNNLQYIAVSNLD-PAVFQVTY
Sycon1                        DTLKLSIPAILYTLQNNLQYVAVSNLN-AATFQVTY
Acropora1                     DAAKMLVPACIYTVQNNLLYIAVSNLD-AATYQVTY
Human_UDPgalt                 DTLKLAVPSLIYTLQNNLQYVAISNLP-AATFQVTY
Bflorid2                      DSLKMAVPALVYTLQNNLAYVAISNLS-AATFQVTY
Nematostella                  DTLKLSVPALIYTIQNNLQYVAISNLD-AATFQVTY
Hydra1                        STFKVAIPSFIYVLQNNLQFIAISNLD-AATFQVTY
Acropora3                     DTLKLSIPALIYTVQNNLQYIAISNLD-AATFQVTY
Desulfovibrio                 -vl--atgslvgvgyfsllfvgmqlgmppglsalvs
Phaeomarinobacter             DVMLILCAGPIGFG---FFFAGLAIAE-ASVVAIAS
Acinetobacter                 EAFI--LAMIGGVIYCLFAYSAFHYAPTAHAAIFLN
Pontibacillus                 ---I--LASLSMAFYQPMFFSAVHITGVAIGTVIAI
```

Sycon3 QLRVVATGIIFQVLFGKQLTRIQWSSLVLLMIGCAVKNVRFWSPAVPRLDLHLLYLLFQVFASC
Hydra QFRVVITGVIFQFLFNKKLSRTQWFSLILLTVGCIIKHLHLSKETF-TLNMSLLMILLQIFCSC
Tribolium QLRVVVTGILFQVIFSKTLSKKQWLSLLILTFGCMLKQINFTNQEFDIVGLNGIFILLQIFCSC
Drosophila QLRVVVTGILFQIIFKKYLSQRQWISLIMLTLGCMMKQIDFGSFYF-DFSLSAVFILAQTICSC
Bathycoccus1 NLNIISTGILYRIFLKKILSGVQWSALILLALGCTIAQLTSGSDQLSTPFMGLMMAIVMAILSG
Vitis NLNIISTGVLYRIILKKKLSEIQWAAFILLCAGCTTAQLNPSSDHLQAPFQGWMMAIVMALLSG
Eleaeis NLNIISTGVLYRIILKKKLSEIQWAAFILLCAGCTTAQLNPSSDHLQTPFQGWVMAIIMALLSG
Zea NLNIISTGVLYRIILKKKLSQIQWAAFILLCAGCTTAQLSPSSDHLQTPIQGWVMAIVMALLSG
Oryza NLNIISTGVLYRIILKKKLSEIQWAAFILLCAGCTTAQLNPSSDHLQTPIQGWVMAIVMALLSG
Brachypodium NLNIISTGVLYRIILNKKLSEIQWAAFILLCAGCTTAQLNPSSDHLQTPIQGWMMAIVMALLSG
Bathycoccus4 NLKIVTTGLLFRIILKRHLNRLQWIALALLMIGATISQISCDKGTLAAPLMGYVLGIISACLSA
Bathycoccus3 NLKIVTTGILFRFALGRLMTRTQWIALLLLTVGATVSQISGCKGELSAPMAGYALGVLSACLSA
Bathy_ct342 NLKIVTTGILFRFALGRMMTRTQWIALLLLTVGATVSQISGCGDELSAPVVGYMLGILSACLSA
Human_35A4 NLKIGSTAVLYCLCLRHRLSVRQGLALLLLMAAGACYAAGGLQVPLHITPLGLLLLILYCLISG
Human_35A5 NFSIITTALLFRIVLKRRLNWIQWASLLTLFLSIVALTAGTKTLQHIRLGMGHVLIIVQCFISS
Bathycoccus2 NLKVLATAIFAQILLNSRISNRVWRTLTQLVLGVTLISWESSPNNDLSYAFGVFLALVQTMLSG
Reticulomyxa QLKILSAAVCSYLILRESLASRQWRALFLLMAGGILIENHTFEIHANDPVKGTIAILTIVSLSG
Diplonema QVKLLLAALFSVWILDRYLTCVQWTSVVVLTFGIVLASQVPGTEKHQNVLLGAFSCVASGTSSA
Bodo QTKLLSAAVFSVWLLDRRLNRWQWISLVLLTLGVYFSQQTAQRESYQSFFLGAVACIISGLSSS
Trypano_grayi QTKLLSAAVLSVLMLGRRLSMMQWVSLFVLTLGVLLTQQQDLKKTSQRPLLGIAACVLSGISSS
Emiliania2 QSKIVFTAIFSVTLLNKKISVNQWCGGGAPA-----RALSPGRRHEKNRVVGMGAFLLAAVCTS
Guillardia QLKILTTAGFSILILERKLTIQQWSSLVLLTIGAAVVQVDNSSPGNLSSTIGLACALLAQCTSG
Sycon2 QLKIMTTAIFSVLMLGKQLDVKKWTALVMLMFGVACVQMPSSSSKGSS-VVGLVAVLSACCSSG
Caenorh_elegans QLKILTTAIFSVTMLGKSLHRYNWMALILLTAGVALVQYPSGDSTASDNILGLGAVLAACFSSG
Caenorh_remanei QLKILTTAIFSVTMLGKSLHRYNWLALILLTAGVALVQYPSGDSPASDNILGLGAVLAACFSSG
Monosiga QLKILTTAIFSVIMLGRSLNMYQWLSLVLLMGGVALVQMPSSSDEIGNQIVGLIAVLSACCSSG
Salpingoeca QLKILTTAVFSVTMLSKVISSRQWISLVLLMAGVALVQMPADDGSNKNQFVGLVAVLSACCSSG
Amphimedon2 QLKILTTAIFAVTLLRKQILKSQWLALGMLTLAVALVQWPSGGSENSMKLVGLVAVLLACVSSG
Human_UDPNAct QLKILTTALFSVSMLSKKLGVYQWLSLVILMTGVAFVQWPSDSQLAGSQFVGLMAVLTACFSSG
Exaiptasia QLKILTTALFSVAMLNRKLNAYKWFALVLLMAGVALVEWPTDGDKASSKFVGLIAVLSACCSSG
Acropora4 QLKILTTALFSVCMLHKRLGMLKWLSLVLLMIGVTLVQWHVDEKALSGQVVGVLAVLTACCSSG
Sugiyamaella QLKILTTAFFAVTILHKDLSKLQWLCLVLLTFGIGLVQMPPEAFNEMNGLIGFVAVSIACILSG
Tuber QLKILTTALFSVLMLHRNLNAKKWASLIMLTVGIAIVQLPAAAKSEMNRAIGLIAVAVACTISG
Anthurium QLKILTTALCSVIMLKTSLSGIKWFSLVLLTFGVALVQMPSGDSVSMEKIVGLGAVAVACVISG
Entoamoeba QLKVFTTAIFSVLFLGRKLSVAQWRALALLVTGVILVEISTNRYSTENNLLGIVLSLVMACCSG
Emil_sc187_Ctg5084 QTKTLFTAVFSRMMLGRRLAHSQWLALFLLFLGTVLVSDLCEPHPR-PRPLATRRDLGAAVLSS
Emilh_sc1_Ctg98 QSKTLTTALFYVLYLGGALRRLQWLSLSLLCAGVGLVQASDSVSAAGRPLLGISAVLASSLLSG
Emliania1 QLKTISTAFFTVLLLRRSFRAAQWVSFLLLMAGVALVQSEDGKSSGAAPWLGVSAALAAASISG
Bflori1 QMKIPCTALLSVMMLGRSLSSMQWIAVFVLTGGVILVQGIGGEAVEGSYVVGLTALTIAVFCSG
SLC35A1_Callorhinchus QLKIPCTALCTVWMLNRSLSKIQWLSVFVLCTGVTLVQWKPVETTEQNPVWGFTAIAIAVLCSG
Human_CMPSAt QLKIPCTALCTVLMLNRTLSKLQWVSVFMLCAGVTLVQWKPAQATEQNPLLGFGAIAIAVLCSG
SLC35A1_Danio QLKIPCTALCTVLMLNRSLSRLQWFSVFMLCAGVTLVQWTPPHSTEQNPFLGFMAIAVAVLCSG
Nematos_sc43 QLKIMTTALFSILLLNKSISRKRWFYLLMLMIGVAVVEIELHRKISKSFLIGFLSVLAASVISG
Acropora2 QLKILSTAIFSIALLKRQLTKRKWISLFLLSLGVTMVQVDSQTSVKQSSILGFSAVLASSLASG
Chrysochromulina2 QTKTLSTAIFGVVILAKQLRCNQWCALTVLVAGVVLAQSSQRKPGKGDAAIGVMAALGVSICSG
Aureococcus NLKILTSAFFSVTLLGQRLGRRRWISLVVLFLGVTIVQTDNPKNELGSQTLGFVAVGGAAITSG
Diplonema_LMZG01004873.1 QMKLVTTALLSVVMLKRSLSPTQWFSVFILTAGIAMVQTSEAPVTDENHLLGFIAVLAACCTSA
Guil_sc140_Ctg3497 QFKILTTALFTVTMLNRTILPLKWLSLVILVIGIALVQLPNIGAGSGNPALGLSAVVAACFMSG
Albugo QLKILSTAIFSVAIMGKSILPVQWISIIVLMLGVALVQFDESNESEQSTLTGLIAVVCACICSG
Nannochloropsis QLKILTTAVFSVVLLNRQLTCRQWLSLLVLIVGVSLVQTSGLKDGNGSTSLGFVCVLLASCSSG
Chrysochromulina1 QLKLLTTAFFTVTMLKRHISNRRWAALGLLFVGVVMVQAPAGSSGGQSPVLGMAAVFGACFLSG
Phaeodactylum QLKILTTALFSALLLQRKFSTMKWLSLVVLTIGVAIVQLSGSGDQAHTRWVGLVAVLCAACTSG
Clunio_UDPGlut QLKILTTAVFAVIMLRKKLIPTQWGSLLILVLGVALVQLSQGGETAQNRFLGFSAALGACCLSG
Ceratitis_UDPNAcT QLKILTTAMFAVFILRRRLINTQWGALVLLIAGVVMVQLAQTVDDEQNRMLGLWAALGACFLSG
Apis_UDPGalt QLKILTTAFFAVVILRRSLRNTQWGALILLVIGVVLVQLAQSSDTEQNHLLGFSAALSACFLSG
Exophiala QCKILTTALFAVLMLGQTLSAKKWLALVILTAGVACVQIPSSTTSPGNYMLGIIAVAVACLCSG
Capronia QCKILTTALFAVVMLGQSLSSRKWLALVILTAGVACVQIPSSTTPQGNYLIGISAVTIACICSG
Amphimedon1 QLKILTTAVFSVIMLQKTLTRTQWGSLFLLFIGVTLVQLKLASSNNNSQIVGLLAVIVSCLSSG
Sycon1 QLKILTTAIVSVILLGKVLTRIQWTSLVILFVGVCMVQVQLQNPKKQNPILGLIAVIISAFSSA
Acropora1 QLKILTTALFSVVMLKKSISKIQWFSLFMLFVGVSVVQLQPVTDKSQNPVLGLAAVVASSLCSG
Human_UDPgalt QLKILTTALFSVLMLNRSLSRLQWASLLLLFTGVAIVQAQQAGGGDQNPGAGLAAVVASCLSSG
Bflorid2 QLKIMTTALFSILMLGKSISRMQWVSLFLLFAGVSAVQLESTGATEQNPLLGLIAVVVSCISSG
Nematostella QLKILTTALFSVLMLNKSLGRLQWLSLVMLFAGVSIVQLQSSSTKKQNALLGFGAVVMSSLCSG
Hydra1 QLKILTTALFSVLMLNKSLTKGQWFSLFLLFVGVALVQFQPNQVNSQNPIVGLTAVVVSSLCSG
Acropora3 QLKILTTALFSVFMLSKNLVKLQWVSLVMLFAGVSIVQLQPTNLSTQNPFLGLVAVIMSCLCSG
Desulfovibrio qaqvlftalfsvwllrqrptawqwcgmgvagaglyl-------sadlaplagfalvllaalfwg
Phaeomarinobacter QMAVPISTIFSVMFLGERIRWRRWLGITLAFLGVMV-------ISVFSYIEGLIFVLCSAIVGS
Acinetobacter GCIPLCTAIVAYFLLNQAFDKHTWASLVIMIIAICLMSILMY---GIPFGMGDVLFFISAILWG
Pontibacillus GSAPILAGVLERVFLKRRLSKVWWYSTFLSILGCTLLFINTGSVQGILMALGAGLSFASY---A

Sycon3 FAGVYNEYLIKSNGPLMLQNVFMYVDSIVANI--VALALTQSDAVSSHNV-HALLDWNVIAII
Hydra FAGVYNEYLLKDKGPFMLQNVFMYTDSVICNV--LLLSYSGENVFLKKNI-DSVLHPIVLTVV
Tribolium LAGVYNEYLLKKQGNIFIQNVFMYLDSIVCNV--VLLSVRVSSAFTYENI-SKVFHYKVLLVM
Drosophila LAGVYNEYLLKDKGNIFVQNVFMYLDSIVCNA--VILLLRGEDAFSPHNL-SSIMRFSVLIII
Bathycoccus1 AAGVYTELIMKKQPNVNAQNVYLYLFGVIFNM--VAIFLYD-DAVFGRGYFYGYNA-IVCTMI
Vitis FAGVYTEAIIKKRPNINVQNFWLYVFGMVFNA--VAIVIQD-DAVMNKGFFHGYSL-ITVLMI
Eleaeis FAGVYTEAIIKKRPNINVQNFWLYIFGMIFNL--IAIVVQD-DEVMNKGFFHGYSF-ITVCMI

```
Zea                          FAGVYTEVIIKKNPNINAQNFWLYIFGMLFNL--VAICVQD-DAVMNKGFFHGYSF-ITVLMI
Oryza                        FAGVYTEAIIKKRPNINVQNFWLYIFGMLFNL--VAICVQD-DAVMNKGFFHGYSF-ITVLMI
Brachypodium                 FAGVYTEAIIKKRPNINVQNFWLYIFGMLFNL--VAICVQD-DAVMNKGFFHGYSF-ITFLMI
Bathycoccus4                 LAGVYTEKLMKMN-NLYWQNIQLYGFGVIFNG--LRLFFDD-NVGYSNVVTRGYNI-ITWFVV
Bathycoccus3                 TAGVYTEFLLKKN-NLYWQNVQLYAFGVVFNG--LRLTWDD-FGENSGDCTNGFTA-ITWLIV
Bathy_ct342                  TAGVYTEFLLKKN-NLYWQNVQLYAFGVVFNA--LRLTWDD-FGENSGDCTNGFTA-ITWLIV
Human_35A4                   LSSVYTELLMKRQRPLALQNLFLYTFGVLLNL---GLHA---GGGSGPGLLEGFSG-WAALVV
Human_35A5                   MANIYNEKILKEGNSIFIQNSKLYFFGILFNG--LTLGLQR-DQIKNCGFFYGHSA-FSVALI
Bathycoccus2                 FGSVYFEKVLKKRTDVWDRNIQLALCSILIYVP-ISIYE------TKGNLFQGWTF-LVIFIA
Reticulomyxa                 VAGVITQLLLKNKSSVWDRNAQLAFWSILIGVF-SLILDYQ-WITDHKHIFFGWTL-NTWILV
Diplonema                    FASVYFEKILKGTPSIAVRNVQLGIFSIILAV--GSMLVIDG---RSFVYFGGYSA-LTWVLV
Bodo                         FAGVYFEKVVKTTSSLAVRNIHLSLFGIPFAL--LSLLVLDIL--PRFRFWQGYNG-MTWCLV
Trypano_grayi                YAGVYFEKVVKTTASLAVRNIHLSLFGIPFAA--ISMFFLDIEQEQTFEYWRGYDQWLTLCLV
Emiliania2                   FASVYFEKMIKSDSSLWLRNLQLAMYSGVIAV--AGLLIND-DEIARQGPLHGFGF-WTWCAV
Guillardia                   FAGVFCEKMLKGGSNMSVRNIQLGVPGFVFGI--AGVLLTD-TKVTTGGFFQGYTY-LTWIVI
Sycon2                       FAGVYFEKILKGTKSLWLRNFQLAFFSILLGF--GGVVL-D-KVVLSEGFFHGYSP-LVWTVV
Caenorh_elegans              FAGVYFEKILKTSKSLWIRNIQLAFFSVFGAL--LVCWLYD-QAISDDGFLRGYNG-VIWIVV
Caenorh_remanei              FAGVYFEKILKTSKSLWIRNIQLAFFSVFGSL--FVCWLYD-QAISDDGFLRGYNK-IIWIVV
Monosiga                     FAGVYFEKILKGTKSLWLRNVQLGLFGMVLGL--IGVYAND-QAVAENGFFQNYDG-ITWTAI
Salpingoeca                  FAGVYFEKILKGTKSLWLRNIQLSLFSIVLGL--IGVVVND-DRVAEGGFFQYYST-VTWIAI
Amphimedon2                  FSGVYFEKMLKGSESIWIRNIQLGILGLVFGL--MAVFVTD-NKVMKDGFFQGYNI-VVWTVI
Human_UDPNAct                FAGVYFEKILKETKSVWIRNIQLGFFGSIFGL--MGVYIYD-ELVSKNGFFQGYNR-LTWIVV
Exaiptasia                   FAGVYFEKILKGTKSIWLRNIQLGSFGIIFGL--LGVLFND-KAVQANGFFQGYNY-ITWMVI
Acropora4                    FAGVYFEKILKGTKSIWMRNVQLGSFGVVFGL--MAVMTND-SQVSRGGFLQGYNR-ITWIVI
Sugiyamaella                 LAGIYFEKVLKGSKSLWTRNVQLSMFSLIPALF-LGVLAKD-SKIAENGFFHGYNN-VVWAAI
Tuber                        LAGVYFEKVLKGSNTLWVRNVQLSFYSLFPAFF-IGVVAKD-REILERGFFDGYNN-VVWSAI
Anthurium                    IAGVYFEKVLKNSQSVWIRNVQLSFFSLFPALI-MGVWWKD-SGVWENGFFYNYNY-VVVAAI
Entoamoeba                   FSGVYMEKILKNKTNIWERNIQLCVYGCGFAL--LSTFIFD-KSILDNGFFGGWSY-ITILLI
Emil_sc187_Ctg5084           LSSVYFERMLKKPAGLWLRNVQLQ--------------------LRSYGLLHGFDG-VVWLVV
Emilh_sc1_Ctg98              FANIYLEKKMKASESLWVRNVQLAVFGIPQAA--LPSPSAERAALAASGPFVGFTP-TVWSVV
Emliania1                    FAGVFLEKMFTSGGSLWMRNVQLGLFAIPLQC--IAIAQVD-QRVMRRGLVQGFHT-STWVVV
Bflori1                      FAGVYFEKLLKGSDSLWVRNVQMYTWGMLSAF--LGVVMHD-QNVRENGFLYGYTP-LVWLVV
SLC35A1_Callorhinchus        FAGVYFEKVLKSSDSLWMRNIQMYISGIVITA--AGILLND-AGVMEKGFFFGYTH-WVYLVI
Human_CMPSAt                 FAGVYFEKVLKSSDSLWVRNIQMYLSGIIVTL--AGVYLSD-AEIKEKGFFYGYTY-YVWFVI
SLC35A1_Danio                FAGVYFEKVLKSSDSLWVRNIQMYLSGIAVTL--MGVYMTD-ARVLEKGFFYGYTP-WVCLVV
Nematos_sc43                 FAGVFLEKIVKH-KSLWIMNVHLYSWGVCLGV--LGVVLKD-YQISQLGFFYGYDS-VVWTVV
Acropora2                    FAGVYMEKIFKGQKSFWVANAQLYAIGVILGL--VGVFYQD-GGIAKMGFFYGYDL-VVCLVI
Chrysochromulina2            FASVYLERILKGDKSIWVRNVQLCIFSIPLQL--LAVYRTP-DKVQTQGWMVGFCP-STWAVV
Aureococcus                  FSGVYQQRILQSCKDMWIRNVQMGVTSVTLGF--LCTFLKD-QAIADGGFFQGYSR-LVWVVV
Diplonema_LMZG01004873.1     GAGVYLEKLVKQTKSVWIRNIQLAVFGLIVGC--LGCFMKD-EAVAKGGFLQGFSP-IVWAVV
Guil_sc140_Ctg3497           FAGVYFEKMLKGTPSVWMRNIQMGTIGGILAL--AAVFIKD-QAVLSAGFFQGWNL-FVWGVV
Albugo                       FAGVYFEKILKHIDTIWERNVQMGIVSILLAS--LGLFWQD-EFLREFGFFYGYRL-VVWGAI
Nannochloropsis              FAGTYFEKVLKDSESVWVRNVELALIGIPVGV--FGVWYTD-AAVREAGFFSGYSP-LVWSVV
Chrysochromulina1            LAGVWLERIIKQTAPIWVRNIQLGVVSLALGL--GSVALLD-AAVAERGFFQGYTW-LTASVV
Phaeodactylum                FSGVYFEKILKGSRSLWIRNVQMGLSSIVIAY--LTVYVKD-EAIRTQGFWGGYNT-LVWTVV
Clunio_UDPGlut               FAGIYFEKMLKTSDSVWMRNIQLSLLSLPLSL--LMTMIND-TAISNQGFFFGYDL-FVWFLV
Ceratitis_UDPNAcT            FAGIYFEKILKGADSVWMRNVQLSLLSIPFGI--LTCFIND-RQISNVGFFHGYDL-FIWYLI
Apis_UDPGalt                 FAGIYFEKILKDSDSVWIRNIQLSLLSLPFGL--ITCFVND-EMLQKQGFFFGYDL-FICYLI
Exophiala                    FAGVYFEKVLKGGHSIWVRNIQLSVGCLAIAL--VGALMWD-QAIRQDGFFQGYNP-IVVVTI
Capronia                     FAGVYFEKVLKGGQSIWVRNIQLSVGCLGIAL--FGALVWD-KAIREGGFFQGYNA-VVVATV
Amphimedon1                  FAGVYVEKMIKGGGSLWMRNIQLSLFGSLTAV--LGMLMND-GAVMSLGFFYGYNF-LVFFVV
Sycon1                       FAGVYFEKILKGSASVWMRNIQLGVFGTVIGL--VGMLMND-TAVAKNGFFYGYTG-WVWFVI
Acropora1                    FAGVYFEKIVKGTRSLWARNFQLALFSIIIGT--GGMYIND-EKIRQKGILFGYHK-LVWLIV
Human_UDPgalt                FAGVYFEKILKGSSSVWLRNLQLGLFGTALGL--VGLWWAE-TAVATRGFFFGYTP-AVWGVV
Bflorid2                     FAGVFFEKVLKGSVSVWVRNIQLAFFSILLGL--ISMWTKD-AAVSEKGFFYAYNW-VTWMTI
Nematostella                 FAGVYFEKILKGTSSVWLRNVQLGAYSTVIGL--IGMQLND-AKIAEKGFFQGYSS-LVWSVI
Hydra1                       FAGVYFEKILKGSGSIWLRNIQLGIFGALIGA--VGMIAND-TKIKQNGLLFGYSA-IVWFVI
Acropora3                    FAGVYFEKIVKGSQSVWLRNIQLGIYGTIIGI--AGMYVKD-EKISDKGMLFGYST-LVWIVI
Desulfovibrio                mgnimmkklgggmkdgfrltiwmslvpivpnl--ilsfwte-ea-lshiswmgafa------v
Phaeomarinobacter            FGTILQRRIKG---GTFELQAWVATVALPFSL--AATFLFE-AL-MASADWIEWGG------I
Acinetobacter                MFTVLLRKYQLSAWAMSGVAIWSAVVYVPIYLLFLPKHLTD-HL-ISQTLFHGIFV----VII
Pontibacillus                MVSEKLVKEH-SSLVIAIVFTLSAFLLLPFLFVYDMSWVTE-----MVSLHLGIIA----TGL

Sycon3                       LNNASVGI------VTSLFLRSLNSILKTFASGLELVFTAILAWIF--F-GIRVGVSTWL
Hydra                        LNNGAIGI------VTAMFLKSLNSILKTFASALELMFTAILSWII--F-GIPVNFMTIV
Tribolium                    FNNAAIGI------VTSFFLKTLNSILKTFASALELVLTAILSYLF--F-RIAIHLNTVL
Drosophila                   VNNAAIGI------VTSFFLKYMNSILKTFASALELLFTAVLCYFL--F-SIPIYMNTAL
Bathycoccus1                 LNHSLSGI------AVSLVMKYADNIVKVYSTSVAMILTTLVSIPL--F-GFQLTLPFVL
Vitis                        VNHALSGI------AVSMVMKYADNIVKVYSTSVAMLLTALVSVFL--F-GFHLSLAFFL
Eleaeis                      LNHALSGI------AVSMVMKYADNIVKVYSTSVAMLLTAVVSVFL--F-GFHLSLAFFL
Zea                          LNHALSGI------AVSMVMKYANNIIKVYSTSVAMLLTATVSVFL--F-GFHLSLAFLL
Oryza                        LNHALSGI------AVSMVMKYADNIVKVYSTSVAMLLTAVVSVFL--F-GFHLSLAFFL
Brachypodium                 LNHALSGI------AVSVVMKYADNIVKVYSTSVAMLLTAIISVFL--F-GFHLSLAFFL
Bathycoccus4                 FNLAFTGL------LVSWIMKYADTIVKVYSTSMAMLVTMLFSIIL--F-DISPNLQLLL
Bathycoccus3                 INFSFSGL------FVSWLQKFADTIVKVYATSSAMLLTALLSVSF--F-GLEPSLQLFL
Bathy_ct342                  INFSFSGL------FVSWLQKFADTIVKVYATSSAMLLTALLSVSF--F-GLEPSLQLFL
Human_35A4                   LSQALNGL------LMSAVMKHGSSITRLFVVSCSLVVNAVLSAVL--L-RLQLTAAFFL
```

```
Human_35A5                    FVTAFQGL------SVAFILKFLDNMFHVLMAQVTTVIITTVSVLV--F-DFRPSLEFFL
Bathycoccus2                  ALHALGGI------LVALSVLYSSSVTKTVAVCAALVLTTVFGHIL--F-FEPLNGPILL
Reticulomyxa                  FLWSSGGI------LVALTIKYTDVIIKGFASAISLIVISVLGWLV--L-GDVLDLVFGI
Diplonema                     LVHAGGGI------LVAVVVKYADNILKGFATGVAIIVSGLYAAVA--W-GFAPSLHFIV
Bodo                          FVHAFGGL------LVAVVVKYADNILKGFATGVAVIVSGAFAALF--W-GYEPSMLFVV
Trypano_grayi                 FIHALGGL------LVAIAVKYADNIVKGFATGVAVVVSGVMSFSI--W-GVVPSMAFLW
Emiliania2                    LNNALGGL------LVAVIIKYADNILRSFAQGLAIVSGAVGSYLL--F-DFQITAQFML
Guillardia                    CLHSIGGL------LVTVIMKYADNIAKTIAIGISLVVSTAVSMY---IFDFVLTTNFCI
Sycon2                        ILQGIGGL------VVAAVIKYADNILKGFATSVSLIFSAVISFIF--LDDLELTSLFVI
Caenorh_elegans               LLQAYGGL------VIALVVKYADNILKGFAVSLSIILSSFTSWLV--LGDLTITTTFAI
Caenorh_remanei               LLQAYGGL------VIALVVKYADNILKGFAVSLSIILSSFTSWLV--LGDLTITTTFAI
Monosiga                      SLQAFGGL------IIAAVIKYADNILKGFANSISIILTGLISFIM--LADFQLTFMFAI
Salpingoeca                   SLQAFGGL------IIAAVIKFADNILKGFANSISIILTGLLSYLL--LGDVRFTMYFAV
Amphimedon2                   ALQALGGL------IVATVIKYADNILKGFATAVSIVVSSVLSYFF--LGDFDPTIKFGI
Human_UDPNAct                 VLQALGGL------VIAAVIKYADNILKGFATSLSIILSTLISYFW--LQDFVPTSVFFL
Exaiptasia                    FLQAFGGL------VVAAVVKYADNILKGFATSLSIILSSVVSYYL--LQDFNPSISFFV
Acropora4                     SLQAFGGL------VVAAVVKYADNILKGFATSLSIVVSSFVSFYF--LGDFQPTSFFFL
Sugiyamaella                  WLQAIGGI------VVALCVKFADNIAKNFATSISILISFVASVY---FFDFAVHVNFLI
Tuber                         GFQAFGGI------VVALCVNYADNIAKNFATSISILLSFIASIY---CFDFEVTIGFMI
Anthurium                     ACQAVGGI------IVAMVVKYADNILKGFATSISIILSFLASVY---LFEFIVTSTFLM
Entoamoeba                    IIQGVGGI------FVALVMTYADNIVKGFSIGCAIVLTTICSIFI--F-GTQVDTTFII
Emil_sc187_Ctg5084            LLNGCGGL------LVAATMKYADNIVKCFAAALAIITGMLLSVPL--F-NVPLSPIFLV
Emilh_sc1_Ctg98               WLRALGGL------LVAAVIKYADNIVKTYATAVAIVLTCVVSSVL--D-GSRPTRAFLQ
Emliania1                     AVQVVGAL------CTAVVIKFAGNVLKTFATVLALLLTCGSSMVL--F-DFHPTQLFFE
Bflori1                       LLGSGGGI------YTSIVVKYTDNIMKGFAAAAAIVLSTVASIMF--M-GLVVGWMFVL
SLC35A1_Callorhinchus         FLASVGGL------YTSVVVKYTDNIMKGFSAAAAIVISTIASIFL--F-GLQITSTFIA
Human_CMPSAt                  FLASVGGL------YTSVVVKYTDNIMKGFSAAAAIVLSTIASVML--F-GLQITLTFAL
SLC35A1_Danio                 FLASVGGM------YTSVVVKYTDNIMKGFSAAAAIVLSTVASVLL--F-GLQITLTFIS
Nematos_sc43                  ALASAGGI------LVSLVLKYASTITKGFATSCAIVLSSLASVII--F-GFDPSIYFIL
Acropora2                     LFASAGGI------IVSLTLKYASCITKGFATSFAIVLSSFVSAYV--F-NFIPSMQFIV
Chrysochromulina2             FMFAFGGL------LVAIVIRFADNNLKNLAMAVAILVSCVASIPL--F-GFEPNGTFAA
Aureococcus                   SLQALGGL------NVAFILKYADNILKGFAAAFSTIASCIIEMVL--F-QFRPSPLFLF
Diplonema_LMZG01004873.1      LLQSLGGI------LIAVVVKYADNIIKGFATGLSIICSCLASVFL--F-NFAVTPQYLA
Guil_sc140_Ctg3497            T--QLGGL------ILPLVVRYPNNILKGFATSLVRFKTPSS------------------
Albugo                        TISAAGGL------LTAIVVKYADNILKAFATSIATVLSVLMSILL--F-NKIPTAQFAL
Nannochloropsis               GLQAVGGI------AIALVVKYADSVLKNFSTSVSIVVSCLVSYVV--FGETDLSPQFLA
Chrysochromulina1             LQVSAGGL------LVGLIMKYADNLLKGFATSLSIILSSVVSTFIPAF-GFEPGLAFFC
Phaeodactylum                 TVQAVGGL------IVATVVKYADNVLKVFATSFSIVVSCIVSAFL--F-DFHPSVSFLV
Clunio_UDPGlut                LLQACGGL------IVAVVVKYADNILKGFATSLAIVLSCIVSIYL--F-SFLLTLQFAF
Ceratitis_UDPNAcT             FLQAGGGL------IVAVVVKYADNILKGFATSLAIVVSCVASIYI--F-EFHLTIRFAV
Apis_UDPGalt                  ILQAGGGL------IVAMVVKYADNILKGFATSLAIIISCVASIYL--F-NFNLSFQFSI
Exophiala                     CIQAAGGL------IVAMVIKYADNILKGFATSLSIILSTVASVFL--F-NFMPTIYFLL
Capronia                      CIQAAGGL------IVAMVIKYADNILKGFATSVSIILSTVASVFL--F-NFVPTVYFLL
Amphimedon1                   FQQALGGL------IVSVVMKYADNILKGFSTSLSIIISCVVSVFV--F-SFVISTYFVI
Sycon1                        FMQAFGGL------LVAVVVKYADNILKGFATSLAIILSTVASLYL--FPDFRVSFTFLI
Acropora1                     GLQAFGGI------LVGFVVKYTDNILKGFSAAISIVVSCIASVYL--F-QFKVSIQFVT
Human_UDPgalt                 LNQAFGGL------LVAVVVKYADNILKGFATSLSIVLSTVASIRL--F-GFHVDPLFAL
Bflorid2                      CMQAFGGL------LVAVVVKYADNILKGFATSFSIILSCIASVYL--F-SFHITLQFAF
Nematostella                  CMQAFGGL------LVAVVVKYADNILKGFATSFSIVLSCIVSIYL--F-AFHASLQFVV
Hydra1                        FMQAFGGL------LVAVVVKYADNILKGFATSFAILVSCIVSIYA--F-NFVLSLEFVA
Acropora3                     FMQAFGGL------LVAVVVKYADNILKGFATSFAIILSCVVSVYL--F-SFQLSSQFLF
Desulfovibrio                 lytawgstiigwgawawllrkyaapqvapf-sllvpvvtlviavsl--g-meplkpetgm
Phaeomarinobacter             IYVAFASSLVGHAGIYWLLQRYEVSQTAPY-TLLAPLFTVTFGVWL--L-GDMLTERMIF
Acinetobacter                 ATLTYA----------EAIKRLGAFKAGSI-TNLAPFIAALLAVPL--L-GESLSIAMIC
Pontibacillus                 AYFLFA----------RGLIHTPSSTAVTL-SLAEPLTAALLGVFL--V-GERLELLSWM
```

2 - MSA Sialidases

```
Sialidase_aspergillus
Reticulomyxa_ETO10819.1            -----T---DTNINMNM----------NMSMNVKSSSI----KAKVNPP--
Bathycoccus                        GLASVA-IV-VDDFWTVSS---------TIGND---IT--KAS-------S
Bathycoccus_XP_007512415.1         ELPSQT-TLSKQVF--------------DSGGGGGSSVIIKHSTYVKRFDE
Aureococcus                        AA--ND-NVLSVPF--------------VSGGDGYACT--KIPSLLRLASG
Monosiga4                          AVYARV-SCLRTVF--------------EGGKEGYFCY--RIPSLVQLGPD
Monosiga2                          AVPVPP-VGPSVVF--------------AKGEAGYYCI--KIPDVVHFPNS
Bodo                               IAEPSP-FHEVDVF--------------TIGEAGYFCI--KIPYLFTTFNG
Karenia1                           TAASTK-VGLVNVF--------------SKGESGYYCI--KIPYLTSLPDN
Emiliania                          LTPPPD-LTSTDLFTPGMP---------DTAGVTYACY--RIPSMVWVANE
Chrysochromulina1                  ISA--A-PTMFDVFHRQQV---------DSANVSYFCF--RIPALVRRAD-
NEU2_HSA                           ASLPVL-QKES-VF--Q----------S-----GAHAY--RIPALLYLPGQ
NEU3_HSA                           EEVTTCSFNSPLFRQED----------D-----RGITY--RIPALLYIPPT
NEU4_HSA                           MGVPRT-PSRTVLFERE----------R-----TGLTY--RVPSLLPVPPG
Saccoglossus5                      ---------------------------F-----NCASY--RIPAIVYNKG-
Saccoglossus6                      M-----SEVMDVFTGNE----------K-----GFNVT--RVPGMLYHDG-
Trypanosoma_grayi                  HALGVN-SRRVELFARGKSTVPFEQDDGEVVPRVAHSF--RIPSLVEVD-G
Oscarella2                         NPKPH--HDPQYVWTPN-----GA---D-----QMGVY--RIPLITAAPNG
Oscarella1                         FLNPFV-EEEFILWNKR-----GD---G-----DVDHF--RIPIATWNERG
Saccoglossus1                      VTPK-V-VQEKLIWIKG-----Q----E-----EVSLY--RIPIISYTSQG
Saccoglossus2                      VTPK-V-VQEKLIWIKG-----Q----E-----EVSLY--RIPIISYTSQG
NEU1_HSA                           VQPLVT--MEQLLWVSG-----RQ--IG-----SVDTF--RIPLITATPRG
Orbicella                          ITANYA-VNAFSIIFLG-----D----I-----GVNTY--RIPIITSLPDG
Nematostella                       SEEFGS-LNEVVLWKRG-----D----H-----GVSDY--RIPLITSLPDG
Saccoglossus3                      SLSPLI-VDDQVLWTSG-----DE---G-----DISFY--RTPMLINTPNG
Saccoglossus4                      VLSPVV-IEEQYLFQPG-----NP---G-----EVDAY--RIPISIMCPDR
Monosiga5                          SNWQSL-VNKTDLFVNN-----------QPYGITYPCY--RQPALILMGEG
Metarhizium_XP_007806611.1         KDVPPA-HKEFVLFKSSNMAGAD----KLANGVGFHSF--RVPAVVTTSTG
Aspergillus_XP_751452.2            KSAAPY-HDEFPLFRSANMASPD----KLSTGIGFHSF--RIPAVVRTTTG
Arthroderma_XP_002847861.1         GKAAQY-HKEYALFKSENMPSPD----KLSSGVGFHSF--RIPAVIRTNTG
Emiliania_XP_005775390.1           PNPCTA-AECTPIFYPG-----L----N-----GSQCY--RIPSIIATHRG
Chrysochromulina3                  -EPRR--GDPVAVFWAG-----Y----N-----DTACF--RIPTVITSHTG
Chrysochromulina2                  LSLCF--NDVTTVFSTG-----M----A-----GEHCY--RIPTIIRLEQG
Rhodanobacter                      QAHVPL-FQRITLARPG-----D----L-----DVHTY--RIPALAVAKDG
Arthrobacter_OOP63695.1            SNPPGS-YVEQVLASNG-----D----NAIDPVLGKYY--RIVALADLGDG
Sporothrix                         HRSSDL-ITNTTLATAS-----S----G-----PFNYY--RIVSLQSLGNG
Sporothrix_KIH89835.1              HRSSDL-ITNTTLATAS-----S----G-----PFNYY--RIVSLQSLGNG
Pseudogymnoascus                   INSREL-FKEQVLATNG-----V----G-----PSPYY--RIVALANLGNG
Penicillium                        ---AAI-TSQATLAKDG-----A----G-----PFAHY--RIVALANLGNG
Blastocystis                       DGCNPR-VKSVELFKPW-----E----D-----GFPCI--RVPSIIRT--P
Monosiga1                          ADCDVN-RTQIAVFTPG-----E----L-----GYPCI--RIPSILLAGDN
Monosiga3                          ADAAIQ-VDQVDVFIGG-----T----R-----GHACY--RVPTAVTLPNN
Karenia2                           TCDSRA-DGSVTVFGAG-----E----A-----NISCF--RIPAIVRTVKG
Endozoicomonas                     EYHPDL-TVVNDVFVGG-----E----N-----GYAIY--RIPGMLIIPAG
Chrysochromulina4                  ASAPPS-ITIVDMFTPG-----M----L-----RVNVF--RIPSLLPLPSG
Psychromonas                       SGEERP-LREVVVYQPG-----T----E-----GYACF--RIPTIVKAGNG
Janthinobacterium                  PVAQAA-ISASTPFPAA-----D----A-----SYGCY--RIPAVVTLQNG
Pedobacter                         IATSRV-DSLNFIFKAG-----E----N-----GYACF--RIPALIRTQNG
Rickettsiales                      PTFPIS-ENTKYIFKSG-----S----D-----GYNTF--RIPTTITTNSG
Kriegella_WP_089893641.1           NNPIES-KELNYIFEGN-----Q----E-----GYECF--RIPAVVKTNKG
Sphingobacteriales_OJY82384.1      METLQS-QELNYLFKQD-----E----S-----GYKCF--RIPAIVSTNNG

Reticulomyxa_ETO10819.1            -------------------------MA-------TTMMKPTTT-----------------
Bathycoccus                        ----SSF----K---ASSHK--YAFT-------------PTNDK-----M-------EYA
Bathycoccus_XP_007512415.1         ----LR----------------YAHMA--------TIAKMKDER-----M-------ILL
Aureococcus                        --AFLAFAEARW---PDCGD--FSRTD--------LVAKRSEDG-------------GKT
Monosiga4                          --SYIAFAEGRK---FSCED--HGWND--------IVAKTSSDG-------------GQT
Monosiga2                          -STLLAFGEARR---DSCSD--YTATE--------LVMKRSEDL-------------GRT
Bodo                               --TMLAFGEGRK---ESCSD--FAPTD--------LVFKVSEDA-------------GAT
Karenia1                           --SLLALGEARW---GSCSD--YTKTD--------LVMKTSWNG-------------GKT
Emiliania                          HAVLLAFAEGRR---GSCAD--KGDVR--------IVARRSSDG-------------GQT
Chrysochromulina1                  -DALIAFAEGRH---VNCDD--AGDVR--------IVRRISYDE-------------GET
NEU2_HSA                           -QSLLAFAEQRA----SKKD--EHAEL--------IVLRRGDYDAPT--H-------QVQ
NEU3_HSA                           -HTFLAFAEKRS----TRRD--EDALH--------LVLRRGLRI--G--Q-------LVQ
NEU4_HSA                           -PTLLAFVEQRL----SPDD--SHAHR--------LVLRRGTLA--G--G-------SVR
Saccoglossus5                      --VFLAFCEARR---TSFRD--YGEMD--------LVLRRGILL--E--K-------QVE
Saccoglossus6                      --QFLVFCEARA----RLAD--NGKMA--------IVSKRGLLQ-PD--N-------SVQ
Trypanosoma_grayi                  --VLVAIGDARY---LSDDD--NAFIE--------TAVKFSVD------G-------GST
Oscarella2                         --NLIALTEGRK---IGGGD--TGYKF--------LSQRSISS------QVVVSTQGDQK
Oscarella1                         --AIVAVSEARK---NTSAD--RSAKF--------LAVRRMAH------S----VWSKDA
Saccoglossus1                      --SLIAASEARK---YSGSD--HGPKF--------LAVKRSTD------K-------GYT
Saccoglossus2                      --SLIAASEARK---YSGSD--HGPKF--------LAVKRSTD------K-------GYT
NEU1_HSA                           --TLLAFAEARK---MSSSD--EGAKF--------IALRRSMD------Q-------GST
Orbicella                          --SLIAMSEGRK---HSSAD--SGPKF--------LAMRRSKD------Q-------GIT
Nematostella                       --SLIALAEARK---HSSSD--SGPKF--------LAMRQSRD------Q-------GVT
Saccoglossus3                      --DLLAFAGARK---YSEGD--TAQKI--------ATMRRSRD------K-------GAS
Saccoglossus4                      --SILAFAEGRK---YGRGD--YGSKF--------LAMRRSKD------S-------GDT
Monosiga5                          ---LLAFAEGRNISS--CAPPLQAAAPSPVEEVGGLVLRRSTD------L-------GRT
```

```
Metarhizium_XP_007806611.1         --RVLAFAEGRR---HDNRD--LGDVK--------VVMKRTKTVNSHGSN-------PSD
Aspergillus_XP_751452.2            --RILAFAEGRR---HTNQD--FGDIN--------LVYKRTKTTANNGAS-------PSD
Arthroderma_XP_002847861.1         --RILAFAEGRR---HNNRD--YGDIN--------LVYKRTKTPTNNGEN-------PSD
Emiliania_XP_005775390.1           --TLLAFAENRL---GGCGD--QGMHN--------LVVRRSSD------N-------GAT
Chrysochromulina3                  --TLLAFAEARL---TSCSD--DTDHK--------LVLRRSLD------G-------GTT
Chrysochromulina2                  --PLLAFAEQRD---PGCGD--GGSNN--------LVMRRSTD------D-------GQT
Rhodanobacter                      --TLLASYDARI---DAAHD-LPGNID--------VMLRRSRD------M-------GRS
Arthrobacter_OOP63695.1            --VLLASYDGRP---DGG-D-SPSPNS--------IVQRRSTD------G-------GKT
Sporothrix                         --VVLAAFDGRP---TGA-D-APAPNS--------ILQRRSTD------Y-------GAT
Sporothrix_KIH89835.1              --VVLAAFDGRP---TGA-D-APAPNS--------ILQRRSTD------Y-------GAT
Pseudogymnoascus                   --VVLAAYDGRP---DGG-D-SPSPNS--------ILQRRSTD------G-------GET
Penicillium                        --ILLASYDGRP---DGG-D-SPSPNS--------IMQRRSTD------G-------GKT
Blastocystis                       -HALIAYAECRMITGDGCNP--TPIHKILNDNTMYVCQKKSVD------G-------GKT
Monosiga1                          -RTLLAFAECRNWTGDGCEP--QGFEDARYNSTSNAN---RDL------C-------MKT
Monosiga3                          --TVLMAVESRR---TTCGD--QAPKD--------IELVRSQD------G-------GFT
Karenia2                           --TLLAFAEARH---GGCGD--GQVHQ--------IAVRRSID------Q-------GKT
Endozoicomonas                     SDRLLAIAEARR---DGALD--NGVID--------LVLKISDD------G-------GRS
Chrysochromulina4                  --VVLSFAEARP---Q-LVD--HGVIN--------IVMRRSTD------A-------GRT
Psychromonas                       --DLLAFAEGRV---DNCGD--HGNIN--------VVLKRSSD------D-------GIT
Janthinobacterium                  --TILAFAEGRP---SGCAD--FGNIQ--------VVMKKSLD------G-------GST
Pedobacter                         --SLLAFAEARK---NNCGD--SGDID--------LVVKRSLD------K-------GKT
Rickettsiales                      --VVLAFAEGRK---NSSSD--TGDID--------IVLKRSLD------G-------GKT
Kriegella_WP_089893641.1           --TLLAFAEGRK---NGCSD--TGNID--------LVLKRSVD------H-------GKT
Sphingobacteriales_OJY82384.1      --TLLAFAEARK---NSCSD--TGDID--------LVLKRSED------G-------GKS

Reticulomyxa_ETO10819.1            ---NSKVKPNGI-----G------NGNGNGNVSAERKR--------------
Bathycoccus                        WAVKGRALPSE----------TRDAKLGTTEPEFKYTHMADGSIAVAF-QAS-
Bathycoccus_XP_007512415.1         YQAAPAAKKDVDGSDGGGGDDGNGGGDGGDATYYEYK------LATEG-L---
Aureococcus                        WSAAQVVAPAAPGTR---GLCGHEVVVGNAAPVQATKG--AGRVLVPH-T---
Monosiga4                          WSALAVVYGE--------SSTKANITIGNPAPVVLH----DGTLLLVF-C---
Monosiga2                          WGNLTVVHAN--------GA----HVVGNAAPVLLP----SGRLLLPH-C---
Bodo                               WGPLRVLHTN--------GS----NTVGNAAPLQLR----NGRILIPF-C---
Karenia1                           WSALKIFHTG--------GE---ADTVGNAAPVVV-----GQDLIIPF-C---
Emiliania                          WSSIEQVAVE------------AGHTIGNPAPVADL----AGSVHLVH-A---
Chrysochromulina1                  WSDIAQVKVE------------PGHTIGNPCPIVDL----TGHIHLLY-S---
NEU2_HSA                           WQAQEVVAQARL----------DGHRSMNPCPLYDAQT---GTLFLFFIAI--
NEU3_HSA                           WGPLKPLMEATL----------PGHRTMNPCPVWEQKS---GCVFLFFICV--
NEU4_HSA                           WGALHVLGTAAL----------AEHRSMNPCPVHDAGT---GTVFLFFIAV--
Saccoglossus5                      WEGIQVIA--SM----------RGERTMNPVPIVDNVN---KAVVVVFNTF--
Saccoglossus6                      WEESKIVV--QV----------AGLRTSNPCPVYDRMR---NTVILVFLAV--
Trypanosoma_grayi                  WETQIAIKNSRVNS--------ERSRVLDPTVIVKG-N----KIYVLVGSYNI
Oscarella2                         WSPTIEEIDNGYT---------MSDGVILGAVVTDNVT---KTMFVIY-GEC-
Oscarella1                         WSPTMFIENDGF----------ASDGVNLGAVLSTT-N---GTTIVVY-GTC-
Saccoglossus1                      WEPQQFVSDDGSI---------VKDGLNLGAILSDDEK---GIMFIFY-TMC-
Saccoglossus2                      WEPQQFVSDDGSI---------VKDGLNLGAILSDDEK---GIMFIFY-TMC-
NEU1_HSA                           WSPTAFIVNDGD----------VPDGLNLGAVVSDVET---GVVFLFY-SLC-
Orbicella                          WSNTTFIEDDGE----------DPDGLNLGTVFVDEDA---NRVFVVY-SYC-
Nematostella                       WTNTTFIEDDQE----------ATDGLNLGSIVVDEIT---KSVFVMY-SFC-
Saccoglossus3                      WDPTVFVIQDLGP---------ETDYFNFGTITVDDAV---NSLVFLY-CHC-
Saccoglossus4                      WTTTEFVLDD-NP---------SVDGLNLGNVIIDYYN---NSVIIVY-VHA-
Monosiga5                          WSAPITLYSGNID---------------FYVTVYDAEK---DVGWLFL-QE--
Metarhizium_XP_007806611.1         WESLQV-VT------------KDDGVWGNPTPVVD-GP----TIYLFL-SWNN
Aspergillus_XP_751452.2            WEPLREVVG------------SGAGTWGNPTPVVDDDN----TIYLFL-SWNG
Arthroderma_XP_002847861.1         WESLREVVG------------TGPHTWGNPTPVVD-GN----TIYLFL-SMND
Emiliania_XP_005775390.1           WGPLITVFAGVVPCPGCP------AAVSNPNPVEVTFPNG-TRSILLA-FDT-
Chrysochromulina3                  WGDLIVAVDGIVPCEGCP------ASVSNANPVAVWLQGG-RAAILLH-FNT-
Chrysochromulina2                  WGDLITIAA------GGG------APFSNPNPVEVDLGDH-KLAVLLH-YDT-
Rhodanobacter                      WSAPQRVVHYG-----------DGIGAGDSSLLVDRDT---GRIFLFY-AYAP
Arthrobacter_OOP63695.1            WGSPTFIARGQLSS--AG---VQKYGFSDPSYVVDKVT---GTVFNFH-VYS-
Sporothrix                         WGSLTYIAEGQAANAGTG---ALAYGFSDPSYVYDSAT---STLFNFH-VFS-
Sporothrix_KIH89835.1              WGSLTYIAEGQAANAGTG---ALAYGFSDPSYVYDSAT---STLFNFH-VFS-
Pseudogymnoascus                   WGAPTYISQGQLGS--TG---VQKYGFSDPSYIVDQDT---GKIFNFH-VFS-
Penicillium                        WGEPTYVAKGQPKS--SS---AEQYGFSDPSYVVDSKT---GKVFNFH-VFS-
Blastocystis                       WGNLTYPFG-------------REIATREASPVYDFIN----DKLVVV-A-N-
Monosiga1                          WSALHVIQ----------------RNAAQPMPVWDYQQ----NQLVLN-FVQ-
Monosiga3                          WSEPVMLVGSL----------TGNTTYRNPYLTVVPGS--TPDLVLLQ-FVN-
Karenia2                           WSELTFAAG------------SDQLFVGNPYPIAMSGG----DV-ALV-FVN-
Endozoicomonas                     WNEQQVVCR--YE---VG---DARGKCGNPTPVFEQTN---GIVFLAH-----
Chrysochromulina4                  WGLARVCVEGAMV---GG---AAKFTVGNPTAVYDKTT---KLIWLLF-CTN-
Psychromonas                       WGPLTVIAE------------FGQLAAQNMTAVYDEYYPHFGR--LMV-MWN-
Janthinobacterium                  WSALSVVAS------------NGTLQAGNPVPVLDTLDPAGGRLFL---FYN-
Pedobacter                         WTGLQLVWS------------DSTNTCGNPVPIQDQTT---GNIILIS-TWN-
Rickettsiales                      WGKLIVVRD------------DEDNVCGNPSPVVDSKT---GKIFLLS-TWN-
Kriegella_WP_089893641.1           WSNLQVVWD------------DKDNTCGNPSPVVDVST---GTIFLLS-TWN-
Sphingobacteriales_OJY82384.1      WSALEVVWS------------DGENTCGNPAPVVDKTN---GNIILLS-TWN-

Reticulomyxa_ETO10819.1            -----------------------ASNNGKDPVKQMDSSLERLTWDT
Bathycoccus                        -----P---------------TEYEGSIRQALYWAISKDDGLTFST
Bathycoccus_XP_007512415.1         -----------------------QDQH---IEYTQSKDLEGKRWTP
Aureococcus                        -----------------------RDNF---AVWSVHSDDDGRTWSA
Monosiga4                          -----------------------RNNQ---AMLLLNSTDGGQTWGG
Monosiga2                          -----------------------LDNK---VVYTMHSDDQGMSWST
Bodo                               -----------------------INNL---YVYQMHSDDNGHTWSI
Karenia1                           -----------------------LNNS---KVFIKRSGDLGETWSN
Emiliania                          -----------------------RDDTQALAVFLVSSADGGRSWSE
Chrysochromulina1                  -----------------------RDNK---EVFVTRSTDGGESWGA
NEU2_HSA                           --PGQVTEQQ-----------QLQTRANVTRLCQVTSTDHGRTWSS
NEU3_HSA                           --RGHVTERQ-----------QIVSGRNAARLCFIYSQDAGCSWSE
```

```
NEU4_HSA                         --LGHTPEAV-----------QIATGRNAARLCCVASRDAGLSWGS
Saccoglossus5                    --PSNVTETD-----------LLKEGVFNQTVFVMKSTDNGITWSD
Saccoglossus6                    --DPRIGEKY-----------LMKMGQHQVTVAVVKSRDFGKTWSD
Trypanosoma_grayi                TWQGDGSDWEPLLAVGE-----------VNKTTVGGKPNATITWGK
Oscarella2                       ------------E--------HA--C-PTTRTYVINSTDNGLSWSD
Oscarella1                       ------------M--------HT--C-PKTRTFTMTSHDDGLTWGE
Saccoglossus1                    ------------A--------HYDHC-DTSTTHVIRSYDDGVTWTN
Saccoglossus2                    ------------A--------HYDHC-DTSTTHVIRSYDDGVTWTN
NEU1_HSA                         ------------A--------HKAGC-QVASTMLVWSKDDGVSWST
Orbicella                        ------------A--------HK--C-VYHTTYLISSDDFGDTWSK
Nematostella                     ------------I--------HK--C-KYATTYVIRSDDNGQSWTM
Saccoglossus3                    ------------P--------HNVCKDSLPTVFMMRSYDWGYTWSK
Saccoglossus4                    ------------H--------------THSELYMVKSLDWGVSWGK
Monosiga5                        ----------------------------ESAVRVFNTTDQGYTWAG
Metarhizium_XP_007806611.1       AKYSRDGEDR--LPNGKKTKKVDGSWKSRRHTYLTQSTDDGQTWSE
Aspergillus_XP_751452.2          ATYSQNGKDV--LPDGTVTKKIDSTWEGRRHLYLTESRDDGNTWSK
Arthroderma_XP_002847861.1       GAYSQNGNNV--LLNGEKTKKIDSTWVGRRHLYLTESIDDGNSWSK
Emiliania_XP_005775390.1         ------------M--------NNPSVAHHGLDMTMWSHDDGVSWVG
Chrysochromulina3                ------------L--------NNPTQLVHGVAMQIWSFDDGLTWEG
Chrysochromulina2                ------------M--------NNPTSAHHGSNMQIWSYDGGATWVN
Rhodanobacter                    RGIGFATSQP--G--------NTTDSSTTLHPRYVWSDDNGASWQG
Arthrobacter_OOP63695.1          KNQGFAGSVL--G--------NDDSDPNVISSQVSVSTDRGLTWST
Sporothrix                       KDASFASSVL--G--------HDDTSRNITSAEVSVSTDGGLSWST
Sporothrix_KIH89835.1            KDASFASSVL--G--------HDDTSRNITSAEVSVSTDGGLSWST
Pseudogymnoascus                 KNVGFGSSVI--G--------NDDANLNIISSQVSVSTDGGVSWST
Penicillium                      KNQGFLGSKV--G--------NDDTDRDIVSAEVSVSTDNGVSWST
Blastocystis                     --LMDSQ------------------------LIMTVSEDEGDSYSP
Monosiga1                        --LTPGD------------------------NLQSMSKDFGQTWTP
Monosiga3                        --STLDEPWT---------------------TFQMESRDAGLTWSS
Karenia2                         --HTHGHGAD--L--------GSG-------NGVIFSRDDGITWSA
Endozoicomonas                   ----------------------NISGIDGMTPVMISSPDGGSSWSE
Chrysochromulina4                --HAADAEWM--I--------HAREGRDTRRVWITSSADEGLHWTV
Psychromonas                     --NADGGEAD--V--------SDDDHPAQRWVLYRTSLDHGVTWSE
Janthinobacterium                --TGNASEST--I--------RN-GGAGVREQWVVTSTDGGATWST
Pedobacter                       --LGTDHEKQ--I--------MDGSSKDGRHVYMLSSGDNGKTWSA
Rickettsiales                    --RGDDSEKE--I--------IDLISNDTRRVYIMNSEDEGLNWTV
Kriegella_WP_089893641.1         --LGTDHESE--I--------IKQTSQDTRRVFVLKSKNDGKRWSK
Sphingobacteriales_OJY82384.1    --LGTDHEKD--I--------IALKSQDKRRVFMLKSTDDGKTWLP

Reticulomyxa_ETO10819.1           MDSSVLECSVVTQLSNHWPSFSFGPDETTESDSL--SHHLDNGTIIQRKNVS---
Bathycoccus                       PKMIAKD-DQ-----DQSRNALRHSPG---------------GDVM------YKV
Bathycoccus_XP_007512415.1        PVKLPIY-ND-CHHTPKSLWVPG--------------------GDI------KYIK
Aureococcus                       PRALPGL-ADGSKLAGPWQFLGLGPPGALELDD---------GRLIAPGYHAYIR
Monosiga4                         FRDVSAG-----IVDPTWPFFATGPPGSIQLHT---------GRILVPADHTSGS
Monosiga2                         PTLVS-N-----AVRDDWRWIGLGPPGGLY-NR---------GRVVIPAYHDTS-
Bodo                              PEFIP-G-----ATQPDWQWVGTGPPGGLQLAS---------GRLVVPSYHSYVA
Karenia1                          AEDISSN-----VTRAHWKWVGLGPPAGLLLSS---------GRILIPGYHTDIF
Emiliania                         RRNLTAA-LK-ANPAPE-AFVMPGPPGGVQLQS---------GRLVVGMYGEDEA
Chrysochromulina1                 STNLTAS-LK-LELDPESIFVATGPPGGVQLAS---------GRLVGGFYYNG-L
NEU2_HSA                          PRDLTDA-AIGPA-YREWSTFAVGPGHCLQLHDR-------ARSLVVPAYAYRKL
NEU3_HSA                          VRDLTEE-VIGSE-LKHWATFAVGPGHGIQLQS---------GRLVIPAYTYYIP
NEU4_HSA                          ARDLTEE-AIGGA-VQDWATFAVGPGHGVQLPS---------GRLLVPAYTYRVD
Saccoglossus5                     PQDITDT-TLGKV-EPAWTIFSPGPGHGIQLSS---------GRLIVPGNYYVKD
Saccoglossus6                     PIDITGT-TIDTIQNPPVAMYAPGPGHGIQLES---------GRILIAGNHYQKD
Trypanosoma_grayi                 PVSLKSI-FP----GAPSAQFLGGVSRAIVLNDK---------TLVFPVQATNE-
Oscarella2                        PVDITHH-------GTNKTVFSPGPGTGLQK-----MHAPHKGRLVFCGHYHDII
Oscarella1                        PVDITEQ-------GGVYEIFAGGPGYGIQK-----RHDPHQGRLIFCGWNCTAP
Saccoglossus1                     SFNVSEQ-------GT--RMFAPGPGYGIQK-----KLNPNKGRLVVCGHSTL--
Saccoglossus2                     SFNVSEQ-------GT--RMFAPGPGYGIQK-----KLPPHVGRLVVCGHTS---
NEU1_HSA                          PRNLSLD-------GT--EVFAPGPGSGIQK-----QREPRKGRLIVCGHGTL--
Orbicella                         PSNLSDQ-------GT--SLFAPGPGFGIQK-----KQDPHRGRLITCGHASP--
Nematostella                      PKNISQQ-------GG--LQFAPGPGYGIQVECSSPALDPWKGRLIICGHSIG--
Saccoglossus3                     PVNLSIS-NP----YN--FAYCPGPGYGIQK-----KIGPYKGRLISCGHTIK--
Saccoglossus4                     AIALSDR-NP----KG--YEWTAGPGYGIQL-----KYGPHKGRLLSCGHGMS--
Monosiga5                         PFAFTAP-VP-----GLRPTVQPAVGHGLQLQRHL-GGCAEAGTLVVPFVCMSDT
Metarhizium_XP_007806611.1        PREMT----------GGRGWDVVGPGNGIVLTSGE---------VVVPAMGRNIV
Aspergillus_XP_751452.2           PVDLT----------DGWAWDAVGPGNGIRLTTGE---------LVIPAMGRNII
Arthroderma_XP_002847861.1        PVDMT----------DGQAWDAVGPGNGIKLTSGE---------LVIPAQGRNII
Emiliania_XP_005775390.1          ARA-TEY-PP-----QPNVGSLIGPAVGLQLQAPE-----ARGQIVFWITSGFGF
Chrysochromulina3                 ASV-LTY-PP-----VHNIGGLIGPSVGLQADDG----------TIFFSARQPE-
Chrysochromulina2                 STNISAF-MP-----EGYAGCMPGPSVGVQSMDG----------TIYFSCHGFG-
Rhodanobacter                     PRDLT---------SPTWKGMFATSGHGLQLFNDQ----GARGRLIQPYVFRG--
Arthrobacter_OOP63695.1           DPGNQPT-LPGVPNVGGVVSMFASSGEGIQLKYGA-----HAGRLIQQFAGTIVQ
Sporothrix                        DPTNQPN-LPSVSDVGAVLGEFAASGQGIQLQYGA-----HAGRLIQQYLGRVVQ
Sporothrix_KIH89835.1             DPTNQPN-LPSVSDVGAVLGEFAASGQGIQLQYGA-----HAGRLIQQYLGRVVQ
Pseudogymnoascus                  DPKNQPS-LPGVASVGGVVGQFAASGEGIQLRYGA-----HAGRLIQQYLGKVIQ
Penicillium                       DPENQPK-LPGVKNVGGIAGLFASSGQGIQLKYGA-----HAGRLVQQFLGRVVQ
Blastocystis                      MENIE------------AHTTYVGVGHGLQIQGG-----PHRGRLIFIGHHGAYE
Monosiga1                         RGTC-------------------------PSLDGSP-GHAVAPNRLLFIGHHGAYQ
Monosiga3                         AQPVN----------SAWNGTLAGPGLGITLAHTH------PGRIIMCGASGYHA
Karenia2                          PLDIS------------ATGSLPGPGAGIELGASH--------RMLVVSHHGAYV
Endozoicomonas                    RIVLP-------------DDLIFGPGHGIQKSKAP-----AIGRIVVPGYQVQKG
Chrysochromulina4                 PAEIT----------PGWTWYATGPGAGIQLTSG---------RLLIPSNHAGDV
Psychromonas                      PTDIT----------PGQKMHIPPTGHAIQLNTQE-AEAGRFGRLFFSGQYNPVG
Janthinobacterium                 PSNIT-----PYNNGAGWRTLAMGPGHGVQMRSG---------RIVVPGNFTAGP
Pedobacter                        ATEIT----------TGWTWYATGPCHGLQVLKGK-----YAGRLVVPINHVETG
Rickettsiales                     PKEIT----------SNWTWYATGPVHGIQIQKGK-----KSGRMIVPCDHIEAE
Kriegella_WP_089893641.1          PEDIT----------PNWTWYATGPGSGIQITGGK-----YKNRLMIASDHIEAK
```

```
Sphingobacteriales_OJY82384.1      AREIT----------EDWTWYATGPVNGIQVQKGK-----YKGRLVIPCDHIEAV

Reticulomyxa_ETO10819.1            AFVLYSSQHNANIAVWNKDMV
Bathycoccus                        GQVVLSYGSEDGMPKVIANTL
Bathycoccus_XP_007512415.1         AVTILSQDEGGGIPKVIANSL
Aureococcus                        GHVVYSDDGG---DSWALASR
Monosiga4                          SMAIYSDDYG---QTWTRGAM
Monosiga2                          PHTLLSDDEG---ATWRIGAT
Bodo                               IHMMINDDPSGNASSWFVG--
Karenia1                           GHAMLSDDGG---KSWRLSAD
Emiliania                          SYAAFSDDHG---RTWAHGSP
Chrysochromulina1                  SAAIFSDDHG---ATWRRGAE
NEU2_HSA                           AFCFLSHDHG---RTWARGHF
NEU3_HSA                           SLMIYSDDLG---VTWHHGRL
NEU4_HSA                           SFAFYSDDHG---RTWRCGGL
Saccoglossus5                      SNVFYSDDGG---SSWHVGGS
Saccoglossus6                      SNVIYSDDGG---DNWHMGGI
Trypanosoma_grayi                  SMIMYSDDDG---ASWSFGSG
Oscarella2                         LKCIYSDDHG---RSWKNGTV
Oscarella1                         VKCIYSDDEG---MRWKSGAW
Saccoglossus1                      MFTVISDDHG---NTWRYGGM
Saccoglossus2                      MVCVISDDHG---ETWKNGGY
NEU1_HSA                           VFCLLSDDHG---ASWRYGSG
Orbicella                          VFCIVSDDHG---VNWRVAGS
Nematostella                       LYCVASDDHG---NSWHRTAE
Saccoglossus3                      MHCIISDDHG---DTWKLAGT
Saccoglossus4                      ASCLYSDDYG---DTWHLGGR
Monosiga5                          SCLLLSSDHG---ASWRFAAL
Metarhizium_XP_007806611.1         ---GKGTPGQ---RTWSYKRL
Aspergillus_XP_751452.2            ---GRGAPGN---RTWSVQRL
Arthroderma_XP_002847861.1         ---GTGPAGN---RTWSMQIL
Emiliania_XP_005775390.1           GFLAVSKDYG---ETFTASQH
Chrysochromulina3                  TFLYWSSDFG---ASWRASEL
Chrysochromulina2                  GFLYWSRDLG---RTWRASEV
Rhodanobacter                      VVNIYSDDHG---TSWKTGVP
Arthrobacter_OOP63695.1            AYSVYSDDHG---KSWNMGTP
Sporothrix                         AYSVYSDDHG---ATWQMGTP
Sporothrix_KIH89835.1              AYSVYSDDHG---ATWQMGTP
Pseudogymnoascus                   AYSVYSDDGG---ATWHMGSP
Penicillium                        AYSVYSDDGG---ATWKKGNV
Blastocystis                       DPVWYTDDVG---KTWAASNT
Monosiga1                          DSVWYSDDGG---LTYNVSKT
Monosiga3                          GVVWFSDDQG---ASYVVSAV
Karenia2                           DYISYSDDRG---TTWKTIGQ
Endozoicomonas                     ALVLLSDDLG---QNWRKSEL
Chrysochromulina4                  AHVIYSDDHG---ATWHIGGV
Psychromonas                       NYAYWSDDHG---ETWEIGGL
Janthinobacterium                  AYVFYSDDHG---ASYQIGND
Pedobacter                         AHTIYSDDHG---KSWNLGNN
Rickettsiales                      SHIIFSDDGG---NSWEIGGI
Kriegella_WP_089893641.1           SHVIYSDDHG---KTWQLGGT
Sphingobacteriales_OJY82384.1      SHAIYSDDGG---DNWKLGNS

Reticulomyxa_ETO10819.1            EWSWNACCEIVRR--YGRRLEVTQL-SCLHLYLDRMRLRSQVN-------------LASD
Bathycoccus                        LPFWREPGKTCPQ----IKSEVK----DASLLR----------------KGSAGVIRSTD
Bathycoccus_XP_007512415.1         LPYWREQQNAIEDGTCTRKPEFK----GSSGPDCRWNKRSRCMTGA---QPFSGVLVSEN
Aureococcus                        GSHGANEMQLV---------QLR----NGSLLA---NSRSLSTGS----RQARLAARSDD
Monosiga4                          ----GNECQAV---------ELP----DGTVMM---NMRTSQAHR--------QVAFSKD
Monosiga2                          DPFFSNENQAA---------VLS----NGSLFF---SARTL-LER----R---SQWLSHD
Bodo                               GIEWTNECQAV---------ELE----PNHLLI---TTHGFVLPQ----R---MQVESFD
Karenia1                           GRHFPNEDQAV---------ELS----GGHVAI---FARGLGFFR----T---RTV-SED
Emiliania                          PVYGGGENQIV---------PYG----GGGTLAM--FLRGRTT-AADDVSH--------N
Chrysochromulina1                  VVYMGGECQVT---------SYA----PAGPQGLVMLMRVRGDFPTNAVDHNHATAISRD
NEU2_HSA                           ------ECQVA---------EVETG--EQ-RVV---TLNARSHL------RARVQAQSTN
NEU3_HSA                           ------ECEVA---------EVTGR--AGHPVL---YCSARTPN------RCRAEALSTD
NEU4_HSA                           ------ECQLA---------AVDGG--QAGSFL---YCNARSPL------GSRVQALSTD
Saccoglossus5                      KTIHANESQVT---------ELDDG--V--ICI---NCRTLGVE------MPRVQAFSKD
Saccoglossus6                      QKIITNEAQVA---------ELDNG--V--VVM---AMRTLMSR------QPRAQAFSYD
Trypanosoma_grayi                  -HIGCSEPSLV---------EW-----EGKLIM---NARVDNA--------PRKVFESTD
Oscarella2                         GDFLPNECQPV---------EMS----DGSILI---SIRNQYKY----HGAARMFASSVD
Oscarella1                         HDFTPSENQLV---------ELS----DGSILI---TTRNDVGY----HGPYRVLARSFD
Saccoglossus1                      GDFQPDECQPV---------ELP----DGSIMI---NMRNQGHY----HCSCRIITRSFD
Saccoglossus2                      GDFNPNECQPV---------ELP----DGSIMI---NMRNQGHY----HCSCRIITRSFD
NEU1_HSA                           NDFNPDECQPY---------ELP----DGSVVI---NARNQNNY----HCHCRIVLRSYD
Orbicella                          GEFEPDESQLI---------ELY----DGTLLM---TSRNQQSF----HCHCRIMSKSYD
Nematostella                       NQFDPNECQMI---------EQS----DGVVLL---SARNQQHE----ECRCRMMAKSYD
Saccoglossus3                      GDFQMDETTII---------ELP----DGSLML---NSRNEHHY----HCRCRIIATSED
Saccoglossus4                      GDFAPGESQIV---------EMY----DGTLIM---MSRNTEAF----HCKCRIFSKSYD
Monosiga5                          L--GSREAQAV---------QIASNTSSAWLYV---TERNLGP-----MPGHRMYAISHD
Metarhizium_XP_007806611.1         ---AGAEGTIV---------QTP----DGKLYR---NDRAGKDE------EYRKIARGTL
Aspergillus_XP_751452.2            ---AGAEGTIV---------QTP----DGKLYR---NDRPSQ-K------GYRMVARGTL
Arthroderma_XP_002847861.1         ---AGSEGTIC---------QTP----DGKLLR---NDRPGP-K------GHRSVARGTL
Emiliania_XP_005775390.1           E--PGSECSIA---------FAAGP-GNSTLIM---NCRSGQDS------RRAQLYWSLL
Chrysochromulina3                  DEFGLDECSIA---------WLRNS-SDGEILM---NCRTRLNE-------RALMTWTAD
Chrysochromulina2                  ---SLNECSIA---------LLANQ----SIAM---NCRTAGSF-------REQLTWAPD
Rhodanobacter                      D--NLDESKAV---------ELA----DGTVME---NMRSSDPR-----MHTRMLARSHD
Arthrobacter_OOP63695.1            T--GMDENKTV---------ELS----DGRVML---NSRDSTGA----NPHFRKVAYSSD
Sporothrix                         V--GMDENKVV---------ELS----NGTVML---NSRPSDGS------GYRKVALSND
```

```
Sporothrix_KIH89835.1              V--GMDENKVV---------ELS----NGTVML---NSRPSDGS------GYRKVALSND
Pseudogymnoascus                   T--GMDENKVV---------ELS----NGNVML---NSRPSDGS------GYRKVAISTD
Penicillium                        N--GMDENKVV---------ELS----NGNLML---NSRPSDGS------GYRKVAISTD
Blastocystis                       K--KMDEAQLV---------ELG----DGIVMA---NMRNQHLQ----G-NTRAVSISMD
Monosiga1                          T--FMDEAQLV---------ELP----DGRVMA---NMRNNHIN----ACKCRAYALSED
Monosiga3                          ---EMQECQVA---------ELA----DGTLLI---NFRNSHLNSS---CDCRAQSVSHD
Karenia2                           P--KMDEATLA---------DLG----GGSVLL---NMRHRAEN-----TKGRGVARSQD
Endozoicomonas                     ---DGDESAVA---------ELE----DGSIYL---STRQHAALGRAPAPNGRWMSTSDN
Chrysochromulina4                  K--HTNECTIS---------QLG----DRRVLL---NARDWSGR------FLRAVQLSSD
Psychromonas                       E--SLNEVQAV---------ELA----NGDIMF---NSRNYRPT----ADKRRAVTLSHD
Janthinobacterium                  P--SANESTAA---------ELS----TSQLML---NSRDQSGL----T-KKRIVSVSSD
Pedobacter                         D--KMNETTLA---------EIS----KGRLML---NMRNSDRT----I-KTRHTAISKD
Rickettsiales                      D--NVNECTIA---------EIG----RGKLIL---NMRNYDRT----K-MNRQISISND
Kriegella_WP_089893641.1           D--QVNECEVV---------ELS----DHSLML---NMRNYNRN----K-NMRQLAYSYD
Sphingobacteriales_OJY82384.1      D--QVNECTIA---------ELS----DGKLML---NMRNYNDT------RVRQVSVSKD

Reticulomyxa_ETO10819.1            QELR--IQAIIA------KTVRCLAHPSSS-----TDNTTSDGCVSPQ-SPFHSSS----
Bathycoccus                        QGLT--WQVFGN--------LTATKGES-------------WLIENTV-VERKSTTKNKK
Bathycoccus_XP_007512415.1         RGKT--WQPRGQ--------ITQ--SNT-------------SLIEGSI-AE---------
Aureococcus                        RGLT--WTPSSF--------VDALPEPF-------------NGCQGSL-AR---------
Monosiga4                          GGAT--WSDPVV--------T----VNG-------------TVCEGST-IM---------
Monosiga2                          NGES--FGPVQL--------VETLIQPV-------------DGCEGSI-VT---------
Bodo                               GGMT--LGE-PY--------FIDIGAPF-------------GGCEGSI-AK---------
Karenia1                           GGDH--WGTTEV--------LETLPQPF-------------IGCEGST-IK---------
Emiliania                          HGLA--W-------------LN---ISG-------------SYCEGSA-VA---------
Chrysochromulina1                  GGET--WTVASL--------IH---ATS-------------SYCEGSI-AT---------
NEU2_HSA                           DGLD--FQESQL--------VKKLVEPP------------PQGCQGSV-ISFPSPRS---
NEU3_HSA                           HGEG--FQRLAL--------SRQLCE-P------------PHGCQGSV-VSFRPLEIPHR
NEU4_HSA                           EGTS--FLPAER--------VASLPE-T------------AWGCQGSI-VGFPAPAP-NR
Saccoglossus5                      DGMT--FTVAQV--------VDKLVEPGYTGSFM-PSTKNFGGCQGSI-LGFPAPEGV--
Saccoglossus6                      GGIT--FQKAEV--------HTSLIEPGYKESYGFLIPKNTPGCQGSI-LSFPAPASN--
Trypanosoma_grayi                  MGET--WSEAVGTLSRVWGPSPSRTEPG---------------SQGSF-ITVTI------
Oscarella2                         GG----------------------------------------------------------
Oscarella1                         A-----------------------------------------------------------
Saccoglossus1                      GAETFSFDNLYF--------DEALIDPA---------------CAAST-LYHN-------
Saccoglossus2                      GAETFSFDNLYF--------DEALIDPA---------------CAAST-LYHN-------
NEU1_HSA                           ACDTLRPRDVTF--------DPELVDPV---------------VAAGAVVTSS-------
Orbicella                          GAESFAHSDIYF--------DETLIDPV---------------VAASL-LQYR-------
Nematostella                       GCETFAHSDIYY--------DHTLIDPT---------------VAASL-LNWK-------
Saccoglossus3                      GADTFPFQNIRL--------DETLVDPA---------------CDGSL-ISHN-------
Saccoglossus4                      AGESFPVSDIWM--------VDDLPDPN---------------VCGSI-LHHN-------
Monosiga5                          AGHS--YTETGI--------NKGLVTPI------------TADWTGTV-ASVESS-----
Metarhizium_XP_007806611.1         A----KFGSFTL--------DKGLPDPD---------------CAGAT-LLFNRA-----
Aspergillus_XP_751452.2            E----GFGAFAP--------DAGLPDPA---------------CQGSV-LRYNS------
Arthroderma_XP_002847861.1         S----GFGPFAT--------DNGLPDPA---------------CQGSI-LSYNR------
Emiliania_XP_005775390.1           DNGTYASTEPTY--------PTQLTDPG---------------CQGSI-LSAAAGAGS--
Chrysochromulina3                  G--L-P-GR-VT--------YPGLTDEN---------------CQGSL-LNAG-------
Chrysochromulina2                  G--S-LLGEIRR--------PAGLVDPN---------------CQGSL-LAHG-------
Rhodanobacter                      GGIH--FGPVAP--------EPQLPDPH---------------NNGDI-IRVAPLAA---
Arthrobacter_OOP63695.1            GGVT--YSTPVA--------ETQLPDPR---------------NNGSI-TRMYPDAA---
Sporothrix                         GGLT--YTTPRT--------ETQLPDPA---------------CNGQI-SRLYPSAA---
Sporothrix_KIH89835.1              GGLT--YTTPRT--------ETQLPDPA---------------CNGQI-SRLYPSAA---
Pseudogymnoascus                   GGVT--YSTPKS--------ETQLPDPA---------------NNGAI-TRMYPDAA---
Penicillium                        GGVN--WSTPKT--------ENQLPDPG---------------NNGAI-ARAYPDAK---
Blastocystis                       GGEN--FGPIYY--------DPALIDPI---------------CAASI-IRDY-------
Monosiga1                          GGAS--FGAIQF--------ADALVSPV---------------CMASI-LRGL-------
Monosiga3                          GGLT--WGPLTY--------IPDLIEPV---------------CSAGL-VAAN-------
Karenia2                           GGLS--WSNITY--------DSALIGPV---------------CQGSM-AAFN-------
Endozoicomonas                     GGIS--WLDVKK--------DVALYTPV---------------CQASV-LEYG-------
Chrysochromulina4                  DGAS--WQKHRY--------DRELIEPR------------PQGCQGSM-LCVPPP-----
Psychromonas                       GGSS--FGETVD--------DDNLIEPT---------------VASAV-IRYT-R-----
Janthinobacterium                  GGAN--FSAGFA--------NATLIDPV---------------CEGSL-LNLG-W-----
Pedobacter                         GGQN--WANVQI--------DTTLIEPI---------------CQGSL-LSHF-Y-----
Rickettsiales                      YGES--WEKLQF--------DETLVEPI---------------CQASI-LRYT-F-----
Kriegella_WP_089893641.1           GGNS--WENMGH--------HDELIEPI---------------CQASI-LRHK-S-----
Sphingobacteriales_OJY82384.1      GGHS--WADLKG--------DPALIEPV---------------CQGSL-LSYK-Q-----

Reticulomyxa_ETO10819.1            QFNFFFI---------------------S-------------------------------------------
Bathycoccus                        DDSSTSFFVAX-------------------------------------------------------------
Bathycoccus_XP_007512415.1         LLLAFNNMKNQYRSRRGRACRTHLHVAISRD--GDGNEWETIARL-----EDEIG-YEA---PRIHYPYMAQ
Aureococcus                        -------LPRALRG-LGLTGRDHLTLWASR---DEGDTWDV--RG-----VVDAG-TAG-YSSLA-------
Monosiga4                          -----------------SNDRANVSIQ-TS---TDGLSWGN--SI-----QVYAG-SSA-YSSLQ-------
Monosiga2                          ------------------DLRYNLTAHVSQ---DNGTSWTP--SV-----VIDPL-PSG-YSALV-------
Bodo                               ------------------------------------------------------------------------
Karenia1                           -----------------TRLREDMSIYVSD---DEGRTWQK--YM-----RVDPG-FSA-YSALA-------
Emiliania                          ------------------------QPWM--------TSLTVVY--------FWPG-FKA-TAPEMGYPVLQP
Chrysochromulina1                  -----------------NGGRANLTVWAAR---PVGESLDAQYSL-----TVYPG-ASA-YSS---------
NEU2_HSA                           QWLLYTH--------PT-WQRADLGAYLNPRPPA-PEAWSE-PVL------LAKG-SCA-YSDLQ---SMGT
NEU3_HSA                           SWLLYSH--------PT-KQRVDLGIYLNQTPLE-AACWSR-PWI------LHCG-PCG-YSDLA---ALE-
NEU4_HSA                           TWLLYSH--------PV-RARLHMGIRLSQSPLD-PRSWTE-PWV------IYEG-PSG-YSDLA---SIGP
Saccoglossus5                      TWALFSN--------PA-ENRINLSVRLSID--G-CKTWSA-PWS------LCPG-PSA-YSDLT---YFEM
Saccoglossus6                      KWVLFSN--------PA-TTRKYMSVRLSYD--S-CKTWSE-PWT------IYPW-YSG-YSDLT---YLGI
Trypanosoma_grayi                  RVMLFTV--------PL-------KGRWLRD--R-LHLWVTDNRRIFDVGQVSDGDEDSPYSSLL---YTKA
Oscarella2                         ------------------------------------------------------------------------
Oscarella1                         ------------------------------------------------------------------------
Saccoglossus1                      GVLFFVN--------AM-NKRENLTLQWSYN--N-GTTWSG-KLN------IFPG-YSA-YSAMT---AFSN
Saccoglossus2                      GVLFFVN--------AM-NKRENLTLQWSYN--N-GTTWSG-KLN------IFPG-YSA-YSAMT---AFSN
NEU1_HSA                           GIVFFSN--------PA-EFRVNLTLRWSFS--N-GTSWRKETVQ------LWPG-PSG-YSSLA---TLEG
```

```
Orbicella                         NTVYFSN--------PA-LLRINMTVRWSGD--K-GTSWVG-SLG------VWKG-PSG-YSCLT---NVPG
Nematostella                      YTVYFSN--------PA-TLRVNMTVRVSGD--M-GETWKG-LRP------IWQG-PSG-YSCLS---GIPT
Saccoglossus3                     NVLFFSN--------PA-SARVNMTLRWSLD--F-GKSWDG-ALV------INAG-SSE-YSCLS---SIDD
Saccoglossus4                     GILYFSG--------LD-TSRTNMTLYWSLD--N-GITWPG-YLP------IYLH-NSA-YSCLT---AIDN
Monosiga5                         SPLFMAL--------PF-TVRCNLTLMTSLD--A-GATWAA-QTV------FWPG-LGA-YTDLS---ALPT
Metarhizium_XP_007806611.1        ARVVFLN--------SA-NTRRAMRVRISYD--KNAKEYSH-GRKLSDAPVSGAG-NEGGYASLT---KTAD
Aspergillus_XP_751452.2           ARTIFLN--------SA-TSRRAMRVRISYD--ADAKKFNY-GRKLEDAKVSGAG-HEGGYSSMT---KTGD
Arthroderma_XP_002847861.1        ARTIFMN--------SA-DRRTAMR---------DAAKFNY-GRDLQDAPLKNVG-NEGGYSSMT---KTSD
Emiliania_XP_005775390.1          HVLYTSN--------AA-SARERMTIHRSDD--G-GVSWSA-GQV------IHAG-PSA-YSQLV---QLPN
Chrysochromulina3                 GAHFLSH--------IN-WERAHLQIHRSTD--G-GKNWTE-VRK------VRRG-AAA-YSQLI---ELGA
Chrysochromulina2                 GALYLSN--------DN-SGRTHIVVKRSAD--A-GLTWDE-GRL------IWAG-PAA-YSQLV---GMGD
Rhodanobacter                     HWLLFSN--------TA-HERRRLTLRLSCN--D-GRTWDA-GRV------LDAG-DAM-YSVMT---RLPD
Arthrobacter_OOP63695.1           KKLLFSN--------SPGGARVDGTVRYSAD--D-GKTWGS-SRV------FKAG-SMS-YSTLS---ALSD
Sporothrix                        KILLFTN--------AN-TSRANGTARFSCD--D-GNTWST-GTV------FSPG-ATS-YSTIT---PLGD
Sporothrix_KIH89835.1             KILLFTN--------AN-TSRANGTARFSCD--D-GNTWST-GTV------FSPG-ATS-YSTIT---PLGD
Pseudogymnoascus                  KILLFTN--------AN-TNRVNGTARYSCD--D-GTTWSS-GRA------FQTG-VTS-YSTIT---ALGN
Penicillium                       KILLFTN--------AN-SSRSNGTIRYSCD--D-GKTWSA-GSV------FQKG-TTS-YSTVT---ALGG
Blastocystis                      NVIYFSN--------AA-TKRINMSVKRSSN--W-GKTWDR-TLL------VYEG-PSA-YSDMID--LDSD
Monosiga1                         NVY-FAN--------PG-SGRSG-GMLRRSA--D-GFAWNQ-SAL------VWPG-SYA-YSCLTH--VPKS
Monosiga3                         DTLYFTN--------PN-DQRINMSVQSSMD--G-GNTWSH-EVT------LWPG-PSG-YSVATNH-PNSK
Karenia2                          GSVFFSN--------PA-SGRDHLTVRRSDD--G-GHTWTA-KLL------IQEG-RSAGYSSLVQGPVLDD
Endozoicomonas                    KGLLFSN--------PA-KARVNMTVRLSND--Q-GSSWNT-SVS------VYPG-ASG-YSDLG---ALSN
Chrysochromulina4                 GILFFCN--------PS-DRREMLTIRRSDD--G-GMTWSR-SYC------LEEG-PSA-YSCLG---RTAD
Psychromonas                      NRLLFSN--------PH-SSRVNMTVQLSYD--E-GQSWPV-KKT------INPG-TSA-YSDLV---IHDD
Janthinobacterium                 QYLLHAN--------PA-TSRNNLTVRASKD--D-GQSWPF-SLL------ITAG-ASA-YSDLT---QVDA
Pedobacter                        PTLLFSN--------PA-KLRANLTLRLSKN--D-GKTWKY-NMV------LHPG-PSA-YSDIA---VIDK
Rickettsiales                     NFLFFLN--------PG-GERINMTLRLSRD--L-GLSWDE-MII------LHKG-PSA-YSDIT---KLPN
Kriegella_WP_089893641.1          VNIFFLN--------PA-KKRVNMTLRMSDD--D-GKTWGQ-SRQ------IFAG-PSA-YSDLT---PIDD
Sphingobacteriales_OJY82384.1     KVLAFSN--------PA-EQRINMTVRLSSD--N-GKTWRY-SKV------LHEG-PSA-YSNLV---VLPN
```

3 – MSA CMP-Sialic acid synthetase (CMAS)

```
Guillardia                  ------------------------------------------------------------
Hydra2                      RMAASRFPGKPMYPILGRPMVEHVFLRAAMYQGWSK------------------------
Bartonella                  RIGSTRLPQKALAEIAGKPMIVHVAEQAKKAA-FGR------------------------
Dictyostelium               RLAATRFPNKPMAPIGGIPMIGHCYLRSKLCSLLDE------------------------
Zea                         RFASTRFEGKPLVPILGKPMIQRTWERVMLASSLDH------------------------
Bathycoccus                 RYGSTRFPGKPLADIAGKPMIWHTYTNACKSRALDL------------------------
Amphimedon                  RYASSRLPGKVLADIGGRPMIALVCELALAGR-VDA------------------------
Clavaria                    RLASTRLPNKPLADIAGLPMIVRVARRAAESG-AGR------------------------
Puccinia                    RYASTRLPGKPLVDIHGKPMVVHVMERARESG-ASR------------------------
Pleurotus                   RFASSRLPGKPLADIGGKPMVVHVMERAKESG-ADR------------------------
E_coli                      RYASTRLPGKPLVDINGKPMIVHVLERARESG-AER------------------------
Beauveria                   RYASTRLPGKPLKEINGKAMVLHVLDRARESG-AER------------------------
Drosophila                  RGGSKGIKLKNLAEVGGISLLARTIKTIQNSACFEH------------------------
Acyrthosiphon               RGGSKGIPRKNLAVIQNTTLLRRSLDTINDCGLFHD------------------------
Bemisia                     RGGSKGIKKKNLSVVNGKTLIKRAVENILESDVFDS------------------------
Chromera                    RGGSKGLPRKNVRPLAGLPLIAYSIRAGQAASSVDR------------------------
Thelohanellus               RGGSKRLPNKNIMLLADKPLIVWTIEAALDSHCFDH------------------------
Campylobacter               RGGSKGVKNKNIRKINKLEMIAYSIIQAQNSKLFKH------------------------
Condylostoma                RGDSKGLPGKNTKNLLGKPLIAYSIEQALQAKEIDA------------------------
Bradyrhizobium              RGGSKGVIGKNARELLGKPLLAWSIEQARQTGLFEA------------------------
Tepidicaulis                RGGSKGVPGKNIRPLGGKPLIAHSIVQALASGLFET------------------------
Prorocentrum                RGGSKGVPGKNIRPLLGKPLIVHSIEQAQASKLFEH------------------------
Shewanella                  RGGSKRIPRKNIKLFKGKPIIGYTIEAALQSGCFEQ------------------------
Hydra1                      RGGSKRIPRKNIKSFCGKPMIAWSIQAARKSGLFDR------------------------
Pelobacter                  RGGSKGIPRKNIKNLAGRPLIYWVLDAATRCDLIDK------------------------
Chromerat                   ------------------------------------------------------------
Nitrosomonas                RANSKSIPDKNIRSIAGRPLFAWSLEQAIVSQCFNE------------------------
Hydrocarboniphaga           RGGSKSIPGKNIRPLAGRPLFAWSLGAAIDSGCFDE------------------------
Aureococcus                 RGGSKGIPRKNIKPLNGVPLLVYNVKAALASGVFGR------------------------
Emiliania                   RGGSVSVPRKNIKILQGRPLIDWVIQAAEYSGIFDEARRAAGTDIITAAATAAAAVSATP
Chrysochromulina            RGGSVSIPKKNIKLLLGRPLIDWVIKAARDSKIFAE------------------------
Galaxea1                    RGGSVSIPLKNIKDLAGRPLLAWCVKAALDSGCFDE------------------------
Micromonas                  RGGSKGIPGKNIIDLNGKPLIQYTIEAALSSKQLDR------------------------
Paramecium                  RGGSKGLPGKNIKPLNGIPLITHSVQHALASKYPKK------------------------
Acytostelium                RGGSKQVPRKNLQRVGGVPLVERAVRAAAAAPGIDL------------------------
Daphnia                     RGGSKGIRLKNLALLKGTPLLLWSLKTMSQSRGLTS------------------------
Strongylocentrotus          RGGSKGIKLKNIKALAGQPLIAWVLRAAIDSGEFDS------------------------
Podocoryne                  RGGSKGIPLKNIKVLAGVPLIGWVIRAAFDSGVFDS------------------------
CMAS1_Dre                   RGGSKGIPLKNIKMLAGVPLIGWVIRAAVDSNVFNS------------------------
CMAS1_HSA_Q0E671.1          RGGSKGIPLKNIKMLAGVPLIGWVIRAAVDSNVFNS------------------------
Homo_sapiens_AAH16609.1     RGGSKGIPLKNIKHLAGVPLIGWVLRAALDSGAFQS------------------------
CMAS2_Dre                   RGGSKGIPLKNIKNLAGVPLIGWVLRAALDSD-VDS------------------------
Galaxea2                    ----------------GVPLIAWVLRAALDSEVVDS------------------------

Guillardia                  ---------IANLIEGIGAK--CIMTSHECESG-TERVREASQQLSA-------------
Hydra2                      LTLATCDDEIANFAASKGIS--CTMTGSHHTRA-LDRVAEAVQRLGEPVA----------
Bartonella                  IIVATDHNNIAKVVTAYGHE--CIITCRDHKSG-SDRIYEALTHIDPER-----------
Dictyostelium               VYVATCDQEIKDYIESIGGK--AVMTSDIHERA-TERSAEALLNIEKELNG---E-----
Zea                         VVVATDDERIAECCRGFGAD--VIMTSASCKNG-SERCCEALKKLD--K-----------
Bathycoccus                 CIVATDDARIKKVVESFGGI--CVLTNPECVNG-TERCLEVYKKCI--KKG---E-----
Amphimedon                  VIVAGDDERILEAARAEGAQ--AILTSSEHRSG-SERIAEAISLLG--LD----------
Clavaria                    IVVATDAPEVAAACAAHGVQ--ALMTRADHPSG-SDRLAEAVEQLG--LA----------
Puccinia                    VIVATDHPEVARAVEAAGGE--VCMTRADHHSG-TERLAEVIEKYQ--FA----------
Pleurotus                   VIVATDHPDVFAAVEAAGGE--VCMTRADHQSG-TERLAEVIELYG--FS----------
E_coli                      IIVATDHEDVARAVEAAGGE--VCMTRADHQSG-TERLAEVVEKCA--FS----------
Beauveria                   IIVATDHPDVARIVEAAGGE--VCLTSPDHQSG-TERLAEVIEKCG--FS----------
Drosophila                  IWVSTDDKRIALEAQKYGAI--IH-DRPEKFAKDNTPSIDAIK----EFLE-VHA-----
Acyrthosiphon               VWVSTDDKEIAAEAELAGAK--VF-HRSAETASDEATSLSAVK----EFSM-YHQ-----
Bemisia                     VWVSTDDMEIAMEAQKATAL--LH-WRHPSTATDSAASILGVQ----DFFA-KHP-----
Chromera                    LVVSTEDAEIAAVARDSGAE--V-VERPAALARDDTPTRPVIEHAVELLAGG-GY-----
Thelohanellus               VYVSTDSPDIGIIAKIAGAE--VPYLRPDNLATDEAATNDVVDHLVGWVEKNVAP-----
Campylobacter               IVISTDSDEIASVAQKYGAE--VFFKREAHLANDRTAKLPVMRDALLRSEEHFKT-----
Condylostoma                VYCSTDSQQIADIAQQYGAI--IPALRPKELADDYCSKLDVINHLVALVKPNHND-----
Bradyrhizobium              IAFSSDSDLLLETALQAGAD--LAVKRPDEMATDTAPKIPAIRHCLEQAIARTGI-----
Tepidicaulis                VAVSSDDEAILRAAEEAGAD--ELVLRPADLASDTAPKLPAILHCVEEVEKRHGH-----
Prorocentrum                IAVSSDDQAILETARAAGVQ--HLVQRPPELATDSAAKLPAIRHCVETVEKDIGE-----
```

```
Shewanella                   VIVSTDDDEIAAVAESFGAI--VPFRRPAEFANDHATTMQVVIHGIDWMLR-NGL-----
Hydra1                       VVVSTDDLEIAEVARQWGAE--VPFMRPPEISDDHAGTTPVIAHAVRWFID-QGI-----
Pelobacter                   VYVATDDAEIAEVVDRFGSERVEVVSRGSETATDTASTESAML----EFSR--QR-----
Chromerat                    -----------------------------ENASDTASTEAAML----EFCEKAEA-----
Nitrosomonas                 IYVATDSSRIRRMVLDEFSSGVTVLDRSAETCTDTASTESALL----EFQQ--RI-----
Hydrocarboniphaga            IWVGTDSDDIRRSVEQEFGPKVKLFRRGAQTCTDAASTESALL----EFAT--AV-----
Aureococcus                  VVVSSDDDEILAVAARAGAS--TH-ARSAASAADAASSEAGVF----DYVD-ATP-----
Emiliania                    VWVSTDDDEIHMIAKKCGAK--VH-RRSPHTATNTASTESALV----DFVK-ARRLAWPA
Chrysochromulina             IWVSTDDDLIAASAIKCGAL--VH-RRAAHTATSTASTESALK----DFAD-AHP-----
Galaxea1                     VYVSTDHDGIAEVAGKAGAR--VH-RRAPETATSSASTELAMA----DFAR-AHP-----
Micromonas                   VILSTDSDEIAEVAQNCGCE--VPFRRPSELAADDSSHLACIVHALNILRETECF-----
Paramecium                   VIVSTDDAKIAAVAKEAGAE--IV-VRPADISGDTASSESALIHALTTVEQ-QGY-----
Acytostelium                 VVVSTDDDEIAAVATSAGAR--VV-RRPAALSGDTATSESAILHALDDLEA-NGD-----
Daphnia                      IWVSSDHDDILELAHQNGAQ--IH-RRSAESSSDGASSLDAIN----EFLD-SHS-----
Strongylocentrotus           VWVSTDHADIARISKEWGAQ--VH-MRSPHTARDQATSIEAMQ----EFLK-EHP-----
Podocoryne                   VWVSTDHAKIAEVAEEFGAQ--VH-RRSPKVSTDSSSSLDAIQ----EFLR-LHN-----
CMAS1_Dre                    VWVSTDHEEIAKVALAWGAK--VH-KRSPEVSQDSSSSLDTIR----EFSR-QHR-----
CMAS1_HSA_Q0E671.1           VWVSTDHEEIAKVALAWGAK--VH-KRSPEVSQDSSSSLDTIR----EFSR-QHR-----
Homo_sapiens_AAH16609.1      VWVSTDHDEIENVAKQFGAQ--VH-RRSSEVSKDSSTSLDAII----EFLN-YHN-----
CMAS2_Dre                    VWVSTDHDEIERVAKLWGAK--VH-RRSPEVSKDSSSSLETIQ----EFIR-LRP-----
Galaxea2                     VWVSTDHDEIERVAKVWGAK--VH-RRSPEVSKDSSSSLETIQ----EFLR-LRP-----

Guillardia                   --EFDIIVNVQGDEPLIDPAHVDKLITHMKK--NQADLVSTLACPIRSM-----------
Hydra2                       --EDDIVVCVQGDEPMMRPDMINVVIEPLLH--DASKAGTILAMHIVDK-----------
Bartonella                   --RYNVILNVQGDLPTITPHEIISALRPLEN---SLTDIATLGAKIVEE-----------
Dictyostelium                --VFDIVVMIQGDEPLIYPEMIEEVLEPMLK---KNVPVSNLMAALPTQ-----------
Zea                          --HYDIVVNIQGDEPLIEPEIIDGVVMSLQR--APDAVF-STAVTSLKP-----------
Bathycoccus                  --EYDVIVNIQGDEPFIEPEHIDLVAQIVCEAEEDDVCMGTLCRPAIDR-----------
Amphimedon                   --PDVVVVNIQADEPLLPPTLIDQVAGLLVD--RPEFAMATLCEPIEKA-----------
Clavaria                     --DDAVVVNVQGDEPLIAPAMIDACAATLTA--QTDCVMATVAHALTDA-----------
Puccinia                     --DDQIIVNVQGDEPMIPADIVHQVATNL-A--QADAGMATLAVPITDA-----------
Pleurotus                    --DEDIIVNVQGDEPLVPPVIIKQVAENL-A--SCEAGMATLAVPIETA-----------
E_coli                       --DDTVIVNVQGDEPMIPATIIRQVADNL-A--QRQVGMATLAVPIHNA-----------
Beauveria                    --DDTVIVNVQGDEPMIPPVIIQQVANNV-A--TSQAGMATLAVPIESA-----------
Drosophila                   --TVKDFALFQCTSVFLKPTYIQEAVQKF-------------------------------
Acyrthosiphon                --KVDIFAIIQCTSPFMTAEYLTRAYKMA-------------------------------
Bemisia                      --DLEVIGLIQCTSPFLTSQFLIKALRLI-------------------------------
Chromera                     --VSDAVLTLQPTSPLRTARHIDEAAALF-------------------------------
Thelohanellus                --I-AKVAILQPTSPLRTAYHIKEAFSLY-------------------------------
Campylobacter                --CFETLIDLDASAPLRSSLDIKKAYESF-------------------------------
Condylostoma                 --L-SRIIDLDVTSPLRDIIDITACVELL-------------------------------
Bradyrhizobium               --TPDVFVDLDVTSPLRLPSDIAGAVELL-------------------------------
Tepidicaulis                 ---SDVVVDLQPTAPLREPSDIQGAVALL-------------------------------
Prorocentrum                 --LFDVVADLQPTSPLRLPSDIVGAVALL-------------------------------
Shewanella                   --APKKMCLMYATAPFITPVLLQESLALLDANPNK-------------------------
Hydra1                       --SPELVCCLYATAPFVHISDLRAGLA---------------------------------
Pelobacter                   --NFEHMVLIQATSPLLEAGDLDRGIRKY-------------------------------
Chromerat                    --DFDVFCLLQATSPLTQRTDIDRGLEKL-------------------------------
Nitrosomonas                 --SFDVVGLIQATSPLTQADDFHAARHKF-------------------------------
Hydrocarboniphaga            --DFDVLCTIQATSPLTRVEDFRRAQQRF-------------------------------
Aureococcus                  --SCAVCCLVQCTSPLTGAEDFAAGHARF-------------------------------
Emiliania                    PPIAPLRSRRRATSPLITPLDFQKGWDAMRRAEISPRSRRDIVGRCRAPRDEISAEIELT
Chrysochromulina             --DYEYMCLIQATSPLIRPEDFVEAMRIM-------------------------------
Galaxea1                     --DYDVLCLIQATSPLTTPYHFREAIALF-------------------------------
Micromonas                   --VPDFVVILQPTSPFRKSIDIDSCINIM-------------------------------
Paramecium                   --IPDLIVFLQCTSPIREMDDIDNAIELL-------------------------------
Acytostelium                 --AVGIVAFLQATSPFIPSDALADAVEEI-------------------------------
Daphnia                      --EVDVVALIQCTSPFVRVAHLNEALAKM-------------------------------
Strongylocentrotus           --EVKFVANVQCTSPCLHPSHLQRTCHMI-------------------------------
Podocoryne                   --EVDIIGNIQATSPCLHPNHLQDATRKI-------------------------------
CMAS1_Dre                    --EVDVICNIQATSPCLHPKHLTEAVELI-------------------------------
CMAS1_HSA_Q0E671.1           --EVDVICNIQATSPCLHPKHLTEAVELI-------------------------------
Homo_sapiens_AAH16609.1      --EVDIVGNIQATSPCLHPTDLQKVAEMI-------------------------------
CMAS2_Dre                    --EVDVICHIQATSPCLHPHHINEALQKI-------------------------------
Galaxea2                     --EVDIVCHIQ-------------------------------------------------

Guillardia                   --------------------KDFKSPNVVKVVF----GLQNQALYFSRSPIPYLPSDFKG
Hydra2                       --------------------DIWTNPDTVKLVH----NATGEVLYTSRAPLPYCKGEFSA
Bartonella                   --------------------NEKTDPNIVKIIGTPLSHNRFRALYFTRATAPYG------
Dictyostelium                --------------------EERDNPNNVKVAK----DFNEKALY---------------
Zea                          --------------------EDAFDTNRVKCVV----DNLGYAIYFSRGLIPFNKSGNAN
Bathycoccus                  --------------------KDVEGVNNVKVVV----DRNMNALYFSRAIIPHNKSGTYD
Amphimedon                   --------------------RDIFDPAVVKVVF----DAQGRALYFSRAPIPWQREAWSS
Clavaria                     --------------------SEFTNPNVVKLVT----DAAGRALYFSRAPIAWWRDGGGQ
Puccinia                     --------------------EEAFNPNAVKVVM----DARGYALYFSRATIPWDRERYAQ
Pleurotus                    --------------------EEAFNPNAVKVVM----DAKGYALYFSRATIPWERERF--
E_coli                       --------------------EEAFNPNAVKVVL----DAEGYALYFSRATIPWDRDRFAE
```

```
Beauveria                     --------------------EEAFNPNAVKVVM----DAQGYALYFSRATIPWDRERFAA
Drosophila                    --------------------KT--HDCVFAA--------------KRSHYLRW-KEVN-D
Acyrthosiphon                 --------------------VNQKFESVFSV--------------TRTHKLRW-KLDSDG
Bemisia                       --------------------EEAHCDSVFSV--------------TRSHQLRW-NLDQRG
Chromera                      --------------------AAAPSADSLVS-C------------VRVPHIFHPNSVMRL
Thelohanellus                 --------------------KKKKANAIVSV-C------------ELEHPYQF-CNKLD-
Campylobacter                 --------------------VENDNSNLITAVP------------ARRNPYFN-LVEIQN
Condylostoma                  --------------------DDE-TDLVITGYE------------ADKNPYFN-MVEYDE
Bradyrhizobium                --------------------NRSGARSVITGAP------------ARRSPYFN-LVEERA
Tepidicaulis                  --------------------EESGAPNVITGSP------------AKCSPYFS-LVEERA
Prorocentrum                  --------------------DDGDVDNVITGSS------------AKCSPYFN-LVEERS
Shewanella                    ----------------------HYCFAVTEF--------------AAPIQRGF-SVSPAG
Hydra1                        ------------------------------------------------------------
Pelobacter                    --------------------LNNKADGLVSV--------------VRQKRFYW-QVADEG
Chromerat                     --------------------QKEGLDAVLSV--------------VNTHRFTW-SKD---
Nitrosomonas                  --------------------VAENLDSLLTA--------------VSSKRFFW-TQS---
Hydrocarboniphaga             --------------------DEQRADSLVTA--------------VRTHRFFW-TDD---
Aureococcus                   --------------------VAQGADSLVTV--------------VRAHRFLW-KQAADG
Emiliania                     SLRDTAEIQPRYSRDTADRGRRAKADSLVTC--------------VRAHRFLW-SVDKRS
Chrysochromulina              --------------------RDQKADSLVTA--------------VRAHRFLW-SVDPTT
Galaxea1                      --------------------KRHQADSLVTA--------------VRSNRFRW-RIDPDG
Micromonas                    --------------------LTSSCDMVLSV--------------CESSLNLS-KNFYFA
Paramecium                    --------------------LKEKSDSLLSA--------------SPNHRFIW-KKGPNG
Acytostelium                  --------------------RSDRADSVFSA--------------HETYAFLW-REGEGS
Daphnia                       --------------------TSGCYDSVFSV--------------TRSHSLRW-TQLEPN
Strongylocentrotus            --------------------RNLGYDSVFAV--------------NRRHLFRW-TETPID
Podocoryne                    --------------------WEDGCDSVFSV--------------VRRHHFRW-QEVKTG
CMAS1_Dre                     --------------------TKQGYDSVFSV--------------VRRHNFRW-KEVEKG
CMAS1_HSA_Q0E671.1            --------------------TKQGYDSVFSV--------------VRRHNFRW-KEVEKG
Homo_sapiens_AAH16609.1       --------------------REEGYDSVFSV--------------VRRHQFRW-SEIQKG
CMAS2_Dre                     --------------------THQGFSYVLSV--------------VRRHQFRW-EELQEN
Galaxea2                      ------------------------------------------------------------

Guillardia                    P--------DEEKSQ----------ENASD-QHRPFKHL-GIYAFRRNFLEL--------
Hydra2                        -------------------------------DLMARRIY-GIFAFRWKYLKMFTEHAETR
Bartonella                    -------------------------------DGPLYHHI-GIYAYRREALEKFVALKPSP
Dictyostelium                 ------------------------------------------------------------
Zea                           -----------------------------P-KYPYLLHL-GIAGFDSKFLKIYPELPPTP
Bathycoccus                   -----------------------------P-DTKYLRKL-GIYSYRADFLPEYVRMPESM
Amphimedon                    T--------ASLRPAADEDEDALDRLPATA-GVRHYRHI-GLYAARAG------------
Clavaria                      -------------------------------PNQALRHV-GLYAYRAGFLRRFPQLAVSP
Puccinia                      S--------R-----------------EQI-GDTLLRHI-GIYAYRAGFIRRYISWEPCP
Pleurotus                     ------------------------------------------------------------
E_coli                        G--------L-----------------ETV-GDNFLRHL-GIYGYRAGFIRRYVNWQPSP
Beauveria                     S--------R-----------------EEI-GDTFLRHI-GIYGYRAGFIRRYVSWAPSQ
Drosophila                    S----------LVPVDF-N--LESRPRRQDWKGDIVETGM-FYFSKRKLVDK--------
Acyrthosiphon                 Q----------LQPSNF-D--VTKRPRRQDWDGEYVENGM-FYFTYKNLIVN--------
Bemisia                       D----------LHAANF-D--ATKRPRRQDWNGEFVENGM-FYVFKTSLLAE--------
Chromera                      T--------EDNTLEPY-L-GEPTVTRRQDKGIAYARNGAAIYITRTDRIK---------
Thelohanellus                 ---------PSLSLDNF-L-NQKNNKRTQELERYYRLNGA-IYLFDREFVGK--------
Campylobacter                 N--------KV---VK--S-KEGNFTTRQSAPKCYDMNAS-IYIFKRDYLLE--------
Condylostoma                  D--------GSIKLVK--P-PLYGVTGRQRAPKVYAMNAS-IYAWHASSLH---------
Bradyrhizobium                D--------GSVGLSK--S-ADPPIVRRQDAPRCFDMNAS-IYVWRVAPFLE--------
Tepidicaulis                  D--------GTVGLSK--P-VDPPLARRQDAPRTFDMNGS-VYVWRREVLTA--------
Prorocentrum                  D--------GSVGLSK--P-TDPPIVRRQDAPRTFDMNGS-IYVWRRATLMR--------
Shewanella                    D------------IEMF-Q-PEHLTTRSQDLIKAYHDAGQ-FYWGKTDAFLK--------
Hydra1                        ------------------------------------------------------------
Pelobacter                    R----------AVPVNY-D--PCSRPRRQDFDGILVENGA-FYVSRKDSLLK--------
Chromerat                     -----------GKPQNY-D--FNNRPRRQDFEGLLIENGA-VYCTTKNALLA--------
Nitrosomonas                  -----------GTPLNY-H--PAKRPRRQDFEGWLMENGA-FYLTRAKVLEE--------
Hydrocarboniphaga             -----------GRALNY-D--PLKRPRRQDWAGTLMENGA-FYFSSRAVLES--------
Aureococcus                   -S---------AAPCNY-D--PVKRPRRQDWDGELIENGA-FYFFTVAALRA--------
Emiliania                     GV---------ARAVNY-D--PAKRPRRQDWDGELIENGA-FYFTTKEVMER--------
Chrysochromulina              KV---------AKAKNY-E--PLKRPRRQDWEGELVENGA-FYYYTKAHWDA--------
Galaxea1                      -A---------AHAENY-L--PAKRPRRQDWDGELIENGA-FYLTKKDVLEK--------
Micromonas                    ADG-----TLSPFAEST-A--EIDYTPRQKLLKTYAENGA-VYVLRTQSLLYPPDNAPN-
Paramecium                    F-----------YSVNY-D--YNKRQRRQDLEPEYVENGS-IYIFKPWVLKE--------
Acytostelium                  E----------AVAINH-Q--AAHRPRRQDREPHHLETGA-FYVFRAAGFRE--------
Daphnia                       ETEVDVANTEEIRAVNF-D--PHCRPKRQDWNGDLVENGA-FYLSSVQLLRR--------
Strongylocentrotus            QA-------VSTKAENL-D--PAKRPRRQDWAGELYENGS-FYFATRELLMA--------
Podocoryne                    G--------VDTKPLNL-D--PANRPRRQDWDGELCENGS-FYFATRELIEK--------
CMAS1_Dre                     GD-------CSTEPMNL-N--PACRPRRQDWSGELCENGS-FYFAKKELIEQ--------
CMAS1_HSA_Q0E671.1            GD-------CSTEPMNL-N--PACRPRRQDWSGELCENGS-FYFAKKELIEQ--------
Homo_sapiens_AAH16609.1       VR-------EVTEPLNL-N--PAKRPRRQDWDGELYENGS-FYFAKRHLIEM--------
CMAS2_Dre                     ED-------RNPKSFNI-N--VAQRPRRQDWPGELYENGS-FYFSTRKAWES--------
Galaxea2                      ------------------------------------------------------------
```

```
Guillardia                    ------------------------------------------------------------
Hydra2                        LEQLEACDSNRILDMSFRQHIAPYPNIQSYSVDSPGDIALVEKYMQGDALWESYK-----
Bartonella                    LEQREKLEQLRALEHNMRIDVEIVDTI-PLGVDTQRDLERVRKILA--------------
Dictyostelium                 ------------------------------------------------------------
Zea                           LQMEEDLEQLKVLENGYRMKVIKVDH-DAHGVDAPEDVEKIEALMRTRNIQ---------
Bathycoccus                   LQISEDLEQNKVIEAGYKIKLGWVED-AVHGVDTVGQLEALNLAIERGELMHRGKRIKKG
Amphimedon                    ------------------------------------------------------------
Clavaria                      LEQIESLEQLRVLWHGERIAVHVSPERPGPGVDTPEDLERVRRLIQV-------------
Puccinia                      LEQIELLEQLRVLWYGEKIHVAVAKTIPGVGVDTPEDLTRVRAAMLR-------------
Pleurotus                     ------------------------------------------------------------
E_coli                        LEHIEMLEQLRVLWYGEKIHVAVAQEVPGTGVDTPEDLERVRAEMR--------------
Beauveria                     LEQIEMLEQLRVLWNGEKIHVAVAKAIPSIGVDTPEDLERVRLAMR--------------
Drosophila                    -----------GLLQNNRCSIVEINVEDGLEIDSSHDLSLAKYILSSRTKTDL-------
Acyrthosiphon                 -----------NKLQGGKLGVVVIPLNRSLEIDTQFDLQAARLLAPLLDKPKNV--TNRT
Bemisia                       -----------NVLQGGRARVVEIPKNRSLEIDTEFDLVVAKVVAQILDSESQQ--MIP-
Chromera                      -----------DFVFGGRLLPYEMPLEDSIDIDGEDDWRLAEERLKAQ------------
Thelohanellus                 ----------QSELYGPNSFAYIMNSRDSVDIDNEFDFEFASFLMSLK------------
Campylobacter                 ----------NDSVFGKNTGLFVMDESTAFDIDSELDFKIVEFLISLKNLSPKDF-----
Condylostoma                  -----------KGLWSGKVKLHEMPRSRSIDIDDEIDFELVEYFMRKKIKSE--------
Bradyrhizobium                ----------SPAVFYPDTQLFEMPEERSVDIDSDLDFTLVELLLRKRLALPETQS----
Tepidicaulis                  ----------DMKLFQPGTRLYEMPEERSVDIDTALDFEFAAFLMGRRRA----------
Prorocentrum                  ----------DVGLFLEKTHLFEMPEERSADIDTELDFQFVEFLAERSAAH---------
Shewanella                    ----------NLPVFSAHSVPYLLPQHLVQDIDTLDDWHRAEWLFAMQQKEKI-------
Hydra1                        ------------------------------------------------------------
Pelobacter                    ----------SGSRLSGHVVTYEMPEATYFEIDDEVDWQIIEGMLANRKISVTSEQ----
Chromerat                     ----------SKNRLSGKIGIIEMSEDTLVEIDSETDWQVVEQLLISHFKQ--N------
Nitrosomonas                  ----------TGSRLGGRIGIHEMPAETAIEIDDEADWIVVEQLLLRQKLASIQ------
Hydrocarboniphaga             ----------GRCRLGGKIVVHEMAADSAAEIDEPEDWTTIERLIEKSRPA---------
Aureococcus                   ----------SGSRLSGRVVAHEMPEETLAEIDTLTDWQIVEGLAADKFKDAPKAARRAP
Emiliania                     ----------EECRLGGRVALHEMEEHTFAELDSPTDWQSRGNKNTNTVWQIVANMAMIH
Chrysochromulina              ----------TGCRLGGKLVLYEMAEHTFVELDSLVDWQ------------MVSHMAYDF
Galaxea1                      ----------DNCRLGGKIALYEMPEHTFVELDSPVDWS------------IMECLCKEH
Micromonas                    ---------VGSF-RSADTKGYEMPVERSLDIDNPFDLHVAR------------LLMAKP
Paramecium                    ----------NNNRLGGKVSLYLMSDESSYEIDSAMDFLIIE------------NLMKGL
Acytostelium                  ----------NRHRFFGRTRIAAVPEWTAIEIDDADQLRVAR------------A-LARL
Daphnia                       ----------GLI-QGGKISFVEMSAAHSIDIDTAYDLWLAG------------QQASYF
Strongylocentrotus            ----------GLF-QGGKVGYCEMQPEYSVDIDTDIDWPIAE------------QRVIKF
Podocoryne                    ----------GYL-QGGKMAYYEMLPEFSVDIDIDIDWPVAE------------QRVLRF
CMAS1_Dre                     ----------GLL-QGGKKTYYEMKPEYSVDIDVDIDWPVAE------------QRVLRF
CMAS1_HSA_Q0E671.1            ----------GLL-QGGKKTYYEMKPEYSVDIDVDIDWPVAE------------QRVLRF
Homo_sapiens_AAH16609.1       ----------GYL-QGGKMAYYEMRAEHSVDIDVDIDWPIAE------------QRVLR-
CMAS2_Dre                     ----------GLT-ELGRIAYYEMPPEFSVDIDVDIDWPVAE------------QRVLRF
Galaxea2                      ------------------------------------------------------------
```

4 – MSA UDP-GlcNAc 2-epimerase

```
Epimerase_2
Dictyoglomus_turgidum             VISIVGARPQFIKLAPFSIELRKS-IKEVILHTGQHYDENMS------DLFFKELEIP-E--PDYNLGIGSSSHGEQT
Naegleria_excavate_gruberi        VVIVLGTRPEAIKCAPLISHLKSNELEVIVLSTGQHSEIL--------KQTLGVFHQHVD--IDLKLMTPNQSISSFF
Ostreococcus_lucimar              IAIVFGTRPEAVKMAPVIQAVARSTLSAILISTGQHKQML--------EQVLRQFSLQDKIQHELALMKPNQQLAELT
Capsaspora_owczar                 VLSIFGTRPEVIKFYPVLKEMDRRSLLSITCVTGQHRQMI--------DPLLSLFDIATD--IDLNLMTQGQTLSNLS
Spizellomyces_Fungi_punct         VMIVMGTRPETIKMFPIIRALNTHI-NPIICVTGQHQEMV--------EPLLRLFDIKPH--VNLNVMKSGQSLNSLT
Paulinella_chromato               VSIVLGTRPEAIKLAPVIMSFQKCALRTRTILTGQHREMV--------NQVMKLFQLSFD--KDLALMEPNQTLTHLT
Cupriavidus_sp                    IICVVGTRPEAIKMAPLINSLKNEWVECKVLATAQHREML--------DQVLGLFDICPD--IDLNIMRPNQSLVELT
Talaromyces                       IAFSSNTRADWVKLQPLADILV--GFDVDIFVTGMHMIKEYGLTFKNIEK--HTKFGVFT-RNTWAPGDSEIQNA---
Gymnopus_Luxurians                LLFITGTRADWGKLEPLVKKAV--GSPTDIFVTGMHTLKDYGRTETEIRK--LPGIRICT-YVNHSAGDDPTTIL---
Ganoderma                         LLFVTGTRADWGKLKPLAEAAA--GFLTDFFVTGMHLLEDYGMTEKEIRK--LPNVQICA-YPNHRAGDAPTTIL---
Sinorhizobium_meliloti            LLFLTGTRADFGKIEPLAVAAS--GYAINFFVTGMHMLEKYGATKHEVHR--LEGVEVHE-FINQRLGDPQDLIL---
Rhodobacter_capsulatus            ILFVTGTRADFGKIEPLALAAR--GFKVSFLVTGMHMLDRYGLTKIEVHR--VQGATVHE-FLNQREGDPQDTIL---
Photobacterium_sp                 ILFLTGTRADFGKLKSLINKVE--ELEAHLFVTGMHMLSQYGMTSIEVEK--SGFKNIYK-YINQNSHDSMDSIL---
Sterkiella_Alveolate              ILFLTGTRADFGKIKSLISILE--EFEVFVFVTGMHLQEEYGYTLIEIER--CNFKNIHT-FVNHTHETTMDLTL---
Arcobacter_marinus                IVFLTGTRADFGKIKSLIKITQ--LFDVHIFATGMHMIAKYGKTIIEIEK--SGFKNIYP-FINHDDIDHMDRNL---
Termitomyces_sp                   ---MTGTRADWSKMQPIARGI---FACLDILSTGMHEDGQHGNTGNDIEGSVQGVRQYHR-RPNHLQGSDPVNAG---
Ensifer_sp                        -------------MKVPARAFLNAGTFLDIFITGMHMLPEYGLTKNEVLG-LEGVREFHE-YDNHAQGPDQVDVA---
Chromera1                         ISIVVASRANYGRIKSVLRAIEDHDLELSIIVAASALLYRYGEVVERMRE--DGFEIAAQ-VYSIVEGENPTTMAKST
Polaromonas_sp                    ICVVVGSRANYSSIKAVMRAVAAHALELQVVAGASALLDRYGAVVDIMEK--DGFTIDAR-LFMLVEGETPATMAKST
Chromera2                         ICVVIGSRANYSSIKSALRAIDQHELELQLVIGASALLDRYGSVVDLIEA--DGFRADAR-VHMLIEGETPVTMAMST
Rhodopseudomonas_palustris        ICVVVGSRANYSSIKSAMRAIQDHALELQLIVAASAVLDRYGSVVNLIEK--DGFRPHAR-VTMLIEGETPATMAKST
Bflo_XP_002588099.1               VCVATCNRADYSKLAPVMFALRDDM-ELQVVVMGSHLIDDYGSTYRMIQQ--DEFEVDGY-LHTIVRGEDEASMAESV
HSA_GNE_AKI72325.1                VCVATCNRADYSKLAPIMFGIKTEFFELDVVVLGSHLIDDYGNTYRMIEQ--DDFDINTR-LHTIVRGEDEAAMVESV
Drer_NP_957177.1                  VCVATCNRADYSKLAPIMFGIKSHIFDLEVVVLGSHLIDDYGNTFRMIEQ--DDFDIGSK-LHTIVRGEDEAAMVESV
Micromonas_commoda                ICVVTSNRSDWSKLKLVAINLRKLDIQVDIICLGSHLLHELGATKNIVKE--DFPNA-YE-LHTLVAGDSVESMTDSV
Chromera3                         VCVVTSTRADYSKLRGLMLALEKSLFELVPLVIGSHLLIESGYSFRLVEK--DFAKSATK-VSTVAAGNDTNNMADSV
Paramecium_biaurelia              VGVLTSSRADFGIYLPLLKALQKEAFHLELIVFGTHLSKFHGYTVEQIER--EGFKIAKR-IESLLLGDTPSAIASAY
Prorocentrum1                     VMFVTGSRAEYDILYPVIEAVSRSSLRPEIVVTGSHLASVYGLTVKDVEA--DGYPVVAK-IDNLLASDSDAARAKSA
Condylostoma_magnum               IAIITTTRADFGLLRFLMTKIANDDLTLQTLATGTHLSKKHGLTKHEITK--AGLIINEE-IFMDLDDDRPLALSKSV
Prorocentrum2                     ------TRAEYGLLYWLLKDIQADELQLQLIVSGSHLSPEFGLTYQQIED--DGIVITEK-IEMLLSSDSAVGTVKSM
Vibrio_cholerae                   VAVFTGTRAEYGLLFWLLKDIQADELQLQLLVSGMHLSPEFGETYQQIER--DGFHIDEK-IEILLSSDSAVGTAKSM
Bacteroides_fragilis              ICVITGSRAEYGLLSGLMAKIQFDDLQLQIIATNMHLSPEFGLTYKEIET--DGFRIDKK-VEMLLSSDTANGTAKSV
Campylobacter_concisus            ICVVTSTRAEYGLLYWLLKEISADELELQIIATGMHLSPEFGLTYKEIEK---EFNIDKK-VEILLSSDTSIGISKSM
Nonlabens_marinus                 ICIVTGTRAEYGIMYWLIKFLQEDDIDLQLIVTGMHLSPEFGSTYKEIEG--VGFKIDKK-IEMLLSSDSEVGISKSM

Dictyoglomus_turgidum              GRMLIGIEEVLIKEK--P-DVVIVYGDTNSTLAGALASSKLHIPLAHVEAGLRSFN--KK
Naegleria_excavate_gruberi         TLAFQKISLEFEKQG--PISMVLVQGDTTTSLVAALAASYLKIPVGHVEAGLRSYDFNSP
Ostreococcus_lucimar               SSAVRAVDGVLRSSK--P-DAVLVQGDTTTAFITSLAAFYLKIPVGHIEAGLRTRDIYSP
Capsaspora_owczar                  ARLFTALAPVFEAVR--P-HLVLVQGDTTTAMIAAMCAFYYRVPVGHIEAGLRTNDRYNP
Spizellomyces_Fungi_punct          ERIVGRMTKAVTHYR--P-DWLLVQGDTTSAFVASVVAFHEKIAVGHVEAGLRTYKRYSP
Paulinella_chromato                ATILEGLRKEFLVHR--P-SLVLVQGDTTTALASAMAAFYEQIPVGHIEAGLRTNDIDDP
Cupriavidus_sp                     SRLLVEVDRVLSQEA--P-DAVIAQGDTTTVMTVALACFYRGIPFGHVEAGLRTWDMRNP
Talaromyces                        TVTMEATFQLLQQAD--Y-DLLIVHGDRLEAKAAADAAHLFRCRLGHVEGGELTG-----
Gymnopus_Luxurians                 SNTLLSLRNWVQQFR--P-DMLVVHGDRVEAFAACIVAAQMPLPCAHIEGGELSG-----
Ganoderma                          SNTLTGLRDWVPQNK--P-DLLIVHGDRVEAFAACTVASQLSLRCAHIEGGEVSG-----
Sinorhizobium_meliloti             SKTILGVSDWVYEHK--P-DLVIVHGDRIEALAVSLVCATNYIRCAHIEGGEVSG-----
Rhodobacter_capsulatus             AKSIIGFSDIFAELR--P-DLVVFHGDRIEALACALVCATNYIRSAHIEGGEVSG-----
Photobacterium_sp                  AKTIHGLSDYVKELK--P-DLIIVHGDRVEAMAGAIVGSLNNVLVGHIEGGEVSG-----
Sterkiella_Alveolate               AKTIEGFSAYCNTVK--P-DMIVVHGDRVETLAGAIVGSLNNILVTHIEGGEVSG-----
Arcobacter_marinus                 AKTIDGFSHYILEIK--P-DLIVVHGDRIEAMAGAIVGSLNNILVTHIEGGEISG-----
Termitomyces_sp                    IETMKIIADLTREHG--Y-TDIFIHGDRIEANYSSLVALSLNMRIHHIEGGEITG-----
Ensifer_sp                         EETIKHLKKVIKREK--I-TDMFIHGDRVEALACATAARLLNVRVHHIEGGEITG-----
Chromera1                          GLGVIELSTLFENLK--P-DIVLTVADRFETMATAIAASYMNIVLAHTQGGEVTG-----
Polaromonas_sp                     GLGLIELPTIFERLQ--P-DVVLTVGDRFETMATTLAAAYMNIPLAHTMGGEVSG-----
Chromera2                          GVGMIELPQIFSRLD--P-HVVLTIGDRFETMATTLAAAYINIPVAHTMGGEVTG-----
Rhodopseudomonas_palustris         GLGLIELPTLFEQLG--P-DVVLTVGDRFETMATTLAAAYMNIPVAHTMGGEVSG-----
Bflo_XP_002588099.1                GLALVKLPDVLVRLK--P-DLLIVHGDRFDALSLATCAALMNVRILHIEGGEVSG-----
HSA_GNE_AKI72325.1                 GLALVKLPDVLNRLK--P-DIMIVHGDRFDALALATSAALMNIRILHIEGGEVSG-----
Drer_NP_957177.1                   GLALVKLPDVLQRLA--P-DILLVHGDRFDALALATAAALMNIRILHLEGGEVSG-----
Micromonas_commoda                 GFGIVKLTSLLCALK--P-NIVLVHGDRFDAFCAAIAANMLNLTIAHVEGGELSG-----
Chromera3                          ALTILKVSQFLALAK--P-KIVIVHGDRFDAFGVASASTLLQIFTVHLEGGELSG-----
Paramecium_biaurelia               GLTALKFAEYWQENEQSF-DVVFALGDRFEMAAAVTASIPFGIKIAHLHGGETTLG----
Prorocentrum1                      AIQLQGLVDVVAYRR--P-DFLVVVGDREEAITVALTGGYLDIPVVHIAGGDTADDF---
Condylostoma_magnum                GIGLMGFAEAYDRLK--P-DIIVVLGDRFELISAVTPAFMANIPIAHLHGGEITAG----
Prorocentrum2                      GLALIGLADTFARLQ--P-DVLVLLGDRFETLAAAQSAMLMKIPVLHLHGGEITEG----
Vibrio_cholerae                    GLGVLGFADALARLK--P-DVLVILGDRFEALAAAQTAMILRIPVIHLHGGEITEG----
Bacteroides_fragilis               GLATIGFADAFEDLS--P-DLILVLGDRFEILAAVSAALFYKIPVAHIHGGEITEG----
Campylobacter_concisus             GLAQISFSEAYEELN--P-DIVVVLGDRYEIFSATSAAMIARLPIAHIHGGEATEG----
Nonlabens_marinus                  GLAQISFSEAFQDLA--P-DVILVLGDRFEIFSAVAAAMIARIPVAHLHGGEATQG----

Dictyoglomus_turgidum              MPEEINRIVADHLSDILFAPTETAVENLKREG-IE-KGVYLVGDIMFDALMHFSKLAKEK
Naegleria_excavate_gruberi         FPEELNRKVIDSFASLLFAPTEFSKAALVREG-ICPQHVHVTGNTGIDSFYSLKSLPPKI
Ostreococcus_lucimar               FPEEVNRQCISVMATYHFAPTEHAAKNLYDEG-RR-TNVFTTGNTVTEPSDRVIELSKVV
Capsaspora_owczar                  FPEEVNRRIVSVVGNLHFAPTNYAAEALRAER-IDMQTVFVTGNPVIDAILLIREKAPSA
Spizellomyces_Fungi_punct          FPEETSRRLIAGLASYHFVPTQHAANNLKAEG-IPSENVYITGNTVIDALQWVASLEPSP
Paulinella_chromato                FPEEGNRCLISQITKLHFSPTIQSAANLQASG-VI-GEIHITGNTVIDALLSMSQKKS--
Cupriavidus_sp                     FPEEMNRVIAGRIATWHFAPTESSRGNLLKEG-VPEREIVLTGNTVIDALLDVVTRCD--
Talaromyces                        GDDNKNRYAITAVADYHFPSSQDAADRLICCG-QRIETIFPIGSPDLDVFLRPSAIPIDH
Gymnopus_Luxurians                 SIDEIYRHCNSKLSTYHFVSSETAKRRVTSLG-EDPSTIFNIGSPELDSHFGLSGVTIDE
Ganoderma                          SIDEIYRHCNTKLSTYHFVSSETAKRRIISLG-EDPRRIFTIGSPELDDHVRENGVTIGE
Sinorhizobium_meliloti             TIDEIYRHCNSKLCTYHFVSSEIARARVLALG-EDPQFVYNIGSPELDLHRGDSGVTLSK
Rhodobacter_capsulatus             TIDEVFRHCNTKLAACHFVSSEAAAKRVMTLG-EPADRIHVIGSPELDFHARPSGVTLPE
Photobacterium_sp                  TIDELIRHSVSKMSHIHFVCNDKAKQRLIQLG-ELESSIFVIGSPDLDIMTSTSLPNIQK
Sterkiella_Alveolate               TVDELIRHSVSKLSHIHFVSNQEAAKRLIQMG-EVKESVFTIGSPDIDIMFSDKLPDLNT
Arcobacter_marinus                 TIDELIRHSISKLSHIHLTSNEQAKKRLIQMG-EDRNCIFNIGSPDLDVMNSKNLPTLKV
Termitomyces_sp                    SVDNALRYSVSAHAHFHYVCNEEAKNRLIRCG-NNPATIFVIGSPEMDRHAQTGERSLEE
Ensifer_sp                         SVDNDIRYAVTALSRFHYVCTEDAQQRLIATG-QPAETVFNVGSPEMDAHAVVGSLTLDQ
Chromera1                          SIDESVRHAITKLSHLHFVTTDLSRQRVLQMG-EDPDAVFITGCPSIDPIASIDLS----
Polaromonas_sp                     NIDENIRHAITKFATIHFPASEDARNRIIKLG-EQPDTVHNVGCPRMDLVAGFLR-NETE
Chromera2                          TIDESIRHAVTKFAHIHFPASRDAAERIEKLG-ERPEMIFQVGCPRIDLVAEVLERDEAD
Rhodopseudomonas_palustris         TIDESIRHAVTKFAHIHFPASQGAAERIIKLG-ELPRHVHMVGCPRIDLVAEILGRSSGG
Bflo_XP_002588099.1                TIDDSIRHSITKLSHYHACCTDRARRRLLSMC-EDNDHILLAGCPTYDKLLSCN-AK---
```

```
HSA_GNE_AKI72325.1                      TIDDSIRHAITKLAHYHVCCTRSAEQHLISMC-EDHDRILLAGCPSYDKLLSAK-NK---
Drer_NP_957177.1                        TIDDSIRHAISKLAHYHAVCTLSAERHLISMC-EDHSRILLAGCPSYDKLLSAYKRD---
Micromonas_commoda                      TVDGTLRHAITKLSHLHFTCTPEAARRIRGMG-ENPASIFVTGCPSYESLFAVSATCWED
Chromera3                               TIDGKLRHSISKLSDLHFTCTEAARMRVIEMG-EENSAVFCTGCPSYDVFLEMEHIGSGH
Paramecium_biaurelia                    AIDNIYRHSISLASQLHFVAAEPFAKKLKQLLDDEKASIYNVGSLSLENLKNIDFLSVEA
Prorocentrum1                           NIDNSVRHAVTKLAHLHMTASAESAERVLRLG-EEPWRVHNVGAPGLDRLVGEPEIALAC
Condylostoma_magnum                     AFDDSIRHAISKMSHLHFVAHDDYKARLCQLG-EQPERIFTVGGMGVDALGHLQFLCKDE
Prorocentrum2                           ATDDAIRHAVTKLSYLHATSTEAYRQRVIQLG-EAPERVQNVGAIGLDHLYRSTLLTKEQ
Vibrio_cholerae                         AYDDAIRHAITKLSYLHGTSTEAYRQRVIQLG-EAPERVKNIGAIGLDHLKRAQFMDVPA
Bacteroides_fragilis                    AYDDNIRHAITKMSHLHFTSAEPYRRRVIQLG-ESPDRVFNVGALGVENIKHVPLLSKEE
Campylobacter_concisus                  LIDEAIRHSITKMSHLHFTATNEYKNRVIQLG-EDPDRVYNVGGMGIENIKRLKLLNKKE
Nonlabens_marinus                       LIDEPIRHSITKMSQLHFTATEVYRKRVIQMG-ENPSRVFNVGSPGLDNIHRLTLLDKRQ

Dictyoglomus_turgidum                   --LEKLSLYPKGYYLVTIHRAENTDNPERLKSIFSALQ
Naegleria_excavate_gruberi              KHVDATTSSHRTVILVTMHRRENIP-H--FKEMCNAIK
Ostreococcus_lucimar                    --KTVSTLRDVRLLLLTAHRRENLGEP--ILNIFTSIE
Capsaspora_owczar                       TNATLPAGQKARHILLTAHRRENHGEG--IAAICRAVK
Spizellomyces_Fungi_punct               --AAKSHTSQYRLLLVTMHRRENLGSP--LISVCKAIK
Paulinella_chromato                     --INGLNWKDKRVILATVHRRENWGTR--LNGIAYGFF
Cupriavidus_sp                          ----PNIPQGRRLILVTSHRRENFGEP--FEEICRAIR
Talaromyces                             LHKYGI-PFK-DFGIAPFHPNSAESET--SGEQAMNFY
Gymnopus_Luxurians                      KTSYGI-SFT-EYAMVTFHFVTSETET--IGDQAASLF
Ganoderma                               KHKYGI-PFT-EYGIVTFHSITSETDT--IGDQARHLF
Sinorhizobium_meliloti                  RDRYAI-PFS-EYGIVTFHPVTSETDT--IGAQARSLF
Rhodobacter_capsulatus                  MTRYDI-PFD-DYGVTLFHPVTSEAAT--MGRQAADLF
Photobacterium_sp                       KLRYNI-PYD-DYALFMYHPVTTDVKN--LKEKIRKVI
Sterkiella_Alveolate                    KEYYKI-PFE-NFSIVMFHPVTTEIEN--MKQYAENFV
Arcobacter_marinus                      KKYYEI-DFK-EYAIVMFHPVTTEMVK--LKKQIKTFL
Termitomyces_sp                         KRHHKI-SYD-KYGIASLHPVTTVDPT--VTLEEALY-
Ensifer_sp                              KELHGI-PFTGQFGIVSFHSVTTEQQF--MANDAANLF
Chromera1                               GSGPTL-DPRKPYVVVLQHPVTTEFGQ--GFEQINETL
Polaromonas_sp                          GVGSSF-DVKGDFLLVSQHPVTGEYGS--GERQVAATL
Chromera2                               GVGDRL-DLSGPFVVMSQHPVTTEYGE--GERQITETL
Rhodopseudomonas_palustris              GVGQQF-SVDEPFALVSQHPVTTEYGT--GEAQITLTL
Bflo_XP_002588099.1                     WVGEGV-K-PKDFIVALQHPVTTDIKG--SLKMFDLML
HSA_GNE_AKI72325.1                      WLGDDV-K-SKDYIVALQHPVTTDIKH--SIKMFELTL
Drer_NP_957177.1                        WIGDDV-K-EQDYIVALQHPVTTDIKN--SIKIYELML
Micromonas_commoda                      GTPFKL-K-PNKFILVIMHPVTNDLEE--SNTLYGSLL
Chromera3                               -VTHRV-V-PHEYFIIMYHPDTCSIQD--TVKQYRSIL
Paramecium_biaurelia                    KNKWQI-DLNIKTILVTVHPETVAYQK--NLAYCEETI
Prorocentrum1                           WKMMKVAPVEGPFLIMIQHPLLPEMHD--AERQMKATL
Condylostoma_magnum                     EKSLDF-TFLKRNLLITHHPVTVELGK--ARQELKALL
Prorocentrum2                           AQALDF-KITRPYFVVTYHPVTLADEP--PLESFNALL
Vibrio_cholerae                         AQSLNF-ALTQPYFLVTYHPVTLGEEA--PEVSFQALL
Bacteroides_fragilis                    EKSLNF-QLGDKSLLVTFHPVTLENST--AEQQCMNLL
Campylobacter_concisus                  EESINF-KLNKKNLLVTFHPVTLENDT--AEEQFQALL
Nonlabens_marinus                       EKSIDF-KLNQKNILITFHPVTLEKNT--SKKQFKALL

Dictyoglomus_turgidum                   E--------LDKEVIFPIHPRTKNKVKEFGLE-------DYLKSRIRIIDPVGYLDMIQL
Naegleria_excavate_gruberi              TISNTFG--KNVMVILPVHPNPNAKSVVHEV--------LSSLDNVQLVDPIAYDIFPHV
Ostreococcus_lucimar                    KLLQEY---PDVVVIYPIHLNPMSDHA-PPT--------TTHLRRLLIVPPLDHADLLFM
Capsaspora_owczar                       RIIAQH---KDVNVWYPVHLNPAVCVPVRQE--------LGKVERVHLLDPLEYDVFVHF
Spizellomyces_Fungi_punct               RIVDGF---PDVHIVLPVHMNPTVQETVTQF--------LGNDKRITLLGPLSYEFFAQL
Paulinella_chromato                     KLLERH---SDVALLLPLHKNPAVRKPLNDI--------LKDHPRAFLTEPLAYDQLVAA
Cupriavidus_sp                          TLVENN---SDVEVLYPVHPNPNVKETAHRM--------LSGLDRVTLTDPLDYLPFVAA
Talaromyces                             STLLASG--RNFVVPRPN-NDKGTENVQEVLDSLP-------SDRVCVVPNFEFNDYVVL
Gymnopus_Luxurians                      DCLTNSG--KNFVVISPN-NDPGTEKIGDVISRLD-------PSRFHRVSSLSFNDFSIL
Ganoderma                               DCLSNSG--KRFVVIWPN-NDPGTEKIHAVISALD-------PSRFHGVSSLSFDDFSTL
Sinorhizobium_meliloti                  TTLAESG--KNFVIISPN-NDPGSDDIFAEISKLD-------PHRFRHLPSMRFSYFSEL
Rhodobacter_capsulatus                  GALEASG--KNFVVIAPN-NDPGSREIFAVLEQLP-------RERFRLIPSMRFAHFSEL
Photobacterium_sp                       DGMISTD--DNYVIIYPN-NDHGSEIIIQELERLR------NNPRFRIFPSIRFEYFLTL
Sterkiella_Alveolate                    HALLKDN--HNYIVIYPN-NDLGSRFVLDSYDKLK------SNERFRVFPSLRFEYFLTL
Arcobacter_marinus                      NSLIKSD--LNYIVIYPN-NDMGSNIILEEYKKIT------NNNKFKIYPSLRFEYFLVL
Termitomyces_sp                         FALRASG--KSWIVIKPN-NDPNSEQILLALEKVLEEDQKSAQPQFFVTDNIPFDDFSTL
Ensifer_sp                              MGGGKSI--KPFLVIMPN-NDPGTDDIRRIIEIIFDDR---DSELFFRAANIPFEHFSVL
Chromera1                               AAVHALG--LQTVWLWPN-IDAGSDDVSKGLRMFRTRH---HKAPIHFFRNFGVEDYARL
Polaromonas_sp                          GACLELG--MQTLVLWPN-ADAGSEDVSRGIRKFREQH---PDAPFRYVKNLPPEIYMPL
Chromera2                               HAVRQVG--P-VIALWPN-PDAGSDDIARGLRKWREQG---KAENMHFFKNLPIDVYIRL
Rhodopseudomonas_palustris              EAVREQG--LAAIVLWPN-ADAGSDDISRGIRKWRERK---LDDRMHFFKNLPIETYVNL
Bflo_XP_002588099.1                     DALLEFN--VKTLVLFPN-IDAGSKEMTRMIRLR---G-LEHHPNFSLAKHIPYDQFIIL
HSA_GNE_AKI72325.1                      DALISFN--KRTLVLFPN-IDAGSKEMVRVMRKK---G-IEHHPNFRAVKHVPFDQFIQL
Drer_NP_957177.1                        DALISFN--KKTLILFPN-IDAGSKEMVRVMRRK---G-IEQHQNFRAVKHVPFDQFIQL
Micromonas_commoda                      SCLFSRK--TPTVMFYPN-VDPGNKSMIQTLHKHQKAD-PASTSWLRLVTHMPHAKFTAL
Chromera3                               EAVDRTG--ERAILFYPN-IDPGSKDMIQTLHEIQKTS-PTFSKNVNCMTSLPHEEFIIL
Paramecium_biaurelia                    KALEQLANEFQIVITMPN-ADTAGMVFRELAK-----K----QQSIKIIENFGTQSYFTC
Prorocentrum1                           DAVVACG-L-PTFVSSPN-SDPGNQAMSRLLSDYANRY----P-KLVAYKNLSRAVFVNL
Condylostoma_magnum                     HALDELK-ETGLIFTMPN-ADPDSDALALMIKNFAKD-----KAHVKCFASMGQLNYLST
Prorocentrum2                           SALDQFK-QYQIILT---------------------------------------------
Vibrio_cholerae                         DALDTYP-EHQIILTYPN-ADDGGRRIIPMLEAYAAAN----PQRVLAIPSLGQVRYLSA
Bacteroides_fragilis                    ESLESFP-DYRIIFTFPN-SDTDGRIIIDLIKRFVERH----RSESVAFVSLGKVRYLST
Campylobacter_concisus                  NAIDELE-NTNIIFTKAN-SDTNGRIINLMIDRYVSNN----QDKSVCFASLGQLRYLSA
Nonlabens_marinus                       HSISNLE-DTSFIFTKPN-SDTDGRIIIQLIDEYVSKN----PKTSCAFKSLGQLRYLSA

Dictyoglomus_turgidum                   EKNAYAILTDSGGVQKEAFWLKVPCITLREETEWVEIVKLKWNRLVGANK--EKILEAVR
Naegleria_excavate_gruberi              ISEADIVVTDSGGIQEESASIGKPVILMRDTTERPEGIYIGTIKKIGVNY--HHIVKAMT
Ostreococcus_lucimar                    MKESFFVMTDSGGIQEEAVTLGKPVLVLRDTTERPEGVLAGAAKLVGHGA--ESIYTEAA
Capsaspora_owczar                       LDRMYMVMTDSGGIQEEVTALAKPTIVLRETTERPEGVDAGVTKLVGIHE--DTIVKEAN
Spizellomyces_Fungi_punct               LKRATLLLTDSGGVQEEGTAFSKPILVLRNDTERPEGVTAGVAKLIGTDE--DNVFRHVQ
Paulinella_chromato                     MRSCSLVLTDSGGIQEEAATFGKPVLILRRTTERSEAVEGGTARLIGTDT--TDILNEAS
Cupriavidus_sp                          MRGAYLILSDSGGVQEEAPALGKPVLVLRRETERPEAVDAGVVKLVGPDH--ARIVEEAQ
Talaromyces                             LKQAGCVAGNSSVVVTSAPAIGKPCLNIGSRQQGRTP-PTNGLFNFSPND--RHGILRCL
Gymnopus_Luxurians                      MKDCKAVIGNSSAGVRETPFLGVPSLNIGTRQQNRAE--ADSITHSSAFD--TATIQKFL
Ganoderma                               MKSCKAVIGNSSAGVRETPFLGIPSLNVGTRQRNRAE-DVPSITHCSAFD--TPRIEEFL
Sinorhizobium_meliloti                  MKNAKAIIGNSSAGVREAPFLGVPSLNVGTRQNNRSS--AGTITDASAFD--TDTIREFL
Rhodobacter_capsulatus                  MKHAACLVGNSSAGVREAPFLGIPSLDIGTRQTNRAE--APSLFSADAAE--REKIAAFL
Photobacterium_sp                       LKNADYMLGNSSAGVREAPFYGLPSINLGSRQDNRSK--APSIINVLENQ--EQIYNAVV
Sterkiella_Alveolate                    LKNSQFIIGNSSAGIREAPYYGIPIINIGTRQQNRAI--HADIINVDYSE--KQIAEALS
Arcobacter_marinus                      LKNAQFIIGNSSAGIREAPYYNIPTINIGNRQNNRVK--SKTIKSIDFIE--KDITKAIK
Termitomyces_sp                         QANAAFAAGNSSAMNMDA-----------------------VINIKAFI--PDLIVHTI
Ensifer_sp                              QRNAALFIGNSSAGVREMPFHGVASINVGSRQDGRAP-DIQSIVTVAPND--NATMLKAL
Chromera1                               INNAVCLIGNSSSGIREGAFMGVPVVNIGTRQTGRER--GPNVIDVGYDR--GEIQAAAQ
Polaromonas_sp                          MARTRCLIGNSSSAIREGAFIGTPAVNIGSRQTARER--GQNVVDSGYTQ--AEILAAIR
Chromera2                               MRRAVCLVGNSSSGIREAGFIGTPVVNIGSRQAGREH--GENVLHVDYDR--GAIADAMM
```

```
Rhodopseudomonas_palustris             MRSAACLVGNSSSGIREGAYIGTPVVNIGTRQHMRDR--GDNVIDVGYDK--KQISDAIA
Bflo_XP_002588099.1                    LANTGCLIGNSSAGIREAGAFGTPVINLGNRQIGREA--GENVLHVRDADTKGKILHAVN
HSA_GNE_AKI72325.1                     VAHAGCMIGNSSCGVREVGAFGTPVINLGTRQIGRET--GENVLHVRDADTQDKILQALH
Drer_NP_957177.1                       VAHAVCMIGNSSCGVREAGAFGTPVINLGTRQTGRET--GENVLHVRDADTHNKIYHALE
Micromonas_commoda                     MRHASAMVGNSSAGIRESCVFGIPTLNLGSRQEGRRV--PANVTTLVKPS-IRSIDCWFD
Chromera3                              LKNCKAIVGNSSAIIREAAFFGTPAVNVGTRQQGRTC--PPNVFSVKGEN-SDEVLQALL
Paramecium_biaurelia                   MKYAKLMVGNTSSGIVEAASFQKYVLNLGDRQKDRLC--GENVIHVPFNH--DLIVKNTL
Prorocentrum1                          MRHASALVGNSSCGIIEAPLLKLPVVNVGSRQVGREH--AGNVEFVDYNA--TEIEAALK
Condylostoma_magnum                    MRVSDAVVGNSSSGICEAPSFHIGTINIGDRQQGRLS--ASSVIHCTGDY--QEIKQAIH
Prorocentrum2                          ------------------------------------------------------------
Vibrio_cholerae                        VKHASVVIGNSSSGIIEVPAFDVPTVNIGVRQQGRLA--AKSVLHCRVSK--EEIEQAIQ
Bacteroides_fragilis                   LQYVSAVVGNSSSGIIEVPSFGIPTLNIGDRQKGRIS--SRSVVHCGTSK--EDICLGIQ
Campylobacter_concisus                 LQYVDAVVGNSSSGLAEAPSFKIATINIGDRQRGRIK--ASSVIDCQADK--NSILKAFD
Nonlabens_marinus                      LKYVDVVLGNSSSGLAEAPSFKVATIDIGDRQKGRIK--ADSVISCDPTE--ESISQALQ

Dictyoglomus_turgidum                  NIKEGEDVN---------FEENYSAPKMREILVREL
Naegleria_excavate_gruberi             DAIKDSQNS-KLILSKHIFGDGKASMRISKIVRDFV
Ostreococcus_lucimar                   SLLKDPDSYRSMSGSKKTYGDGNAAGNIVAKEKQKE
Capsaspora_owczar                      VLLTDEQTFAKMSCKCFPFGDGTAAKQIVDIMLMSV
Spizellomyces_Fungi_punct              ELLTDKQAYAAMAIKTFPYGDGTAGSKIVDIVLKGS
Paulinella_chromato                    LLLKDDSAYRAMSQFNNPFGDGKASDRILTAARTFL
Cupriavidus_sp                         RLLDDASAYQAMARGVSPYGDGKASQRIVKVLREYF
Talaromyces                            RENWGKT-----YPSNTHYGDGCAAERFFEALSSRS
Gymnopus_Luxurians                     ETKWGKR-----FDPDYAFGRGNAAQQFVEVLGREG
Ganoderma                              DSKWGQR-----FRPHHGFGRGTAAQEFVGTLNVDE
Sinorhizobium_meliloti                 NTTWGRK-----FESDQSFGNGNAASRFVQVLNDDT
Rhodobacter_capsulatus                 ATEWGKR-----YPPHTAFGEGRAAERFLEVLADEG
Photobacterium_sp                      DAKKS-K-----FEPTKEFGQGDSDLRFIEIINNKD
Sterkiella_Alveolate                   IIDSHKV-----QKSDDDFGQGNSAELFLSCLQKSD
Arcobacter_marinus                     DALLTKK-----VTKDVDFGNGNSDKKFYNLLQKNT
Termitomyces_sp                        KVKWDKK-----FERDTTFGTGKAEETFTQILAAPG
Ensifer_sp                             EEGWGKK-----FPRDTTFGDGECEERLTQIFKTAG
Chromera1                              RQIDHGR-----YPENPLYGDGQAGR-IADLLAQ-A
Polaromonas_sp                         DRLKTGR-----LPSNPIYGDGHAGPRIAEILAT-A
Chromera2                              QQQRNGR-----WESQSTYGDGRAGMRIADTLASLG
Rhodopseudomonas_palustris             RQVEHGR-----YAMDPIYGDGTAGTKIADILVT-E
Bflo_XP_002588099.1                    -LQYQKQ-----FPCSYIYGDGHAVQRIMKFIHKIR
HSA_GNE_AKI72325.1                     -LQFGKQ-----YPCSKIYGDGNAVPRILKFLKSI-
Drer_NP_957177.1                       -LQFGKR-----YPCSKIYGDGNAVQRILKFLQTI-
Micromonas_commoda                     -NELGKR-----YAQSTMYGFPDSAKRIAHHLSRID
Chromera3                              -EHPKER-----FDRDTTYGDGHAVSRMMKHLEGV-
Paramecium_biaurelia                   ------EYATKEYNGENIYFKSNPSEIIIQILK---
Prorocentrum1                          KAVFDDQYRAQVEVAKNPYGDGTAGKQICDVLVQET
Condylostoma_magnum                    K-LYSHEFQDKIKQTINPYGQGNASTQIHEILKSHP
Prorocentrum2                          ------------------------------------
Vibrio_cholerae                        T-AITRGYKADGDVIDNPYGQGDSSAQVIAMIKSLH
Bacteroides_fragilis                   T-VLSDPVVRIAKQKRNPYDKKGTADAICTILKTVS
Campylobacter_concisus                 K-VYSYDFQQVLKNVVNPYGDGCASKKIIEVIKRVD
Nonlabens_marinus                      L-AFSKHFQESLKNVKNPYGEGGASEKIVKILKDFD
```

5 – MSA Sialin

```
MFS
Dictyostelium           -----------------SIDTSTLSNSIASLSNSIEAPI
Emiliania               SARVTVLLLCCAIGAVCALDRVLISIA-------I----
Cryptosporidium         ISCISLVSMLMFSIFLCYADRIIMPSC-------I----
Aureococcus             EPGGTLVLLGAVVMLFVSVHRSLFSVG-------I----
Enterobacter            KARYLLVGVAFTLTALLYIDRVAISVA-------K----
Chromera                -----------------------LSVA-------K----
Cellvibrionaceae        PLRYWIVVATFLLSVLLYIDRICVSVA-------K----
SLC17A9_HSA             PECQAWTGTLLLGTCLLYCARSSMPIC-------T----
Hydra2                  ------------------CVRTSMSIC-------I----
Chrysochro1             ---------------------------------------
Micromonas              SYRFVVVALIALALLLCNADRVVMSVV-------G----
Bathycoccus1            NYRFTILFLVALALLLCNADRVIMSIA-------G----
Amphiamblys             RVRHVFALLCSVGIIIAYADRGNMALS-------I----
Bathycoccus2            KKRWAMVFSLFVAFVLCNLDKVNMSVA-------I----
Guillardia              PKRFILSLLLHLGLFVVYALRVCISVA-------AAAPS
Basidiobolus            ---------------------------------------
Congregibacter          PKRHVLVGFCFLATFICYIDRVNISVA-------I----
Sphingorhabdus          QKRHQLVLMAFLAIFICYMDRVAISVA-------I----
Sphingomonadales        QKRHQLVLMAFLAIFICYMDRVAISVA-------I----
Thelohanellus           ------------------------------MAKERE---
Chrysochro2             GRRFTISLLCATAAAISYAQRYGIAIAIVRMQ-------
SLC17A1_HSA             SFRYGLSFLVHCCNVIITAQRACLNLTMVVMVNSTDPHG
SLC17A3_HSA             SARYGIALVLHFCNFTTIAQNVIMNITMVAMVNSTSPQS
Strongylo3              STRYLVAYLACLGFLTAFMGRAALSVSMTAMVNSTIDHE
Strongylo5              SCRYIVSYISFLGIACAFMNRVNISVTMTAMANSSYSST
Strongylo1              STRYFVAVMACLGFASSYGSRVNMSVAIIAMANRSYSTD
Ciona1                  RQRYVLASLGCMGYFLISCLRNCINVTVLSMVRWNNTET
Drosophila2             EARTVLWYMTFMGFIVNYMIRINLNITIVDMIVGKGAIS
Strongylo2              PKRYELLFLAWLGTAMMFVMRINVSVTMAVMTNATFTTQ
Drosophila1             PKRVNLAIMLFMACLLSYMMRVNLSINIIAMVEDTSSHE
Drosophila4             SCRQVLNLLTMLGFMLNYALRVNLTIAIVDMVRPNVTSA
Drosophila3             PQRWILAIMGFFAIFNAYTMRVCLSQAITVLVVKRNHTG
Aplysia4                SQRLTLAMMVFLGNCVSYFARVNMSVAILCMVRSDVTVL
Aplysia1                SKRWILAYIGMVGFFFIYSLRVNLSVAIVCMVKDDVNKT
Capitella               SCRVTLALLTSVGYAFVYILRVDLSVAIVCMVKDPFINS
Hydra1                  SKRYVLAILSFFGFFNVYCLRVDLSVALVAMTNNHTRVM
Caenorhabditis          STRFALSLVMFFGCLVTYMMRTNMSFAVVCMVNENKTDT
SLC17A8_HSA             PKRYIIAIMSGLGFCISFGIRCNLGVAIVEMVNNSTVYV
SLC17A6_HSA             PRRYIIAIMSGLGFCISFGIRCNLGVAIVDMVNNSTIHR
SLC17A7_HSA             PRRYIIAIMSGLGFCISFGIRCNLGVAIVSMVNNSTTHR
Tribolium               KRRYVVAVLAFFGFFNAYALRANLSIAIVAMTENKTTVL
Aedes                   RRRYIVVFMAFFGFFNVYALRVNLSVAIVAMTEEREVVL
Amphimedon1             PARYILAVMSFFGFVIVYALRVNLSMAIVAMVGNSSNET
Amphimedon2             PARYTLAIMSFLGFVIIYALRVNLSMAIVIMVNNSANGS
Amphimedon3             PARYTLAIMSFLGFVMIYALRVNLSMAIVIMVNNSANGS
Oscar                   PARYVLAFMALLGFANVYALRVNLSVAMVKMK-------
Acropora                KCRYVLAALSCSGFCVIYLLRVNLSVALVAMVNSTYTNE
Exaiptasia2             -CRYVLAILSFWGMSVTYSLRVNLSIALVAMVNSTFANA
Nematostella            -VRYTLALLACLGFCVVYGLRVNLSVALVAMVNSTYDGI
Exaiptasia1             -CRHLLALLSSLGFCVVYALRVNLSVALVAMVNSTYENS
Aplysia2                SSRLGLAIIGFLGFINVYAVRVNLSVAIVCMVNQTAIRS
Trichuris               GWRHIVTILCFFGFFFLYSLKVNLSVAIVAMLNHTAIQA
Daphnia                 GTRHTLVLLGFLGFANVYAMRVNLSVTIVAMVNQSAIPH
Sialin_HSA              SARYNLAILAFFGFFIVYALRVNLSVALVDMVDSNTTLE
Aplysia3                SCRHILALMAFFGFVNVYALRVNLSVALVAMVNNTGTDK
Crassostrea             GSRHVLAFWAFLGFFNVYCLRVNLSVALVAMVNSTNNDP
Biomphalaria            AARHVLAIMAFLGFVNVYCLRVDLSVALVAMVNSSSS-S
Lottia                  NTRTVLAIMSFLGFVNVYCLRVNLSVALVAMINNTGLDN
Ciona2                  STRFCLAYLACFGFMNVYALRVNLSVAILSMVNSSYMEI
Strongylo6              SARHALAFLAFLGFVNVYAMRVNLSVALADMVNSTTTV-
Klebsiella_Nant         RAQWRAFSAAWLGYLLDGFDFVLIALVL-----------
Escherichia_nant        RAQWRAFSAAWLGYLLDGFDFVLIALVL-----------
Acyrthosiphon_sv2       ---------------------MPILLSL-----------
Fonsecaea_Nant          TQQWLFFLVAFFGWSWDAFDFFTVSLTV-----------
Calocera_nant           LLQWAMFFSGFIAWTIDAIDFFSVSLSV-----------
Hebeloma_nant           FTQWALYFSGWLAWTCDAIDFFSVSLSV-----------
Gymnopus_nant           LRQWAEFWTGWLAWSCDAIDFFSVSLSV-----------
Mycobacterium_Nant      GDQRNAFMASFLGWTMDAFDYFLVVLVY-----------
Drosophila_nant         ATARHVVAASFLAWMLDACDFFLVLFTL-----------
```

```
Amphimedon_sv2             KAQILIMLGVGLVLASDSVEVLGIGYIL-----------
Orbicella_sv2              RHQLRIYFVVSLISLPLAAQMLIVVFVG-AVPEWKCPSP
Strongylocentrotus_sv2     WFQVKISFIVGFNWMADAFEIMLLSVLS-----------
Homo_sv2                   KFQWKLSVLTGLAWMADAMEMMILSILA-----------
Daphnia_sv2                RFQVKLSLITGLSWMADSMEMMILSIIS-----------

Dictyostelium              --GG-RYSRFSGEIP---KYYIPRRLFLIVLGFYVMQIPAHFLCN--RF
Emiliania                  ---LPMAEQFAY--S---DSTKGAIAAGFSLGYCLGLLPAGVLAS--SG
Cryptosporidium            ---KSISEEFGF--N---KSDQGFILGLFYGGYIWTQIIGGYISDTSKL
Aureococcus                ---VPIQKELGL--G---ASTVGVLQSAYLAGYALTNAPGGAIAD--RL
Enterobacter               ---ESVTGEFGL--T---DTEFGWVLSIFALGYAIFQAPAGAMAD--KW
Chromera                   ---DPVAESLHL--S---DRQMGWAMSIFALGYALFQTPAGLLAD--KY
Cellvibrionaceae           ---ESIVSDLSL--T---DKEMGWVLSAFALGYALFQAPSGWLAD--RF
SLC17A9_HSA                ---VSMSQDFGW--N---KKEAGIVLSSFFWGYCLTQVVGGHLGD--RI
Hydra2                     ---SALSDELHW--D---KKTEGFVLTSYFAGYLLTNIIGGYLAD--FK
Chrysochro1                --------------D---AKIEGRLLSAFYWGYAVSQTPLGMLAQ--RI
Micromonas                 ---LPLANLNGW--S---PSVVGLVQSSFLWGYALTPLVGGVLAD--KI
Bathycoccus1               ---LPMSKMNGW--D---VKVLGLIQSSFLFGYALTPIFGGVLAD--EI
Amphiamblys                ---VPMAEEYRW--S---MVEKGSVLSGFFYGYVVTQVLSGWAAD--LF
Bathycoccus2               ---VPMAKSFGW--T---ATQKGLVASAFFWGYAFTQIPGGWLSS--KY
Guillardia                 VTGVSMYTEYSW--T---NTEQGIILGSFFNGYITTQIIGGILAR--RY
Basidiobolus               -----MSEEYSW--S---STTQGLILSAFFYGYICTQVLGGFLAD--KF
Congregibacter             ---IPMAEEFGW--S---ATTKGYVLSSFFVGYLLAMIPTGWLAN--KY
Sphingorhabdus             ---IPMVETYGW--D---LSTQGLVLSSFFIGYLLTQVVGGKLAD--RY
Sphingomonadales           ---IPMVETYGW--D---LSTQGLVLSSFFIGYLLTQVVGGKLAD--RY
Thelohanellus              TLIQIRTEKFEW--S---ESMQNYVLLAYNVGYMIGHVPGAFLSI--TF
Chrysochro2                -------SRLNW--S---RSIQGQILASFFLGYMVAQLPAGWAAA--RY
SLC17A1_HSA                LLDNIKNPMYNW--S---PDIQGIILSSTSYGVIIIQVPVGYFSG--IY
SLC17A3_HSA                L-----------------------------------PVDSFGG--LS
Strongylo3                 ASAKGQDGPFQW--S---SWTQELILASFYNGFPWLQLPAGYLAD--AN
Strongylo5                 NEENVREGEFPW--D---AHTQEQILAAIYYGFPVLQVPAGILAD--KY
Strongylo1                 TGGRSKEGEFPW--D---AHTQELILSAYYYGFVLLQVPAGILSD--RY
Ciona1                     NLTIEKSKGFEW--S---SSQEGLFLGAYFYGYVCTNILGGWLGN--KF
Drosophila2                ASIPYDRDGFNW--N---EKQQGALLGSFFWAHWTLQIPGGILAT--KY
Strongylo2                 DENGQDGGEFLW--S---SHEQELILAGFFYGYAAAQIPGGWLTD--KI
Drosophila1                EALPNYGPRYNW--T---QSDQALLLGAYFYGYMITSLPAGTLAE--ML
Drosophila4                DGVDVYEERFPW--D---SYQTNFVLGCFFWGYILTELPGGRLAE--LI
Drosophila3                APVSREGGDYEW--S---EEKQGLILASFYIGYIVTHLPGGVLAD--KF
Aplysia4                   DLLRDDTGEFSW--D---KGTQSKLLAMYFYGYIFTQVPGGWLAS--RF
Aplysia1                   PSNSTNNGEFDW--D---KDTQSLMLSAFFYGYIVLQVPGGWMAG--RF
Capitella                  SSPSPYKGEFEW--N---KEMRGIILGSFFYGYILTQVPGGWLTV--RF
Hydra1                     GTEYLVAPEFNW--S---TQLQGHILSAFYYGYLLTQIPGGYIAA--RF
Caenorhabditis             ESNSSVIGEFDW--D---KQTTGMVLSSFFYGYIGSQIIGGHLAS--RY
SLC17A8_HSA                GKPEIQTAQFNW--D---PETVGLIHGSFFWGYIMTQIPGGFISN--KF
SLC17A6_HSA                GKVIKEKAKFNW--D---PETVGMIHGSFFWGYIITQIPGGYIAS--RL
SLC17A7_HSA                GHVVVQKAQFSW--D---PETVGLIHGSFFWGYIVTQIPGGFICQ--KF
Tribolium                  GTTIQEPPEFDW--D---SKVQGYVLSSFFYGYITTQLLGGWLSA--KI
Aedes                      GTTVVE-QYFDW--T---TQMQGFVLSSFFYGYILTPFLGGFISN--KL
Amphimedon1                -----DHDHFNW--T---STQQGWILSAFFYGYIITQIPGGFLAG--VI
Amphimedon2                -----ANNQFNW--T---STEQGWILSAFFYGYVITQIPGGLLAA--RY
Amphimedon3                -----TNNQFNW--T---STEQGWILSAFFYGYTITQIPGGLLAA--RY
Oscar                      -------DEFGW--D---SSTRGIILGSFFYGYIVTQIPGGMLAT--RY
Acropora                   --SLQKDEQFNW--D---EKTQGLILGSFFYGYILTQLPGGWLGA--RF
Exaiptasia2                --THNKVGT---------DKYMWMILAAFFYGYIVTQIPGGWLAS--RF
Nematostella               --TTYKNGDFNW--D---QKTQGLILGSFFYGYICTQLPGGWLAS--QF
Exaiptasia1                --SSHTGGTFNW--D---QKTQGIILGSFFYGYIVTQIPGGWLAS--RI
Aplysia2                   SSDLAEMGSLEW--D---KTTQGTILGSFFWGYLFGQVPAGWFAT--KF
Trichuris                  -GQVKKDGPFDW--D---AATQGNILGAFFYGYIVTQVPGGLLAT--KF
Daphnia                    QSGLVKDGEFAW--D---EYKQGIILGSFFWGYVLTQIPGGRLAE--LF
Sialin_HSA                 VHHNQTGKKYQW--D---AETQGWILGSFFYGYIITQIPGGYVAS--KI
Aplysia3                   ATDGGKSGEFDW--D---PTTQGYILGAFFYGYIVTQLPGGWLAS--RF
Crassostrea                -TTSTNTGEFNW--D---SNTQGLVLGAFFYGYIITQIPGGWLAE--VF
Biomphalaria               STSPAKVGEFDW--D---ETTQGYILGAFFYGYIVTQVPGGWLAS--KF
Lottia                     TSPSTHPGEFHW--D---QTTQGVILGSFFYGYITTQLPGGWLAE--KV
Ciona2                     -HKNSTTGEFNW--D---AHKRSLVLGAFFYGYILTQLPGGYLAG--RF
Strongylo6                 -NTTQKEGEFNW--D---SNTKEQILASFFYGYILTQIPGGFLGD--IM
Klebsiella_Nant            ---TEVQGEFGL--T---TVQAASLISAAFISRWFGGLMLGAMGD--RY
Escherichia_nant           ---TEVQGEFGL--T---TVQAASLISAAFISRWFGGLMLGAMGD--RY
Acyrthosiphon_sv2          ---AILTK------S-----EAGILGSASLIGSALGGWSAGMLAD--RL
Fonsecaea_Nant             ---SDLAETFNK--S---NTDITWGITLVLMFRSVGSTIFGIAAD--RY
Calocera_nant              ---TALTKQFNR--S---TNDITTSITLTLLFRSVGALVFGMLSD--RY
Hebeloma_nant              ---TNLQTQFNR--S---THDITTAITLTLLFRSVGAVVFGILSD--RF
Gymnopus_nant              ---TNLSKQFGR--D---TTAITQAITLTLLLRSVGAVLFGIISD--RF
Mycobacterium_Nant         ---ADIATTFHH--T---KTDVAFLTTATLAMRPVGALLFGLWAD--RV
Drosophila_nant            ---DNVAHSFNT--S---LESVLLAPTLTLMTRPIGAYLCGRAAD--TY
Amphimedon_sv2             -QYLRLETEFGI--A---SWQVALLSSNTFVGMLIGGYIWGGLSD--IG
Orbicella_nant             -EFTSIATEWNL--ACTHRYKCELSQSIFVAGYMFGVLIFGILSD--KY
```

```
Strongylocentrotus_sv2      ----KLRCEWDL--Y---PYQQALLTTFVFTGYFIGAPLWGMMGD--KF
Homo_sv2                    ----QLHCEWRL--P---SWQVALLTSVVFVGMMSSSTLWGNISD--QY
Daphnia_sv2                 ----ALKCDWRL--P---DWKQALITTGVFLGMMMSAVFWGQLSD--KY

Dictyostelium               GG--KRVFLIGVTGSILCTIVLPPIAH-A----NQSALVLIRVLTGLSQGIAYPTMNWLM
Emiliania                   SP--RQVLLGGLVVWSAATCATPLAAA-S----SVPALLATRAVMGVGEAAAVPTLQAVA
Cryptosporidium             GG--KGVLFFGVTFWSLCMIFTSFLSY-M----GITGFIICRIFLGVGEGVSFPALNSIV
Aureococcus                 GG--APVMLACLAAWSVAVALMPVAAA-S--PAPVAALVALRLLFGLASGPALPGSLAVV
Enterobacter                GP--RKMLAIFVLIWSAFTGLTGLAWG-------FASLLVFRFLFGLAEAGAFPTFARAI
Chromera                    GP--RKILTAVVALWSIFTALTGAAWN-------YITLLIVRFLFGAGEAGAFPGMARAI
Cellvibrionaceae            GP--RKILTTVVILWSLFTALTAAAYN-------YMSMLIVRFLFGAGEAGAFPSISRVN
SLC17A9_HSA                 GG--EKVILLSASAWGSITAVTPLLAH-L-SSAHLAFMTFSRILMGLLQGVYFPALTSLL
Hydra2                      GG--ERVIFYSTFVWSFFTILIPALVN-SVVKFGFKTVIVCRFIIGLSQGMFFPSLSAIL
Chrysochro1                 NA--QHLLMLAVLVWSASSVGVALVGN-S--PEAVPAIFALRVLVGMAEAANYPCQMQLL
Micromonas                  GG--DAVLMYGITLWSLATIATPFAAT----SASLPLLLLTRAVMGLGEGVALPCMNNLV
Bathycoccus1                GG--ARVLLGV-------------------------------------------------
Amphiamblys                 GG--VNLAFSGMVVCCAGILCIPFAAS----R-GVWALFLCRAVLGVFEGLVFPVFHSMV
Bathycoccus2                GG--KAVLFYGVVLWSLGTLIAPWCAG----L-GMGPLLASRFLVGLGEGVAPSAATGIL
Guillardia                  GG--KSVLFGGVLLASLFTFLTPPASK------NFTMLVICRTLMGAVEGVSFPAILTII
Basidiobolus                GG--KSVLALGAFLWSLFTLATPWVAH------HVFLLFACRMLLGAGEGLGFPAIHSLI
Congregibacter              GG--KLLLGVALIGWSLFTFLTPIAAG----V-SLGALLLTRVLMGMGEAASFPGVYNLL
Sphingorhabdus              GG--KVVLGFGVFIWSLFTLVAPPAAA----L-GITVLIVARILMGMGEAVTFPAIYALY
Sphingomonadales            GG--KVVLGFGVLIWSLFTLVAPPAAA----L-GITVLIVARILMGMGEAVTFPAIYALY
Thelohanellus               GY--RRVMIFCLAASTILTIASLFAAP------HQWVFFAIRSMIGLVNGPLYPIIHETI
Chrysochro2                 GA--RITVLASLLCGSIISIALPPAAL-I----HPWLVMLLRLLHGLAQGVLFPGFAALW
SLC17A1_HSA                 ST--KKMIGFALCLSSVLSLLIPPAAG-I----GVAWVVVCRAVQGAAQGIVATAQFEIY
SLC17A3_HSA                 KA--PKS-------------------------------------LPTKSSILGGQFAIW
Strongylo3                  GRAAVWLLGFGYGVSAVCTLLTPLAAHLG----V-PFLMATQVVSGITQGWTFPVFIALM
Strongylo5                  PQSCIWLIGVGYLLSAIFTLFVPLAAYAG----GAPLIITLRILSGLSESGTYPGLYSLM
Strongylo1                  PKSCSWIYGLGVFLSSVGSLLIPLAAHHS----VT-AIFVTRFICGLAESGTYPSMYSFM
Ciona1                      GF--PIVFGLPIFISALLSIATPFAAY-T----SFPLVIACRVLMGLLQGASVCAFQGCW
Drosophila2                 GT--KLIFGWSNGIGVFCCFLIPIVSY-W----SYTGLIVLRVFQGWITGLAWPSMHVLT
Strongylo2                  GG--TRVFGISMFLSAAVSLLSPIAAK-L----GYSYFFAVRILCGIGEAGTFPALGSMV
Drosophila1                 GA--RNVAGYSCLVAGILTALTPAAAA-W----DKYAVFAVRFLIGFLNGVVYPCCHSLV
Drosophila4                 GG--RRVFGHSMLWASLLTLITPLAAH-I----NYVVLIVVRVVLGFMLGASWPAIHPVA
Drosophila3                 GG--KWTLSIGIFLTAAFTLITPVCIVYG----GADALIVLRVLMGLGEGTTFPALSVLL
Aplysia4                    GG--RRVWGVCMFVSAVCTLLTPVAVR-T----HIYIAYGLRFLLGFVSGVNFPCIQAIF
Aplysia1                    GG--KRVLGIGVLVTAIATVLLPVAAR-Q----DHRAVYALRVIMGLAEGVSFPCMHSMW
Capitella                   GP--KKVFGVAMVINVMATILTPIAAR-V----SVYLVLVLRVLLGLCQGVCFPASHHMW
Hydra1                      GG--KNLFGIAILFSAVLTLMTPMASR-S----HWMWLFLLRLIEGLCEGCIYPAMYALW
Caenorhabditis              GG--KRVVFVTILGSALLTLLNPVAAR-T----SEYALAILRAAIGFLQGATFPAMHTMW
SLC17A8_HSA                 AA--NRVFGAAIFLTSTLNMFIPSAAR-V----HYGCVMCVRILQGLVEGVTYPACHGMW
SLC17A6_HSA                 AA--NRVFGAAILLTSTLNMLIPSAAR-V----HYGCVIFVRILQGLVEGVTYPACHGIW
SLC17A7_HSA                 AA--NRVFGFAIVATSTLNMLIPSAAR-V----HYGCVIFVRILQGLVEGVTYPACHGIW
Tribolium                   GG--KRVFGGGIAVTAFLTLITPILAR-I----NLSLLLTLRVIEGIFEGVTYPCIHAVW
Aedes                       GG--NYVFGVGIGTTAILTLLTPLAAK-A----GLGWMIGVRVIEGIFEGVTFPCIHAVW
Amphimedon1                 GG--KLVLGLGIVITAALSLLTPLAAR-I----HFGALIALRVLEGFFEGVTFPAMHAMW
Amphimedon2                 GG--KTVLGLGIVITAALTLLTPLAAK-L----NLGVLIGLRVLEGLFEGVTFPTMHAMW
Amphimedon3                 GG--KTVLGLGVVITATLTLLTPLAAR-I----NFGALIGLRILEGLFEGVTFPTMHAMW
Oscar                       GA--KWVFGLGCLCTTVLTLVTPLAAY-K----SKWLLVAVRVLEGLGEGVTFPAMHAMW
Acropora                    GA--KNLFGFGVLTTSVLTMLTPLAAC-H----SVGTLIAVRILEGLGEGVTFPAMHAMW
Exaiptasia2                 GG--KHLFGLGILCTSVLTLLTPWVAH-R----GVGAFVALRLLEGLGEGMAFPSINALW
Nematostella                GG--KHLFGFGVLCTSIFTLLTPWAAH-Q----GIPMLIALRILEGLGEGVTFPAMHAIW
Exaiptasia1                 GG--KHLFGLGVLCTSVFTLMTPWAAH-Q----GVGTLVALRVLEGLGEGVTFPAMHAVW
Aplysia2                    GG--KWVYGITMLIAAVATVFTPLLAE-A----HYIALIIVRVILGAVTGMTFPAMHAIW
Trichuris                   GA--KWVFGLGTFLTAVFALLTPVAAN-A----GAGMVIFVRVLMGIAEGVTWPASQALF
Daphnia                     GG--RKLLGYGILSTSVFTLLTPFAAR-A----SDTLLIFCRVLMGLGEGVTFPAMYAML
Sialin_HSA                  GG--KMLLGFGILGTAVLTLFTPIAAD-L----GVGPLIVLRALEGLGEGVTFPAMHAMW
Aplysia3                    GG--KRLFGLGVLCTSVLTLLTPVAAR-T----SEYLFIALRILEGIGEGVTFPAMYAIW
Crassostrea                 GG--KKLFGFGVLCTAILTLLTPLAAR-W----NLYVFIALRVIEGIGEGVTFPAMNAMW
Biomphalaria                GG--KNLFGYGVLCTSVLTLITPVAAR-Y----SVYLLIAVRVLEGLGEGVTFPAMHAMW
Lottia                      GG--RRLFGYGILCTSLLTLLTPVVAR-W----SLPGLIVLRVVEGIGEGVTFPAMHAMW
Ciona2                      GG--KWLFGLGILCTSVLTLLTPVATR-T----SFVLLIILRIFEGIGEGVTFPAMHSMW
Strongylo6                  GA--KWLFGSGVLCTAIFTLLTPVAAR-T----GLPWLIVVRVIAGIGEGVTFPAMNAMW
Klebsiella_Nant             GR--RLAMVTSIVLFSLGTLACGFAPG-------YTSLFIARLVIGMGMAGEYGSSATYV
Escherichia_nant            GR--RLAMVTSIVLFSAGTLACGFAPG-------YITMFIARLVIGMGMAGEYGSSATYV
Acyrthosiphon_sv2           GR--IRVMQLTVLWVACFTALTALCGD-------FWQFLIVRFLQGLGYGGEVVVGGVLI
Fonsecaea_Nant              GR--KWPFIINNILFIALELGTGFTQT-------YGQFLAVRALFGIAMGGLYGNAAATA
Calocera_nant               GR--KWPLIANLILCTIFSLCTAFALN-------YGAFLGVRCLFGIAMGGIWGLSAATA
Hebeloma_nant               GR--KWPLVWNLVLDAILELGAGFVQT-------FPQFLALRSLFGIGMGGIWGLAASTA
Gymnopus_nant               GR--KYPLVLNLLVVSVLELGAGFVQT-------YSQFLAVRSLFGIGMGGIWGLAASTA
Mycobacterium_Nant          GR--RVPLMVDVSFYSVIGFLCAFAPN-------FTVLVILRLLYGIGMGGEWGLGAALS
Drosophila_nant             GR--KPVMIITILVYSVIEVLSAFSPT-------LWVFLVLRTLFGIALGGEWGVGTSLI
Amphimedon_sv2              GR--RTVLIMSLFFNSFFAFVSAMSPN-------YYFLLATRFLSGVGVGGSLPVFGPYL
Orbicella_nant              GR--RKPWLFAYIVGGVLALMSAFVGT-------YEEYIALRFAMGVMNGGGGLITYVLS
Strongylocentrotus_sv2      GR--KKTLALCSFHIFYFGFLSSFSPN-------LIWLLILRGLLGASLGGTS-QSVIIC
Homo_sv2                    GR--KTGLKISVLWTLYYGILSAFAPV-------YSWILVLRGLVGFGIGGVP-QSVTLY
```

```
Daphnia_sv2                GR--KKALALSAVLLSIFGLLSSFSPT-------FFWILLLRLLVGFAIGCTP-QSVTLY

Dictyostelium              NQWIPPSQRTSSAAIIWSGAYIGTIVADF-SVPKIIES------------HSWDVCFYLF
Emiliania                  ARFVQPERRSLFWGCLTASLSCGTIAAYV-LAPPLIAE------------QGWPFVFEAF
Cryptosporidium            GHHIPSKYSSTVISIIIASSFIGGGFAAF-VTPPMILS------------LGWRGPFYVF
Aureococcus                SRWLPLAKRSSGIADVFVFFNAGNAAGLL--LGGLIPV------------LGWRALMVGG
Enterobacter               YSWLPAGERALAQGINLSGSRLGAAFALP-FVAWLLTT------------AGWRESFLIL
Chromera                   YSWIPLSERGIVNGINFSGGRIGAAFALP-LVAWLMDL------------VGWRLTFVLL
Cellvibrionaceae           FSWIPIEERGLVTGINFSGSRLGAAFALP-TLAWLIAV------------IGWKVTFVVL
SLC17A9_HSA                SQKVRESERAFTYSIVGAGSQFGTLLTG-AVGSLLLEW------------YGWQSIFYFS
Hydra2                     AKSVSIEDRSFIFSCTFSGSSIGTVITG-LFGTILLEQ------------VHWSSVFYFF
Chrysochro1                SVWAPYKERSRFWSFAATGEALGTIVAL-GVGPALVHA------------YGWRSIFFAC
Micromonas                 ARWAPTRERSRAVSVCMGGFQSGSMVGL-LAAPHMLRV------------GGVHGPFVAF
Bathycoccus1               ------------------------------APLLMSK------------FGIAGPFYVF
Amphiamblys                CRWIPPCERSFTVSLFTASMQLGMIFSNILSPLSIESS------------LGWRGVFFVS
Bathycoccus2               AKTIPASQRSKAVTATFGGLDVGSLLGLLIAPPIILFL------------GGWQAVFYLF
Guillardia                 GHWAPAQERSTMVGFVYAGAYTGNVVTFP-LSAWIMDT------------YGWRTIFYFF
Basidiobolus               AQWIPHHESSRAVATVTAASYAGAILALLISAPIAASS------------LGWKWIFYIF
Congregibacter             GRWIPKQEKSRAAAVNLTGIPLGTIFAL-STTGLLVSA------------YGWQSVFFVF
Sphingorhabdus             ARWIPVKERSRSAGFSNSGIPLGTVFAL-LATPIIVAQ------------WGWEWVFYLF
Sphingomonadales           ARWIPVKERSRSAGFSNSGIPLGTVFAL-LATPVIVAS------------WGWEWVFYLF
Thelohanellus              AGHSPPSERTFLTMFTHTGNLVSLAIILP-IGGLFIDNFV----------NGWKYVFILS
Chrysochro2                SMWAPPLERSQLDGIPRAGGFFGAMLCNA-LAGTQCDLLV----G-VPLIGDWSGVFTAW
SLC17A1_HSA                VKWAPPLERGRLTSMSTSGFLLGPFIVLL-VTGVICES------------LGWPMVFYIF
SLC17A3_HSA                EKWGPPQERSRLCSIALSGMLLGCFTAIL-IGGFISET------------LGWPFVFYIF
Strongylo3                 SRWAPPSERSSLVAIGTSGIPLGQVIGQP-VAGLWASSEF----A-----GGWPSAFYFS
Strongylo5                 SRWAPPADRSKLLAIVFAGSSVGQIIAQP-ISGILSESDF----L-----GGWPSTFYLF
Strongylo1                 SRWAPSADRSILLAIVYAGLPIGHIVMQP-ISGLLAESSY-----------GWPSSFYMI
Ciona1                     SSWAPPLERSLLNSIALSGFPFGLGFANF-FAGYICGT------------LGWEAVFYIL
Drosophila2                AKWIPPNERSKFVS-AYLGSSVGVALFYP-IFGYIIDW------------TRWEWVYYIC
Strongylo2                 ARWFPPQDFSAAFSIALSSAKFGTFIGTV-LSGLISGSDL----L-----GGWPMTYYAF
Drosophila1                SKWAPPDEKGKFVASL-MGGTFGTVITWP-LSGVIIEN------------MGWDWAFYMV
Drosophila4                AVWIPPMERSKFMSNMMA-SSLGAAITMP-ICGYLISV------------AGWASVFYLT
Drosophila3                AAWVPATERGMLGALVLGGGQVGSIAGNL-LSGLILDS------------MDWPWVFYIF
Aplysia4                   GRWVPPLERSIMNAIASSGMLLGNIFTFS-ISGVLCKHGF----D-----NGWGSIFYLS
Aplysia1                   GRWSPTMERTRLASFTYAGLNVGNVVTYI-VSGLLCAYGF----D-----NGWAAIFYLT
Capitella                  GRWAPPLERSKLVSFSYAGTIFGTVVALS-LSGFLCSVEF----A-----GGWPLIFYIY
Hydra1                     SRWAPPMERAKLVTIPHSGSYAGSVAGTL-IAGYLCEL------------CGWAWVFYLF
Caenorhabditis             SVWGPPLELSVLTGVTYAGAQIGNVIVLP-LSGFLCEYGF----D-----GGWPSIFYII
SLC17A8_HSA                SKWAPPLERSRLATTSFCGSYAGAVVAMP-LAGVLVQY------------IGWSSVFYIY
SLC17A6_HSA                SKWAPPLERSRLATTSFCGSYAGAVIAMP-LAGILVQY------------TGWSSVFYVY
SLC17A7_HSA                SKWAPPLERSRLATTAFCGSYAGAVVAMP-LAGVLVQY------------SGWSSVFYVY
Tribolium                  SRWAPPLERTRLATLAYAGSHIGTVVSMP-VSAYLATA------------LGWPSIFYFF
Aedes                      SKWAPPSERSRMASIAFAGNYAGTVVAMP-LSGIFANA------------YGWESVFYIF
Amphimedon1                GKWAPPAERSILTTITYAGPFFGNVISFP-LSAVLCVYGF----A-----GGWPSVFYTF
Amphimedon2                GKWAPPAERSTLATITYAGPIVGNVVSFP-LSAVLCVYGF----D-----GGWPSVFYTF
Amphimedon3                GKWAPPAERSTLATITYAGPIVGMVVSFP-LSALLCVYGF----A-----GGWPSVFYTS
Oscar                      SKWAPPMERSKLATIGYTGSYVGTVIAMP-ISGLLCDHGF----TSHPHGSRWPSVFYVF
Acropora                   SSWAPPLERSKLITFSYAGLQLGTIIGMP-LTGYLCSSSF----W-----GGWPSVFYIF
Exaiptasia2                SSWAPPLERSKLVSITFSGSRIGTILALG-ISGFLCDSDV----T-----GGWPSVFYLF
Nematostella               SSWAPPLERSKLLTLSYAGCQLGNIIFMP-LAGVLCASEI----A-----GGWPSVFYIF
Exaiptasia1                SSWAPPLERSKLATFSYAGAQIGTIISNP-VSGALCDSNF----L-----GGWPSVFYIF
Aplysia2                   GNWAPPLERTKLMTFTYGAQVGIVIGFP-LSGFLCKYGF----A-----GGWPSIFYVT
Trichuris                  AKWAPPSERSKLPSFAISGCFIGTVVAFI-ASGFLADSNI----M-----GGWPSVFYCF
Daphnia                    AEWAPPWERSKMAAFAFTGAQFGTVITLP-LSGILCEHGF----D-----GGWPTVFYVF
Sialin_HSA                 SSWAPPLERSKLLSISYAGAQLGTVISLP-LSGIICYY------------MNWTYVFYFF
Aplysia3                   GQWAPVWERSRLVAFTTAGAQFGTVISLP-LSGLLCDSDI----L-----GGWPSVFYIF
Crassostrea                GKWAPLWERSKLLSFTYAGAQLGTVFSMP-ISGILCKSDF----L-----GGWPSVFYLF
Biomphalaria               GSWAPVWERSKLAAFTYAGAQLGTVISMP-VSGLLCDSDF----G-----GGWPSAFYVF
Lottia                     GSWAPLLERSKLVSISYSGAQLGTVISMP-ISGLLCDYGF----A-----GGWPSVFYVF
Ciona2                     GIWAPPSERSRLVSITYAGCHLGTVIAQP-ISGILCASTF----L-----GGWPSVFYVF
Strongylo6                 ANWAPPVERSRLLTFTYAGSHFGTVLALP-ISGVLCNSDF----L-----GGWPSVFYVF
Klebsiella_Nant            IESWPKHLRNKASGFLISGFSVGAVVAAQ-VYSLVVP------------VWGWRALFFIG
Escherichia_nant           IESWPKHLRNKASGFLISGFSVGAVVAAQ-VYSLVVP------------VWGWRALFFIG
Acyrthosiphon_sv2          SEVIRASYRGRVGASIQSGYAIGYAISLA-VLPMLLNF--------FPQQIAWKLFFLAG
Fonsecaea_Nant             LEDCPEEARGIISGMLQQGYAFGYLLATA-FARALVNT----------TSHGWRPLFWFG
Calocera_nant              LENLPVQVRGLASGVLQEGYAVGYLIAAV-INLTLVSY----------DPFGWRALFWVA
Hebeloma_nant              LENLPVEARGLASGFLQEGYAVGYLLAAV-INLFLVPK----------AKAGWRALFWTA
Gymnopus_nant              LENLPVETRGLASGFLQQGYAVGYLIAAV-INLFLVPE----------QSHSWRALFWTA
Mycobacterium_Nant         MEKVPAERRGVFSGLLQEGYAFGYLLASV-AALVVMNW----------LGLSWRWLFGLS
Drosophila_nant            MESVPASWRGTASGILQAGYPAGYLLASI-LFLT---L----------PVVGWRGLFILG
Amphimedon_sv2             SEFAGSKYRGSYLNFMSTSWITGAVAVTG-VAWALLPQEDIGLTVNGTSVHSWRVFLFLC
Orbicella_nant             TESIGPSYRGSAGTWQQAFYAVGYVVL----ALE------------AYFIRDWRTLTVIT
Strongylocentrotus_sv2     AEFLPSKSRGLCLVCLEAFWVIGVCLEIT-LAMV------------VMPTLGWRYLLIFS
Homo_sv2                   AEFLPMKARAKCILLIEVFWAIGTVFEVV-LAVF------------VMPSLGWRWLLILS
Daphnia_sv2                AEFLPVQQRGKCVVLLDSFWALGACFEVL-LALI------------IMPTLGWRWLLALS
```

```
Dictyostelium              GAVGLFWALLWLIFLKDDPANAWGIH------PNEVHAIKSQMET-LKVV
Emiliania                  GAAGLSIAVLWAVLGADAPRELESAG------ASS-----------GEVP
Cryptosporidium            GLIGVFWSIVWLFLDVKSLSWTNKPSIYEFYDTKEKNDLEIQLPDFIFNF
Aureococcus                GAAGLAWGAGGLALAKSIAAKHDPPE------D-------------GGL-
Enterobacter               CAVGLIWAVCWYAWFRNDPAEHKSIS------KAELAIIEA-----NSTP
Chromera                   GAIGILWGILWFLWFRDDPKDHKGMS------DPEVNYIRE-----VKLD
Cellvibrionaceae           GLIGVFWALLWFWFFRDEPQSHPFIS------THEKHHILE-----SSIN
SLC17A9_HSA                GGLTLLWVWYVYRYLLSEKDLILALG--------------------SRVP
Hydra2                     GIASFLWAFLLKCLHWSKKKNPLDTD--------------------KIVP
Chrysochro1                AASGGVWALLFGLLAASHPEKHRGIS------TTELAHI-------VFTP
Micromonas                 GALGLTWAVAWRFAASAYPRGSRREGRV---SDAELSLI-------GKVP
Bathycoccus1               GTIGVAWAAVWNVCATSYPRVNERVG------KVELKFI-------KKTP
Amphiamblys                GAVGLVWAGVWLFFMTNTPREHRSVS------EKEAVYIEREI---RGIP
Bathycoccus2               GVLGFVWGLWWWFGYANDKSVDM--K------E-------------LNIP
Guillardia                 ACLGFLWCLLFHLFTTSTPSQHRSMH------AAELN---------ATVP
Basidiobolus               AIMGFGWNIPWYIFGASTPDQCSELS------SLRSMYDGKKD---NRIP
Congregibacter             GASGLLFAVVWLRVVHAKPSVHPTIS------AEERA---------EPIP
Sphingorhabdus             GGVGFIWCVIWYYAIAPTPSRQSGIS------QSELD---------TATP
Sphingomonadales           GGVGFIWCAIWYFLIASSPRKQPGIS------QAELD---------VATP
Thelohanellus              GALGVLATIMWTLFVYSEPEENPWMS------ESEQKYITGQIYPLKNVP
Chrysochro2                GLIGLAFSIVWWRHVEDSPAMDRTCS------AAEAAFIAQEIRAHARRL
SLC17A1_HSA                GACGCAVCLLWFVLFYDDPKDHPCIS------ISEKEYITSSLVQQQSLP
SLC17A3_HSA                GGVGCVCCLLWFVVIYDDPVSYPWIS------TSEKEYIISSLKQQQPLP
Strongylo3                 GALGVLWFIVWMCLVYPSPSSHPRIS------SKEREYIEKSLLHEAVYP
Strongylo5                 GSLEVLWCILWFLVIYPSPMAHPRIS------QEEKDYIMAELKLEKDYP
Strongylo1                 GVIGIIWFVFWTIFIYDSPNSHPSIN------SEEKEYLIKELQLWKSYP
Ciona1                     GGVAIGWSVVWIIIVSDSPRTNRCIT------AAEVAYIEKSIGFTQVVP
Drosophila2                GIVGTLWFIAWQFLVFDTPAQHPRIA------DSERKYIEKSLGASGPTP
Strongylo2                 GGFSLVLVVVWMARIYETPGRHPRIS------SKEKQYLSTTVITEISIP
Drosophila1                GIFVLIVVSIWFFLVTDTPAQHSTIS------LKEREYIENSLGSTKWPP
Drosophila4                GAVGLLWSLAWFTFVYETPATHPRIS------AEERREIEEAIGTTSHVP
Drosophila3                GIIALVWFTVFSLICYSYPYKHPFIK------PSEREFLKQEIPPKPKTP
Aplysia4                   GVMFLLWVVAWFPVTSDTPAQHKRVS------AGERKYIEGSIGRSSDVP
Aplysia1                   GGAAFIWVFAWFVFVYDSPEDHPSIS------QAERDYIVSHRGDKFKVP
Capitella                  GGACAVWCVAWFYLMHDTPMKHPRIT------QKERNLITSEVGAATHVP
Hydra1                     GLLALMWVAVWMIMISDSPEEDNHIS------TEEKNMILKSLEEDGSLP
Caenorhabditis             GVFGVLWTAVWWYVSSDKPATHPRIT------PEEKQYIVTAVEASPSTP
SLC17A8_HSA                GMFGIIWYMFWLLQAYECPAAHPTIS------NEEKTYIETSIGEG----
SLC17A6_HSA                GSFGMVWYMFWLLVSYESPAKHPTIT------DEERRYIEESIGESFKTP
SLC17A7_HSA                GSFGIFWYLFWLLVSYESPALHPSIS------EEERKYIEDAIGESFSTP
Tribolium                  GSLGLIWFVIWWVVVAESPAEDSRIS------KEELEYIEQSLGNVIVYP
Aedes                      GVIGCVWFVLWTFMIKTSPEVDRGIS------DKEKEFILSSLGRTIRHP
Amphimedon1                GILGIIWYIFWLLLIFDTPAKHPRIS------EREKEYIEGGIADVVPVP
Amphimedon2                GILGIIWYIFWLILIFDSPANHPRIS------KAERDYIESGIPDAVPIP
Amphimedon3                GILGIIWYIFWLILIFDSPANHPRIS------KAERDYIESGIPDAVPIP
Oscar                      GVVGLLWCVAWLCLVHSSPQSHPRIS------PEEREYIEMAIKEGAAIP
Acropora                   GAIGVIWFFIWMTFTYDRPSDHPRIS------IKEREYIESTIGEGCLAH
Exaiptasia2                GAQGILWFIVWTIFIHEKPAEHPRIS------IEEKQYILKSLGSTHSAP
Nematostella               GSLGILWFVVWTLMVTEKPADHPTIS------EAERDYIISSIGTSQNTP
Exaiptasia1                GSLGILWFIVWTVLVYDKPADHPRIS------KEEKDYIQ-TIGSQHSTP
Aplysia2                   GAFSLIWAVAWILLVSDSPSTHKRIS------DAEREYILSSLQSGIRVP
Trichuris                  GIAAILWFIIWTIFVSNGPEGHRFIG------NAEKTYIVNSLKVEKDIP
Daphnia                    GTLGIVWFAVWMPLTADSPSRHKNIS------KEEKEYICSSLRDSAPVP
Sialin_HSA                 GTIGIFWFLLWIWLVSDTPQKHKRIS------HYEKEYILSSLRNQKSVP
Aplysia3                   GAVACLWCVFWMLIVHDTPAKHPRIS------VAEREYILTSLGDTISPP
Crassostrea                GVIGCVWFAVWMLVVHDTPAQHPRIS------QEEKDYIETSVGTRLKTP
Biomphalaria               GSLGCVWFVAWMFLVHNTPAEHPRIS------TSEREYIESSVGKRIKTP
Lottia                     GALGCIWVFFWMFIVYDHPSKHPRIS------KSERLYIESTTGKRTTTP
Ciona2                     GTLGILWCIVWFIFAHSKPADHPRIT------TSELNYIQSNLEPKVSVP
Strongylo6                 GVCGVVWFILWIALVHDKPEKHPRIS------PEELQFLQKAIKPRLKVP
Klebsiella_Nant            IVPIVFALWLRKN-IPEAEDWKEKHE-----GKAPVRTMVDILYRG--SF
Escherichia_nant           ILPIIFALWLRKN-IPEAEDWKEKHA-----GKAPVRTMVDILYRG--SF
Acyrthosiphon_sv2          IVPAILVWFIRRL-VPESPVFLRKKV-----EENKSSVS-----------
Fonsecaea_Nant             ACPPVLIILFRLC-LPETNTFRARQA-----L------------RA--SE
Calocera_nant              AGISAFAAVVRLC-LPESQVFLRAKE-----DKRIKAAE-----GH--RE
Hebeloma_nant              SGISLFAAGIRVL-LPESEVFLRAKA-----AEVEK--------GH--KE
Gymnopus_nant              AGISAFAAFIRLC-LPESKIFLKARE-----DAKLREQE-RLGYRA--RE
Mycobacterium_Nant         IIPALISLIIRYR-VKESEVWEAAQD-----RMRLTKTR---------RD
Drosophila_nant            GSAVFSALYIWLR-VPESPDWLARQK-----ASASPTQK---------QG
Amphimedon_sv2             AVPAFVGGVLCIL-MPEGPRYLIEVR-----KEKRAIRILRNMYYLILKR
Orbicella_nant             ILPSFIILLVFKS-IPESPRWLASQG-----RIKEAENILRNIEKE-QYG
Strongylocentrotus_sv2     SFPLVIFVVLVTF-LPESASYQQASG-----NWSGAMATLEDISRTPKGS
Homo_sv2                   AVPLLLFAVLCFW-LPESARYDVLSG-----NQEKAIATLKRIATE-RGK
Daphnia_sv2                TIPVFVFTLVCAW-LPESARFLAANG-----RTEEALAVLRRIAEEWKSP

Dictyostelium              LKKLFSNSGI----YAVLIYFLTTSFGFYMLLMWYPTWLSKVSGLT-STLGFF
Emiliania                  WRALASSRPV----WAMTAAHCSSNFFMYFGLSWLPTYFSYQFGLS-ADASTA
```

```
Cryptosporidium            VKVLLLNKSI----FAIIVAQYCHGWTQFGFVTWMPIYFTDVCKVN-AYLGYY
Aureococcus                --SLRQVGQL----AALTHVHNCLNWSFFFMQAWLPTYFSQELGLE-SKSAGL
Enterobacter               PDMSFRSGNM----WLLMGQYFASNFTLFFALTWLFPYLKKTYELD-VTTGFL
Chromera                   FGKLFRSKTM----WQLMGQYFASNFTFFFCLTWLFPHIKEKYGLD-LEAGFY
Cellvibrionaceae           VSTLFGSGNM----WLVMMQYFCSNFIFYFSLTWLYPHLKAKYALD-VSAGLY
SLC17A9_HSA                WRRLFRKPAV----WAAVVSQLSAACSFFILLSWLPTFFEETFPD--AKGWIF
Hydra2                     IKVLLFNKSV----LAMLFAYFSMSVSFYVFLSWSPTYFHETYPK--GKAWLF
Chrysochro1                FWRILTNRPF----LATIVTHCAYNWGYYVGLSWVSKFFNSKYDAD-AQLGLL
Micromonas                 FRLLLSKMPT----WACVVANFVNNWGYFILLAWMPLYFREVVGLD-ASASYF
Bathycoccus1               WKMLLSHPAT----WACVVANFVNNFGFFILLAWMPKYFNDVVKLN-ATSSWF
Amphiamblys                WRGVFSSGVF----WAVLATQFFNSWCMFVFQQWLPTYYRAVFQTD-FATGLS
Bathycoccus2               WVKFAKSKEF----WALMVAHFTWNYFSYGLLAWLPSFLSSALNVS-AKSSFL
Guillardia                 WKKILVCMPA----WALFVVHTCFNWAFYTLLTQLPSYMALVLGFN-QQSGFL
Basidiobolus               WCKILRAKEV----WAILINQFCNSWGFYVLLSWLPTYYKDVFNVD-NELGYF
Congregibacter             WGLFLRHPSI----WALFINHFCANWTLYLFLAWLPSYFRDVQGLS-AGSGLF
Sphingorhabdus             WGALLGNMPV----WAIIVAHFCNNWWFYVLLAWLPTFVTQGLGVD-ASVGLF
Sphingomonadales           WRALMTNMPV----WAVIVAHFCNNWWFYVLLAWLPTFVTKGLGVD-AAVGYF
Thelohanellus              FCAIFTSPHL----YIFTIAHFAKMFVLYMNLFGIVKYLYKFFTLS-VMAGNL
Chrysochro2                YGHIFRSSAC----WAICLAHTAGDIGLYVLDDGLPPYLRDVKGVE-SQIGLM
SLC17A1_HSA                IKAILKSLPV----WAISTGSFTFFWSHNIMTLYTPMFINSMLHVN-KENGFL
SLC17A3_HSA                IKAMLRSLPI----WSICLGCFSHQWLVSTMVVYIPTYISSVYHVN-RDNGLL
Strongylo3                 WKSMLTSVPV----LAVCIADFSLLWILYLFTTNLPIYLKTVLQFD-RQTGFL
Strongylo5                 WKHFFTSLPL----LAVVVADFALMWVLYSLTSNLPIFLKEALRFD-SQAGIL
Strongylo1                 WFRIITSLPV----MAVGMADFAILWILHSLTTNLPIFLSEALRFD-ITAGAL
Ciona1                     WGAIMRSRSV----WAIFVAHFCDNWSGYTFNAILPTFMSKIFNFN-FQSGAV
Drosophila2                WKAIATSRPV----WLNVVAQWGGIWGLFTLMTHAPTYFSKIHHWN-RATGFL
Strongylo2                 VVDMLTSIPV----WTIIVTSFCGSWTNQAMFTNLPIYLKHVQGMD-ELIGIA
Drosophila1                YKELILSLPF----WSLMLLHYGSMWGLFFLITATPKFLSEVLGFN-SSAGFL
Drosophila4                WGQLLCSPAV----WAIIICHGLAVFGFFTVVNQLPTFMSKILHFD-KQNGLF
Drosophila3                WLAIFANIPM----WALISAQIGHDWGFYIMVSYLPKYMSDVLRFS-KANGLY
Aplysia4                   WVSIIRSGPL----WAIIVSHFCGNWLTYTLLTSLPTFMKESLRFD-SQNGAL
Aplysia1                   WKSVVKSRAM----WMCLGAHLCNNWMHYTLLTGLPTFMKQVLKYD-KQNGVL
Capitella                  WKHIFKSPAT----WAIVTAHVCNNWGSYTVLTSIPMYMKEVLYFD-KSNGLL
Hydra1                     WQKIITSMPF----LAILVAHTCEGWGFGTMQTGLPKFLSEAMNFR-SKTGEY
Caenorhabditis             WIKILTSPAV----WACWAGHFAGDWGAYTMLVSLPSFLKDVLGLN-SSLGAV
SLC17A8_HSA                -----------------------------------------------LSVGLL
SLC17A6_HSA                WRKFFTSMPV----YAIIVANFCRSWTFYLLLISQPAYFEEVFGFE-SKVGML
SLC17A7_HSA                WRRFFTSMPV----YAIIVANFCRSWTFYLLLISQPAYFEEVFGFE-SKVGLV
Tribolium                  WKSIFTSAPV----WAIVVAHFTDNWGFYTLLTQLPKFMKEVLNFP-NTSGIL
Aedes                      WKAMLTSMAV----WALVASHFSENWGFYTLLTQLPTFLKDTMHFQ-EKTGFI
Amphimedon1                WLQILTSPAV----WAIIVAHVSVNWGNYIMLTCIPSYFHDALGLSFIENGIF
Amphimedon2                WFQIFTSRAV----WAIIVCFISYGWGGYIMLTCIPSYLHDTLGLSLIENGIF
Amphimedon3                WFQIFTSRAV----WAIIVCFVCYGWGGYIMLTCIPSYLHDTLGLSFIENGIF
Oscar                      FGPMMKSSAM----WAIIISYFCNNWGFYTLLTDIPSFFKDVLGFD-TQDGFL
Acropora                   -----LQPAV----HNYIIISFIVCYCY----DCKKSLFASFISLY-FQDGFL
Exaiptasia2                WLSILTSRAV----WAIFIVNFSTSWGFYIFLTTLPMYFQQVLNIX-AKNGVV
Nematostella               WSAIWSSPAV----WAIIIAHFCNNWGFYTFLTLLPSYFKEVLNFS-LTNGFL
Exaiptasia1                WFAIWTSPVV----WAIIIGHFCNNWGFYTFLTSLPLYFKEVLNFK-LQNGIL
Aplysia2                   WLKIMTSLPV----YAIIIANVASDWGAYTLLTNIPTYISEVLKFD-AANGLV
Trichuris                  LKSILTSIPI----WAAMISHFTYNWALYVLLTGLPLYFKNVLGFD-KSNGVL
Daphnia                    WRCMFRSKAC----WAVGAANVGHAWGFYTLLTELPSYMDNILHFN-KQNSFV
Sialin_HSA                 WVPILKSLPL----WAIVVAHFSYNWTFYTLLTLLPTYMKEILRFN-QENGFL
Aplysia3                   WKQIFLSPAL----WGIMGAHFASNWGFYTLLTSLPTYMADILKFD-KQNGLL
Crassostrea                WLSIWTSPAV----FAICAAHFANNWGFYTMLTCLPTYMKKILHFD-QQDGFL
Biomphalaria               WLQILTCPAL----WGISLAHFTNNWCYYTLLTCLPAYMSNILRFN-KENGLL
Lottia                     WKSIFTSGPL----WGIAAGHFANNWGMYSMLTCLPTYMKEILKFD-KANGIL
Ciona2                     WWEIATSKRV----WAISIAHFCNNWGFYTLLTCLPTYLKDVLKFD-QQDGFI
Strongylo6                 WFKMLTSVRL----LAISLSHFSNNFGFYTLLTNLPSYLKFGLGFD-SQSGFL
Klebsiella_Nant            MVQSSGK--RWPTGVMLMVVVLFAFLYSWPIQALLPTYLKTEL----------
Escherichia_nant           MVQSAGK--RWPTGVMLMVVVLFAFLYSWPIQALLPTYLKTDL----------
Acyrthosiphon_sv2          -----GK--NLRITITATLLASGIFGGAYVMITWLPTYLRMEL----------
Fonsecaea_Nant             GRVALKK--HWLLLIYMVLLMAGFNFMSHGSQDLYPTMLTNQF----------
Calocera_nant              TRAMLRQ--HWLRCIYAVLLMTGFNFLSHGSQDLYPTYLQQTK----------
Hebeloma_nant              TKDMLKK--HWLLCIYAVLLMTGFNFLSHGSQDLYPTYLTVSK----------
Gymnopus_nant              TGKMLRH--HWKLCIYACLLMTGFNFFSHGSQDLFPTYIQTTK----------
Mycobacterium_Nant         VLGN-PA--IVRRFVYLVLLMTAFNWMSHGTQDVYPTFLTATTDHGA------
Drosophila_nant            LWPIVRQ--NAALCVFAITLMTGFNFMSHGSQDLYPKVFLALERHI-------
Amphimedon_sv2             TLKLFHKN-LVRSTILLMFINFGLSFGSYGVTLWFPSYVDQLTNNGLKGSSFL
Orbicella_nant             VIDLFTHKSVCIVTVIMMFTWCVNSMVYYGLALNVKN----------------
Strongylocentrotus_sv2     IRELFSTKLLAMTTVILINLWFCNAFLYYGNVLLSAELFSS----GV------
Homo_sv2                   MRDLFTPH-FRWTTLLLWFIWFSNAFSYYGLVLLTTELFQA----GD------
Daphnia_sv2                LQRLLSPE-LKLTSLLLWFIWLACAFCYYGMVLMSTELLAG----AG------

Dictyostelium              ------SVLPYIGSFALSNISGLITDYLL-SRG
Emiliania                  ------ALYPFAAGAVGSLAAGAACDALVSSLH
Cryptosporidium            ------TTPPWVLQAFFVIFFGFLADKLV-SSS
Aureococcus                ------SALPMVTMALGSKVGGQAATRLI-AEG
```

```
Enterobacter               ------AAAPLVAGAIGNWTGGAVVDFLYRRG-
Chromera                   ------ASAPLIFGALGNWFSGFLVDFIYKKN-
Cellvibrionaceae           ------ASLPLIMGACGNWLSGWWVDRIYAKN-
SLC17A9_HSA                ------NVVPWLVAIPASLFSGFLSDHLI-NQG
Hydra2                     ------NVLPYLASFLLSLGSGKVANMLI-VCG
Chrysochro1                ------SIAPYIALLIATCLSGIVADGLENGCG
Micromonas                 ------SALPWATMAVVGVCAGALADWMIAR-G
Bathycoccus1               ------SALPWATMAVSGVFAGILADRMLSEWK
Amphiamblys                ------VVLPYSVQCCVALCVGYFGERVV-QQA
Bathycoccus2               ------SILPYLSTVAVTTLVAPIADSLENKGI
Guillardia                 ------SSMPYLFMFIVSILGGMLADWTISRGL
Basidiobolus               ------SVLPYIVQGLVGLASGFIGDYAIYRLG
Congregibacter             ------AIGPWVCQFLAGNLSAVVADRWI-AKG
Sphingorhabdus             ------AMMPHIALFLCMNISGLVADRLI-GRG
Sphingomonadales           ------AMMPHIALFLCINLSGVVADRLI-GGG
Thelohanellus              ------AVIPFLADFIFQTFYPKFVNSMKKSG-
Chrysochro2                ------LALPGLLKPCIILLSASLADRLRRR--
SLC17A1_HSA                ------SSLPYLFAWICGNLAGQLSDFFLTRNI
SLC17A3_HSA                ------SALPFIVAWVIGMVGGYLADFLLTKKF
Strongylo3                 ------AAVPFVFYWLVWTVGGRFTDFLIARTK
Strongylo5                 ------SAVPHIVFFIFILGGGVLADFLLSHTN
Strongylo1                 ------SAFPYAILLMTLLGGGFLADYLISRTK
Ciona1                     ------MTAPYIAQVVVSILAGFMTDAARQRKW
Drosophila2                ------SGLPHLMRMIFAYVFSTFADYLLRTDR
Strongylo2                 ------AAIPFAVESFFLVVGGMISNKLIKRQV
Drosophila1                ------SSLPHVARLLCAFGFGAVADWIRRRGW
Drosophila4                ------SSLPYLGKYVMAVASSYLADYLRKKGT
Drosophila3                ------SSLPYVTMWIMSLLSGCVADQMIKRNF
Aplysia4                   ------SSAPYLTQLVTSLMSGVAGDKLRQRRV
Aplysia1                   ------SAVPYMAMIFTQIFAGHAADFLRRR-F
Capitella                  ------SAVPYMVMYVVSIFAGWFADFLRQRRI
Hydra1                     ------TATLYLVMGINVFLSGQLADFIRQRCY
Caenorhabditis             ------ASIPYIAYFLAINAGGVLADTLRSKGI
SLC17A8_HSA                ------SAVPHMVMTIVVPIGGQLADYLRSRQI
SLC17A6_HSA                ------SAVPHLVMTIIVPIGGQIADFLRSKQI
SLC17A7_HSA                ------SALPHLVMTIIVPIGGQIADFLRSRRI
Tribolium                  ------SAIPYLAMAITIQLSGHLADRLLEKKI
Aedes                      ------SAIPYLVMGILLFVSGYLADLCQVKGW
Amphimedon1                ------SGIPYVGFVVSTIIAGQVADFLRKKWI
Amphimedon2                ------SAIPYXGYAIVTIVGGRVADLLRK-CI
Amphimedon3                ------SSIPYIGYAVVTIVSGQVADLLRKRGI
Oscar                      ------SAAPYAVMAAVVPLTGWIADTLREKNI
Acropora                   ------SAIPFLCMYVFGISAGQVADWLRLNKI
Exaiptasia2                ------SAIPNALAYVFIMVSGVMADWLQKSXI
Nematostella               ------SAVPYSTQWLFIVIAGQIADWLLRRRI
Exaiptasia1                ------SAIPYAAQFTVIVAGGQIADWLRDHRI
Aplysia2                   ------SALPYIAFWIVINLGGWVADFVRDRKL
Trichuris                  ------SSIPYLVQALIQSAAGQVADTLRTKCK
Daphnia                    ------SALPYLAMWLFSLLCSTIADLLISKQI
Sialin_HSA                 ------SSLPYLGSWLCMILSGQAADNLRAKWN
Aplysia3                   ------SALPYAGSWIIGPVAGLVADIIRRRHI
Crassostrea                ------SALPYLVCWMCQTISGQLADYIRKNQY
Biomphalaria               ------SAMPYAVCFLTQNTSGVLADFLRGRGY
Lottia                     ------SALPYLALWLLQLISGFIADWLRGRGI
Ciona2                     ------SALPYLVMWISINFNGLLADYLREKEI
Strongylo6                 ------SAVPYLVMWITINAGGQVADFLRGRYI
Klebsiella_Nant            HTVANVLFFSGFGAAVGCCVGGFLGDWLG----
Escherichia_nant           HTVANVLFFSGFGAAVGCCVGGFLGDWLG----
Acyrthosiphon_sv2          LSMSGYLAINILGSLIGPFLYGRLSDRFG----
Fonsecaea_Nant             DAVTVTQVVANLGAMTGGTTIGYCSQIFG----
Calocera_nant              HNANIATIIGNCGAVAGGIFAGILSQFIG----
Hebeloma_nant              HNATVATIIGNCGAIAGGIFAGWLSQYIG----
Gymnopus_nant              HDATVMTIIGNCGAIAGGTIAGFVSQYIG----
Mycobacterium_Nant         LTARWIVVIYNIGAIIGGLAFGTLSQRFS----
Drosophila_nant            PSITLIVVLYNVAAIAGGLFFGVLSQRIG----
Amphimedon_sv2             DAEYSHDLIITASGYVGNLVSAIAVFFFI----
Orbicella_nant             GSVYINFALASVIELPSYLVTQVLLARLG----
Strongylocentrotus_sv2     TQGYVGLLVSSLGEIPGILLTLFMIDTAG----
Homo_sv2                   EEDYMDLLWTTLSEFPGVLVTLWIIDRLG----
Daphnia_sv2                SADYTDLLWTTLAEFPGIVVTLVVIEFLG----

Dictyostelium              VRKIVLRKVFGILGGVLPGALLLIIS--F---SDISS----------SNQIAAMVLTIAT
Emiliania                  FRRTDARKVMQSVALGGPALAMLALC--L---L-SAG-AGGLQLER-DEAEALFVVAVGC
Cryptosporidium            IKPIIVRKLFQSVSMFVGAGCQLALV--L---LNNMG------LTSASYAIMVISLMFIF
Aureococcus                WAPFDVRRLMIAISSLIPAAALLALG-----GVADAG-----------VAVACLVLALGA
Enterobacter               -LGMYSRRLPAMFGFALSAAGMFGCM-----SADSAV-----------SAVAWLCLAVFG
Chromera                   -HWALSRKVPAIIGFLLASIGIVASV-----YMDEVT-----------PAIILISIAVFG
```

```
Cellvibrionaceae          -QWQLSRRLPAMVGFACAAIGLVCSL-----YMENVI-----------GAVLFLSLAIFG
SLC17A9_HSA               YRAITVRKLMQGMGLGLSSVFALCLG-----HTSSFC-----------ESVVFASASIGL
Hydra2                    FSVTFVRKLFGSLLFLGMCFFSLLLA-----ATHSFE-----------TTLFVMTLNIGM
Chrysochro1               ASATCARKVVNSIGMVGGALGFALLA-------IVCGPPGRLPRSQAYVGAGCLSVAIGL
Micromonas                VSTTTTRKLIQGVGFLGPAAALVALT-----FTSTPA-----------QALTALTVAIGC
Bathycoccus1              VSTATTRKFIQSVGFLGPALCLLILG--LKGFAITPG-----------FALTTLTVAVGC
Amphiamblys               RRRTLLRRVAQVCAMCIPASLLLVVV-----LCPASY----------TVSLCVFTVCIAA
Bathycoccus2              LSRTDVRKMSQTLCFGGGAVALSTVG-----FIVSRTPPASVTNTTIVMIMSALAFCFGM
Guillardia                LSRTRVRKTWMVTSLMVPACCLVLCG-----YASSWP----------V-AIFFMTCALGF
Basidiobolus              IPVVTVRRVAQCVGMLGPGVFLLLAG-----YTATSV----------TIGMVYISLALGF
Congregibacter            ISVTFVRKFMQCGGLIGGAAMLLLAA-----QASTPG----------M-ALFTLCAAFGV
Sphingorhabdus            MTITHVRKLMMIIGFGGSIIALLLVG-----QVESAV----------G-AIAIMTVGSAI
Sphingomonadales          MTITRVRKLMMLIGFGGSISALLLVG-----QAQDAV----------T-AITIMTIGSAL
Thelohanellus             MKITVIRKLNTFIGSFGCSSFLIASS--FISCTNLV------------GGIILQSLSLMF
Chrysochro2               FRTLHVRKAITIGAFIPQIGFLFALATGLLVEPKTIG----------VAMVIILPLGM--
SLC17A1_HSA               LSVIAVRKLFTAAGFLLPAIFGVCLP--YLSSTFY-------------SIVIFLILAGAT
SLC17A3_HSA               -RLITVRKIATILGSLPSSALIVSLP--YLNSGYI-------------TATALLTLSCGL
Strongylo3                FKITTIRKLLTFLAFMPSSVLLVAMG--YVGCNTS-------------VAMVTLTLGLSI
Strongylo5                FSITTVRKFMTTIGILPSGIFLVLAG--YVGCNAP-------------LAITFISLGLAC
Strongylo1                LSRTVIRKLMTSLGLIFSALFMVSSG--YTGCNAA-------------ATITMLSLGLAS
Ciona1                    ISTSAARKVNTIIAQSFLNLFMIIAC--YCT--DST------------TVIVLFVVGMSL
Drosophila2               LSRTNVRKLATFICCGVKGLLILALA--YFGYNAT-------------AAIMLVTVATMF
Strongylo2                LGVITTRKLVTFIGFALSALCFLLLA--YVGCDTV-------------LAVVFMIGAVGF
Drosophila1               LSVTRMRKAFCLPSHILPGVMLIILA--YFGRD-PY------------VCVAIMTISLGF
Drosophila4               LSTTATRKLFTTFALVIPGLLMIVQV--FLGYDAT-------------WSVTIFSLALFA
Drosophila3               MSTTNTRKIMTGLAAFGPAIFMVGAS--YAGCNRL-------------LVVALFTICMGL
Aplysia4                  MSTTAVRKTFQLAAFWGSAVCIVLAG--QMTCDQRY------------WAVVLLCLCVGF
Aplysia1                  LSTTHTRRLFQSVSFLGSGACIVAVG--FIDCERRT------------IAVVLLTLAVAL
Capitella                 LRTVTVRKMWQCISFWPPGVCIAAMG--FMSCEHRY------------VSVMLLSATIAL
Hydra1                    LSVTTTRKSFTVIGFLANAVAFIISM--YMF--SPE------------GAVIVIIVGAGI
Caenorhabditis            LSTLNTRRAAMLVALIGQGIFLVASG--YCGCGQDV------------LVIIFITCGMAI
SLC17A8_HSA               LTTTAVRKIMNCGGFGMEATLLLVVG--FSH--TKG------------VAISFLVLAVGF
SLC17A6_HSA               LSTTTVRKIMNCGGFGMEATLLLVVG--YSH--TRG------------VAISFLVLAVGF
SLC17A7_HSA               MSTTNVRKLMNCGGFGMEATLLLVVG--YSH--SKG------------VAISFLVLAVGF
Tribolium                 FTTTQVRKIFNCGAFIIHAGFMIGAA--FC--ETAV------------STITCLVLAVGL
Aedes                     LTTTQVRRFFNCGAFLGQTVFMITGA--FI--LKPG------------ATITCFTIAVGM
Amphimedon1               -STKNVRKGITLIAFVFGAAGLLVTG--YFGK-KKL------------DSVIFLTISVTG
Amphimedon2               -STKNVRKAMTISALVLGAAGLLVCG--YFSK-SKF------------DSVLYLSLTVGV
Amphimedon3               -STKNVRKGMTVSALVLEAAGLLVCG--YFSK-SKF------------DSVLYLSLTVGA
Oscar                     LSTTKTRKLMNTPGQLLPALFLVITG--YTT--NKT------------IAVACLCMAVGF
Acropora                  LPTGEVRKVFATGGFLVPACLMVSTS--YVGCEDTT------------LAVLLFSLALGT
Exaiptasia2               STTAEVRKIFTLAGLLPPAFLLVANS--YAGCNDKT------------LSIIIFSIAVGL
Nematostella              SSVSDVRKVFCVTAFIIPACLLVATS--YTGCTTTT------------LSVCLFSTALGF
Exaiptasia1               VTTTEVRKILTIGGLIAPACLLVATS--YTGCNDRG------------LSVGLFSTALGL
Aplysia2                  LTTGVTRKAFNAFGKILPAIMLISLG--YVDCSQPT------------VAIALLVLAVSL
Trichuris                 LSTTCVRKLMDCFGHLIPAICVIFVG--YVGCNSA-------------AAVALLTLSVGF
Daphnia                   FRVITVRKIFNSIGQYGPALALVGAG--FIGCNTV-------------AAVILLTLAVGL
Sialin_HSA                FSTLCVRRIFSLIGMIGPAVFLVAAG--FIGCDYS-------------LAVAFLTISTTL
Aplysia3                  LSTVCTRKVFNTLGCLIPATTLILVG--YVGCNHV-------------LVVVCLTLSVSL
Crassostrea               LTTANTRKVVNSCGLLLPAILLCCVQ--FAGCNHA-------------AVVAIVTFAVGL
Biomphalaria              LSTGNARKLFTSIGLLSPAIFLIVVN--YVGCDHV-------------LAVLFLTLSVGL
Lottia                    LNTNKTRKLMNTLGLLIPAVFMIGVS--YSGCDHT-------------LAVVLLIFAVGT
Ciona2                    LNTTQTRKIFNAVAFIGPGIFLVASG--FVGCNKV-------------AAVSLICLATAF
Strongylo6                LSTTNTRKLFNTLGLVLPAAFLVITG--YIGCNHV-------------LAVAMLTLAVGT
Klebsiella_Nant           ------TRKAYVCSLLASQLLIVPVF--AIGGTNVW------------VLGLLLFFQQML
Escherichia_nant          ------TRKAYVCSLLASQLLIIPVF--AIGGANVW------------VLGLLLFFQQML
Acyrthosiphon_sv2         ------RWKTIILFL-MGQIAVVSVY--MFADISLNI-----------TLVLGFFLGSLQ
Fonsecaea_Nant            ------RRFSIIFIS-IVGGALLYPY--TFVTS-KA-------------VIAAAFFEQFC
Calocera_nant             ------RRLTIVIFT-LSIGAFIPLW--ILPSGFSP-------------LAAGAFCVQFG
Hebeloma_nant             ------RRLTIIIHV-ALIGAFIPLW--ILPSTFSG-------------LAAGAFCIQFG
Gymnopus_nant             ------RRLTIITFT-LIAAAFIPLW--ILPNNFAG-------------LSAGAFCIQFG
Mycobacterium_Nant        ------RRYTIVFCA-ALGLPIVPLF--AYSRTAAM-------------LCLGSFLMQVF
Drosophila_nant           ------RQYAIALAA-LLTIPLLPLW--TLPQSAFW-------------LAVGAMCIQFC
Amphimedon_sv2            ------RSYWLAFSLFISTISVFMLY--FIRTETGAV--------------VLLCVFKAI
Orbicella_nant            ------RRQSLFFFLLSASVSCFLCM--FLQGGQNAA-----------AVSITALVGRFC
Strongylocentrotus_sv2    ------RKLTMGLEMLVCAVFSFLLL--M--CVDGIP-----------Q-MIFIFVIRGM
Homo_sv2                  ------RKKTMALCFVIFSFCSLLLF--I--C-VGRN-----------VLTLLLFIARAF
Daphnia_sv2               ------RKKTMALEFFIFSLTVFLIM--IV-CISNRS-----------MLTFMLFLARGI

Dictyostelium             AGFSA-AGVNVNTLDLAPN-YAGIVMGIANTVATIPGI-VGPLIA-------
Emiliania                 QAGSA-AGYGCGAQDISTR-LSSLIYGGTSVFAVIAGA-SGQYFTGWLLEQN
Cryptosporidium           NTMSG-GGVTVYQFDIAPE-FPAVVYAIGNTFGTIAGL-FSVSLTGLILNRS
Aureococcus               HSFSS-AGYHAHIADVAPS-SSGKILGFTNTVGVFVGI-VANVVTGRVLEAT
Enterobacter              SDITV-PASWAYCIDTAGK-KSGVVSGKMNMAGNLGSF-VTSLAFPYLMLAF
Chromera                  ADMTL-SPSWSVCVDVGKQ-FSGAVSGTMNMAGNIGSF-LTALAFPYLLELT
Cellvibrionaceae          ADMTL-SPSWSFCVDIGKN-NAGTVSGTMNMAGNVGAF-VTSLAFPYLKDWF
SLC17A9_HSA               QTFNH-SGISVNIQDLAPS-CAGFLFGVANTAGALAGV-VGVCLGGYLMETT
```

```
Hydra2                      NAFAA-SSVAINPQDLSPE-QAGVLYGVANTCGALGGF-LGVLISGIILSNS
Chrysochro1                 GGFAVGAGYWANFVDLSPR-HSQVLLAISNSFASVPGI-LGVSLTGSLLSST
Micromonas                  TAFTQ-AGFLVNFQEIGPR-YVGALHGMANTAGSAAGI-VGTYGAGVILGK-
Bathycoccus1                TAFSQ-SGFLVNFAEIGPK-YASTIHGVANTAGSIAGM-VGTYVVGWILSHS
Amphiamblys                 NALSI-IGVQVAHMEIGPR-YGGVLFGIINTVCILAGL-VGVKTSGWILEAT
Bathycoccus2                GAWVR-TGLFCGHQDLSPK-YASIMLGVTNTAAAIGSL-LSTFFIGYFMEVT
Guillardia                  SGLSN-AGYSANYLEISAG-LSGILISLGNTLATVPGM-VSPVLTGVIMDAH
Basidiobolus                NSLTL-IGVSISQLDIAPR-FAGIIFGIGNTAATLPGI-LGVTVTGLLLESK
Congregibacter              SSMAW-AGFACNHLDVAPK-HADVLFSITNIGGTLPGI-VGVALTGVLVDLT
Sphingorhabdus              GGFAV-SGFFVNHMDIAPE-HAGTLMGITNTAGTIPGI-VGVMVSGWILDST
Sphingomonadales            GAFAV-SGFFVNHMDIAPE-HAGTLMGITNTAGTIPGI-IGVLVSGWILDVT
Thelohanellus               LAPFQ-AGYFTAIIEHAPQ-YSGVTFSFINIGGTFSGV-VQRFLVAKLSERI
Chrysochro2                 -AANG-GGYAVNHLDIAPT-IASLVLAFYNTGGQVAGW-MAPWAIGVLTAYP
SLC17A1_HSA                 GSFCL-GGVFINGLDIAPR-YFGFIKACSTLTGMIGGL-IASTLTGLILKQD
SLC17A3_HSA                 STLCQ-SGIYINVLDIAPR-YSSFLMGASRGFSSIAPV-IVPTVSGFLLSQD
Strongylo3                  TGLAM-SGASMCQLDFAPR-YSGIISAVVNMFAATSGF-LAPLLIGRFTENQ
Strongylo5                  TGLAY-SGSCLSMMELATP-YAGMVVAVSYSIATFTGF-ISPAVVAMYTENQ
Strongylo1                  TGLVY-SGASLTQMEFATP-YAGIVTAIANTFANTPGF-LSPLIAGVVTENQ
Ciona1                      RGFTY-AGHNSSPIDIAPM-YAGVVFGISNTIASITGF-LGPLTAGLVITDK
Drosophila2                 HGAVS-SGPLASMVDLSPN-YAGIVLGVSGMIGGMPGF-ISPLIVGQLTYQN
Strongylo2                  NGICT-CGYYVNLMDIAPR-LAGSLIGLVNSSSAFSGA-ISPYVVGVLTPNQ
Drosophila1                 NGAAT-ASNLANSQDLAPN-YAGTLYGIINCVGTTPGI-FSPLIVAAFTKNE
Drosophila4                 HGAVT-AGYLGNGLDIAPN-FGGTIFGLANTLSSFGGF-LSTWMVGALTFKD
Drosophila3                 MGTYY-AGMKLTPLDLSPN-YAGTLMAITNGIGAITGV-ISPYLVGVMTPNA
Aplysia4                    MGLTI-SGFTVNHLDLAPA-YAGILFGITNTMGTVPGM-IAPLVAGAMTPNR
Aplysia1                    EGLCY-AGYMVNQVDFAPR-YAGVLFGITNFISTIPGT-LAPIVTGKLTPNN
Capitella                   TAFGR-SAYSVNHVDIAPR-YGGALFSLSNTVATFPGF-ISPAVVAALTPNG
Hydra1                      ETLAW-AGFGINHLDIAPR-YASVLFGITNTCATVPNI-LSPILVGVLTDLG
Caenorhabditis              SGLQY-AGFVVNYLEIAPP-FSGTVMGTGNTISALAGI-ISPAVSSYLTPNG
SLC17A8_HSA                 SGFAI-SGFNVNHLDIAPR-YASILMGISNGVGTLSGM-VCPLIVGAMTRHK
SLC17A6_HSA                 SGFAI-SGFNVNHLDIAPR-YASILMGISNGVGTLSGM-VCPIIVGAMTKNK
SLC17A7_HSA                 SGFAI-SGFNVNHLDIAPR-YASILMGISNGVGTLSGM-VCPIIVGAMTKHK
Tribolium                   GVFSW-SGFGVNYLDIAPQ-HASVIMGVSNTFGTLAGI-FSPIVTGYIVTTP
Aedes                       GAFAW-SGFAVNHLDLSPK-SAGVLMGISNTFATIPGI-VSPILTGYITSNK
Amphimedon1                 VGLAN-AGFNINHIDIAPR-FAGVLMGITNCAATIPGI-VAPTVAKYIAQKP
Amphimedon2                 SALIQ-AGFMINHIDLAPR-FAGVLMGITNTFGTLPGI-VSPVVAKYIAQKP
Amphimedon3                 GALIQ-AGFMINHIDLAPR-FAGVLMGITNTFGTIPGI-VAPVVAKYMAQEP
Oscar                       GGMAM-SGFNVNHLDIAPH-YAGILMGITNAAGTLPGF-IGPYVAGAMTPAH
Acropora                    SNFNA-ASFNVNHLDIAPR-YAGVLMGITNAAGTIPGI-VGPFVVGVLTNNE
Exaiptasia2                 NGLTA-SGFLVNHLDIAPS-QLVVNG-KRFSILLLPSTSLVP----------
Nematostella                TGFNL-SGFNVNHLDIAPR-YAGILMGITNSFGTVPGI-AAPYVAGWITNKK
Exaiptasia1                 TGLGS-SGYNINHLDIAPR-YAGVLMGITNMFATIPGI-VSPYVTGWITNDN
Aplysia2                    TGTQY-SGFLVNHVDIAPA-FAGILFGISNSIAAVTGF-ISPVIVGVITEER
Trichuris                   SGLCG-GGFFVNYLDLCPQ-YTGLVFGISNTLATIPGI-LGPNITGWLTKGE
Daphnia                     SGASY-SAFQVNFVEIAPP-YAGTLFGVTNAVANLCGF-MAPYAVGVLVKGN
Sialin_HSA                  GGFCS-SGFSINHLDIAPS-YAGILLGITNTFATIPGM-VGPVIAKSLTPD-
Aplysia3                    ASFNG-GGFSVNHLDLSPR-FAGILLGLTNTVATIPGF-VSPALVGYLTDGN
Crassostrea                 GGFCM-GGFNCNHIDIASN-FSGTLMGITNMFATIPGF-LGPAVVGWLTSHE
Biomphalaria                QGCIM-GGYNINHLDIAPK-FAGVLMGITNSLGTIPGF-MGPAVVGYLTNNN
Lottia                      GGFSM-AGYQVNHLDIAPR-FAGSLMGITNMIATIPGF-LAPLVVGILTDHQ
Ciona2                      NGAGF-PGFNTNHVDIGPR-YAGILMGITNTWATIPGF-AAPAVVGLLTENN
Strongylo6                  GGFAM-SGFNVNHLDIAPA-YGGLLMGITNTIGTIPGI-LGPSMVGLYVTNQ
Klebsiella_Nant             GQGIS-GILPKLIGGYFETDQRAAGLGFTYNVGALGGA-LAPILGALIAQRL
Escherichia_nant            GQGIA-GILPKLIGGYFDTDQRAAGLGFTYNVGALGGA-LAPIIGALIAQRL
Acyrthosiphon_sv2           GGLAS-GLTPA-FSELYSTYIRGSGAGFCASFGRGFGS-LMP---AIVGIAM
Fonsecaea_Nant              VQGAW-GVIPIHLMELSPGSLRTFVVGTSYQLGNLASS-ASSTIESTIGERY
Calocera_nant               VQGAW-GVIPIHLAEISPPAFRATFPGVAYQLGNMVSS-ASAQIEATGGEHQ
Hebeloma_nant               VQGAW-GVIPIQLAEMSPPAFRATFPGVAYQLGNMVSS-ASAQIEATAGEHL
Gymnopus_nant               VQGAW-GVVPIQLAELSPPAFRATFPGVAYQVGNMVSS-ASAQIEATGGDHQ
Mycobacterium_Nant          VQGAW-GVIPAHLTEMSPDAIRGVYPGVTYQLGNLLAA-FNLPIQERLAESH
Drosophila_nant             IQGAW-GVVPAYLSELSPASIRATFPGLAYQCGNLLAA-SNALLQTEIGRVL
Amphimedon_sv2              TIPAW-SSTTVLVADLFPTALRSTAVGVFLLLARIGAI-IGTYTFGLFTSTS
Orbicella_nant              ISASF-ALLYVYSAELFPTVVRNAGMGISSLSARLGGI-VAPFIVLLGDQR-
Strongylocentrotus_sv2      ISGAF-QALFVYTPEVFPTNVRSVGLGWCVAFSKLGSI-VTPFVAQVLIKQ-
Homo_sv2                    ISGGF-QAAYVYTPEVYPTATRALGLGTCSGMARVGAL-ITPFIAQVMLES-
Daphnia_sv2                 ISGVF-QAAYVYTPEVYPTYLRSVGIGVCSGMARLGAM-VTPFVAQVLVQD-

Dictyostelium               -------------------------------------------
Emiliania                   --DFTPMFALVVAVELAGLVAWNRCLVFTLFCEQTRNRKGDRQ
Cryptosporidium             IERWKWVLAIYAIHNIIGGLLFVL------LAD----------
Aureococcus                 --SFRACFALAAAIYASEFAVFFG------FVR------GGRL
Enterobacter                --STTPFFIAAMGFNLLAVIFWLR------LSS------GKRE
Chromera                    --SEVPFFYLAAGLNLLAIV-----------------------
Cellvibrionaceae            --SVTPFFLIGAGLCVMGILCWSK------MDA------EKPL
SLC17A9_HSA                 --SWTCLFNLVAIISNLGLCTFLV------FGQ------AQRV
Hydra2                      --EWFRVFIMTSVISFLGFLVYIM------YGS------GK--
Chrysochro1                 --DWRIIFLIAGAVEFGGALIFIA------FAE------AAEQ
```

```
Micromonas                  TGSWSAVLLITAGVYAFGAVFWLA------FST------GE-R
Bathycoccus1                ESGWTNVMYMTSAVYVVGALVWLT------FMS------GE-Q
Amphiamblys                 --RWDVVWGVCCLSFAAGALVWGC------FYR------AE-L
Bathycoccus2                SWAWSLFYPIAALQVASALLFSAL------WKS------TP-I
Guillardia                  QQAYQTVFWIAFMVYTFGAVFYAT------FAT------SE-R
Basidiobolus                --AWPLVFGLAASFYFVGAWTWLF------WGG------GS-Q
Congregibacter              --GYTATFVVAAGINVFGAIVWLL------FAT------GE-P
Sphingorhabdus              --SWALVFQVAAAVSAFGLLFYLL------FGS------GE-R
Sphingomonadales            --SWALVFQVAAGVAAFGLIFYLI------FGS------GE-R
Thelohanellus               RVAYKYSFIITGIVSFVLSMVYVV------FGT------ADLQ
Chrysochro2                 DDEWRIVFGLGGLLQAAGGLAFLA------LAS------DRVQ
SLC17A1_HSA                 ESAWFKTFILMAAINVTGLIFYLI------VAT------AEIQ
SLC17A3_HSA                 EFGWRNVFFLLFAVNLLGLLFYLI------FGE------ADVQ
Strongylo3                  PRGWKIVFWTSAGIMMFGLVFFLI------FGT------AEIQ
Strongylo5                  IAGWRSFFWVTFGITVVAWLLFMI------FGT------SELQ
Strongylo1                  MLGWRKMYWIAFGIAIFAEVIVLL------FCT------SELQ
Ciona1                      IGQWQTMFWINGALGFFGSIFFAC------FAS------EVER
Drosophila2                 IEAWKNVFLLSSFMLTGSGILYVL------FSE------STLQ
Strongylo2                  ITGWQTVFIICCGINSFACLVFLV------FGS------GKEQ
Drosophila1                 IDQWHWIFIIGAAAYILPALFFWV------FGS------GKIQ
Drosophila4                 FHSWQIVFWILGATYISGAVVFAI------LGS------GELQ
Drosophila3                 MMEWRIVFWVAFAVLFITAIVYCI------WAS------GEVQ
Aplysia4                    AEEWRSVFYVCGAVAGVGGIIYVI------LAD------GELQ
Aplysia1                    QSEWRTVFYLCGAFTLIGTIIFGG------FAS------GEIQ
Capitella                   QAEWKIVFFSCGAVYAIGGVIYII------FAR------ADVQ
Hydra1                      RQHWDRVFYISAAMFLFGALFYGF------FAS------GERQ
Caenorhabditis              QEEWQMVLWLTAGILTIGALLFSI------FAS------GEVQ
SLC17A8_HSA                 REEWQNVFLIAALVHYSGVIFYGV------FAS------GEKQ
SLC17A6_HSA                 REEWQYVFLIAALVHYGGVIFYAI------FAS------GEKQ
SLC17A7_HSA                 REEWQYVFLIASLVHYGGVIFYGV------FAS------GEKQ
Tribolium                   ADEWQIVFFIASGLFVLGSIVYGI------FAS------GEVQ
Aedes                       DDEWRVVFYIAAGIYLIGCVIYWI------WAS------GELQ
Amphimedon1                 QAEWREVFIIAAEVYLFGAAVYLI------LAE------GTKQ
Amphimedon2                 QEEWREVFLISAEIYIFGAAVYLI------LAD------GKKQ
Amphimedon3                 QEEWRKVFLISVQVYIFGAAVYLI------LAD------GKKQ
Oscar                       QLEWRNVFYLAAEIYAFGGLAFLI------LSS------GELQ
Acropora                    REQWQKVFYISAGVYVLGWLTFTL------LAS------GKQQ
Exaiptasia2                 -------------------------------------------
Nematostella                RAHWQIVFFISAGLYVIGAISFVT------LSK------GSER
Exaiptasia1                 RAQWQKVFYLSAILYVVGGISYLI------LGT------GKEQ
Aplysia2                    RAEWQIVFYVAAAIYIFGAVFYII------FAS------GELQ
Trichuris                   FESWRIVFFIAGALYFFSVIFYAI------FGK------GTLQ
Daphnia                     LGQWRLVFLIAAGVYCVSNTIFLI------FGS------SQVQ
Sialin_HSA                  VGEWQTVFYIAAAINVFGAIFFTL------FAK------GEVQ
Aplysia3                    RAQWQIVFYVAASIYALGALIFAL------LAR------GVEQ
Crassostrea                 RAKWQIVFYISAAIYVTGCILFNV------FAR------GEEQ
Biomphalaria                RGQWQIVFYITAGMCFVGAIGYDL------LAK------GEEM
Lottia                      SGQWRIFFFITAAIYIVGAILFLA------LSK------GEEL
Ciona2                      RSQWRIVFYIAAGVYLTGTILYSL------MAT------GEEQ
Strongylo6                  ISQWQIAFWVCCGVYMFGAVTYLL------MGT------GELQ
Klebsiella_Nant             ---------TALGSLSFSL--TFVVILLI--G---LDMPTRVQ
Escherichia_nant            ---------TALASLSFSL--TFVVILLI--G---LDMPSRVQ
Acyrthosiphon_sv2           -------------------------------------------
Fonsecaea_Nant              KAVKRYNYGKVICIFMGAVYAYTILLTFV--G---PERRG--R
Calocera_nant               -GQDAPDYAKVQGIFLGVVAAFVIFMTLI--G---AENHS--S
Hebeloma_nant               VPKNVPDYATVQGILIGVVAAFVVIVTII--G---PENHG--S
Gymnopus_nant               GMKVVPDYATVQGIFIGCIAAFLLVVTLF--G---PEKHG--Y
Mycobacterium_Nant          ------PFALAATIVPVL--LVVAVLTAI--G---KDATG--I
Drosophila_nant             ------GLAPALMLTVGLAALAVLVLTLL--N---AKLHP--Q
Amphimedon_sv2              ----VPVLLTA-AILLGS-----AILSLM-LP---WTTRK---
Orbicella_nant              ----LPMFVFA-CTALLA-----ALVGLK-L----RETQG---
Strongylocentrotus_sv2      ----MTFCVYG-GCAVFA-----SLLAFI-LP---TETKG---
Homo_sv2                    ----LTLAVYS-GCCLLA-----ALASCF-LP---IETKG---
Daphnia_sv2                 ----VAIGLYG-SVSLLA-----MVASLC-LP---IETKG---
```

6 – NANS

```
Aureococcus_anophagefferens_ACJI01001814.1      -----------------------------PCKVIAEIGCNHMGSFETAKELMLLAKATGA
Treponema_pallidum_SpsE_NP_219001.1             -----------------MFTCGGRCFRPDADILTIAEIGSAHAGSFDRARALIDAAADAA
Reticulomyxa_filosa_ASPP01005516.1              -----------------------REIGPAHPPLVIAEIGINHGGSLEVAKEMVRLAHLSG
Neisseria_meningitidis_NeuB_NnaB_1XUU           ------------MQNNNEFKIGNRSVGYNHEPLIICEIGINHEGSLKTAFEMVDAAYNAG
Neisseria_meningitidis_NeuB_2WQP_A              ------------XQNNNEFKIGNRSVGYNHEPLIICEIGINHEGSLKTAFEXVDAAYNAG
Acanthamoeba_palestinensis_CDFD01053174.1       --------------PMNSIIIQNRPVGAAHSPYIVAELSGNHNGSIERAKDIILEAHKCG
Cladosiphon_okamuranus2_BDDF01000073.1          -------------------LINGRKIGTEYPPFIIAEMSGNHNQSLDRAMQLVESAAESG
Cronartium_ribicola_AWVX0102625.1               -----------------------QFIGVNHRPFIIAEMSGNHNQSLDRALAIVEAAAACG
CBA31353.1_Curvibacter                          ---------------MKNISIAGRLIGKAWPPFVIAEMSGNHNQSLERALEIVDAAAQAG
Hydra_vulgaris_ABRM01085349.1                   ----------------------GRLIGKAWPPFVIAEMSGNHNQSLERALEIVDAAAQAG
WP_018506507.1_pseudaminicAS_Thiobacillus       ---------------MKPIQIAGRQIGPSYPPFIIAEMSGNHNQSLERALEIVEAAAKSG
WP_009207339.1_pseudaminicAS_Sulfuricella       -----------------MIKIGSREIGRQQAPFVIAEMSGNHNQSLERALEIVEAAAKTG
WP_067217189.1_pseudaminicAS_Marinomonas        -----------------------MVNVMLNSPFIIAELSGNHDQDFDTAKAMIKAAAEAG
WP_087462101.1_pseudaminicAS_Oleiphilus         ----------MTQA-LSEVRIASRRVSDQEDPFIIAEFSGNHAQDLAIAEEMVRAAARAG
Bankia_setacea_MRUH01000164.1                   ----------------------GREISAAAAPFVIAELSGNHAQNLDTAKAMIMAAAAAG
KIX22924.1_NeuB_PseudAS_Flavobacterium          -------------------------MHKNSPVFIIAELSANHNGSLETALETVKAAKRAG
XP_001618767.1_NANS_Nematostella                ------------------------------------------------------------
WP_092376349.1_pseudaminicAS_Desulfomicrobium   ----------------MTVMINGRAIGPDHPPYIIAELSANHNGSLERALQTIEAAHRCG
Condylostoma_magnum2_CVLX01030206.1             ----------------------GRAIGPDHPPYIIAELSANHNGSLVRALETLEVAKQCG
AAD45660.1_NeuB_Aeromonas                       ----------MDKIIKPFITINGRKIGPDYPPYIIAELSANHNGDINRAFAIMEEAKKAG
WP_094706949.1_pseudaminicAS_Hahella            ------------MAFKNTVTINGRTLGNGHPPYIIAELSANHNGSLERALSIIDMAHRCG
Aureococcus_anophagefferens_ACJI01001814.1      ------------------------------PCKVIAEIGCNHMGSFETAKELMLLAKATG
Emiliania_huxley_AHAL01004984.1                 -----------------------MTRIDFKQPHVMAEIGCNHMGEFETAKELLTLAKEAG
Melampsora_pinitorqua_AUYS0100826.1             ---------------------------------IIAEIGQNHNGDMDAAEALIRAAKAGG
EEN59689.1_NANS_Branchiostoma                   ------------------------MVGGDHPCFVVAEIGQNHQGDINIAKEMIKKAKEAG
Q9VG74.2_NANS_Drosophila                        --------------MLLNDIISGKL---VDSVYIIAEIGQNHQGCVETAKKMIWEAKKAG
EFX62694.1_NANS_Daphnia                         ------------MTARSFELV-------------------------NNTWIGIKMAKDCG
XP_008190466.1_NANS_Tribolium                   --------------MAELQITPKRTIGHNHPTFIIAEIGQNHQGDVKIAKKLIHLAKESG
Q9NR45.2_NANS_HSA                               -------------MPLELELCPGRWVGGQHPCFIIAEIGQNHQGDLDVAKRMIRMAKECG
XP_783181.3_NANS_Strongylo                      -------------MPLEFEIAPGRMIGNDHPCFIIAEIGQNHQGDINIAKQLIKVAKDAG
KMQ51676.1_Chitinispirillum                     ---------------MGVISLGSKLVGAGHPVFVIAEVGINHQGDIDIAKKLIKEAKNCG
OUX54928.1_Flammeovirgaceae_bacterium           --------------MKRNFILPNNTLDNNSSAFIIAEIGINHQGSIDIAKKLILQAKDCG
OUV47462.1_Flavobacteriaceae_bacterium          ---------------MSVIELDKKKVGENHPTYIIAEIGINHQGDLDTAKELIQKASEAG
OIO66895.1_Candidatus_Marinimicrobia            ---------------MSTLQLGSKTVGDGNPTYIIAEIGINHQGDVKIARELVRQAALAG
WP_027268045.1_Legionella                       --------------MRRVLNLGHREISDSSSCYVIAEIGHNHQGSLEKCKDLIQAAVEAG
OGP61643.1_Deltaproteobacteria                  --------------MKRQIAINGRVINDETDCYIIAEIGHNHQGDLQKAKELFNVAKACG
WP_022662582.1_Desulfovibrio                    ---------------MRSLKLNGTIVNDENEAYVIAEIGNNHQGDVEICKQMFDAAKECG
OGP71522.1_Deltaproteobacteria                  ---------------MPELVIDGRLINPEGDCYVIAEVGHNHQGDLETARQLFRAAQEAG
Cladosiphon_okamuranus_BDDF01000009.1           --------------------------------YIIAEIGINHNGDLSKALEMVRKSKEAG
Elaphomyces_granulatus_NPHW01007775.1           -------------------------VGDGYPVYIIAEIGINHNGSLEIAKKMIDGAVFAG
Thermobifida_fusca_NeuB_YXQ47U14                --MTQNATTDLSVSVPAPVAIGDILVGPGQPTYVIGEIGINHNGDVDIARQLIEVAAEAG
Acytostellium_ellipticum_FLTD01002867.1         ----------------MTVSIGSRVIGGGRPAYVIAEIGLNHNGDVDIAKKLIDVAARAG
Dictyostelium_deminutivum_FLTF01003146.1        ----------------MTVSIGSRVIGGGRPAYVIAEIGLNHNGDVDIAKKLIDVAARAG
Lentinus_polychrous_JSYW01008743.1              ------------------------------------------------------------
WP_035074769.1_Desulfovibrio                    ------------------MK-ISNFDT-DKKIFIIAEIGNNHEGDFELAKNMVIKAVAAG
SBT09427.1_Candidatus_Propionivibrio            ------------------MK-IGDIDL-SREVMVIAEIGNNHEGDFGLARDMILAAAAAG
WP_008313751.1_NeuB_Leptolyngbya                ------------------MK-IGRHSL-EQDVFVIAEIGNNHEGDIQLAQEMIHAAAASG
WP_096701985.1_Magnetospirillum                 ------------------MK-IGPIDL-DRDVLIVAEIGNNHEGDMALAEEMIAQAAQAG
Chromera_velia_ARZB01016018.1                   MLVAEQDGDVVGFLQALQSS-NGTLVIDLLAVLVVAEIGNNHEGDLGRAEEMIRRAAEAG
ABS75841.1_SpsE_Bacillus_velezensis             ---------------MASCQIGTKSIGEGSPVFIIAEAGINHDGKLSQAFELIDAASEAG
Bacillus_subtilis_P39625.1_SpsE                 ---------------MAAFQIANKTVGKDAPVFIIAEAGINHDGKLDQAFALIDAAAEAG
Prorocentrum_minimum2_JXLM01007734.1            --------------------------------YVIAEIGVNHEGDFDTAKRLIDLAKEGG
Phytophthora_lateralis_AMZP02003661.1           --------------------------------FIIAEAGVNHNGDEKMAVELVEAAAKSG
AAC43302.1_neuB_Escherichia                     ----------------------------MSNIYIVAEIGCNHNGSVDIAREMILKAKEAG
ABD95628.1_NeuB_Streptococcus                   ------------------------------MVYIIAEIGCNHNGDINLAKKMVDVAVSCG
Tuber-aestivum_CZPR01000680.1                   -------------------------------VYIIAEAGVNHNGERDLAFALVDAAAAAG
Methanococcoides_burtonii_NeuB_NnaB_WP_011499634.1  ------------------MQKMDRI-SENAPVFIIAEAGVNHNGSIALAKQLIDVAAKSN
AEG18372.1_Methanobacterium                     ------------------MEEIDKFNKDNCSCFIMAEAGVNHNGSLNLAKKLIDAAKESG
WP_045444639.1_Neub_Tepidicaulis                -------------------------MSKPPYVEIIAEAGVNHNGSLERALKLVDVAADAG
WP_029638846.1_NeuB_alphaproteobacterium        ----------------------------MNHVTVIAEAGVNHNGSLERAIEMVDVAADIG
Prorocentrum_minimum1_JXLM01000037.1            --------------------------------SVIAEAGVNHNGSLERAIEMIDVAADIG
Paramecium_biaurelia_JPFL01000669.1             -------------------------------VLIIAEAGVNHNGELQKAIELIDAAAHAK
ADL05453.1_Clostridium                          ----------------------------MSKIMIIAEAGVNHNGDITIAKQLIDAASVTG
EFM37961.1_Campylobacter                        ----------------------------MKKTLIIAEAGVNHNGDLNLAKKLIEIAADSG
EIJ34212.1_Thiothrix                            -----------------------------MSVFIIAEVGVNHNGSLDLAKQLVDVANLCG
Condylostoma_magnum_CVLX01013231.1              --------------------------------IIIAEAGVNHNGDINLAKALIEKAAMAG

Treponema_pallidum_SpsE_NP_219001.1             AAAVKFQLIYAHEILHPLTGAVRLPSGAVSLY-------QRFE-ELEVPLSFYAQC----
Reticulomyxa_filosa_ASPP01005516.1              CECIKHQTHFLDDEMTDEAKAIFPPNADVSIWD-VMARCALSA-------GDEVAL----
Neisseria_meningitidis_NeuB_NnaB_1XUU           AEVVKHQTHIVEDEMSDEAKQVIPGNADVSIYE-IMERCALNE-------EDEIKL----
Neisseria_meningitidis_NeuB_2WQP_A              AEVVKHQTHIVEDEXSDEAKQVIPGNADVSIYE-IXERCALNE-------EDEIKL----
Acanthamoeba_palestinensis_CDFD01053174.1       ANAIKIQTFKPDSITLPCYRDEFMIKK--GIWQ-GSNLYELYK-KTCLPYEWHQEL----
Cladosiphon_okamuranus2_BDDF01000073.1          VHALKLQTASPEGLTLDIESPEFMIDDPASPWH-GRNLYQLYK-EAVTPWEWHKPI----
Cronartium_ribicola_AWVX0102625.1               AHALKLQTYTADTMTLDLSHGEFFISDPASLWK-DTSLHKLYQ-EAYTPWEWHKPI----
CBA31353.1_Curvibacter                          AHGLKIQTYTPDTMTLDLDEREFHIDDAKSLWK-GTSLYKLYG-QAYTPWEWHKPI----
Hydra_vulgaris_ABRM01085349.1                   AHGLKIQTYTPDTMTLDLDEREFHIDDAKSLWK-GTSLYKLYG-QAYTPWEWHKPI----
WP_018506507.1_pseudaminicAS_Thiobacillus       AHALKIQTYTPDTMTIDLDEREFHISDSNSLWK-GASLYKLYG-EAYTPWEWHKPI----
WP_009207339.1_pseudaminicAS_Sulfuricella       AHALKIQTYTPDTMTLDLNEREFYISDPKSLWV-GTSLYKLYG-EAYTPWEWHKSI----
WP_067217189.1_pseudaminicAS_Marinomonas        VDAIKLQTYTADTMTLNVRNEEFMIGEADSLWQ-GQNLYDLYG-KACTPWEWHQPL----
WP_087462101.1_pseudaminicAS_Oleiphilus         VDAIKLQTYTADTITLDGKSREFQIRDDSSLWK-GETLHSLYK-KACTPWAWHSQL----
Bankia_setacea_MRUH01000164.1                   ADAIKLQTYTADTITLNSTNPEFQINEASSLWQ-GENLHSLYQ-KAHTPWQWHAEL----
KIX22924.1_NeuB_PseudAS_Flavobacterium          ANCIKLQTYTADTITLDSEKPDFVIKG--TIWD-GRKLHDLYK-EAYTPWEWHEKI----
XP_001618767.1_NANS_Nematostella                ------------------------------------------------------------
```

```
WP_092376349.1_pseudaminicAS_Desulfomicrobium          ADAVKLQTYTADTLTIDCDAPDFLIKG--GLWD-GFKLYDLYK-WAETPYEWHQAM----
Condylostoma_magnum2_CVLX01030206.1                    ADAIKIQTYTADTMTINCSMPDFMIKG--GLWD-GFKLYDLYK-WAETPYEWHKPL----
AAD45660.1_NeuB_Aeromonas                              ADAIKLQTYTADTITFECDSEEFQIHG--GLWD-GKNLYQLYK-EAQMPWEWHQPL----
WP_094706949.1_pseudaminicAS_Hahella                   ADAIKLQTYKADTITFDSDKEDFQIHG--GLWD-GKTLYELYE-WAHMPWDWHKPL----
Aureococcus_anophagefferens_ACJI01001814.1             AGYAKFQKRCPRELLTKEQYDAPHPVP-HNAYG-QT-YGAH-REFLEFSKEQHEQL----
Emiliania_huxley_AHAL01004984.1                        ASVGKFQKRCPKELLTPEQYAAPHPNP-RNAYG-DT-YGAH-REYLELTVDQHREL----
Melampsora_pinitorqua_AUYS0100826.1                    ADVAKFQVYDARALFPPKE-------S--NPWF------DY-NCKTELQRGDVVRL----
EEN59689.1_NANS_Branchiostoma                          ADAAKFQKSELTRRFNRAALARPYTSP--NAWG-PT-YGET-NSTWSSATISTRSSRLTP
Q9VG74.2_NANS_Drosophila                               CHCVKFQKSDLPAKFTRSALDREYISD--HAWG-KT-YGEH-KEYLEFSKDQYLQL----
EFX62694.1_NANS_Daphnia                                ADCVKFQKSDLHSKFTKSVLARHYESP--HAWA-ST-YGEH-KTFLEFSEDHYKEL----
XP_008190466.1_NANS_Tribolium                          ADCVKFQKTCLTEKFNKSALDRPYRGP--HSWG-AT-YGEH-KQFLEFTKEQFREL----
Q9NR45.2_NANS_HSA                                      ADCAKFQKSELEFKFNRKALERPYTSK--HSWG-KT-YGEH-KRHLEFSHDQYREL----
XP_783181.3_NANS_Strongylo                             ADCAKFQKSELDFKFNQAALEKPYTSK--HSWG-KT-YGEH-KRFLEFSHDQYREL----
KMQ51676.1_Chitinispirillum                            ADAVKFQKRSISRILTREGLEMPYENS--NSFG-KT-YGEH-KRALELDETDYVEL----
OUX54928.1_Flammeovirgaceae_bacterium                  ANAVKFQKRSISRILTKEGLEMPYKNR--NSFG-KT-YGEH-KYALELSKDDYREL----
OUV47462.1_Flavobacteriaceae_bacterium                 ANAVKFQKRSIQRILTQEGLNAPYENP--NSFG-KT-YGEH-KKALELSKSDYAEL----
OIO66895.1_Candidatus_Marinimicrobia                   ADAVKFQKRNVERILTRDGLEMPYDNP--NSFG-KT-YGEH-KRALELTEGDYHEL----
WP_027268045.1_Legionella                              ADAVKLQKRNNRNLFTREMYNSPYNSE--NAFG-ST-YGEH-REFLEFGKLEYLEL----
OGP61643.1_Deltaproteobacteria                         AEAVKLQKRNNRKLFTKELFNKPYENP--NSFG-ET-YGEH-REYLEFGKKEYEEL----
WP_022662582.1_Desulfovibrio                           ANAVKLQKRDNRSMFTKAAFNQVYNSE--NAFG-RT-YGEH-REFLEFGEREYAVL----
OGP71522.1_Deltaproteobacteria                         AEAVKLQKRDNRSLYTRAMYTQPYDHE--NSFG-AT-YGEH-REALEFGESQYREL----
Cladosiphon_okamuranus_BDDF01000009.1                  CDAVKFQKRTIDIVYSREELARPREN----VFG-PT-NGDL-KSGLEFGKDDYDQI----
Elaphomyces_granulatus_NPHW01007775.1                  ADAVKFQKRTPEFCVPQHQWNIERDT----PWG-RMTYLEY-KRKIEFNFDDYERI----
Thermobifida_fusca_NeuB_YXQ47U14                       CHAVKFQKRTPEICVPKEQRDKIRQT----PWG-EMTYLEY-KHRVEFGRDEYTEI----
Acytostellium_ellipticum_FLTD01002867.1                ADAVKFQKRTPEISTPEHMRDVPRET----PWG-VMSYLDY-RRRVEFGRDEYVEI----
Dictyostelium_deminutivum_FLTF01003146.1               ADAVKFQKRTPEISTPEHMRDVPRET----PWG-VMSYLDY-RRRVEFGRDEYVEI----
Lentinus_polychrous_JSYW01008743.1                     ADVVKFQIYTGTALVSPVESPD---------------RHAH-FKRFELTPDQHIAL----
WP_035074769.1_Desulfovibrio                           ADAVKFQTIVPCELVSSDQLE----------------RLAM-LEKFQFSYEQFAEL----
SBT09427.1_Candidatus_Propionivibrio                   AHAVKFQTIQPDQLVSASQPA----------------RLEQ-LGRFAFSRDEFAEL----
WP_008313751.1_NeuB_Leptolyngbya                       VQAVKFQTIIPERLVAKSQSA----------------RISQ-LSRFAFSPDQFANL----
WP_096701985.1_Magnetospirillum                        AHAVKFQSIIPAHLVAPDQPA----------------RLAQ-LGRYQLSAENHVRL----
Chromera_velia_ARZB01016018.1                          AQAVKFQTIDPARLVAADQTA----------------RLQQ-LQRFALTPDDHRHL----
ABS75841.1_SpsE_Bacillus_velezensis                    ADAVKFQMFQADKMYQKDPGLY---KT--AAGK-NVSIFSL-VQSMEMPAEWIEPL----
Bacillus_subtilis_P39625.1_SpsE                        ADAVKFQMFQADRMYQKDPGLY---KT--AAGK-DVSIFSL-VQSMEMPAEWILPL----
Prorocentrum_minimum2_JXLM01007734.1                   ADAAKFQTYKAEKIASKHSPAY--WDQ--NAEP-TNSQRELFLKYDAFEEKDYVAL----
Phytophthora_lateralis_AMZP02003661.1                  ADAVKFQTFSADKLTRKGAEKAEYQKL--ATGD--GDQHGM-LKALEMSESLHRRL----
AAC43302.1_neuB_Escherichia                            VNAVKFQTFKADKLISAIAPKAEYQIK--NTGE-LESQLEM-TKKLEMKYDDYLHL----
ABD95628.1_NeuB_Streptococcus                          VDAVKFQTFKAEKLISKFAPKAEYQKA--TTGT-ADSQLEM-TKRLELSFEEYLEM----
Tuber-aestivum_CZPR01000680.1                          ADAVKFQTFDAARLASKSAPKATYQKQ--TTDA-AESQLAM-LKKLELPKEWHADL----
Methanococcoides_burtonii_NeuB_NnaB_WP_011499634.1     ADAVKFQTFVTEDVVSINTPKAEYQKH--TTES-SESQFEM-IKKLELSKTDHKEL----
AEG18372.1_Methanobacterium                            ADAVKFQTFKTENLVTKNAEKAEYQKE--TT-T-ENSQYEM-IKKLELSEDNFREL----
WP_045444639.1_Neub_Tepidicaulis                       ADVIKFQTFSADAIATKAAPKAAYQKL--ETGKA-QSQYEM-LKALELDEAAHRTL----
WP_029638846.1_NeuB_alphaproteobacterium               VDVVKFQTFNAEALVTRSAPKADYQKE--TTEESE-TQLDM-LRALELDVHAHRTL----
Prorocentrum_minimum1_JXLM01000037.1                   VDVVKFQTFNAEALVTRSAPKADYQKE--TTEESESTQLDM-LRALELDAQAHRTL----
Paramecium_biaurelia_JPFL01000669.1                    ADYVKFQTFKAEKIVNPTAKKAQYQVT--NMQGEGDTQFEM-LKKLEMGDDWYPIL----
ADL05453.1_Clostridium                                 ADAIKFQTFKTDLLVVPASGKAEYQIK--NTGG-AMSQYEM-LKNLELSWQAFEEL----
EFM37961.1_Campylobacter                               ADFVKFQSFKAKNCISTKAKKAPYQLK--TTAS-DESQLQM-VQKLELDLKAHKEL----
EIJ34212.1_Thiothrix                                   ADAVKFQTFKASTLVTKTARQADYQTT--NTQK-EESQFDM-LTRLELSEAGHHAL----
Condylostoma_magnum_CVLX01013231.1                     ADYVKFQSFKASELTTKLAQKADYQKE--NTNP-TDSQLDM-IKALELSYENHKSL----

Treponema_pallidum_SpsE_NP_219001.1                    -FNHARSRGMLVGISPFGPRSATEA-LALKPD-FLKVASPELN-YPTLISTLA-A---AE
Reticulomyxa_filosa_ASPP01005516.1                     -KDYTESLGLIYISTPFSRKAADFL-AEIGVP-AFKIGSGEAD-NLPLIRHIA-R---FG
Neisseria_meningitidis_NeuB_NnaB_1XUU                  -KEYVESKGMIFISTPFSRAAALRL-QRMDIP-AYKIGSGECN-NYPLIKLVA-S---FG
Neisseria_meningitidis_NeuB_2WQP_A                     -KEYVESKGXIFISTLFSRAAALRL-QRXDIP-AYKIGSGECN-NYPLIKLVA-S---FG
Acanthamoeba_palestinensis_CDFD01053174.1              -FSYAKNVGITIFSTPFSEADVEFL-ESLDTP-AYKIASNELT-HLPLIKEVI-N---TK
Cladosiphon_okamuranus2_BDDF01000073.1                 -FDRCGELGLTVFSSPFELSAIDLL-EELNAP-CYKIASFELV-DLPLIRKAA-A---TG
Cronartium_ribicola_AWVX0102625.1                      -FDRCRELGMIGFSTPFDESAVNFL-EELDVP-LYKIASFENT-DIPLIQKVA-K---TG
CBA31353.1_Curvibacter                                 -FDRAVALGMIPFSTPFDDTAVDFL-ESLNVP-CYKIASFENT-DLPLIRRVA-A---TG
Hydra_vulgaris_ABRM01085349.1                          -FDRAVALGMIPFSTPFDDTAVDFL-ESLNVP-CYKIASFENT-DLPLIRRVA-A---TG
WP_018506507.1_pseudaminicAS_Thiobacillus              -FDRARELGIIPFSTPFDDTAVDFL-EALDVP-CYKIASFENT-DLPLIRRVA-A---TG
WP_009207339.1_pseudaminicAS_Sulfuricella              -FDRARELGIIAFSTPFDITAVDFL-EGLNVP-CYKIASFENT-DLPLIRRVA-A---TG
WP_067217189.1_pseudaminicAS_Marinomonas               -FEYAKSLGLVAFSSPFDASAVEFL-ESLNVP-LYKVASFELT-DIPLIQKIA-V---TG
WP_087462101.1_pseudaminicAS_Oleiphilus                -FSLARENGLIAFSSPFDETSVELL-ESLDVP-CYKIASFELN-HFPLLKALA-R---TG
Bankia_setacea_MRUH01000164.1                          -FQLAKDQGLIAFSSPFDETSVDFL-ESLQVP-CYKIASFEIN-HFPLLKKVA-A---TG
KIX22924.1_NeuB_PseudAS_Flavobacterium                 -YKAAEAEGLICFSSPFDKTAVDYL-ENLNTP-AYKIASFEIT-DIPLIEYVA-S---KM
XP_001618767.1_NANS_Nematostella                       ------------------------------------------------------------
WP_092376349.1_pseudaminicAS_Desulfomicrobium          -FDHARNLGITIFSTPFDETAVDLL-EDLGTP-AYKIASFEIV-DLPLIRYAA-S---TG
Condylostoma_magnum2_CVLX01030206.1                    -FEHARELGITVFSTPFDETAVDLL-ESLDTP-AYKIASFEIV-DLPLIRYVA-S---TG
AAD45660.1_NeuB_Aeromonas                              -FEKAKELGITIFSSPFDFTAVDLL-EDLDAP-AYKIASFELI-DLPLIKRVA-Q---TG
WP_094706949.1_pseudaminicAS_Hahella                   -FEHARKLGITIFSSPFDDTAVDLL-EDLGAP-AYKIASFEAI-DLPLIRYTA-S---TG
Aureococcus_anophagefferens_ACJI01001814.1             -HAYGKEIGIEWATSVWDVTSAREM-ADIPCD-YLKVPSACNN-HFDMLKVLRDE---YA
Emiliania_huxley_AHAL01004984.1                        -KH-CEEIGLGYSCSVWDVTSAKEI-VSLNPI-LIKVGSPSNQ-HWEMQKVLRDE---YG
Melampsora_pinitorqua_AUYS0100826.1                    -ARCCEKVGIEFMASVFDTSRIGWL-EDVGVK-RYKVASRSVR-DTALLAALA-K---TK
EEN59689.1_NANS_Branchiostoma                          NSKASTSLPQQWMRYGLELLPADFLADELDVP-FIKVASADAN-NLPYLKHTA-S---KG
Q9VG74.2_NANS_Drosophila                               -QAHCKELNVDFTASAMDERSLEFL-SALNVP-FIKIGSGDAN-NFPLLKKAA-N---LN
EFX62694.1_NANS_Daphnia                                -MAFASLINIHFTASAMDEISLDFL-VSLGVP-FIKMGSGDAN-NLILLEKAA-K---TK
XP_008190466.1_NANS_Tribolium                          -QSFAAEIGILFTASAMDSQSLRFL-ASLNVP-FIKIGSGDSN-NLLLIQEAA-A---MN
Q9NR45.2_NANS_HSA                                      -QRYAEEVGIFFTASGMDEMAVEFL-HELNVP-FFKVGSGDTN-NFPYLEKTA-K---KG
XP_783181.3_NANS_Strongylo                             -QAYSKEIGIYFTASGMDEKAVEFL-DSINVP-FFKVGSGDTN-NLPYIEKVA-K---MN
KMQ51676.1_Chitinispirillum                            -KRFCAQNEILFCASGWDEESIDFL-DSIDVP-FFKMASADLT-NLPLLEHTA-K---KD
OUX54928.1_Flammeovirgaceae_bacterium                  -KKFTDNLGLLFSASGWDEESIDFL-MEIDVP-FFKMASADLS-NFPLLEHTA-K---TG
OUV47462.1_Flavobacteriaceae_bacterium                 -FACANTHNVSFIASGWDEESVDFL-DHLGVS-FFKMASADLT-NFPLLEHTA-K---KN
OIO66895.1_Candidatus_Marinimicrobia                   -LSFANEMNVDFTASGWDEESIDFL-DTLGID-FFKMASADLT-NFPLLEHTA-K---KG
WP_027268045.1_Legionella                              -KTFCHNLRVDFFSTAFDFESADFL-EELDIP-YFKIASGDLR-NLPLIEYVA-R---FG
OGP61643.1_Deltaproteobacteria                         -KQYAKEIEIDFFATAFDFASADFL-AELDMP-AFKIASGDLN-NIPLLKYVA-K---IG
WP_022662582.1_Desulfovibrio                           -KEYASEIGIDFFATAFDLNSADFL-ASFDMP-FYKIASGDLT-NTPLLKHVA-K---IG
OGP71522.1_Deltaproteobacteria                         -QHYARELGITLLATAFDFPSVDFL-AKLEMP-AYKIASGDLT-NIPLLKYAA-Q---VG
Cladosiphon_okamuranus_BDDF01000009.1                  -DELCSELSIDWFASPWDEPSVDFL-MKYKTP-YLKIASAMVM-DRDFLKHCA-S---TG
Elaphomyces_granulatus_NPHW01007775.1                  -DKHCKEKGIQWFASCWDVDSVNFM-EKFDPP-VYKAASASLT-DIELLKKMK-S---TG
```

```
Thermobifida_fusca_NeuB_YXQ47U14                      -AKICEENGLQWFASPWDVPSVEFL-ESFDVV-AHKVASASIT-DHELLRALA-A---TG
Acytostellium_ellipticum_FLTD01002867.1               -GDHATMLGLDWFASPWDVPSVAFL-EDLNVV-AHKVASASLT-DTELLIALR-E---TG
Dictyostelium_deminutivum_FLTF01003146.1              -GDHATMLGLDWFASPWDVPSVAFL-EDLNVV-AHKVASASLT-DTELLIALR-E---TG
Lentinus_polychrous_JSYW01008743.1                    -AEMCRAAGVGYVSSVWDLESLEWI--DPYMD-FYKIGSGDLT-AWPLLRNFA-Q---RG
WP_035074769.1_Desulfovibrio                          -SALAKKEGALFMSSPFDEITVREL-NKF-VP-AFKVASCDVT-FYPLLKEIA-A---TG
SBT09427.1_Candidatus_Propionivibrio                  -ATIAGAAGTVFLSTPFSPEVVPWL-DEL-VP-AFKIASGDNN-YASLLAAVA-A---TG
WP_008313751.1_NeuB_Leptolyngbya                      -AATAAKAQVEFLSTPFAPEVVPWL-DDL-VP-AFKVASGDNN-YWALLEAIA-L---TG
WP_096701985.1_Magnetospirillum                       -AQVARDNAIMFMSSPFSLDAVDLL-APL-VP-AMKVASGDND-HVVLLDRIA-A---TG
Chromera_velia_ARZB01016018.1                         -AAIAADAGVLFLSTPFSLEAVAML-EPL-VP-AFKIASGDND-FAPLLAQTA-R---TG
ABS75841.1_SpsE_Bacillus_velezensis                   -LEYCKKKGVLFLSTVCDEGSADLL-YATSPS-AFKIASYELN-HLPLLKYVA-G---LK
Bacillus_subtilis_P39625.1_SpsE                       -LDYCREKQVIFLSTVCDEGSADLL-QSTSPS-AFKIASYEIN-HLPLLKYVA-R---LN
Prorocentrum_minimum2_JXLM01007734.1                  -AEHCQNVGIDFASTPFDAEAVSFL-DPL-MT-YFKIASADLT-NIPMLRLIA-N---TG
Phytophthora_lateralis_AMZP02003661.1                 -FARCTELGIEFMSTAFDEEALDFL-VALGIK-RIKVPSGEIT-NAPLLRHMA-S---KG
AAC43302.1_neuB_Escherichia                           -MEYAVSLNLDVFSTPFDEDSIDFL-ASLKQK-IWKIPSGELL-NLPYLEKIA-KLPIPD
ABD95628.1_NeuB_Streptococcus                         -RDYAISKGVETFSTPFDEESLEFL-ISTDMP-IYKIPSGEIT-NLPYLEKIG-K---QQ
Tuber-aestivum_CZPR01000680.1                         -QAHAHRSGIEFISTAFDTDSLKFL-CEMNLP-FFKVPSGELT-NGPLLWQFA-H---TG
Methanococcoides_burtonii_NeuB_NnaB_WP_011499634.1    -MKHAEQKNIMFLSTPFDERSVDLL-VELGVP-LIKISSGEIT-NHPLLKYIS-R---KG
AEG18372.1_Methanobacterium                           -AKYADEKDIIFLSTPFDFESVDLL-DEIGVP-AFKLGSGELT-NFPLLEHVA-S---KG
WP_045444639.1_Neub_Tepidicaulis                      -ITRCAERGIEFLSTPFDFPSLDLL-ANRLELARLKIGSGELT-NAPLLLAIA-R---TR
WP_029638846.1_NeuB_alphaproteobacterium              -IQRCAEKGVQFLSTPFDLGSLDLL-ANELNVATLKVGSGELT-NAPLLHACA-T---AG
Prorocentrum_minimum1_JXLM01000037.1                  -IQRCAEKGVQFLSTPFDLGSLDLL-ANELNVATLKVGSGELTSNAPLLHACA-T---AG
Paramecium_biaurelia_JPFL01000669.1                   -IQRCQEKGIHFLSTGFDQDSIDFL-NDFEIP-FYKIPSGEIT-NKPYLQHIA-R---KG
ADL05453.1_Clostridium                                -YHYCALRHIKFLSSPFDEESILFL-DRLGVD-PIKIPSGEIT-NYGYLKKTA-S---LK
EFM37961.1_Campylobacter                              -ILHAKKCNIAFLSTPFDLESVDLL-NELGLK-IFKIPSGEIT-NLPYLKKIA-K---LN
EIJ34212.1_Thiothrix                                  -FEYCDRKNIEFMSTPFDLHSIQFL-AELGVK-RFKIPSGEIT-NYPYLSLIG-S---FN
Condylostoma_magnum_CVLX01013231.1                    -IDECKKHHIRFLSTAFDEQSLNML-LELNCLDFIKIPSGEVT-NLPFLRVIA-S---KN

Treponema_pallidum_SpsE_NP_219001.1                   LPLILSSGVCLLKEIEGALAQCRQ-----------------------YTKQGSSHALLHC
Reticulomyxa_filosa_ASPP01005516.1                    KPVILSTGMQTIETIRESVAILDA-----------------------AG--V-DYALLEC
Neisseria_meningitidis_NeuB_NnaB_1XUU                 KPIILSTGMNSIESIKKSVEIIRE-----------------------AG--V-PYALLHC
Neisseria_meningitidis_NeuB_2WQP_A                    KPIILSTGXNSIESIKKSVEIIRE-----------------------AG--V-PYALLHC
Acanthamoeba_palestinensis_CDFD01053174.1             KPLILSTGTATIEEIEQTVEFLHQ-----------------------HS--CNNFILLYC
Cladosiphon_okamuranus2_BDDF01000073.1                KPLIMSTGMASVSDIHDAVQAAKA-----------------------EG--NDSIILLKC
Cronartium_ribicola_AWVX0102625.1                     KPMIISTGMASVAELDETVRAIRE-----------------------LG--CENFVLLKC
CBA31353.1_Curvibacter                                KPLIISTGMATVAELDETVRAARD-----------------------AG--CKDLILLKC
Hydra_vulgaris_ABRM01085349.1                         KPLIISTGMATVAELDETVRAARD-----------------------AG--CKDLILLKC
WP_018506507.1_pseudaminicAS_Thiobacillus             KPLIISTGMATVAELDETVRAARE-----------------------AG--CMDLILLKC
WP_009207339.1_pseudaminicAS_Sulfuricella             KPLIISTGMATVAELDETVRAARE-----------------------AG--CNDLILLKC
WP_067217189.1_pseudaminicAS_Marinomonas              KPTIMSTGMASLEEIEEAVAAFRH-----------------------IS--DAELVLLKC
WP_087462101.1_pseudaminicAS_Oleiphilus               KPIIMSTGMATLEDIDASVEYLYQ-----------------------QG--CSELVLLKC
Bankia_setacea_MRUH01000164.1                         KPVIMSTGMSSSEEIEEAVAHLRE-----------------------NG--CTDIALLKC
KIX22924.1_NeuB_PseudAS_Flavobacterium                KPIIISTGIAELIDIELAVEACKR-----------------------VG--NHNIALLKC
XP_001618767.1_NANS_Nematostella                      ---------------------------------------------------NHNIALLKC
WP_092376349.1_pseudaminicAS_Desulfomicrobium         KPMIMSTGMASEEEIDEAVAAAHE-----------------------AG--CKDLILLHC
Condylostoma_magnum2_CVLX01030206.1                   KPIILSTGMATEDEIDEALTMAHE-----------------------AG--CKDLILLHC
AAD45660.1_NeuB_Aeromonas                             KPMIMSTGMANAEEIAEAIATAQS-----------------------NG--CQELVVLHC
WP_094706949.1_pseudaminicAS_Hahella                  KPLIISTGMATEDEIQEAAAAAAD-----------------------AG--CQELVLLHC
Aureococcus_anophagefferens_ACJI01001814.1            GDVHVSTGMTTLAEIEDVVAFFEA-----------------------TGQ-AKRLVVYNC
Emiliania_huxley_AHAL01004984.1                       GDIHISTGMTTKEEVE----------------------------------------KINC
Melampsora_pinitorqua_AUYS0100826.1                   KPLIVSLGMWNEPSFPSI-------------------------------PSAAHVDFLYC
EEN59689.1_NANS_Branchiostoma                         KPMILSSGMQSMGIMERAVGTVTE-----------------------NMPGEKRLVVMQC
Q9VG74.2_NANS_Drosophila                              LPLVISTGMQTMQTVERIVQTMRE-------------------------SGKEDYALMHC
EFX62694.1_NANS_Daphnia                               LPIIYSTGMQDMQTVKTAMERMNA-------------------------C-NPRVCILQC
XP_008190466.1_NANS_Tribolium                         VPLVISTGMQDLGGVRATYECVAQ-------------------------Y-HKKFALLHC
Q9NR45.2_NANS_HSA                                     RPMVISSGMQSMDTMKQVYQIVKP-------------------------L-NPNFCFLQC
XP_783181.3_NANS_Strongylo                            RAMVVSSGMQSMETMTRVYETCKK-------------------------Y-NDKFCILQC
KMQ51676.1_Chitinispirillum                           KPVILSTGMACLEDVRNAYVTVGK-------------------------HN-DKIAILQC
OUX54928.1_Flammeovirgaceae_bacterium                 KPIILSTGMADMELVKKAVDLVSK-------------------------FN-SNIAILQC
OUV47462.1_Flavobacteriaceae_bacterium                KPIILSTGMSAIDTVKKAYDLINN-------------------------YN-NQIAILQC
OIO66895.1_Candidatus_Marinimicrobia                  RPMILSTGMADLEMVKQAYQLVRS-------------------------FN-LQIALLQC
WP_027268045.1_Legionella                             KPIFISTGGADFEDVQRVYDRIMP-------------------------IN-SNICIMQC
OGP61643.1_Deltaproteobacteria                        KPMIVSTGGATMENVKRAYDAIMP-------------------------LN-ENLCILQC
WP_022662582.1_Desulfovibrio                          KPVILSTGGGTMADVRRAYEAIMP-------------------------IN-DQLAILQC
OGP71522.1_Deltaproteobacteria                        KPLIVSTGGGAMEDVQRAYDAVMP-------------------------IN-PQLCLLQC
Cladosiphon_okamuranus_BDDF01000009.1                 RPLLVSTGMCDLPMIRRAVETIEA-------------------------ANGEIACLYHC
Elaphomyces_granulatus_NPHW01007775.1                 KPLIVSTGMSTMQEIEESIETAGR----------------------------DNLLIAHS
Thermobifida_fusca_NeuB_YXQ47U14                      KPIILSTGMSTIEEIDAAVEIFDR----------------------------SKLILMHS
Acytostellium_ellipticum_FLTD01002867.1               KPVILSTGMSTIEQIDRALDTLGT----------------------------DRVVLMHA
Dictyostelium_deminutivum_FLTF01003146.1              KPVILSTGMSTIEQIGRALDTLGT----------------------------DRVVLMHA
Lentinus_polychrous_JSYW01008743.1                    KPILLSTGLATLDEILQTVAFIQSVDDR--------------------YRQPEWLCLMQC
WP_035074769.1_Desulfovibrio                          KPIIMSTGAASEEEVLNSCKYIESCWEK-------------------AGIKSPGLALLHC
SBT09427.1_Candidatus_Propionivibrio                  KPVLLSTGMAGMTGIREACRIIETAAQK-------------------SGHS-AHIALLHC
WP_008313751.1_NeuB_Leptolyngbya                      KPVLLSTGMSYLADVKQAIAVIETVWQN-------------------HGTQ-STLIPLHC
WP_096701985.1_Magnetospirillum                       LPVILSTGMTDLDGAAFSAGTLCRAWAK-------------------AGIADPGLVLLHC
Chromera_velia_ARZB01016018.1                         KPLLISTGMSRPEDIAQAVTRVRMAWAT-------------------AEFRDPGLVLLHC
ABS75841.1_SpsE_Bacillus_velezensis                   TPIIFSTAGADISDIQEAYQTITG-------------------------EGNTEIAIMHC
Bacillus_subtilis_P39625.1_SpsE                       RPMIFSTAGAEISDVHEAWRTIRA-------------------------EGNNQIAIMHC
Prorocentrum_minimum2_JXLM01007734.1                  KPVLLSTGASSLAEIDIAVNELIS-------------------------HGCENLTLLQC
Phytophthora_lateralis_AMZP02003661.1                 LPLIVSTGMAELDEVVAAIGIIRAARES----------------HGFAEPLGDIVTILHC
AAC43302.1_neuB_Escherichia                           KKIIISTGMATIDEIKQSVSIFIN-----------------------NKVPVGNITILHC
ABD95628.1_NeuB_Streptococcus                         KKVILSTGMAVMEEIHQAVNILRQ-----------------------NG--TTDISILHC
Tuber-aestivum_CZPR01000680.1                         KPLVLSTGMATLSEVEQGLAIVAHALATDREPANLDEVWKNWSQPQARTRLKGHVTLLHC
Methanococcoides_burtonii_NeuB_NnaB_WP_011499634.1    IPIILSTGMSTLEEVAEAVSVIKD-------------------------AGCEDLTLLHC
AEG18372.1_Methanobacterium                           RPVILSTGMATMDEIKEAVKLFED-------------------------K-TNDLILMHC
WP_045444639.1_Neub_Tepidicaulis                      RPVILSTGMARLEEIEAALGVLAFGYGEGGVPSKSAFQAAYKSGAGQR-ALRANVTLLHC
WP_029638846.1_NeuB_alphaproteobacterium              RDLILSTGMATLDEVQTMLGVVAHGFLGESSPSEKAFKEAFESGAGKA-VLKSRVKLLHC
Prorocentrum_minimum1_JXLM01000037.1                  RNLILSTGMATLDEVQTMLGVVAHGYLGPDRPSEKAFKEAFESDAGKASVLKSRVKLLHC
Paramecium_biaurelia_JPFL01000669.1                   KDIILSTGMADLKEVKEAIEVIEA-----------------------EGITRNRITVLHC
ADL05453.1_Clostridium                                KQVLLSTGMSTEAEIGEALEILEE-----------------------SGK---EIILLHC
```

```
EFM37961.1_Campylobacter                              KKIILSTGMANLGEIEEALNALCK-----------------------NGTKRQNITLLHC
EIJ34212.1_Thiothrix                                  KEIILSTGMANLGEVEAAIQVLLE-----------------------AGTEQHKITVLHA
Condylostoma_magnum_CVLX01013231.1                    KPIILSTGMCDEDEVANALAVLTQ-----------------------AGVKKEMITILHC

Treponema_pallidum_SpsE_NP_219001.1                   ITAYPAPETEYNLALLPALATIF---NINVGVSDHSVD---PLLVPLLARAHGAC-IVEK
Reticulomyxa_filosa_ASPP01005516.1                    ------------------------------------------------------------
Neisseria_meningitidis_NeuB_NnaB_1XUU                 TNIYPTPYEDVRLGGMNDLSEAFP--DAIIGLSDHTLD---N-YACLGAVALGGS-ILER
Neisseria_meningitidis_NeuB_2WQP_A                    TNIYPTPYEDVRLGGXNDLSEAFP--DAIIGLSDHTLD---N-YACLGAVALGGS-ILER
Acanthamoeba_palestinensis_CDFD01053174.1             VSSYPAPLEDYNLSTMKEIQNRFG--CL-VGLSDHSMG---I-IAPIVATTMGAC-FIEK
Cladosiphon_okamuranus2_BDDF01000073.1                TSSYPATAADANIATIPHMRETF---GVQVGLSDHTLG---I-GVPCAAAALGAT-VIEK
Cronartium_ribicola_AWVX0102625.1                     TSTYPSTPAFTNLNTIPHLRQLF---NCQVGLSDHTGG---I-GAAVAAVALGAT-VIEK
CBA31353.1_Curvibacter                                TSTYPATASNTNILTIPHLRELF---GCEVGLSDHTMG---V-GVSVASVALGAT-VIEK
Hydra_vulgaris_ABRM01085349.1                         TSTYPATASNTNILTIPHLRELF---GCEVGLSDHTMG---V-GVSVASVALGAT-VIEK
WP_018506507.1_pseudaminicAS_Thiobacillus             TSTYPATAENTNILTIPHLRELF---GCEVGLSDHTMG---V-GVSVASVALGAS-VIEK
WP_009207339.1_pseudaminicAS_Sulfuricella             TSTYPATAENTNILTIPHLRELF---GCEVGLSDHTMG---V-GVSVASVALGAT-VIEK
WP_067217189.1_pseudaminicAS_Marinomonas             TSTYPANMADSNLATIPDLAKRF---DCRVGLSDHTRG---I-GASVASIAFGAT-VIEK
WP_087462101.1_pseudaminicAS_Oleiphilus               TSSYPAPIKDANLRTIEDMKRRF---QIPVGLSDHCQG---I-DVALASVALGAN-VIER
Bankia_setacea_MRUH01000164.1                         TSAYPASVADANLATIADMKIRF---GLPIGLSDHSLG---I-GVAIVAAAMGAE-IIER
KIX22924.1_NeuB_PseudAS_Flavobacterium                TSSYPAPIEEANMCMVKDLAERFN---VISGLSDHTMG---A-TVPIVATALGAK-IIEK
XP_001618767.1_NANS_Nematostella                      TSSYPAPIEEANMCMVKDLAERFN---VISGLSDHTMG---A-TVPIVATALGAK-IIEK
WP_092376349.1_pseudaminicAS_Desulfomicrobium         ISSYPAPIEQANLRQIPELARRFG---VISGLSDHTMG---T-TAAVTAVALGAC-VIEK
Condylostoma_magnum2_CVLX01030206.1                   ISSYPAPMDQANLRQIPELSKRF---DILTGLSDHTMG---T-SAAVTATALGAC-IIEK
AAD45660.1_NeuB_Aeromonas                             VSGYPAPADQYNLRTIADMAERFG---VLSGLSDHTID---N-ATAVAAVALGAC-LIEK
WP_094706949.1_pseudaminicAS_Hahella                  VSGYPAPAEDYNLRTLTDMMDRYN---CLVGLSDHTID---N-AAAIASVALGAC-LIEK
Aureococcus_anophagefferens_ACJI01001814.1            TSGYPVPFPSVCMLELVRLYERYGDRVKELAFSGHHLG---I-AIDVAAYTLGAK-WIER
Emiliania_huxley_AHAL01004984.1                       TSGYPVPFEDVSLHATACEQV--PFEDVAIGFSGHHLG---I-AVDVAAYAMGAQ-WNER
Melampsora_pinitorqua_AUYS0100826.1                   ISKYPTPLSDLRLGKVDF--------KKHSGFSDHSEG---I-TAACGAFVLGAR-ILEK
EEN59689.1_NANS_Branchiostoma                         TSTYPLPPADVHLRCINLFKEKFP--HAIIGYSGHESG---I-AISLAAVSLGAK-VVER
Q9VG74.2_NANS_Drosophila                              VSSYPTDPKDCSLQLISVLRTRFP--NVAIGYSGHELG---V-IISQAAVLLGAR-IVER
EFX62694.1_NANS_Daphnia                               TSSYPTPEDQVHLRVMQSYRDHFP--GSPVGYSGHELG---L-SITLAAAALGAH-VIER
XP_008190466.1_NANS_Tribolium                         VSAYPTPLQEINLNVIKLYETEFP--DVVIGYSGHELG---I-EASVAAVAVGAK-IIER
Q9NR45.2_NANS_HSA                                     TSAYPLQPEDVNLRVISEYQKLFP--DIPIGYSGHETG---I-AISVAAVALGAK-VLER
XP_783181.3_NANS_Strongylo                            TSAYPLHPEDVHLRIIQVFQEAFP--DIPIGYSGHEGG---L-SITLASVALGAK-VVER
KMQ51676.1_Chitinispirillum                           TSTYPSKFSEINLNVLHTFKKEFP--DAVIGFSGHELG---I-AIPPVAVGLGAC-IIEK
OUX54928.1_Flammeovirgaceae_bacterium                 TSTYPAQFNEINLNVIRTYLDIFP--KYVIGYSGHELG---I-AISGVAVALGAK-IIER
OUV47462.1_Flavobacteriaceae_bacterium                TSTYPTSFEEIHLNVIRSYKRLFP--GVVIGYSGHELG---I-AVPSAAVAIGAK-IIER
OIO66895.1_Candidatus_Marinimicrobia                  TSTYPSRFNEINLRVLETFRMAFP--EAVIGYSGHELG---I-AISEVAVALGAK-IVER
WP_027268045.1_Legionella                             TSGYPCEYNELNLRVIETYRKEFP--DVVVGFSSHDSG---I-ASPVAGYVLGAR-VIEK
OGP61643.1_Deltaproteobacteria                        TAAYPAEHDILNLNVISTYRKAFP--KNVIGLSDHENG---I-AMAIAAFVLGAR-IFEK
WP_022662582.1_Desulfovibrio                          TAAYPCQPEDMNLRVIETYMREFP--DVVIGLSDHQNG---I-AMAMVAYTMGAR-IFEK
OGP71522.1_Deltaproteobacteria                        TASYPVDPGDMNLRVISTYRRLFP--DLIIGLSDHQNG---I-AMAVAAYVLGAR-VVEK
Cladosiphon_okamuranus_BDDF01000009.1                 TSTYPTLDEEINLMGIQQLQQAFP--ALRIGFSGHEQG---I-LPSVCAAALGAK-SVER
Elaphomyces_granulatus_NPHW01007775.1                 TSTYPCKAEELNLKMVQTLKEKYH--GNPIGYSGHEVG---L-SPTWAAVAMGAS-FVER
Thermobifida_fusca_NeuB_YXQ47U14                      TSTYPMPPEEANLRTIITLRERY---GVPVGYSGHERG---L-QISLAAVTLGAV-AVER
Acytostellium_ellipticum_FLTD01002867.1               TSTYPLEPEEANLRVIATLRDRYP--GVPVGYSGHERG---L-QISLAAVAIGAV-AVER
Dictyostelium_deminutivum_FLTF01003146.1              TSTYPLEPEEANLRVIATLRDRYP--GVPVGYSGHERG---L-QISLAAVAIGAV-AVER
Lentinus_polychrous_JSYW01008743.1                    TSMYPIPDSDANLCVMDTLRAM-T--GLSIGYSDHTTG---M-MALRAATAMGAE-ALEF
WP_035074769.1_Desulfovibrio                          VSSYPTPVNEANLKAVSTLA-KT---GYVAGYSDHTLG---I-DAAVLSIGLGAR-IIEK
SBT09427.1_Candidatus_Propionivibrio                  VSAYPTPPAQANLRAISTLADEI---GGVVGYSDHTLG---I-EAAVLSVALGAR-IIEK
WP_008313751.1_NeuB_Leptolyngbya                      VSAYPTPLEQANLGAIQTLAQET---GQIVGYSDHTLG---I-EAAIFSVLLGAR-VIEK
WP_096701985.1_Magnetospirillum                       VSSYPTPADQANLRAIRTLA-GL---GHVVGYSDHTLG---I-EAAVLSVALGAR-VIEK
Chromera_velia_ARZB01016018.1                         VSAYPTPPSAANLRAILALP-TY---GAVAGYSDHTLG---I-EAAVLSVALGAR-VIEK
ABS75841.1_SpsE_Bacillus_velezensis                   VAKYPAPPEYSNLSVIPMLAAAFP--DAVIGFSDHSAH--PY-DVPCAAVRLGAK-LIEK
Bacillus_subtilis_P39625.1_SpsE                       VAKYPAPPEYSNLSVIPMLAAAFP--EAVIGFSDHSEH--PT-EAPCAAVRLGAK-LIEK
Prorocentrum_minimum2_JXLM01007734.1                  TLNYPCPYENANLNMIDGLKRAFP--QFEIGYSDHTHPDPDM-MILTAAWLKGAS-ILEK
Phytophthora_lateralis_AMZP02003661.1                 TSNYPAESADVNLRAMNTMARTT---GLPVGYSDHTLG---L-AVSTGAVALGAC-VIEK
AAC43302.1_neuB_Escherichia                           NTEYPTPFEDVNLNAINDLKKHFP--KNNIGFSDHSSG---F-YAAIAAVPYGIT-FIEK
ABD95628.1_NeuB_Streptococcus                         TTEYPTPYPSLNLNVIHTLKDEFK--DLTIGYSDHSIG---S-EVPIAAAAMGAE-VIEK
Tuber-aestivum_CZPR01000680.1                         TSQYPTPWGEVNLRAMDTLRA-F---DLAVGYSDHTEG---G-LISVAAVARGAK-VIEK
Methanococcoides_burtonii_NeuB_NnaB_WP_011499634.1    TSSYPARVEDCNLLTMETMADVF---DVQVGYSDHTSG---I-CVPLAAAAMGAC-VIEK
AEG18372.1_Methanobacterium                           VTSYPAKIDDINLKVIETLRSTF---KLPVGFSDHTLG---I-EMPIAAVALGSC-VIEK
WP_045444639.1_Neub_Tepidicaulis                      TSAYPAPDGEINLKAMDTLRQKF---GLPVGFSDHSVG---I-DIPIAAAALGAA-MIEK
WP_029638846.1_NeuB_alphaproteobacterium              TSSYPTPDGDVNLRAIETLRAKF---DLPVGFSDHSDG---I-VHAIASVAMGAC-IIEK
Prorocentrum_minimum1_JXLM01000037.1                  TSSYPTPDGDVNLRAIETLRAKF---DLPVGFSDHSEG---I-VHAIASVAMGACSIIEK
Paramecium_biaurelia_JPFL01000669.1                   NTEYPTPMQDVNLLAMNQMAEKL---GVQIGYSDHTLG---I-EVPIAAVALGAK-VIEK
ADL05453.1_Clostridium                                SSAYPTMMEDVNLNAMATLKDRF---HKQVGYSDHTPG---I-EVPIAAAALGAC-VIEK
EFM37961.1_Campylobacter                              TTEYPAPFDEVNLKAMQSLKDAF---KLDVGYSDHTQG---I-HISLAAVALGAC-VIEK
EIJ34212.1_Thiothrix                                  TTDYPTQMIDVNLRAMQTMAQAFK---FPVGYSDHTPG---I-EVPTAAVALGAS-VIEK
Condylostoma_magnum_CVLX01013231.1                    TTDYPTEMQDVNLKAMQTMGKKF---GTKIGYSDHTIG---I-EVPIVAAAMGAS-VIEK

Treponema_pallidum_SpsE_NP_219001.1                   HICLSRTDAG-LDDSIALDPADFRTMTAALNSCARRSPSQIISFLHERGYAPHVVRAVI-
Reticulomyxa_filosa_ASPP01005516.1                    ------------------------------------------------------------
Neisseria_meningitidis_NeuB_NnaB_1XUU                 HFTDRMDRPG-PDIVCSMNPDTFKELKQGAHAL----------------------KLAR-
Neisseria_meningitidis_NeuB_2WQP_A                    HFTDRXDRPG-PDIVCSXNPDTFKELKQGAHAL----------------------KLAR-
Acanthamoeba_palestinensis_CDFD01053174.1             HFTLDRNDGG-SDSSFSLEPAELKLMCDSVRSA----------------------YACI-
Cladosiphon_okamuranus2_BDDF01000073.1                HFTLRREDGG-VDSTFSLEPWEFAALVEESERA----------------------WASV-
Cronartium_ribicola_AWVX0102625.1                     HFTLRRADGG-VDSTFSMEPEEMEELVLETERA----------------------WQAL-
CBA31353.1_Curvibacter                                HFTLSRAEGG-VDSAFSMEPAEMAQLVLETERA----------------------WQSL-
Hydra_vulgaris_ABRM01085349.1                         HFTLSRAEGG-VDSAFSMEPAEMAQLVLETERA----------------------WQSL-
WP_018506507.1_pseudaminicAS_Thiobacillus             HFTLSRADGG-VDSAFSMEPAEMTQLVLEAERA----------------------WQAL-
WP_009207339.1_pseudaminicAS_Sulfuricella             HFTLNRADGG-VDSTFSMEPSEMTQLVVETERA----------------------WQAL-
WP_067217189.1_pseudaminicAS_Marinomonas             HFVLDRSAGG-VDAEFSMEPGEMAALVEACHQA----------------------YTAI-
WP_087462101.1_pseudaminicAS_Oleiphilus               HIVLDRNTEA-VDGAFSSTPEEFSRLVGQTKNI----------------------SAAL-
Bankia_setacea_MRUH01000164.1                         HFVLDRDSNA-VDAAFSSTPAELAQLVKEASQI----------------------KSAI-
KIX22924.1_NeuB_PseudAS_Flavobacterium                HFILDRSIGG-PDASFSMNEEEFTAMVKAVREA----------------------EKAI-
XP_001618767.1_NANS_Nematostella                      HFILDRSIGG-PDASFSMNEEEFTAMVKAVREA----------------------EKAI-
WP_092376349.1_pseudaminicAS_Desulfomicrobium         HFTLSRQDKG-PDCEFSLEPHEMERLCVEAKDA----------------------WRAL-
Condylostoma_magnum2_CVLX01030206.1                   HFTLNREDKG-PDSEFSLEPDELKRLCIDTKEA----------------------WMAL-
AAD45660.1_NeuB_Aeromonas                             HVTMDRNGGG-ADDSFSLEPHELAALCKDAKTA----------------------WSAL-
```

```
WP_094706949.1_pseudaminicAS_Hahella                      HVTLDRKGGG-PDDSFSLEPDDLKMLCESSRTA----------------------WQAL-
Aureococcus_anophagefferens_ACJI01001814.1                HFTKDRTWKG-TDHAASLEAGGFGKLVRDLEAT----------------------HQA--
Emiliania_huxley_AHAL01004984.1                           HFTKDRTWKG-TDHAASLEPAGLTKLCRDLQAT----------------------WKC--
Melampsora_pinitorqua_AUYS0100826.1                       HLTLDKTSYG-PDHSCSMTPDDLAAIDRFRRDW----------------------EECA-
EEN59689.1_NANS_Branchiostoma                             HVTLDKSWKG-SDHAASLTFDELKDLCDNIRIV----------------------ETA--
Q9VG74.2_NANS_Drosophila                                  HFTLDKSQKG-SDHRCSLEPQELKALTTAITNF----------------------KLSSV
EFX62694.1_NANS_Daphnia                                   HVTLDKSWKG-NDHACSLDPLELKSLVEGIRAI----------------------EKA--
XP_008190466.1_NANS_Tribolium                             HVTLDKTQKG-SDHQCSLEPQELKLLIEKIREL----------------------DIT--
Q9NR45.2_NANS_HSA                                         HITLDKTWKG-SDHSASLEPGELAELVRSVRLV----------------------ERA--
XP_783181.3_NANS_Strongylo                                HITMDKSWKG-NDHEASLDPEELAELVREIRIV----------------------ERS--
KMQ51676.1_Chitinispirillum                               HFTLDRTMKG-GDHAASLEPQGFAKMVRDIHHV----------------------TEA--
OUX54928.1_Flammeovirgaceae_bacterium                     HFTLDRTMKG-GDHAASLEPHGFSKMVRDIRHI----------------------EGA--
OUV47462.1_Flavobacteriaceae_bacterium                    HFTLDRTMKG-GDHAASLEPLGFKKMVRDIRNI----------------------EKA--
OIO66895.1_Candidatus_Marinimicrobia                      HFTLDRTMKG-GDHAASLEPGGLQKLIRDIRHI----------------------EDA--
WP_027268045.1_Legionella                                 HFTLNRTWKG-TDHAFSLGPQGFRKMVRDIRRV----------------------EQA--
OGP61643.1_Deltaproteobacteria                            HFTLNHTWKG-TDHAFSLEPVGFRKLVRDLKRL----------------------PKA--
WP_022662582.1_Desulfovibrio                              HFTLDRTWKG-TDHAFSLTSKGLSNLVRDLHRA----------------------RTS--
OGP71522.1_Deltaproteobacteria                            HFTLNRAMKG-TDHAFSLEPAGLRKLVRDLRRA----------------------RAA--
Cladosiphon_okamuranus_BDDF01000009.1                     HVTLDRSDWG-SDQKASLEMSEVAELVSQIRRV----------------------EVV--
Elaphomyces_granulatus_NPHW01007775.1                     HITLDRAMWG-TDQAASVEIMGLYRLVSNIRDI----------------------QRA--
Thermobifida_fusca_NeuB_YXQ47U14                          HITLDRTMWG-SDHAASLEPAGLQHLVRDIRVI----------------------EQA--
Acytostellium_ellipticum_FLTD01002867.1                   HITLDRTMWG-SDHAASLEPTGLEHLVRDIRVI----------------------ERA--
Dictyostelium_deminutivum_FLTF01003146.1                  HITLDRTMWG-SD-----------------------------------------------
Lentinus_polychrous_JSYW01008743.1                        HFTLSREGQTFRDHLVSLTADEVLALKDDIAQI----------------------IALR-
WP_035074769.1_Desulfovibrio                              HFTVDKNYSEFRDHQLSADPNEFKLMVEKIRSA----------------------EALL-
SBT09427.1_Candidatus_Propionivibrio                      HFTLSKTQSEFRDHALSADPAEMTELVRRVRLA----------------------QELL-
WP_008313751.1_NeuB_Leptolyngbya                          HFTLSKTQSDFRDHALSADPNEMAELVQQVQRV----------------------QAIL-
WP_096701985.1_Magnetospirillum                           HFTLSKTQSEFRDHQLSAEPHELKALVERVAEA----------------------NALL-
Chromera_velia_ARZB01016018.1                             HFTLSKTQSDFRDHQLSAEPSELAELVERVREA----------------------ETLL-
ABS75841.1_SpsE_Bacillus_velezensis                       HFTIDKNLPG-ADHSFALNPDELKEMVAAIRSV----------------------EAELR
Bacillus_subtilis_P39625.1_SpsE                           HFTIDKNLPG-ADHSFALNPDELKEMVDGIRKT----------------------EAELK
Prorocentrum_minimum2_JXLM01007734.1                      HFTHDKSLPG-NDHYHAMDVDDLKTFRANVALL----------------------STAL-
Phytophthora_lateralis_AMZP02003661.1                     HFTLDCELPG-PDHKASLEPDQLVALVRQIRDV----------------------EVAL-
AAC43302.1_neuB_Escherichia                               HFTLDKSMSG-PDHLASIEPDELKHLCIGVRCV----------------------EKSL-
ABD95628.1_NeuB_Streptococcus                             HFTLDTNMEG-PDHKASATPDILAALVKGVRIV----------------------EQAL-
Tuber-aestivum_CZPR01000680.1                             HFTLDRSMPG-PDHKASLEPDELKAMISQIRVL----------------------ELAL-
Methanococcoides_burtonii_NeuB_NnaB_WP_011499634.1        HFTLDKNLTG-PDHMASLEPTELEEMVRGIRLV----------------------EKAR-
AEG18372.1_Methanobacterium                               HFTLNKNLDG-PDHKASLEPADFKKMVLAIRNV----------------------EKGM-
WP_045444639.1_Neub_Tepidicaulis                          HFTLDKTLPG-PDHKASIEPDGLRALVSGVRRV----------------------SAAL-
WP_029638846.1_NeuB_alphaproteobacterium                  HYTLDKDLPG-PDHKASATAEELSDLVDGIRRV----------------------SAGL-
Prorocentrum_minimum1_JXLM01000037.1                      HYTLDKDLPG-PDHKASATPEELSDLVDGIRRV----------------------SAGL-
Paramecium_biaurelia_JPFL01000669.1                       HFTLDRNLPG-PDHAASLEPAELKEMVKTIRNI----------------------ELAI-
ADL05453.1_Clostridium                                    HMTLDKTMPG-PDHKASLEPDEFKHMADSIRNI----------------------EQAL-
EFM37961.1_Campylobacter                                  HFTLDKNMSG-PDHKASLEPHELKTLCTQIRQI----------------------QKAM-
EIJ34212.1_Thiothrix                                      HFTLDRNLPG-PDHKASLEPDELKAMVVAIRNI----------------------EIAL-
Condylostoma_magnum_CVLX01013231.1                        HFTLDRGLAG-PDHKVSLIPDELSQMVKAIRNI----------------------ELAL-

Treponema_pallidum_SpsE_NP_219001.1                       ----------------------GSGEKVLAPSERAHYQ--------------KSN-RSLH
Reticulomyxa_filosa_ASPP01005516.1                        ------------------------------------------------------------
Neisseria_meningitidis_NeuB_NnaB_1XUU                     ----------------------GGKKDTIIAGEKPTKD--------------FAF-ASVV
Neisseria_meningitidis_NeuB_2WQP_A                        ----------------------GGKKDTIIAGEKPTKD--------------FAF-ASVV
Acanthamoeba_palestinensis_CDFD01053174.1                 ----------------------GSPTFGYKDCEKR-SP--------------IFK-RHYY
Cladosiphon_okamuranus2_BDDF01000073.1                    ----------------------GRVVYGGAAVEQ-KSL--------------KFR-RSLY
Cronartium_ribicola_AWVX0102625.1                         ----------------------G-------------------------------------
CBA31353.1_Curvibacter                                    ----------------------GDVSYGPTDVEKK-SL--------------QFR-RSLY
Hydra_vulgaris_ABRM01085349.1                             ----------------------GDVSYGPTDVEKK-SL--------------QFR-RSLY
WP_018506507.1_pseudaminicAS_Thiobacillus                 ----------------------GRVSYGPTQAEK-KSL--------------QYR-RSLY
WP_009207339.1_pseudaminicAS_Sulfuricella                 ----------------------GQVSYGATEAEV-KSI--------------QFR-RSLY
WP_067217189.1_pseudaminicAS_Marinomonas                  ----------------------GSIMYGGSDAEQ-KAK--------------KYR-RSIY
WP_087462101.1_pseudaminicAS_Oleiphilus                   ----------------------GKVQYGPSESEL-DSL--------------KYR-RSIY
Bankia_setacea_MRUH01000164.1                             ----------------------GS------------------------------------
KIX22924.1_NeuB_PseudAS_Flavobacterium                    ----------------------GNVDYTLTEKQA-KGK--------------DFS-RSLY
XP_001618767.1_NANS_Nematostella                          ----------------------GNVDYTLTEKQA-KGK--------------DFS-RSLY
WP_092376349.1_pseudaminicAS_Desulfomicrobium             ----------------------GKAGYTREEAE-EKSK--------------IFR-RSVY
Condylostoma_magnum2_CVLX01030206.1                       ----------------------GQAGFERQKAEKD-NK--------------IFR-RSIY
AAD45660.1_NeuB_Aeromonas                                 ----------------------GSVNYTRTEAEKG-NV--------------KFR-RSLY
WP_094706949.1_pseudaminicAS_Hahella                      ----------------------GSVDYSRKASEVG-NA--------------KFR-RSLY
Aureococcus_anophagefferens_ACJI01001814.1                --------------------L-TLKASEILPIEDEQRD--------------KL------
Emiliania_huxley_AHAL01004984.1                           --------------------M-TPKASEILPIEKEQRA--------------KL------
Melampsora_pinitorqua_AUYS0100826.1                       ----------------------G-------------------------------------
EEN59689.1_NANS_Branchiostoma                             --------------------L-GKPIKEPRPTDIFTADLTVIKCSMIFTNEEKTT-KWTC
Q9VG74.2_NANS_Drosophila                                  PMPPQEIVKKLNGDEELEAALQHVESKTILPCELPCRN--------------KLG-KSIV
EFX62694.1_NANS_Daphnia                                   --------------------L-GSAVKSKQPSEEPCHQ--------------KLG-KSVV
XP_008190466.1_NANS_Tribolium                             --------------------L-GKPVKAFQASERPCYA--------------KLG-KSLV
Q9NR45.2_NANS_HSA                                         --------------------L-GSPTKQLLPCEMACNE--------------KLG-KSVV
XP_783181.3_NANS_Strongylo                                --------------------L-GSRVKEMRECELPTFN--------------KLG-KSVV
KMQ51676.1_Chitinispirillum                               --------------------M-GSFEKGIQDSEYPIFK--------------KLT-KSIV
OUX54928.1_Flammeovirgaceae_bacterium                     --------------------M-GIREKELQESEKPIFK--------------KLA-KSIV
OUV47462.1_Flavobacteriaceae_bacterium                    --------------------L-GSSEKTVQKSEIKVFK--------------KLG-KSLV
OIO66895.1_Candidatus_Marinimicrobia                      --------------------L-GTGEKRIQESEAPYFK--------------KLA-KSVV
WP_027268045.1_Legionella                                 --------------------L-GDGIKRCYQSEENPHY--------------KMA-KKIV
OGP61643.1_Deltaproteobacteria                            --------------------L-GNGIKTVYDIEKEPIK--------------KMG-KSIV
WP_022662582.1_Desulfovibrio                              --------------------L-GNGVKQPLDLEVSPIK--------------KMG-KKIV
OGP71522.1_Deltaproteobacteria                            --------------------L-GDSLKRPLPCEAKPLF--------------KMG-KKLV
Cladosiphon_okamuranus_BDDF01000009.1                     --------------------R-GDGQVRFYEDEKPIAE--------------KLR-R---
Elaphomyces_granulatus_NPHW01007775.1                     --------------------M-GDGIKKVYESELSSLK--------------KLR-K---
Thermobifida_fusca_NeuB_YXQ47U14                          --------------------L-GDGVKRVFPGEEEPRK--------------RLR-RTTA
Acytostellium_ellipticum_FLTD01002867.1                   --------------------T-GDGVKRVFDSERAPMA--------------KL------
Dictyostelium_deminutivum_FLTF01003146.1                  ------------------------------------------------------------
```

```
Lentinus_polychrous_JSYW01008743.1                    ----------------------GRGVKVPQPSEVSTGHTT------------SFR-RAAY
WP_035074769.1_Desulfovibrio                          ----------------------GSGDITVANCESGNRV--------------LIR-RSAA
SBT09427.1_Candidatus_Propionivibrio                  ----------------------GTGEKLPQEAEAPVIA--------------AAR-RSIV
WP_008313751.1_NeuB_Leptolyngbya                      ----------------------GNGVKTIQDAELPVAA--------------AAR-RSIV
WP_096701985.1_Magnetospirillum                       ----------------------GDGVKRVMPGEEATAQ--------------AAR-RSLC
Chromera_velia_ARZB01016018.1                         ----------------------GDGIKRIHEEEAPVAA--------------AAR-RSIC
ABS75841.1_SpsE_Bacillus_velezensis                   Q----------GHHAPVPEVLSGSSHKTTTPIEGDIRR--------------FAY-RGIF
Bacillus_subtilis_P39625.1_SpsE                       Q----------GITKPVSEKLLGSSYKTTTAIEGEIRN--------------FAY-RGIF
Prorocentrum_minimum2_JXLM01007734.1                  ----------------------GDSHKHPLP-----------------------------
Phytophthora_lateralis_AMZP02003661.1                 ----------------------GSDIKAPTASELPVRD--------------LVR-RSVT
AAC43302.1_neuB_Escherichia                           ----------------------GSNSKVVTASERKNKI--------------VAR-KSII
ABD95628.1_NeuB_Streptococcus                         ----------------------GRFEKIPDPVEEKNKI--------------VAR-KSVV
Tuber-aestivum_CZPR01000680.1                         ----------------------GDACKAPQPSEWDTRR--------------AAR-QQVV
Methanococcoides_burtonii_NeuB_NnaB_WP_011499634.1    ----------------------GSSVKAPVESELEVRD--------------IVR-RSIV
AEG18372.1_Methanobacterium                           ----------------------GTGIKELTIEEKEIKK--------------IAR-KSIV
WP_045444639.1_Neub_Tepidicaulis                      ----------------------GDGIKEPRGVETETAD--------------VAR-KSLV
WP_029638846.1_NeuB_alphaproteobacterium              ----------------------GDGCKEPQPAEISNMA--------------IGR-KSLV
Prorocentrum_minimum1_JXLM01000037.1                  ----------------------GDGCKEPQPAEISNMA--------------IGRSKSLV
Paramecium_biaurelia_JPFL01000669.1                   ---------------------SGSGIKEPSASENKNRA--------------IAR-KSLH
ADL05453.1_Clostridium                                ----------------------GDGVKRPTLAELKNRD--------------YVR-KYLV
EFM37961.1_Campylobacter                              ----------------------GDGIKKASKSERKNIN--------------IVR-KSLV
EIJ34212.1_Thiothrix                                  ----------------------GDGIKRASPNEAKNKP--------------IAR-KSIV
Condylostoma_magnum_CVLX01013231.1                    ----------------------GDGVKKPTKGELKNRP--------------IAR-KIIV

Treponema_pallidum_SpsE_NP_219001.1                   YLHAYPRGTVLQKENLVI-VR-SEA---NLSAGEAPEHANLF-VGAVLQRSVHAGEGARF
Reticulomyxa_filosa_ASPP01005516.1                    ------------------------------------------------------------
Neisseria_meningitidis_NeuB_NnaB_1XUU                 ADKDIKKGELLSGDNLWV-KR-PGN---GDF---SVNEYETL-FGKVAACNIRKGAQIKK
Neisseria_meningitidis_NeuB_2WQP_A                    ADKDIKKGELLSGDNLWV-KR-PGN---GDF---SVNEYETL-FGKVAACNIRKGAQIKK
Acanthamoeba_palestinensis_CDFD01053174.1             AIKDINKGEILTLDNIAA-VR-SPQ---GI----SSSQYNFV-LGKKAINNIQKHDPILA
Cladosiphon_okamuranus2_BDDF01000073.1                IAKAVKKGEVLTEQNLRI-VR-PGY---GL----APKHIDVL-LGRRV------------
Cronartium_ribicola_AWVX0102625.1                     ------------------------------------------------------------
CBA31353.1_Curvibacter                                LVHDLKAGTVLMPDHVRA-IR-PGF---GL----PPKYLTQV-LGRRLNRDVVRGTALAW
Hydra_vulgaris_ABRM01085349.1                         LVHDLKAGTVLMPDHVRA-IR-PGF---GL----PPKYLTQV-LGRRL------------
WP_018506507.1_pseudaminicAS_Thiobacillus             VVQDIKAGDVLTKENVRA-IR-PGL---GL----PTKFLGEI-LGKTVSQDVKRGTALNW
WP_009207339.1_pseudaminicAS_Sulfuricella             IVQDLKAGDVLTRENVRA-IR-PGL---GL----PTKYLEQI-LGKIVNQDVKRGTALGW
WP_067217189.1_pseudaminicAS_Marinomonas              IAQDLPAGTLLRPEHIKI-IR-PSL---GL----APKHWNEV-IGRTLSADVQLGQPLSW
WP_087462101.1_pseudaminicAS_Oleiphilus               VTQNLEPGAILTAENIKV-IR-PGY---GL----APKFFEEV-LGKKVNKAKLANTPLHA
Bankia_setacea_MRUH01000164.1                         ------------------------------------------------------------
KIX22924.1_NeuB_PseudAS_Flavobacterium                IAENITEGEVFTESNLRS-VR-PGF---GL----HPKYFNKI-IGKKANQKLEKGTPMSL
XP_001618767.1_NANS_Nematostella                      IAENITEGEVFTESNLRS-VR-PGF---GL----HPKYFNEI-IGKK-------------
WP_092376349.1_pseudaminicAS_Desulfomicrobium         FVRDLKKGAKITRDDIRR-IR-PGM---GL----APKYYESI-IGKKTKLSVLKGTATSK
Condylostoma_magnum2_CVLX01030206.1                   FVKDLPAGHVIGPEDIRR-IR-PGM---GL----APKYFNDV-LNKKLKVSVQQ------
AAD45660.1_NeuB_Aeromonas                             VIRDIKAGDVLSNDNVRS-IR-PGF---GL----APKYLEQV-LGKTAIVDIKRGTPLSF
WP_094706949.1_pseudaminicAS_Hahella                  AVKNIRKGEPFTLENVRS-IR-PGF---GL----APKHREEI-LGKSAAHDIEAGTALNW
Aureococcus_anophagefferens_ACJI01001814.1            ------------------------------------------------------------
Emiliania_huxley_AHAL01004984.1                       ------------------------------------------------------------
Melampsora_pinitorqua_AUYS0100826.1                   ------------------------------------------------------------
EEN59689.1_NANS_Branchiostoma                         LYMNVQCYDVFSVFSAG-VKAAEPF---GI----PPDQIYEL-VGQTVTKAIEEDATIPK
Q9VG74.2_NANS_Drosophila                              AARNLNKGYRLQLADMA-IKVSEPS---GL----TAEDFLDL-VGKELADNIGEDEPILG
EFX62694.1_NANS_Daphnia                               CTQKLEAGVQLCSEHLT-VKVGQPV---GW----PPQHLDQL-VGKTLSRNVDQDETITS
XP_008190466.1_NANS_Tribolium                         AATTLRKGDILQHENIK-IKVAEPK---GI----DASLLKDV-IGKHVKGVINEDESILQ
Q9NR45.2_NANS_HSA                                     AKVKIPEGTILTMDMLT-VKVGEPK---GY----PPEDIFNL-VGKKVLVTVEEDDTIME
XP_783181.3_NANS_Strongylo                            AAKDIPAGTVITEDMLT-VKVAVPK---GI----LPETIYNL-FGKRTAKAL--------
KMQ51676.1_Chitinispirillum                           SACELCKDDVITADMLT-TKG-PGT---GI----SPTRMEAV-IGKRVAKLIPADSVIKE
OUX54928.1_Flammeovirgaceae_bacterium                 TSKNIKKGEKIKSDMLT-TKG-PGS---GI----SPTKFNDI-IGKSLNKDVSIDVIIKD
OUV47462.1_Flavobacteriaceae_bacterium                TLKDIDINTIITREMIT-TKG-PGT---GI----SPMELGSI-LGKKTKVHLQKDVIIKE
OIO66895.1_Candidatus_Marinimicrobia                  STMPIPQGTIITRDMLT-TKG-PGT---GI----SPARIGDV-VGKSPKVDLKGDVVIRE
WP_027268045.1_Legionella                             AGRNLPIGHIISFEDLK-FKS-PAD---GV----PPYRVNEF-LGKKTLVALNEDDSVNF
OGP61643.1_Deltaproteobacteria                        ASRNLKKGMTVFLKDLA-FKS-PGG---GL----PPYEFEKI-IGKKLKVSMKEDQLFSL
WP_022662582.1_Desulfovibrio                          AARDLPAGTVLTFEDLA-FKC-PAD---GM----PPYELDNV-LGKTLTAELPEDGDLSF
OGP71522.1_Deltaproteobacteria                        AARDLPAGHVLTLQDVA-IKS-PND---GL----PPYEFDRV-MGQVLVRPLQEDENIAF
Cladosiphon_okamuranus_BDDF01000009.1                 ------------------------------------------------------------
Elaphomyces_granulatus_NPHW01007775.1                 ------------------------------------------------------------
Thermobifida_fusca_NeuB_YXQ47U14                      ------------------------------------------------------------
Acytostellium_ellipticum_FLTD01002867.1               ------------------------------------------------------------
Dictyostelium_deminutivum_FLTF01003146.1              ------------------------------------------------------------
Lentinus_polychrous_JSYW01008743.1                    ACRPVAAGATITADDLCV-LR-PAH---GT----DARDFDRI-VGATALHAFE-------
WP_035074769.1_Desulfovibrio                          ARGNIAEGSIVESDNIR-WVR-PGT---GI----TPDKSAMI-IGRKATRNLIDGELFKT
SBT09427.1_Candidatus_Propionivibrio                  ARLDLPAGHVLTEHDLE-WLR-PGG---GL----MPGQESIV-LGHRLLRPVVRGEMLLP
WP_008313751.1_NeuB_Leptolyngbya                      ARTSLTAGHVIGLEDLE-WLR-PGG---GL----SPGQEAAL-LGRCLQQAVAQGEMLTA
WP_096701985.1_Magnetospirillum                       AAADLPAGTVLGLGDLA-WLR-PAG---GM----APGAEWRI-LGRRLTRPVAAGDRLAL
Chromera_velia_ARZB01016018.1                         AARDLTPGQTVAPQDLT-WLR-PAG---GL----SPGSEADL-LGRSVRRPVPAGTALTL
ABS75841.1_SpsE_Bacillus_velezensis                   STAPIQKGECFTAENLA-VLR-PGQKPQGL----HPRYFELLTDGARAVRDIPADTGITW
Bacillus_subtilis_P39625.1_SpsE                       TTAPIQKGEAFSEDNIA-VLR-PGQKPQGL----HPRFFELLTSGVRAVRDIPADTGIVW
Prorocentrum_minimum2_JXLM01007734.1                  ------------------------------------------------------------
Phytophthora_lateralis_AMZP02003661.1                 TVRPLAAGAVVGREDVTL-MR-PGT---GI----PPVDLDKV-IGRKSARHIPAGETV--
AAC43302.1_neuB_Escherichia                           AKTEIKKGEVFSEKNIT-TKR-PGN---GI----SPMEWYNL-LGKIAEQDFIPDELIIH
ABD95628.1_NeuB_Streptococcus                         AXKPIKKGDIYSIENIT-VKR-PGN---GI----SPMNWYDI-LGQEAQDDFEEDEVIRD
Tuber-aestivum_CZPR01000680.1                         AARDIAKGSIIGRDDLT-TAR-ADS---GF----PAAALWEL-VGTRSEFAVQAGEII--
Methanococcoides_burtonii_NeuB_NnaB_WP_011499634.1    AKVNIPAGTVIAEDLLA-FKR-PGV---GM----PPKYLDRL-IGKVPISEIKKDSLINF
AEG18372.1_Methanobacterium                           ARANIPSETVITEEMLA-IKR-PGT---GI----EPKFLKSL-IGKTTTSKIKKDDFLRW
WP_045444639.1_Neub_Tepidicaulis                      AARPIAKGELFTEENLT-VKR-PGT---GL----SPLAFWSL-LGKPAPRAFAPDELIGL
WP_029638846.1_NeuB_alphaproteobacterium              ASTPINAGDVFTTENLT-VKR-PGT---GM----RPELYWSL-LGTHAARAYAPDDMITQ
Prorocentrum_minimum1_JXLM01000037.1                  ASTPINAGDVFTTENLT-VKR-PGT---GM----RPELYWSL-LGTHAARAYAPDDMITQ
Paramecium_biaurelia_JPFL01000669.1                   LREDLKKDTVLQAHHLM-ELR-PGD---GI----SPMDIDQV-IGKKII-----------
ADL05453.1_Clostridium                                AAREIRKGEAFTLHNLC-AKR-CGY---GI----SPMELHSL-LGKTADRNYQKDERIVL
EFM37961.1_Campylobacter                              AKKDIQKGEIFNEENLT-TKR-PAN---GI----SAMRYEEF-LGKIATKNYKEDELICE
EIJ34212.1_Thiothrix                                  AKQDIQAGKVFTLENLT-TKR-PGT---GV----SPMRWHEV-LGKTANRAFVADELIEL
Condylostoma_magnum_CVLX01013231.1                    AKCDINKGDKLNDKNLT-TK----------------------------------------
```

7 - **NANP**: N-acetylneuraminate-9-phosphate phosphatase (Neu5Ac-9-Pase; E.C. 3.1.3.29)

```
APD07702.1_NANP_Flavobacteriaceae                DNTLWDFIKNSKITLENLYKEHHVEEEHHIAFST--WYDHYYDINEQLWAEY-RDHKIT-
KLO97606.1_Fusarium                              DNTLFNHQQSIYYAMSAVRILLP--LPREIPLDV--LVDKYNEALDLVYNQY-LRNEIA-
OPB44498.1_Trichoderma                           DGTLFDHYHSLRLAITAIQRNYN--ELEEKNVDE--LIDKYNLALQRAYDAY-LDKVIT-
AMQ60564.1_dUMPphosphatase_Klebsiella            DETLFTFDSFSGLQRMFLDYSV--TF-TAKDF------QDYQAVNKPLWVDY-QNGAIT-
KLD50379.1_dUMP_phosphatase_Escherichia          DETLFTFDSFTGLQRMFLDYSV--TF-TAEDF------QDYQAVNKPLWVDY-QNGAIT-
XP_005772526.1_Emiliania                         DDTLVPTSKIDRAAILHAAA-----L-ASG--------DE---LPAVVAARF-SELLKA-
WP_007107590.1_HADfamhydrolase_Natrinema         DGTLLRYERSPGTV-------LEAAF-ERAGVEPLFAVDEYYARY---------------
WP_050914405.1_HADfamhydrolase_Vibrio            DETLCGTSQADRVAGQEFANWIAQTY-PQVADSTAFVQRYLQGVYKKLNHEF-PQLIAL-
WP_009842834.1_haloacid_dehalogenase_Vibrio      DETLCGTSQADKVAGQEFATWVAQTY-PQVNDSVALVQRYLQGVYKKLNAEF-PQLIAL-
XP_001415685.1_Ostreococcus                      DDTLAETTLADRVAYRECAIRMETVY-G---LSKK--------RQDEVIAAY-KRRLAE-
XP_007512834.1_NANP_Bathycoccus                  DDTLVPTSVYDVRAYADVKTSVLAWV-GEEKFQSL--------DVDKLVADF-KEKFVK-
ETR71346.1_NANP_CndtMagnetoglobus                DHTLVDCATADKRTYEI-----LATI-ARKQLDTI--------NTPSLIHDF-RQLLIK-
SNQ44313.1_NANP_Cellulophaga                     DDTLWVNETYFREAEETFAKLLEGYETKNK------------IDQELFKMEM-QNLGR--
EHX56208.1_FMN_phosphatase_Escherichia           DDTLYDNRPVILRTEREALTFVQNYHPALR------------SFQNEDL----QRLRQA-
WP_021711615.1_FMN_phosphatase_Vibrio            DDTLYDNVAVIVKMEQELLAWFKEHHPVVA------------QMSAEDW----RVVKRR-
KPV63575.1_HAD-hydrolase_CndtBathyarchaeota      DETLIDSFKGQQAAHIEVSKMLISSLKLRG-----------------IQVNF-QDLLRQ-
XP_015928753.1_NANP_Parasteatoda                 DNTLINTKGADRLACQQVSQYLQN-CGIRR------------STAREVVDKF-YNLIRE-
KXJ84308.1_NANP_Aedes                            Q---------NYIDAIIIADLLHKEYDLPR------------DLATEVSTTF-LTSYRR-
XP_008195993.1_NANP_Tribolium                    DNTLIATRKADKQTCSQLAQILWEKWDVPT------------DFATNASKAF-LKAFRK-
XP_017870155.1_NANP_Drosophila                   DNTLIPTRAGDSKAIRKLADVLETQYSFTK------------DDANLATQNF-LKSFRR-
CDG68557.1_NANP_Hydra                            -----------------------------P------------ATANLIVNEF-VTLVNK-
XP_001640674.1_Nematostella                      DNTLVQTNKSDLEALEKVKQWLMETL--SE------------EQALAATSEF-SRLLHE-
XP_020912840.1_NANPlq_Exaiptasia                 DNTLVTTTKSDIKALENVKERLSNDF--XQ------------ENVEAXANKY-LELLAI-
XP_019642312.1_NANPlq_Branchiostoma              DNTLIPTKEADDEAYELVREVIEEAC--PG------------YDTAAMTAKFRVDLGWN-
NP_689880.1_NANP_Homo                            DNTLIDTAGASRRGMLEVIKLLQSKY--HY------------KEEAEIICDK-VQVKLS-
XP_787883.3_NANP_Strongylocentrotus              DNTLIWTKQSDANAFVQVARFVQKET--PS------------CNAEEIVSTF-RKLLQS-

APD07702.1_NANP_Flavobacteriaceae                -KQ---------ELKDSRFRK--------AFQAVGVKD------------TSLP---EAF
KLO97606.1_Fusarium                              -HE---------DQDSVKVKL--------FFKSLHLEE------------PDSKCI-ARF
OPB44498.1_Trichoderma                           -YE---------QADVQKIHL--------FFSSLGLPE------------PSLDEI-QKF
AMQ60564.1_dUMPphosphatase_Klebsiella            ----------SLQLQH--------QRFDSWAERLNVPP--------------GELN-DAF
KLD50379.1_dUMP_phosphatase_Escherichia          ----------SLQLQH--------GRFESWAERLNVEP--------------GKLN-EAF
XP_005772526.1_Emiliania                         -EP-FPPEPSGLDVPAWRTGL--------WERALSGSSSDG--RAG-ASPAARQAY-EAW
WP_007107590.1_HADfamhydrolase_Natrinema         ------------DDFAERCDSMAALRSECFAALAAAN-------GY-DRRLGRDVA-AAF
WP_050914405.1_HADfamhydrolase_Vibrio            -LP---------DENAFRCGL--------IQTILAEQG-----IEI-DAEQAQQAQ-SFF
WP_009842834.1_haloacid_dehalogenase_Vibrio      -LP---------DENAFRCGL--------IQVILAEQG-----IEI-DAEQAQQAQ-NVF
XP_001415685.1_Ostreococcus                      -RP-WNDEF-A-HVWTHRERL--------WAEAFGDDD-----RG---LAMRHDVN-STF
XP_007512834.1_NANP_Bathycoccus                  -AP-WDPEY-KTDVFTWRSRV--------WEHALKLQN-----VEN-AEEVGVKCQ-KCF
ETR71346.1_NANP_CndtMagnetoglobus                -QP-FDPDG-RIDVHTWRIDL--------WTQALSKQN-----IDH-ED-LARQLN-HAF
SNQ44313.1_NANP_Cellulophaga                     --------Y-GYGIKAFVLSM--------VESALELSNN-----KV-SNTVIAKIL-NIG
EHX56208.1_FMN_phosphatase_Escherichia           -VREAEPEI-YHDVTRWRFRS--------IEQAMLDAGLSAEEASA-GAHAAMINF-AKW
WP_021711615.1_FMN_phosphatase_Vibrio            -LLREKPAL-KHDVTLLRFVQ--------IQAVFLSQGYSSLQANQ-IAQQAVELA-LEW
KPV63575.1_HAD-hydrolase_CndtBathyarchaeota      -VSDFDDEM-EVKLIRERDNW--------WPTLFRMLGGR----RL-SKKTAQRLTKAYW
XP_015928753.1_NANP_Parasteatoda                 -QPHDPNNE-DGDVDAWRVLL--------WKQALGP---------G-LSTKASIAY-SMW
KXJ84308.1_NANP_Aedes                            ----CPDNP-DVPLAQWRIQL--------WEDALPS---------D-RKHLAALVY-PRW
XP_008195993.1_NANP_Tribolium                    ----CPENL-SMSLDAWRRLL--------WAQALGD---------Q-YNKYAGEVY-RMW
XP_017870155.1_NANP_Drosophila                   ----CPDNS-QTSLDSWRTHL--------WRESLQQ---------K-HKHLAEQIY-PQW
CDG68557.1_NANP_Hydra                            -SE-KAIVY-ESDEDLWRKQI--------WQKVLIDN-------KA-ANVEIDTFY-SYY
XP_001640674.1_Nematostella                      -HW-VDPDG-TKSVHEWRTSL--------WLKAINIL-PE----NI-TNITAGELY-SFW
XP_020912840.1_NANPlq_Exaiptasia                 -GS-IDPEG-KVDPHLWRTLL--------WKKAIDFV-SK----ND-ENPKAESIY-NLW
XP_019642312.1_NANPlq_Branchiostoma              -DG-CDPEG-KLTVDEWRTKL--------WENILNK-QGI----TQ-YNGLPAKMY-ATW
NP_689880.1_NANP_Homo                            -KE-CFHPY-NTCITDLRTSH--------WEEAIQETKGG----AA-NRKLAEECY-FLW
XP_787883.3_NANP_Strongylocentrotus              -AE-KDPEN-KIPIDEWRTQL--------WKTALNS--------NQ-NEEFAARVY-QLW

APD07702.1_NANP_Flavobacteriaceae                EAVYLNHLPKNNFLRDGAHDLLEYLKKQNKYTLHIITN--GFKDVSLKKIKGSGIDSYFD
KLO97606.1_Fusarium                              RSVYKRTYRGKRKAMPGSIETLRSL-RKNGYRTAIITN--GPTEIQIEKAKAIGVFDLVD
OPB44498.1_Trichoderma                           RDTYKAVYRKNRRATPGSIEALARL-REHGYRIAIITN--GQVEDQTTKAKAIGIEHLID
AMQ60564.1_dUMPphosphatase_Klebsiella            MNA---MAEI-CAPLPGAVSLLNAL-Q-GKVKMGIITN--GFTSLQQTRLERTGLRDHFD
KLD50379.1_dUMP_phosphatase_Escherichia          INA---MAEI-CTPLPGAVSLLNAI-R-GNAKIGIITN--GFSALQQVRLERTGLRDYFD
XP_005772526.1_Emiliania                         SSE---RLSS-FRFADDVDAMVRRL-QSAGYKTGVLTN--GHADVQRAKTAACGAGSLFG
WP_007107590.1_HADfamhydrolase_Natrinema         NDE---RDQSNVELVPPAARVLDEL-S-REYRLAVITN--GARDAQRQKIDAVNLERWLD
WP_050914405.1_HADfamhydrolase_Vibrio            DSA---RMGA-FTFFPGVKEMLTEL-R-QHYKLVVITN--GPIFSQHPKIKATQMSEWVD
WP_009842834.1_haloacid_dehalogenase_Vibrio      DSA---RMQA-FTFFPGVKEMLTEL-R-QHYKLVVITN--GPVFSQHPKLKATQMSEWVD
XP_001415685.1_Ostreococcus                      RDC---RLEQ-LRLNSSVCGGIEKL-RAKNVHVVIITN--GHHVVQREKLAACGIYEVVK
XP_007512834.1_NANP_Bathycoccus                  DDN---RMGT-FPLHDCVKELSEYI-NSKGLEMCIITN--GHHRVQRDKLEACSAYTLFE
ETR71346.1_NANP_CndtMagnetoglobus                HFE---RLAF-YVLDHSVQTLLNDL-L-QTYTGIIITN--GDTTIQRPKLAACNAHQYFG
SNQ44313.1_NANP_Cellulophaga                     KQM---IDQP-VELLPGVESVLQKL-Q-SKYRLIVLTK--GDLLDQEQKLEKSGLKKYFH
EHX56208.1_FMN_phosphatase_Escherichia           R-------SR-IDVPQQTHDTLKQL-A-KKWPLVAITN--GNAQP-----ELFGLGDYFE
WP_021711615.1_FMN_phosphatase_Vibrio            R-------NR-VTIAPDNIALLQTL-A-EKVPLVAITN--GNVDC-----KKIGLTPYFQ
KPV63575.1_HAD-hydrolase_CndtBathyarchaeota      TAY---A-NN-SPPYEDAIPTLEYL-RSKGYKIGLVTDTDGTPGMKRWRVALTGLEKYVD
XP_015928753.1_NANP_Parasteatoda                 KES---RTIH-LKFDLALQNMLVEL-R-KHYKLGLITN--GPSSSQWEKIRKVNGSIFFD
KXJ84308.1_NANP_Aedes                            VEY---RTRY-LAPSPEIISMLQTL-R-LQYLLGIVTN--GPSASQWEKIDRLALGRFFD
XP_008195993.1_NANP_Tribolium                    LQL---RYDN-LALSPEIQNLLIKL-R-QHYFVGLITN--GTSRAQWEKIQLLHLQSFFD
XP_017870155.1_NANP_Drosophila                   LKL---RYRY-LAIPPDYVQLLQRM-RRAGYLLALITN--GPSNAQREKINKLHVRGHFD
CDG68557.1_NANP_Hydra                            KES---FLEL-IEIKKEVKKMLKNL-Q-NSFKIIILTN--GNSQWQRKKLEKSEANKYVD
XP_001640674.1_Nematostella                      RES---RVKG-LGIPTGVQFLLEGL-G-HQYKMAIITN--SDPVIQKEKLEFCKVEKYFD
XP_020912840.1_NANPlq_Exaiptasia                 RFA---RLEG-IKLEKEVADLLKNL-R-EKXKLSLVTN--SDPIIQREKLQSCGIIEYFD
XP_019642312.1_NANPlq_Branchiostoma              KRE---RLKR-MVFTEEIKQMLVGL-R-KEYKLQMMTN--GPLEPQREKSQVCESDVYFD
NP_689880.1_NANP_Homo                            KST---RLQH-MTLAEDVKAMLTEL-R-KEVRLLLLTN--GDRQTQREKIEACACQSYFD
XP_787883.3_NANP_Strongylocentrotus              KKL---RLEG-LYFDEEVRAQLKRL-R-LRYKLLLLTN--GDSQVQREKVAQIGAEDFFD

APD07702.1_NANP_Flavobacteriaceae                --VVVSAED-V-----------------------NTRKPDPKVFQHALDLAQA-SKEESI
KLO97606.1_Fusarium                              --CVITSQE-A-----------------------GHPKPDVRIFQYALEKLDV-KPDDAH
OPB44498.1_Trichoderma                           --RIITSEE-A-----------------------GYRKPDRRIFQYAIEQLGA-SLDTTC
AMQ60564.1_dUMPphosphatase_Klebsiella            --LLIISEE-V-----------------------GVAKPDARIFDYALAQAGNPSRDRVL
KLD50379.1_dUMP_phosphatase_Escherichia          --LLVISEE-V-----------------------GVAKPNKKIFDYALEQAGNPDRSRVL
XP_005772526.1_Emiliania                         EERVIIAGA-GDASTATVARARPLMGGCGPLGEHAEQKPHASIFRVA---------SATL
WP_007107590.1_HADfamhydrolase_Natrinema         --EVVVAGH-D-----------------------TPPKPDPEPFEQAMRLLDA-TPATAV
WP_050914405.1_HADfamhydrolase_Vibrio            --HIIVGGE-E-----------------------PEEKPAASIFHKALNLVDV-KPEEAI
WP_009842834.1_haloacid_dehalogenase_Vibrio      --HIIVGGE-E-----------------------PEEKPAASIFHKALNLVGA-SPEEAI
XP_001415685.1_Ostreococcus                      LENILVGGE-EVL-------------------AGRDEKPEASIFHEACKRVDV-VPDEVM
XP_007512834.1_NANP_Bathycoccus                  --NIIVGGE-EVL-------------------AGRKEKPDRGIFMKACKYVGC-LPSEAI
ETR71346.1_NANP_CndtMagnetoglobus                -QNIIVGGE-E-----------------------PHEKPHASIFEKACDMADC-SPQEAI
SNQ44313.1_NANP_Cellulophaga                     --HVEVLSD-K-----------------------K-----EENYKNLLDHLQI-KTSEFV
```

```
EHX56208.1_FMN_phosphatase_Escherichia           --FVLRAGP-H-----------------------GRSKPFSDMYFLAAEKLNV-PIGEIL
WP_021711615.1_FMN_phosphatase_Vibrio            --HILRAGP-D-----------------------GAAKPDRDMFQKAQQLLAL-PADNIL
KPV63575.1_HAD-hydrolase_CndtBathyarchaeota      --AVVVGGEDI-----------------------MERKPSPELFLLAARRLGA-PAERCL
XP_015928753.1_NANP_Parasteatoda                 --AIVVSGD-V-----------------------KHSKPGVAIFEDAFRILDS-SSLECV
KXJ84308.1_NANP_Aedes                            --CILVSSD-L-----------------------PWAKPDRNIFYAACHYLGV-RPEECA
XP_008195993.1_NANP_Tribolium                    --VVLVSGD-L-----------------------PWEKPHREIFNIACEYLGV-EPQQCI
XP_017870155.1_NANP_Drosophila                   --CVLVSSD-L-----------------------PWEKPHPEIFYAACNFLGV-KPHECA
CDG68557.1_NANP_Hydra                            --DIIISGE-H-----------------------KISKPDPRLFQLACLKLGL-ESKQCI
XP_001640674.1_Nematostella                      --AIIISGE-Q-----------------------PEAKPCVSIFQTACDAIGL-APEDCV
XP_020912840.1_NANPlq_Exaiptasia                 --XXIISGD-E-----------------------PXPKPHPSIFIKACANIGV-PPEQCI
XP_019642312.1_NANPlq_Branchiostoma              --SIVLCGL-Y-----------------------PEQKPYPSVYQRALQDLQL-NADQCV
NP_689880.1_NANP_Homo                            --AVVVGGE-Q-----------------------REEKPAPSIFYYCCNLLGV-QPGDCV
XP_787883.3_NANP_Strongylocentrotus              --EIVISGD-H-----------------------PEPKPHPSIFKTSCKLLGV-EASQCV

APD07702.1_NANP_Flavobacteriaceae                IIGDDYIADIVGGLDFGIQA-------IFYNILDMEVKNN-------------------N
KLO97606.1_Fusarium                              MVGDSVEADIKGALDAQISP-------ILYSPGSNSSLKLLFGK---------------E
OPB44498.1_Trichoderma                           MIGDSAESDIKGALDAQMFA-------IMYSPTAQDSQKLLFGQ---------------Q
AMQ60564.1_dUMPphosphatase_Klebsiella            MVGDTAESDIRGGVNAGLA-------TCWLNAHQQTLPADLQ-----------------P
KLD50379.1_dUMP_phosphatase_Escherichia          MVGDTAESDILGGINAGLA-------TCWLNAHNREQPEGIA-----------------P
XP_005772526.1_Emiliania                         MVGDSYAADVAGGINASLLA------TVWVRPPLEPPETGAQGSLMHGHLSAVPAGQPSP
WP_007107590.1_HADfamhydrolase_Natrinema         HVGDSLETDIVGSAAAGLE-------SVWVSDRSDGSGA---EP----T-----------
WP_050914405.1_HADfamhydrolase_Vibrio            HIGDSLPADIAGANNMGIL-------SVWVNETGTANPT-DIKP----N-----------
WP_009842834.1_haloacid_dehalogenase_Vibrio      HIGDSLPADIAGANNMGML-------SVWVNATGTENPT-DIEP----H-----------
XP_001415685.1_Ostreococcus                      HVGDSWTADMVGAENAGLRW------RVWVSQRPDDEKCESEQE----LSSSKRAKKVDA
XP_007512834.1_NANP_Bathycoccus                  HVGDSLGADIQGGINAELLA------TVFINVKSRDASTFQPMP----T-----------
ETR71346.1_NANP_CndtMagnetoglobus                MVGDRLHTDIQGGINAGLAA------TVWVNPNNDPLDHNGPEP----H-----------
SNQ44313.1_NANP_Cellulophaga                     MLGNSLKSDVLPIVKLGAQAVHIPFHTTWVHETVSKEE-----------------EESNS
EHX56208.1_FMN_phosphatase_Escherichia           HVGDDLTTDVGGAIRSGMQA------C-WIRPE-------NG------DLMQTWDSRLLP
WP_021711615.1_FMN_phosphatase_Vibrio            HVGDHLISDVKGAISANFAS------C-WFNNT-------GC------QIRTLCHGGLLP
KPV63575.1_HAD-hydrolase_CndtBathyarchaeota      MVGDKAFVDIKGAIAAGMKA------ILIHR--------------------REWDIPLKP
XP_015928753.1_NANP_Parasteatoda                 MVGDNLNTDIKGGIRANCAA------TVWIS---------DE------GL-PDREGCPKP
KXJ84308.1_NANP_Aedes                            MIGDKLETDIQGGLESRLAA------TIWLPLPKDLRLMHDK------SL-ADMGDLAHP
XP_008195993.1_NANP_Tribolium                    MVGDKLETDILGGLKAKLGG------TVWVPLNSIEVG--DE--------------DPRP
XP_017870155.1_NANP_Drosophila                   MIGDKLETDIKGGHLAQLGL------TFWLPLSTSSAA--SQ------CL-DD--VEYKP
CDG68557.1_NANP_Hydra                            MVGNNKKADIFGGFNAGLKA------TIWIR---------DK------EY---DNDSIQP
XP_001640674.1_Nematostella                      MIGDNLVDDIQGGRDAGVRA------TVWVR---------GE------DAKGPSEKGMKP
XP_020912840.1_NANPlq_Exaiptasia                 MIGDNXRHDIQGGVNAGLFA------TVWVK---------KE------SQQEMTISDPRP
XP_019642312.1_NANPlq_Branchiostoma              MVGDRLNTDIXGGLNAKLLS------TVWIN---------ST------GA-AIPEGGPVP
NP_689880.1_NANP_Homo                            MVGDTLETDIQGGLNAGLKA------TVWIN---------KN------GI-VPLKSSPVP
XP_787883.3_NANP_Strongylocentrotus              MVGDSQETDIQGGANARVLA------TVWIN---------PH------GK-QPSSDYVKA

APD07702.1_NANP_Flavobacteriaceae                FIEIKHLNEVKKYL-
KLO97606.1_Fusarium                              IPVIRQFDQLPMVLE
OPB44498.1_Trichoderma                           IPIIRHMAQLPGYLG
AMQ60564.1_dUMPphosphatase_Klebsiella            DWTVTSLSELEQLLC
KLD50379.1_dUMP_phosphatase_Escherichia          TWTVSSLHELEQLLC
XP_005772526.1_Emiliania                         TFTVESVLEVEACLE
WP_007107590.1_HADfamhydrolase_Natrinema         -YRVASIGDLLT--P
WP_050914405.1_HADfamhydrolase_Vibrio            -YEVKETVELKEILK
WP_009842834.1_haloacid_dehalogenase_Vibrio      -YEVRETIELNEILK
XP_001415685.1_Ostreococcus                      VPRVENIKEFFELLD
XP_007512834.1_NANP_Bathycoccus                  -YTIKHIAELKEIID
ETR71346.1_NANP_CndtMagnetoglobus                -FQVVSVLELPAILS
SNQ44313.1_NANP_Cellulophaga                     FLTLNKIEDILEYLK
EHX56208.1_FMN_phosphatase_Escherichia           HLEISRLASLTSLI-
WP_021711615.1_FMN_phosphatase_Vibrio            DMEVSQLASLSRLVE
KPV63575.1_HAD-hydrolase_CndtBathyarchaeota      HHTIHRLAELRSLL-
XP_015928753.1_NANP_Parasteatoda                 DFQVSNVTDIMDLLP
KXJ84308.1_NANP_Aedes                            DYIVESVLDLPSVLP
XP_008195993.1_NANP_Tribolium                    DYVIKNVTELPNLLP
XP_017870155.1_NANP_Drosophila                   HVKLNSLLDLYKYFP
CDG68557.1_NANP_Hydra                            DYIIDDICELESVLV
XP_001640674.1_Nematostella                      NFVVQSLLELPEVLN
XP_020912840.1_NANPlq_Exaiptasia                 DYTIDSVIELPKILA
XP_019642312.1_NANPlq_Branchiostoma              HHIVTSVLELPKILD
NP_689880.1_NANP_Homo                            HYMVSSVLELPALLQ
XP_787883.3_NANP_Strongylocentrotus              DYTIKSVLEIDSILQ
```
