# Supplementary material for: Reconstruction of the sialylation pathway in the ancestor of eukaryotes
Source: Sci Rep. 2018 Feb 13;8:2946. doi: 10.1038/s41598-018-20920-1 (PMC5811610; doi:10.1038/s41598-018-20920-1)
Supplement: Supplementary file 1 — Supplemental Dataset 1, Dataset 3, Dataset 4 and Supplemental Figures [file 41598_2018_20920_MOESM1_ESM.zip › supplemental data 4 AHL.pdf]

Reconstruction of the sialylation pathway in the ancestor of Eukaryotes

Daniel Petit[1], Elin Teppa[2], Ugo Cenci[3,4], Steven Ball[3,4] and Anne Harduin-Lepers[3,4]

[1]Laboratoire de Génétique Moléculaire Animale, UMR 1061 INRA, Université de Limoges Faculté des Sciences et Techniques, 123 avenue Albert Thomas, 87060 Limoges, France

[2]Bioinformatics Unit, Fundación Instituto Leloir -IIBBA CONICET, Av. Patricias Argentinas 435, C1405BWE, Buenos Aires, Argentina

[3]Univ. Lille, CNRS, UMR 8576 - UGSF - Unité de Glycobiologie Structurale et Fonctionnelle, F 59000 Lille, France

[4]UGSF, Bât. C9, Université de Lille - Sciences et Technologies, 59655, Villeneuve d'Ascq, France

Correspondence : Anne Harduin-Lepers, Laboratoire de Glycobiologie Structurale et Fonctionnelle, CNRS UMR 8576, Université Lille Nord de France, Lille 1, 59655 Villeneuve d'Ascq, France. Phone: +33 320 3362 46 ; FAX : +33 320 43 65 55 ; E-mail : anne.harduin@univ-lille1.fr

Short title: Origin and transfer of sialic acid in LECA

Supplemental data 4: Additional information on the other actors of the sialylation machinery evolutionary history

We reasoned that for efficient sialylation reactions by eukaryotic GT29 ST enzymes, an organism must dispose of a source of sialic acids and should be able to activate sialic acid in sugar-nucleotide *i.e.* CMP-sialic acid (Figure 1). However, despite a good understanding of the sialic acid metabolism pathways in Opisthokonta, Coelomata and Bacteria [1,2], nothing is known in protist organisms. Therefore, we also explored the evolutionary trajectories of the major components of the sialylation pathway that could provide the eukaryotic cell with sialic acid either from the Eukaryota environment (*i.e.* Sialidases (NEU) and the transporters Sialin (SLC35A5) and NanT) or from endogenous sialic acid biosynthetic pathway (*i.e.*: UDP-GlcNAc 2-epimerase, NeuAc-9-P synthase (NANS) and NANP) and those molecules that provide activated sialic acid donor substrate for the Golgi ST enzymes (*i.e.*: the CMP-NeuAc synthase CMAS and the Golgi transporter SLC35A1). In addition to the identification of homologous sequences with the BLAST search approach, we also checked their architecture domain using Conserved Domain Architecture Retrieval Tool C-Dart in NCBI (C-Dart, [3]) and SMART tools [4]. To conduct comparative genomic studies, we adopted a strategy at two levels. Using the human sequence as seed, we first searched for representative sequences of the families close to the designed one, given by C-Dart in NCBI. In a second step, we also searched in protein and WGS databases of NCBI the protein sequences close to the human in the different Eukaryota lineages defined in figure 2. We gave a special attention to Metazoa, exploring independently within the Sponges, Cnidaria, Arthropoda, Lophotrochozoa, and basal Deuterostoma, and for Archaeplastida within Prasinophyta and Streptophyta. For each eukaryotic sequence retrieved, we searched for the closest Bacteria and Archaea sequences to detect potential LGT. The MSA were conducted using Clustal Omega and the phylogenetic analyses with Minimum Evolution method, including the options JTT, partial deletion and 350 bootstrap replicates, implemented in MEGA 7.0.

1- Molecules that could provide the eukaryotic cell with exogenous sialic acid (*i.e.* Sialin/NanT and sialidases)

The bacterial transporter NanT and the Opisthokonta Solute Carrier SLC17A5, also known as sialin are single polypeptide carriers belonging to the very large Major Facilitator Superfamily (MFS) able to transport sugars inside the cells in response to chemiosmotic ion gradients [5]. NanT and sialin have different subcellular location, on the plasma and lysosome membrane respectively, and ensure the income of sialic acid in the cytoplasm of cells. Using the Bacteria *Mycobacterium* NanT sequence as a seed, we found NanT sequences in the Bacteria *Klebsiella* and *E. coli* and in a few Eukaryota like in the Insect *Drosophila elegans*, in the Fungi Basidiomycota *Gymnopus luxurianss*, *Calocera cornea* and *Hebeloma cylindrosporum*, and in the Ascomycota *Fonsecaea pedrosoi*. The C-Dart tool allowed the identification of sv2 (synaptic vesicular glycoprotein 2) as a family close to NanT. We found sv2 sequences in Metazoa from the Sponge *Amphimedon* to Man. As previously described, in Metazoa, the

SLC17A family encompasses 4 groups of structurally related proteins that mediate the transmembrane transport of organic anions [6,7]. To build our dataset, in addition to the Sialin group (SLC17A5), we introduced members of each group (VGLUTs = SLC17A6-8 ; VNUTs = SLC17A9 ; Type I phosphate transporters = SLC17A1-3). Using the human sialin sequence as a seed and BLAST search, we obtained 57 sequences, of which 41 sequences are of Metazoa, 4 of Bacteria, and the rest of other Eukaryota (Supplemental data 3). The NanT-sv2 and SLC17A subfamilies sequences were aligned to root the tree and organize the relationships between members of the SLC17 family. Most Metazoa sequences of our phylogeny shown in supplementary figure S4 fit in the groups defined previously [6,7]. The clades including the human sialin and corresponding to VGLUTs comprise only Metazoa sequences, from Cnidaria to human, and from Porifera to human, respectively. The SLC17A10 group, initially limited to *C. elegans* is enriched by Mollusca and Annelid sequences (Protostoma), the Type I phosphate transporter group, initially restricted to Vertebrata [6] is now enlarged to Deuterostoma with the sea-urchin *S. purpuratus* and the VNUT group comprising Bilateria in [6] now spreads from Cnidaria to human with the addition of Hydra, associated to parasitic protists (*Sporobolus*, *Cryptosporidium* and *Amphiamblys*). Taking into account non-Metazoa Eukaryota in our work highlights the presence of a new group named SLC17A11 at the base of the previous clades containing protist sequences, as the Prasinophyta *Bathycoccus prasinos* and *Micromonas*, the Stramenopile *Aureococcus*, and the Haptophyta *E. huxleyi*. Interestingly, there is an outgroup to the whole SLC17A family, composed of two γ-Proteobacteria and an Alveolata *Chromera velia*. To assign function to this new SLC17A11 clade, we blasted each sequence to the human proteome available in GenBank. The result reveals a majority of sequences related to sialin, the rest being closer to VGLUTs sequences further suggesting that the ancestral role for this family would be the transport of glutamate, glucuronic and sialic acids. This transport was likely achieved through vesicular membranes, as the only clade where a signal for membrane location is present (SLC17A1-4) is not basal in the phylogeny (Supplemental figure S4). Moreover, we suggest that the lysosomal location of the SLC17A5 transporter is a more recent given the terminal position of the sialin SLC17A11 clade, although these SLC17 transporters remain to be biochemically characterized. Altogether, our phylogenetic analysis suggests that the emergence of SLC17A sialic acid transporter through a vesicle membrane dates back to the ancestor of Eukaryota. Several duplication events and subsequent functional divergence have taken place very early during Metazoa evolution, since Cnidaria sequences are present in the 4 SLC17A clades. The γ-proteobacterial sequences at the root of SLC17A family further suggest an early LGT into to the genome of LECA, and a more recent one into the genome of *C. velia*.

Sialidases, also known as neuraminidases (NEU genes) release α-linked sialic acids from glycoconjugates and polysaccharides. These enzymes are common in Opisthokonta and also in microorganisms. The human enzymes NEU 1-4 classified in the CAZy GH33 family are specific of sialylated glycoconjugates and show characteristic tissue and cell expression pattern [8]. Interestingly, NEU 1 is found in the lysosome of Metazoa where it is involved in the hydrolysis of exogenous

sialyloglycoconjugates (Figure 1) and the recent and detailed phylogenetic analysis of Giacopuzzi *et al.* described NEU 1 as the most ancient neuraminidase [9]. We have extended the exploration of sialidases having the closest sequence to the human NEU 1 to protists and Bacteria using BLAST analysis in GenBank. In Metazoa, sialidases are restricted to human, Porifera, Cnidaria, and the Deuterostoma *S. kowalevskii*. The constructed dataset contained 45 sequences (Supplemental data 2), and their phylogenetic analysis revealed 6 star-like groups, similar to the previous analysis of Giacopuzzi *et al.* (Supplemental figure S5). The NEU 1 group is enriched by the recently sequenced Porifera *Oscarella lobularis* and the NEU 2-3-4 group is also enlarged by the marine endosymbiont α-Proteobacteria *Endozoicomonas* (Supplemental figure S5). The 4 remaining groups do not include any Metazoa. As the relative positions of these 6 clades have weak bootstrap support, we introduced other sialidase sequences found in *B. prasinos*, and several bacteria as *Janthinobacterium* and *Reticulomyxa* to provide better phylogenetic. We also considered the pairwise deletion instead of partial deletion option. The final result is that the groups 1 and 4 were always associated with NEU 1 and NEU 2/NEU 3/NEU 4 group respectively, whereas the positions of groups 2 and 3 were versatile. The clade formed by NEU 1 and group 1 contains γ-proteobacteria (*Psychromonas* and *Arthrobacter*) and Ascomycota Fungi (*Penicillium*, *Sporothrix* and *Pseudogymnoascus*). The clade formed by NEU 2/NEU 3/NEU 4 group and group 4 gathers mostly Bacteroïdetes Bacteria and Ascomycota Fungi like *Aspergillus fumigatus* [10,11], *Arthroderma* and *Metarhizium*, and the Haptophyta *Chrysochromulina* and the Euglenozoa *Trypanosoma*. Groups 2 and 3 associate the Alveolata Dinoflagellata *Karenia* and the Choanoflagellata *Monosiga*, as mentioned by Giacopuzzi *et al.* [9], and are extended to other protists: group 3 contains the Stramenopile *Blastocystis* and the Haptophyta *Chrysochromulina*, and group 2 includes 2 Stramenopiles, *A. anophagefferens* and *Chrysochromulina*, 1 Excavata Euglenozoa *Bodo saltans*, and 1 Haptophyta *Emiliania huxleyi*. As groups 2 and 3 are composed of diverse Eukaryota lineages, they are likely issued from duplications events that occurred in LECA. Interestingly, the clade NEU 1-group 1 (Opisthokonta) could result from an LGT with γ-Proteobacteria as sialidase-1 donors. In parallel, the clade NEU 2/NEU 3/NEU 4 - group 4 (Eukaryota) could result from another independent LGT with Bacteroïdetes Bacteria as alpha-exo-sialidase donors. As a result, we suggest that the different compartments of sialidase activities recorded in Vertebrata *i.e.*: lysosome and plasma membrane, would illustrate different LGT events, and not a change during evolution as previously hypothesized by Giacopuzzi *et al.* [9]. It will be interesting to explore the biochemical characteristics of sialidases belonging to groups 2 and 3. Of note, the sialidase of *Blastocystis*, the most prevalent eukaryotic gut parasites, could be inherited from duplication in LECA, as the other sequences of group 3, and not from a horizontal acquisition [12,13].

2- Molecules that could provide the eukaryotic cell with endogenous sialic acid (*i.e.* UDP-GlcNAc 2-epimerase, NANS and NANP)

The human *GNE* gene (Figure 1) encodes a bifunctional enzyme with uridine diphospho-*N*-acetylglucosamine (UDP-GlcNAc) 2-epimerase and *N*-acetyl-mannosamine (ManNAc) kinase activity due to the presence of an epimerase pfam domain (PF03568) and a kinase pfam domain (Repressor, Open Reading Frame, Kinase, or ROK, PF00480). Only the 2-epimerase domain known as NeuC is required for the biosynthesis of sialic acid in Bacteria and De Mendoza and Ruiz-Trillo [14] showed that the fused *GNE* gene is restricted to all Deuterostoma genomes, including the Acoele *Symsagittifera roscoffensis*. In their work, most Eukaryota sequences bearing the 2-epimerase domain (clade B) are distantly related to the sequences present in Deuterostoma (clade A), and clearly closer to a bacterial clade including *Dictyoglomus thermophilus*. These authors gave a convincing view that the 2-epimerase gene in Deuterostoma came from a LGT from a probable bacterial donor and/or could result from hidden paralogy (*i.e.* the 2-epimerase domain was present at the origin of Eukaryota and was lost in all lineages except in Deuterostoma). Here, we searched what kind of organisms could be candidate donors of 2-epimerase to Deuterostoma. Through BLAST exploration in NCBI and Compagen databases, we found new sequences mainly in Alveolata and Fungi. In addition, for each Eukaryota sequence, we searched for the closest bacterial sequences, making a dataset of 34 sequences (Supplemental data 3). As illustrated in supplemental figure S6, Eukaryota 2-epimerase sequences of clade A is now enriched with several Alveolata sequences (subclade A1), including the Ciliates *Condylostoma magnum* and *Paramecium biaurelia*, the Dinoflagellata *P. minimum*, and the Chromerida *C. velia*. Of the 3 sequences present in this last organism, 1 is related to the 2-epimerase sequence of the Prasinophyta *Micromonas commoda*, these 2 being themselves sisters to the Deuterostoma 2-epimerase sequences. Our phylogenetic analysis shows that no bacterial sequences are closer to Deuterostoma 2-epimerase than the one found in the protists *M. commoda* and *C. velia* suggesting the occurrence of LGT from Alveolata to Deuterostoma. Given the heterogeneous distribution of Bacteria hosting epimerase-2 gene in this subclade (α, β, γ, ε–Proteobacteria), these sequences could originate from several Eukaryota to Bacteria LGTs. In addition, there is a subclade A2 containing several Fungi Basidiomycota (*Gymnopus luxurians, Termitomyces sp.* and *Ganoderma lucidum*) and Ascomycota (*Talaromyces wortmanii*), associated with several α-Proteobacteria. It also includes the Alveolata Ciliate *Sterkiella histriomuscorum* associated with 2 different Proteobacteria. Altogether, the Deuterostoma 2-epimerase likely results from an LGT involving an Alveolata as donor and not bacteria as previously suggested and whether LECA possessed a 2-epimerase remains open question. If it is the case, Alveolata and Fungi could have inherited this enzyme from LECA, with a massive loss in other eukaryotic lineages followed by an LGT from an Alveolata to the ancestor of Deuterostoma. If not, Alveolata and Fungi have acquired this enzyme through independent LGT from different bacterial donors (α-Proteobacteria in the case of Fungi).

In the cytosolic compartment of Vertebrates, the *N*-acetylneuraminic acid synthase (NANS) catalyzes the direct formation of *N*-acetylneuraminic acid-9-P (NeuAc-9-P) from *N*-acetylmannosamine-6-P (ManNAc-6-P) and phosphoenolpyruvate (PEP) (Figure 1) and of the 2-keto-

3-deoxy-D-glycero-D-galacto-nononic acid (KDN-9-P) from mannose-6-P (Man-6-P) and PEP [15,16]. Interestingly, besides its major ManNAc-6-P synthase activity, the human NANS has been shown to produce also KDN-9-P from Mannose-6-P (Man-6-P) further suggesting a secondary KDN-9-P activity and an evolutionary related family of enzymes [17,18]. This enzyme belongs to the SPSE (Spore coat Polysaccharide Biosynthesis proteins) superfamily and includes two distinct domains: a C-terminal SAF domain (antifreeze protein like domain) and a N-terminal catalytic Neub domain. This superfamily also includes the prokaryotic Neub enzyme responsible of the synthesis of *N*-acetylneuraminic acid from *N*-acetylmannosamine in Bacteria and Archaea, the Pseudaminic acid synthase, the Legionaminic acid synthase [19-21]. To decipher the evolutionary relationships between these sialic acid synthases and their distribution in Eukaryota, we searched the sequences related to SPSE superfamily in the WGS and reference protein databases of NCBI. In addition, we systematically investigated the closest bacterial sequences to the ones found in Eukaryota, leading to 63 sequences in the dataset. After MSA and the construction of phylogenetic tree using Minimum Evolution implemented in MEGA7.0 (partial deletion, JTT matrix and 350 bootstrap replicates), we obtained the phylogenetical hypothesis shown in supplemental figure S7. Four groups emerged: NANS group is apart with a 98% bootstrap support, the Pseudaminic synthase group is sister to the Neub/N,N diacetyllegionaminate synthase group, each supported at 74%, and there is a fourth less supported Neub clade. Interestingly, there are Bacteria sequences in the NANS group and conversely Eukaryota sequences in the Neub group. More precisely, the NANS clade includes sequences of the Amoebozoa *Acytostellium ellipticum* and *Dictyostelium deminutivum* in the same subgroup as the Ascomycota Fungi *Elaphomyces granulatus* and the Stramenopile *Cladosiphon okamuranus*. Another subgroup contains Metazoa Deuterostoma (the sea urchin *Strongylocentrotus purpureus*, the amphioxus *Branchiostoma floridae* and *Homo sapiens*) and Arthropoda (the insects *Drosophila melanogaster* and *Tribolium castaneum* and the crustacean *Daphnia pullex*). We did not retrieve any sequence of Mollusc, Annelids, Cnidaria or Porifera. Chromalveolata *E. huxley* and the Stramenopile *Aureococcus anophagefferens* sequences were found in another subgroup mainly linked to δ-Proteobacteria. Another set of Bacteria Fibrobacteres *Chitinospirillum* and *Marinimicrobia sp.* and the Bacteroidetes *Flammeovirgaceae* and *Flavobacteriaceae* is a sister group of the previous one. These observations led us to the conclusion that NANS was present in LECA and subsequently lost in several eukaryotic lineages, as Archaeplastida and Alveolata, and several classes within Metazoa. The bacterial sequences could be the result of LGT from Eukaryota. The Neub/ N,N diacetyllegionaminate synthase clade is mainly composed of Archea and different classes of Bacteria sequences, and includes eukaryotic sequences: the Alveolata *Paramecium biaurelia, Prorocentrum minimum* and *Condylostoma magnum*, the Stramenopile *Phytophthora lateralis*, and the Fungi *Tuber aestivum*. As for *P. minimum* sequence, it is highly probable that it comes from an LGT through α-proteobacteria given the high bootstrap support. This view can be extended to all the other Eukaryota sequences of this group, as the basal Neub clades of are clearly Archea and Bacteria. The Pseudaminic acid

synthase clade also contains Bacteria and Eukaryota sequences, and there is clear evidence of several independent LGT events from Bacteria to Eukaryota. The Cnidaria *Hydra vulgaris* and *Nematostella vectensis* inherited their PseS sequences from the symbiont β-Proteobacteria *Curvibacter* and Bacteroidetes *Flavobacterium* respectively, the Mollusca *Bankia setacea* sequence from a γ-Proteobacteria, the Alveolata *Condylostoma magnum* from the δ-Proteobacteria *Desulfomicrobium*. The enzymatic activity of this enzyme in Eukaryota remains to be determined.

One of the final steps of the sialic acid synthesis is dephosphorylation of *N*-acylneuraminate 9-phosphate catalyzed by the cytosolic *N*-acetylneuraminate-9-phosphatase (NANP, figure 1). This enzyme belongs to the haloacid dehalogenase-like (HAD) hydrolases family, which catalyzes carbon or phosphoryl group transfer reactions onto widely diverse substrates. The HAD-like hydrolases are characterized by a highly conserved alpha/beta core domain and a small mobile cap domain. We searched for eukaryotic sequences in the protein division of GenBank using the human NANP sequence as a seed and retrieved Bacteria, Archaea and Eukaryota HAD-related sequences. Twenty-six aa sequences including NANP, dUMP and FMN phosphatases, were aligned and analyzed through ME method (JTT matrix, 350 bootstrap replicates and partial deletion as options).

The phylogenetic tree (Supplemental figure S8) contains a set of Metazoa sequences related to the human NANP, with a low bootstrap support: cnidarian and deuterostomes sequences ranging from the sea urchin *S. purpuratus* to *Homo sapiens,* and Arthropoda sequences from the house spider *Parasteatoda tepidariorum* to insects like *Drosophila arizonae*. Next to this Metazoa group, there is a clade including other Eukaryota, as the Prasinophyta *Ostreococcus lucimarinus* and *B. prasinos*, the Haptophyta *E. huxleyi*, the δ-Proteobacteria *Candidatus Magnetoglobus* and the γ-Proteobacteria *Vibrio harveyi*. At the base of the previous group, there is another clade containing Eukaryota including the Fungi *Fusarium fujikuroi* and *Trichoderma guizhouense* related to the Bacteria NANP and dUMP phosphatases. Finally, we also found a fifth clade of bacterian FMN phosphatases that are not directly related to the eukaryotic NANP sequences and could represent an orthologue of the NANP enzyme present in the ancestor of Eukaryota. As for Fungi, there is a doubt whether they possess a true NANP or a dUMP phosphatase activity. Nevertheless, NANP seems to have disappeared in the Metazoa sponges, Lophotrochozoa and Nematodes. Apart from Opisthokonta, they also got lost in SAR, Amoebozoa and Streptophyta among the Archaeplastida. Regarding the origin of NANP in Eukaryota, closely related sequences are found in γ- and δ-Proteobacteria. However, because of a weak bootstrap support, it is difficult to determine the orientation of the LGT.

3- Molecules providing activated sialic acid donor substrate for ST Golgi enzymes

As illustrated in figure 1, activation of the nine-carbon amino sugars to the sugar-nucleotide cytidine monophosphate sialic acid (CMP-sialic acid) is catalyzed by the cytidine monophosphate N-acetylneuraminic acid synthetase (CMAS or CSS) in the nucleus of mammalian cells [22]. Intriguingly,

Zebrafish possess two copies of the CMAS enzyme with different subcellular localization and slightly different enzymatic specificities since CMAS1 is found in nucleus and favor CMP-Neu5Ac biosynthesis, whereas CMAS2 is retained in the cytosol and drives the expression of CMP-Kdn [23]. A unique *cmas* gene was identified in *Drosophila melanogaster* genome [24] and the cognate DmCMAS enzyme was shown to be localized in the Golgi apparatus with evolutionary adaptation to pH and ionic environment [25,26]. The biological significance of the nuclear sequestration of the mammalian enzymes is still an enigma. However, the evolution of the different subcellular localizations of CMAS is highlighted by the presence of a stretch of basic aa KRRKSRH in the *N*-terminus of the protein targeting the enzyme into the nucleus, shared by the fish *Danio rerio* DreCMAS1 and Human sequence, and absent in non-vertebrate sequences, suggesting that the 2-R event generated 2 copies resulting in two subcellular locations in early vertebrates. An analysis of protein modular architecture of CMAS sequences using C-Dart in NCBI [3] revealed that the CMAS family with Pfam domain PF02348 includes the bacterial 4-acyl neuraminate cytidyltransferase (NeuA) and the Pseudaminic acid cytidylyltransferase (PseF), also known as flagellum modification protein B described in *C. jejuni* and *C. pylori* [20] and the CMP-Kdo synthetase (KdsB) involved in the biosynthesis pathway of the bacterial LPS from UDP-GlcNAc. We systematically blasted protists, Metazoa and Archaeplastida genomes using the sequences of these three enzyme families. After MSA and construction of phylogenetic tree using Minimum Evolution (Supplemental figure S9), we found several monophyletic groups. In Metazoa, orthologous sequences of human CMAS were retrieved in the Cnidaria *Podocoryne carnea* and *Galaxea*, the Deuterostoma *S. purpuratus* and the Arthropoda *Daphnia magna* and *Drosophila bipectinata* among others. Another set of sequences related to bacterial NeuA was found in the Stramenopile *A. anophaggerens*, the Haptophyta *E. huxleyi*, *Chrysochromulina sp.*, and the Alveolata *C. velia*. The sequence of the Alveolata *P. minimum* is closely related to bacterial sequences suggesting LGT. Regarding the bacterial PseF enzyme, we found orthologous sequences in the Cnidaria *Hydra* sp. and *Thelohanellus kitauei*, in the Ciliates *Paramecium biaurelia* and *Condylostoma magnum*. Sequences related to the CMP-Kdo synthetase form a strong monophyletic group more distantly related to CMAS sequences. It includes several sequences found in Fungi, in the Amoeobozoa *Dictyostelium polycephalum*, in the Archaeplastida *B. prasinos* and *Zea mays* and in the Cryptophyta *G. theta*. Two Metazoa contain orthologues for this enzyme, the sponge *Amphimedon queenslandica* and the Cnidaria *Hydra* sp. The eccentric position of *A. queenslandica* is due to its incomplete sequence. In summary, each of the 3 CMAS-related families likely emerged in the LECA.

The human protein SLC35A1 is a nucleotide sugar transporter localized in the Golgi membranes of mammalian cells (Figure 1) that acts as an antiporter ensuring the exchange of a CMP-NeuAc molecule from cytosol into the Golgi compartment and a CMP molecule [27,28]. We identified homologous SLC35A1 sequences in eukaryotic genomes and to assess the presumed function of these sequences, we introduced in the dataset human members of the four other nucleotide-sugar carriers of the SLC35A family, named SLC35A2-5 [29]. Interestingly, although these organisms lack Golgi

organelle, we found several bacterial SLC35A-like sequences that were annotated as EamA family transporter [30]. The less distantly related sequences were found in *Pontibacillus*, *Acinetobacter*, *Phaeomarinobacter* and *Desulfovibrio*. The whole dataset contains 69 sequences, and 199 aa positions were selected in the final alignment (Supplemental data 3). The family of nucleotide-sugar transporters SLC35A is restricted to Eukaryota, as the 4 bacterial sequences stand apart. Within Eukaryota, the sequences are organized in 4 monophyletic groups. The two families known as SLC35A2 and SLC35A3 are sister groups ensuring the transfer of UDP-galactose (UDP-Gal) and UDP-*N*-acetylglucosamine (UDP-GlcNAc), respectively. Interestingly, SLC35A3 is restricted to Opisthokonta, *i.e.* Metazoas from Sponges to Human and Fungi, although there is a sequence in the Green Plant *Anthurium* (Supplemental figure S10). The second SLC35A2 family is even more limited as only Metazoa sequences from Sponges to Human could be retrieved. The third family corresponds to SLC35A1 (CMP-NeuAc transporter) and is sister group to the 2 previous ones. These SLC35A1-like sequences are found in Deuterostoma from Cephalochordata (*B. floridae*) to Human, Excavata (*B. saltans*, *Diplonema papillatum*, *Trypanosoma grayi*), Haptophyta (*E. huxleyi* and *Chrysochromulina* sp). The fourth family is the most basal within Eukaryota and is assigned to SLC35A4-5. This group contains both Metazoa sequences including Arthropoda (*D. elegans* and *Tribolium castaneum*), and also Archaeplastida sequences from the Prasinophyta *B. prasinos* to Angiosperms. Although the precise function of SLC35A4-5 transporters remains unknown, it was hypothesized that SLC35A4 could be involved in CMP-Kdo translocation in plants [31]. As most Arthropoda SLC35A sequences belong to this group, it is probable that these proteins ensure the transport of CMP-Sialic acid. Vertebrate proteins of this SLC35A group still await biochemical characterization and given its distribution, the SLC35A1 transporter is likely to be ancestral in Eukaryota.

4- Towards a model of the evolution of the sialylation pathway in Eukaryota

Altogether, our phylogenetic analysis indicates that the sialic acid source in LECA could be ensured either by an intake from the extracellular space *via* the lysosomal SLC17A transporter or could result from an endogenous biosynthesis from UDP-GlcNAc. The SLC17A transporters duplicated at least for times in LECA and later on the SLC17A5 evolved a differentiated function assigned to sialic acid transport**.** We further hypothesized that the basal member of this family, *i.e.* SLC17A11 likely ensured transport of sialic acid molecule in a nonspecific manner. Both transporters can be associated to sialidases Neu1 and/or Neu2-4. Regarding the intrinsic synthesis of sialic acid, the first step is accomplished by the UDP-GlcNAc 2-epimerase scarcely found in Eukaryota and mainly in Alveolata. This restricted distribution suggests an LGT from α- or β-Proteobacteria to Alveolata, although it does not exclude a basal presence of the 2-epimerase in LECA followed by a massive loss in several Eukaryota lineages. Interestingly, the NANS and NANP enzymes are widely distributed in most Eukaryota lineages, even in these lineages where the 2-epimerase is absent. This odd observation

favors the assumption of an ancestral presence in LECA of NANS and NANP, but not of the 2-epimerase further suggesting that the fundamental origin of the biosynthetic pathway in LECA could be ManNAc, and not UDP-GlcNAc.

In Metazoa, the extrinsic source of sialic acid through SLC17A5 (sialin) and intrinsic one through the canonical biosynthetic pathway from UDP-GlcNAc are found only in Deuterostoma, given the exclusive presence of GNE, resulting as a probable LGT from symbiotic or parasitic marine Alveolata. In Protostoma, these two origins are limited to Arthropoda, where the starting step of sialic acid synthesis begins with ManNAc-6-P [32]. In Cnidaria, biosynthesis of sialic acid appears to be missing since only the last step of the synthesis of sialic acid can be achieved with NANP. This raises the question of the origin of the mandatory Neu5Ac-9P. However, these organisms still can use exogenous sialic acid *via* an import from the environment owing the presence of the transporter SLC17A5 and the sialidase Neu1. In Fungi, a few species have acquired one enzyme of the biosynthetic pathway, *e.g.* UDP-GlcNAc 2-epimerase in *Spizellomyces* and *Termitomyces* through independent LGT events, and NANS and Neub in the ascomycetes *Elaphomyces* and *Tuber* respectively, raising serious doubts on their capacity to achieve this synthesis. Despite the loss of the SLC17A transporters, an uptake can be achieved in Basidiomycota and in a few Ascomycota by NanT, probably inherited through LGT from γ-Proteobacteria or Actinobacteria. Moreover, Ascomycota Fungi have neuraminidases close to the Neu2-4 group or NEU 1 group according to the species.

The situation is comparable in Amoebozoan where a NANS sequence could be retrieved in *Dictyostelium* and *Acytostelium*, although its role in sialic acid biosynthesis remains to be demonstrated. Concerning the sialic acid transport within the cell, the only transporter found in *Dictyostelium* belongs to SLC17A9 group and does not correspond to the canonical enzyme.

In Archaeplastida, the Prasinophyta are the only group handling sialic acid metabolism although they can unlikely ensure its biosynthesis, given the absence of NANS. However, Prasinophyta may use sialic acid from their environment thanks to the SLC17A11 transporter. Both potential sialic acid sources appear to be lost in Streptophyta.

In SAR, the most remarkable feature in the sialic acid synthetic pathway is the replacement of the canonical eukaryotic pathway by a bacterial one in Alveolata, involving a Neub protein acquired through a LGT event from an as yet unknown category of Proteobacteria. Regarding intake, its possibility is limited to *Chromera*, ensured by the transporter SLC17A11 owing to a specific LGT from γ-proteobacteria. In the Stramenopiles, the sialic acid biosynthesis could start with ManNAc as there is Neub in *Phytophtora* and an NANS orthologous sequence in *Cladosiphon* and *Aureococcus*, which could play the function of Neub as no NANP exists in this lineage. In addition, the SLC17A11 transporter exists in *Aureococcus*, which could insure sialic acid uptake.

In Hacrobia, the endogenous biosynthesis of sialic acid could start from ManNAc in the the Haptophyta *E. huxleyi* and an uptake of exogenous sialic acid involving the SLC17A11 transporter could exist. In the Cryptophyta *Guillardia*, the only way to acquire sialic acid could be the intake, through an alternative transporter, related to SLC17A10.

In conclusion, the availability of sialic acid within LECA has a double origin, although one of these might have disappeared in several lineages. For instance, the uptake mechanism has disappeared in most Alveolata and likely in Amoebozoa, whereas the biosynthetic pathway was lost in Cnidarian and Porifera. In the Archaeplastida Streptophyta and Metazoa Lophotrochozoa, both sialic acid sources are lost. Interestingly, the canonical eukaryotic pathway of sialic acid biosynthesis is replaced by a bacterial one in Alveolata. In some Fungi, *e.g.* the Basidiomycota, the SLC17A5 transporter typical of Eukaryota is replaced by the NanT transporter of bacterial origin.

References

1 Li, Y. & Chen, X. Sialic acid metabolism and sialyltransferases: natural functions and applications. *Appl Microbiol Biotechnol* **94**, 887-905, doi:10.1007/s00253-012-4040-1 (2012).

2 Vimr, E. R., Kalivoda, K. A., Deszo, E. L. & Steenbergen, S. M. Diversity of microbial sialic acid metabolism. *Microbiol Mol Biol Rev* **68**, 132-153 (2004).

3 Geer, L. Y., Domrachev, M., Lipman, D. J. & Bryant, S. H. CDART: protein homology by domain architecture. *Genome Res* **12**, 1619-1623, doi:10.1101/gr.278202 (2002).

4 Letunic, I., Doerks, T. & Bork, P. SMART: recent updates, new developments and status in 2015. *Nucleic Acids Res* **43**, D257-260, doi:10.1093/nar/gku949 (2015).

5 Thomas, G. H. Sialic acid acquisition in bacteria-one substrate, many transporters. *Biochem Soc Trans* **44**, 760-765, doi:10.1042/BST20160056 (2016).

6 Reimer, R. J. SLC17: a functionally diverse family of organic anion transporters. *Mol Aspects Med* **34**, 350-359, doi:10.1016/j.mam.2012.05.004 (2013).

7 Sreedharan, S. *et al.* Glutamate, aspartate and nucleotide transporters in the SLC17 family form four main phylogenetic clusters: evolution and tissue expression. *BMC Genomics* **11**, 17, doi:10.1186/1471-2164-11-17 (2010).

8 Monti, E. *et al.* Sialidases in vertebrates: a family of enzymes tailored for several cell functions. *Adv Carbohydr Chem Biochem* **64**, 403-479, doi:10.1016/S0065-2318(10)64007-3 (2010).

9 Giacopuzzi, E., Bresciani, R., Schauer, R., Monti, E. & Borsani, G. New insights on the sialidase protein family revealed by a phylogenetic analysis in metazoa. *PLoS One* **7**, e44193 (2012).

10 Telford, J. C. *et al.* The Aspergillus fumigatus sialidase is a 3-deoxy-D-glycero-D-galacto-2-nonulosonic acid hydrolase (KDNase): structural and mechanistic insights. *J Biol Chem* **286**, 10783-10792, doi:10.1074/jbc.M110.207043 (2011).

11 Warwas, M. L. *et al.* Cloning and characterization of a sialidase from the filamentous fungus, Aspergillus fumigatus. *Glycoconj J* **27**, 533-548, doi:10.1007/s10719-010-9299-9 (2010).

12 Eme, L., Gentekaki, E., Curtis, B., Archibald, J. M. & Roger, A. J. Lateral Gene Transfer in the Adaptation of the Anaerobic Parasite Blastocystis to the Gut. *Curr Biol* **27**, 807-820, doi:10.1016/j.cub.2017.02.003 (2017).

13 Moreira, D. & Lopez-Garcia, P. Protist Evolution: Stealing Genes to Gut It Out. *Curr Biol* **27**, R223-R225, doi:10.1016/j.cub.2017.02.010 (2017).

14 de Mendoza, A. & Ruiz-Trillo, I. The mysterious evolutionary origin for the GNE gene and the root of bilateria. *Mol Biol Evol* **28**, 2987-2991, doi:10.1093/molbev/msr142 (2011).

15 Angata, T., Nakata, D., Matsuda, T., Kitajima, K. & Troy, F. A., 2nd. Biosynthesis of KDN (2-keto-3-deoxy-D-glycero-D-galacto-nononic acid). Identification and characterization of a KDN-9-phosphate synthetase activity from trout testis. *J Biol Chem* **274**, 22949-22956 (1999).
16 Inoue, S. & Kitajima, K. KDN (deaminated neuraminic acid): dreamful past and exciting future of the newest member of the sialic acid family. *Glycoconj J* **23**, 277-290, doi:10.1007/s10719-006-6484-y (2006).
17 Cotton, T., Parker, E. & Joseph, D. The role of sialic acid synthases in sialic acid biosynthesis. *Chemistry in New Zealand* **78**, 69-74 (2014).
18 Hao, J., Vann, W. F., Hinderlich, S. & Sundaramoorthy, M. Elimination of 2-keto-3-deoxy-D-glycero-D-galacto-nonulosonic acid 9-phosphate synthase activity from human N-acetylneuraminic acid 9-phosphate synthase by a single mutation. *Biochem J* **397**, 195-201, doi:10.1042/BJ20052034 (2006).
19 Kandiba, L. & Eichler, J. Analysis of putative nonulosonic acid biosynthesis pathways in Archaea reveals a complex evolutionary history. *FEMS Microbiol Lett* **345**, 110-120, doi:10.1111/1574-6968.12193 (2013).
20 Schoenhofen, I. C., McNally, D. J., Brisson, J. R. & Logan, S. M. Elucidation of the CMP-pseudaminic acid pathway in Helicobacter pylori: synthesis from UDP-N-acetylglucosamine by a single enzymatic reaction. *Glycobiology* **16**, 8C-14C, doi:10.1093/glycob/cwl010 (2006).
21 Schoenhofen, I. C., Vinogradov, E., Whitfield, D. M., Brisson, J. R. & Logan, S. M. The CMP-legionaminic acid pathway in Campylobacter: biosynthesis involving novel GDP-linked precursors. *Glycobiology* **19**, 715-725, doi:10.1093/glycob/cwp039 (2009).
22 Sellmeier, M., Weinhold, B. & Münster-Kühnel, A. in *SialoGlyco Chemistry and Biology I: Biosynthesis, structural diversity and sialoglycopathologies* 139-167 (Springer Berlin Heidelberg, 2015).
23 Schaper, W. *et al.* Identification and biochemical characterization of two functional CMP-sialic acid synthetases in Danio rerio. *J Biol Chem* **287**, 13239-13248, doi:10.1074/jbc.M111.327544 (2012).
24 Viswanathan, K. *et al.* Expression of a functional Drosophila melanogaster CMP-sialic acid synthetase. Differential localization of the Drosophila and human enzymes. *J Biol Chem* **281**, 15929-15940, doi:10.1074/jbc.M512186200 (2006).
25 Di, W. *et al.* Diverse subcellular localizations of the insect CMP-sialic acid synthetases. *Glycobiology* **27**, 329-341, doi:10.1093/glycob/cww128 (2017).
26 Mertsalov, I. B., Novikov, B. N., Scott, H., Dangott, L. & Panin, V. M. Characterization of Drosophila CMP-sialic acid synthetase activity reveals unusual enzymatic properties. *Biochem J* **473**, 1905-1916, doi:10.1042/BCJ20160347 (2016).
27 Lepers, A. *et al.* Transport of CMP-N-glycoloylneuraminic acid into mouse liver Golgi vesicles. *FEBS Lett* **250**, 245-250 (1989).
28 Lepers, A. *et al.* A study on the regulation of N-glycoloylneuraminic acid biosynthesis and utilization in rat and mouse liver. *Eur J Biochem* **193**, 715-723 (1990).
29 Orellana, A., Moraga, C., Araya, M. & Moreno, A. Overview of Nucleotide Sugar Transporter Gene Family Functions Across Multiple Species. *J Mol Biol* **428**, 3150-3165, doi:10.1016/j.jmb.2016.05.021 (2016).
30 Vastermark, A., Almen, M. S., Simmen, M. W., Fredriksson, R. & Schioth, H. B. Functional specialization in nucleotide sugar transporters occurred through differentiation of the gene cluster EamA (DUF6) before the radiation of Viridiplantae. *BMC Evol Biol* **11**, 123, doi:10.1186/1471-2148-11-123 (2011).
31 Bakker, H. *et al.* A CMP-sialic acid transporter cloned from Arabidopsis thaliana. *Carbohydr Res* **343**, 2148-2152, doi:10.1016/j.carres.2008.01.010 (2008).
32 Koles, K., Repnikova, E., Pavlova, G., Korochkin, L. I. & Panin, V. M. Sialylation in protostomes: a perspective from Drosophila genetics and biochemistry. *Glycoconj J* **26**, 313-324 (2009).
