# Supplementary material for: Reconstruction of the sialylation pathway in the ancestor of eukaryotes
Source: Sci Rep. 2018 Feb 13;8:2946. doi: 10.1038/s41598-018-20920-1 (PMC5811610; doi:10.1038/s41598-018-20920-1)

Reconstruction of the sialylation pathway in the ancestor of eukaryotes

Daniel Petit[1], Elin Teppa[2], Ugo Cenci[3,4], Steven Ball[3,4] and Anne Harduin-Lepers[3,4]

[1]Laboratoire de Génétique Moléculaire Animale, UMR 1061 INRA, Université de Limoges Faculté des Sciences et Techniques, 123 avenue Albert Thomas, 87060 Limoges, France

[2]Bioinformatics Unit, Fundación Instituto Leloir -IIBBA CONICET, Av. Patricias Argentinas 435, C1405BWE, Buenos Aires, Argentina

[3]Univ. Lille, CNRS, UMR 8576 - UGSF - Unité de Glycobiologie Structurale et Fonctionnelle, F 59000 Lille, France

[4]UGSF, Bât. C9, Université de Lille - Sciences et Technologies, 59655, Villeneuve d'Ascq, France

Correspondence : Anne Harduin-Lepers, Laboratoire de Glycobiologie Structurale et Fonctionnelle, CNRS UMR 8576, Université Lille Nord de France, Lille 1, 59655 Villeneuve d'Ascq, France. Phone: +33 320 3362 46 ; FAX : +33 320 43 65 55 ; E-mail : anne.harduin@univ-lille1.fr

List of supplementary Figures:

Supplementary figure S1: Evolutionary relationships between ST of the CAZy family GT29 and GT42 sequences.

Supplementary figure S2: Bayesian phylogeny of 180 ST-like sequences of the GT29 CAZy family.

Supplementary figure S3: Bayesian phylogeny of the 180 ST sequences of the GT29 CAZy family and the 4 ST sequences of the GT42 CAZy family.

Supplemental figure S4: Sialin (SLC17A5) phylogenetic tree.

Supplemental figure S5: Sialidases (Neu) phylogenetic tree**.**

Supplemental figure S6: Uridine diphospho-N-acetylglucosamine (UDP-GlcNAc) 2-epimerase phylogenetic tree

Supplemental figure S7 : NANS phylogenetic tree.

Supplementary figure S8: NANP phylogenetic tree.

Supplementary figure S9: CMP-sialic acid synthase (CMAS or CSS) phylogenetic tree.

Supplementary figure 10: Nucleotide-sugar Transporters (NST) solute carrier family SLC35A phylogenetic tree.

Supplementary figure legends

Supplementary figure S1: **Evolutionary relationships between ST of the CAZy family GT29 and GT42 sequences.** A) The evolutionary history of the 180 ST of the CAZy family GT29 and the 5 ST of the CAZy family GT42. The evolutionary history was inferred using the ME method and the optimal tree with the sum of branch length = 60.99717189 is shown. The percentage of replicate trees in which the associated taxa clustered together in the bootstrap test (350 replicates) are shown next to the branches. The tree is drawn to scale, with branch lengths in the same units as those of the evolutionary distances used to infer the phylogenetic tree. The evolutionary distances were computed using the JTT matrix-based method and are in the units of the number of amino acid substitutions per site. The ME tree was searched using the Close-Neighbor-Interchange (CNI) algorithm at a search level of 1. The Neighbor-joining algorithm [1] was used to generate the initial tree. The analysis involved 185 amino acid sequences and all positions with less than 95% site coverage were eliminated. That is, fewer than 5% alignment gaps, missing data, and ambiguous bases were allowed at any position. There were a total of 92 positions in the final dataset. Evolutionary analyses were conducted in MEGA7.0 [2] and bootstrap support values were estimated from 350 replicates. We also midpoint rooted the tree, and observed the GT42 as an outgroup for a better appreciation of the bacterial outgroup of GT29 ST. Colors of the leaves represent the affiliation of sequences to their respective phylum Archaea (fluorescent green), Bacteria GT29 (orange), Bacteria GT42 (black), Opisthokonta (red), Archaeplastida (green), Protists (pink). Colors of the branches refer to the known functions of ST in Opisthokonta (blue: ST3Gal; yellow: ST6Gal; brown: ST6GalNAc and purple: ST8Sia).

Supplementary figure S2: Bayesian phylogeny of 180 ST-like sequences of the GT29 CAZy family. The consensus tree shown here was obtained with Phylobayes 4.1 with ML boostrap values (BS) and bayesian posterior probabilities (pp) mapped onto the nodes and written as BS/pp. Bootstrap values >50% are shown, while only posterior probabilities >0.6 are shown. The tree is midpoint rooted. The scale bar shows the inferred number of amino acid substitutions per site. Sequences are

colored according to their taxonomic affiliation: Opisthokonta are in red, protists are in pink, Viridiplantae are in green, Bacteria are orange. We also stained branches according to their known function: ST3Gal in blue, ST6Gal in yellow, ST6GalNAc in brown, ST8Sia in purple.

Supplementary figure S3: **Bayesian phylogeny of the 180 ST sequences of the GT29 CAZy family and the 4 ST sequences of the GT42 CAZy family**. The consensus tree shown here was obtained with Phylobayes 4.1 with ML bootstrap values (BS) and bayesian posterior probabilities (pp) mapped onto the nodes and written as BS/pp. Bootstrap values >50% are shown, while only posterior probabilities >0.6 are shown. The tree is rooted with ST sequences of the GT42 CAZy family, since they are considered as the closest GT29 sequences [3] scale bar shows the inferred number of amino acid substitutions per site. Sequences are colored according to their taxonomic affiliation: Opisthokonta are in red, protists are in pink, Viridiplantae are in green, Bacteria are orange, GT42 sequences are in black. We also stained branches according to their known function: ST3Gal in blue, ST6Gal in yellow, ST6GalNAc in brown, ST8Sia in purple. Bacterial ST sequences of the GT29 and GT42 CAZy families are basal in the tree, indicating that the GT29 function appeared earlier in evolution than previously thought.

Supplemental figure S4: Sialin (SLC17A5) phylogenetic tree

The evolutionary history of the SLC17A5-related sequences was inferred using the ME. The optimal tree with the sum of branch length = 40.90219812 is shown. The percentage of replicate trees in which the associated taxa clustered together in the bootstrap test (350 replicates) are shown next to the branches. The tree is drawn to scale, with branch lengths in the same units as those of the evolutionary distances used to infer the phylogenetic tree. The evolutionary distances were computed using the JTT matrix-based method and are in the units of the number of amino acid substitutions per site. The ME tree was searched using the Close-Neighbor-Interchange (CNI) algorithm at a search level of 1. The NJ algorithm [1] was used to generate the initial tree. The analysis involved 74 aa sialin-related

sequences. All positions with less than 95% site coverage were eliminated and there were a total of 299 positions in the final dataset. Evolutionary analyses were conducted in MEGA7.0 [2]. The tree is midpoint rooted.

Supplemental figure S5: **Sialidases (Neu) phylogenetic tree.**

The evolutionary history of sialidase sequences was inferred using the ME method and the optimal tree with the sum of branch length = 22.28030272 is shown. The percentage of replicate trees in which the associated taxa clustered together in the bootstrap test (350 replicates) is shown next to the branches. The tree is drawn to scale, with branch lengths in the same units as those of the evolutionary distances used to infer the phylogenetic tree. The evolutionary distances were computed using the JTT matrix-based method and are in the units of the number of amino acid substitutions per site. The ME tree was searched using the CNI algorithm at a search level of 1. The NJ algorithm [1] was used to generate the initial tree. The analysis involved 45 aa sialidase-related sequences. All positions with less than 95% site coverage were eliminated and there were a total of 171 aa positions in the final dataset. Evolutionary analyses were conducted in MEGA7.0 [2]. The tree is midpoint rooted.

Supplemental figure S6: **Uridine diphospho-N-acetylglucosamine (UDP-GlcNAc) 2-epimerase phylogenetic tree**

The evolutionary history of the 2-epimerase-related sequences was inferred using the Minimum Evolution method and the optimal tree with the sum of branch length = 16.39485451 is shown. The percentage of replicate trees in which the associated taxa clustered together in the bootstrap test (350 replicates) are shown next to the branches. The tree is drawn to scale, with branch lengths in the same units as those of the evolutionary distances used to infer the phylogenetic tree. The evolutionary distances were computed using the JTT matrix-based method and are in the units of the number of amino acid substitutions per site. The ME tree was searched using the CNI algorithm at a search level of 1. The Neighbor-joining algorithm was used to generate the initial tree. The analysis involved 34

amino acid sequences. All positions with less than 95% site coverage were eliminated and there were a total of 283 positions in the final dataset. Evolutionary analyses were conducted in MEGA7.0 [2]. The tree is midpoint rooted.

Supplemental figure S7 : N-acetylneuraminate synthase (NANS) phylogenetic tree.

The evolutionary history of the NANS-related sequences was inferred using the ME method and the optimal tree with the sum of branch length = 18.94238793 is shown. The tree is drawn to scale, with branch lengths in the same units as those of the evolutionary distances used to infer the phylogenetic tree. The evolutionary distances were computed using the JTT matrix-based method [3] and are in the units of the number of aa substitutions per site. The ME tree was searched using the CNI algorithm at a search level of 1. The NJ algorithm [1] was used to generate the initial tree. The analysis involved 50 aa sequences, all positions with less than 95% site coverage were eliminated and there were a total of 210 positions in the final dataset. Evolutionary analyses were conducted in MEGA7.0 [2]. Bootstrap test =350 replicates. The tree is midpoint rooted.

Supplementary figure S8: **N-acetylneuraminate-9-phosphate phosphatase (NANP) phylogenetic tree.**

The evolutionary history was inferred using the ME method and the optimal tree with the sum of branch length = 17.65588876 is shown. The tree is drawn to scale, with branch lengths in the same units as those of the evolutionary distances used to infer the phylogenetic tree. The evolutionary distances were computed using the JTT matrix-based method and are in the units of the number of aa substitutions per site. The ME tree was searched using the CNI algorithm. The NJ algorithm [1] was used to generate the initial tree. The analysis involved 26 amino acid sequences. All positions with less than 95% site coverage were eliminated and there were a total of 143 positions in the final dataset. Evolutionary analyses were conducted in MEGA7.0 [2]. Bootstrap test =350 replicates. The tree is midpoint rooted.

Supplementary figure 9: CMP-sialic acid synthase (CMAS or CSS) phylogenetic tree.

The evolutionary history of CMAS-related sequences was inferred using the ME method and the optimal tree with the sum of branch length = 31.17236376 is shown. The tree is drawn to scale, with branch lengths in the same units as those of the evolutionary distances used to infer the phylogenetic tree. The evolutionary distances were computed using the JTT matrix-based method and are in the units of the number of aa substitutions per site. The ME tree was searched using the CNI algorithm at a search level of 1. The NJ algorithm [1] was used to generate the initial tree. The analysis involved 65 aa sequences, all positions with less than 95% site coverage were eliminated and there were a total of 153 positions in the final dataset. Evolutionary analyses were conducted in MEGA7.0 [2]. Bootstrap replicates = 350. The tree is midpoint rooted.

Supplementary figure 10: Nucleotide-sugar Transporters (NST) solute carrier family SLC35A phylogenetic tree.

The evolutionary history was inferred using the ME method and the optimal tree with the sum of branch length = 29.13303078 is shown. The tree is drawn to scale, with branch lengths in the same units as those of the evolutionary distances used to infer the phylogenetic tree. The evolutionary distances were computed using the JTT matrix-based method and are in the units of the number of amino acid substitutions per site. The ME tree was searched using the CNI algorithm at a search level of 1. The NJ algorithm [1] was used to generate the initial tree. The analysis involved 69 aa sequences. All positions with less than 95% site coverage were eliminated and there were a total of 199 positions in the final dataset. Evolutionary analyses were conducted in MEGA7.0 [2]. The tree is midpoint rooted.

Additional references for supplemental figure legends :

1. Saitou N, Nei M: **The neighbor-joining method: a new method for reconstructing phylogenetic trees**. *Mol Biol Evol* 1987, **4**(4):406-425.
2. Kumar S, Stecher G, Tamura K: **MEGA7: Molecular Evolutionary Genetics Analysis Version 7.0 for Bigger Datasets**. *Mol Biol Evol* 2016, **33**(7):1870-1874.
3. Huo L, Zhang H, Huo X, Yang Y, Li X, Yin Y: **pHMM-tree: phylogeny of profile hidden Markov models**. *Bioinformatics* 2017, **33**(7):1093-1095.

Supplemental figure S1

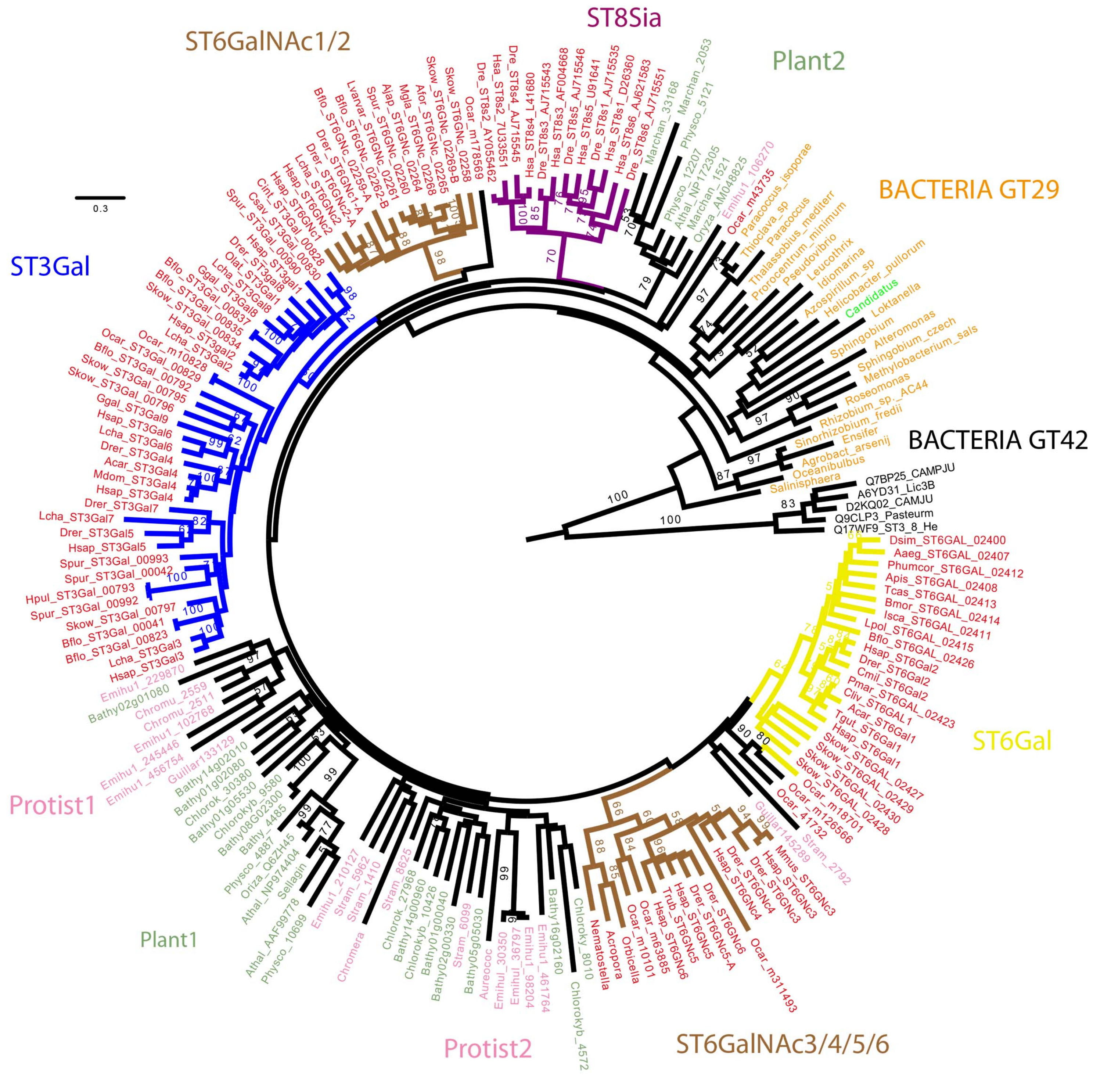

Supplementary figure S2

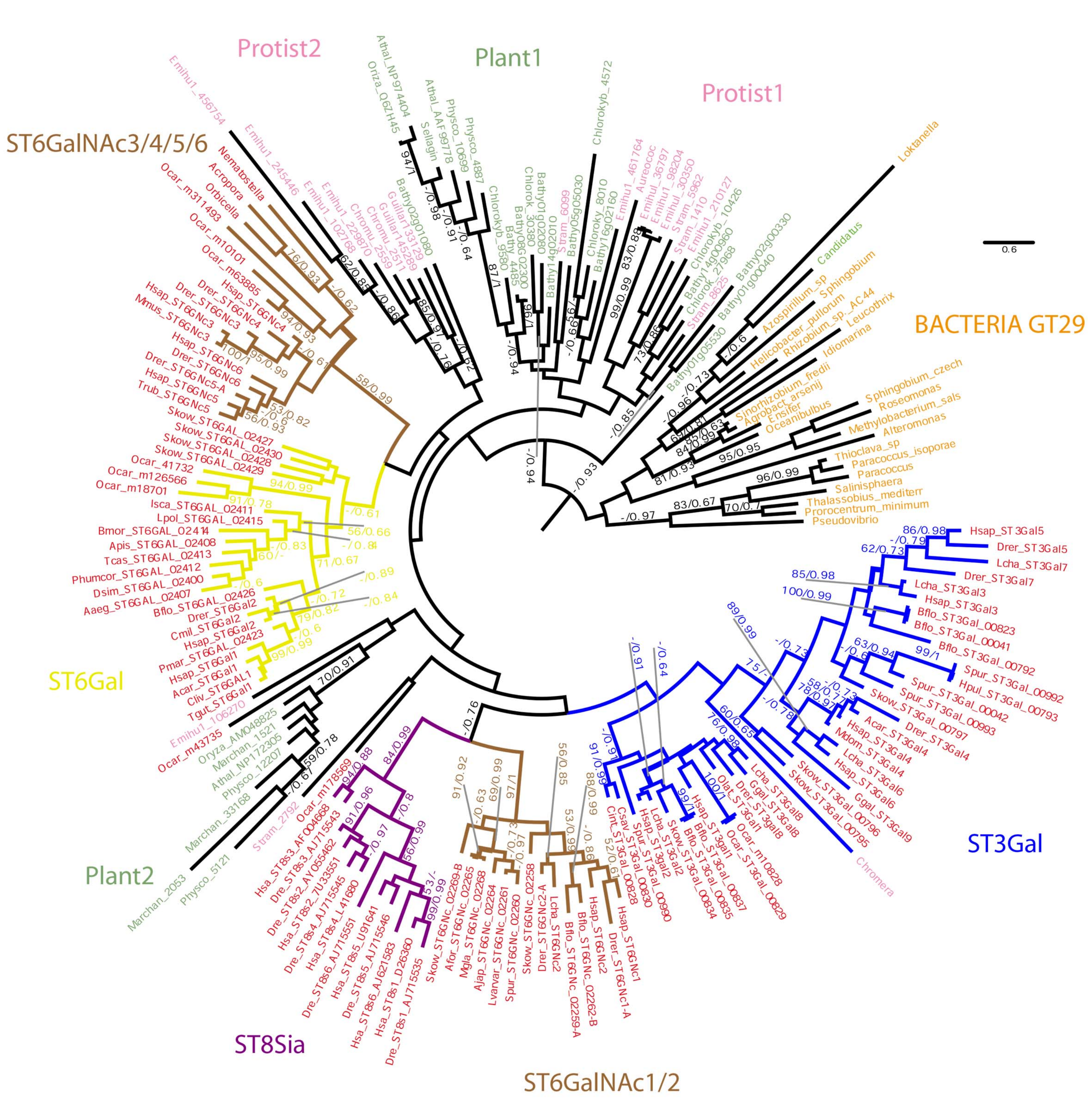

Supplementary figure S3

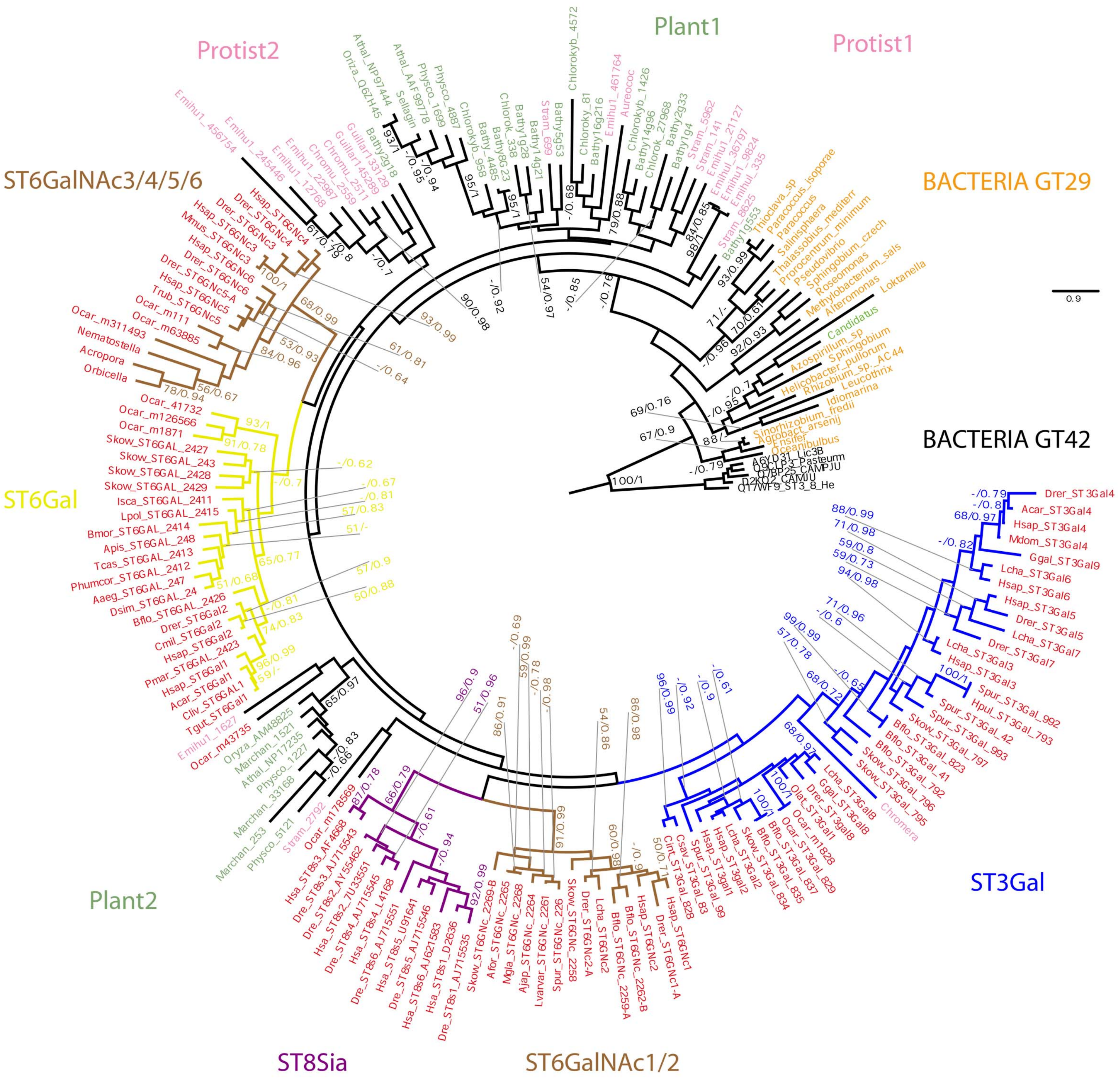

Supplementary S4: Sialic acid transporter SLC17A

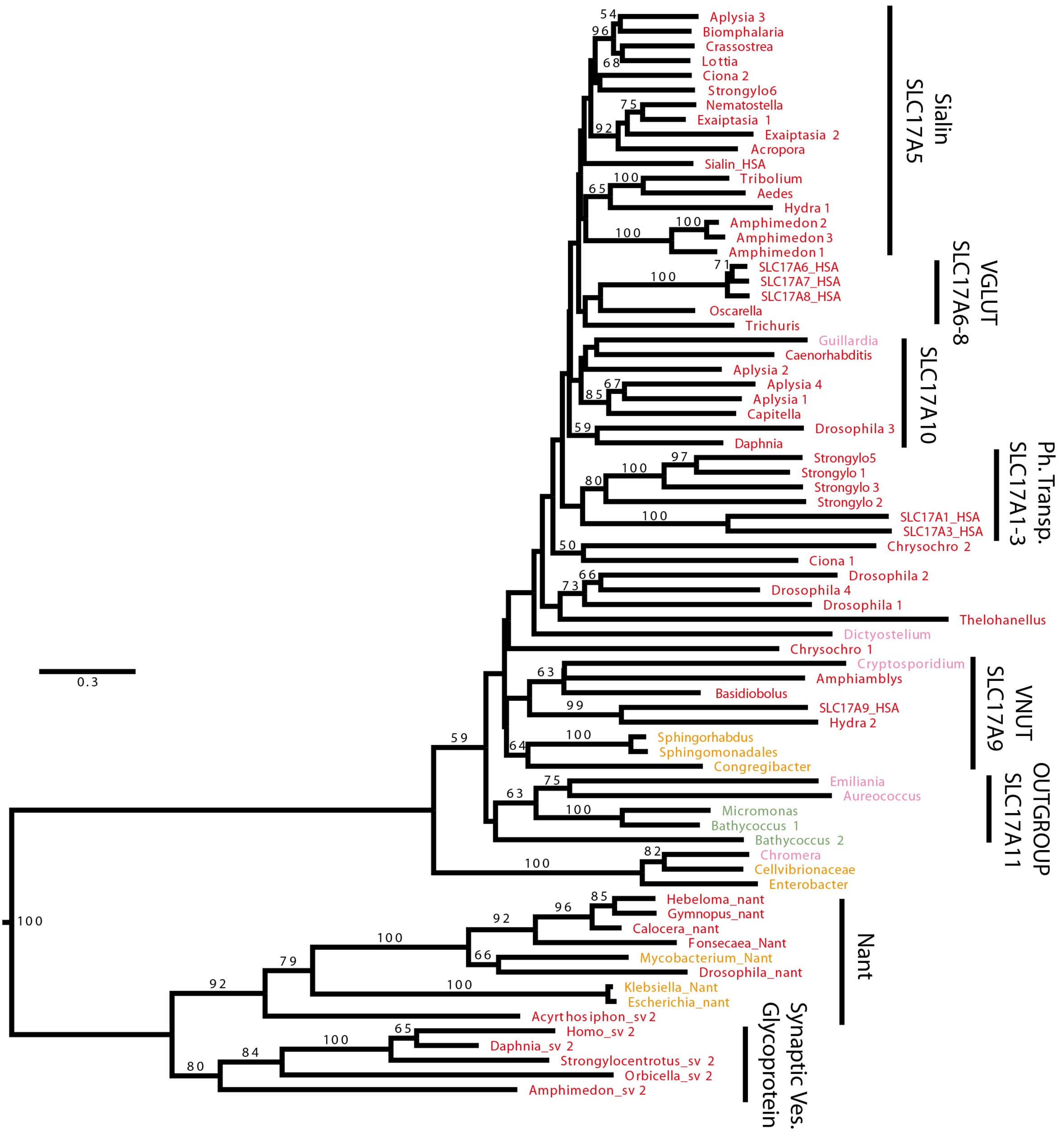

Supplemental figure S5: Sialidases (Neu)

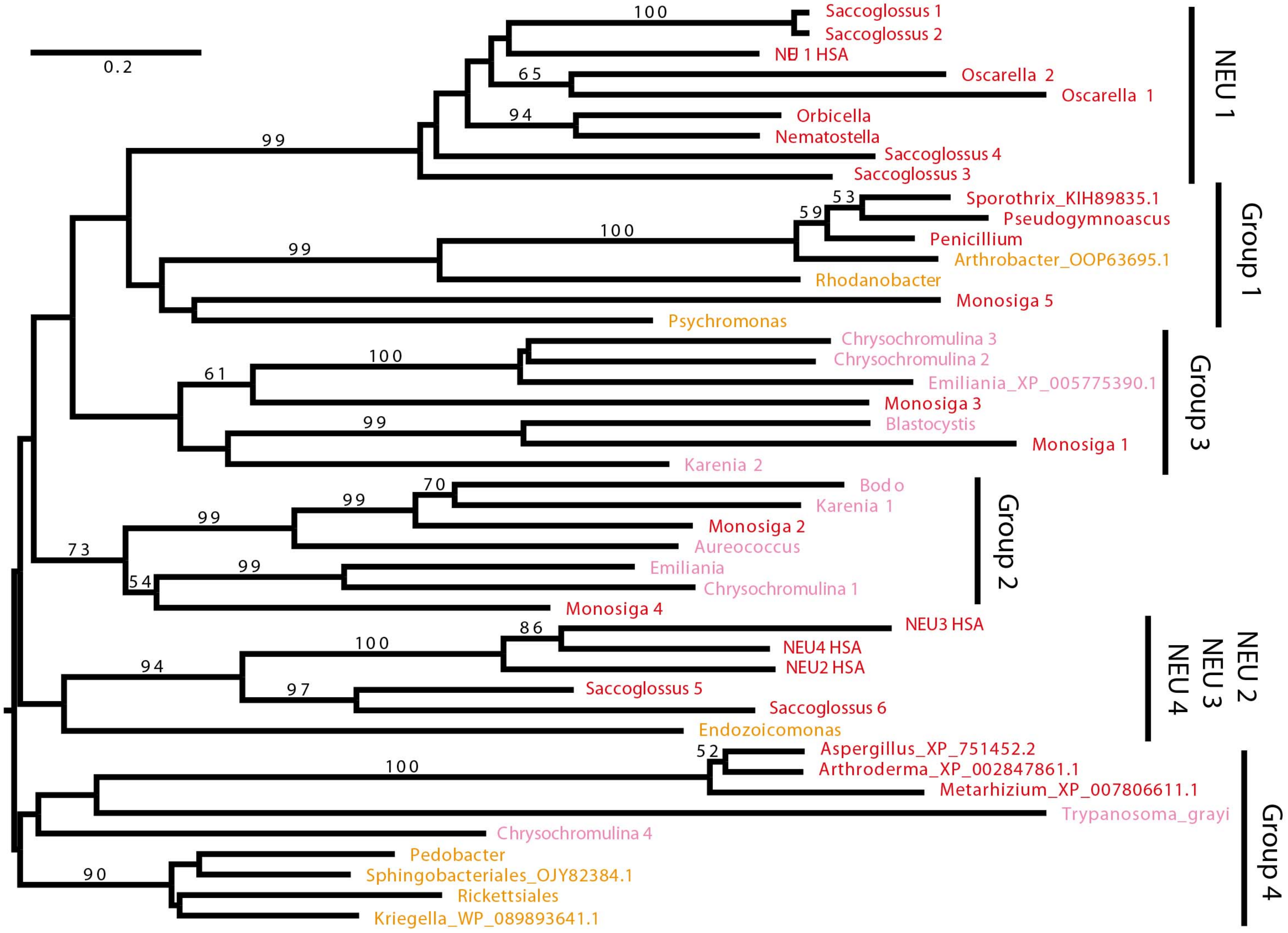

Supplementary figure S6: UDP-GlcNAc 2-epimerase

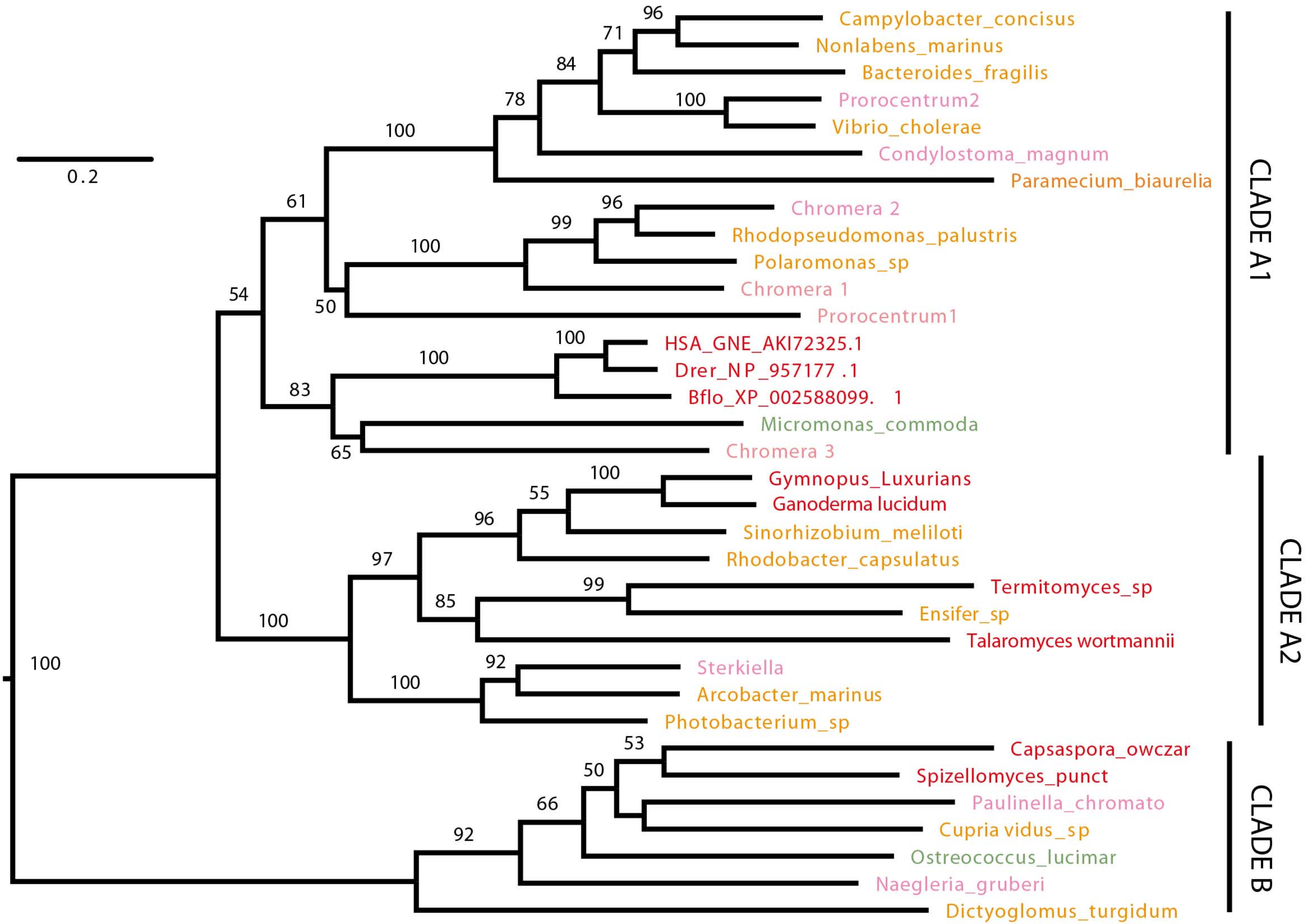

Supplementary figure S7: NANS

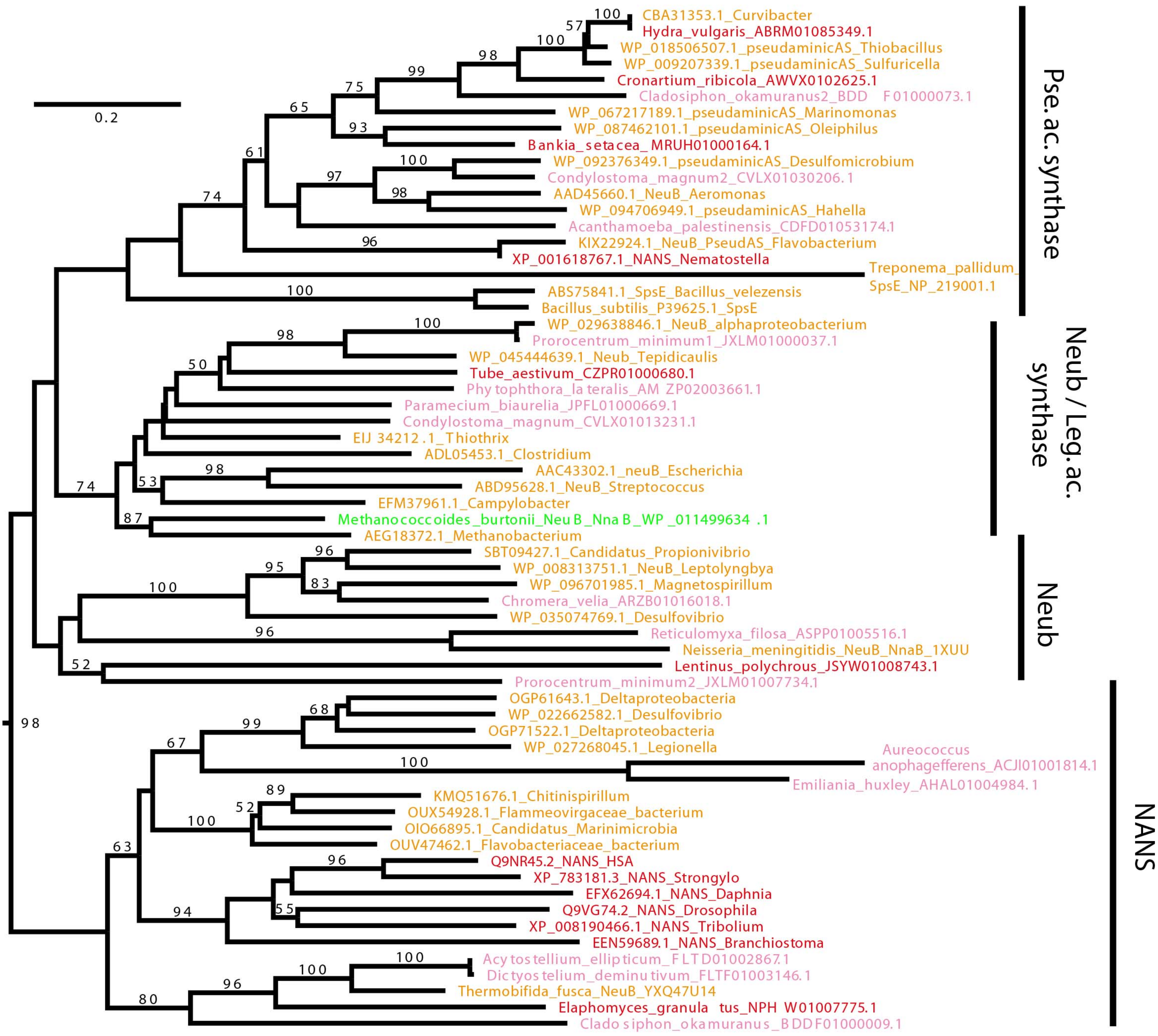

Supplementary figure S8: NANP

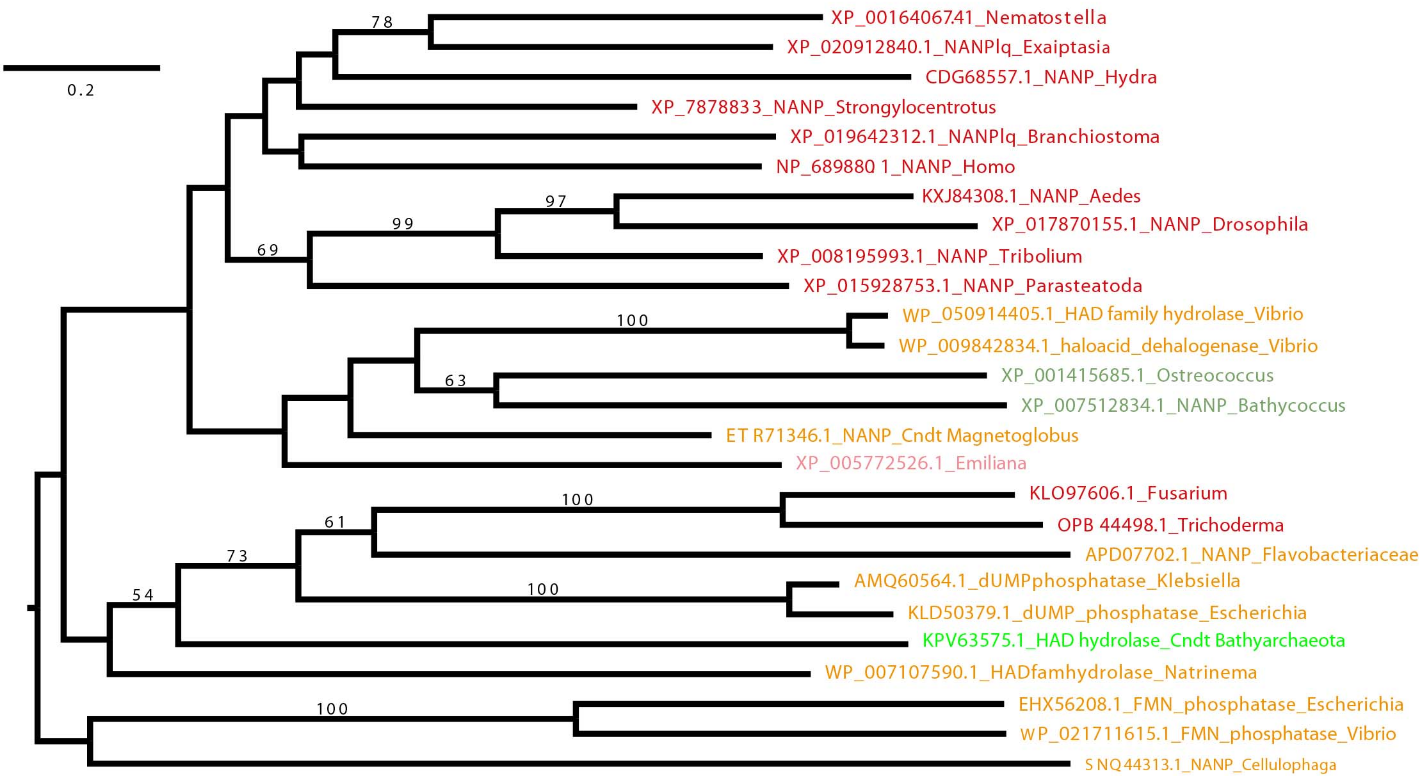

Supplemental figure S9: CMAS

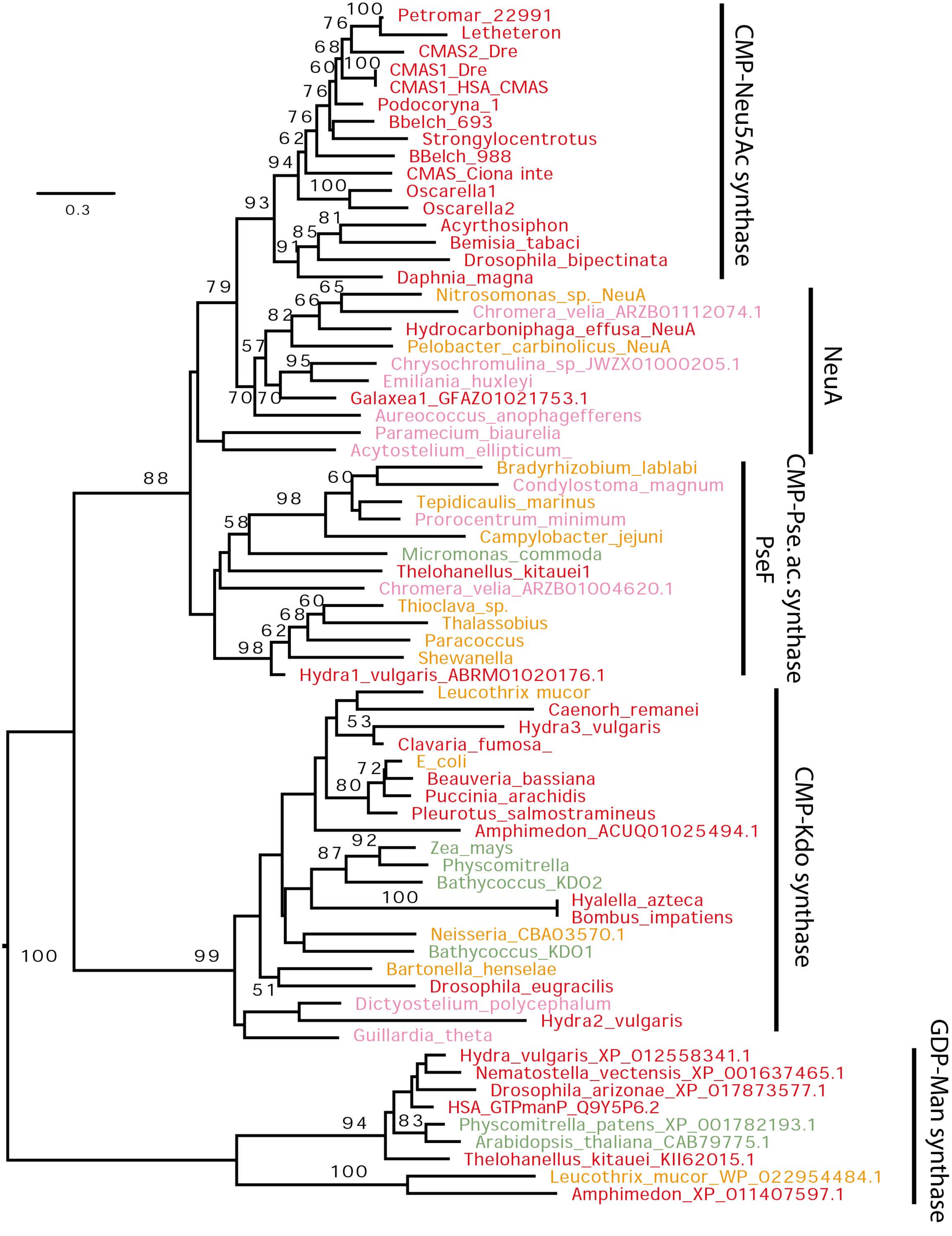

Supplemental figure S10: SLC35A transporter

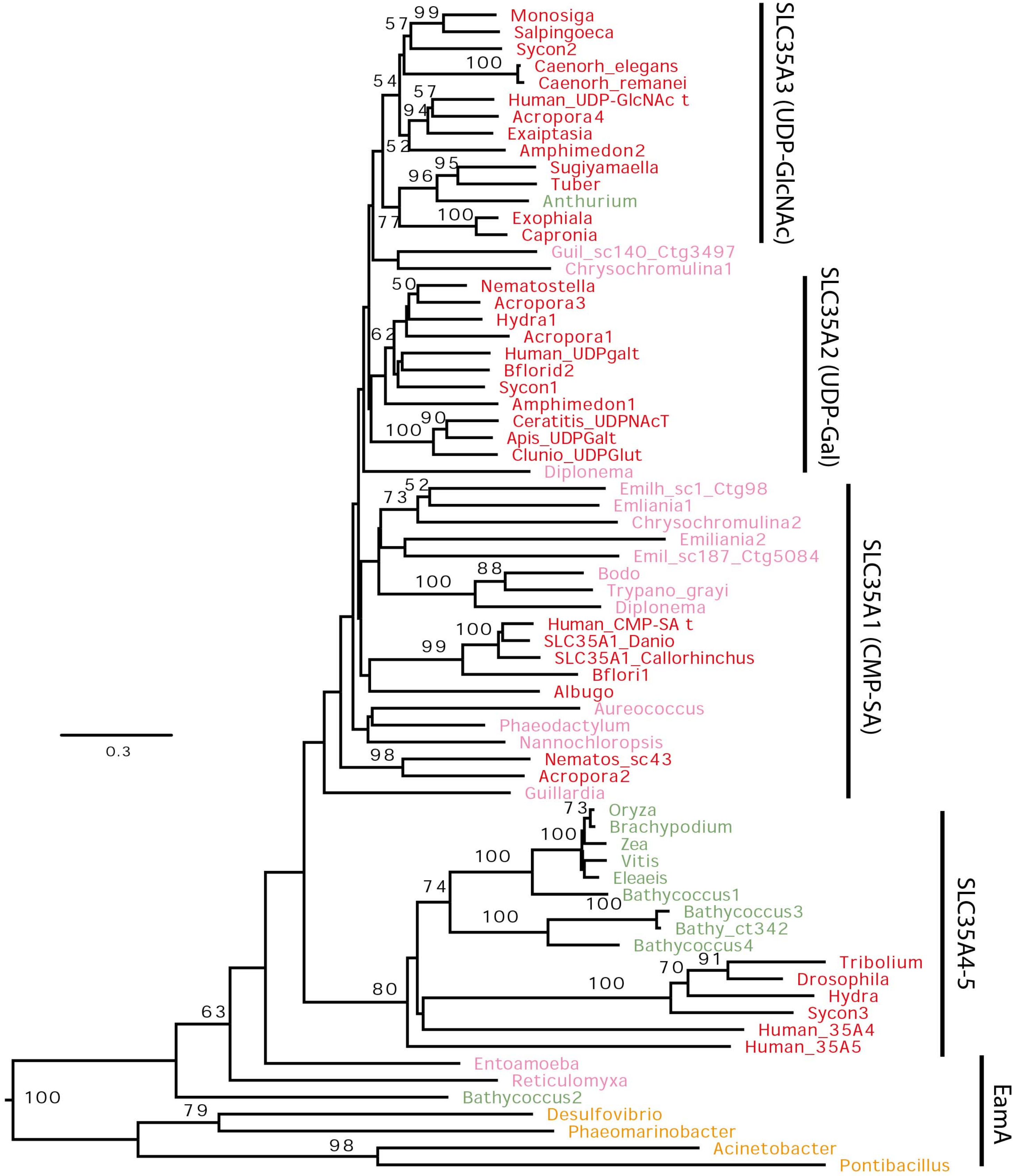

Supplement: Supplementary file 1 — Supplemental Dataset 1, Dataset 3, Dataset 4 and Supplemental Figures [file 41598_2018_20920_MOESM1_ESM.zip › Supplementary figures 2018.pdf]
